# Access to Unexplored 3D Chemical Space: *cis*‐Selective Arene Hydrogenation for the Synthesis of Saturated Cyclic Boronic Acids

**DOI:** 10.1002/anie.202206687

**Published:** 2022-07-04

**Authors:** Akash Kaithal, Tobias Wagener, Peter Bellotti, Constantin G. Daniliuc, Lisa Schlichter, Frank Glorius

**Affiliations:** ^1^ Westfälische Wilhelms-Universität Münster Organisch-Chemisches Institut Corrensstraße 40 48149 Münster Germany; ^2^ Westfälische Wilhelms-Universität Münster Westfälische Center for Soft Nanoscience (SoN) and Organisch-Chemisches Institut Busso-Peus-Str.10 48149 Münster Germany

**Keywords:** 3D Chemical Space, Hydrogen Bonding, Hydrogenation, Molecular Recognition, Rhodium

## Abstract

A new class of saturated boron‐incorporated cyclic molecules has been synthesized employing an arene‐hydrogenation methodology. *cis*‐Selective hydrogenation of easily accessible, and biologically important molecules comprising benzoxaborole, benzoxaborinin, and benzoxaboripin derivatives is reported. Among the various catalysts tested, rhodium cyclic(alkyl)(amino)carbene [Rh‐CAAC] (**1**) pre‐catalyst revealed the best hydrogenation activity confirming turnover number up to 1400 with good to high diastereoselectivity. A broad range of functional groups was tolerated including sensitive substituents such as −F, −CF_3_, and −silyl groups. The utility of the synthesized products was demonstrated by the recognition of diols and sugars under physiological conditions. These motifs can have a substantial importance in medicinal chemistry as they possess a three‐dimensional structure, are highly stable, soluble in water, form hydrogen bonds, and interact with diols and sugars.

## Graphical abstract

Novel and unexplored saturated cyclic boronic acids were synthesized using an arene hydrogenation methodology. Rhodium‐catalyzed *cis*‐selective hydrogenation of biologically important molecules comprising benzoxaborole, benzoxaborinin, and benzoxaboripin‐derivatives is reported. The utility of the synthesized molecules was shown by their high tendency to form intermolecular hydrogen bonding and molecular recognition under physiological conditions.

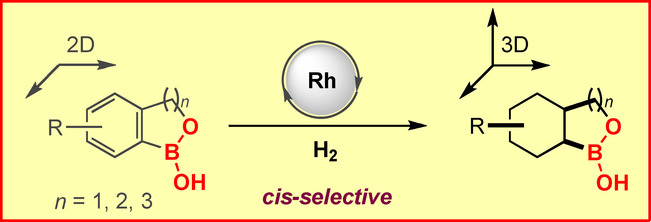

## Introduction

**Scheme 1:**
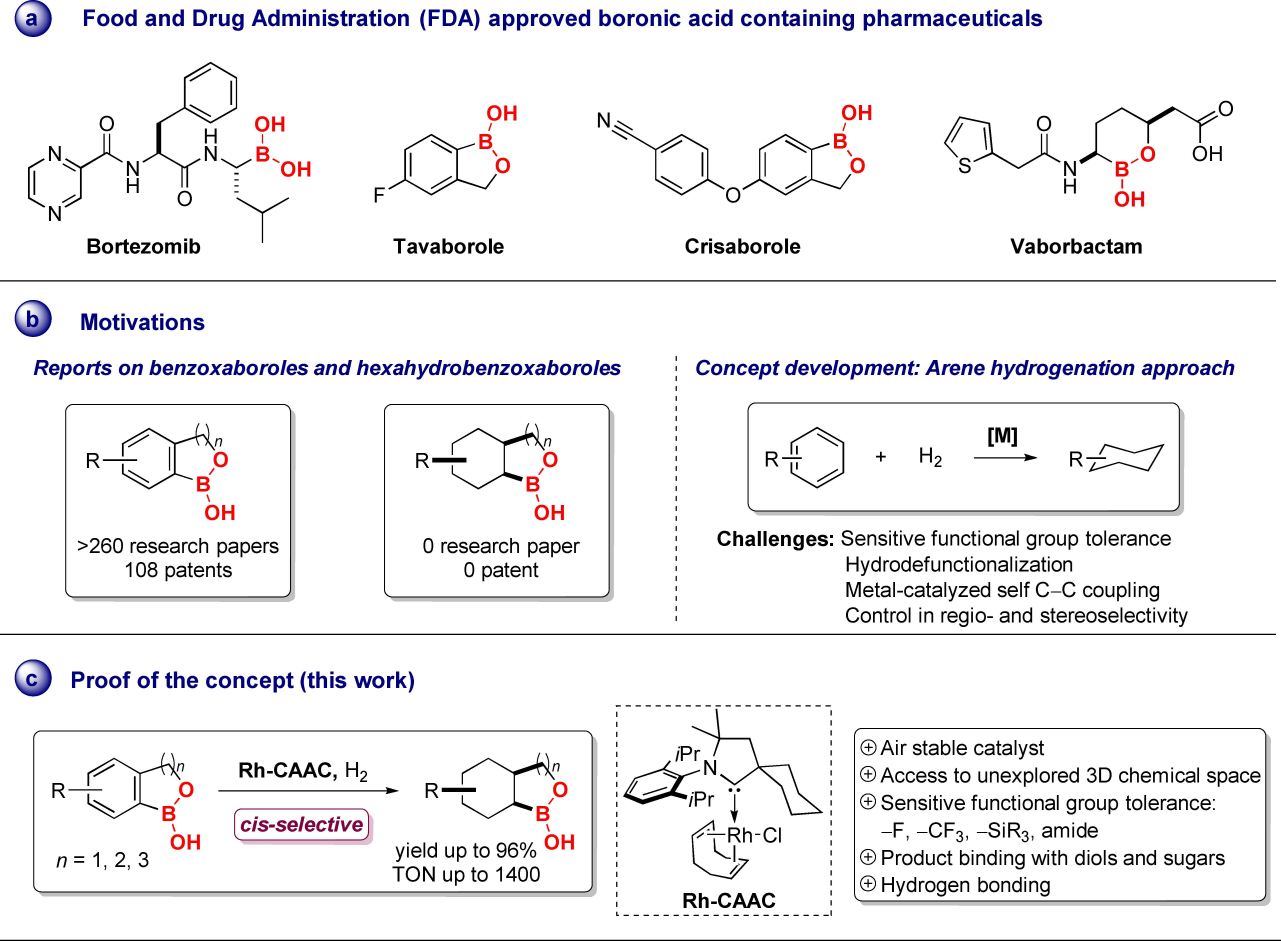
Development of the catalytic system for the hydrogenation of benzoxaborole derivatives.

Boronic acids and their analogues have been utilized in numerous biomedical applications, for example, in drug discovery and sensors.^[1]^ The latest pharmacopeia currently features a limited number of elements, in particular hydrocarbon frameworks.^[2]^ Almost all drugs incorporate nitrogen, and oxygen, as well as, to variable extents, sulfur, and fluorine.^[3]^ Since the FDA approved bortezomib, the first boron‐containing pharmaceutical used as a proteasome inhibitor,^[4]^ boron is considered an important element in pharmaceuticals and is gaining vast significance in medicinal applications.^[5]^ Thenceforth, additional four boron‐containing organic motifs were developed and revealed clinical utility (Scheme 1a). Among tested boron compounds in clinical trials, benzoxaborole and oxaborinin derivatives were intensively studied as they have shown high binding affinity with saccharides at physiological pH and react to the active centers of enzymes by noncovalent interactions, for example via dative bonds as well as by esterification.[^1a^, ^1e^, ^6^] These scaffolds can be found in antifungal drug tavaborole, crisaborole an antidermatitis drug, and vaborbactam, an effective β‐lactamase inhibitor.^[7]^ Additionally, these structures have also been examined to treat various diseases, several are in clinical trials and studied as antitrypanosomal,^[8]^ antibacterial,^[9]^ antimalarial,^[10]^ antiviral,^[11]^ and anti‐inflammatory agents.^[12]^ Benzoxaboroles have reported selective activity in inhibiting enzymes such as HCV NS3/4A serine protease,^[13]^ β‐lactamase,^[14]^ D,D‐carboxypeptidase,^[15]^ and PDE4 nucleotide phosphodiesterase.^[16]^ Therefore, since 1985, over 260 research papers, 41 reviews, and 108 patents have been published for the development of the chemistry of benzoxaborole derivatives.^[17]^

While benzoxaborole derivatives (two‐dimensional structure, 2D) are well‐explored and developed in drug discovery, to our best knowledge, we did not find any report where the reduced form of benzoxaboroles, hexahydrobenzoxaborole‐derivatives (three‐dimensional structure, 3D) were synthesized or studied (Scheme 1b). This can stem from the tedious multistep preparation of these molecules and the in situ formation of sensitive and difficult to handle carbon‐boron or boron‐oxygen bonds. It is well‐established that introducing the 3D structures into the organic framework constitutes an important value for applied sciences, for instance in medicinal chemistry.^[18]^ Advancing the chemical space from 2D to 3D can upgrade various molecular properties such as solubility, receptor‐ligand affinity and improve the drug potency.[^18a^, ^18c^] In this context, by comprehending 3D chemical space as well as important elements such as boron and oxygen, hexahydrobenzoxaborole derivatives might be of great interest in drug discovery and sensing applications.

Arene hydrogenation offers a direct pathway to leverage the formation of underexplored 3D structures comprising high stereo‐ and regioselectivity from simply accessible 2D frameworks.^[19]^ However, a primary challenge to face is the tolerance of sensitive functional groups directly attached to the aromatic ring during hydrogenation (Scheme 1b). Under reductive conditions, such sensitive substituents can influence the reactivity and drive towards the reaction termination or defunctionalization. Encouraged by the recent reports on arene hydrogenation by the group of Zheng on chemo‐selective hydrogenation of aromatic ketones,^[20]^ along with our group on *cis*‐selective hydrogenation of fluoroarenes,^[21]^ silylated^[22]^ and borylated^[23]^ arenes, we set to perform the hydrogenation of benzoxaborole, benzoxaborinin, and benzoxaboripin derivatives. The major challenge for the hydrogenation of these derivatives is to overcome the undesirable hydro‐defunctionalization, ring‐opening, or metal‐catalyzed homocoupling products. Crucial to unravel the desired reactivity is the recognition of a well‐suited catalyst that can lower the activation barrier for arene hydrogenation, resulting in mild reaction conditions and tolerance towards sensitive functional groups.

## Results and Discussion

Herein, we describe the synthesis of a new class of saturated boron‐incorporating cyclic organic molecules via the additive‐free *cis*‐selective hydrogenation of benzoxaborole, benzoxaborinin, and benzoxaboripin derivatives (Scheme 1c). At the outset, benzoxaborole (**5 a**) was chosen as a parent substrate for hydrogenation to identify the catalyst selection rationale (Table 1). Employing **5 a** (0.1 mmol), H_2_ (50 bar), 40 °C, and dichloromethane as a solvent, a series of previously established organometallic arene‐hydrogenation pre‐catalysts such as [Rh‐CAAC(COD)Cl] (CAAC=cyclic (alkyl)(amino)carbene, COD=1,5‐cyclooctadiene) (**1**),^[24]^ [Rh(COD)Cl]_2_ (**2**), [(*η* ^5^‐C_5_Me_5_)Rh(ppy)H] (**3**), and [Ru(*p*‐cymene)Cl_2_]_2_ (**4**) were chosen for hydrogenation. Our group,[^21a^, ^22^, ^23^, ^25^] Zeng,[^20^, ^26^] Bullock,^[27]^ and others^[28]^ have established several arene hydrogenation catalytic systems utilizing complex **1** and its analogs as pre‐catalysts. Lately, it is well‐established that complex **1**, in the presence of molecular sieves or silica and hydrogen, generates the supported Rh^0^‐nanoparticles in situ which further catalyze the arene‐hydrogenation reaction.[^23^, ^25a^] Complex **1** showed by far the best reactivity and selectivity from a range of organometallic precursors **1**–**4**, resulting in very high conversion (>99 %) and NMR yield (>99 %) towards the hydrogenated product **6 a** (Table 1, entries 1–4). However, other organometallic precursors such as complex **3**, employed by the group of Chirik for the hydrogenation of heteroarenes,^[29]^ and complex **4**, used by the group of Gunanathan for the hydrogenation of arenes^[30]^ did not indicate any hydrogenation activity for the hydrogenation of **5 a**. Next, to compare the reactivity of organometallic complexes with the reduced heterogeneous catalysts, various heterogeneous metal species were investigated. Among the tested heterogeneous catalysts, rhodium on charcoal (Rh/C) was found to be an effective catalyst, confirming >99 % conversion and 91 % yield towards **6 a** (Table 1, entry 5). Other heterogeneous catalysts either resulted in low reactivity or were inactive for the hydrogenation of **5 a** (Table 1, entries 6–8).

**Table 1:** Hydrogenation of benzoxaborole (**5 a**): Influence of catalyst precursor and reaction condition.^[a]^

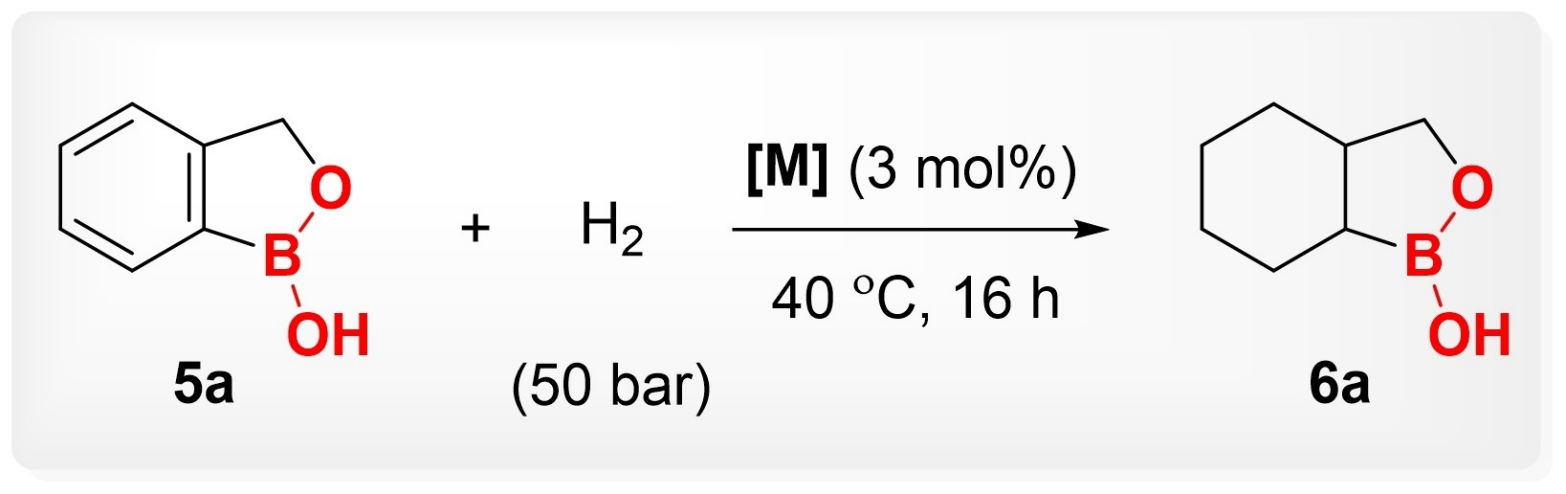			
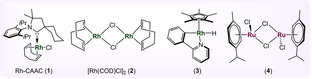			
Entry	Catalyst [M]	Conversion [%]	NMR Yield [%]
1^[b]^	[Rh‐CAAC] (**1**)	>99	>99
2	[Rh(COD)Cl]_2_ (**2**)	67	57
3	**3**	7	0
4	[Ru(*p*‐cymene)Cl_2_]_2_ (**4**)	14	0
5	Rh/C	>99	91
6	Pd/C	20	4
7	Pd(OH)_2_/C	0	0
8	Ru/C	35	20
9^[b,c]^	[Rh‐CAAC] (**1**)	>99	>99
10^[b,d]^	[Rh‐CAAC] (**1**)	>99	>99

[a] **5 a** (0.1 mmol), H_2_ (50 bar), [**M**] (3 mol %), CH_2_Cl_2_ (0.5 mL), *T*: 40 °C, and reaction time: 16 h. [b] 50 mg 4 Å molecular sieves was used. [c] 1 mol % of complex **1** was used. [d] 0.5 mol % of complex **1** was used.

Based on these results, further optimization was carried out using complex **1** and Rh/C. Complex **1** showed high reactivity while decreasing the catalyst loading from 3 to 0.5 mol % resulting in >99 % NMR yield to product **6 a** (Table 1, entries 9 and 10). Variation of reaction conditions such as catalyst loading, pressure, solvent, and temperature led to a standard set of conditions utilizing 1 mol % of complex **1**, 40 bar of H_2_, 40 °C temperature, 100 mg 4 Å molecular sieves, and dichloromethane as solvent (for detailed reaction optimization, see the Supporting Information). Complex **1** enabled tolerance to sensitive substituents such as −F, −CF_3_, and −silyl groups too. Unfortunately, Rh/C catalyst revealed attenuated reactivity under lower catalyst loading (1 mol %), giving 24 % yield of product **6 a** and did not show any activity for the above‐mentioned sensitive substituents (for details see the Supporting Information).

The reaction‐condition‐based sensitivity screening^[31]^ and the substrate scope were explored utilizing complex **1** with the optimized reaction conditions. Sensitivity screening indicated that the reaction is sensitive to high oxygen concentration and water, however, other reaction parameters did not substantially affect the product formation (Table 1, for details, see the Supporting Information). Focusing on the substrate scope, the hydrogenation of benzoxaborole (**5 a**) provided the hydrogenated product with the isolated yield of 92 % and 80 : 20 diastereomeric ratio (d.r.) (Table 2, entry **6 a**). Subsequently, we examined the hydrogenation of functionalized arenes. Initially, the substitution on the *C*5‐position of benzoxaborole was tested. Various electron‐donating and electron‐withdrawing functional groups were investigated. Reaction with methoxy‐ and hydroxy‐substituted benzoxaboroles resulted in 80 % and 90 % yield, respectively with high d.r. of 89 : 11 and 88 : 12, respectively (entries **6 b**, **6 c**). The free hydroxyl group present in the product can lead to further functionalization and guide towards the variety of products. The trifluoromethyl group was also well‐tolerated showing 69 % yield and a very high d.r. (92 : 8) (entry **6 d**). Representatively, the single‐crystal structure of the major isomer of product **6 d** was solved to distinctly establish that the hydrogenation is *cis*‐selective.^[32]^ Interestingly, hydrogenation of these molecules resulted in the formation of only two among all possible diastereomers.

**Table 2:**
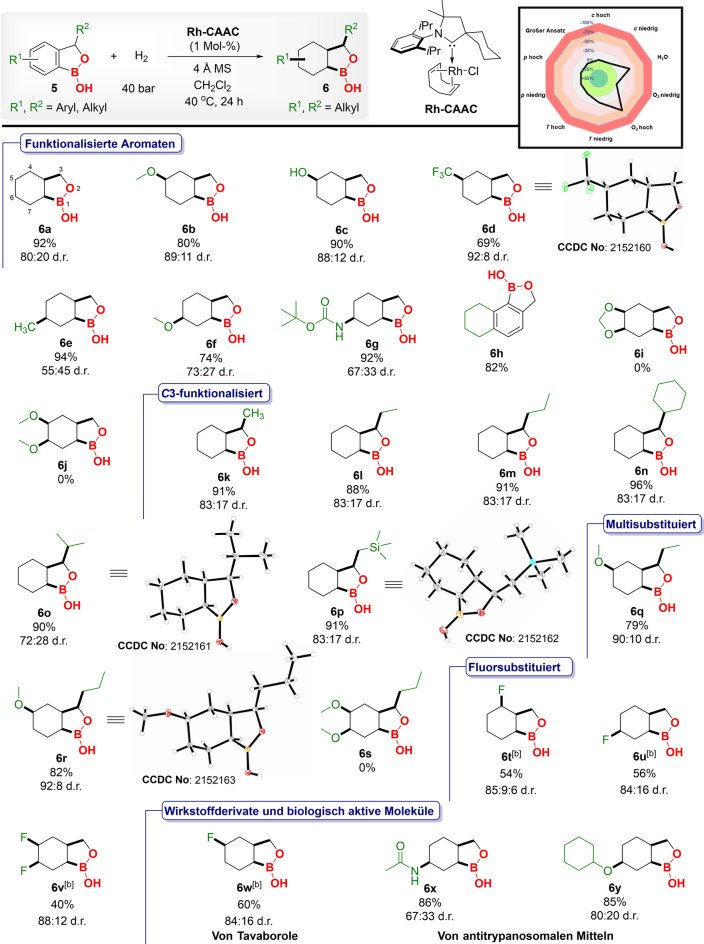
Substrate scope for the hydrogenation of benzoxaborole derivatives using complex **1** under optimized protocols and reaction‐condition based sensitivity assessment.^[a]^

[a] **5** (0.3 mmol), H_2_ (40 bar), **1** (1 mol %), *T*: 40 °C, 4 Å molecular sieves: 100 mg, CH_2_Cl_2_ (1 mL), and reaction time: 24 h, Yields correspond to the isolated product after performing column chromatography, d.r. was determined by NMR. The stereochemistry of products shown in table 2 refers to the relative configuration of the major diastereomer, products were obtained as racemic mixtures. [b] **5** (0.3 mmol), H_2_ (40 bar), **1** (3 mol %), *T*: 40 °C, 4 Å molecular sieves: 100 mg, hexane (1 mL), and reaction time: 16 h.

Afterwards, hydrogenation of *C*6‐substituted benzoxaboroles was examined. Hydrogenation of these compounds revealed lower d.r. when compared to substitution on *C*5‐position. High yield for methyl‐ (**6 e**) and methoxy‐containing (**6 f**) benzoxaboroles were observed. However, in the case of **6 e**, the d.r. somehow decreased to 55 : 45 ratio, while the methoxy‐substitution resulted in 73 : 27 d.r. Variation of hydrogen pressure did not enhance the reaction diastereoselectivity (for details see the Supporting Information). Furthermore, Boc‐protected amine benzoxaborole (**5 g**), which provides sites for orthogonal functionalization, was well‐hydrogenated in high yield (entry **6 g**). Interestingly, the hydrogenation of naphthalene ring incorporated‐oxaborole (**5 h**) resulted in selective single ring hydrogenation with a yield of 82 % (entry **6 h**). Unfortunately, the hydrogenation of tetra‐substituted arene rings did not show any product formation, likely due to excessive steric hindrance (entries **6 i**, **6 j**).

Subsequently, we focused on the hydrogenation of *C*3‐functionalized benzoxaboroles. Various *C*3‐substituted benzoxaboroles were tested for hydrogenation and revealed high reactivity and diastereoselectivity. Hydrogenation of *C*3‐methyl‐containing benzoxaborole resulted in 91 % yield and 83 : 17 d.r. (entry **6 k**). Similarly, *C*3‐ethyl, ‐propyl, ‐phenyl, and ‐*iso*‐propyl incorporated benzoxaboroles were also hydrogenated with the yield range of 88–96 %. Comprising this methodology, a sensitive silane‐substituted benzoxaborole (**5 p**) was well‐tolerated (entry **6 p**). Silyl groups are broadly studied in chemical synthesis and used as reagents for various organic transformations such as Fleming–Tamao oxidations,^[33]^ Hiyama couplings,^[34]^ or Brook rearrangements.^[35]^ Furthermore, multi‐substituted benzoxaboroles such as **5 q** and **5 r** were hydrogenated with excellent yield and high d.r to the corresponding products, **6 q** and **6 r**, respectively. However, like entry **6 i** and **6 j**, 3‐ethyl‐5,6‐dimethoxybenzo[*c*][1,2]oxaborol‐1(3*H*)‐ol (**5 s**) did not show any formation of hydrogenated product. The X‐ray crystal structures of a major isomer of product **6 o** (*C*3‐isopropyl), **6 p** (*C*3‐silane), and **6 r** were solved and established the product formation as *cis‐*selective.^[32]^

Saturated fluorinated carbocycles are emerging organic motifs in drug discovery, agrochemicals, and for the preparation of functional materials.^[36]^ Due to their high polarity of C−F bonds, they are purposely introduced into drug molecules to enhance their physiochemical properties.^[37]^ In general, the hydrogenation of fluorinated arenes leads to hydrodefunctionalization.[^21a^, ^38^] Interestingly, following our methodology, we were able to perform the hydrogenation of several fluorine‐substituted benzoxaboroles. The hydrogenation of *C*4‐ and *C*6‐fluoro‐substituted benzoxaboroles afforded the desired product in good yield and high d.r. (entries **6 t**, **6 u**). Similarly, poly‐fluorinated benzoxaborole was also hydrogenated yielding 40 % and 88 : 12 d.r. (entry **6 v**).

Next, we targeted the hydrogenation of drugs and biologically active molecules. Tavaborole (**5 w**), an antifungal drug, was hydrogenated to the corresponding hydrogenated product **6 w** in 60 % yield and 86 : 14 d.r. Furthermore, carboxamide benzoxaborole (**5 x**), and 6‐phenoxybenzo[*c*][1,2]oxaborol‐1(3*H*)‐ol (**5 y**), antitrypanosomal agents,^[39]^ are also well‐tolerated showing 86 % and 85 % yield, respectively.

Oxaborinin‐derivatives (six‐membered cyclic borane ring) found remarkable application in medicinal chemistry. Vaborbactam, an effective β‐lactamase inhibitor that has already been approved by FDA,^[7b]^ and taniborbactam, another β‐lactamase inhibitor^[40]^ encapsulates the oxaborinin moiety. Reasoning on the importance of oxaborinin derivatives, we have further examined the hydrogenation of functionalized benzoxaborinins. Employing the same reaction conditions, hydrogenation of benzoxaborinin (**7 a**) yielded 90 % product with 89 : 11 d.r (Table 3, entry **8 a**). Similarly, electron‐withdrawing trifluoromethyl‐ (**7 b**) and electron‐donating methoxy‐ (**7 c**) substituted derivatives could be well tolerated under these reaction conditions (entries **8 b**, **8 c**). The X‐ray crystal structure of the major isomer of product **8 b** was solved and established the product formation as *cis*‐selective.^[32]^ Afterwards, hydrogenation of benzoxaboripin (seven‐membered cyclic borane arene ring) was performed. By using the same reaction conditions, benzoxaboripin was hydrogenated in 69 % yield and 90 : 10 d.r. (entry **8 d**).

**Table 3:**
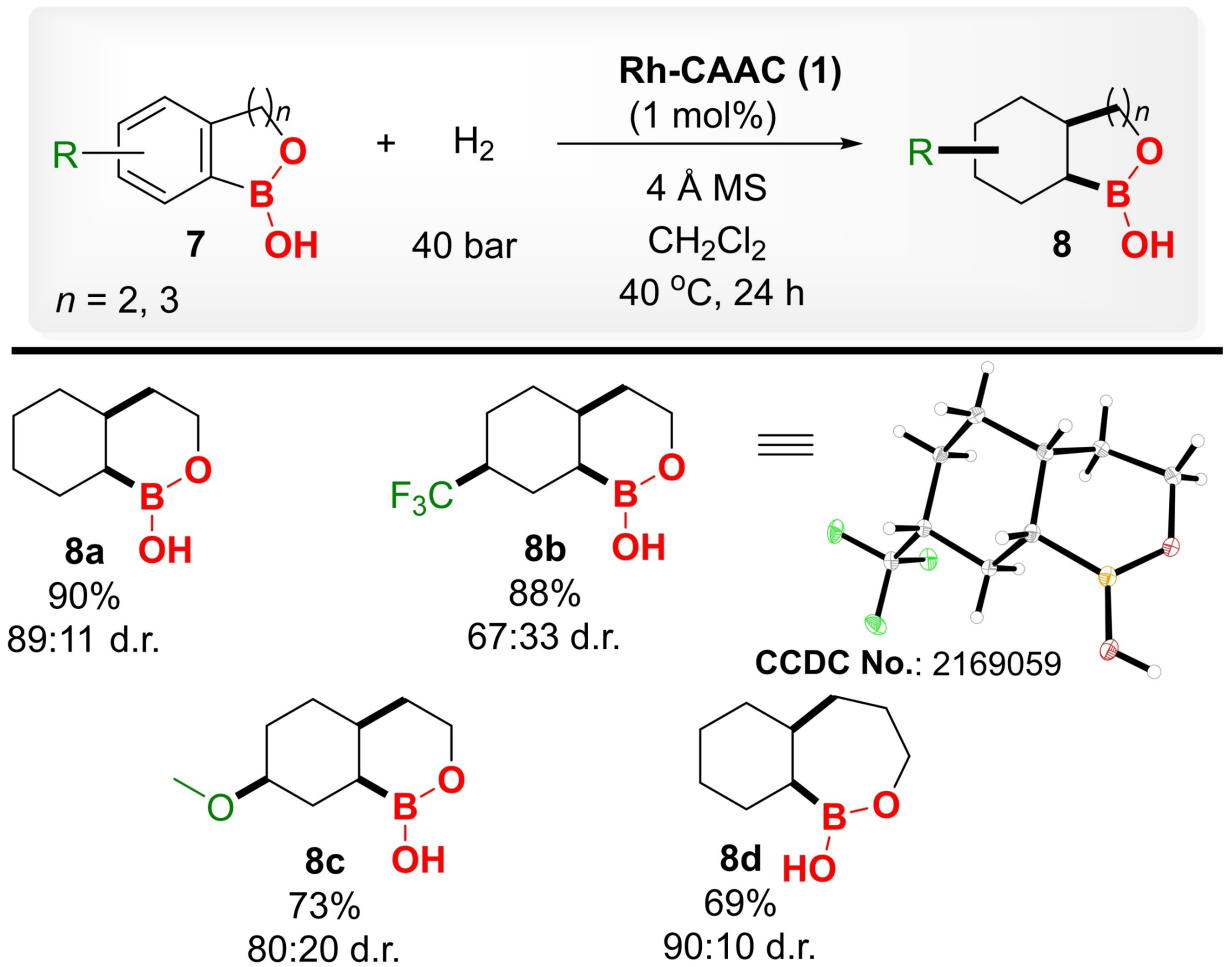
Substrate scope for the hydrogenation of benzoxaborinin, and benzoxaboripin derivatives using complex **1** under optimized protocols.^[a]^

[a] **7** (0.3 mmol), H_2_ (40 bar), **1** (1 mol %), *T*: 40 °C, 4 Å molecular sieves: 100 mg, CH_2_Cl_2_ (1 mL), and reaction time: 24 h, Yields correspond to the isolated product after performing column chromatography, d.r. was determined by NMR. The stereochemistry of products shown in Table 3 refers to the relative configuration of the major diastereomer, products were obtained as racemic mixtures.

To affirm the efficiency and robustness of the catalyst, the turnover number (TON) was optimized. Employing **5 a** as a standard substrate and using only 0.1 mol % of complex **1**, the reaction resulted in >99 % NMR yield with 999 TON. The best TON was obtained when the catalyst loading was decreased to 0.05 mol % showing a TON of 1400 to the desired hydrogenated product.

**Figure 1:**
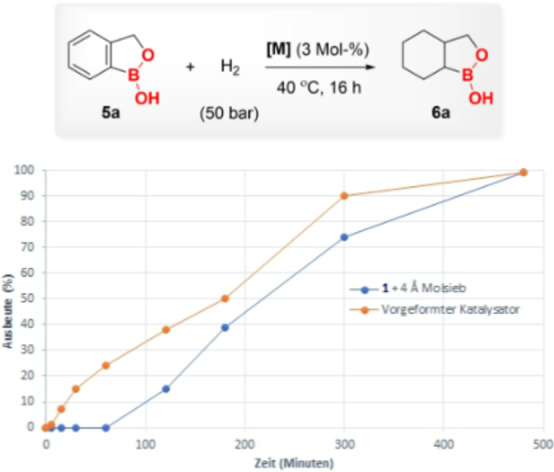
Yield/time profile for the hydrogenation of benzoxaborole (**5 a**) using complex **1**+4 Å molecular sieves (blue) or preformed Rh^0^ catalyst (orange) based on ^1^H NMR analysis of reaction mixtures. Reaction conditions: **5 a** (0.3 mmol), H_2_ (40 bar), **1** (1 mol %)+4 Å molecular sieves (100 mg) or preformed Rh^0^ catalyst (1 mol %) and CH_2_Cl_2_ (1 mL) were heated at 40 °C in a high‐pressure reactor for the indicated reaction time.

We further investigated the kinetic behavior of complex **1** for the hydrogenation of **5 a** (Figure 1). Analysis of the composition of the reaction mixture over‐time revealed that the catalyst required a significant induction period of one hour. The reaction yielded 15 % of the desired product **6 a** after two hours. The reaction rate sharply increased and reached a maximum between 2 and 3 h resulting in a 40 % yield. Subsequently, the reaction progressed continuously and showed >99 % yield after 8 h.

In particular, we have previously observed that the hydrogenation of arenes using complex **1** in the presence of molecular sieves proceeded via in situ formed supported rhodium(0) nanoparticles (NPs).[^25a^, ^27a^] Representatively, in all catalytic reactions, we observed the formation of dark black precipitate after the reaction, which indicated the formation of heterogeneous species in the reaction mixture. To prove whether the active catalytic species is heterogeneous, the standard hydrogenation reaction was performed in the presence of mercury, known to be an efficient poison for heterogeneous catalysts. No product formation was observed under these conditions. Similarly, the control fractional poisoning experiment with benzothiophene did not reveal the desired product formation. These studies indicate that the active catalytic species in the reaction are heterogeneous.

**Figure 2:**
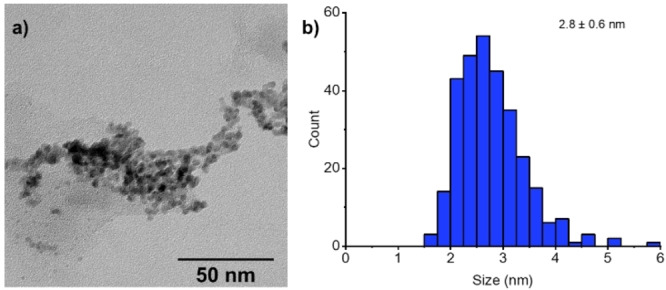
Characterization of the dark black residue after catalysis. a) TEM image of the isolated Rh nanoparticles after catalysis. b) Histogram showing the particle size distribution of Rh nanoparticles.

Next, we investigated the catalytic active species derived from complex **1**. The dark black residue was isolated after hydrogenation via filtration and examined for transmission electron microscopy (TEM) (Figure 2). The analysis of the black residue confirmed the formation of rhodium NPs. The average particle size was calculated to be 2.8±0.6 nm by analyzing the TEM particle size distribution (300 counts). The examination of black residue via infrared spectroscopy displays analogous characteristic bands as the CAAC ligand suggesting that NPs are stabilized by ligand (see the Supporting Information). The isolated black residue was utilized as a catalyst for the hydrogenation of **5 a** and showed the product formation with 85 % NMR yield, delivering comparable reactivity and selectivity (for details see the Supporting Information). Meanwhile, the reaction of the filtrate with fresh **5 a** did not reveal any product formation. Additionally, the preformed Rh^0^ catalyst was synthesized from complex **1** and molecular sieves in the presence of hydrogen and employed as a catalyst for the hydrogenation of **5 a** resulting in a 97 % NMR yield for product **6 a** (for details see the Supporting Information). The kinetic study using a preformed Rh^0^ catalyst displayed no induction period for the hydrogenation of **5 a**, which was observed when using the complex **1** as a pre‐catalyst (Figure 1). Subsequently, 1,10‐phenanthroline, an efficient poison for Ru and Rh NPs for hydrogenation reaction was used. Sub‐stoichiometric amounts of 1,10‐phenanthroline with respect to preformed Rh^0^ catalyst (0.25 equivalents) are capable of completely halting the activity of the catalyst (see Supporting Information).[^30^, ^41^] Based on these results and previous reports, it is established that the black dark residue formed after the hydrogenation reaction is the active catalyst and the reaction proceeds via in situ formed molecular sieves and CAAC ligand supported Rh^0^ nanoparticles.

**Figure 3:**
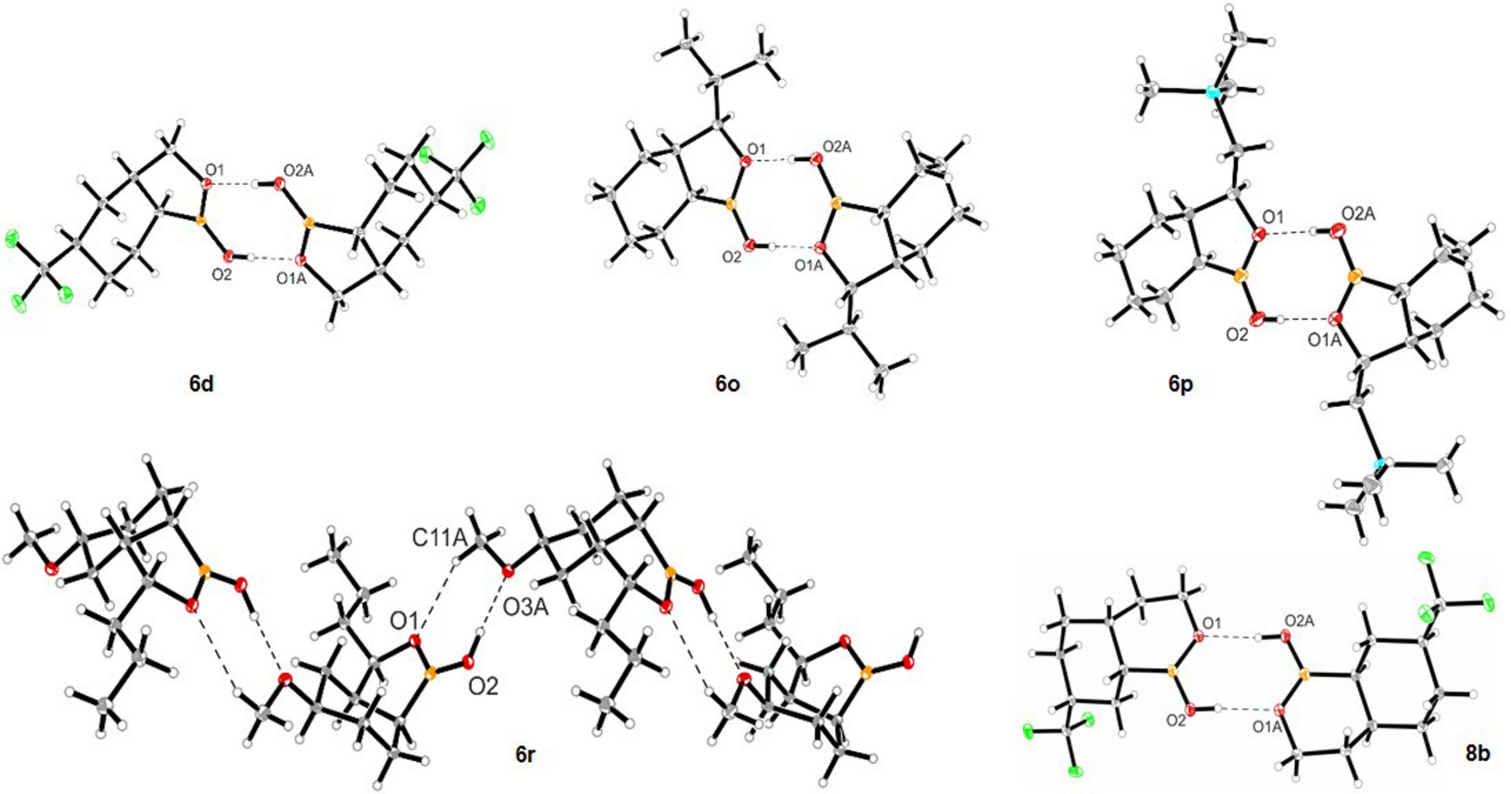
Intermolecular hydrogen bonding in the crystal structures of **6 d**, **6 o**, **6 p**, **6 r**, and **8 b**.

Having an efficient and established method in hand to access the saturated cyclic boronic acids, we further analyzed their synthetic and medicinal utility. Analyzing their physical properties, **6 a** showed high stability in monobasic phosphate buffer solution (pH 7.4), NMR analysis of **6 a** after 12 h and 30 days did not show any decomposition product. Comparing the solubility of **5 a** with **6 a**, **6 a** was three times more soluble in monobasic phosphate buffer solution (pH 7.4). Considering structural characteristics, hydrogen bonds play a crucial role in biochemistry and has great importance in drug discovery.^[42]^ The X‐ray crystal structure of **6 d**, **6 o**, **6 p** and **8 b** showed dimeric head‐to‐head intermolecular hydrogen bonding (O−H⋅⋅⋅O) between the hexahydrobenzoxaborole units (Figure 3). Interestingly, in the case of **6 r**, the presence of the methoxy group is more attractive for the formation of O−H⋅⋅⋅O hydrogen bonds interactions (Figure 3). The hydrogen bonds O−H⋅⋅⋅O were found between the hexahydrobenzoxaborole unit and methoxy group, supported by an additional weak C−H⋅⋅⋅O interaction. A zig‐zag chain is formed and represents head‐to‐tail interactions between the molecules of compound **6 r**. These behaviors and properties are essential for the molecules of pharmaceutical interest.[^18a^, ^18c^, ^42^]

**Figure 4:**
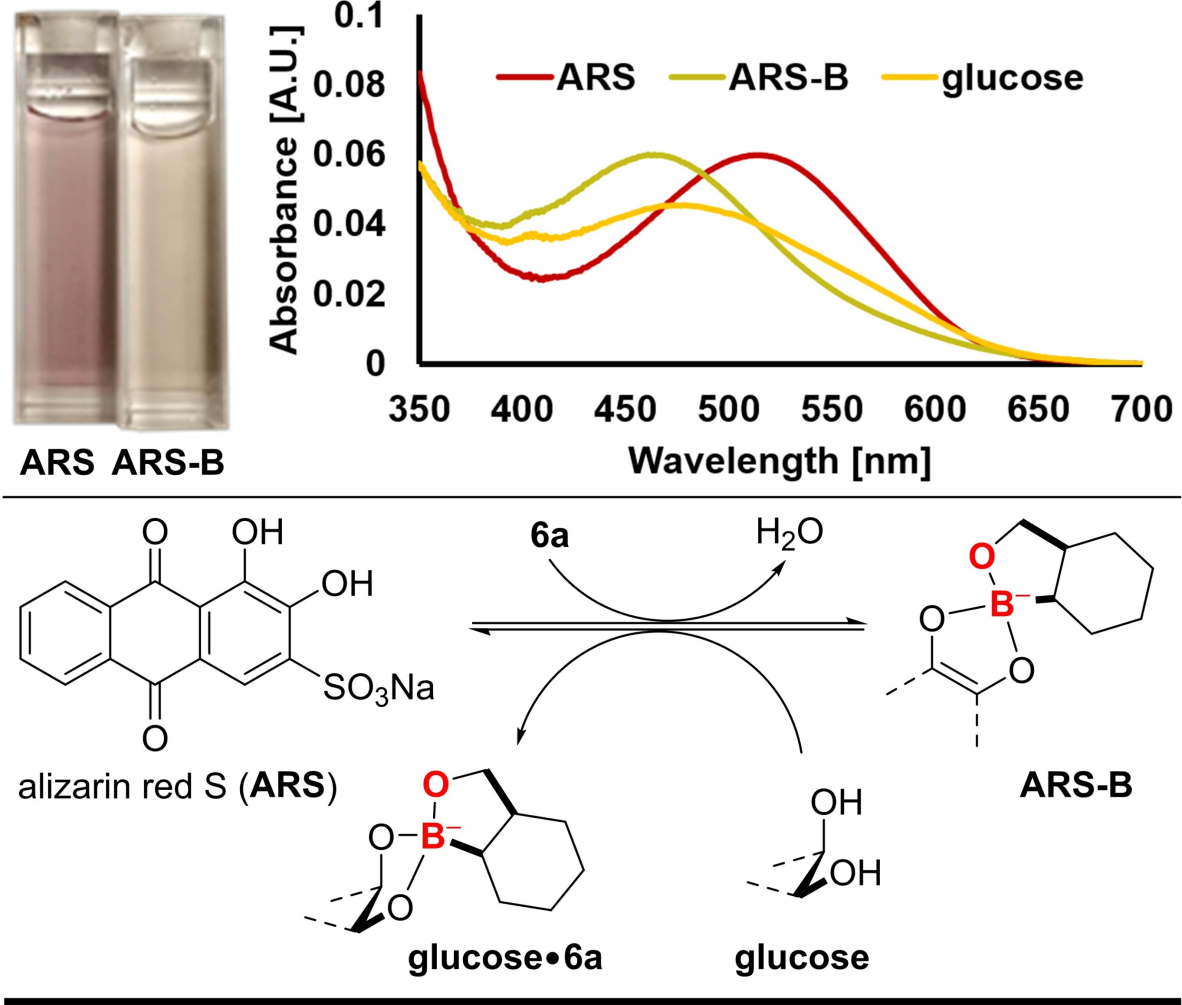
Application of **6 a** in the colorimetric detection of diol systems.

Under physiological conditions, the selective recognition of carbohydrates by small molecules is one of the arduous challenges in chemical biology.^[43]^ The problem lies with the competition between various hydroxyl group in carbohydrates and the excess amount of bulk solvent, water. Lately, phenylboronic acid and benzoxaborole were studied for sugar interaction under physiological conditions.^[44]^ Next, to affirm that our synthesized molecules are also prone to make complex with diols and sugars, **6 a** was further examined for binding studies. Initially, we focused on NMR studies to analyze the interaction between sugars and **6 a** (details see the Supporting Information). The NMR analysis clearly indicated the complex formation of **6 a** with d‐glucose, d‐fructose, and methyl‐α‐d‐glucopyranoside. Subsequently, we followed Wang's qualitative colorimetric assay based on the competitive removal of alizarin red S (ARS) (details see the Supporting Information).^[45]^ In the presence of **6 a**, ARS absorption maximum underwent hypsochromic shift (red line, *λ*_max_^ARS^=514 nm; yellow line, *λ*_max_^ARS‐B^=463 nm), as also visible from the color transition from cherry red to yellow (Figure 4). This result clearly indicated that **6 a** has high binding affinity for ARS (for details see the Supporting Information). The competitive binding of **6 a** between ARS and diol systems offered an indirect colorimetric probe for the detection of sugars. Upon addition of d‐glucose, the bathochromic shift of the absorption maximum (green line, *λ*_max_=479 nm) indicated equilibrium perturbation towards unbound ARS, thus probing the formation of d‐glucose **6 a** complex species.

## Conclusion

In conclusion, we have developed an additive‐free catalytic arene hydrogenation reaction which can straightforwardly produce a new class of saturated cyclic boronic acids with different ring sizes employing rhodium‐CAAC (**1**) as a catalyst. The reaction displays an impressively broad substrate scope providing high to excellent yields and moderate to high *cis*‐selectivity including the tolerance of sensitive functional groups such as −F, −CF_3_, and −SiR_3_. The maximum TON of 1400 was achieved for the hydrogenation of benzoxaborole. The overall transformation is atom economical as no side or byproducts were observed. These compounds were found to be stable and soluble in monobasic phosphate buffer solution and water under physiological conditions. The most critical finding is their tendency to form hydrogen bonding and good binding with diols and sugars. Overall, we envisage that these molecules have great potential in medicinal chemistry, as they provide direct access to 3D motifs, high stability, hydrogen bonding, and good binding with sugars and diols. Given the compelling importance of these 3D structures, additional biological studies (e.g. binding with proteins, nucleic acids, and calculation of association constant with sugars and diols) emerge very promising.

## Conflict of interest

The authors declare no conflict of interest.

1

## Supporting information

As a service to our authors and readers, this journal provides supporting information supplied by the authors. Such materials are peer reviewed and may be re‐organized for online delivery, but are not copy‐edited or typeset. Technical support issues arising from supporting information (other than missing files) should be addressed to the authors.

Supporting Information

## Data Availability

The data that support the findings of this study are available in the Supporting Information of this article.
