# Supplementary material for: Access to Unexplored 3D Chemical Space: cis‐Selective Arene Hydrogenation for the Synthesis of Saturated Cyclic Boronic Acids
Source: Angew Chem Int Ed Engl. 2022 Jul 4;61(32):e202206687. doi: 10.1002/anie.202206687 (PMC9400866; doi:10.1002/anie.202206687)
Supplement: Supplementary file 1 — Supporting Information [file ANIE-61-0-s001.pdf]

Angewandte Chemie
Eine Zeitschrift der Gesellschaft Deutscher Chemiker

Supporting Information

Access to Unexplored 3D Chemical Space: *cis*-Selective Arene Hydrogenation for the Synthesis of Saturated Cyclic Boronic Acids

*A. Kaithal, T. Wagener, P. Bellotti, C. G. Daniliuc, L. Schlichter, F. Glorius**

Table of Contents

1. General Methods ... 4

2. Synthesis of benzoxaborole derivatives using boronic acids ... 5

3. Synthesis of substituted 2-(4,4,5,5-tetramethyl-1,3,2-dioxaborolan-2-yl)benzaldehyde from substituted 2-bromobenzaldehyde ... 7

4. Synthesis of benzoxaborole derivatives from substituted 2-(4,4,5,5-tetramethyl-1,3,2-dioxaborolan-2-yl)benzaldehyde ... 10

5. Synthesis of benzoxaborole derivatives from the reaction of substituted 2-(4,4,5,5-tetramethyl-1,3,2-dioxaborolan-2-yl)benzaldehyde and Grignard reagents ... 13

6. Synthesis of 6-nitrobenzo[*c*][1,2]oxaborol-1(3*H*)-ol ... 18

7. Synthesis of 6-aminobenzo[*c*][1,2]oxaborol-1(3*H*)-ol ... 18

8. Synthesis of *tert*-butyl (1-hydroxy-1,3-dihydrobenzo[*c*][1,2]oxaborol-6-yl)carbamate (5g) ... 18

9. Synthesis of *N*-(1-hydroxy-1,3-dihydrobenzo[*c*][1,2]oxaborol-6-yl)acetamide (5x) ... 19

10. Synthesis of 6-phenoxybenzo[*c*][1,2]oxaborol-1(3*H*)-ol (5y) ... 19

11. Synthesis of functionalized 3,4-dihydro-1*H*-benzo[*c*][1,2]oxaborinin-1-ol (Benzoxaborinin derivatives) ... 20

12. Synthesis of 4,5-dihydrobenzo[*c*][1,2]oxaborepin-1(3*H*)-ol (7d) (Benzoxaboripin) ... 24

13. Synthesis of catalysts ... 25

14. Catalyst optimization for the hydrogenation of benzoxaborole (5a) ... 25

15. Temperature optimization for the hydrogenation of benzoxaborole (5a) using Rh-CAAC (1) ... 26

16. Pressure optimization for the hydrogenation of benzoxaborole (5a) using Rh-CAAC (1) ... 26

17. Solvent optimization for the hydrogenation of benzoxaborole (5a) using Rh-CAAC (1) ... 27

18. Effect of pressure in diastereomeric ratio for the hydrogenation of benzoxaborole derivatives using Rh-CAAC (1) ... 28

  18.1. Hydrogenation of benzoxaborole (5a) at different pressures ... 28

  18.2. Hydrogenation of 6-methylbenzo[c][1,2]oxaborol-1(3H)-ol (5e) at different pressures ... 28

  18.3. Hydrogenation of 3-methylbenzo[c][1,2]oxaborol-1(3H)-ol (5k) at different pressures ... 29

  18.4. Hydrogenation of 3-isopropylbenzo[c][1,2]oxaborol-1(3H)-ol (5o) at different pressures ... 29

19. Evaluation of Rhodium on carbon (Rh/C) as a catalyst for the hydrogenation of benzoxaborole derivatives ... 30

20. General procedure for the hydrogenation of benzoxaborole derivatives ... 31

21. General procedure for the hydrogenation of benzoxaborinin derivatives ... 44

22. General procedure for the hydrogenation of benzoxaboripin ... 47

23. Mechanistic Experiments ... 48

  23.1. Yield/time profile experiments for the hydrogenation of benzoxaborole (5a) using Rh-CAAC (1) ... 48

  23.2. Yield/time profile experiments for the hydrogenation of benzoxaborole (5a) using preformed [Rh] on 4 Å MS catalyst ... 49

23.3. Filtration tests 50
23.4. Poisoning Experiments 51
23.5. Analysis of molecular sieves supported rhodium(0) nanoparticles derived from Rh-CAAC (1) and 4Å MS using TEM analysis 53
23.6. Infrared spectroscopy measurements 54
24. Sensitivity Assessment 55
25. Single Crystal X-Ray Diffraction Studies 57
25.1. X-ray crystal structure analysis of 6d (glo10173) 57
25.2. X-ray crystal structure analysis of 6o (glo10256) 59
25.3. X-ray crystal structure analysis of 6p (glo10234) 61
25.4. X-ray crystal structure analysis of 6r (glo10254) 63
25.5. X-ray crystal structure analysis of 7b (glo10313) 65
25.6. X-ray crystal structure analysis of 8b (glo10318) 66
26. ^{1}H NMR binding studies of 6a with *D*-glucose, *D*-fructose, and methyl-α-*D*-glucopyranoside 68
27. Colorimetric assay and competitive binding experiment 72
28. Stability of compound 6a in monobasic phosphate buffered D_2O solution (pH = 7.4) 73
29. Spectral data of the starting materials 76
30. Spectral data of the products 135
31. Representative NMR spectra for the determination of diastereomeric ratio 176
32. Representative NMR spectra at different pressures for the hydrogenation of benzoxaborole derivatives to determine the diastereomeric ratio (Table S4-S7) 187
33. NMR spectra for the hydrogenation of benzoxaborole derivatives using Rhodium on carbon (Rh/C) as a catalyst 189
34. NMR spectra for the hydrogenation of 5a using filtration test 191
References 192

1. General Methods

All catalytic and stoichiometric reactions were performed under argon atmosphere using standard Schlenk and glovebox techniques unless otherwise noted. The used glassware was dried under vacuum at high temperatures, evacuated, and refilled with argon at least three times. The solvents were purified using solvent purification systems and were stored and handled under argon. Chemicals were purchased from Sigma-Aldrich, Alfa-Aesar, abcr, Acros Organics, TCI chemicals and used without further purification. NMR-spectra were recorded on Bruker NEO 400, AgilentDD2 500 or AgilentDD2 600 in the indicated solvents and temperatures with the chemical shifts (δ) given in ppm relative to TMS and the coupling constants (J) in Hz. The solvent signals were used as references and the chemical shifts converted to the TMS scale (Acetonitrile-d_3: δ_H = 1.94 ppm and δ_C = 118.3, 1.3 ppm; $CDCl_3$: δ_H = 7.26 ppm and δ_C = 77.1 ppm; CD_2Cl_2: δ_H = 5.32 ppm and δ_C = 54.00 ppm; C_6D_6: δ_H = 7.16 ppm and δ_C = 128.1 ppm; THF-d_8: δ_H = 1.72 ppm, 3.58 ppm and δ_C = 67.2 ppm, 25.3 ppm; toluene-d_8: δ_H = 2.08, 6.97, 7.01, 7.09 ppm and δ_C = 137.5, 128.9, 127.9, 125.1, 20.4 ppm; DMSO-d_6: δ_H = 2.50 ppm and δ_C = 39.5 ppm).[1]

Analytical thin layer chromatography (TLC) was performed on silica gel 60 F254 aluminum plates (Merck). TLC plates were visualized by exposure to short wave ultraviolet light (254 nm, 366 nm) and were dipped into a solution of $KMnO_4$. Flash chromatography was performed on Acros Organics silica gel (35-70 mesh) under a positive pressure of air, eluting with the specified solvent system.

ESI mass spectra were recorded on a Bruker Daltonics MicroTof spectrometer. APCI mass spectra were recorded on a Thermo Fisher Scientific Orbitrap LTQ XL.

Transmission electron microscopy (TEM) experiments were performed using a Thermo Fisher Scientific FEI Themis G3 60-300 transmission electron microscope (Thermo Fisher, Waltham, Massachusetts, US) equipped with a high brightness field emission gun (X-FEG), a monochromator, a quadrupole energy-dispersive x-ray system, a high-angle annular dark field detector (Fishione Model 3000), an image Cs-corrector, and a fast CMOS camera (Ceta 2 speed upgrade 4k x 4k). The microscope was operated at an acceleration voltage of 300 kV. Samples were measured on carbon coated copper grids (Plano EM, S160). Images were analyzed using TIA version 4.5 (FEI) and ImageJ version 1.50i (National Institutes of Health, US, Java 1.8.0_77).

Infrared spectra were recorded on a Varian 1300 FT-IR spectrometer from the neat solid compound. The wave numbers ($\tilde{\nu}$) of recorded IR-signals are quoted in cm^{-1}.

Note: Catalytic reactions involving pressurized hydrogen was carried out in Berghof High Pressure Reactors equipped with a pressure transducer and external electrical heating.

Safety advice: Conducting high-pressure experiments present a significant risk. They must be conducted by following appropriate safety procedures in conjunction with the use of suitable equipment.

2. Synthesis of benzoxaborole derivatives using boronic acids

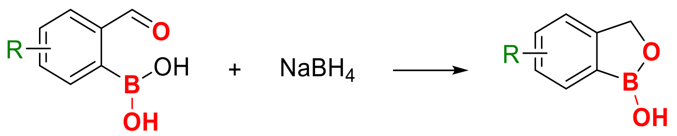

Reaction procedure: At 0 ºC, in an oven dried 100 mL round bottom flask, boronic acid was dissolved in methanol. While stirring, $NaBH_4$ was added portion wise over 5 minutes to the reaction mixture. After the addition of $NaBH_4$, ice bath was removed, and the reaction mixture was further stirred for 2 h. Further, the resulting solution was maintained to pH 7 using 2 M of HCl solution, poured into water and the organic layer was extracted with DCM and washed with brine. $MgSO_4$ was added to the organic fraction and stirred for 10 minutes. The reaction mixture was filtered and concentrated in vacuo. Purification by column chromatography over silica gel (100-200 mesh) using ethyl acetate/pentane mixture as eluent afforded the corresponding benzoxaborole product.

Benzo[*c*][1,2]oxaborol-1(3*H*)-ol (5a)[2]

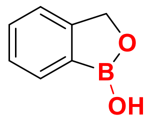

Prepared by following the general experimental procedure with: (2-formylphenyl)boronic acid (3.0 g, 20.0 mmol), $NaBH_4$ (832.0 mg, 22 mmol), mmol), and MeOH (130.0 mL).

Chemical formula: $C_7H_7BO_2$

Molecular weight: 133.94

^{1}H NMR (400 MHz, CD_2Cl_2, 298 K) δ = 7.72-7.74 (m, 1H, ArC*H*), 7.47-7.52 (m, 1H, ArC*H*), 7.37-7.39 (m, 2H, ArC*H*) 5.10 (s, 2H, CH_2).

^{13}C{^{1}H}-NMR (101 MHz, CD_2Cl_2, 298 K) δ = 131.68, 131.37, 130.64, 127.50, 121.59, 71.66.

^{11}B{^{1}H}-NMR (128 MHz, CD_2Cl_2, 298 K): 32.64.

6-methylbenzo[*c*][1,2]oxaborol-1(3*H*)-ol (5e)[3]

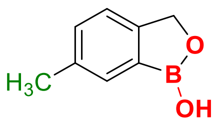

Prepared by following the general experimental procedure with: (2-formyl-5-methylphenyl)boronic acid (327.9 mg, 2.0 mmol), $NaBH_4$ (83.2 mg, 2.2 mmol), and MeOH (30 mL).

Chemical formula: $C_8H_9BO_2$

Molecular weight: 147.97

^{1}H NMR (400 MHz, CD_2Cl_2, 298 K) δ = 7.44 (s, 1H, ArC*H*), 7.23 (dd, 1H, *J* = 7.91, 1.68 Hz, ArC*H*), 7.18 (d, 1H, ArC*H*), 4.97 (s, 2H, CH_2), 2.31 (s, 3H, CH_3).

$^{13}C\{^1H\}$-NMR (101 MHz, CD_2Cl_2, 298 K) δ = 151.76, 137.11, 132.43, 130.81, 121.28, 71.50, 21.37.

$^{11}B\{^1H\}$-NMR (128 MHz, CD_2Cl_2, 298 K): 32.55.

[1,3]dioxolo[4',5':4,5]benzo[1,2-*c*][1,2]oxaborol-1(3*H*)-ol (5i)

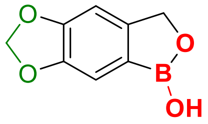

Prepared by following the general experimental procedure with: (6-formylbenzo[*d*][1,3]dioxol-5-yl)boronic acid (388.1 mg, 2.0 mmol), $NaBH_4$ (83.2 mg, 2.2 mmol), and MeOH (30 mL).

Chemical formula: $C_8H_7BO_4$

Molecular weight: 177.95

^1H NMR (400 MHz, CD_3OD, 298 K) δ = 6.98 (s, 1H, ArC*H*), 6.83 (s, 1H, ArC*H*), 5.98 (s, 2H, CH_2), 4.94 (s, 2H, CH_2).

$^{13}C\{^1H\}$-NMR (101 MHz, DMSO-d6, 298 K) δ = 150.31, 149.23, 147.05, 108.62, 102.20, 101.15, 69.62.

$^{11}B\{^1H\}$-NMR (128 MHz, CD_3OD, 298 K) δ = 31.64.

4-fluorobenzo[*c*][1,2]oxaborol-1(3*H*)-ol (5t)[4]

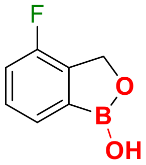

Prepared by following the general experimental procedure with: (3-fluoro-2-formylphenyl)boronic acid (300.0 mg, 1.69 mmol), $NaBH_4$ (70.6 mg, 1.86 mmol), and MeOH (10 mL).

Chemical formula: $C_7H_6BFO_2$

Molecular weight: 151.93

^1H NMR (400 MHz, CD_3OD, 298 K) δ = 7.45-7.48 (m, 1H, ArC*H*), 7.35-7.41 (m, 1H, ArC*H*), 7.13-7.20 (m, 1H, ArC*H*), 4.90 (s, 2H, CH_2).

$^{13}C\{^1H\}$-NMR (101 MHz, CD_3OD, 298 K) δ = 158.56 (d, *J* = 247.59 Hz), 140.41 (d, *J* = 15.05 Hz), 130.79 (d, *J* = 5.67 Hz), 127.17 (d, 3.60 Hz), 117.98 (d, *J* = 19.65 Hz), 68.92.

$^{11}B\{^1H\}$-NMR (128 MHz, CD_3OD, 298 K) δ = 31.40.

$^{19}F\{^1H\}$-NMR (376 MHz, CD_3OD, 298 K) δ = –123.23.

3. Synthesis of substituted 2-(4,4,5,5-tetramethyl-1,3,2-dioxaborolan-2-yl)benzaldehyde from substituted 2-bromobenzaldehyde

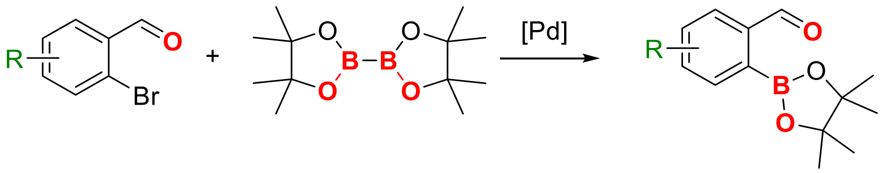

Reaction Procedure: At room temperature, in a schlenk tube, substituted 2-bromobenzaldehyde, Bis-(pinakolato)-diboran, [Pd(dppf)Cl_2·CH_2Cl_2] [1,1′-Bis(diphenylphosphino)ferrocene]dichloropalladium(II), and potassium acetate were added under an argon atmosphere. Degassed and dried dioxane was used as a solvent. The reaction mixture was heated at 90 °C and stirred for 12 h. Dioxane was evaporated, and the resulting solution was poured into water. The organic layer was extracted with DCM and washed with brine. $MgSO_4$ was added to the organic fraction and stirred for 10 minutes. The reaction mixture was filtered and concentrated in vacuo. Purification by column chromatography over silica gel (100-200 mesh) using ethyl acetate/pentane mixture as eluent afforded the corresponding borylated compound.

5-methoxy-2-(4,4,5,5-tetramethyl-1,3,2-dioxaborolan-2-yl)benzaldehyde[5]

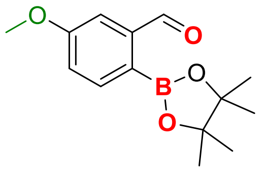

Prepared by following the general experimental procedure with: 2-bromo-5-methoxybenzaldehyde (2.15 g, 10.0 mmol), Bis-(pinakolato)-diboran (2.53 g, 10.0 mmol), [Pd(dppf)Cl_2·CH_2Cl_2] (163.0 mg, 2 mol%), potassium acetate (3.93 g, 40.0 mmol), and dioxane (40 mL).

Chemical formula: $C_{14}H_{19}BO_4$

Molecular weight: 262.11

^{1}H NMR (400 MHz, $CDCl_3$, 298 K) δ = 10.67 (s, 1H, C*H*O), 7.86 (d, *J* = 8.3 Hz, 1H, ArC*H*), 7.50 (d, *J* = 2.6 Hz, 1H, ArC*H*), 7.12 (dd, *J* = 8.3, 2.7 Hz, 1H, ArC*H*), 3.87 (s, 3H, C*H*$_3$), 1.36 (s, 12H, C*H*$_3$).

^{13}C{^{1}H}-NMR (101 MHz, $CDCl_3$, 298 K) δ = 195.02, 162.07, 143.72, 138.07, 120.14, 110.37, 84.29, 55.55, 25.01.

^{11}B{^{1}H}-NMR (128 MHz, $CDCl_3$,, 298 K) δ = 30.87.

5-hydroxy-2-(4,4,5,5-tetramethyl-1,3,2-dioxaborolan-2-yl)benzaldehyde[6]

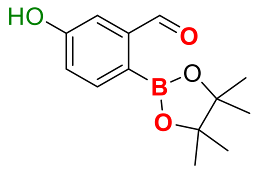

Prepared by following the general experimental procedure with: 2-bromo-5-hydroxybenzaldehyde (603.1 mg, 3.0 mmol), Bis-(pinakolato)-diboran (761.8 mg, 3.0 mmol), [Pd(dppf)Cl_2·CH_2Cl_2] (48.9 mg, 2 mol%), potassium acetate (1177.7 mg, 12 mmol), and dioxane (15 mL).

Chemical formula: $C_{13}H_{17}BO_4$

Molecular weight: 248.09

^{1}H NMR (400 MHz, $CDCl_3$, 298 K) δ = 10.58 (s, 1H, C*H*O), 7.78 (d, 1H, *J* = 8.05 Hz, ArC*H*), 7.42-7.43 (m, 1H, ArC*H*), 7.03 (dd, 1H, *J* = 8.15, 2.62 Hz, ArC*H*), 1.69 (br s, 1H, O*H*), 1.30 (s, 12H, CH_3).

^{13}C{^{1}H}-NMR (101 MHz, $CDCl_3$, 298 K) δ = 195.71, 159.21, 144.19, 139.01, 120.98, 113.66, 84.85, 25.40.

^{11}B{^{1}H}-NMR (128 MHz, $CDCl_3$, 298 K) δ = 31.03.

5-(trifluoromethyl)-2-(4,4,5,5-tetramethyl-1,3,2-dioxaborolan-2-yl)benzaldehyde[5]

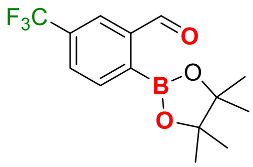

Prepared by following the general experimental procedure with: 2-bromo-5-(trifluoromethyl)benzaldehyde (1.51 mL, 10.0 mmol), Bis-(pinakolato)-diboran (2.53 g, 10.0 mmol), [Pd(dppf)Cl_2·CH_2Cl_2] (163.0 mg, 2 mol%), potassium acetate (3.93 g, 40.0 mmol), and dioxane (40 mL).

Chemical Formula: $C_{14}H_{16}BF_3O_3$

Molecular Weight: 262.11

^{1}H NMR (400 MHz, $CDCl_3$, 298 K) δ = 10.59 (s, 1H, C*H*O), 8.21 (s, 1H, ArC*H*), 8.01 (d, *J* = 7.7 Hz, 1H, ArC*H*), 7.82 (dd, *J* = 7.8, 1.1 Hz, 1H, ArC*H*), 1.40 (s, 12H, CH_3).

^{13}C{^{1}H}-NMR (101 MHz, $CDCl_3$, 298 K) δ = 193.37, 141.84, 136.43, 132.83 (q, *J* = 32.7 Hz), 129.15 (q, *J* = 3.6 Hz), 124.59 (q, *J* = 3.7 Hz), 123.70 (q, *J* = 271.7 Hz), 85.08, 25.01.

^{11}B{^{1}H}-NMR (128 MHz, $CDCl_3$,, 298 K) δ = 30.83.

^{19}F{^{1}H}-NMR (376 MHz, $CDCl_3$, 298 K) δ = –63.26.

4-methoxy-2-(4,4,5,5-tetramethyl-1,3,2-dioxaborolan-2-yl)benzaldehyde[5]

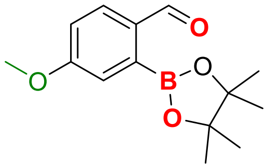

Prepared by following the general experimental procedure with: 2-bromo-4-methoxybenzaldehyde (2.15 g, 10.0 mmol), Bis-(pinakolato)-diboran (2.53 g, 10.0 mmol), [Pd(dppf)Cl_2·CH_2Cl_2] (163.0 mg, 2 mol%), potassium acetate (3.93 g, 40.0 mmol), and dioxane (40 mL).

Chemical formula: $C_{14}H_{19}BO_4$

Molecular weight: 262.11

^{1}H NMR (400 MHz, $CDCl_3$, 298 K) δ = 10.36 (s, 1H, C*H*O), 7.93 (d, *J* = 8.6 Hz, 1H, ArC*H*), 7.28 (d, *J* = 2.7 Hz, 1H, ArC*H*), 7.02 (dd, *J* = 8.6, 2.6 Hz, 1H, ArC*H*), 3.89 (s, 3H, C*H*$_3$), 1.39 (s, 12H, C*H*$_3$).

^{13}C{^{1}H}-NMR (101 MHz, $CDCl_3$, 298 K) δ = 193.13, 163.47, 134.75, 130.80, 120.00, 116.32, 84.58, 55.67, 25.00.

4,5-dimethoxy-2-(4,4,5,5-tetramethyl-1,3,2-dioxaborolan-2-yl)benzaldehyde[5]

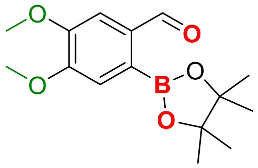

Prepared by following the general experimental procedure with: 2-bromo-4,5-dimethoxybenzaldehyde (731.9 mg, 3.0 mol%), Bis-(pinakolato)-diboran (761.2 mg, 3 mmol), [Pd(dppf)Cl_2·CH_2Cl_2] (48.96 mg, 2 mol%), potassium acetate (1177.1 mg, 12 mmol), and dioxane (15 mL).

Chemical formula: $C_{15}H_{21}BO_5$

Molecular weight: 292.14

^{1}H NMR (400 MHz, $CDCl_3$, 298 K) δ = 10.59 (s, 1H, C*H*O), 7.55 (s, 1H, ArC*H*), 7.35 (s, 1H, ArC*H*), 4.00 (s, 3H, OC*H*$_3$), 3.96 (s, 3H, OC*H*$_3$), 1.37 (s, 12H, C*H*$_3$).

^{13}C{^{1}H}-NMR (101 MHz, $CDCl_3$, 298 K) δ = 193.90, 152.91, 151.32, 136.42, 117.32, 108.71, 84.45, 56.30, 56.12, 25.02.

^{11}B{^{1}H}-NMR (128 MHz, $CDCl_3$, 298 K) δ = 31.18.

4-fluoro-2-(4,4,5,5-tetramethyl-1,3,2-dioxaborolan-2-yl)benzaldehyde[5]

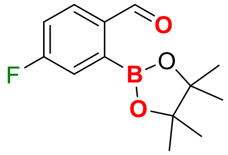

Prepared by following the general experimental procedure with: 2-bromo-4-fluorobenzaldehyde (1.51 mL, 10.0 mmol), Bis-(pinakolato)-diboran (2.53 g, 10.0 mmol), [Pd(dppf)Cl_2·CH_2Cl_2] (163.0 mg, 2 mol%), potassium acetate (3.93 g, 40.0 mmol), and dioxane (40 mL).

Chemical Formula: $C_{13}H_{16}BFO_3$

Molecular Weight: 262.11

^{1}H NMR (400 MHz, $CDCl_3$, 298 K) δ =10.47 (s, 1H, C*H*O), 7.99 (dd, *J* = 8.6, 5.4 Hz, 1H, ArC*H*), 7.52 (dd, *J* = 8.9, 2.7 Hz, 1H, ArC*H*), 7.24 – 7.18 (m, 1H, ArC*H*), 1.39 (s, 12H, C*H*3).

^{19}F{^{1}H}-NMR (376 MHz, $CDCl_3$, 298 K) δ = –104.75.

4,5-difluoro-2-(4,4,5,5-tetramethyl-1,3,2-dioxaborolan-2-yl)benzaldehyde

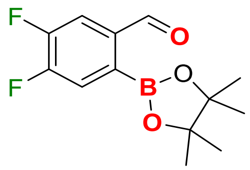

Prepared by following the general experimental procedure with: 2-bromo-4,5-difluorobenzaldehyde (442.0 mg, 2.0 mmol), Bis-(pinakolato)-diboran (507.8 mg, 2.0 mmol), [Pd(dppf)Cl_2·CH_2Cl_2] (32.6 mg, 2 mol%), potassium acetate (785.1 mg, 8 mmol), and dioxane (10 mL).

Chemical formula: $C_{13}H_{15}BF_2O_3$

Molecular weight: 268.07

^{1}H NMR (400 MHz, $CDCl_3$, 298 K) δ = 10.56 (d, 1H, *J* = 3.09 Hz, ArC*H*), 7.80 (dd, 1H, *J* = 10.74, 7.63 Hz, ArC*H*), 7.71 (dd, 1H, *J* = 10.38, 7.99 Hz, ArC*H*), 1.38 (s, 12H, C*H*3).

^{13}C{^{1}H}-NMR (101 MHz, $CDCl_3$, 298 K) δ = 192.38, 154.24 (dd, *J* = 102.90, 12.), 151.68 (dd, *J* = 99.58, 12.96 Hz), 139.37 (m), 125.03 (d, *J* = 16.77 Hz), 116.51 (m), 85.04, 24.99.

^{11}B{^{1}H}-NMR (128 MHz, $CDCl_3$, 298 K) δ = 30.33

4. Synthesis of benzoxaborole derivatives from substituted 2-(4,4,5,5-tetramethyl-1,3,2-dioxaborolan-2-yl)benzaldehyde

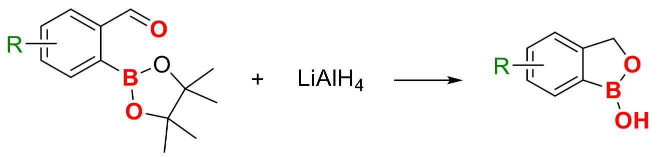

Reaction Procedure: In a round bottom flask, at 0 °C, substituted-borylated benzaldehyde was dissolved in THF. $LiAlH_4$ was added portion wise over 5 minutes to the reaction mixture. The reaction mixture was stirred for 2 h at 0 °C. The resulting solution was maintained to pH 7 using 2 M of HCl solution and poured into water. The organic layer was extracted with DCM and washed with brine. $MgSO_4$ was added to the organic fraction and stirred for 10 minutes. The reaction mixture was filtered and concentrated in vacuo. Purification by column chromatography over silica gel (100-200 mesh) using ethyl acetate/pentane mixture as eluent afforded the corresponding benzoxaborole compound.

5-methoxybenzo[*c*][1,2]oxaborol-1(3*H*)-ol (5b)[7]

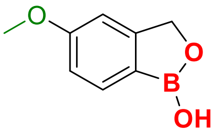

Prepared by following the general experimental procedure with: 5-methoxy-2-(4,4,5,5-tetramethyl-1,3,2-dioxaborolan-2-yl)benzaldehyde (2.05 g, 7.8 mmol), $LiAlH_4$ (296.0 mg, 7.8 mmol), and THF (8 mL).

Chemical Formula: $C_8H_9BO_3$

Molecular Weight: 163.97

^{1}H NMR (400 MHz, $(CD_3)_2SO$, 298 K) δ = 8.99 (s, 1H, O*H*), 7.62 (d, *J* = 8.1 Hz, 1H, ArC*H*), 6.96 (d, *J* = 2.2 Hz, 1H, ArC*H*), 6.92 – 6.87 (m, 1H, ArC*H*), 4.92 (s, 2H, CH_2), 3.78 (s, 3H, OCH_3).

^{13}C{^{1}H}-NMR (101 MHz, $(CD_3)_2SO$, 298 K) δ = 161.76, 156.41, 131.69, 114.31, 105.78, 69.68, 55.15.

^{11}B{^{1}H}-NMR (128 MHz, $(CD_3)_2SO$, 298 K) δ = 32.25.

Benzo[*c*][1,2]oxaborole-1,5(3*H*)-diol (5c)[8]

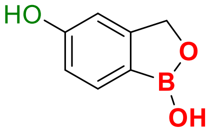

Prepared by following the general experimental procedure with: 5-hydroxy-2-(4,4,5,5-tetramethyl-1,3,2-dioxaborolan-2-yl)benzaldehyde (248.1 mg, 1 mmol), $LiAlH_4$ (37.9 mg, 1 mmol), and THF (1 mL).

Chemical formula: $C_7H_7BO_3$

Molecular weight: 149.94

^{1}H NMR (400 MHz, CD_2Cl_2, 298 K) δ = 7.52 (d, 1H, *J* = 7.93 Hz, ArC*H*), 6.75-6.80 (m, 2H, ArC*H*), 4.97 (s, 2H, CH_2), 3.24 (br s, 2H, O*H*).

^{13}C{^{1}H}-NMR (101 MHz, $CDCl_3$, 298 K) δ = 159.93, 156.99, 132.06, 115.53, 107.93, 71.27.

^{11}B{^{1}H}-NMR (128 MHz, CD_2Cl_2, 298 K) δ = 31.89.

5-(trifluoromethyl)benzo[*c*][1,2]oxaborol-1(3*H*)-ol (5d)

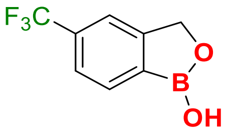

Prepared by following the general experimental procedure with: 2-(4,4,5,5-tetramethyl-1,3,2-dioxaborolan-2-yl)-5-(trifluoromethyl)benzaldehyde (2.19 g, 7.3 mmol), $LiAlH_4$ (277 mg, 7.3 mmol), and THF (8 mL).

Chemical Formula: $C_8H_6BF_3O_2$

Molecular Weight: 201.94

^{1}H NMR (400 MHz, $(CD_3)_2SO$, 298 K) δ = 9.49 (s, 1H, O*H*), 7.93 (d, *J* = 7.8 Hz, 1H, ArC*H*), 7.77 (s, 1H, ArC*H*), 7.66 (d, *J* = 6.3 Hz, 1H, ArC*H*), 5.07 (s, 2H, CH_2).

^{13}C{^{1}H}-NMR (101 MHz, $(CD_3)_2SO$, 298 K) δ = 154.64, 131.45, 130.85 (q, J = 31.2 Hz), 124.42 (q, J = 272.6 Hz), 123.59, 118.51 – 118.24 (m), 69.99.

^{11}B{^{1}H}-NMR (128 MHz, $(CD_3)_2SO$, 298 K) δ = 31.79.

^{19}F{^{1}H}-NMR (376 MHz, $(CD_3)_2SO$, 298 K) δ = –61.06.

6-methoxybenzo[*c*][1,2]oxaborol-1(3*H*)-ol (5f)[9]

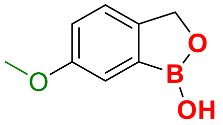

Prepared by following the general experimental procedure with: 4-methoxy-2-(4,4,5,5-tetramethyl-1,3,2-dioxaborolan-2-yl)benzaldehyde (2.10 g, 8.0 mmol), $LiAlH_4$ (304 mg, 8.0 mmol), and THF (15 mL).

Chemical Formula: $C_8H_9BO_3$

Molecular Weight: 163.97

^{1}H NMR (400 MHz, $CDCl_3$, 298 K) δ = 9.14 (s, 1H, O*H*), 7.30 (d, J = 8.3 Hz, 1H, ArC*H*), 7.25 (d, J = 2.6 Hz, 1H, ArC*H*), 7.04 (dd, J = 8.3, 2.6 Hz, 1H, ArC*H*), 4.92 (s, 2H, CH_2), 3.76 (s, 3H, CH_3).

^{13}C{^{1}H}-NMR (101 MHz, CD_2Cl_2, 298 K) δ = 158.16, 145.94, 122.33, 118.04, 113.67, 69.62, 55.12.

^{11}B{^{1}H}-NMR (128 MHz, CD_3OD, 298 K) δ = 31.88.

5,6-dimethoxybenzo[*c*][1,2]oxaborol-1(3*H*)-ol (5j)[10]

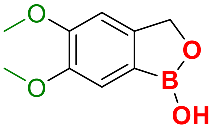

Prepared by following the general experimental procedure with: 4,5-dimethoxy-2-(4,4,5,5-tetramethyl-1,3,2-dioxaborolan-2-yl)benzaldehyde (292.0 mg, 1.0 mmol), $NaBH_4$ (37.83 mg, 1.0 mmol), and EtOH (7 mL).

Chemical formula: $C_9H_{11}BO_4$

Molecular weight: 193.99

^{1}H NMR (400 MHz, CD_2Cl_2, 298 K) δ = 7.07 (s, 1H, ArC*H*), 6.78 (s, 1H, ArC*H*), 4.91 (s, 2H, CH_2), 4.82 (br s, 1H, O*H*), 3.78 (s, 3H, OCH_3), 3.77 (s, 3H, OCH_3).

^{13}C{^{1}H}-NMR (101 MHz, CD_2Cl_2, 298 K) δ = 152.96, 149.44, 148.45, 111.67, 104.22, 71.26, 56.22, 56.10.

^{11}B{^{1}H}-NMR (128 MHz, CD_3OD, 298 K) δ = 32.63.

6-fluorobenzo[*c*][1,2]oxaborol-1(3*H*)-ol (5u)

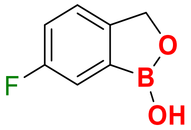

Prepared by following the general experimental procedure with: 6-fluoro-2-(4,4,5,5-tetramethyl-1,3,2-dioxaborolan-2-yl)benzaldehyde (2.0 g, 8.1 mmol), $LiAlH_4$ (308.0 mg, 8.1 mmol), and THF (8 mL).

Chemical Formula: $C_8H_6BF_3O_2$

Molecular Weight: 201.94

^{1}H NMR (400 MHz, $(CD_3)_2SO$, 298 K) δ =9.43 (s, 1H, O*H*), 7.42 (dt, *J* = 8.3, 4.1 Hz, 2H, ArC*H*), 7.31 – 7.21 (m, 1H, ArC*H*), 4.94 (s, 2H, CH_2).

^{13}C{^{1}H}-NMR (101 MHz, $(CD_3)_2SO$, 298 K) δ = 161.93 (d, *J* = 242.3 Hz), 149.75 (d, *J* = 1.8 Hz), 123.64 (d, *J* = 8.0 Hz), 118.26 (d, *J* = 23.4 Hz), 116.15 (d, *J* = 20.1 Hz), 69.90.

^{11}B{^{1}H}-NMR (128 MHz, $(CD_3)_2SO$, 298 K) δ = 31.91.

^{19}F{^{1}H}-NMR (376 MHz, $(CD_3)_2SO$, 298 K) δ = –116.71.

5,6-difluorobenzo[*c*][1,2]oxaborol-1(3*H*)-ol (5v)

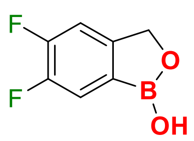

Prepared by following the general experimental procedure with: 4,5-difluoro-2-(4,4,5,5-tetramethyl-1,3,2-dioxaborolan-2-yl)benzaldehyde (397.3 mg, 1.48 mmol), $LiAlH_4$ (56.2 mg, 1.48 mmol), and THF (2 mL).

Chemical formula: $C_7H_5BF_2O_2$

Molecular weight: 169.92

^{1}H NMR (400 MHz, CD_3OD, 298 K) δ = 7.45 (t, 1H, *J* = 7.45 Hz), 7.30 (dd, 1H, ArC*H*), 5.03 (s, 2H, CH_2).

^{11}B{^{1}H}-NMR (128 MHz, CD_3OD, 298 K) δ = 31.30.

^{19}F{^{1}H}-NMR (376 MHz, CD_3OD, 298 K) δ = –132.08 (d, *J* = 19.42 Hz), –138.77 (d, *J* = 18.26 Hz).

5. Synthesis of benzoxaborole derivatives from the reaction of substituted 2-(4,4,5,5-tetramethyl-1,3,2-dioxaborolan-2-yl)benzaldehyde and Grignard reagents

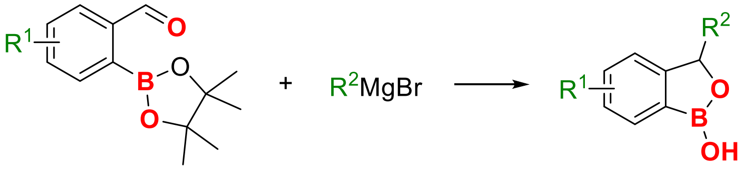

In a schlenk tube, at 0 °C, substituted-borylated benzaldehyde was dissolved in THF under argon atmosphere. Grignard reagent was added portion wise over 5 minutes to the reaction mixture. After the addition of Grignard reagent, the ice bath was removed, and the reaction mixture was further stirred for 2 h. Water was added to the reaction mixture and the resulting solution was maintained to pH 7 using 2 M of HCl solution. The organic layer was extracted with DCM (2 x 50 mL) and washed with brine (20 mL).

$MgSO_4$ was added to the organic fraction and stirred for 10 minutes. The reaction mixture was filtered and concentrated in vacuo. Purification by column chromatography over silica gel (100-200 mesh) using ethyl acetate/pentane mixture as eluent afforded the corresponding benzoxaborole compound.

3-methylbenzo[*c*][1,2]oxaborol-1(3*H*)-ol (5k)[11]

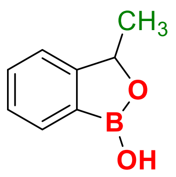

Prepared by following the general experimental procedure with: 2-(4,4,5,5-tetramethyl-1,3,2-dioxaborolan-2-yl)benzaldehyde (696.0 mg, 3.0 mmol), methylmagnesium bromide (1.2 mL, 3 M in Et_2O, 3.6 mmol) and THF (15 mL).

Chemical Formula: $C_8H_9BO_2$

Molecular Weight: 147.97

^{1}H NMR (400 MHz, $(CD_3)_2SO$, 298 K) δ = 9.09 (s, 1H, O*H*), 7.70 (dt, *J* = 7.3, 1.1 Hz, 1H, ArC*H*), 7.46 (td, *J* = 7.4, 1.2 Hz, 1H, ArC*H*), 7.43 – 7.29 (m, 2H, ArC*H*), 5.20 (q, *J* = 6.6 Hz, 1H, C*H*), 1.39 (d, *J* = 6.6 Hz, 3H, CH_3).

^{13}C{^{1}H}-NMR (101 MHz, $(CD_3)_2SO$, 298 K) δ = 158.40, 130.56, 130.40, 126.94, 121.15, 76.49, 22.55.

3-ethylbenzo[*c*][1,2]oxaborol-1(3*H*)-ol (5l)

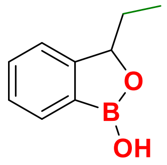

Prepared by following the general experimental procedure with: 2-(4,4,5,5-tetramethyl-1,3,2-dioxaborolan-2-yl)benzaldehyde (696.3 mg, 3.0 mmol), ethylmagnesium bromide (1.2 mL, 3 M in Et_2O, 3.6 mmol) and THF (15 mL).

Chemical Formula: $C_9H_{11}BO_2$

Molecular Weight: 161.99

^{1}H NMR (400 MHz, $(CD_3)_2SO$, 298 K) δ = 9.10 (s, 1H, O*H*), 7.70 (d, *J* = 7.3 Hz, 1H, ArC*H*), 7.48 – 7.43 (m, 1H, ArC*H*), 7.40 – 7.29 (m, 2H, ArC*H*), 5.08 (dd, *J* = 7.4, 3.8 Hz, 1H, C*H*), 1.96 (dqd, *J* = 14.7, 7.4, 3.8 Hz, 1H, CH_2), 1.50 (dp, *J* = 14.6, 7.3 Hz, 1H, CH_2), 0.85 (t, *J* = 7.3 Hz, 3H, CH_3).

^{13}C{^{1}H}-NMR (101 MHz, $(CD_3)_2SO$, 298 K) δ = 156.73, 130.54, 130.36, 126.97, 121.26, 81.05, 28.83, 9.12.

^{11}B{^{1}H}-NMR (128 MHz, $(CD_3)_2SO$, 298 K) δ = 31.62.

3-propylbenzo[*c*][1,2]oxaborol-1(3*H*)-ol (5m)

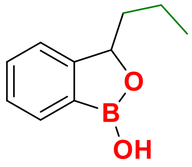

Prepared by following the general experimental procedure with: 2-(4,4,5,5-tetramethyl-1,3,2-dioxaborolan-2-yl)benzaldehyde (696.3 mg, 3.0 mmol), propyl-magnesium chloride (1.8 mL, 2 M in Et_2O, 3.6 mmol) and THF (15 mL).

Chemical Formula: $C_{10}H_{13}BO_2$

Molecular Weight: 176.02

^{1}H NMR (400 MHz, $(CD_3)_2SO$, 298 K) δ = 9.09 (s, 1H, O*H*), 7.69 (dt, *J* = 7.2, 1.1 Hz, 1H, ArC*H*), 7.45 (td, *J* = 7.4, 1.2 Hz, 1H, ArC*H*), 7.39 – 7.35 (m, 1H, ArC*H*), 7.32 (tt, *J* = 7.3, 0.9 Hz, 1H, ArC*H*), 5.16 – 5.08 (m, 1H, CH_2), 1.97 – 1.80 (m, 1H, C*H*), 1.50 – 1.27 (m, 3H, CH_2), 0.91 (t, *J* = 7.3 Hz, 3H, CH_3).

^{13}C{^{1}H}-NMR (101 MHz, $(CD_3)_2SO$, 298 K) δ = 157.15, 130.53, 130.38, 126.93, 121.23, 79.92, 38.35, 18.10, 13.90.

^{11}B{^{1}H}-NMR (128 MHz, $(CD_3)_2SO$, 298 K) δ =31.66.

3-phenylbenzo[*c*][1,2]oxaborol-1(3*H*)-ol (5n)

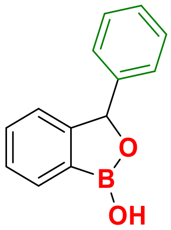

Prepared by following the general experimental procedure with: 2-(4,4,5,5-tetramethyl-1,3,2-dioxaborolan-2-yl)benzaldehyde (696.3 mg, 3.0 mmol), phenyl-magnesium bromide (1.2 mL, 3 M in Et_2O, 3.6 mmol) and THF (15 mL).

Chemical Formula: $C_{13}H_{11}BO_2$

Molecular Weight: 210.04

^{1}H NMR (400 MHz, $(CD_3)_2SO$, 298 K) δ =9.41 (s, 1H, O*H*), 7.77 (d, *J* = 7.1 Hz, 1H, ArC*H*), 7.41 (td, *J* = 7.4, 1.4 Hz, 1H, ArC*H*), 7.38 – 7.32 (m, 3H, ArC*H*), 7.31 – 7.25 (m, 3H, ArC*H*), 7.17 (d, *J* = 7.5 Hz, 1H, ArC*H*), 6.18 (s, 1H, C*H*).

^{13}C{^{1}H}-NMR (101 MHz, $(CD_3)_2SO$, 298 K) δ = 156.94, 141.28, 130.91, 130.48, 128.49, 127.78, 127.24, 126.15, 122.19, 81.66.

^{11}B{^{1}H}-NMR (128 MHz, $(CD_3)_2SO$, 298 K) δ =32.21.

3-isopropylbenzo[*c*][1,2]oxaborol-1(3*H*)-ol (5o)

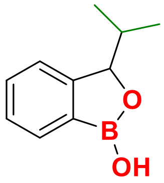

Prepared by following the general experimental procedure with: 2-(4,4,5,5-tetramethyl-1,3,2-dioxaborolan-2-yl)benzaldehyde (696.3 mg, 3.0 mmol),

isopropylmagnesium chloride (1.8 mL, 2 M in Et_2O, 3.6 mmol) and THF (15 mL).

Chemical Formula: $C_{10}H_{13}BO_2$

Molecular Weight: 176.02

^{1}H NMR (400 MHz, $(CD_3)_2SO$, 298 K) δ = 9.10 (s, 1H, O*H*), 7.69 (d, *J* = 7.3 Hz, 1H, ArC*H*), 7.46 (td, *J* = 7.4, 1.2 Hz, 1H, ArC*H*), 7.38 – 7.30 (m, 2H, ArC*H*), 5.05 (d, *J* = 3.1 Hz, 1H, C*H*), 2.14 (pd, *J* = 6.8, 3.0 Hz, 1H, C*H*), 1.05 (d, *J* = 6.9 Hz, 3H, CH_3), 0.51 (d, *J* = 6.9 Hz, 3H, CH_3).

^{13}C{^{1}H}-NMR (101 MHz, $(CD_3)_2SO$, 298 K) δ = 155.79, 130.51, 130.28, 126.95, 121.41, 84.18, 32.06, 19.50, 14.71.

^{11}B{^{1}H}-NMR (128 MHz, $(CD_3)_2SO$, 298 K) δ = 31.65.

3-((trimethylsilyl)methyl)benzo[*c*][1,2]oxaborol-1(3*H*)-ol (5p)

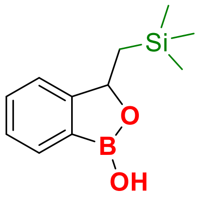

Prepared by following the general experimental procedure with: 2-(4,4,5,5-tetramethyl-1,3,2-dioxaborolan-2-yl)benzaldehyde (696.3 mg, 3.0 mmol), ((trimethylsilyl)methyl)magnesium chloride (3.6 mL, 1 M in Et_2O, 3.6 mmol) and THF (15 mL).

Chemical Formula: $C_{11}H_{17}BO_2Si$

Molecular Weight: 220.15

^{1}H NMR (400 MHz, $(CD_3)_2SO$, 298 K) δ = 9.03 (s, 1H, O*H*), 7.67 (dt, J = 7.3, 1.1 Hz, 1H, ArC*H*), 7.44 (td, J = 7.4, 1.2 Hz, 1H, ArC*H*), 7.36 (dd, J = 7.6, 1.0 Hz, 1H, ArC*H*), 7.30 (tt, J = 7.2, 0.9 Hz, 1H, ArC*H*), 5.27 (dd, J = 9.4, 4.1 Hz, 1H, C*H*), 1.37 (dd, J = 14.7, 4.1 Hz, 1H, CH_2), 0.80 (dd, J = 14.7, 9.5 Hz, 1H, CH_2), –0.01 (s, 9H, CH_3).

^{13}C{^{1}H}-NMR (101 MHz, , $(CD_3)_2SO$, 298 K) δ = 159.71, 130.51, 130.24, 126.72, 121.25, 78.62, 24.53, –0.41.

^{11}B{^{1}H}-NMR (128 MHz, $(CD_3)_2SO$, 298 K) δ = 31.90.

3-ethyl-5-methoxy-1,3-dihydrobenzo[*c*][1,2]oxaborole (5q)

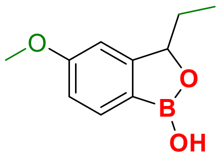

Prepared by following the general experimental procedure with: 5-methoxy-2-(4,4,5,5-tetramethyl-1,3,2-dioxaborolan-2-yl)benzaldehyde (300.0 mg, 1.14 mmol), ethylmagnesium bromide (183.0 mg, 1.37 mmol), and THF (6 mL).

Chemical formula: $C_{10}H_{13}BO_3$

Molecular weight: 192.02

^{1}H NMR (400 MHz, $CDCl_3$, 298 K) δ = 7.62 (d, 1H, *J* = 8.08 Hz, ArC*H*), 6.91 (dd, 1H, *J* = 8.10, 2.24 Hz, ArC*H*), 6.78 (d, 1H, *J* = 2.20 Hz, ArC*H*), 5.22 (br s, 1H, O*H*), 5.15 (dd, 1H, *J* = 7.26, 3.94 Hz, C*H*), 3.85 (s, 3H, OC*H*$_3$), 1.91-2.06 (m, 1H, C*H*), 1.63-1.70 (m, 1H, C*H*), 0.96 (t, 3H, *J* = 7.35 Hz, C*H*$_3$).

^{13}C{^{1}H}-NMR (101 MHz, $CDCl_3$, 298 K) δ = 162.58, 159.52, 131.77, 114.39, 106.13, 82.58, 55.47, 29.27, 9.13.

^{11}B{^{1}H}-NMR (128 MHz, $CDCl_3$, 298 K) δ = 31.95.

5-methoxy-3-propyl-1,3-dihydrobenzo[*c*][1,2]oxaborole (5r)

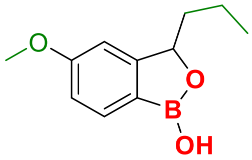

Prepared by following the general experimental procedure with: 5-methoxy-2-(4,4,5,5-tetramethyl-1,3,2-dioxaborolan-2-yl)benzaldehyde (300.0 mg, 1.14 mmol), propylmagnesium bromide (140.9 mg, 1.37 mmol), and THF (6 mL).

Chemical formula: $C_{11}H_{15}BO_3$

Molecular weight: 206.04800

^{1}H NMR (400 MHz, $CDCl_3$, 298 K) δ = 7.57 (d, 1H, *J* = 8.23 Hz, ArC*H*), 6.81 (dd, 1H, *J* = 8.12, 2.26 Hz, ArC*H*), 6.69 (d, 1H, *J* = 2.21 Hz, ArC*H*), 5.08-5.15 (m ,1H, C*H*), 3.75 (s, 3H, OC*H*$_3$), 1.80-1.86 (m, 1H, C*H*), 1.33-1.58 (m, 3H, C*H* & C*H*$_2$), 0.87 (t, 3H, *J* = 7.31 Hz, C*H*$_3$).

^{13}C{^{1}H}-NMR (101 MHz, $CDCl_3$, 298 K) δ = 159.88, 146.84, 129.55, 118.37, 113.03, 111.51, 74.48, 55.34, 41.33, 19.16, 14.09.

^{11}B{^{1}H}-NMR (128 MHz, $CDCl_3$, 298 K) δ = 31.90.

3-ethyl-5,6-dimethoxybenzo[*c*][1,2]oxaborol-1(3*H*)-ol (5s)

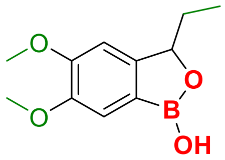

Prepared by following the general experimental procedure with: 4,5-dimethoxy-2-(4,4,5,5-tetramethyl-1,3,2-dioxaborolan-2-yl)benzaldehyde (292.2 mg, 1 mmol), ethylmagnesium bromide (159.9 mg, 1.2 mmol), and THF (5 mL).

Chemical formula: $C_{11}H_{15}BO_4$

Molecular weight: 222.05

^{1}H NMR (400 MHz, CD_2Cl_2, 298 K) δ = 7.04 (s, 1H, ArC*H*), 6.71 (s, 1H, ArC*H*), 5.02 (dd, 1H, *J* = 7.32, 3.81 Hz, C*H*), 3.79 (s, 3H, OC*H*$_3$), 3.76 (s, 3H, OC*H*$_3$), 1.47-1.61 (m, 2H, C*H*$_2$), 0.84 (t, 3H, *J* = 7.34 Hz, C*H*$_3$).

$^{13}C\{^1H\}$**-NMR** (101 MHz, CD_2Cl_2, 298 K) δ = 152.46, 151.14, 149.08, 111.19, 103.91, 82.34, 55.82, 55.72, 29.23, 8.75.

$^{11}B\{^1H\}$**-NMR** (128 MHz, CD_2Cl_2, 298 K) δ = 32.03.

6. Synthesis of 6-nitrobenzo[*c*][1,2]oxaborol-1(3*H*)-ol

The synthesis was performed according to a literature procedure: *ACS Chem. Biol.* **2020**, *15*, 1930–1941.[12]

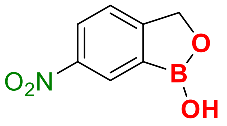

Chemical formula: $C_7H_6BNO_4$

Molecular weight: 178.94

^{1}**H NMR** (400 MHz, $(CD_3)_2SO$, 298 K) δ = 9.59 (br s, 1H, O*H*), 8.59 (d, 1H, *J* = 2.24 Hz, ArC*H*), 8.34 (dd, 1H, *J* = 8.42, 2.29 Hz, ArC*H*), 7.71 (d, 1H, *J* = 8.40 Hz, ArC*H*), 5.13 (s, 2H, CH_2).

$^{13}C\{^1H\}$**-NMR** (101 MHz, $(CD_3)_2SO$, 298 K) δ = 160.62, 147.20, 125.63, 125.49, 123.08, 70.08.

$^{11}B\{^1H\}$**-NMR** (128 MHz, $(CD_3)_2SO$, 298 K) δ = 32.44.

7. Synthesis of 6-aminobenzo[*c*][1,2]oxaborol-1(3*H*)-ol

The synthesis was performed according to a literature procedure: *ACS Chem. Biol.* **2020**, *15*, 1930–1941.[12].

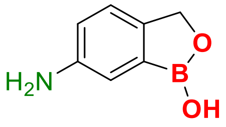

Chemical formula: $C_7H_8BNO_2$

Molecular weight: 148.96

^{1}**H NMR** (400 MHz, $(CD_3)_2SO$, 298 K) δ = 8.91 (s, 1H, O*H*), 7.03 (d, 1H, *J* = 8.06 Hz, ArC*H*), 6.88 (d, 1H, *J* = 2.08 Hz, ArC*H*), 6.70 (dd, 1H, *J* = 8.09, 2.18 Hz, ArC*H*), 4.98 (br s, 2H, NH_2), 4.81 (s, 2H, CH_2).

$^{13}C\{^1H\}$**-NMR** (101 MHz, $(CD_3)_2SO$, 298 K) δ = 147.57, 141.38, 121.41, 117.57, 114.57, 69.61.

8. Synthesis of *tert*-butyl (1-hydroxy-1,3-dihydrobenzo[*c*][1,2]oxaborol-6-yl)carbamate (5g)

Reaction Procedure: Na_2CO_3 (212.0 mg, 2.0 mmol) and Boc_2O (327.4 mg, 1.5 mmol) were added to a solution of 6-aminobenzo[c][1,2]oxaborol-1(3*H*)-ol (149.0 mg, 1.0 mmol) in H_2O (2 mL) and THF (1 mL) at 0 °C. Afterwards, the ice bath was removed, and the reaction mixture was stirred at room temperature for 12 h, it was neutralized with HCl (10%) until pH 2 had been reached. The mixture was then extracted with

EtOAc, washed with brine, and dried over Na_2SO_4. Concentration gave the crude *N*-Boc-5-aminobenzo[c][1,2]oxaborol-1(3*H*)-ol (**5g**).

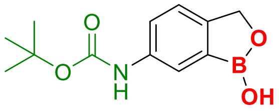

Chemical formula: $C_{12}H_{16}BNO_4$

Molecular weight: 249.07

^{1}H NMR (400 MHz, $CDCl_3$, 298 K) δ = 7.68 (d, 1H, *J* = 2.17 Hz, ArC*H*), 7.52 (dd, 1H, *J* = 8.35, 2.14 Hz, ArC*H*), 7.26-7.28 (m, 1H, ArC*H*), 6.54 (br s, 1H, N*H*), 5.06 (s, 2H, CH_2), 4.91 (br s, 1H, O*H*), 1.53 (s, 9H, CH_3).

^{13}C{^{1}H}-NMR (101 MHz, $CDCl_3$, 298 K) δ = 167.24, 148.60, 137.55, 137.47, 121.66, 121.56, 71.11, 71.04, 28.36.

^{11}B{^{1}H}-NMR (128 MHz, $CDCl_3$, 298 K) δ = 32.72.

9. Synthesis of *N*-(1-hydroxy-1,3-dihydrobenzo[*c*][1,2]oxaborol-6-yl)acetamide (5x)

The synthesis was performed according to a literature procedure: *ACS Med. Chem. Lett.* **2010**, *1*, 165–169.[13]

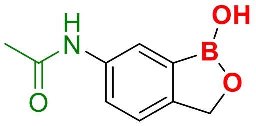

Chemical formula: $C_9H_{10}BNO_3$

Molecular weight: 190.99

^{1}H NMR (400 MHz, CD_3OD, 298 K) δ = 7.69 (m, 1H, ArC*H*), 7.55 (dd, 1H, *J* = 8.23, 2.05 Hz), 7.22 (d, 1H, *J* = 8.26 Hz), 4.93 (s, 2H, CH_2), 2.03 (s, 3H, CH_3).

^{13}C{^{1}H}-NMR (101 MHz, CD_3OD, 298 K) δ = 170.31, 149.48, 137.57, 123.05, 121.15, 121.09, 70.72, 22.33.

^{11}B{^{1}H}-NMR (128 MHz, CD_3OD, 298 K) δ = 31.93.

10. Synthesis of 6-phenoxybenzo[*c*][1,2]oxaborol-1(3*H*)-ol (5y)

The synthesis was performed according to a literature procedure: *ACS Chem. Biol.* **2020**, *15*, 1930–1941.[12]

2-bromo-4-phenoxybenzaldehyde[12]

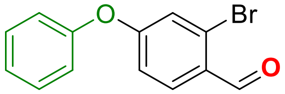

Chemical formula: $C_{13}H_9BrO_2$

Molecular weight: 277.12

^{1}H NMR (400 MHz, DMSO-d6, 298 K) δ = 10.11 (s, 1H, C*H*O), 7.87 (dq, 1H, *J* = 8.63, 1.60 Hz, ArC*H*), 7.48-7.53 (m, 2H, ArC*H*), 7.27-7.33 (m, 2H, ArC*H*), 7.18-7.22 (m, 2H, ArC*H*), 7.06-7.10 (m, 1H, ArC*H*).

4-phenoxy-2-(4,4,5,5-tetramethyl-1,3,2-dioxaborolan-2-yl)benzaldehyde[12]

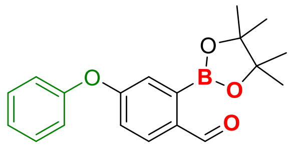

Chemical formula: $C_{19}H_{21}BO_4$

Molecular weight: 324.18

^{1}H NMR (400 MHz, $CDCl_3$, 298 K) δ = 10.39 (s, 1H, C*H*O), 7.92 (d, 1H, *J* = 8.53 Hz, ArC*H*), 7.37-7.43 (m, 3H, ArC*H*), 7.17-7.21 (m, 1H, ArC*H*), 7.02-7.07 (m, 3H, ArC*H*), 1.38 (s, 12H, CH_3).

^{13}C{^{1}H}-NMR (101 MHz, $CDCl_3$, 298 K) δ = 193.11, 161.85, 155.66, 136.18, 130.89, 130.18, 124.65, 124.43, 120.13, 119.31, 84.68, 24.98.

^{11}B{^{1}H}-NMR (128 MHz, $CDCl_3$, 298 K) δ = 30.63.

6-phenoxybenzo[*c*][1,2]oxaborol-1(3H)-ol (5y)[12]

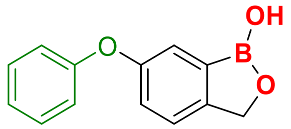

Chemical formula: $C_{13}H_{11}BO_3$

Molecular weight: 226.04

^{1}H NMR (400 MHz, CD_3OD, 298 K) δ = 7.32-7.39 (m, 3H, ArC*H*), 7,08-7.19 (m, 3H, ArC*H*), 6.95-6.97 (m, 2H, ArC*H*), 5.06 (s, 2H, C*H*$_2$).

^{13}C{^{1}H}-NMR (101 MHz, CD_3OD, 298 K) δ = 157.66, 156.65, 148.68, 129.48, 122.87, 122.33, 122.00, 119.19, 118.21, 70.67.

^{11}B{^{1}H}-NMR (128 MHz, CD_3OD, 298 K) δ = 31.68.

11.Synthesis of functionalized 3,4-dihydro-1*H*-benzo[*c*][1,2]oxaborinin-1-ol (Benzoxaborinin derivatives)

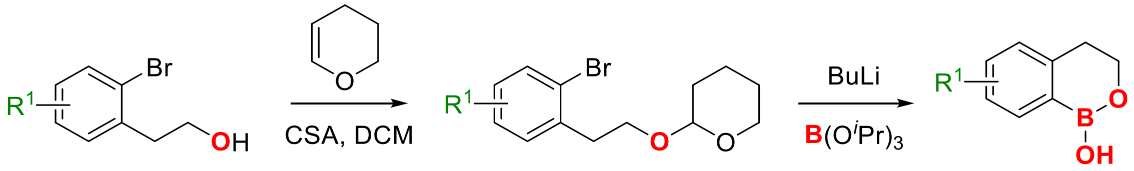

Reaction Procedure: At room temperature, in a schlenk tube, functionalized 2-(2-bromo-phenyl)-ethanol, 3,4-dihydro-2H-pyran followed by camphorsulfonic acid was added and dissolved in DCM. The mixture was stirred at room temperature for 2 h. Afterwards, K_2CO_3 was added to the reaction mixture, and the reaction mixture was filtered to remove the precipitate, the filtrate was washed with H_2O, and brine. The organic phase was dried over $MgSO_4$, filtered and the filtrate was concentrated under reduced pressure. The oily residue was applied to silica chromatography eluting with EtOAc/pentane (0:100 to 20:80) to give the corresponding product.

2-(2-bromophenethoxy)tetrahydro-2*H*-pyran[14]

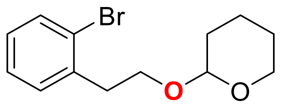

Prepared by following the general experimental procedure with: 2-(2-bromophenyl)-ethanol (3.7 g, 18.4 mmol), 3,4-dihydro-2*H*-pyran (2.5 mL, 27.7 mmol), camphorsulfonic acid (100 mg), and DCM (100 mL).

Chemical formula: $C_{13}H_{17}BrO_2$

Molecular weight: 285.18

^{1}H NMR (400 MHz, CD_2Cl_2, 298 K) δ = 7.46 (dd, 1H, *J* = 7.97, 1.26 Hz, ArC*H*), 7.14-7.23 (m, 2H, ArC*H*), 7.00 (td, 1H, *J* = 7.63, 1.86 Hz, ArC*H*), 4.54 (dd, 1H, *J* = 4.26, 2.91 Hz, C*H*), 3.88 (dt, 1H, *J* = 9.78, 7.21 Hz, C*H*), 3.69 (ddd, 1H, *J* = 11.24, 8.34, 3.28 Hz, C*H*), 3.57 (dt, 1H, *J* = 9.79, 7.12 Hz, C*H*), 3.37-3.42 (m, 1H, C*H*), 2.99 (t, 2H, *J* = 7.17 Hz, C*H*$_2$), 1.69-1.78 (m, 1H, C*H*), 1.59-1.66 (m, 1H, C*H*), 1.39-1.55 (m, 4H, C*H*$_2$).

^{13}C{^{1}H}-NMR (101 MHz, CD_2Cl_2, 298 K) δ = 138.51, 132.86, 131.34, 128.07, 127.41, 124.84, 98.79, 66.54, 62.27, 36.63, 30.77, 25.60, 19.60.

2-(2-bromo-4-(trifluoromethyl)phenethoxy)tetrahydro-2*H*-pyran

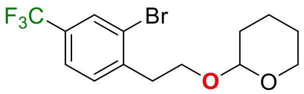

Prepared by following the general experimental procedure with: 2-(2-bromo-4-(trifluoromethyl)phenyl)ethan-1-ol (380.5 mg, 1.4 mmol), 3,4-dihydro-2*H*-pyran (179.2 mg, 2.13 mmol), camphorsulfonic acid (10 mg), and DCM (10 mL).

Chemical formula: $C_{14}H_{16}BrF_3O_2$

Molecular weight: 353.18

^{1}H NMR (400 MHz, $CDCl_3$, 298 K) δ = 7.73 (m, 1H, ArC*H*), 7.35-7.44 (m, 2H, ArC*H*), 4.53 (dd, 1H, *J* = 4.22, 2.72 Hz, C*H*), 3.88-3.94 (dt, 1H, *J* = 9.87, 6.89 Hz, C*H*), 3.56-3.69 (m, 2H, C*H*$_2$), 3.37-3.43 (m, 1H, C*H*), 3.04 (t, 2H, *J* = 6.50 Hz, C*H*$_2$), 1.58-1.76 (m, 2H, C*H*$_2$), 1.40-1.49 (m, 4H, C*H*$_2$).

^{13}C{^{1}H}-NMR (101 MHz, $CDCl_3$, 298 K) δ = 142.93, 131.61, 130.18, 130.52, 129.76 (q, *J* = 3.96 Hz), 124.17 (q, *J* = 3.66 Hz), 98.90, 65.93, 62.36, 36.56, 30.71, 25.53, 19.58.

2-(2-bromo-5-methoxyphenethoxy)tetrahydro-2*H*-pyran

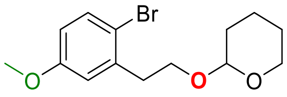

Prepared by following the general experimental procedure with: 2-(2-bromo-5-methoxyphenyl)ethan-1-ol (374.9 mg, 1.63 mmol), 3,4-dihydro-2*H*-pyran (205.7 mg, 2.45 mmol), camphorsulfonic acid (10 mg), and DCM (10 mL).

Chemical formula: $C_{14}H_{19}BrO_3$

Molecular weight: 315.21

^{1}H NMR (400 MHz, $CDCl_3$, 298 K) δ = 7.12 (d, 1H, *J* = 8.47 Hz, ArC*H*), 7.02 (d, 1H, *J* = 2.66 Hz, ArC*H*), 6.72 (dd, 1H, *J* = 8.47, 2.66 Hz, ArC*H*), 4.53 (dd, 1H, *J* = 4.29, 2.81 Hz, C*H*), 3.83 (dt, 1H, *J* = 9.67, 7.24 Hz, C*H*), 3.68-3.73 (m, 4H, C*H* & OCH_3), 3.53 (dt, 1H, *J* = 9.67, 7.17 Hz, C*H*), 3.37-3.43 (m, 1H, C*H*), 2.92 (t, 2H, *J* = 7.21 Hz, CH_2), 1.71-1.78 (m, 1H, C*H*), 1.59-1.66 (m, 1H, C*H*), 1.41-1.54 (m, 4H, CH_2).

Reaction Procedure: Under argon atmosphere, at −78 °C, functionalized 2-[2-(2-bromo-phenyl)-ethoxy]-tetrahydro-pyran in THF followed by the slow addition of *n*-BuLi were added. Triisopropyl borate was then added, and the reaction mixture was allowed to warm to room temperature gradually and stirred overnight. After carefully adding HCl (10 mL, 6N), the solution was stirred at room temperature for another 1 h and then poured into a mixture of EtOAc and H_2O. The layers were separated, and the aqueous phase was extracted with EtOAc. Combined organic extracts was washed with H_2O, brine, dried over $MgSO_4$, filtered and the filtrate was concentrated under reduced pressure. The residue was applied to silica chromatography eluting with EtOAc/Heptanes (0:100 to 100:0) to give corresponding product.

3,4-dihydro-1*H*-benzo[*c*][1,2]oxaborinin-1-ol (7a)[14]

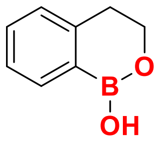

Prepared by following the general experimental procedure with: 2-(2-bromophenethoxy)tetrahydro-2*H*-pyran (1.0 g, 3.5 mmol), *n*-BuLi (2.4 mL, 1.6 M solution in THF, 3.8 mmol), Triisopropyl borate (1.2 mL, 5.25 mmol), and THF (20 mL).

Chemical formula: $C_8H_9BO_2$

Molecular weight: 147.97

^{1}H NMR (400 MHz, CD_2Cl_2, 298 K) δ = 7.69-7.72 (m, 1H, ArC*H*), 7.41 (td, 1H, *J* = 7.53, 1.48 Hz, ArC*H*), 7.25-7.29 (m, 1H, ArC*H*), 7.19-7.21 (m, 1H, ArC*H*), 4.68 (s, 1H, O*H*), 4.20 (t, 2H, *J* = 6.01 Hz, CH_2), 2.95 (t, 2H, *J* = 6.01 Hz, CH_2).

^{13}C{^{1}H}-NMR (101 MHz, CD_2Cl_2, 298 K) δ = 146.38, 133.15, 131.81, 127.20, 126.54, 64.75, 32.83.

^{11}B{^{1}H}-NMR (128 MHz, $CDCl_3$, 298 K) δ = 28.43.

7-(trifluoromethyl)-3,4-dihydro-1*H*-benzo[*c*][1,2]oxaborinin-1-ol (7b)

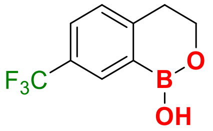

Prepared by following the general experimental procedure with: 2-(2-bromo-4-(trifluoromethyl)phenethoxy)tetrahydro-2H-pyran (428.0 mg, 1.2 mmol), *n*-BuLi (0.9 mL, 1.6 M solution in THF, 1.8 mmol), Triisopropyl borate (343.0 mg, 1.82 mmol), and THF (7.5 mL).

Chemical formula: $C_9H_8BF_3O_2$

Molecular weight: 215.97

^{1}H NMR (400 MHz, CD_3OD, 298 K) δ = 7.88 (s, 1H, ArC*H*), 7.66 (d, 1H, *J* = 8.01 Hz, ArC*H*), 7.39 (d, 1H, 7.97 Hz, ArC*H*), 4.26 (t, 2H, *J* = 6.02 Hz, CH_2), 3.02 (t, 2H, *J* = 6.10 Hz, CH_2).

^{13}C{^{1}H}-NMR (101 MHz, CD_3OD, 298 K) δ = 151.20, 130.10 (q, *J* = 3.85 Hz), 128.53 (q, *J* = 3.77 Hz), 128.43, 125.93 (d, *J* = 271.05), 124.59, 65.12, 33.23.

^{11}B{^{1}H}-NMR (128 MHz, CD_3OD, 298 K) δ = 27.23.

7-methoxy-3,4-dihydro-1*H*-benzo[*c*][1,2]oxaborinin-1-ol (7c)

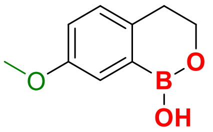

Prepared by following the general experimental procedure with: 2-(2-bromo-5-methoxyphenethoxy)tetrahydro-2*H*-pyran (476.0 mg, 1.5 mmol), *n*-BuLi (1.0 mL, 1.6 M solution in THF, 1.7 mmol), Triisopropyl borate (427.6 mg, 2.27 mmol), and THF (10 mL).

Chemical formula: $C_9H_{11}BO_3$

Molecular weight: 177.99

^{1}H NMR (400 MHz, $CDCl_3$, 298 K) δ = 7.28 (d, 1H, *J* = 2.82 Hz, ArC*H*), 7.11 (d, 1H, *J* = 8.28 Hz, ArC*H*), 6.97 (dd, 1H, *J* = 8.27, 2.82 Hz, ArC*H*), 4.68 (br s, 1H, O*H*), 4.19-4.22 (m, 2H, CH_2), 3.83 (s, 3H, OCH_3), 2.90 (t, 2H, *J* = 5.99 Hz, CH_2).

^{13}C{^{1}H}-NMR (101 MHz, $CDCl_3$, 298 K) δ = 158.18, 137.99, 128.12, 118.51, 116.67, 64.83, 55.52, 31.70.

^{11}B{^{1}H}-NMR (128 MHz, $CDCl_3$, 298 K) δ = 28.46.

12.Synthesis of 4,5-dihydrobenzo[*c*][1,2]oxaborepin-1(3*H*)-ol (7d) (Benzoxaboripin)

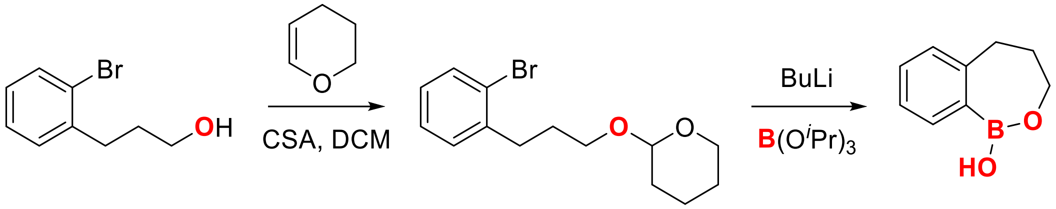

Reaction Procedure: At room temperature, in a schlenk tube, 3-(2-bromophenyl)propan-1-ol (400.0 mg, 1.9 mmol), 3,4-dihydro-2*H*-pyran (234.7 mg, 2.8 mmol) followed by camphorsulfonic acid (10 mg) was added and dissolved in DCM (10 mL). The mixture was stirred at room temperature for 2 h. Afterwards, K_2CO_3 (30 mg) was added to the reaction mixture, and the reaction mixture was filtered to remove the precipitate, the filtrate was washed with H_2O (100 mL), and brine (100 mL). The organic phase was dried over $MgSO_4$, filtered and the filtrate was concentrated under reduced pressure. The oily residue was applied to silica chromatography eluting with EtOAc/pentane (0:100 to 50:50) to give 2-[2-(2-bromo-phenyl)-ethoxy]-tetrahydro-pyran as a colorless oil.

2-(3-(2-bromophenyl)propoxy)tetrahydro-2*H*-pyran[15]

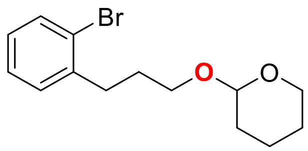

Chemical formula: $C_{14}H_{19}BrO_2$
Molecular weight: 299.21
^{1}H NMR (400 MHz, CD_3OD, 298 K) δ = 7.51-7.53 (m, 1H, ArC*H*), 7.20-7.25 (m, 2H, ArC*H*), 7.05 (ddd, 1H, *J* = 7.98, 6.25, 2.77 Hz), 4.60 (dd, 1H, *J* = 4.47, 2.86 Hz, C*H*), 3.86-3.92 (m, 1H, C*H*), 3.80 (dt, 1H, *J* = 9.76, 6.51 Hz, C*H*), 3.47-3.54 (m, 1H, C*H*), 3.44 (dt, 1H, *J* = 9.75, 6.43 Hz, C*H*), 2.77-2.90 (m, 2H, C*H*), 1.91-1.96 (m, 2H, CH_2), 1.82-1.89 (m, 1H, C*H*), 1.70-1.77 (m, 1H, C*H*), 1.58-1.64 (m, 2H, CH_2), 1.51-1.56 (m, 2H, CH_2).
^{13}C{^{1}H}-NMR (101 MHz, $CDCl_3$, 298 K) δ = 141.46, 132.93, 130.55, 127.67, 127.50, 124.65, 98.97, 66.83, 62.51, 33.00, 30.92, 29.89, 25.65, 19.79.

Reaction Procedure: Under argon atmosphere, at –78 ºC, 2-(3-(2-bromophenyl)propoxy)tetrahydro-2*H*-pyran (508.0 mg, 1.70 mmol) in THF (10 mL) followed by the slow addition of *n*BuLi (1.16 mL, 1.6 M solution in THF, 1.9 mmol) were added in a schlenk tube. Triisopropyl borate (480.8 mg, 2.56 mmol) was then added, and the reaction mixture was allowed to warm to room temperature gradually and stirred overnight. After carefully adding HCl (10 mL, 6N), the yellowish solution was stirred at room temperature

for another 1 h and then poured into a mixture of EtOAc (30 mL) and H_2O (20 mL). The layers were separated, and the aqueous phase was extracted with EtOAc (3 x 30 mL). Combined organic extracts was washed with H_2O (50 mL), brine (50 mL), dried over $MgSO_4$, filtered and the filtrate was concentrated under reduced pressure. The oily residue was applied to silica chromatography eluting with EtOAc/Heptanes (0:100 to 100:0) to give 4,5-dihydrobenzo[c][1,2]oxaborepin-1(3H)-ol.

4,5-dihydrobenzo[*c*][1,2]oxaborepin-1(3*H*)-ol (7d)[15]

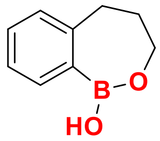

Chemical formula: $C_9H_{11}BO_2$

Molecular weight: 161.99

^{1}H NMR (400 MHz, CD_3OD, 298 K) δ = 7.54 (d, 1H, *J* = 7.29 Hz, ArC*H*), 7.30 (td, 1H, *J* = 7.50, 1.51 Hz, ArC*H*), 7.13-7.20 (m, 2H, ArC*H*), 3.93 (t, 2H, *J* = 6.54 Hz, CH_2), 2.83 (t, 2H, *J* = 6.91 Hz, CH_2), 1.99-2.05 (m, 2H, CH_2).

^{13}C{^{1}H}-NMR (101 MHz, CD_3OD, 298 K) δ = 144.70, 133.19, 129.98, 127.71, 125.11, 63.52, 31.84, 30.03.

^{11}B{^{1}H}-NMR (128 MHz, CD_3OD, 298 K) δ = 30.04.

13.Synthesis of catalysts

Synthesis of Rh-CAAC catalyst 1

The synthesis was performed according to a literature procedure.[16]

Synthesis of [(η^5-C_5Me_5)Rh(ppy)H] catalyst 3

The synthesis was performed according to a literature procedure.[17]

14.Catalyst optimization for the hydrogenation of benzoxaborole (5a)

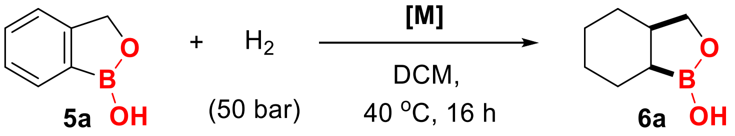

Organometallic complexes or heterogeneous catalysts (3 mol%), and benzoxaborole (**5a**) (13.4 mg, 0.1 mmol) were filled to an oven-dried 4 mL screw-cap vial equipped with a stirring bar. Dichloromethane (0.5 mL) was added under argon atmosphere. The glass vial was placed in a 150 mL stainless steel autoclave under argon atmosphere. The autoclave was sealed, pressurized, and depressurized with hydrogen gas three times before the 50-bar pressure was set. The reaction mixture was stirred at the 40 °C temperature for 16 h. After the autoclave was carefully depressurized, 1,2,4,5-tetramethylbenzene as an internal standard was added and the mixture was stirred vigorously for 5 min. After filtration over Whatman® filter, conversion, and yield were determined by NMR analysis (see table 1 in the manuscript).

15.Temperature optimization for the hydrogenation of benzoxaborole (5a) using Rh-CAAC (1)

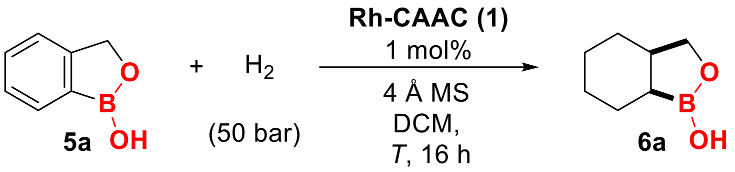

[Rh(CAAC)(COD)Cl] **1** (0.6 mg, 1 mol%), benzoxaborole (**5a**) (13.4 mg, 0.1 mmol), and 4 Å molecular sieves (50 mg) were filled to an oven-dried 4 mL screw-cap vial equipped with a stirring bar. Dichloromethane (0.5 mL) was added under argon atmosphere. The glass vial was placed in a 150 mL stainless steel autoclave under argon atmosphere. The autoclave was sealed, pressurized, and depressurized with hydrogen gas three times before the 50-bar pressure was set. The reaction mixture was stirred at the indicated temperature for 16 h. After the autoclave was carefully depressurized, 1,2,4,5-tetramethylbenzene as an internal standard was added and the mixture was stirred vigorously for 5 min. After filtration over Whatman® filter, conversion, and yield were determined by NMR analysis.

Table S1: Temperature optimization for the hydrogenation of benzoxaborole (5a)

#	Temperature (°C)	NMR Yield (%)
**1**	25	80
**2**	40	>99
**3**	50	>99

16.Pressure optimization for the hydrogenation of benzoxaborole (5a) using Rh-CAAC (1)

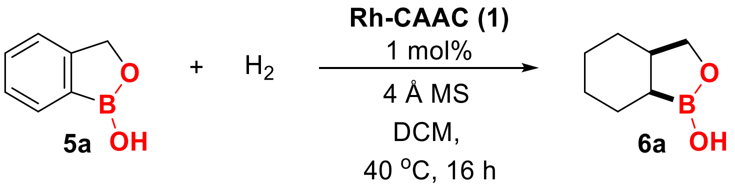

[Rh(CAAC)(COD)Cl] **1** (0.6 mg, 1 mol%), benzoxaborole (**5a**) (13.4 mg, 0.1 mmol), and 4 Å molecular sieves (50 mg) were filled to an oven-dried 4 mL screw-cap vial equipped with a stirring bar. Dichloromethane (0.5 mL) was added under argon atmosphere. The glass vial was placed in a 150 mL stainless steel autoclave under argon atmosphere. The autoclave was sealed, pressurized, and depressurized with hydrogen gas three times before the indicated pressure was set. The reaction mixture was stirred at the 40 °C temperature for 16 h. After the autoclave was carefully depressurized, 1,2,4,5-tetramethylbenzene as

an internal standard was added and the mixture was stirred vigorously for 5 min. After filtration over Whatman® filter, conversion, and yield were determined by NMR analysis.

Table S2: Pressure optimization for the hydrogenation of benzoxaborole (5a)

#	Pressure (bar)	NMR Yield (%)
**1**	20	30
**2**	30	93
**3**	40	>99
**4**	50	>99
**5**	60	66
**6**	80	62

17. Solvent optimization for the hydrogenation of benzoxaborole (5a) using Rh-CAAC (1)

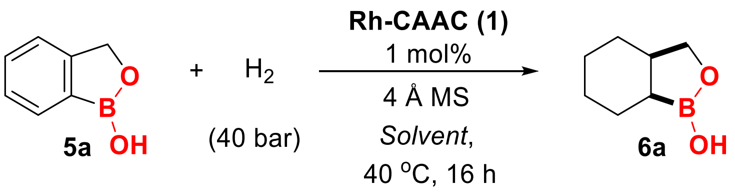

[Rh(CAAC)(COD)Cl] **1** (0.6 mg, 1 mol%), benzoxaborole (**5a**) (13.4 mg, 0.1 mmol), and 4 Å molecular sieves (50 mg) were filled to an oven-dried 4 mL screw-cap vial equipped with a stirring bar. Solvent (0.5 mL) was added under argon atmosphere. The glass vial was placed in a 150 mL stainless steel autoclave under argon atmosphere. The autoclave was sealed, pressurized and depressurized with hydrogen gas three times before the pressure was set to 40 bar. The reaction mixture was stirred at the 40 °C temperature for 16 h. After the autoclave was carefully depressurized, 1,2,4,5-tetramethylbenzene as an internal standard was added and the mixture was stirred vigorously for 5 min. After filtration over Whatman® filter, conversion, and yield determined by NMR analysis.

Table S3: Solvent optimization for the hydrogenation of benzoxaborole (5a)

#	Solvent	NMR Yield (%)
**1**	DCM	>99
**2**	$CHCl_3$	0
**3**	CH_3OH	0
**4**	EtOAc	87
**5**	THF	>99

**6**	Hexane	89
**7**	CH_3CN	0
**8**	1,4-dioxane	76

18.Effect of pressure in diastereomeric ratio for the hydrogenation of benzoxaborole derivatives using Rh-CAAC (1)

18.1. Hydrogenation of benzoxaborole (5a) at different pressures

[Rh(CAAC)(COD)Cl] **1** (1.7 mg, 1 mol%), benzoxaborole (**5a**) (40.21 mg, 0.3 mmol), and 4 Å molecular sieves (100 mg) were filled to an oven-dried 4 mL screw-cap vial equipped with a stirring bar. Dichloromethane (1.0 mL) was added under argon atmosphere. The glass vial was placed in a 150 mL stainless steel autoclave under argon atmosphere. The autoclave was sealed, pressurized, and depressurized with hydrogen gas three times before the indicated pressure was set. The reaction mixture was stirred at the 40 ^{o}C temperature for 24 h. After the autoclave was carefully depressurized, mesitylene as an internal standard was added and the mixture was stirred vigorously for 5 min. After filtration over Whatman® filter, yield and d.r. were determined by NMR analysis.

Table S4: Hydrogenation of benzoxaborole (5a) at different pressures

#	Pressure (bar)	NMR Yield (%)	d.r.
**1**	20	30	n.d.
**2**	30	93	80:20
**3**	40	>99	80:20
**4**	50	>99	81:19
**5**	60	66	83:17
**7**	80	62	85:15

Discussion: Increasing the pressure above 50 bars for the hydrogenation of benzoxaborole (**5a**) enhances the diastereomeric ratio. However, the yield was decreased for the product **6a** and ^{1}H and ^{13}C NMR confirmed the formation of side products. n.d.: not determined.

18.2. Hydrogenation of 6-methylbenzo[c][1,2]oxaborol-1(3H)-ol (5e) at different pressures

[Rh(CAAC)(COD)Cl] **1** (1.7 mg, 1 mol%), 6-methylbenzo[c][1,2]oxaborol-1(3H)-ol (**5e**) (44.42 mg, 0.3 mmol), and 4 Å molecular sieves (100 mg) were filled to an oven-dried 4 mL screw-cap vial equipped with a stirring bar. Dichloromethane (1.0 mL) was added under argon atmosphere. The glass vial was placed in a 150 mL stainless steel autoclave under argon atmosphere. The autoclave was sealed, pressurized, and

depressurized with hydrogen gas three times before the indicated pressure was set. The reaction mixture was stirred at the 40 °C temperature for 24 h. After the autoclave was carefully depressurized, mesitylene as an internal standard was added and the mixture was stirred vigorously for 5 min. After filtration over Whatman® filter, yield and d.r. were determined by NMR analysis.

Table S5: Hydrogenation of 6-methylbenzo[c][1,2]oxaborol-1(3H)-ol (5e) at different pressures

#	Pressure (bar)	NMR Yield (%)	d.r.
**1**	40	>99	55:45
**2**	80	82	56:44

Discussion: Increasing the pressure from 40 to 80 bars did not enhance the diastereomeric ratio. However, the yield was decreased for the product **6e** and ^{1}H and ^{13}C NMR confirmed the formation of side products.

18.3. Hydrogenation of 3-methylbenzo[c][1,2]oxaborol-1(3H)-ol (5k) at different pressures

[Rh(CAAC)(COD)Cl] **1** (1.7 mg, 1 mol%), 3-methylbenzo[c][1,2]oxaborol-1(3H)-ol (**5k**) (44.42 mg, 0.3 mmol), and 4 Å molecular sieves (100 mg) were filled to an oven-dried 4 mL screw-cap vial equipped with a stirring bar. Dichloromethane (1.0 mL) was added under argon atmosphere. The glass vial was placed in a 150 mL stainless steel autoclave under argon atmosphere. The autoclave was sealed, pressurized, and depressurized with hydrogen gas three times before the indicated pressure was set. The reaction mixture was stirred at the 40 °C temperature for 24 h. After the autoclave was carefully depressurized, mesitylene as an internal standard was added and the mixture was stirred vigorously for 5 min. After filtration over Whatman® filter, yield and d.r. were determined by NMR analysis.

Table S6: Hydrogenation of 3-methylbenzo[c][1,2]oxaborol-1(3H)-ol (5k) at different pressures

#	Pressure (bar)	NMR Yield (%)	d.r.
**1**	40	>99	83:17
**2**	80	>99	83:17

Discussion: Increasing the pressure from 40 to 80 bars did not enhance the diastereomeric ratio. The NMR yield for the product **6k** at both pressures was same.

18.4. Hydrogenation of 3-isopropylbenzo[c][1,2]oxaborol-1(3H)-ol (5o) at different pressures

[Rh(CAAC)(COD)Cl] **1** (1.7 mg, 1 mol%), 3-isopropylbenzo[c][1,2]oxaborol-1(3H)-ol (**5o**) (52.83 mg, 0.3 mmol), and 4 Å molecular sieves (100 mg) were filled to an oven-dried 4 mL screw-cap vial equipped with a stirring bar. Dichloromethane (1.0 mL) was added under argon atmosphere. The glass vial was placed in a 150 mL stainless steel autoclave under argon atmosphere. The autoclave was sealed, pressurized, and

depressurized with hydrogen gas three times before the indicated pressure was set. The reaction mixture was stirred at the 40 °C temperature for 24 h. After the autoclave was carefully depressurized, mesitylene as an internal standard was added and the mixture was stirred vigorously for 5 min. After filtration over Whatman® filter, yield and d.r. were determined by NMR analysis.

Table S7: Hydrogenation of 3-isopropylbenzo[c][1,2]oxaborol-1(3H)-ol (5o) at different pressures

#	Pressure (bar)	NMR Yield (%)	d.r.
**1**	40	>99	72:28
**2**	80	>99	71:29

Discussion: Increasing the pressure from 40 to 80 bars did not enhance the diastereomeric ratio. The NMR yield for the product **6e** at both pressures was same.

19. Evaluation of Rhodium on carbon (Rh/C) as a catalyst for the hydrogenation of benzoxaborole derivatives

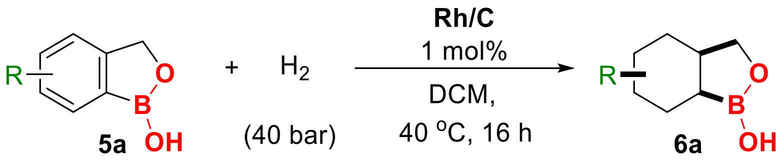

Rhodium on carbon (Rh/C) (6.12 mg, 1 mol%), and benzoxaborole derivatives (0.3 mmol) were filled to an oven-dried 4 mL screw-cap vial equipped with a stirring bar. Dichloromethane (DCM) (1 mL) was added under argon atmosphere. The glass vial was placed in a 150 mL stainless steel autoclave under an argon atmosphere. The autoclave was sealed, pressurized, and depressurized with hydrogen gas three times before the 40-bar pressure was set. The reaction mixture was stirred at the 40 °C temperature for 16 h. After the autoclave was carefully depressurized, mesitylene as an internal standard was added and the mixture was stirred vigorously for 5 min. After filtration over Whatman® filter, conversion and yield were determined by NMR analysis.

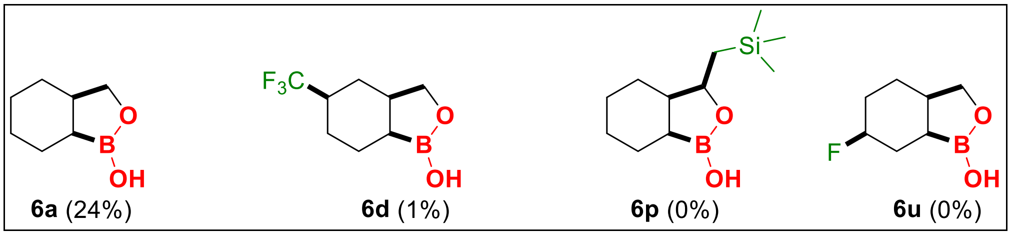

20. General procedure for the hydrogenation of benzoxaborole derivatives

[Rh(CAAC)(COD)Cl] **1** (1.71 mg, 1 mol%), benzoxaborole derivatives (0.3 mmol), and 4 Å molecular sieves (100 mg) were filled to an oven-dried 4 mL screw-cap vial equipped with a stirring bar. Dichloromethane (DCM) (1 mL) was added under argon atmosphere. The glass vial was placed in a 150 mL stainless steel autoclave under an argon atmosphere. The autoclave was sealed, pressurized, and depressurized with hydrogen gas three times before the 40-bar pressure was set. The reaction mixture was stirred at the 40 °C temperature for 24 h. After the autoclave was carefully depressurized, 1,2,4,5-tetramethylbenzene as an internal standard was added and the mixture was stirred vigorously for 5 min. After filtration over Whatman® filter, conversion and diastereomeric ratio (d.r.) were determined by NMR analysis. The crude reaction mixture was applied to silica chromatography eluting with EtOAc/pentane (50:50 to 100:0) to give desired functionalized hexahydrobenzoxaborol product. These compounds were found to be sensitive for GC measurements, therefore, the diastereomeric ratio (d.r.) of the compounds were calculated using NMR.

Hexahydrobenzo[*c*][1,2]oxaborol-1(3*H*)-ol (6a)

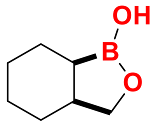

Prepared by following the general experimental procedure with: [Rh(CAAC)(COD)Cl] **1** (1.71 mg, 1 mol%), Benzoxaborole (40.22 mg, 0.3 mmol), H_2 (40 bar), 4 Å molecular sieves (100 mg), DCM (1 mL), 40 °C, and 24 h.

Chemical formula: $C_7H_{13}BO_2$

Molecular weight: 139.99

^{1}H NMR (400 MHz, CD_2Cl_2, 298 K) δ = 5.22 (br s, 1H, O*H*), 3.90-3.93 (dd, 1H, *J* = 8.84, 5.16, C*H*), 3.66-3.69 (dd, 1H, *J* = 8.87, 2.35, C*H*), 2.13-2.20 (m, 1H, C*H*), 1.64-1.74 (m, 2H, CH_2), 1.46-1.62 (m, 3H, C*H* & CH_2), 1.40-1.44 (m, 1H, C*H*), 1.10-1.26 (m, 3H, CH_2 & C*H*).

^{13}C{^{1}H}-NMR (101 MHz, CD_2Cl_2, 298 K) δ = 72.66, 38.72, 29.11, 25.91, 24.85, 23.62.

^{11}B{^{1}H}-NMR (128 MHz, CD_2Cl_2, 298 K) δ = 35.80.

Diastereomeric ratio (d.r.): 80:20.

HRMS(ESI$^-$) calcd. for $[C_7H_{12}O_2B]^-$: 139.09372; found 139.09371.

Isolated Yield: 92% (38.6 mg).

5-methoxyhexahydrobenzo[*c*][1,2]oxaborol-1(3*H*)-ol (6b)

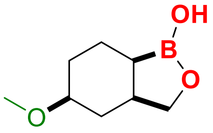

Prepared by following the general experimental procedure with: [Rh(CAAC)(COD)Cl] **1** (1.71 mg, 1 mol%), 5-methoxybenzo[*c*][1,2]oxaborol-1(3*H*)-ol (49.19 mg, 0.3 mmol), H_2 (40 bar), 4 Å molecular sieves (100 mg), DCM (1 mL), 40 ºC, and 24 h.

Chemical formula: $C_8H_{15}BO_3$

Molecular weight: 170.02

^{1}H NMR (400 MHz, CD_2Cl_2, 298 K) δ = 5.52 (br s, 1H, O*H*), 3.86-3.89 (dd, 1H, *J* = 8.89, 4.74 Hz, C*H*), 3.62 (d, 1H, *J* = 8.88 Hz, C*H*), 3.21 (s, 3H, OCH_3), 2.92-2.99 (tt, 1H, *J* = 10.80, 3.54 Hz, C*H*), 2.13-2.20 (dq, 1H, *J* = 11.65, 5.59 Hz, C*H*), 1.80-1.94 (m, 3H, CH_2 & C*H*), 1.40-1.50 (m, 1H, C*H*), 1.30-1.34 (m, 1H, C*H*), 0.87-1.04 (m, 2H, CH_2).

^{13}C{^{1}H}-NMR (101 MHz, CD_2Cl_2, 298 K) δ = 78.70, 72.59, 55.59, 38.66, 35.28, 31.16, 21.81.

^{11}B{^{1}H}-NMR (128 MHz, CD_2Cl_2, 298 K) δ = 35.66.

Diastereomeric ratio (d.r.): 89:11.

HRMS(ESI$^+$) calcd. for $[C_8H_{15}O_3BNa]^+$: 193.10080; found 193.10173.

Isolated Yield: 80% (40.8 mg).

Hexahydrobenzo[*c*][1,2]oxaborole-1,5(3*H*)-diol (6c)

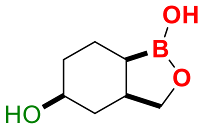

Prepared by following the general experimental procedure with: [Rh(CAAC)(COD)Cl] **1** (1.71 mg, 1 mol%), benzo[*c*][1,2]oxaborole-1,6(3*H*)-diol (44.7 mg, 0.3 mmol), H_2 (40 bar), 4 Å molecular sieves (100 mg), DCM (1 mL), 40 ºC, and 24 h.

Chemical formula: $C_7H_{13}BO_3$

Molecular weight: 155.99

^{1}H NMR (400 MHz, CD_3OD, 298 K) (mixture of diastereoisomers) δ = 3.85-3.88 (dd, 1H, *J* = 9.15,), 3.56-3.64 (d, 1H, *J* = 9.18 Hz, C*H*), 3.45-3.47 (m, 0.12H, C*H*), 3.28-3.35 (tt, 0.89H, *J* = 11.37, 3.91 Hz, C*H*), 2.35-2.43 (m, 0.13H, C*H*), 2.12-2.19 (m, 0.88H, C*H*), 1.68-1.84 (m, 3H, CH_2 & C*H*), 1.39-1.50 (m, 1H, C*H*), 1.19-1.26 (m, 1H, C*H*), 0.85-1.01 (m, 2H, CH_2).

^{13}C{^{1}H}-NMR (101 MHz, CD_3OD, 298 K) (mixture of diastereoisomers) δ = 73.52 (minor), 73.41 (major), 70.21 (major), 67.29 (minor), 39.71 (major), 39.37 (major), 37.58 (minor), 35.35 (major), 33.94 (minor), 22.63 (major).

^{11}B{^{1}H}-NMR (128 MHz, CD_2Cl_2, 298 K) δ = 35.35.

Diastereomeric ratio (d.r.): 88:12.

HRMS(ESI$^+$) calcd. for $[C_7H_{13}O_3BNa]^+$: 179.08513; found 179.08499.

Isolated Yield: 90% (42.1 mg).

5-(trifluoromethyl)hexahydrobenzo[*c*][1,2]oxaborol-1(3*H*)-ol (6d)

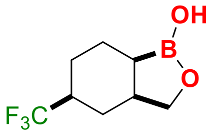

Prepared by following the general experimental procedure with: [Rh(CAAC)(COD)Cl] **1** (1.71 mg, 1 mol%), 6-(trifluoromethyl)benzo[*c*][1,2]oxaborol-1(3*H*)-ol (60.6 mg, 0.3 mmol), H_2 (40 bar), 4 Å molecular sieves (100 mg), DCM (1 mL), 40 °C, and 24 h.

Chemical formula: $C_8H_{12}BF_3O_2$

Molecular weight: 207.99

^{1}H NMR (400 MHz, CD_2Cl_2, 298 K) δ = 5.46 (br s, 1H, O*H*), 3.90-3.93 (dd, 1H, *J* = 9.13, 4.54 Hz, C*H*), 3.62 (d, 1H, *J* = 9.09 Hz, C*H*), 2.13-2.20 (dq, 1H, *J* = 11.50, 5.40 Hz, C*H*), 1.77-1.95 (m, 4H, CH_2), 1.44-1.55 (m, 2H, CH_2), 0.97-1.18 (m, 2H, CH_2).

^{13}C{^{1}H}-NMR (101 MHz, CD_2Cl_2, 298 K) δ = 124.09-132.39 (q, *J* = 278.39), 72.66, 41.59 (q, *J* = 26.52), 37.96, 28.24-28.32 (q, *J* = 2.60), 24.49-24.57 (q, *J* = 2.63), 22.40.

^{11}B{^{1}H}-NMR (128 MHz, CD_2Cl_2, 298 K) δ = 35.52.

Diastereomeric ratio (d.r.): 92:8.

HRMS(ESI$^-$) calcd. for $[C_8H_{11}O_2BF_3]^-$: 207.08112; found 207.08096.

Isolated Yield: 69% (43.1 mg).

5-methylhexahydrobenzo[*c*][1,2]oxaborol-1(3*H*)-ol (6e)

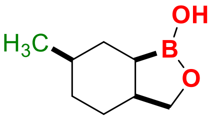

Prepared by following the general experimental procedure with: [Rh(CAAC)(COD)Cl] **1** (1.71 mg,1 mol%), 5-methylbenzo[*c*][1,2]oxaborol-1(3*H*)-ol (44.4 mg, 0.3 mmol), H_2 (40 bar), 4 Å molecular sieves (100 mg), DCM (1 mL), 40 °C, and 24 h.

Chemical formula: $C_8H_{15}BO_2$

Molecular weight: 154.02

^{1}H NMR (400 MHz, CD_2Cl_2, 298 K) δ = 4.90 (br s, 1H, O*H*), 3.83-3.86 (dd, 1H, *J* = 8.85, 4.55 Hz, C*H*), 3.56 (d, 1H, *J* = 8.86 Hz, C*H*), 1.96-2.03 (dq, 1H, *J* = 11.70, 5.52 Hz, C*H*), 1.72 (dt, 1H, C*H*), 1.60-1.66 (m, 1H, C*H*), 1.48-1.53

(m, 2H, CH_2), 1.41-1.43 (m, 1H, CH), 1.05-1.15 (m, 3H, CH_2 & CH), 0.80 (d, 3H, J = 5.96 Hz, CH_3).

^{13}C{^{1}H}-NMR (101 MHz, CD_2Cl_2, 298 K) δ = 73.04, 38.53, 33.99, 32.67, 32.10, 30.03, 22.85.

^{11}B{^{1}H}-NMR (128 MHz, CD_2Cl_2, 298 K) δ = 35.82.

Diastereomeric ratio (d.r.): 55:45.

HRMS(ESI$^-$) calcd. for $[C_8H_{14}O_2B]^-$: 153.10829; found 153.10930.

Isolated Yield: 94% (43.5 mg),

6-methoxyhexahydrobenzo[*c*][1,2]oxaborol-1(3*H*)-ol (6f)

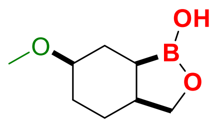

Prepared by following the general experimental procedure with: [Rh(CAAC)(COD)Cl] **1** (1.71 mg, 1 mol%), 6-methoxybenzo[*c*][1,2]oxaborol-1(3*H*)-ol (49.19 mg, 0.3 mmol), H_2 (40 bar), 4 Å molecular sieves (100 mg), DCM (1 mL), 40 °C, and 24 h.

Chemical formula: $C_8H_{15}BO_3$

Molecular weight: 170.02

^{1}H NMR (400 MHz, CD_2Cl_2, 298 K) (mixture of diastereoisomers) δ = 5.25 (br s, 1H, OH), 3.87-3.95 (m, 1H, CH), 3.68-3.73 (m, 1H, CH), 3.20-3.33 (m, 4H, OCH_3, CH), 2.06-2.22 (m, 1H, CH), 1.64-1.78 (m, 2H, CH_2), 1.42-1.62 (m, 3H, CH_2 & CH), 1.08-1.38 (m, 2H, CH_2).

^{13}C{^{1}H}-NMR (101 MHz, CD_2Cl_2, 298 K) (mixture of diastereoisomers) δ = 78.62 (minor), 76.02 (major), 72.46 (minor), 71.98 (major), 55.66, 38.17 (minor), 37.81 (major), 30.28 (minor), 29.41 (minor), 27.81 (major), 27.51 (major), 23.09 (major).

^{11}B{^{1}H}-NMR (128 MHz, CD_2Cl_2, 298 K) (mixture of diastereoisomers) δ = 35.67.

Diastereomeric ratio (d.r.): 73:27.

HRMS(ESI$^+$) calcd. for $[C_8H_{15}O_3BNa]^+$: 193.10080; found 193.10071.

Isolated Yield: 74% (37.8 mg).

***tert*-butyl (1-hydroxyoctahydrobenzo[*c*][1,2]oxaborol-6-yl)carbamate (6g)**

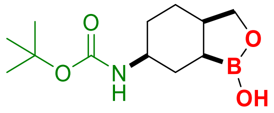

Prepared by following the general experimental procedure with: [Rh(CAAC)(COD)Cl] **1** (1 mol%), *tert*-butyl (1-hydroxy-1,3-

dihydrobenzo[*c*][1,2]oxaborol-6-yl)carbamate (74.7 mg, 0.3 mmol), H_2 (40 bar), 4 Å molecular sieves (100 mg), DCM (1 mL), 40 °C, and 24 h.

Chemical formula: $C_{12}H_{22}BNO_4$

Molecular weight: 255.12

^{1}H NMR (400 MHz, CD_3OD, 298 K) δ = 3.79-3.86 (m, 1H, C*H*), 3.57-3.76 (m, 1H, C*H*), 3.17-3.22 (m, 1H, C*H*), 1.91-2.19 (m, 1H, C*H*), 1.41-1.73 (m, 4H, C*H*$_2$), 1.33 (s, 9H, CH_3), 0.93-1.29 (m, 3H, CH_2 & C*H*).

^{13}C{^{1}H}-NMR (101 MHz, CD_3OD, 298 K) δ = 156.32 (minor), 156.30 (major), 78.42 (major), 72.02 (minor), 53.44 (major), 48.49 (major), 37.52 (major), 36.50 (major), 34.60 (minor), 31.31 (major), 29.39 (major), 27.90 (major), 27.78 (minor), 27.46 (minor), 27.44 (major), 23.24 (major), 21.61 (minor).

^{11}B{^{1}H}-NMR (128 MHz, CD_3OD, 298 K) δ = 35.25.

Diastereomeric ratio (d.r.): 67:33.

HRMS(ESI$^+$) calcd. for $[C_{12}H_{22}NO_4BNa]^+$: 278.15364; found 278.15340.

Isolated Yield: 92% (70.4 mg).

5a,6,7,8,9,9a-hexahydronaphtho[1,2-*c*][1,2]oxaborol-1(3*H*)-ol (6h)

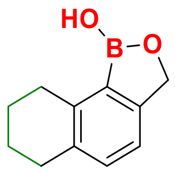

Prepared by following the general experimental procedure with: [Rh(CAAC)(COD)Cl] **1** (1.71, 1 mol%), Naphtho[1,2-*c*][1,2]oxaborol-1(3*H*)-ol (55.2 mg, 0.3 mmol), H_2 (40 bar), 4 Å molecular sieves (100 mg), DCM (1 mL), 40 °C, 24 h.

Chemical formula: $C_{11}H_{15}BO_2$

Molecular weight: 190.05

^{1}H NMR (400 MHz, CD_2Cl_2, 298 K) δ = 7.08 (d, 1H, *J* = 7.75 Hz, C*H*$_{Ar}$), 6.97 (d, 1H, *J* = 7.77 Hz, C*H*$_{Ar}$), 5.34 (br s, 1H, O*H*), 4.91 (s, 2H, C*H*$_2$), 2.88-2.91 (m, 2H, C*H*$_2$), 2.69-2.72 (m, 2H, C*H*$_2$), 1.70-1.77 (p, 4H, *J* = 3.13 Hz, C*H*$_2$).

^{13}C{^{1}H}-NMR (101 MHz, CD_2Cl_2, 298 K) δ = 152.14 (*C*$_{Ar}$), 141.54 (*C*$_{Ar}$), 135.99 (*C*$_{Ar}$), 132.96 (*C*H$_{Ar}$), 118.49 (*C*H$_{Ar}$), 71.07 (*C*H$_2$), 29.76 (*C*H$_2$), 28.69 (*C*H$_2$), 23.64 (*C*H$_2$), 23.46 (*C*H$_2$).

^{11}B{^{1}H}-NMR (128 MHz, CD_2Cl_2, 298 K) δ = 32.74.

HRMS(ESI$^+$) calcd. for $[C_{11}H_{12}O_2B]^-$: 187.09269; found 187.09370.

Isolated Yield: 82% (46.3 mg).

3-methylhexahydrobenzo[*c*][1,2]oxaborol-1(3*H*)-ol (6k)

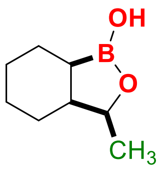

Prepared by following the general experimental procedure with: [Rh(CAAC)(COD)Cl] **1** (1.71 mg, 1 mol%), 3-methylbenzo[*c*][1,2]oxaborol-1(3*H*)-ol (44.4 mg, 0.3 mmol), H_2 (40 bar), 4 Å molecular sieves (100 mg), DCM (1 mL), 40 °C, 24 h.

Chemical formula: $C_8H_{15}BO_2$

Molecular weight: 154.02

^{1}**H NMR** (400 MHz, CD_2Cl_2, 298 K) δ = 5.82 (br s, 1H, O*H*), 4.04-4.10 (qd, 1H, *J* = 6.54, 4.15, C*H*), 1.88-1.95 (m, 1H, C*H*), 1.71-1.75 (m, 1H, C*H*), 1.36-1.63 (m, 5H, CH_2 & C*H*), 1.09 (d, 3H, *J* = 6.59 Hz, CH_3), 0.78-1.04 (m, 3H, CH_2 & C*H*).

^{13}**C{^{1}H}-NMR** (101 MHz, CD_2Cl_2, 298 K) δ = 77.86, 42.72, 26.13, 25.16, 24.51, 24.04, 16.61.

^{11}**B{^{1}H}-NMR** (128 MHz, CD_2Cl_2, 298 K): 35.55.

Diastereomeric ratio (d.r.): 83:17.

HRMS(ESI$^-$) calcd. for $[C_8H_{14}BO_2]^-$: 153.10939; found 153.10905.

Isolated Yield: 91% (42.1 mg).

3-ethylhexahydrobenzo[*c*][1,2]oxaborol-1(3*H*)-ol (6l)

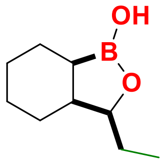

Prepared by following the general experimental procedure with: [Rh(CAAC)(COD)Cl] **1** (1.71 mg, 1 mol%), 3-ethylbenzo[*c*][1,2]oxaborol-1(3*H*)-ol (48.6 mg, 0.3 mmol), H_2 (40 bar), 4 Å molecular sieves (100 mg), DCM (1 mL), 40 °C, 24 h.

Chemical formula: $C_9H_{17}BO_2$

Molecular weight: 168.04

^{1}**H NMR** (400 MHz, CD_2Cl_2, 298 K) (mixture of diastereoisomers) δ = 5.72 (br s, 1H, O*H*), 3.83-3.88 (td, 0.79H, C*H*), 3.77-3.81 (ddd, 0.21H, C*H*), 1.88-2.10 (m, 1H, C*H*), 1.79-1.84 (m, 1H, C*H*), 1.58-1.70 (m, 4H, CH_2), 1.42-1.54 (m, 3H, CH_2 & C*H*), 1.21-1.32 (m, 1H, C*H*), 0.98-1.11 (m, 2H, CH_2), 0.86-0.96 (m, 3H, CH_3).

^{13}**C{^{1}H}-NMR** (101 MHz, CD_2Cl_2, 298 K) (mixture of diastereoisomers) δ = 85.10 (minor), 83.87 (major), 42.26 (minor), 41.48 (major), 29.24 (minor), 27.93 (minor), 26.17 (major), 25.22 (major), 24.44 (minor), 24.32 (major), 24.11 (major), 23.52 (minor), 10.73 (minor), 10.66 (major).

^{11}B{^{1}H}-NMR (128 MHz, CD_2Cl_2, 298 K) δ = 35.48.

Diastereomeric ratio (d.r.): 83:17.

HRMS(ESI$^-$) calcd. for $[C_9H_{17}O_2BNa]^-$: 191.12155; found 191.12105.

Isolated Yield: 88% (44.4 mg).

3-propylhexahydrobenzo[*c*][1,2]oxaborol-1(3*H*)-ol (6m)

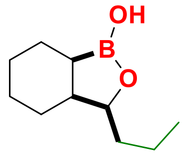

Prepared by following the general experimental procedure with: [Rh(CAAC)(COD)Cl] **1** (1.71 mg, 1 mol%), 3-propylbenzo[*c*][1,2]oxaborol-1(3*H*)-ol (52.8 mg, 0.3 mmol), H_2 (40 bar), 4 Å molecular sieves (100 mg), DCM (1 mL), 40 °C, 24 h.

Chemical formula: $C_{10}H_{19}BO_2$

Molecular weight: 182.07

^{1}H NMR (400 MHz, CD_2Cl_2, 298 K) (mixture of diastereoisomers) δ = 5.54 (br s, 1H, O*H*), 3.84-3.88 (ddd, 0.69H, *J* = 7.69, 5.73, 3.95 Hz, C*H*), 3.78-3.82 (td, 0.31H, *J* = 6.27, 3.84 Hz, C*H*), 1.92-2.01 (m, 1H, C*H*), 1.71-1.83 (m, 1H, C*H*), 1.48-1.63 (m, 3H, CH_2 & C*H*), 1.30-1.46 (m, 5H, CH_2 & C*H*), 1.15-1.28 (m, 2H, CH_2), 0.91-1.03 (m, 2H, CH_2), 0.86 (t, 3H, *J* = 6.79 Hz, CH_3).

^{13}C{^{1}H}-NMR (101 MHz, CD_2Cl_2, 298 K) (mixture of diastereoisomers) δ = 83.38 (minor), 82.09 (major), 42.76 (minor), 41.85 (major), 37.35 (minor), 33.56 (major), 29.19 (minor), 26.19 (major), 25.27 (major), 24.50 (major), 24.45 (minor), 24.13 (major), 23.54 (minor), 19.87 (minor), 19.81 (major), 14.38 (major), 14.27 (minor).

^{11}B{^{1}H}-NMR (128 MHz, CD_2Cl_2, 298 K) δ = 35.46.

Diastereomeric ratio (d.r.): 83:17.

HRMS(ESI$^-$) calcd. for $[C_{10}H_{18}O_2B]^-$:181.14072; found 181.14069.

Isolated Yield: 91% (49.7 mg).

3-cyclohexylhexahydrobenzo[*c*][1,2]oxaborol-1(3*H*)-ol (6n)

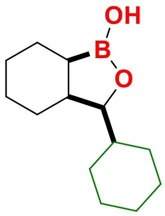

Prepared by following the general experimental procedure with: [Rh(CAAC)(COD)Cl] **1** (1.71 mg, 1 mol%), 3-phenylbenzo[*c*][1,2]oxaborol-1(3*H*)-ol (63.0 mg, 0.3 mmol), H_2 (40 bar), 4 Å molecular sieves (100 mg), DCM (1 mL), 40 °C, 24 h.

Chemical formula: $C_{13}H_{23}BO_2$

Molecular weight: 222.14

^{1}**H NMR** (400 MHz, CD_2Cl_2, 298 K) (mixture of diastereoisomers) δ = 4.72 (br s, 1H, O*H*), 3.40-3.43 (dd, 1H, *J* = 10.43, 3.75 Hz, C*H*), 2.00-2.06 (m, 1H, C*H*), 1.89-1.96 (m, 1H, C*H*), 1.70-1.74 (m, 1H, C*H*), 1.50-1.67 (m, 7H, CH_2 & C*H*), 1.34-1.47 (m, 3H, CH_2 & C*H*), 1.05-1.22 (m, 3H, CH_2 & C*H*), 0.72-1.03 (m, 5H, CH_2 & C*H*).

^{13}**C{^{1}H}-NMR** (101 MHz, CD_2Cl_2, 298 K) (mixture of diastereoisomers) δ = 88.66 (minor), 86.57 (major), 41.35 (minor), 40.78 (major), 39.60 (minor), 38.47 (major), 31.14 (major), 30.71 (minor), 30.22 (minor), 29.34 (minor), 28.90 (major), 27.02 (major), 26.94 (minor), 26.56 (minor), 26.42 (minor), 26.24 (major), 26.13 (major), 25.31 (major), 24.84 (minor), 24.29 (major), 24.21 (major), 23.63 (minor).

^{11}**B{^{1}H}-NMR** (128 MHz, CD_2Cl_2, 298 K) δ = 35.55.

Diastereomeric ratio (d.r.): 83:17.

HRMS(ESI^{+}) calcd. for $[C_{13}H_{22}O_2B]^{-}$: 221.17208; found 221.17185.

Isolated Yield: 96% (64.0 mg).

3-isopropylhexahydrobenzo[*c*][1,2]oxaborol-1(3*H*)-ol (6o)

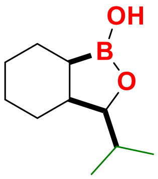

Prepared by following the general experimental procedure with: [Rh(CAAC)(COD)Cl] **1** (1.71 mg, 1 mol%), 3-isopropylbenzo[*c*][1,2]oxaborol-1(3*H*)-ol (52.8 mg, 0.3 mmol), H_2 (40 bar), 4 Å molecular sieves (100 mg), DCM (1 mL), 40 °C, 24 h.

Chemical formula: $C_{10}H_{19}BO_2$

Molecular weight: 182.07

^{1}**H NMR** (400 MHz, CD_2Cl_2, 298 K) (mixture of diastereoisomers) δ = 5.31 (br s, 1H, O*H*), 3.40-3.42 (d, 0.28H, *J* = 7.56, 2.74 Hz, C*H*), 3.33-3.36 (dd, 0.67H, *J* = 10.47, 3.73 Hz, C*H*), 1.99-2.07 (m, 1H, C*H*), 1.52-1.75 (m, 5H, CH_2 & C*H*), 1.37-1.47 (m, 2H, CH_2), 1.11-1.18 (m, 1H, C*H*), 0.97-1.03 (m, 2H, CH_2), 0.92 (d, 2H, *J* = 6.32 Hz, CH_3), 0.82 (d, 2H, *J* = 6.67 Hz, CH_3), 0.75 (d, 2H, *J* = 6.67 Hz, CH_3).

^{13}**C{^{1}H}-NMR** (101 MHz, CD_2Cl_2, 298 K) (mixture of diastereoisomers) δ = 89.64 (minor), 88.32 (major), 41.08 (major), 39.91 (minor), 31.55 (minor), 30.63 (minor), 29.00 (major), 26.21 (major), 26.08 (minor), 25.32 (major), 24.79 (minor), 24.20 (major), 24.17 (major), 23.59 (minor), 20.70 (major), 19.60 (minor), 18.72 (minor), 18.39 (major).

^{11}B{^{1}H}-NMR (128 MHz, CD_2Cl_2, 298 K) δ = 35.59.

Diastereomeric ratio (d.r.): 72:28.

HRMS(ESI^{-}) calcd. for $[C_{10}H_{18}O_2B]^{-}$: 181.14072; found 181.14056.

Isolated Yield: 90% (49.2 mg).

3-((trimethylsilyl)methyl)hexahydrobenzo[*c*][1,2]oxaborol-1(3*H*)-ol (6p)

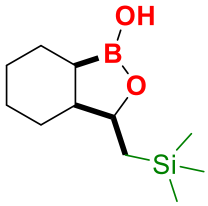

Prepared by following the general experimental procedure with: [Rh(CAAC)(COD)Cl] **1** (1.71 mg, 1 mol%), 3-((trimethylsilyl)methyl)benzo[*c*][1,2]oxaborol-1(3*H*)-ol (66.0 mg, 0.3 mmol), H_2 (40 bar), 4 Å molecular sieves (100 mg), DCM (1 mL), 40 °C, 24 h.

Chemical formula: $C_{11}H_{23}BO_2Si$

Molecular weight: 226.20

^{1}H NMR (400 MHz, CD_2Cl_2, 298 K) (mixture of diastereoisomers) δ = 5.51 (br s, 1H, O*H*), 4.07-4.12 (ddd, 0.77H, *J* = 9.85, 6.48, 3.76 Hz, C*H*), 3.96-4.01 (td, 0.20H, *J* = 7.41, 2.65 Hz, C*H*), 1.89-1.97 (m, 1H, C*H*), 1.75-1.78 (m, 1H, C*H*), 1.37-1.66 (m, 5H, CH_2 & C*H*), 1.10-1.22 (m, 1H, C*H*), 0.80-1.07 (m, 4H, CH_2), 0.00 (s, 9H, CH_3).

^{13}C{^{1}H}-NMR (101 MHz, CD_2Cl_2, 298 K) (mixture of diastereoisomers) δ = 81.98 (minor), 80.30 (major), 45.57 (minor), 43.40 (major), 29.74 (minor), 26.21 (major), 25.29 (major), 24.78 (minor), 24.73 (major), 24.22 (major), 23.64 (minor), 19.57 (major), -0.76 (minor), -0.80 (major).

^{11}B{^{1}H}-NMR (128 MHz, CD_2Cl_2, 298 K) δ = 35.48.

Diastereomeric ratio (d.r.): 83:17.

HRMS(ESI^{+}) calcd. for $[C_{11}H_{22}O_2BSi]^{-}$: 225.14900; found 225.11067.

Isolated Yield: 91% (61.8 mg).

3-ethyl-5-methoxyhexahydrobenzo[*c*][1,2]oxaborol-1(3*H*)-ol (6q)

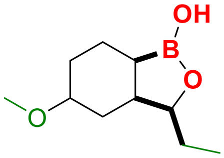

Prepared by following the general experimental procedure with: [Rh(CAAC)(COD)Cl] **1** (1.71 mg, 1 mol%), 3-ethyl-5-methoxybenzo[*c*][1,2]oxaborol-1(3*H*)-ol (57.6 mg, 0.3 mmol), H_2 (40 bar), 4 Å molecular sieves (100 mg), DCM (1 mL), 40 °C, 24 h.

Chemical formula: $C_{10}H_{19}BO_3$

Molecular weight: 198.07

^{1}H NMR (400 MHz, CD_2Cl_2, 298 K) (mixture of diastereoisomers) δ = 3.90-3.94 (ddd, 1H, *J* = 7.91, 6.27, 4.02 Hz, C*H*), 3.35 (s, 3H, OCH_3), 3.03-3.11 (tt, 1H, *J* = 11.22, 3.60 Hz, C*H*), 2.19-2.26 (m, 1H, C*H*), 1.89-2.01 (m, 3H, CH_2 & C*H*), 1.60-1.67 (m, 1H, C*H*), 1.48-1.55 (m, 2H, CH_2), 1.41-1.44 (m, 1H, C*H*), 1.01 (t, 3H, *J* = 7.43 Hz, CH_3), 0.78-0.94 (m, 2H, CH_2).

^{13}C{^{1}H}-NMR (101 MHz, CD_2Cl_2, 298 K) (mixture of diastereoisomers) δ = 89.47 (minor), 88.72 (major), 85.41 (major), 82.13 (minor), 60.65, 47.56 (minor), 47.13 (major), 37.67 (minor), 37.02 (major), 35.92 (major), 33.74 (minor), 30.09 (major), 27.52 (major), 25.43 (minor), 15.72.

^{11}B{^{1}H}-NMR (128 MHz, CD_2Cl_2, 298 K) δ = 34.46.

Diastereomeric ratio (d.r.): 90:10.

HRMS(ESI$^+$) calcd. for $[C_{10}H_{18}O_3B]^-$: 197.13454; found 197.13552.

Isolated Yield: 79% (47.0 mg).

5-methoxy-3-propylhexahydrobenzo[*c*][1,2]oxaborol-1(3*H*)-ol (6r)

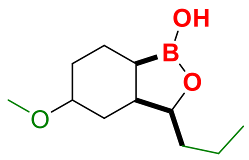

Prepared by following the general experimental procedure with: [Rh(CAAC)(COD)Cl] **1** (1.71 mg, 1 mol%), 5-methoxy-3-propylbenzo[*c*][1,2]oxaborol-1(3*H*)-ol (57.6 mg, 0.3 mmol), H_2 (40 bar), 4 Å molecular sieves (100 mg), DCM (1 mL), 40 °C, 24 h.

Chemical formula: $C_{11}H_{21}BO_3$

Molecular weight: 212.10

^{1}H NMR (400 MHz, CD_3OD, 298 K) (mixture of diastereoisomers) δ = 3.99-4.04 (ddd, 0.80H, *J* = 8.04, 5.18, 4.03 Hz, C*H*), 3.89-3.96 (m, 0.20H, C*H*), 3.35 (s, 3H, OCH_3), 3.03-3.11 (tt, 1H, *J* = 11.26, 3.63 Hz, C*H*), 2.16-2.23 (m, 1H, C*H*), 2.09-2.12 (m, 0.18H, C*H*), 1.89-2.01 (m, 2H, CH_2), 1.37-1.63 (m, 7H, CH_2 & C*H*), 0.99 (t, 3H, *J* = 7.06 Hz, CH_3), 0.79-0.94 (m, 2H, *C*H_2).

^{13}C{^{1}H}-NMR (101 MHz, CD_3OD, 298 K) (mixture of diastereoisomers) δ = 82.99 (minor), 82.10 (major), 80.59 (major), 77.30 (minor), 55.81, 43.27 (minor), 42.67 (major), 38.45 (minor), 34.58 (major), 32.78 (minor), 32.17 (major), 31.25 (major), 31.11 (minor), 22.68 (major), 20.61 (major), 20.53 (minor), 14.50 (major), 14.44 (minor).

^{11}B{^{1}H}-NMR (128 MHz, CD_3OD, 298 K) δ = 34.62.

Diastereomeric ratio (d.r.): 92:8.

HRMS(ESI$^+$) calcd. for $[C_{11}H_{21}O_3BNa]^+$: 235.14780; found 235.15370.

Isolated Yield: 82% (52.2 mg).

4-fluorohexahydrobenzo[*c*][1,2]oxaborol-1(3*H*)-ol (6t)

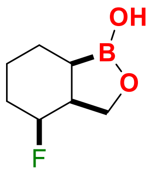

Prepared by following the general experimental procedure with: [Rh(CAAC)(COD)Cl] **1** (1.71 mg, 1 mol%), 4-fluorobenzo[*c*][1,2]oxaborol-1(3*H*)-ol (45.6 mg, 0.3 mmol), H_2 (40 bar), 4 Å molecular sieves (100 mg), hexane (1 mL), 40 °C, 16 h.

Chemical formula: $C_7H_{12}BFO_2$

Molecular weight: 157.98

^{1}H NMR (400 MHz, CD_2Cl_2, 298 K) (mixture of diastereoisomers) δ = 5.13 (br s, 1H, O*H*), 4.72-4.88 (m, 1H, C*H*), 3.98-4.12 (m, 2H, C*H*$_2$), 2.46-2.57 (m, 1H, C*H*), 1.63-1.84 (m, 3H, C*H*$_2$ & C*H*), 1.46-1.61 (m, 2H, C*H*$_2$), 1.29-1.44 (m, 2H, C*H*$_2$).

^{13}C{^{1}H}-NMR (101 MHz, CD_2Cl_2, 298 K) δ = 92.58 (d, *J* = 169.92 Hz), 68.46 (d, *J* = 5.59 Hz), 42.13 (d, *J* = 17.38 Hz), 29.30 (d, *J* = 19.63 Hz), 21.90, 21.48 (d, *J* = 6.72 Hz).

^{11}B{^{1}H}-NMR (128 MHz, CD_2Cl_2, 298 K) (mixture of diastereoisomers) δ = 35.19.

^{19}F{^{1}H}-NMR (376 MHz, CD_2Cl_2, 298 K) (mixture of diastereoisomers) δ = –173.79 (minor), –186.47 (minor), –188.52 (major).

Diastereomeric ratio (d.r.): 85:9:6.

HRMS(ESI$^+$) calcd. for $[C_7H_{12}O_2BFNa]^+$: 181.08079; found 181.08065.

Isolated Yield: 54% (25.6 mg).

6-fluorohexahydrobenzo[*c*][1,2]oxaborol-1(3*H*)-ol (6u)

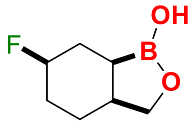

Prepared by following the general experimental procedure with: [Rh(CAAC)(COD)Cl] **1** (1.71 mg, 1 mol%), 6-fluorobenzo[*c*][1,2]oxaborol-1(3*H*)-ol (45.6 mg, 0.3 mmol), H_2 (40 bar), 4 Å molecular sieves (100 mg), hexane (1 mL), 40 °C, 24 h.

Chemical formula: $C_7H_{12}BFO_2$

Molecular weight: 157.98

^{1}H NMR (400 MHz, CD_2Cl_2, 298 K) δ = 4.96 (br s, 1H, O*H*), 4.66-4.82 (m, 1H, C*H*), 3.92-3.96 (dd, 1H, *J* = 9.01, 5.39 Hz, C*H*), 3.72-3.75 (dd, 1H, *J* =

9.01, 3.00 Hz, C*H*), 2.18-2.25 (m, 1H, C*H*), 1.92-2.01 (m, 1H, C*H*), 1.72-1.90 (m, 2H, CH_2), 1.45-1.69 (m, 3H, CH_2 & C*H*), 1.36-1.40 (m, 1H, C*H*).

^{13}C{^{1}H}-NMR (101 MHz, CD_2Cl_2, 298 K) δ = 89.77 (d, *J* = 167.97 Hz), 72.14, 37.38, 29.12 (d, *J* = 20.30 Hz), 28.40 (d, *J* = 20.40 Hz), 22.65 (d, *J* = 4.98 Hz).

^{11}B{^{1}H}-NMR (128 MHz, CD_2Cl_2, 298 K) δ = 35.65.

^{19}F{^{1}H}-NMR (376 MHz, CD_2Cl_2, 298 K) δ = –181.60.

Diastereomeric ratio (d.r.): 84:16.

HRMS(ESI$^+$) calcd. for $[C_7H_{12}O_2BFNa]^+$: 181.08079; found 181.08048.

Isolated Yield: 56% (26.6 mg).

5,6-difluorohexahydrobenzo[*c*][1,2]oxaborol-1(3*H*)-ol (6v)

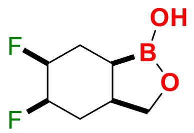

Prepared by following the general experimental procedure with: [Rh(CAAC)(COD)Cl] **1** (1.71 mg, 1 mol%), 5,6-difluoro-1,3-dihydrobenzo[*c*][1,2]oxaborole (51.0 mg, 0.3 mmol), H_2 (40 bar), 4 Å molecular sieves (100 mg), hexane (1 mL), 40 °C, 24 h.

Chemical formula: $C_7H_{11}BF_2O_2$

Molecular weight: 175.97

^{1}H NMR (400 MHz, $CDCl_3$, 298 K) δ = 5.24 (br s, 1H, O*H*), 4.81-4.98 (m, 1H, C*H*), 4.41-4.63 (m, 1H, C*H*), 3.96-4.04 (m, 1H, C*H*), 3.83-3.86 (m, 1H, C*H*), 2.23-2.41 (m, 2H, CH_2), 1.82-2.06 (m, 2H, CH_2), 1.66-1.78 (m, 1H, C*H*), 1.36-1.42 (m, 1H, C*H*).

^{13}C{^{1}H}-NMR (101 MHz, $CDCl_3$, 298 K) δ = 90.09 (dd, *J* = 180.47, 18.55 Hz), 88.76 (d, *J* = 177.37, 17.36 Hz), 71.69 (d, *J* = 1.48 Hz), 36.86 (d, *J* = 9.10 Hz), 28.19 (dd, *J* = 18.86, 5.21 Hz),26.08 (dd, *J* = 20.34, 6.35 Hz).

^{11}B{^{1}H}-NMR (128 MHz, CD_2Cl_2, 298 K) δ = 35.17.

^{19}F{^{1}H}-NMR (376 MHz, $CDCl_3$, 298 K) δ = –185.20 (d, 15.04 Hz), –185.92 (d, *J* = 15.04 Hz), –200.62, –201.07 (d, *J* = 15.05 Hz).

Diastereomeric ratio (d.r.): 88:12

HRMS(ESI$^+$) calcd. for $[C_7H_{10}O_2BF_2]^-$: 175.07487; found 175.07476.

Isolated Yield: 40% (21.1 mg).

5-fluorohexahydrobenzo[*c*][1,2]oxaborol-1(3*H*)-ol (6w)

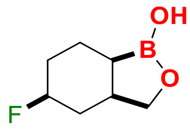

Prepared by following the general experimental procedure with: [Rh(CAAC)(COD)Cl] **1** (1.71 mg, 1 mol%), 5-fluoro-1,3-dihydrobenzo[*c*][1,2]oxaborole (45.6 mg, 0.3 mmol), H_2 (40 bar), 4 Å molecular sieves (100 mg), hexane (1 mL), 40 °C, 24 h.

Chemical formula: $C_7H_{12}BFO_2$

Molecular weight: 157.98

^{1}H NMR (400 MHz, $CDCl_3$, 298 K) δ = 4.84 (br s, 1H, O*H*), 4.37-4.56 (m, 1H, C*H*), 4.00 (ddd, 1H, *J* = 9.08, 5.02, 2.58 Hz, C*H*), 3.79 (dd, 1H, *J* = 8.99, 1.82 Hz, C*H*), 2.32-2.36 (m, 1H, C*H*), 1.95-2.11 (m, 3H, CH_2 & C*H*), 1.58 (m, 2H, CH_2), 1.35-1.48 (m, 2H, CH_2).

^{13}C{^{1}H}-NMR (101 MHz, $CDCl_3$, 298 K) δ = 91.34 (d, *J* = 171.28 Hz), 72.05, 37.98 (d, *J* = 9.38 Hz), 34.95 (d, *J* = 18.38 Hz), 31.73 (d, *J* = 18.17 Hz), 20.38 (d, *J* = 10.65 Hz).

^{11}B{^{1}H}-NMR (128 MHz, CD_2Cl_2, 298 K) δ = 35.73.

^{19}F{^{1}H}-NMR (376 MHz, CD_2Cl_2, 298 K) δ = –169.50 (major), –171.77 (minor).

Diastereomeric ratio (d.r.): 84:16

HRMS(ESI$^+$) calcd. for $[C_7H_{11}OBF]^-$: 157.08430; found 157.08433

Isolated Yield: 60% (28.4 mg).

***N*-(1-hydroxyoctahydrobenzo[*c*][1,2]oxaborol-5-yl)acetamide (6x)**

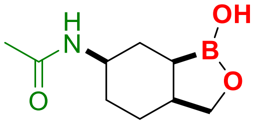

Prepared by following the general experimental procedure with: [Rh(CAAC)(COD)Cl] **1** (1 mol%), *N*-(1-hydroxy-1,3-dihydrobenzo[*c*][1,2]oxaborol-6-yl)acetamide (57.3 mg, 0.3 mmol), H_2 (40 bar), 4 Å molecular sieves (100 mg), DCM (1 mL), 40 °C, 24 h.

Chemical formula: $C_9H_{16}BNO_3$

Molecular weight: 197.04

^{1}H NMR (400 MHz, CD_3OD, 298 K) δ = 7.76 (br s, 1H, N*H*), 3.84-3.87 (dd, 1H, *J* = 9.04, 4.60 Hz, C*H*), 3.59 (d, 1H, *J* = 9.03 Hz, C*H*), 3.32-3.37 (m, 1H, C*H*), 1.96-2.01 (m, 1H, C*H*), 1.85-1.88 (m, 1H, C*H*), 1.80 (s, 3H, CH_3), 1.63-1.75 (m, 2H, CH_2), 1.44-1.47 (m, 1H, C*H*), 1.29-1.36 (m, 1H, C*H*), 1.12-1.19 (m, 1H, C*H*), 0.95-1.04 (m, 1H, C*H*).

^{13}**C{^{1}H}-NMR** (101 MHz, CD_2Cl_2, 298 K) δ = 172.35 (*C*O), 73.41 (*C*H$_2$), 49.85 (*C*H), 38.92 (*C*H), 32.24 (*C*H$_3$), 30.32 (*C*H$_2$), 29.12 (*C*H$_2$), 22.69 (*C*H$_2$).

^{11}**B{^{1}H}-NMR** (128 MHz, CD_2Cl_2, 298 K) δ = 34.56.

Diastereomeric ratio (d.r.): 67:33.

HRMS(ESI^{+}) calcd. for $[C_9H_{15}NO_3B]^-$: 196.11522; found 196.11510.

Isolated Yield: 86% (50.9 mg).

6-(cyclohexyloxy)hexahydrobenzo[*c*][1,2]oxaborol-1(3*H*)-ol (6y)

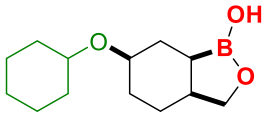

Prepared by following the general experimental procedure with: [Rh(CAAC)(COD)Cl] **1** (1.71 mg, 1 mol%), 6-phenoxybenzo[*c*][1,2]oxaborol-1(3*H*)-ol (67.8 mg, 0.3 mmol), H_2 (40 bar), 4 Å molecular sieves (100 mg), DCM (1 mL), 40 °C, 24 h.

Chemical formula: $C_{13}H_{23}BO_3$

Molecular weight: 238.13

^{1}**H NMR** (400 MHz, CD_3OD, 298 K) (mixture of diastereoisomers) δ = 3.88-3.96 (ddd, 1H, *J* = 18.00, 8.97, 5.53 Hz, C*H*), 3.76-3.80 (dd, 0.66H, *J* = 8.99, 5.86 Hz, C*H*), 3.69 (d, 0.34 H, C*H*), 3.51-3.56 (tt, 1H, C*H*), 3.37-3.44 (dp, 1H, *J* = 9.06, 4.36 Hz, C*H*), 2.01-2.23 (m, 1H, C*H*), 1.81-1.88 (m, 3H, C*H*$_2$ & C*H*), 1.72-1.78 (m, 2H, C*H*$_2$), 1.65-1.70 (m, 1H, C*H*), 1.50-1.61 (m, 4H, C*H*$_2$), 1.09-1.45 (m, 7H, C*H*$_2$ & C*H*).

^{13}**C{^{1}H}-NMR** (101 MHz, CD_2Cl_2, 298 K) (mixture of diastereoisomers) δ = 76.48 (major), 75.93 (major), 75.32 (major), 73.32 (minor), 72.88 (minor), 49.85 (major), 39.10 (minor), 38.36 (major), 34.38 (minor), 34.31 (minor), 33.89 (major), 33.83 (major), 32.60 (major), 31.30 (minor), 29.88 (major), 29.09 (minor), 28.64 (minor), 27.03 (major), 26.89 (minor), 25.42 (minor), 25.19 (major), 25.17 (major), 24.30 (minor).

^{11}**B{^{1}H}-NMR** (128 MHz, CD_3OD, 298 K) δ = 34.85.

Diastereomeric ratio (d.r.): 80:20.

HRMS(ESI^{+}) calcd. for $[C_{13}H_{22}O_3B]^-$: 237.16699; found 237.16675.

Isolated Yield: 85% (60.7 mg).

21.General procedure for the hydrogenation of benzoxaborinin derivatives

[Rh(CAAC)(COD)Cl] **1** (1.71 mg, 1 mol%), benzoxaborinin derivatives (0.3 mmol), and 4 Å molecular sieves (100 mg) were filled to an oven-dried 4 mL screw-cap vial equipped with a stirring bar.

Dichloromethane (DCM) (1 mL) was added under argon atmosphere. The glass vial was placed in a 150 mL stainless steel autoclave under argon atmosphere. The autoclave was sealed, pressurized and depressurized with hydrogen gas three times before the 40-bar pressure was set. The reaction mixture was stirred at the 40 °C temperature for 24 h. After the autoclave was carefully depressurized, 1,2,4,5-tetramethylbenzene as an internal standard was added and the mixture was stirred vigorously for 5 min. After filtration over Whatman® filter, conversion and diastereomeric ratio (d.r.) were determined by NMR analysis. The crude reaction mixture was applied to silica chromatography eluting with EtOAc/pentane (50:50 to 100:0) to give desired functionalized hexahydrobenzoxaborinin product. These compounds were found to be sensitive for GC measurements, therefore, the diastereomeric ratio (d.r.) of the compounds were calculated using NMR.

Octahydro-1*H*-benzo[*c*][1,2]oxaborinin-1-ol (8a)

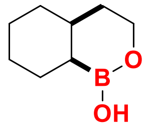

Prepared by following the general experimental procedure with: [Rh(CAAC)(COD)Cl] **1** (1.71 mg, 1 mol%), 3,4-dihydro-1*H*-benzo[*c*][1,2]oxaborinin-1-ol (44.4 mg, 0.3 mmol), H_2 (40 bar), 4 Å molecular sieves (100 mg), THF (1 mL), 40 °C, and 24 h.

Chemical formula: $C_8H_{15}BO_2$

Molecular weight: 154.02

^{1}H NMR (400 MHz, CD_3OD, 298 K) δ = 4.10-4.16 (m, 1H, C*H*), 3.96-4.01 (m, 1H, C*H*), 1.75-1.84 (m, 2H, C*H*$_2$), 1.67-1.73 (m, 2H, C*H*$_2$), 1.56-1.65 (m, 2H, C*H*$_2$), 1.42-1.49 (m, 1H, C*H*), 1.27-1.37 (m, 3H, C*H*$_2$ & C*H*), 1.14-1.25 (m, 2H, C*H*$_2$).

^{13}C{^{1}H}-NMR (101 MHz, CD_2Cl_2, 298 K) δ = 64.07 (*C*H$_2$), 34.55 (*C*H$_2$), 32.89 (*C*H$_2$), 31.25 (*C*H), 27.13 (*C*H$_2$), 26.72 (*C*H$_2$), 26.08 (*C*H$_2$).

^{11}B{^{1}H}-NMR (128 MHz, CD_3OD, 298 K) δ = 30.81.

Diastereomeric ratio (d.r.): 89:11.

HRMS(ESI$^+$) calcd. for $[C_8H_{14}O_2B]^-$: 153.10829; found 153.09229.

Isolated Yield: 90% (41.6 mg).

7-(trifluoromethyl)octahydro-1*H*-benzo[*c*][1,2]oxaborinin-1-ol (8b)

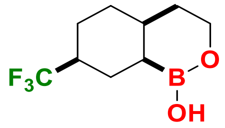

Prepared by following the general experimental procedure with: [Rh(CAAC)(COD)Cl] **1** (1.71 mg, 1 mol%), 7-(trifluoromethyl)-3,4-dihydro-1*H*-benzo[*c*][1,2]oxaborinin-1-ol (66.60 mg, 0.3 mmol), H_2 (40 bar), 4 Å molecular sieves (100 mg), DCM (1 mL), 40 °C, and 24 h.

Chemical formula: $C_9H_{14}BF_3O_2$

Molecular weight: 222.01

^{1}H NMR (400 MHz, $CDCl_3$, 298 K) (mixture of diastereoisomers) δ = 4.66 (br s, 1H, OH_{minor}), 4.59 (br s, 1H, OH_{major}), 3.93-4.17 (m, 3H, CH_2 & CH), 1.82-2.04 (m, 4H, CH_2), 1.70-1.77 (m, 1H, CH), 1.59-1.64 (m, 1H, CH), 1.21-1.45 (m, 4H, CH_2).

^{13}C{^{1}H}-NMR (101 MHz, $CDCl_3$, 298 K) (mixture of diastereoisomers) δ = 123.71-132.02 (m, major & minor), 66.31 (major), 61.98 (minor), 42.56 (q, 26.18 hz, major), 40.84 (q, 26.28 Hz, minor), 32.45 (major), 32.37 (minor), 31.19 (minor), 30.05 (major), 27.92 (major), 27.89 (minor), 25.18 (m, minor), 24.78 (m, major), 21.64 (m, minor), 20.23 (m, major).

^{11}B{^{1}H}-NMR (128 MHz, $CDCl_3$, 298 K) δ = 31.12.

^{19}F{^{1}H}-NMR (376 MHz, CD_2Cl_2, 298 K) δ (mixture of diastereoisomers) δ = -73.98 (minor), -73.57 (major).

Diastereomeric ratio (d.r.): 67:33.

HRMS(ESI$^-$) calcd. for $[C_9H_{13}BF_3O_2]^-$: 221.09552; found 221.09134.

Isolated Yield: 88% (58.6 mg).

7-methoxyoctahydro-1*H*-benzo[*c*][1,2]oxaborinin-1-ol (8c)

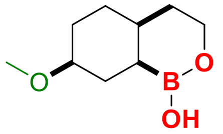

Prepared by following the general experimental procedure with: [Rh(CAAC)(COD)Cl] **1** (1.71 mg, 1 mol%), 7-methoxy-3,4-dihydro-1*H*-benzo[*c*][1,2]oxaborinin-1-ol (53.1 mg, 0.3 mmol), H_2 (40 bar), 4 Å molecular sieves (100 mg), DCM (1 mL), 40 °C, and 24 h.

Chemical formula: $C_9H_{17}BO_3$

Molecular weight: 184.04

^{1}H NMR (400 MHz, CD_2Cl_2, 298 K) (mixture of diastereoisomers) δ = 3.99-4.05 (m ,1H, CH), 3.80-3.86 (m, 1H, CH), 3.20 (s, 3H, OCH_3), 3.11-3.17 (m, 1H, CH), 1.65-1.77 (m, 2H, CH_2), 1.60-1.63 (m, 1H, CH), 1.42-1.57 (m, 4H, CH_2), 1.31-1.40 (m, 1H, CH), 1.17-1.25 (m, 1H, CH), 1.02-1.12 (m, 1H, CH).

^{13}C{^{1}H}-NMR (101 MHz, CD_2Cl_2, 298 K) (mixture of diastereoisomers) δ = 78.31 (minor), 77.81 (major), 64.29 (major), 62.66 (minor), 55.78 (major), 55.63 (minor), 32.24 (major), 31.88 (minor), 30.94 (minor), 30.79 (major), 29.32 (major), 28.70 (major), 27.34 (minor), 27.05 (major).

^{11}B{^{1}H}-NMR (128 MHz, CD_3OD, 298 K) δ = 31.67.

Diastereomeric ratio (d.r.): 80:20.

HRMS(ESI⁻) calcd. for $[C_9H_{16}O_3B]^-$: 183.11887; found 183.11699.

Isolated Yield: 73% (40.3 mg).

22.General procedure for the hydrogenation of benzoxaboripin

[Rh(CAAC)(COD)Cl] **1** (1.71 mg, 1 mol%), benzoxaboripin (0.3 mmol), and 4 Å molecular sieves (100 mg) were filled to an oven-dried 4 mL screw-cap vial equipped with a stirring bar. Dichloromethane (DCM) (1 mL) was added under argon atmosphere. The glass vial was placed in a 150 mL stainless steel autoclave under argon atmosphere. The autoclave was sealed, pressurized and depressurized with hydrogen gas three times before the 40-bar pressure was set. The reaction mixture was stirred at 40 °C for 24 h. Afterwards, the autoclave was carefully depressurized Filtration over Whatman® filter, conversion and diastereomeric ratio (d.r.) were determined by NMR analysis. The crude reaction mixture was applied to silica chromatography eluting with EtOAc/pentane (50:50 to 100:0) to give desired functionalized hexahydrobenzoxaborinin product.

Octahydrobenzo[*c*][1,2]oxaborepin-1(3*H*)-ol (8d)

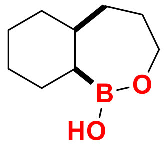

Prepared by following the general experimental procedure with: [Rh(CAAC)(COD)Cl] **1** (1.71 mg, 1 mol%), 4,5-dihydrobenzo[*c*][1,2]oxaborepin-1(3*H*)-ol (48.6 mg, 0.3 mmol), H_2 (40 bar), 4 Å molecular sieves (100 mg), THF (1 mL), 40 °C, and 24 h.

Chemical formula: $C_9H_{17}BO_2$

Molecular weight: 168.04

^{1}H NMR (400 MHz, CD_3OD, 298 K) (mixture of diastereoisomers) δ = 3.99-4.04 (m, 1H, C*H*), 3.81-3.87 (m, 1H, C*H*), 3.49-3.53 (m, 1H, C*H*), 1.81-1.95 (m, 2H, CH_2), 1.53-1.77 (m, 5H, CH_2 & C*H*), 1.40-1.49 (m, 2H, CH_2), 1.15-1.37 (m, 4H, CH_2).

^{13}C{^{1}H}-NMR (101 MHz, CD_2Cl_2, 298 K) (mixture of diastereoisomers) δ = 67.67 (major), 66.92 (minor), 38.65 (major), 37.72 (minor), 36.04 (major), 33.13 (minor), 32.98 (major), 31.71 (minor), 30.08 (minor), 28.58 (major), 27.78 (major), 26.83 (minor), 26.10 (major), 25.91 (major).

^{11}B{^{1}H}-NMR (128 MHz, CD_3OD, 298 K) δ = 31.72.

Diastereomeric ratio (d.r.): 90:10.

HRMS(ESI⁺) calcd. for $[C_9H_{16}O_2B]^-$: 167.12505; found 167.12488.

Isolated Yield: 69% (34.8 mg).

23. Mechanistic Experiments

23.1. Yield/time profile experiments for the hydrogenation of benzoxaborole (5a) using Rh-CAAC (1)

Procedure: Eight individual reactions were performed for the reaction progress experiments and stopped after different time intervals. [Rh(CAAC)(COD)Cl] **1** (1.71 mg, 1 mol%), benzoxaborole (**5a**) (40.22 mg, 0.3 mmol), and 4 Å molecular sieves (100 mg) were filled to an oven-dried 4 mL screw-cap vial equipped with a stirring bar. DCM (1 mL) was added under argon atmosphere. The glass vial was placed in a 150 mL stainless steel autoclave under argon atmosphere. The autoclave was sealed, pressurized, and depressurized with hydrogen gas three times before the 40-bar of hydrogen pressure was set. The reaction mixture was stirred at the 40 °C temperature for certain reaction time. Afterwards, the autoclave was carefully depressurized, 1,2,4,5-tetramethylbenzene as an internal standard was added and the mixture was stirred vigorously for 5 min. After filtration over Whatman® filter, conversion, and yield were determined by NMR analysis.

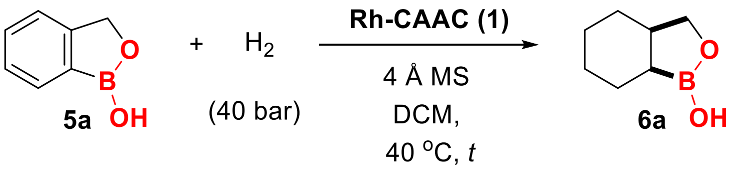

#	Time (minutes)	NMR Yield (%) (6a)
1	0	0
2	5	0
3	15	0
4	30	0
5	60	0
6	120	15
7	180	39
8	300	74
9	480	99

23.2. Yield/time profile experiments for the hydrogenation of benzoxaborole (5a) using preformed [Rh] on 4 Å MS catalyst

Synthesis of rhodium-CAAC derived, molecular sieves supported rhodium catalyst: Rh-CAAC **1** (11.4 mg, 0.02 mmol) was filled to an oven-dried 4 mL screw-cap vial equipped with a stirring bar and 4 Å MS (200 mg, crushed). *n*-Hexane (2 mL) was added under argon atmosphere. The glass vial was placed in a 150 mL stainless steel autoclave under argon atmosphere. The autoclave was pressurized and depressurized with hydrogen gas three times before the pressure was set to 50 bar. The reaction mixture was stirred at 40 °C for 24 h. The pressure was carefully released and the autoclave was directly filled with argon. Under an inert atmosphere, the colorless supernatant was carefully removed and the black solid was washed with *n*-hexane (2 x 5 mL), DCM (3 x 5 mL) and EtOAc (3 x 5 mL). To remove residual EtOAc the black residue was washed two more times with *n*-hexane and dried in vacuo.

Procedure for kinetic study using preformed molecular sieves supported rhodium catalyst: Eight individual reactions were performed for the reaction progress experiments and stopped after different time intervals. Preformed molecular sieves supported Rh(0) catalyst (10.6 mg, 1 mol%), and benzoxaborole (**5a**) (13.40 mg, 0.1 mmol) were filled to an oven-dried 4 mL screw-cap vial equipped with a stirring bar. DCM (0.5 mL) was added under argon atmosphere. The glass vial was placed in a 150 mL stainless steel autoclave under argon atmosphere. The autoclave was sealed, pressurized, and depressurized with hydrogen gas three times before the 40-bar of hydrogen pressure was set. The reaction mixture was stirred at the 40 °C temperature for certain reaction time. Afterwards, the autoclave was carefully depressurized, mesitylene as an internal standard was added and the mixture was stirred vigorously for 5 min. After filtration over Whatman® filter, conversion, and yield were determined by NMR analysis.

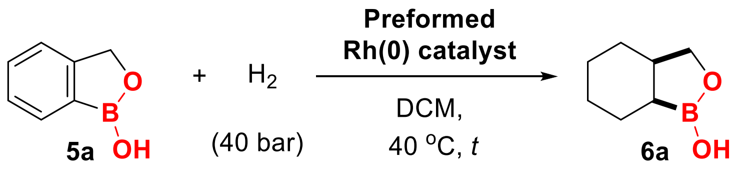

#	Time (minutes)	NMR Yield (%) (6a)
1	0	0
2	5	1
3	15	7
4	30	15
5	60	24
6	120	38

7	180	50
8	300	90
9	480	99

23.3. Filtration tests

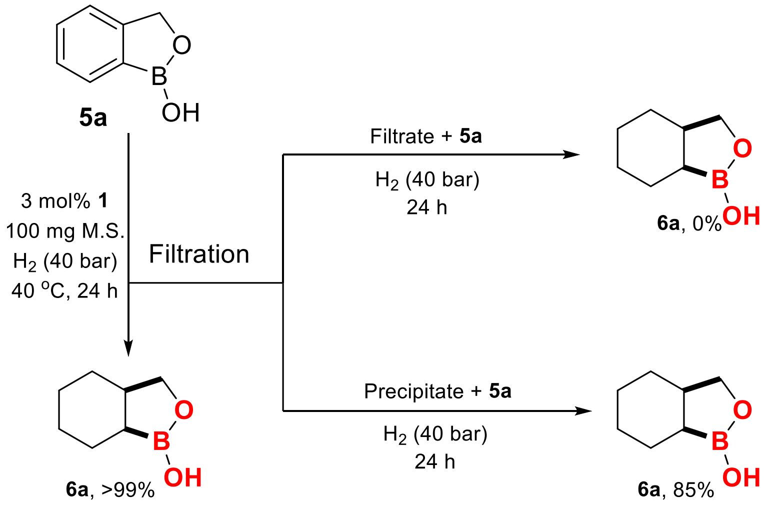

Procedure for the preparation of recovered [Rh] on 4 Å MS: [Rh(CAAC)(COD)Cl] **1** (1.71 mg, 1 mol%), **5a** (40.2 mg, 0.3 mmol), and 4 Å molecular sieves (100 mg) were filled to an oven-dried 4 mL screw-cap vial equipped with a stirring bar. Dichloromethane (DCM) (1 mL) was added under argon atmosphere. The glass vial was placed in a 150 mL stainless steel autoclave under an argon atmosphere. The autoclave was sealed, pressurized, and depressurized with hydrogen gas three times before the 40-bar pressure was set. The reaction mixture was stirred at the 40 °C temperature for 24 h. After the autoclave was carefully depressurized, mesitylene as an internal standard was added and the mixture was stirred vigorously for 5 min and the reaction mixture was filtered over a frit. After filtration, conversion and yield for **6a** were determined by NMR analysis (NMR yield: >99%). The black residue was washed with *n*-pentane (3 x 2 mL), CH_2Cl_2 (3 x 2 mL) and EtOAc (3 x 2 mL), before being dried under reduced pressure. To remove residual EtOAc, the black residue was washed two more times with *n*-pentane and dried in vacuo.

*Hydrogenation of **5a** with recovered [Rh] on 4 Å MS catalyst*

Substrate **5a** (20.10 mg, 0.15 mmol) and Rh catalyst (50 mg, corresponding to 1 mol% catalyst) were filled into an oven-dried 2 mL screw-cap vial equipped with a stirring bar. CH_2Cl_2 (1.0 mL) was added under argon atmosphere and the glass vial was placed in a 150 mL stainless steel autoclave under argon atmosphere. The autoclave was pressurized and depressurized with hydrogen gas three times before the 40 bar pressure was set. The reaction mixture was stirred at the 40 °C temperature for 24 h. After the

autoclave was carefully depressurized, mesitylene as an internal standard was added and the mixture was stirred vigorously for 5 min. After filtration over Whatman® filter, conversion, and yield were determined by NMR analysis (Table S8).

Table S8: Hydrogenation of 5a with different Rh–CAAC catalysts under optimized conditions

#	[Rh] (1 mol%)	Conv. (%)	NMR Yield (%)
**1**	Rh-CAAC (**1**) + 4 Å MS	>99%	>99%
**2**	Recovered Rh-CAAC on 4Å MS	94	85
**3**	Filtrate	0	0
**4**	Preformed Rh-CAAC on 4Å MS	>99%	97

23.4. Poisoning Experiments

Mercury drop test

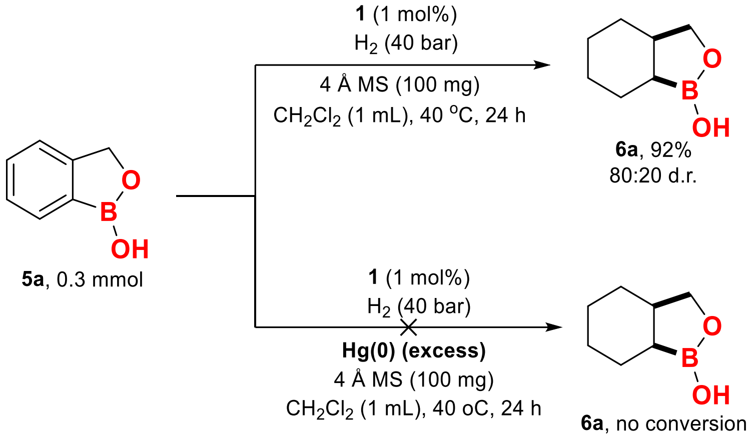

In the absence of Hg(0), under optimized conditions, **5a** was hydrogenated to the corresponding product **6a** with the isolated yield of 92%. In the case of the mercury drop test, the addition of one droplet of elemental mercury under the argon atmosphere into the reaction solution, no conversion was observed. These results indicate that the active species in the catalytic system are heterogeneous.

Controlled fractional poisoning experiments with benzothiophene

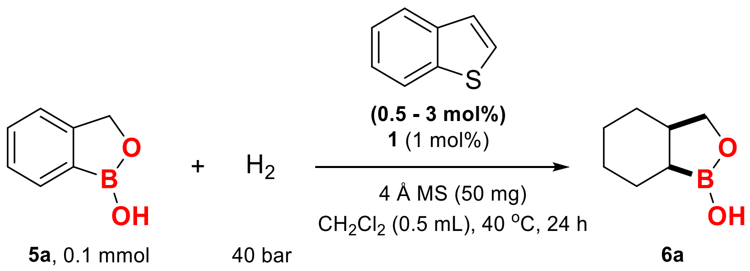

A stock solution of benzothiophene in dry CH_2Cl_2 was prepared. Benzoxaborole **5a** (13.4 mg, 0.1 mmol), **1** (0.6 mg, 1 mol%) and activated 4 Å MS (50 mg, crushed) were filled into an oven-dried 4 mL screw-cap vial equipped with a stirring bar. CH_2Cl_2 was added under argon atmosphere and varying amounts of the benzothiophene stock solution were added to reach a total volume of 0.5 mL. The glass vial was placed in a 150 mL stainless steel autoclave under argon atmosphere. The autoclave was sealed, pressurized, and depressurized with hydrogen gas three times before the indicated pressure was set to 40 bar. The reaction mixture was stirred at 40 °C for 24 h. After the autoclave was carefully depressurized, the yield of the crude mixture was analyzed by NMR. Sub-stochiometric amounts of benzothiophene with respect to the catalyst were sufficient to completely poison the catalyst system leading to no conversion. These results indicate a heterogeneous nature of the active catalyst in the hydrogenation of **5a** (Table S9).[18]

Table S9: Hydrogenation of 5a with complex 1 (1 mol%) in the presence of different concentrations of benzothiophene.

#	Benzothiophene [mol%]	Conversion (%)	Yield 6a [%]
**1**	-	>99%	92
**2**	0.5	0	0
**3**	1	0	0
**4**	3	0	0

1,10-phenanthroline poisoning experiments with preformed molecular sieves supported rhodium catalyst for the hydrogenation of ***5a***

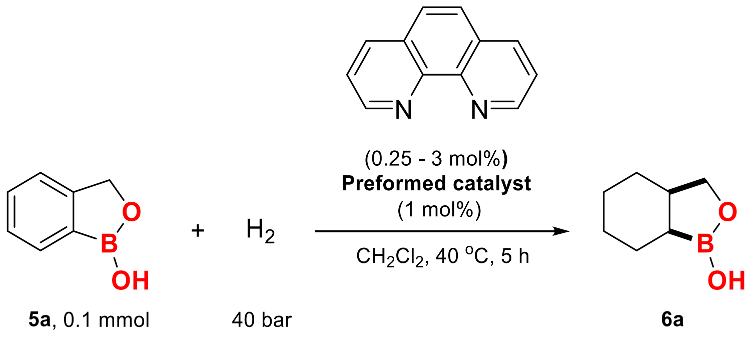

A stock solution of 1,10-phenanthroline in dry CH_2Cl_2 was prepared. Benzoxaborole **5a** (13.4 mg, 0.1 mmol), and preformed Rh(0) catalyst (10.58 mg, 1 mol%) were filled into an oven-dried 4 mL screw-cap vial equipped with a stirring bar. CH_2Cl_2 was added under argon atmosphere and varying amounts of the 1,10-phenanthroline stock solution were added to reach a total volume of 0.5 mL. The glass vial was placed in a 150 mL stainless steel autoclave under argon atmosphere. The autoclave was sealed, pressurized, and depressurized with hydrogen gas three times before the indicated pressure was set to 40 bar. The reaction mixture was stirred at 40 °C for 5 h. After the autoclave was carefully depressurized,

mesitylene as an internal standard was added and the yield of the crude mixture was analyzed by NMR (Table S1). Sub-stochiometric amounts of 1,10-phenanthroline with respect to the catalyst were sufficient to completely poison the catalyst leading to no conversion.

Table S10: Hydrogenation of 5a with complex 1 (1 mol%) in the presence of different concentrations of 1,10-phenanthroline.

#	1,10-phenanthroline [mol%]	Conversion (%)	NMR Yield 6a [%]
**1**	-	92%	90
**2**	0.25	0	0
**3**	0.5	0	0
**4**	1	0	0
**5**	2	0	0

23.5. Analysis of molecular sieves supported rhodium(0) nanoparticles derived from Rh-CAAC (1) and 4Å MS using TEM analysis

Procedure for the preparation of TEM sample: After catalysis, the reaction mixture showed the formation of colorless solution and dark black precipitate. The reaction mixture was centrifuged and the colorless supernatant from the reaction mixture was removed. The black dark residue was washed with DCM two times, and the sample was prepared by direct drop casting, 5 µL of NPs dispersed in DCM onto a copper grid. The sample was measured on a carbon coated copper grid. For the determination of NP size distributions, 300 NPs were measured by hand using ImageJ.

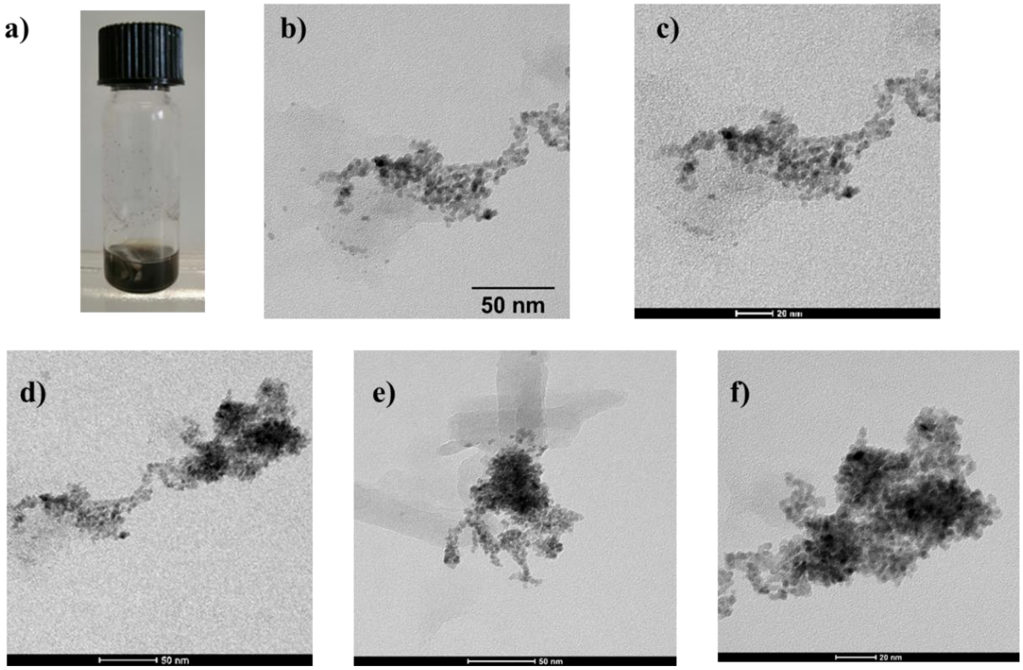

Figure S1: Characterization of the dark black residue after catalysis. a) picture of the reaction vial after catalysis showing the formation of black dark precipitate. b-f) TEM images of the isolated rhodium(0) nanoparticles after catalysis.

23.6. Infrared spectroscopy measurements

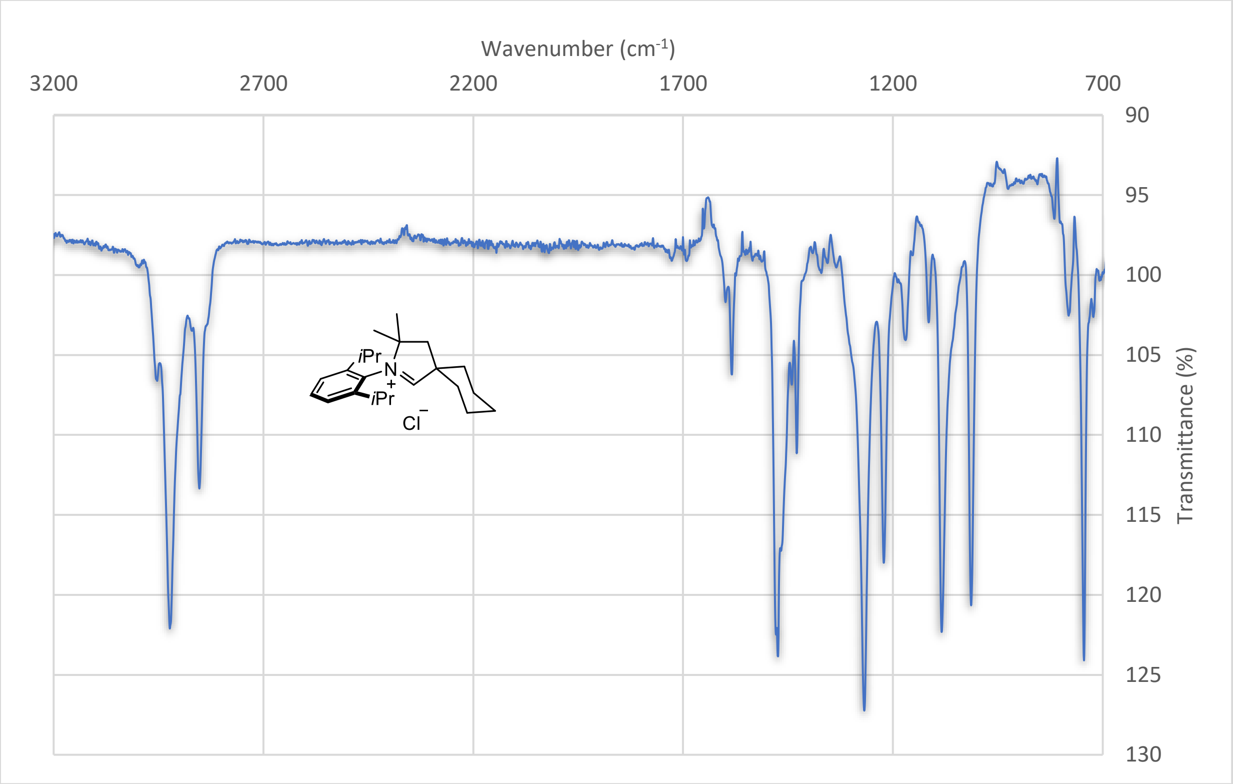

Figure S2: IR spectrum of CAAC ligand.

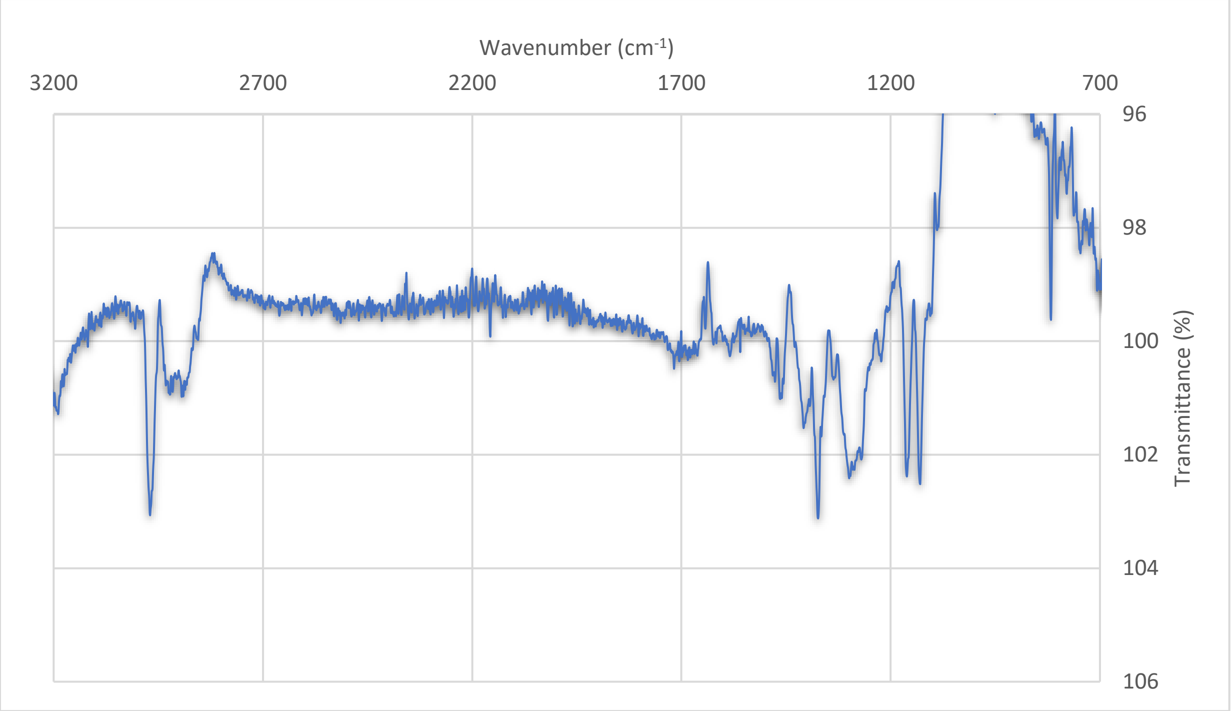

Figure S3: IR spectrum of black residue after catalysis.

24.Sensitivity Assessment

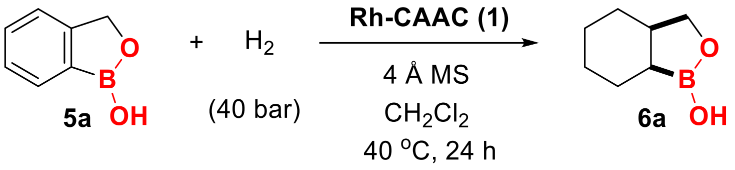

Preparation of stock solution A

To a 10 mL oven dried Schlenk flask was added DCM (5.4 mL) and Benzoxaborole (**5a**) (160.9 mg, 1.2 mmol). The reaction mixture was stirred for 5 min to have a homogeneous solution.

Standard Conditions:

To a 4 ml glass vial (screw-cap) equipped with a stir bar [Rh(CAAC)(COD)Cl] **1** (0.57 mg, 1 mol%) and pulverized 4 Å molecular sieve (50 mg) were added and the vial carefully evacuated. Under argon atmosphere, stock solution **A** (0.45 mL) and additional DCM (0.05 mL) were added. The glass vial was placed in a 150 ml stainless steel autoclave under an argon atmosphere. The autoclave was sealed, pressurized, and depressurized three times with hydrogen gas before the hydrogen pressure was set to 40 bar. The reaction mixture was stirred at 40 °C for 24. After this reaction time, the autoclave was carefully depressurized, mesitylene (14 µl) was added and the reaction mixture was analyzed by NMR.

Table S11: Sensitivity assessment for the hydrogenation of 6a.

#	Modification	Preparation	NMR Yield (%)	Deviation (%)
**1**	High *c*	0.45 mL stock sol.	>99	0
**2**	Low *c*	0.45 mL stock sol. + 0.05 mL DCM	>99	0
**3**	H_2O	Std. cond. + 5 µL H_2O	63	–37
**4**	Low O_2	Std. cond. + degassed	>99	0
**5**	High O_2	Reactor was performed under O_2 atmosphere	44	–54
**6**	Low *T*	Std. cond., T = 35 °C	85	–15
**7**	High *T*	Std. cond., T = 45 °C	>99	0
**8**	Low *p*	Std. cond., P = 35 bar	>99	0
**9**	High *p*	Std. cond., P = 45 bar	>99	0
**10**	Big scale	Reaction was performed in 2 mmol scale	>99	0

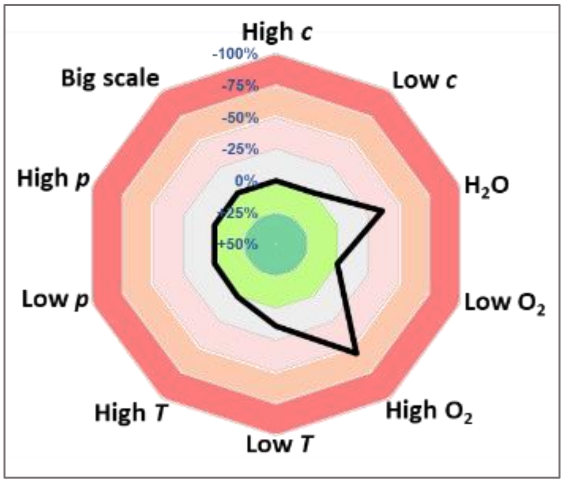

Figure S4: Radar diagram of the condition-based sensitivity screening.

25.Single Crystal X-Ray Diffraction Studies

X-Ray diffraction: Data sets for compounds **6d**, **6o**, **6r**, **7b**, and **8b** were collected with a Bruker D8 Venture PHOTON III diffractometer. Data sets for compound **6p** were collected with a Bruker APEXII CCD diffractometer. Programs used: data collection: APEX3 V2019.1-0 (Bruker AXS Inc., **2019**)[19]; cell refinement: SAINT V8.40A (Bruker AXS Inc., **2019**); data reduction: SAINT V8.40A (Bruker AXS Inc., **2019**); absorption correction, SADABS V2016/2 (Bruker AXS Inc., **2019**); structure solution *SHELXT-2015*[20] (Sheldrick, G. M. *Acta Cryst.,* **2015**, *A71,* 3-8); structure refinement *SHELXL-2015*[21] (Sheldrick, G. M. *Acta Cryst.*, **2015**, *C71* (1), 3-8) and graphics, *XP*[22] (Version 5.1, Bruker AXS Inc., Madison, Wisconsin, USA, **1998**). *R*-values are given for observed reflections, and wR^2 values are given for all reflections.

Exceptions and special features: For compound **7b** two CF_3 groups were found disordered over two positions in the asymmetric unit. Several restraints (SADI, SAME, ISOR and SIMU) were used in order to improve refinement stability.

25.1. X-ray crystal structure analysis of 6d (glo10173)

A colorless plate-like specimen of $C_8H_{12}BF_3O_2$, approximate dimensions 0.091 mm x 0.118 mm x 0.228 mm, was used for the X-ray crystallographic analysis. The X-ray intensity data were measured on a single crystal Bruker D8 Venture Photon III Diffractometer system equipped with a micro focus tube CuKα (CuKα, λ = 1.54178 Å) and a MX mirror monochromator. A total of 1671 frames were collected. The total exposure time was 19.83 hours. The frames were integrated with the Bruker SAINT software package using a wide-frame algorithm. The integration of the data using a monoclinic unit cell yielded a total of 16053 reflections to a maximum θ angle of 66.67° (0.84 Å resolution), of which 1646 were independent (average redundancy 9.753, completeness = 99.6%, R_{int} = 3.60%, R_{sig} = 1.94%) and 1520 (92.35%) were greater than 2σ(F^2). The final cell constants of a = 12.0776(3) Å, b = 8.0205(2) Å, c = 9.8867(3) Å, β = 102.8760(10)°, volume = 933.63(4) Å^3, are based upon the refinement of the XYZ-centroids of 9980 reflections above 20 σ(I) with 7.508° < 2θ < 133.2°. Data were corrected for absorption effects using the Multi-Scan method (SADABS). The ratio of minimum to maximum apparent transmission was 0.910. The calculated minimum and maximum transmission coefficients (based on crystal size) are 0.7670 and 0.8960. The structure was solved and refined using the Bruker SHELXTL Software Package, using the space group $P2_1/c$, with Z = 4 for the formula unit, $C_8H_{12}BF_3O_2$. The final anisotropic full-matrix least-squares refinement on F^2 with 131 variables converged at R1 = 2.92%, for the observed data and wR2 = 7.19% for all data. The goodness-of-fit was 1.023. The largest peak in the final difference electron density synthesis was 0.241 e^-/Å^3 and the largest hole was -0.174 e^-/Å^3 with an RMS deviation of 0.037 e^-/Å^3. On

the basis of the final model, the calculated density was 1.480 g/cm^3 and F(000), 432 e$^-$. CCDC Nr.: 2152160.

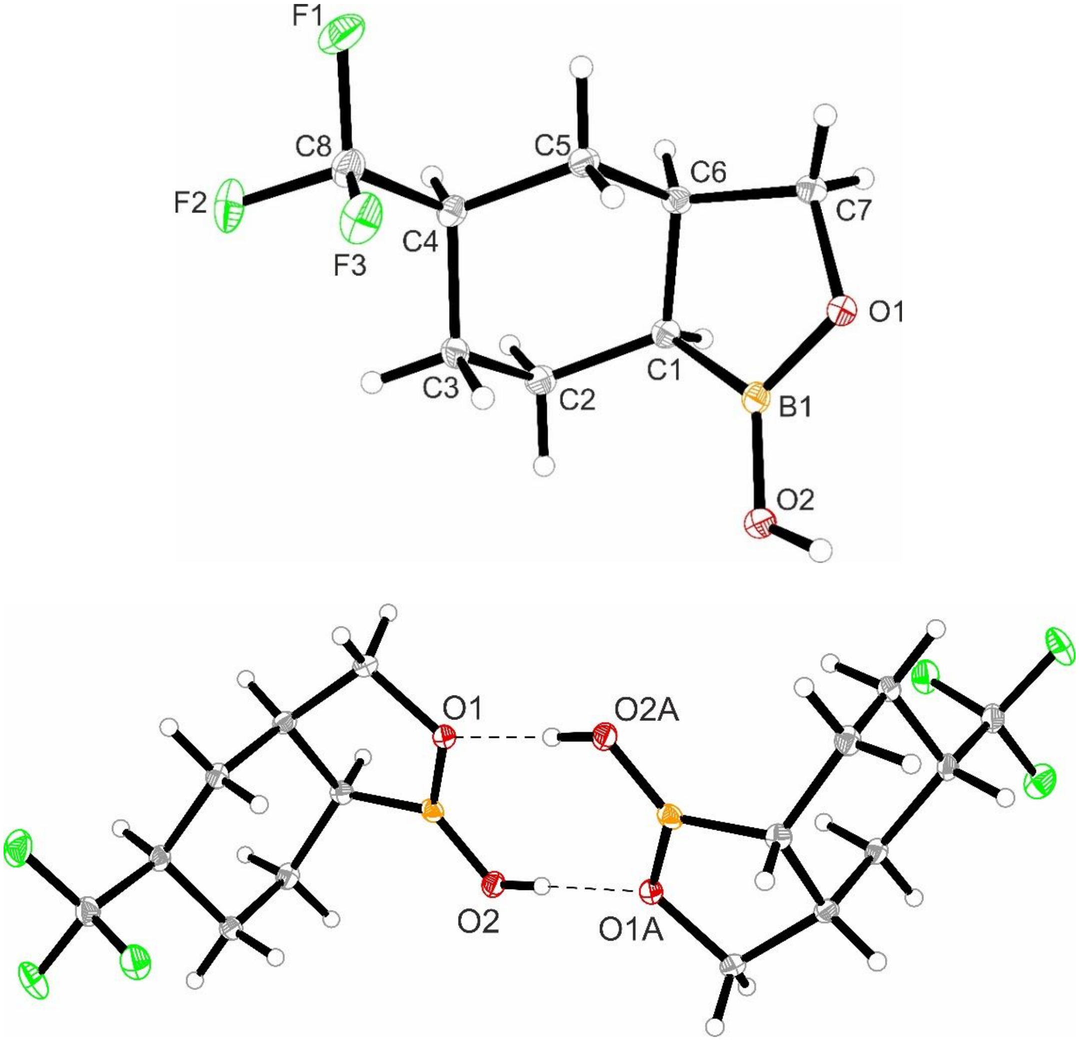

Figure S5: Crystal structure of compound 6d. Thermal ellipsoids are shown at 30% probability. Dimer type formation through O–H···O hydrogen bond interactions (head-to-head) between the hexahydrobenzoxaborole units.

Specified hydrogen bonds:

D-H	H...A	D...A	<(DHA)	
0.86(2)	1.91(2)	2.7774(13)	178.1(18)	O2-H2...O1_$1 (-x+1, -y+1, -z+1)

25.2. X-ray crystal structure analysis of 6o (glo10256)

A colorless, prism-like specimen of $C_{10}H_{19}BO_2$, approximate dimensions 0.112 mm x 0.172 mm x 0.209 mm, was used for the X-ray crystallographic analysis. The X-ray intensity data were measured on a single crystal diffractometer Bruker D8 Venture Photon III system equipped with a micro focus tube Cu ImS (CuKα, λ = 1.54178 Å) and a MX mirror monochromator. A total of 1606 frames were collected. The total exposure time was 17.71 hours. The frames were integrated with the Bruker SAINT software package using a wide-frame algorithm. The integration of the data using a monoclinic unit cell yielded a total of 19030 reflections to a maximum θ angle of 66.55° (0.84 Å resolution), of which 1838 were independent (average redundancy 10.354, completeness = 98.2%, R_{int} = 4.87%, R_{sig} = 2.36%) and 1698 (92.38%) were greater than $2\sigma(F^2)$. The final cell constants of a = 5.2644(2) Å, b = 15.7743(4) Å, c = 12.8358(4) Å, β = 95.8230(10)°, volume = 1060.41(6) $Å^3$, are based upon the refinement of the XYZ-centroids of 9980 reflections above 20 σ(I) with 13.87° < 2θ < 133.0°. Data were corrected for absorption effects using the Multi-Scan method (SADABS). The ratio of minimum to maximum apparent transmission was 0.877. The calculated minimum and maximum transmission coefficients (based on crystal size) are 0.8870 and 0.9370. The structure was solved and refined using the Bruker SHELXTL Software Package, using the space group $P2_1/n$, with Z = 4 for the formula unit, $C_{10}H_{19}BO_2$. The final anisotropic full-matrix least-squares refinement on F^2 with 124 variables converged at R1 = 3.91%, for the observed data and wR2 = 9.76% for all data. The goodness-of-fit was 1.041. The largest peak in the final difference electron density synthesis was 0.298 $e^-/Å^3$ and the largest hole was -0.179 $e^-/Å^3$ with an RMS deviation of 0.034 $e^-/Å^3$. On the basis of the final model, the calculated density was 1.140 g/cm^3 and F(000), 400 e^-. CCDC Nr.: 2152161.

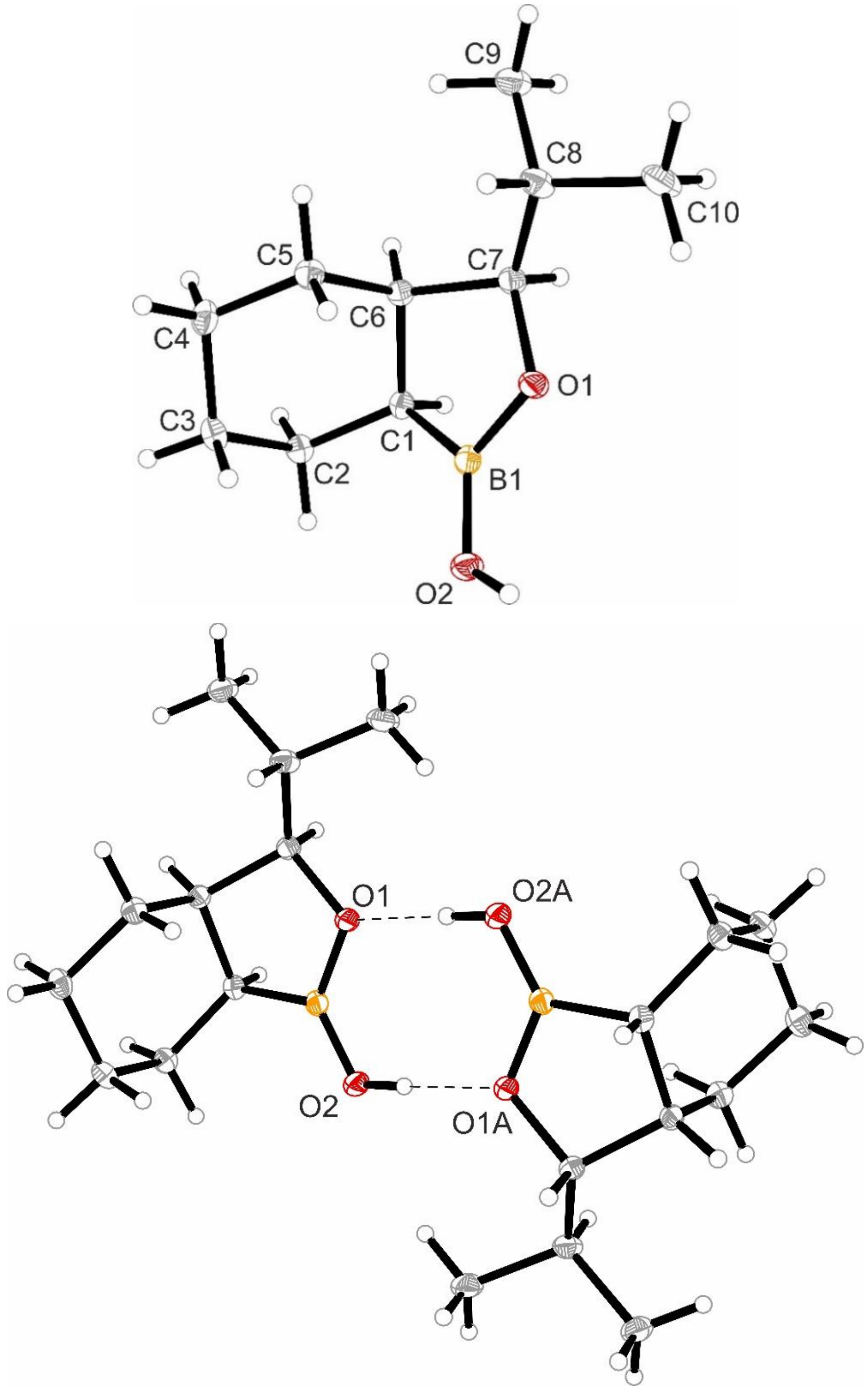

Figure S6: Crystal structure of compound 6o. Thermal ellipsoids are shown at 30% probability. Dimer type formation through O–H···O hydrogen bond interactions (head-to-head) between the hexahydrobenzoxaborole units.

Specified hydrogen bonds:

D-H	H...A	D...A	<(DHA)		
0.897(15)	1.859(15)	2.7563(12)	178.3(18)	O2-H2...O1_$1	(-x, -y+1, -z+1)

25.3. X-ray crystal structure analysis of 6p (glo10234)

A colorless, prism-like specimen of $C_{11}H_{23}BO_2Si$, approximate dimensions 0.120 mm x 0.160 mm x 0.280 mm, was used for the X-ray crystallographic analysis. The X-ray intensity data were measured (λ = 1.54178 Å). A total of 1312 frames were collected. The total exposure time was 19.43 hours. The frames were integrated with the Bruker SAINT software package using a wide-frame algorithm. The integration of the data using an orthorhombic unit cell yielded a total of 23639 reflections to a maximum θ angle of 66.62 (0.84 Å resolution), of which 2407 were independent (average redundancy 9.821, completeness = 99.8%, R_{int} = 5.24%, R_{sig} = 2.61%) and 2079 (86.37%) were greater than $2\sigma(F^2)$. The final cell constants of a = 12.8524(3) Å, b = 10.5201(2) Å, c = 20.1607(4) Å, volume = 2725.90(10) Å^3, are based upon the refinement of the XYZ-centroids of 6455 reflections above 20 σ(I) with 8.772° < 2θ < 133.1°. Data were corrected for absorption effects using the multi-Scan method (SADABS). The ratio of minimum to maximum apparent transmission was 0.902. The calculated minimum and maximum transmission coefficients (based on crystal size) are 0.7030 and 0.8540. The structure was solved and refined using the Bruker SHELXTL Software Package, using the space group *Pbca*, with Z = 8 for the formula unit, $C_{11}H_{23}BO_2Si$. The final anisotropic full-matrix least-squares refinement on F^2 with 156 variables converged at R1 = 4.25%, for the observed data and wR2 = 12.43% for all data. The goodness-of-fit was 1.034. The largest peak in the final difference electron density synthesis was 0.513 e^-/Å^3 and the largest hole was -0.290 e^-/Å^3 with an RMS deviation of 0.045 e^-/Å^3. On the basis of the final model, the calculated density was 1.102 g/cm^3 and F(000), 992 e^-. CCDC Nr.: 2152162.

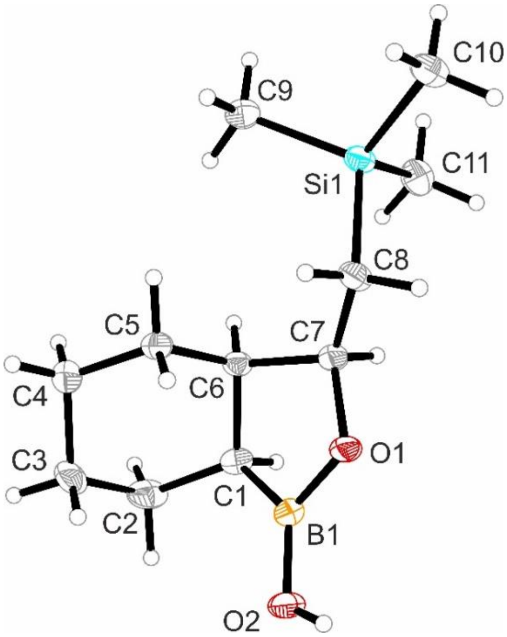

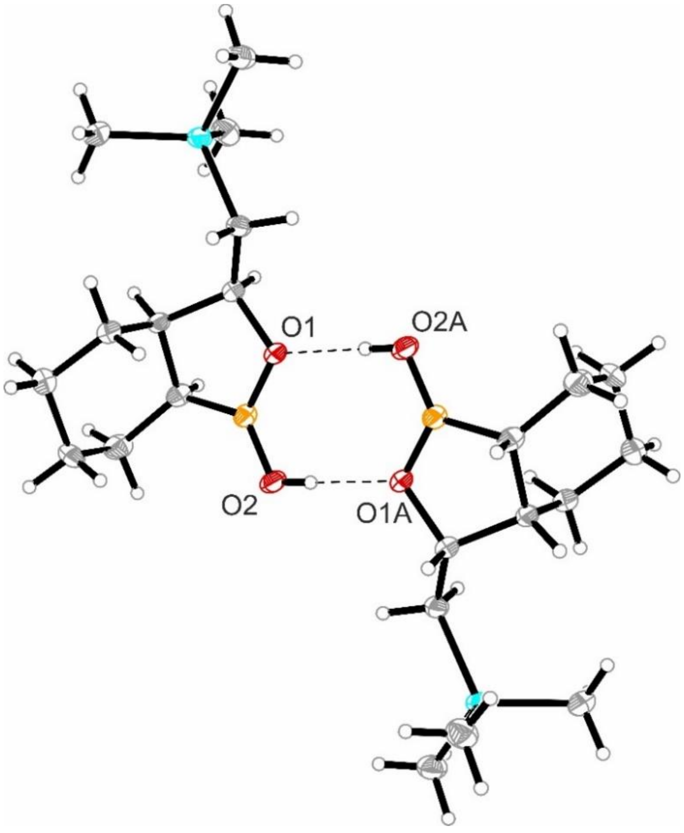

Figure S7: Crystal structure of compound 6p. Thermal ellipsoids are shown at 30% probability. Dimer type formation through O–H···O hydrogen bond interactions (head-to-head) between the hexahydrobenzoxaborole units.

Specified hydrogen bonds:

D-H	H...A	D...A	<(DHA)	
0.82(3)	1.97(3)	2.7857(19)	177(3)	O2-H2...O1_$1 (-x+1, -y+1, -z+1)

25.4. X-ray crystal structure analysis of 6r (glo10254)

A colorless, plate-like specimen of $C_{11}H_{21}BO_3$, approximate dimensions 0.050 mm x 0.067 mm x 0.124 mm, was used for the X-ray crystallographic analysis. The X-ray intensity data were measured on a single crystal diffractometer Bruker D8 Venture Photon III system equipped with a micro focus tube Cu ImS (CuKα, λ = 1.54178 Å) and a MX mirror monochromator. A total of 1815 frames were collected. The total exposure time was 34.72 hours. The frames were integrated with the Bruker SAINT software package using a wide-frame algorithm. The integration of the data using a monoclinic unit cell yielded a total of 23223 reflections to a maximum θ angle of 66.62° (0.84 Å resolution), of which 2097 were independent (average redundancy 11.074, completeness = 99.8%, R_{int} = 8.85%, R_{sig} = 3.63%) and 1622 (77.35%) were greater than 2σ(F^2). The final cell constants of a = 5.1352(2) Å, b = 12.9768(4) Å, c = 17.9339(5) Å, β = 90.875(2)°, volume = 1194.95(7) $Å^3$, are based upon the refinement of the XYZ-centroids of 5037 reflections above 20 σ(I) with 8.410° < 2θ < 132.8°. Data were corrected for absorption effects using the multi-Scan method (SADABS). The ratio of minimum to maximum apparent transmission was 0.921. The calculated minimum and maximum transmission coefficients (based on crystal size) are 0.9230 and 0.9680. The structure was solved and refined using the Bruker SHELXTL Software Package, using the space group $P2_1/n$, with Z = 4 for the formula unit, $C_{11}H_{21}BO_3$. The final anisotropic full-matrix least-squares refinement on F^2 with 142 variables converged at R1 = 4.12%, for the observed data and wR2 = 9.69% for all data. The goodness-of-fit was 1.053. The largest peak in the final difference electron density synthesis was 0.210 $e^-/Å^3$ and the largest hole was -0.189 $e^-/Å^3$ with an RMS deviation of 0.045 $e^-/Å^3$. On the basis of the final model, the calculated density was 1.179 g/cm^3 and F(000), 464 e^-. CCDC Nr.: 2152163.

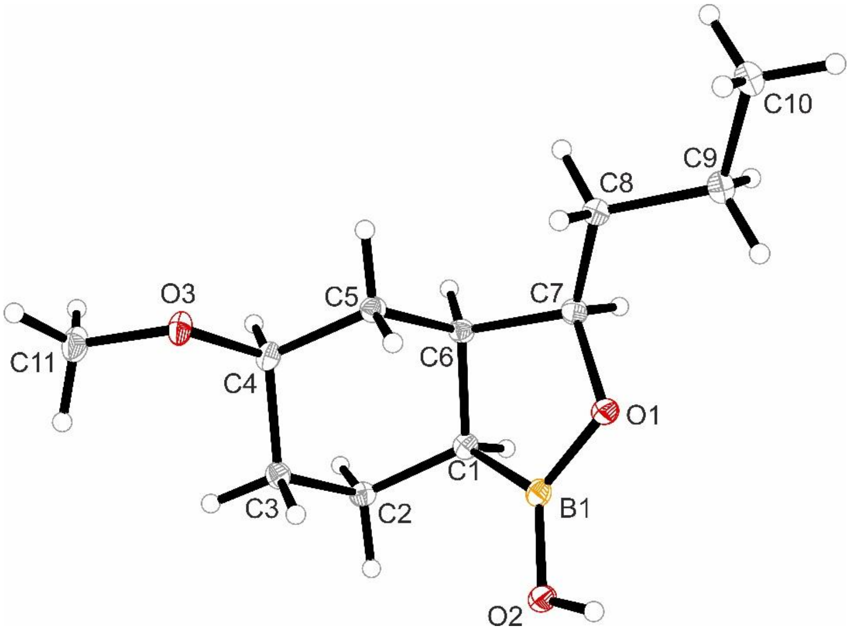

Figure S8: Crystal structure of compound 6r. Thermal ellipsoids are shown at 30% probability.

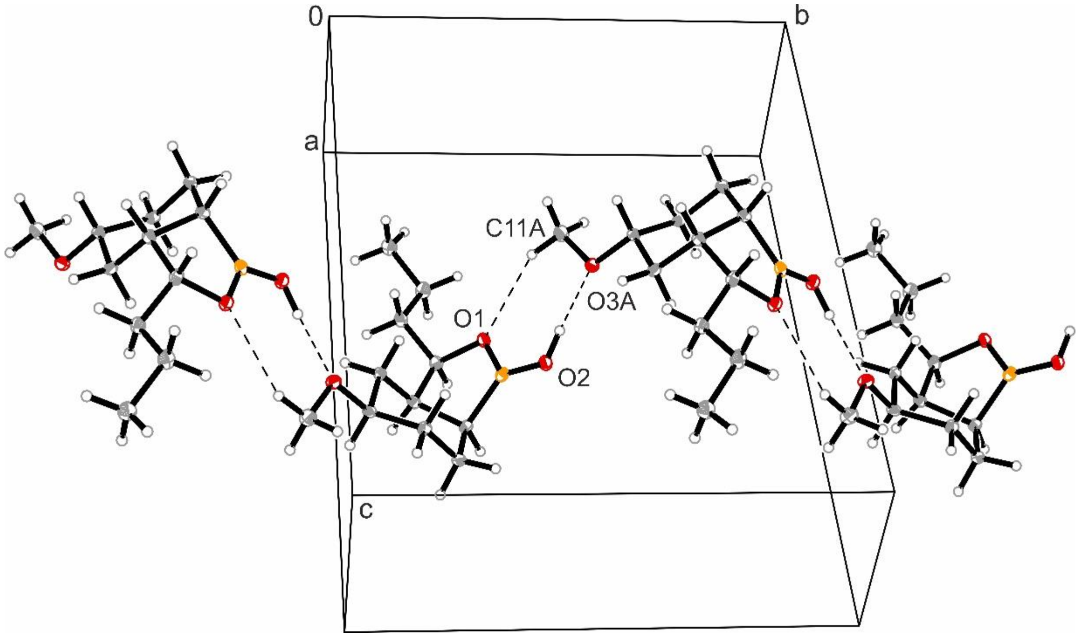

Figure S9: Excerpt of the packing diagram of compound 6r representing the formation of a chain along "b"-axis through O–H···O and C–H···O interaction (head-to-tail) between the hexahydrobenzoxaborole unit and the methoxy substituent.

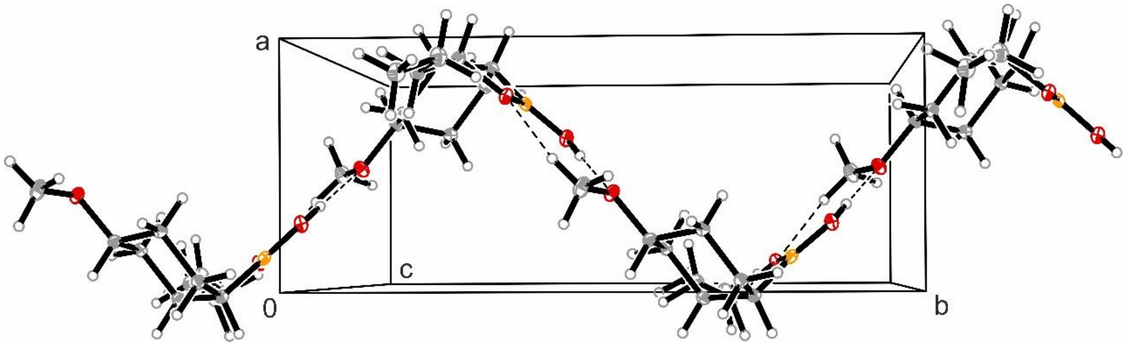

Figure S10: Second view perpendicular on the ab-plane representing the corresponding zig-zag chain.

Specified hydrogen bonds (with esds except fixed and riding H):

D-H	H...A	D...A	<(DHA)		
0.894(17)	1.856(17)	2.7493(17)	177(2)	O2-H2...O3_$1	(-x+1/2, y-1/2, -z+3/2)
0.980	2.482	3.331	144.71	C11-H11...O1_$1	(-x+1/2, y-1/2, -z+3/2)

25.5. X-ray crystal structure analysis of 7b (glo10313)

A colorless, plate-like specimen of $C_9H_8BF_3O_2$, approximate dimensions 0.054 mm x 0.126 mm x 0.157 mm, was used for the X-ray crystallographic analysis. The X-ray intensity data were measured on a single crystal diffractometer Bruker D8 Venture Photon III system equipped with a micro focus tube Mo ImS (MoKα, λ = 0.71073 Å) and a MX mirror monochromator. A total of 783 frames were collected. The total exposure time was 8.70 hours. The frames were integrated with the Bruker SAINT software package using a narrow-frame algorithm. The integration of the data using a monoclinic unit cell yielded a total of 38293 reflections to a maximum θ angle of 26.79 (0.79 Å resolution), of which 3996 were independent (average redundancy 9.583, completeness = 99.6%, R_{int} = 4.23%, R_{sig} = 2.02%) and 3299 (82.56%) were greater than 2σ(F^2). The final cell constants of a = 6.71300(10) Å, b = 16.2246(4) Å, c = 17.3371(4) Å, β = 97.3550(10)°, volume = 1872.75(7) $Å^3$, are based upon the refinement of the XYZ-centroids of 9987 reflections above 20 σ(I) with 4.738° < 2θ < 53.54°. Data were corrected for absorption effects using the Multi-Scan method (SADABS). The ratio of minimum to maximum apparent transmission was 0.949. The calculated minimum and maximum transmission coefficients (based on crystal size) are 0.9780 and 0.9920. The structure was solved and refined using the Bruker SHELXTL Software Package, using the space group $P2_1/n$, with Z = 8 for the formula unit, $C_9H_8BF_3O_2$. The final anisotropic full-matrix least-squares refinement on F^2 with 335 variables converged at R1 = 3.55%, for the observed data and wR2 = 9.65% for all data. The goodness-of-fit was 1.071. The largest peak in the final difference electron density synthesis was 0.381 $e^-/Å^3$ and the largest hole was -0.206 $e^-/Å^3$ with an RMS deviation of 0.040 $e^-/Å^3$. On

the basis of the final model, the calculated density was 1.532 g/cm^3 and F(000), 880 e$^-$. CCDC Nr.: 2169058.

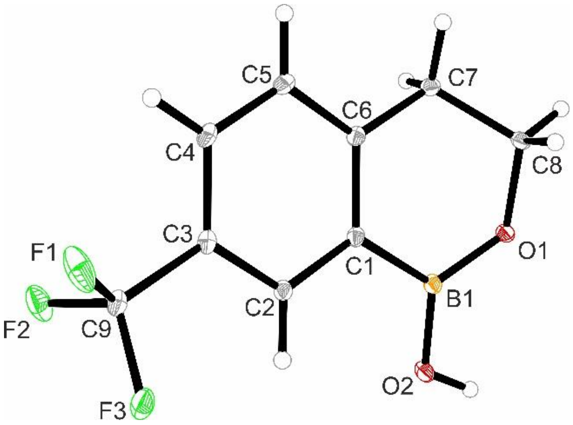

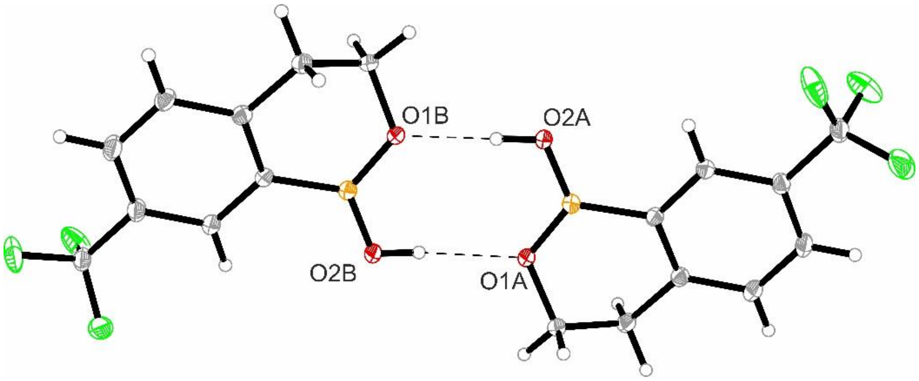

Figure S11:: Crystal structure of compound 7b. Thermal ellipsoids are shown at 15% probability. Dimer type formation through O-H···O hydrogen bond interactions (head-to-head) between the benzoxaborole units.

Specified hydrogen bonds:

D-H	H...A	D...A	<(DHA)	
0.874(16)	1.859(16)	2.7285(13)	173(2)	O2A-H21...O1B
0.868(15)	2.033(16)	2.8960(13)	173(2)	O2B-H22...O1A

25.6. X-ray crystal structure analysis of 8b (glo10318)

A colorless, plate-like specimen of $C_9H_{14}BF_3O_2$, approximate dimensions 0.033 mm x 0.087 mm x 0.235 mm, was used for the X-ray crystallographic analysis. The X-ray intensity data were measured on a single crystal diffractometer Bruker D8 Venture Photon III system equipped with a micro focus tube Cu ImS (CuKα, λ = 1.54178 Å) and a MX mirror monochromator. A total of 1562 frames were collected. The total exposure time was 19.04 hours. The frames were integrated with the Bruker SAINT software package using a wide-frame algorithm. The integration of the data using a monoclinic unit cell yielded a total of 17797 reflections to a maximum θ angle of 66.61° (0.84 Å resolution), of which 1780 were independent

(average redundancy 9.998, completeness = 99.0%, R_{int} = 4.47%, R_{sig} = 2.17%) and 1639 (92.08%) were greater than 2σ(F^2). The final cell constants of a = 10.6359(3) Å, b = 10.1337(3) Å, c = 10.3905(3) Å, β = 114.8860(10)°, volume = 1015.91(5) $Å^3$, are based upon the refinement of the XYZ-centroids of 9966 reflections above 20 σ(I) with 9.165° < 2θ < 133.0°. Data were corrected for absorption effects using the Multi-Scan method (SADABS). The ratio of minimum to maximum apparent transmission was 0.840. The calculated minimum and maximum transmission coefficients (based on crystal size) are 0.7710 and 0.9630. The structure was solved and refined using the Bruker SHELXTL Software Package, using the space group $P2_1/c$, with Z = 4 for the formula unit, $C_9H_{14}BF_3O_2$. The final anisotropic full-matrix least-squares refinement on F^2 with 140 variables converged at R1 = 2.99%, for the observed data and wR2 = 7.80% for all data. The goodness-of-fit was 1.051. The largest peak in the final difference electron density synthesis was 0.282 $e^-/Å^3$ and the largest hole was -0.166 $e^-/Å^3$ with an RMS deviation of 0.036 $e^-/Å^3$. On the basis of the final model, the calculated density was 1.452 g/cm^3 and F(000), 464 e^-. CCDC Nr.: 2169059.

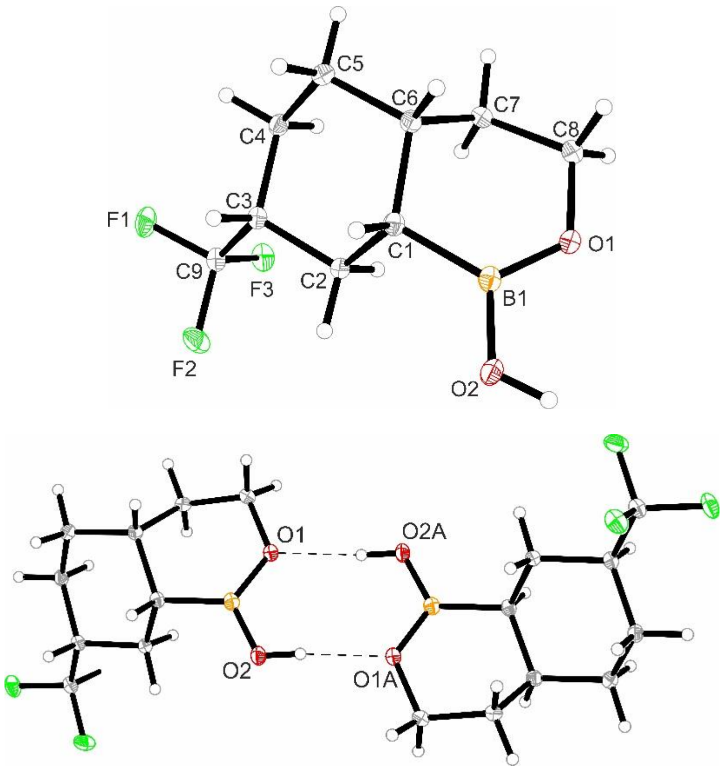

Figure S12: Crystal structure of compound 8b. Thermal ellipsoids are shown at 30% probability. Dimer type formation through O-H···O hydrogen bond interactions (head-to-head) between the hexahydrobenzoxaborole units.

26. ^{1}H NMR binding studies of 6a with *D*-glucose, *D*-fructose, and methyl-*α*-*D*-glucopyranoside

Preparation of sodium phosphate buffer solution (0.1 M). In an Erlenmeyer flask equipped with a PTFE-coated stirring bar, sodium phosphate monobasic (156.0 mg) was dissolved in D_2O (10 mL) and the pH was adjusted to 7.40±0.01 by adding NaOH 2 M solution (controlled using a pH-meter).

Preparation of 6a solution (0.1 M): In a round bottom flask equipped with a PTFE-coated stirring bar, **6a** (56.0 mg) was dissolved in phosphate buffer (4 mL) and the pH was adjusted to 7.40±0.01 by adding NaOH 2 M solution (controlled using a pH-meter).

Preparation of glucose solution (0.1 M): In a round bottom flask equipped with a PTFE-coated stirring bar, glucose (18.0 mg) was dissolved in phosphate buffer (1 mL) and the pH was adjusted to 7.40±0.01 by adding NaOH 2 M solution (controlled using a pH-meter).

Preparation of fructose solution (0.1 M): In a round bottom flask equipped with a PTFE-coated stirring bar, fructose (18.0 mg) was dissolved in phosphate buffer (1 mL) and the pH was adjusted to 7.40±0.01 by adding NaOH 2 M solution (controlled using a pH-meter).

Preparation of methyl-*α*-*D*-glucopyranoside solution (0.1 M): In a round bottom flask equipped with a PTFE-coated stirring bar, Methyl-*α*-*D*-glucopyranoside (19.4 mg) was dissolved in phosphate buffer (1 mL) and the pH was adjusted to 7.40±0.01 by adding NaOH 2 M solution (controlled using a pH-meter).

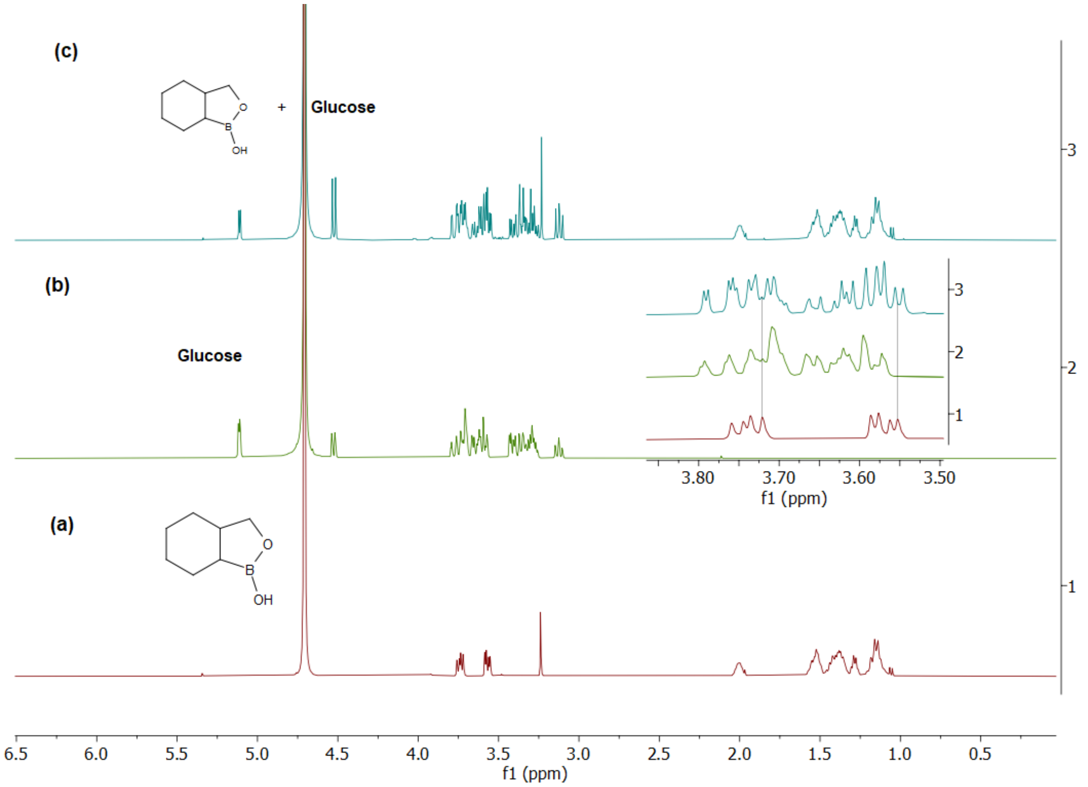

Figure S13: **^{1}H NMR (400 MHz) spectrum of a solution of 6a (0.1 M) in phosphate buffered D_2O solution (0.1 M) at pH 7.4 (a). Glucose (0.1 M) in phosphate buffered D_2O solution (0.1 M) at pH 7.4 (b). Mixture of 6a (0.1 M) and glucose (0.1 M) in phosphate buffered D_2O solution (0.1 M) at pH 7.4 (c).**

Explanation: By ^{1}H NMR analysis, the mixture of **6a** and glucose shows a considerable chemical shift in comparison to the separate reaction solution of 6a **(a)** and glucose **(b)**. The change in the chemical shift indicates that there is an interaction or bound complex formation between **6a** and glucose.

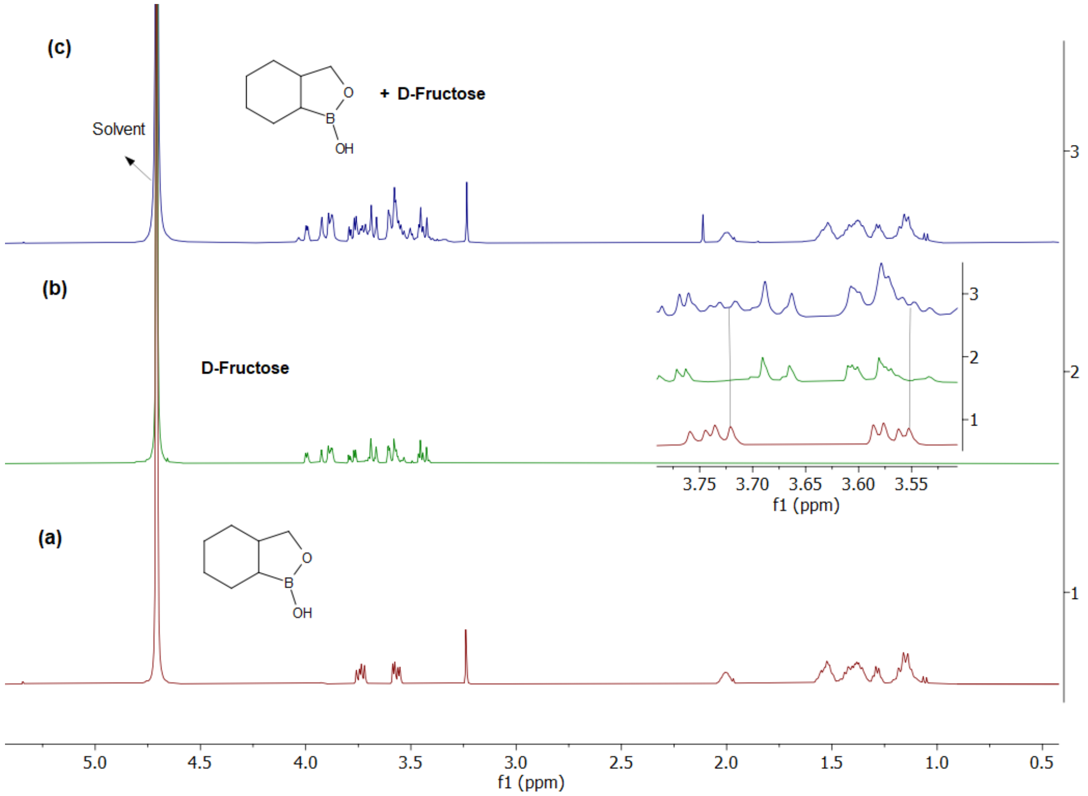

Figure S14: ^{1}H NMR (400 MHz) spectrum of a solution of 6a (0.1 M) in phosphate buffered D_2O solution (0.1 M) at pH 7.4 (a). D-Fructose (0.1 M) in phosphate buffered D_2O solution (0.1 M) at pH 7.4 (b). Mixture of 6a (0.1 M) and D-Fructose (0.1 M) in phosphate buffered D_2O solution (0.1 M) at pH 7.4 (c).

Explanation: By ^{1}H NMR analysis, the mixture of **6a** and *D*-Fructose shows a considerable chemical shift in comparison to the separate reaction solution of 6a **(a)** and *D*-Fructose **(b)**. The change in the chemical shift indicates that there is an interaction or bound complex formation between **6a** and *D*-Fructose.

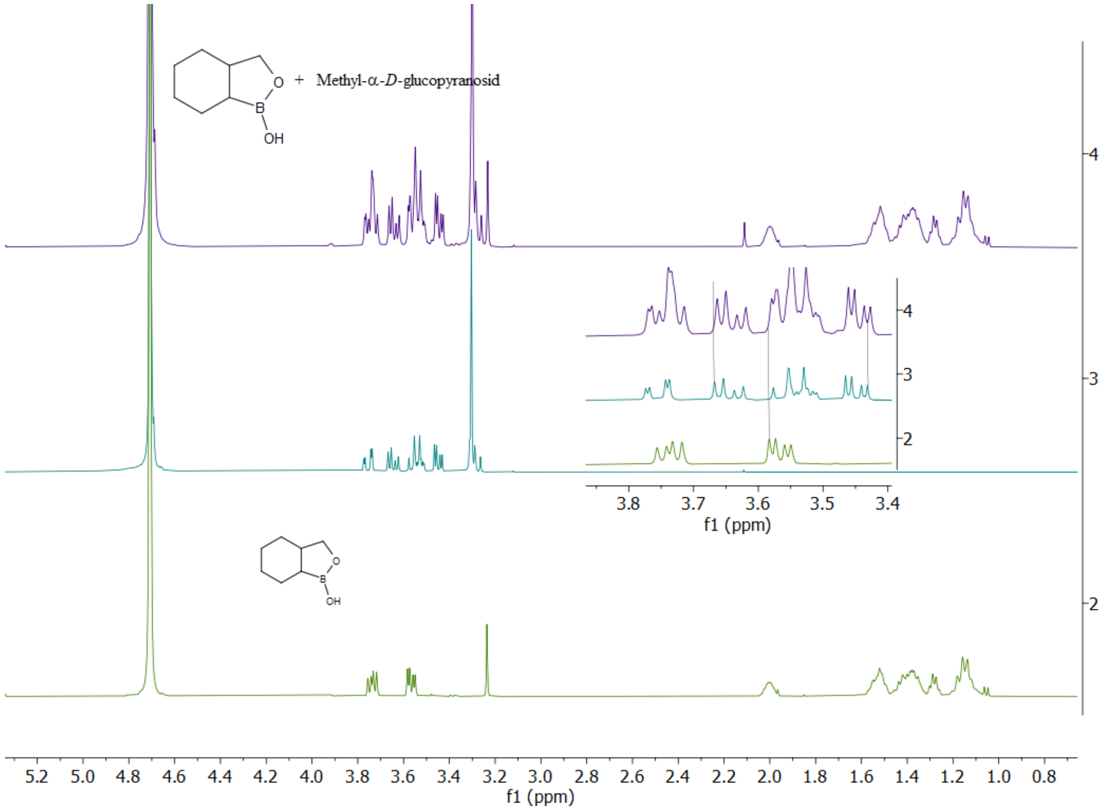

Figure S15: ^{1}H NMR (400 MHz) spectrum of a solution of 6a (0.1 M) in phosphate buffered D_2O solution (0.1 M) (0.4 mL) at pH 7.4 (a). Methyl-*α*-*D*-glucopyranoside (0.1 M) in phosphate buffered D_2O solution (0.1 M) at pH 7.4 (b). Mixture of 6a (0.1 M) and Methyl-*α*-*D*-glucopyranoside (0.1 M) in phosphate buffered D_2O solution (0.1 M) at pH 7.4 (c).

Explanation: By ^{1}H NMR analysis, the mixture of **6a** and Methyl-*α*-*D*-glucopyranoside shows considerable chemical shift in comparison to the separate reaction solution of 6a **(a)** and Methyl-*α*-*D*-glucopyranoside **(b)**. The change in the chemical shift and peak broadening indicates that there is an interaction or bound complex formation between **6a** and Methyl-*α*-*D*-glucopyranoside.

27. Colorimetric assay and competitive binding experiment

Preparation of sodium phosphate buffer solution (0.1 M). In an Erlenmeyer flask equipped with a PTFE-coated stirring bar, sodium phosphate monobasic (3.0 g) was dissolved in deionized water (250 ml) and the pH was adjusted to 7.40±0.01 by adding NaOH 2 M solution (controlled using a pH-meter).

Preparation of the ARS solution (10^{-4} M). In a round-bottom flask, alizarin red S (17.1 mg) was dissolved in the pH 7.40 buffer solution (50 ml) and gently stirred for 5 minutes, then a 1.0 ml aliquot was transferred to another flask and diluted with 9.0 ml of pH 7.40 buffer solution to obtain a 10^{-4} M ARS solution.

UV-visible measurements. UV-visible spectra were recorded using a Jasco V-650 spectrophotometer, equipped with a temperature control unit at 25 °C. The samples were measured in Starna Fluorometer Semi-Micro quartz cuvettes (volume: 1.4 ml, path length: 10 mm) equipped with a PTFE-stopper. The spectra have been acquired from 350 to 700 nm using 0.5 nm steps. Three samples were measured: (**I**) ARS 10^{-4} M solution (red line); (**II**) ARS 10^{-4} M solution and 0.02 M of **6a** (green line); (**III**) ARS 10^{-4} M solution and 0.02 M of **6a**, with added *D*-glucose (0.5 M) (yellow line).

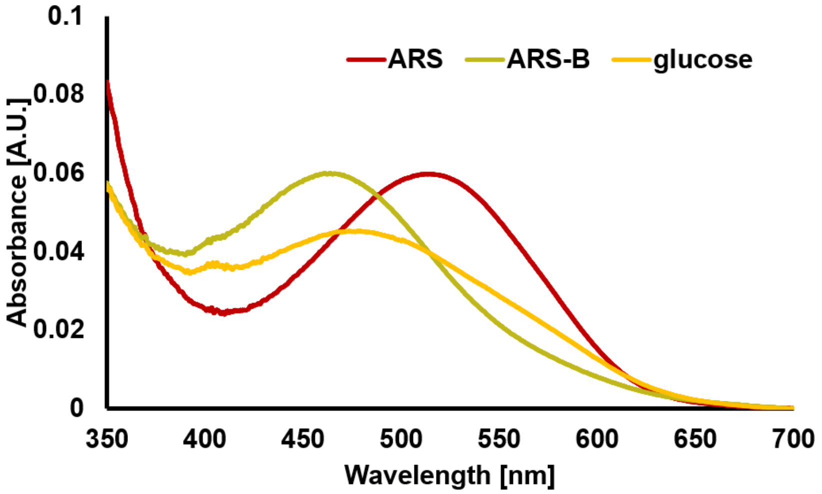

Figure S16: UV-visible spectra for the detection of diol (ARS) and Glucose using 6a.

28. Stability of compound 6a in monobasic phosphate buffered D_2O solution (pH = 7.4)

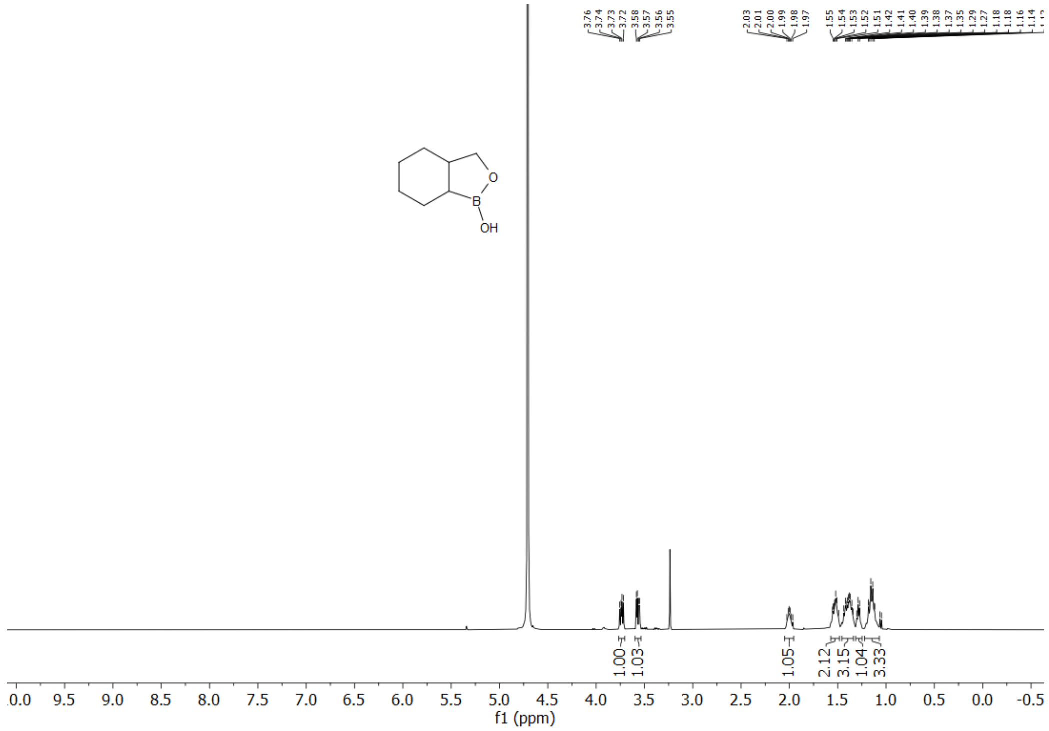

Figure S17: ^1H NMR (400 MHz, D_2O, 298 K) spectrum of Hexahydrobenzo[*c*][1,2]oxaborol-1(3*H*)-ol (6a) after 0 h.

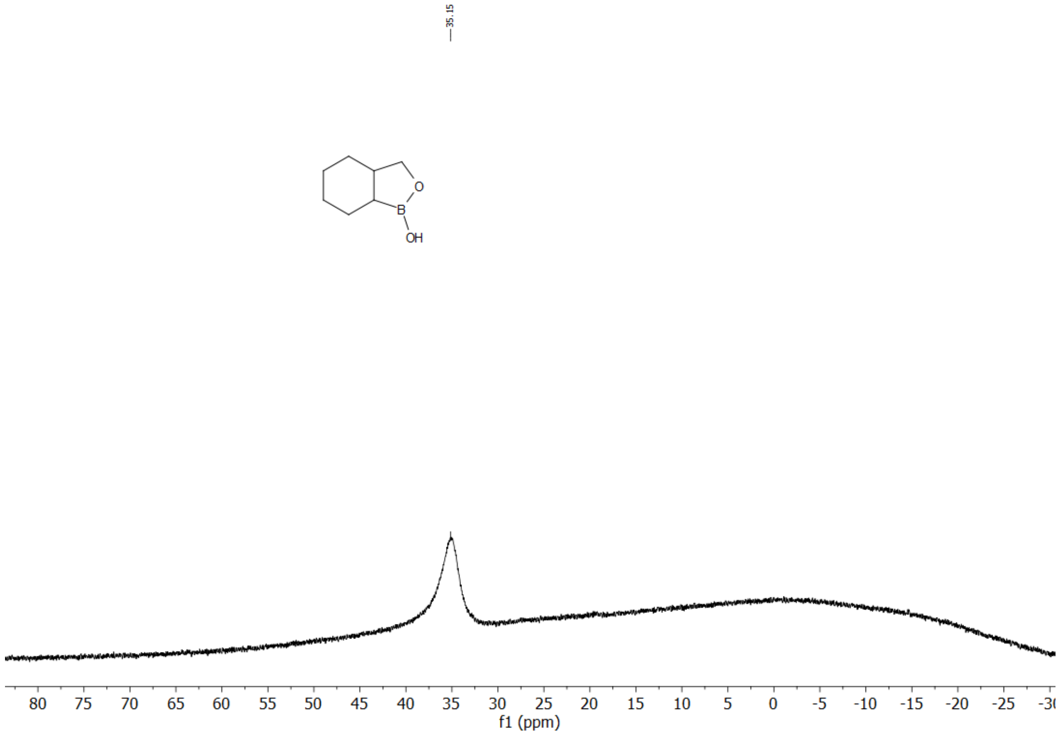

Figure S18: $^{11}B\{^1H\}$ NMR (128 MHz, D_2O, 298 K) spectrum of Hexahydrobenzo[*c*][1,2]oxaborol-1(3*H*)-ol (6a) after 0 h.

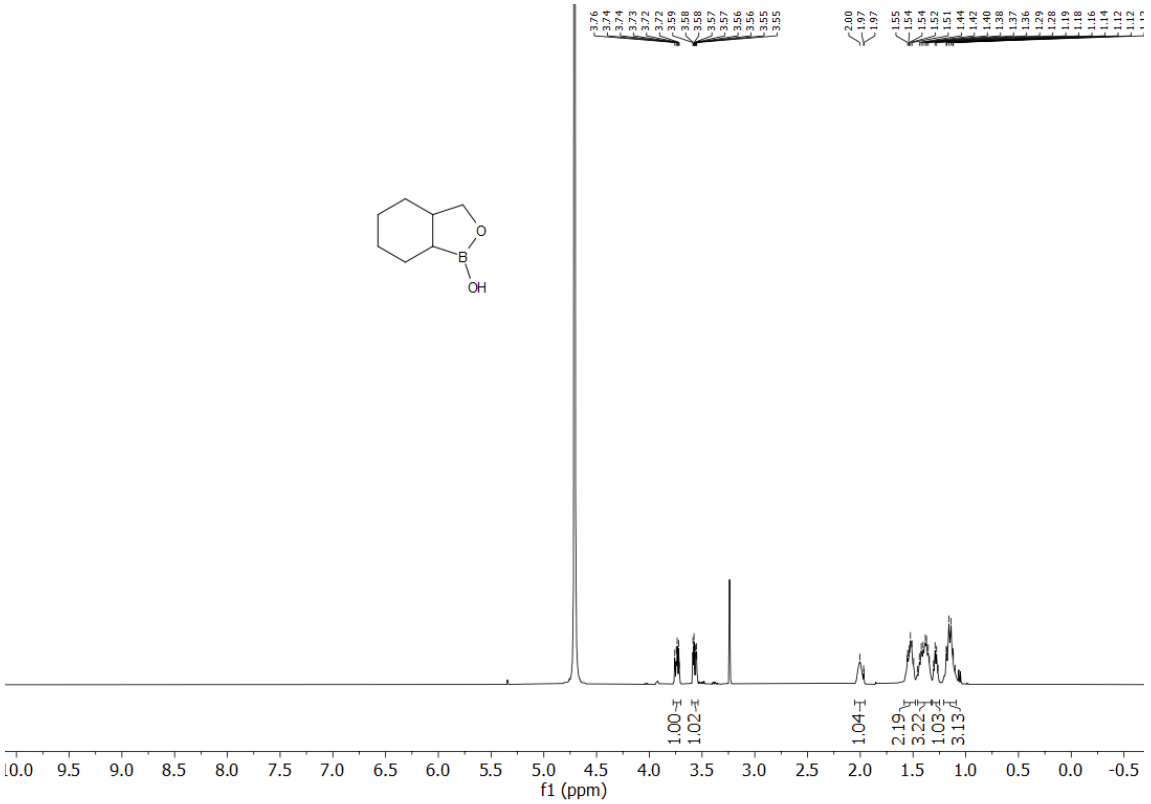

Figure S19: ^{1}H NMR (400 MHz, D_2O, 298 K) spectrum of Hexahydrobenzo[*c*][1,2]oxaborol-1(3*H*)-ol (6a) after 12 h.

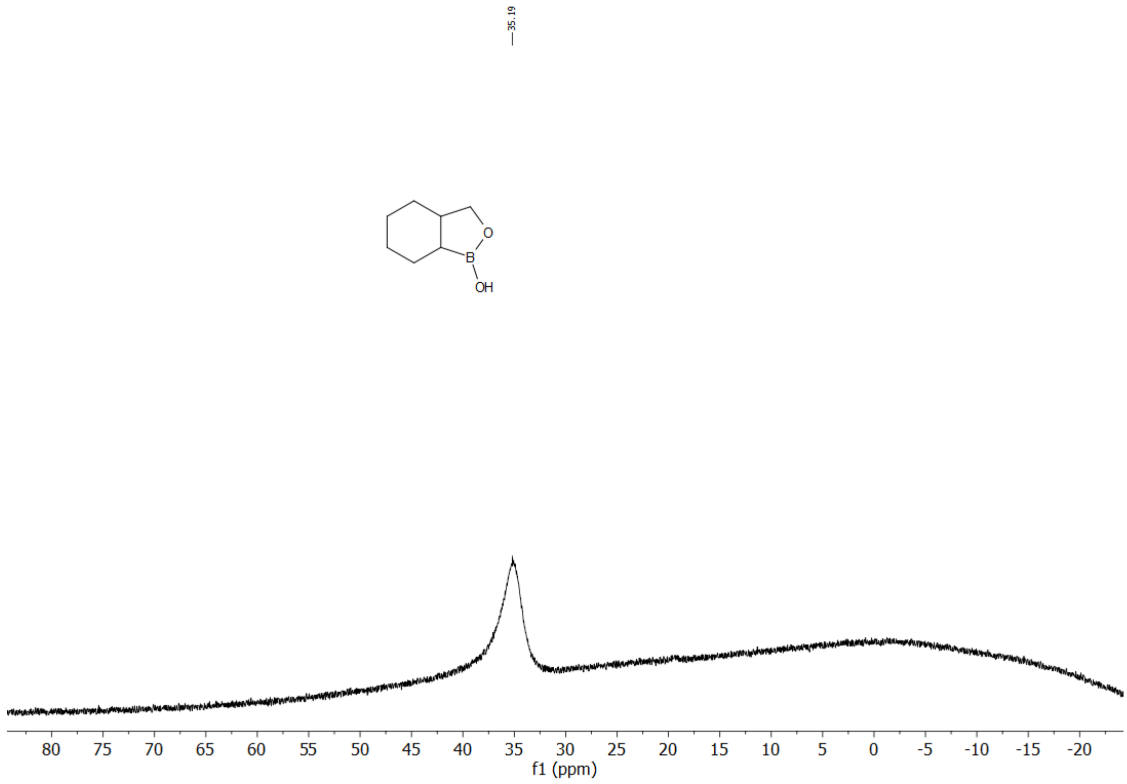

Figure S20: $^{11}B\{^{1}H\}$ NMR (128 MHz, D_2O, 298 K) spectrum of Hexahydrobenzo[*c*][1,2]oxaborol-1(3*H*)-ol (6a) after 12 h.

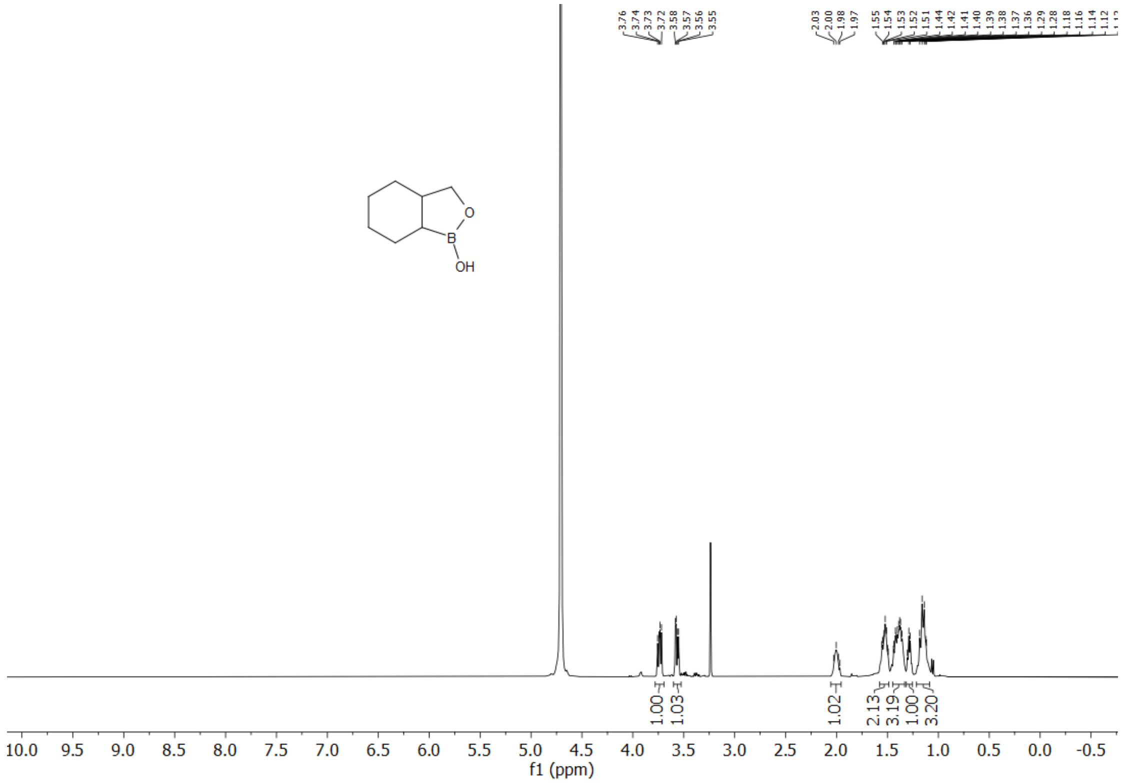

Figure S21: ^{1}H NMR (400 MHz, D_2O, 298 K) spectrum of Hexahydrobenzo[*c*][1,2]oxaborol-1(3*H*)-ol (6a) after 30 days.

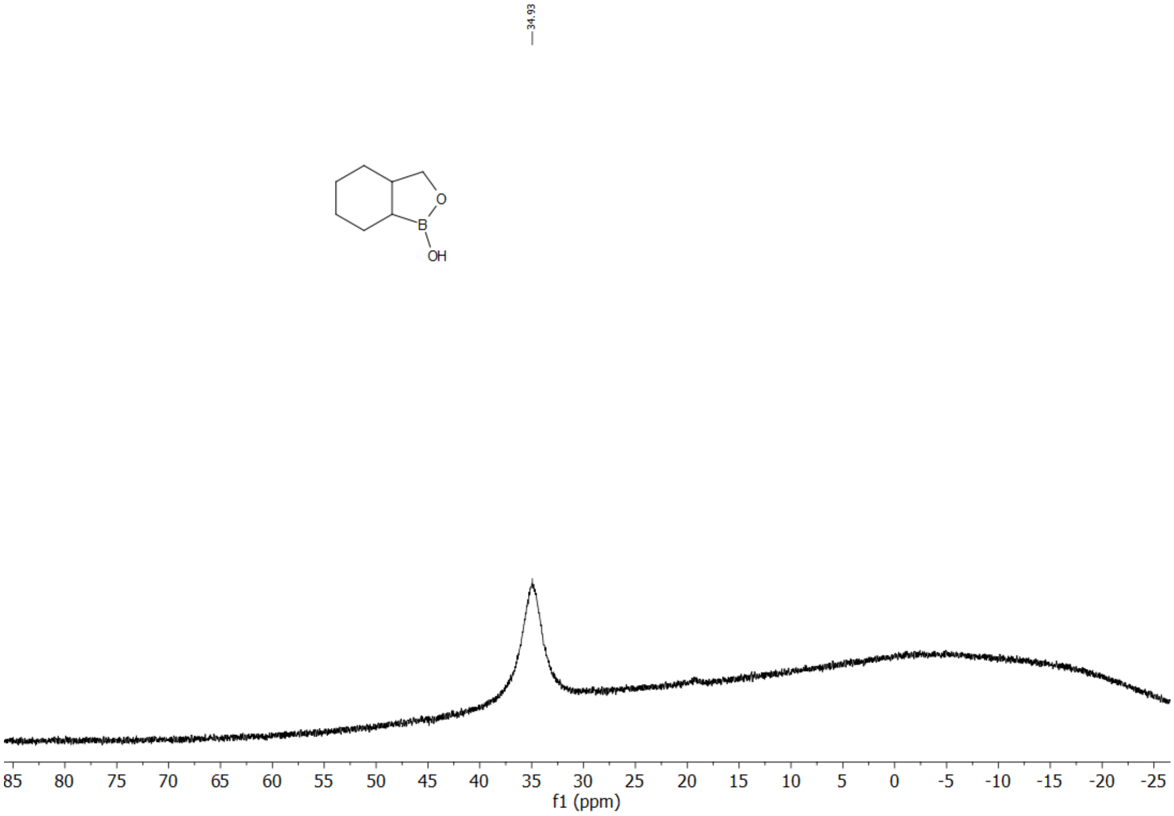

Figure S22: $^{11}B\{^{1}H\}$ NMR (128 MHz, D_2O, 298 K) spectrum of Hexahydrobenzo[*c*][1,2]oxaborol-1(3*H*)-ol (6a) after 30 days.

29.Spectral data of the starting materials

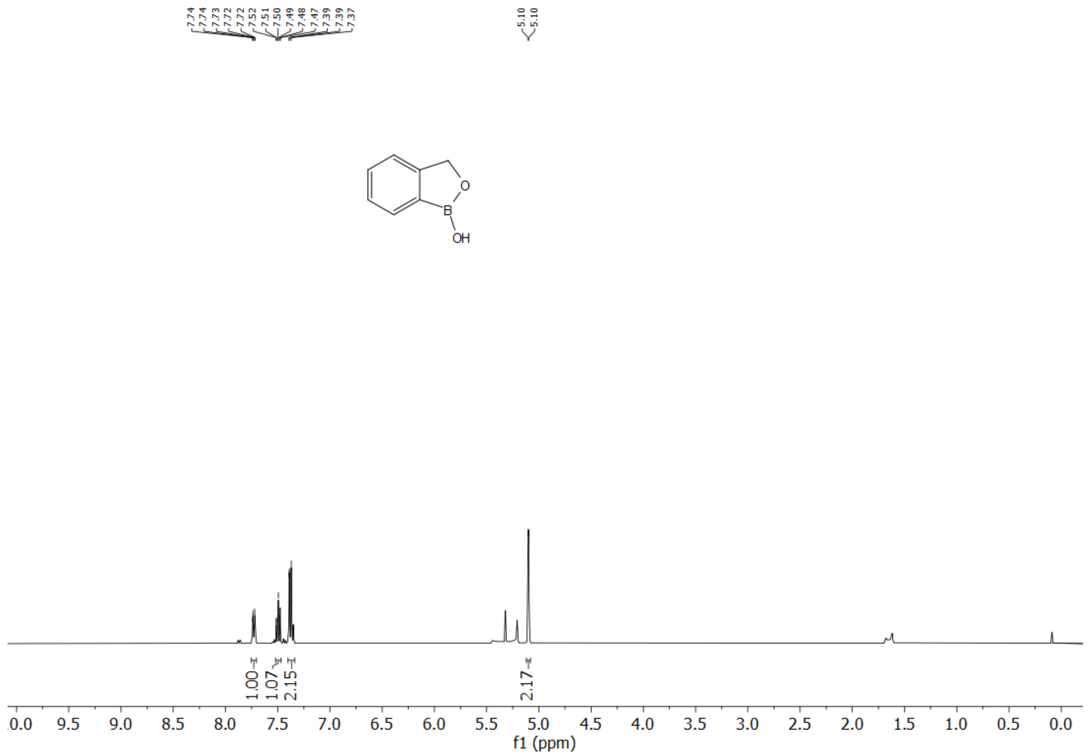

Figure S23: ^{1}H NMR (400 MHz, CD_2Cl_2, 298 K) spectrum of Benzo[*c*][1,2]oxaborol-1(3H)-ol (5a).

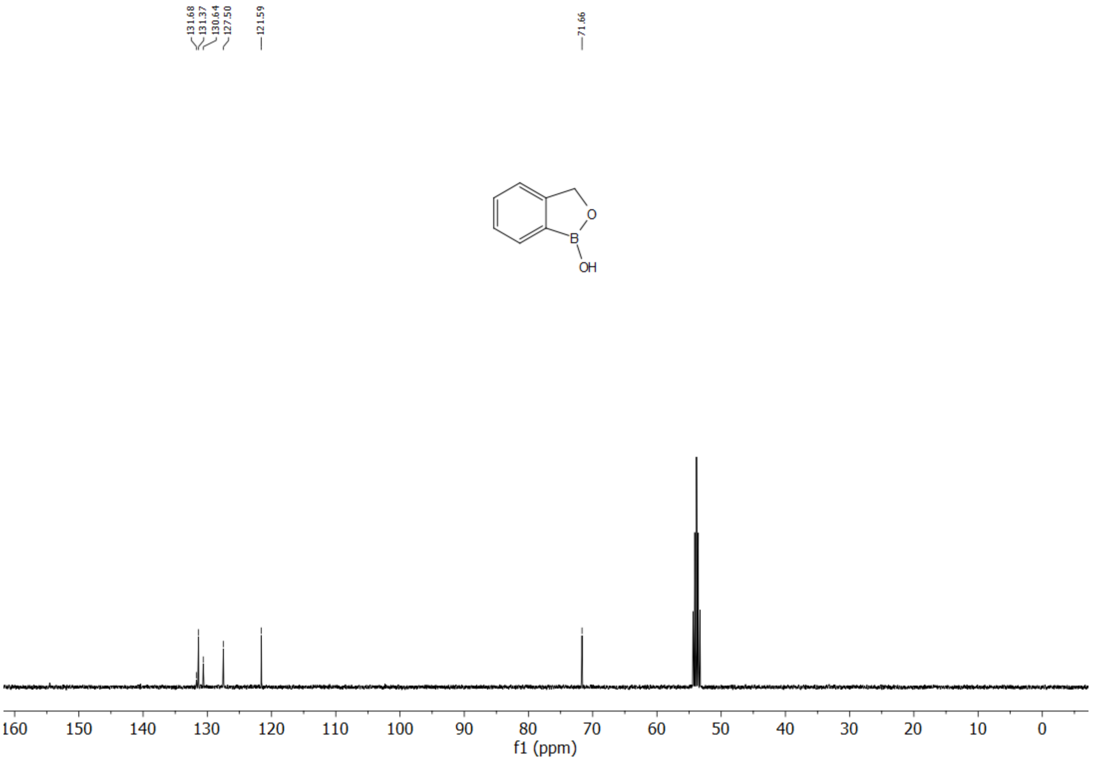

Figure S24: ^{13}C{^{1}H} NMR (101 MHz, CD_2Cl_2, 298 K) spectrum of Benzo[*c*][1,2]oxaborol-1(3H)-ol (5a).

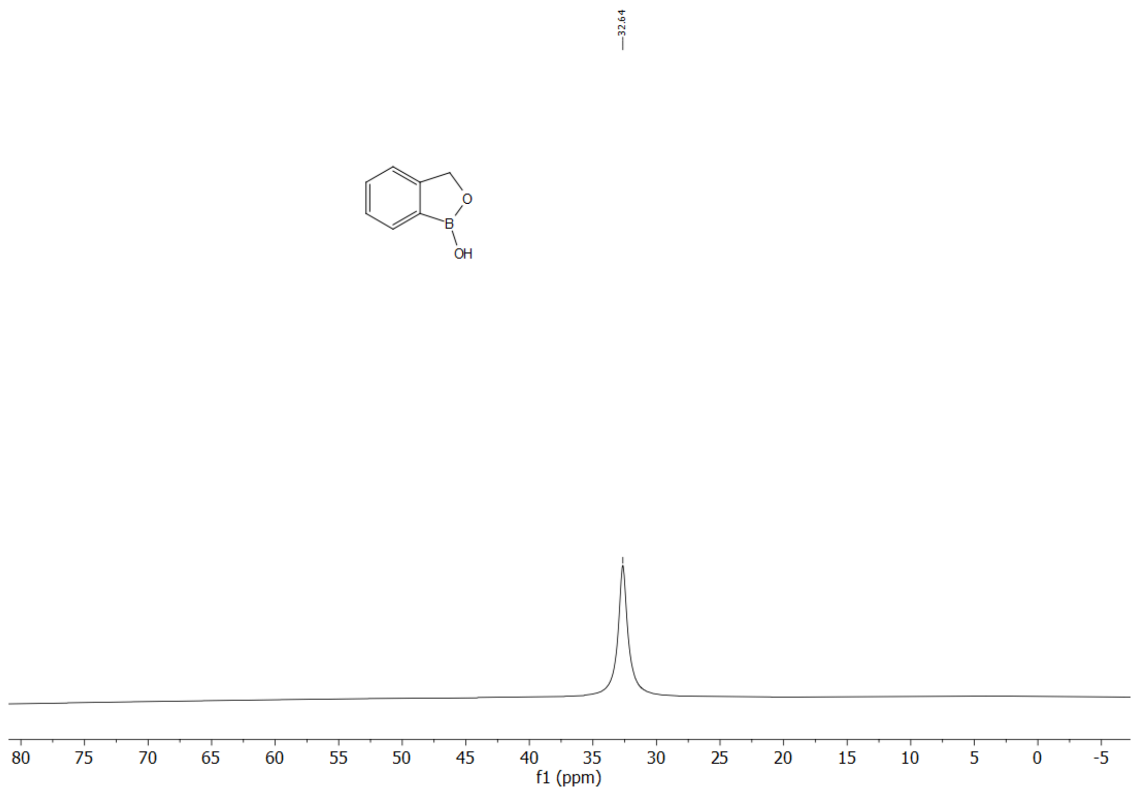

Figure S25: $^{11}B\{^{1}H\}$ NMR (128 MHz, CD_2Cl_2, 298 K) spectrum of Benzo[*c*][1,2]oxaborol-1(3H)-ol (5a).

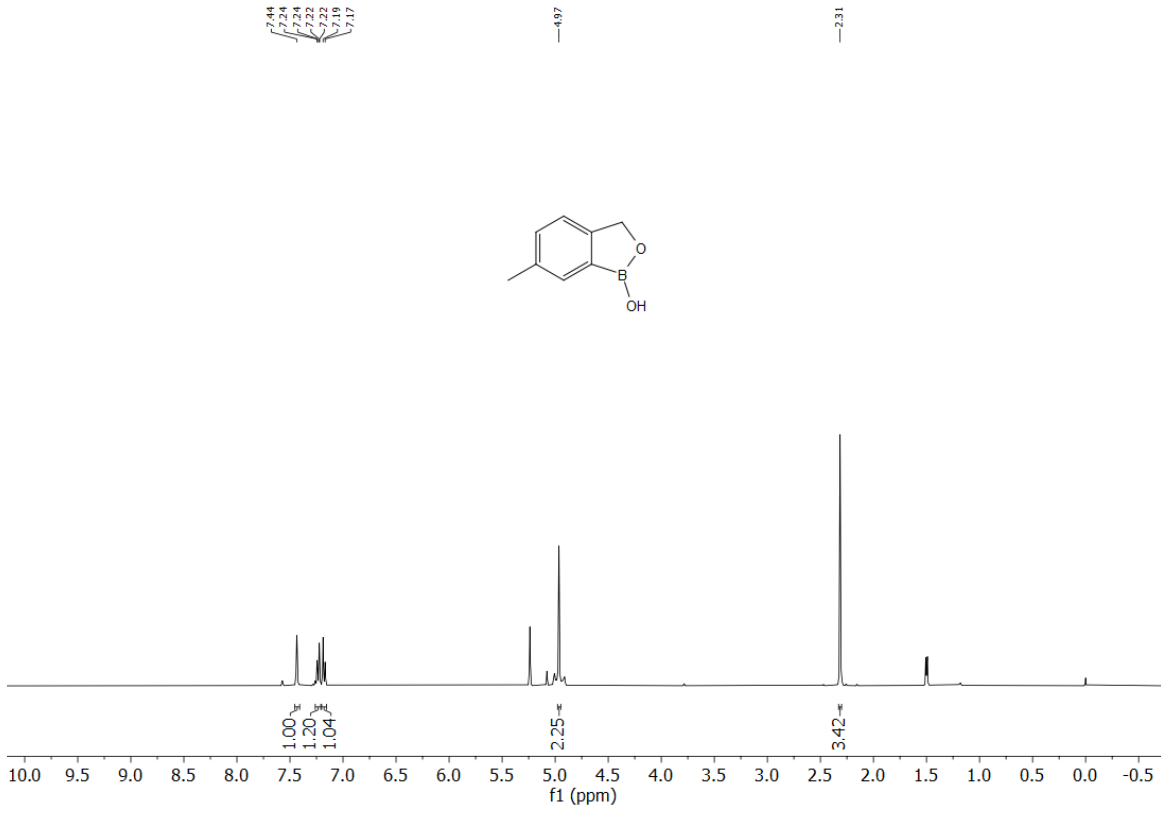

Figure S26: ^{1}H NMR (400 MHz, CD_2Cl_2, 298 K) spectrum of 6-methylbenzo[*c*][1,2]oxaborol-1(3*H*)-ol (5e).

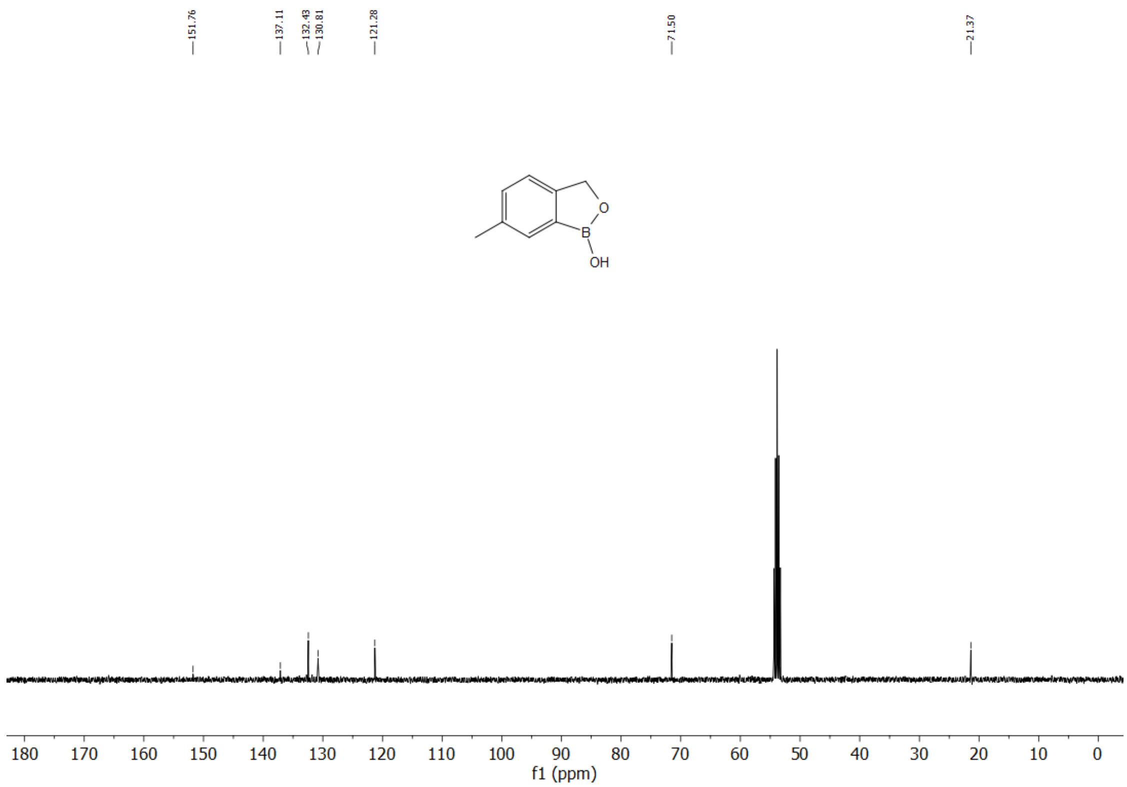

Figure S27: $^{13}C\{^{1}H\}$ NMR (101 MHz, CD_2Cl_2, 298 K) spectrum of 6-methylbenzo[*c*][1,2]oxaborol-1(3*H*)-ol (5e).

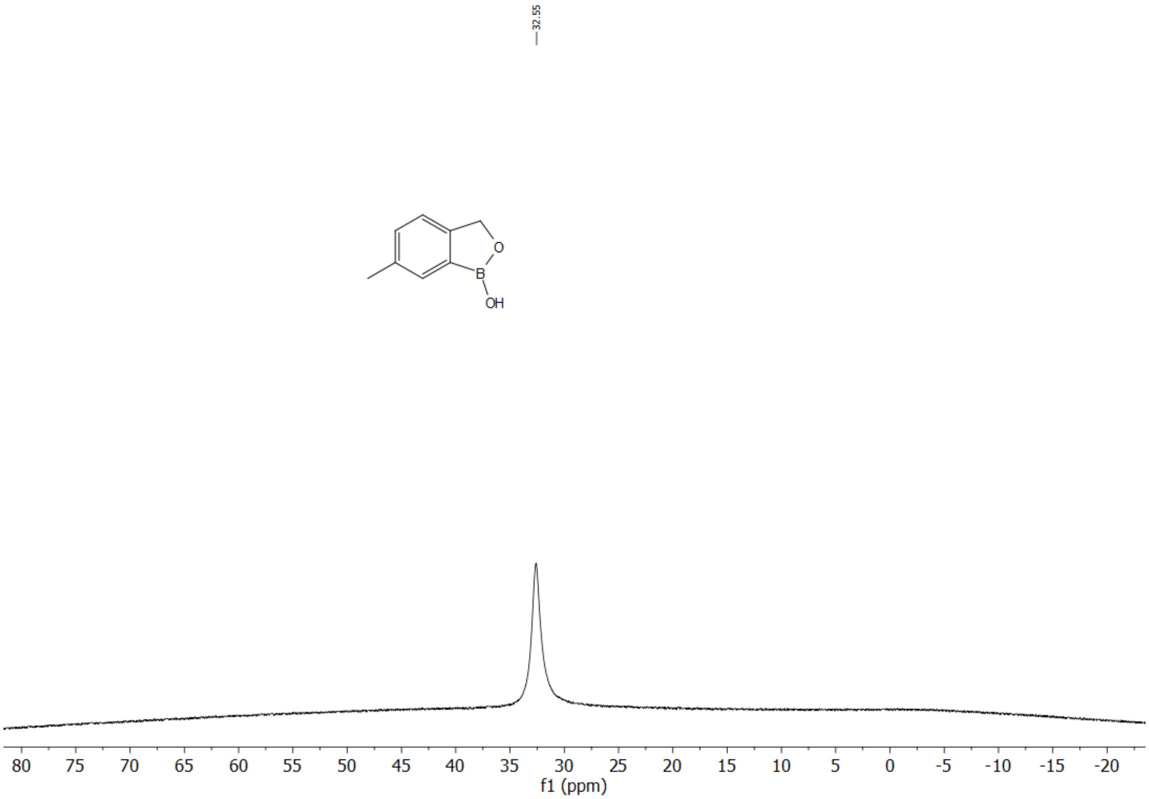

Figure S28: $^{11}B\{^{1}H\}$ NMR (128 MHz, CD_2Cl_2, 298 K) spectrum of 6-methylbenzo[*c*][1,2]oxaborol-1(3*H*)-ol (5e).

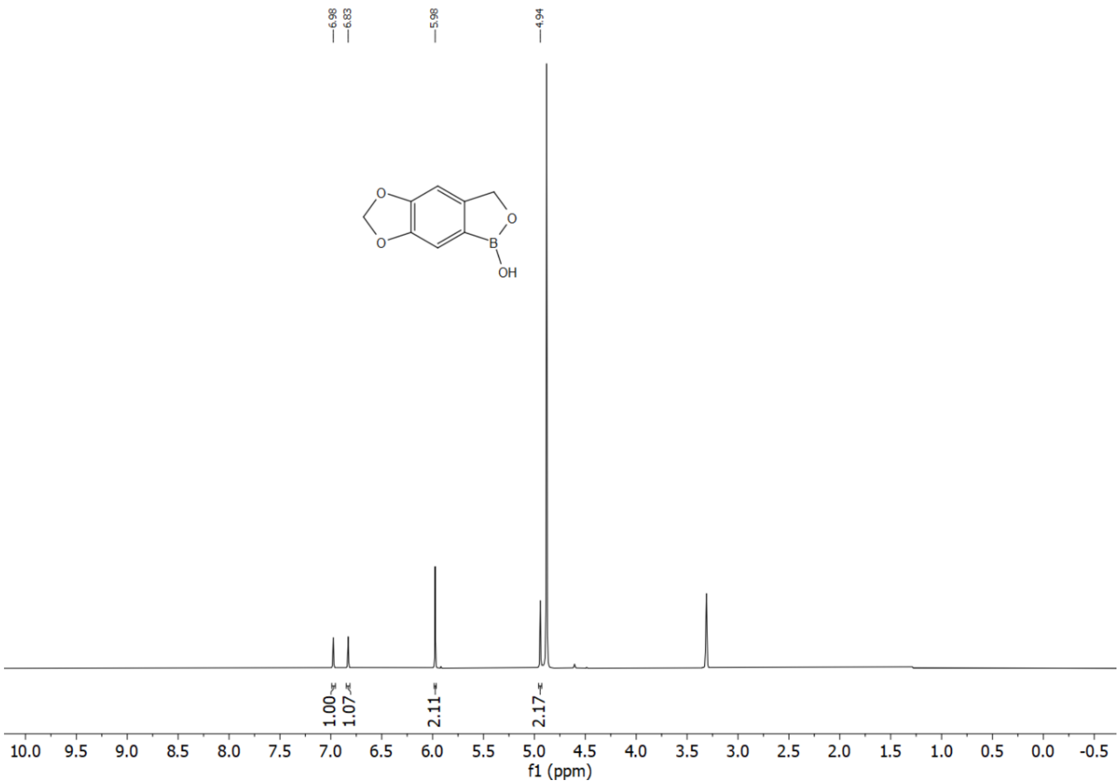

Figure S29: ^{1}H NMR (400 MHz, CD_3OD, 298 K) spectrum of [1,3]dioxolo[4',5':4,5]benzo[1,2-*c*][1,2]oxaborol-1(3*H*)-ol (5i)

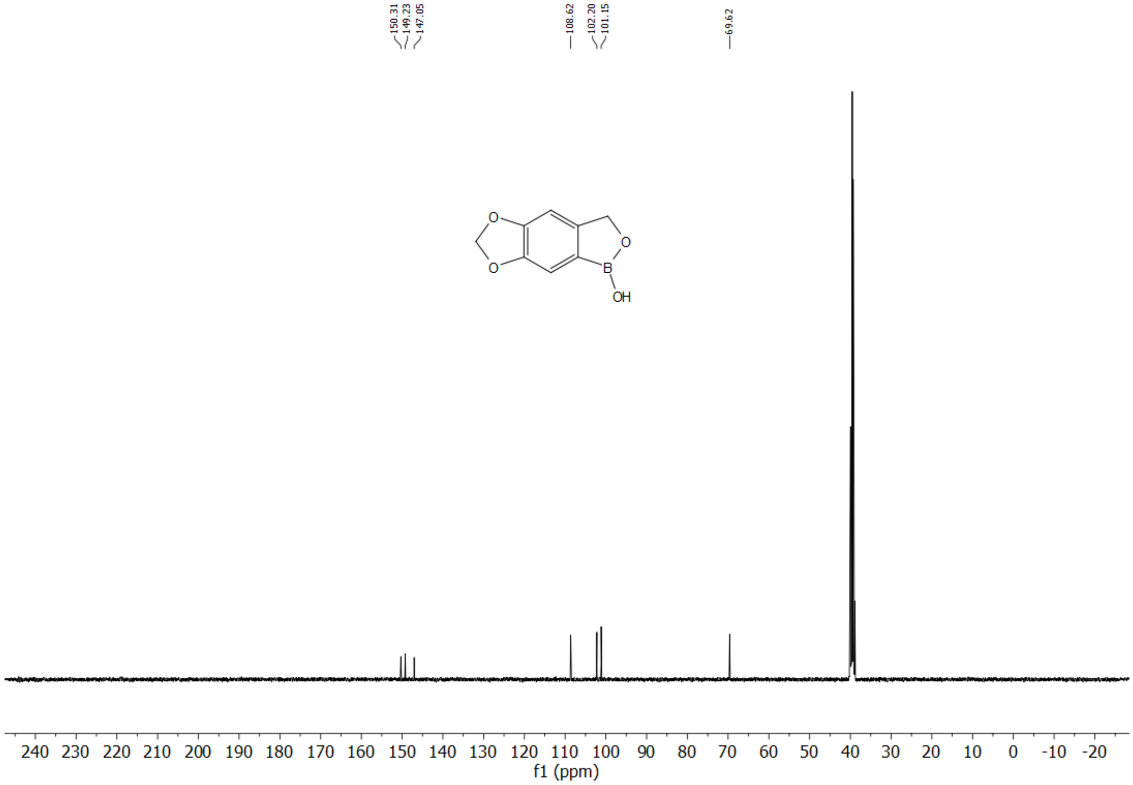

Figure S30: ^{13}C{^{1}H} NMR (101 MHz, DMSO-d6, 298 K) spectrum of [1,3]dioxolo[4',5':4,5]benzo[1,2-*c*][1,2]oxaborol-1(3*H*)-ol (5i)

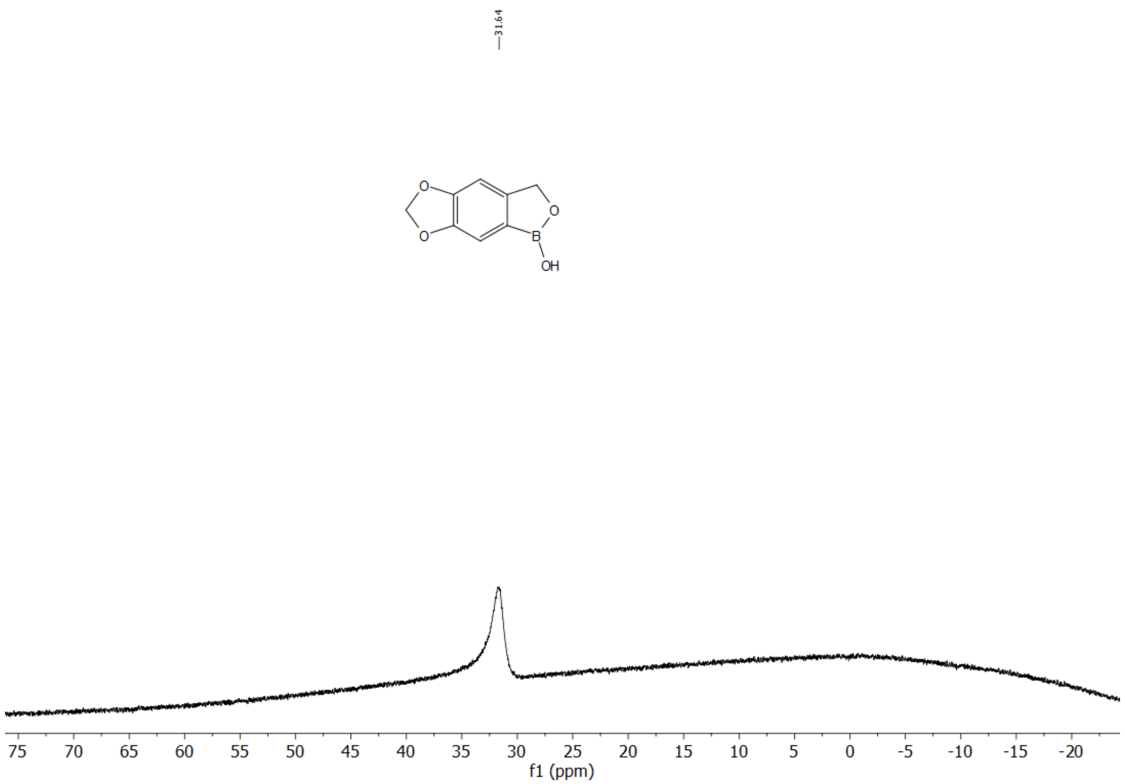

Figure S31: $^{11}B\{^{1}H\}$ NMR (128 MHz, CD_3OD, 298 K) spectrum of [1,3]dioxolo[4',5':4,5]benzo[1,2-*c*][1,2]oxaborol-1(3*H*)-ol (5i)

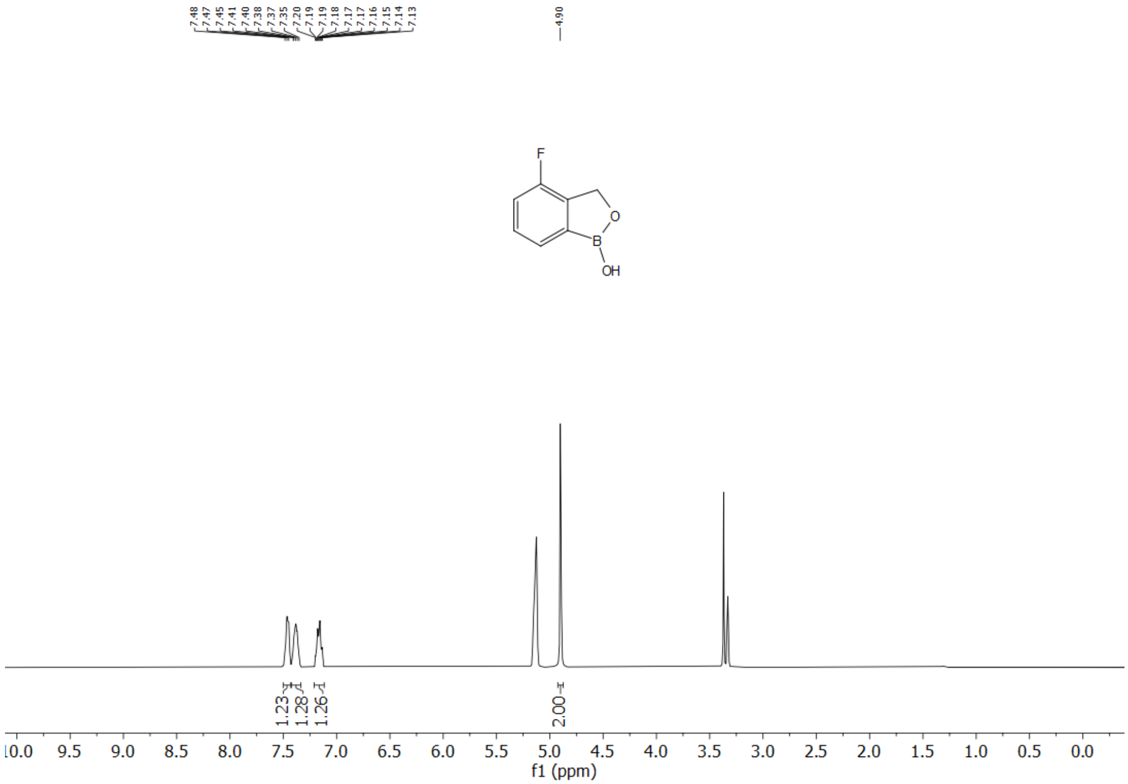

Figure S32: ^{1}H NMR (400 MHz, CD_3OD, 298 K) spectrum of 4-fluorobenzo[*c*][1,2]oxaborol-1(3*H*)-ol (5t).

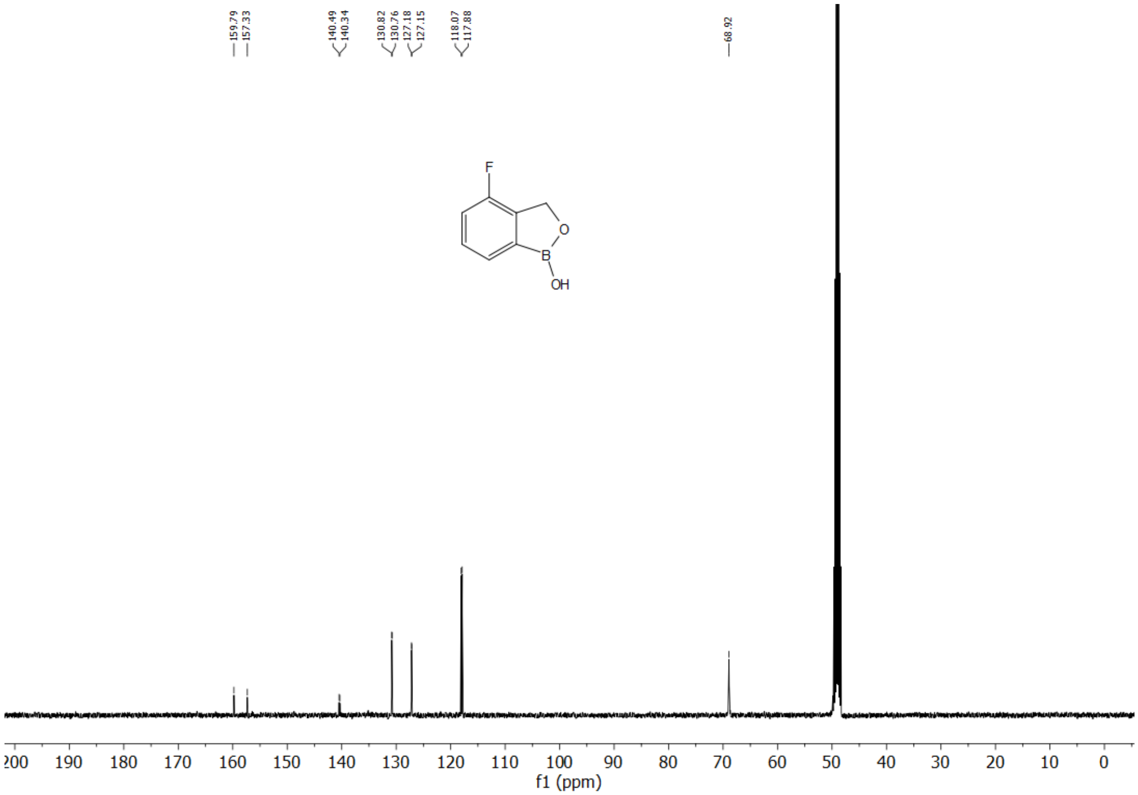

Figure S33: $^{13}C\{^1H\}$ NMR (101 MHz, CD_3OD, 298 K) spectrum of 4-fluorobenzo[*c*][1,2]oxaborol-1(3*H*)-ol (5t).

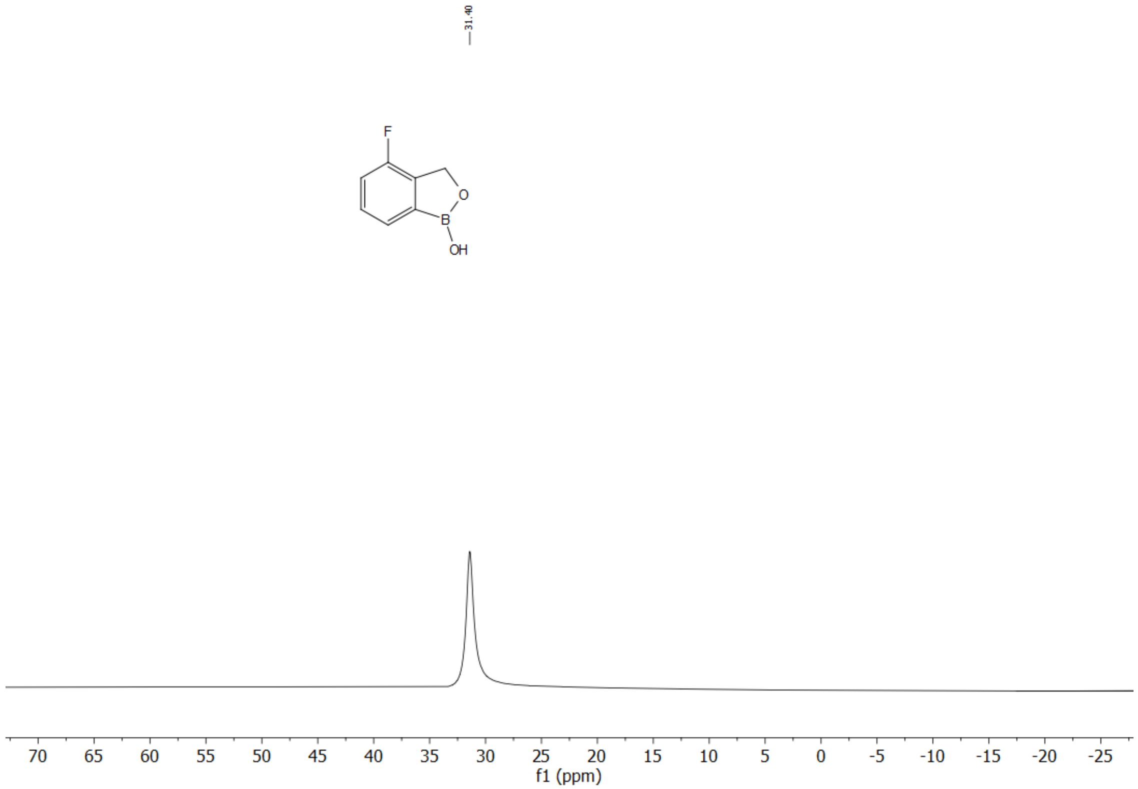

Figure S34: $^{11}B\{^1H\}$ NMR (128 MHz, CD_3OD, 298 K) spectrum of 4-fluorobenzo[*c*][1,2]oxaborol-1(3*H*)-ol (5t).

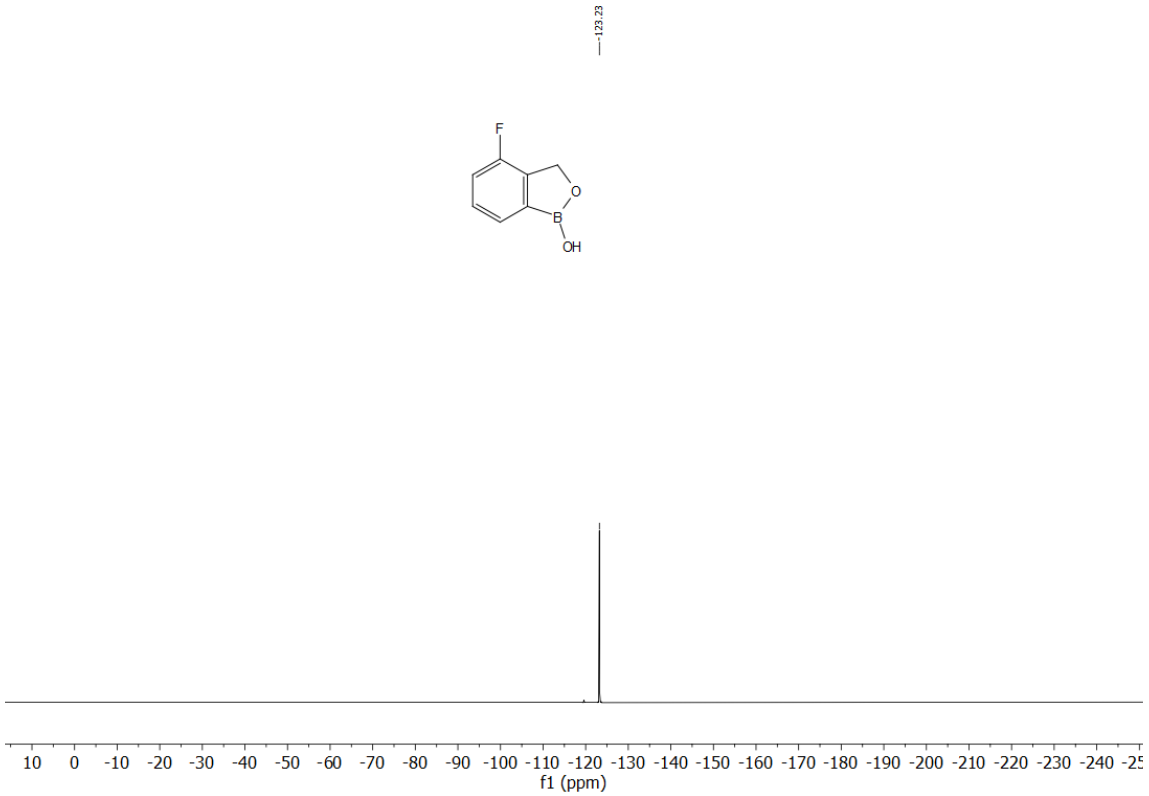

Figure S35: $^{19}F\{^{1}H\}$ NMR (376 MHz, CD_3OD, 298 K) spectrum of 4-fluorobenzo[*c*][1,2]oxaborol-1(3*H*)-ol (5t).

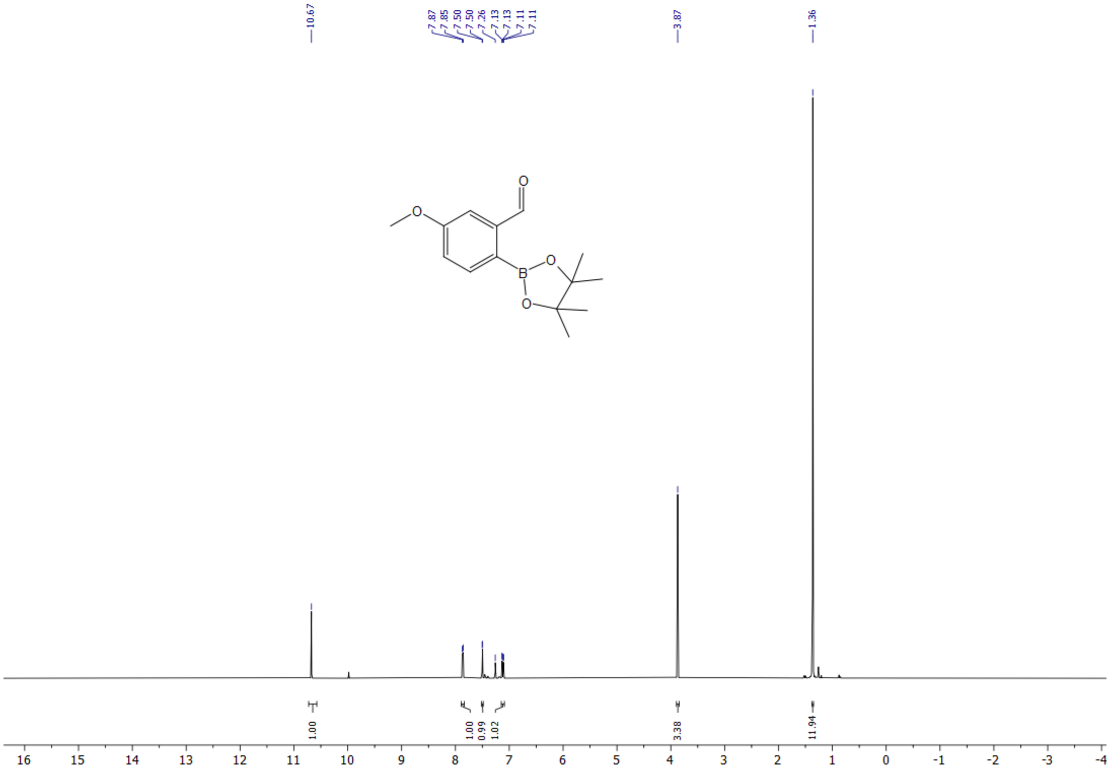

Figure S36: ^{1}H NMR (400 MHz, $CDCl_3$, 298 K) spectrum of 5-methoxy-2-(4,4,5,5-tetramethyl-1,3,2-dioxaborolan-2-yl)benzaldehyde.

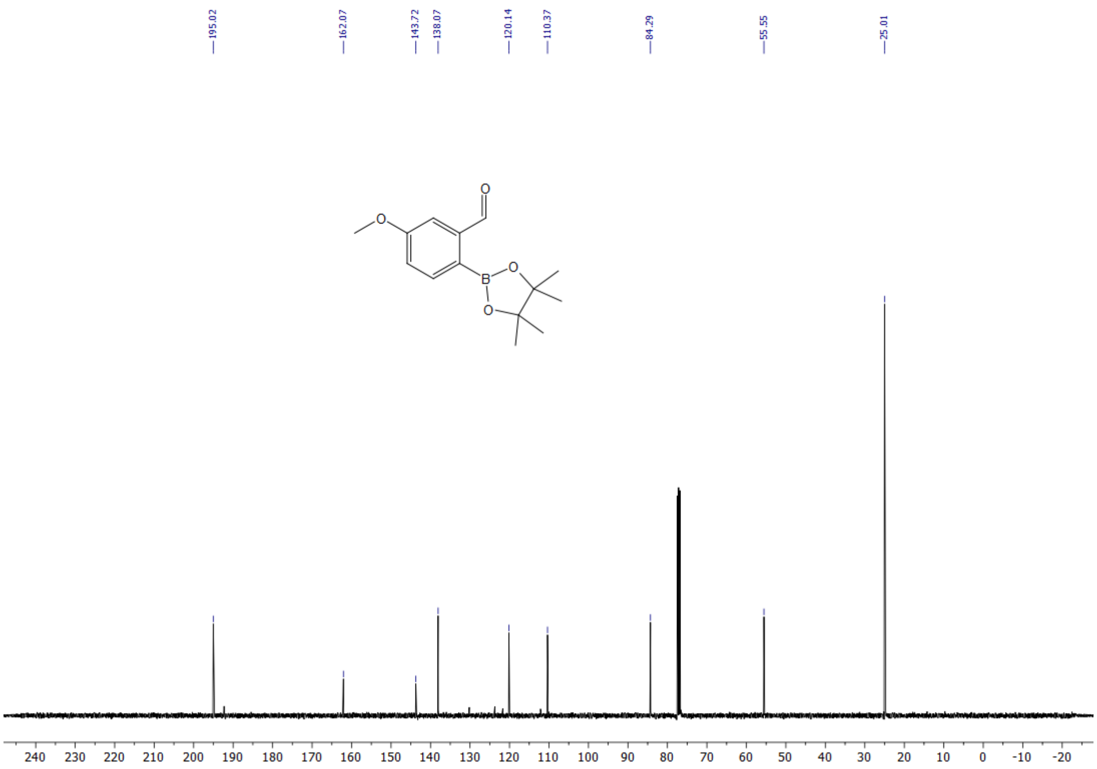

Figure S37: $^{13}C\{^1H\}$ NMR (101 MHz, $CDCl_3$, 298 K) spectrum of 5-methoxy-2-(4,4,5,5-tetramethyl-1,3,2-dioxaborolan-2-yl)benzaldehyde.

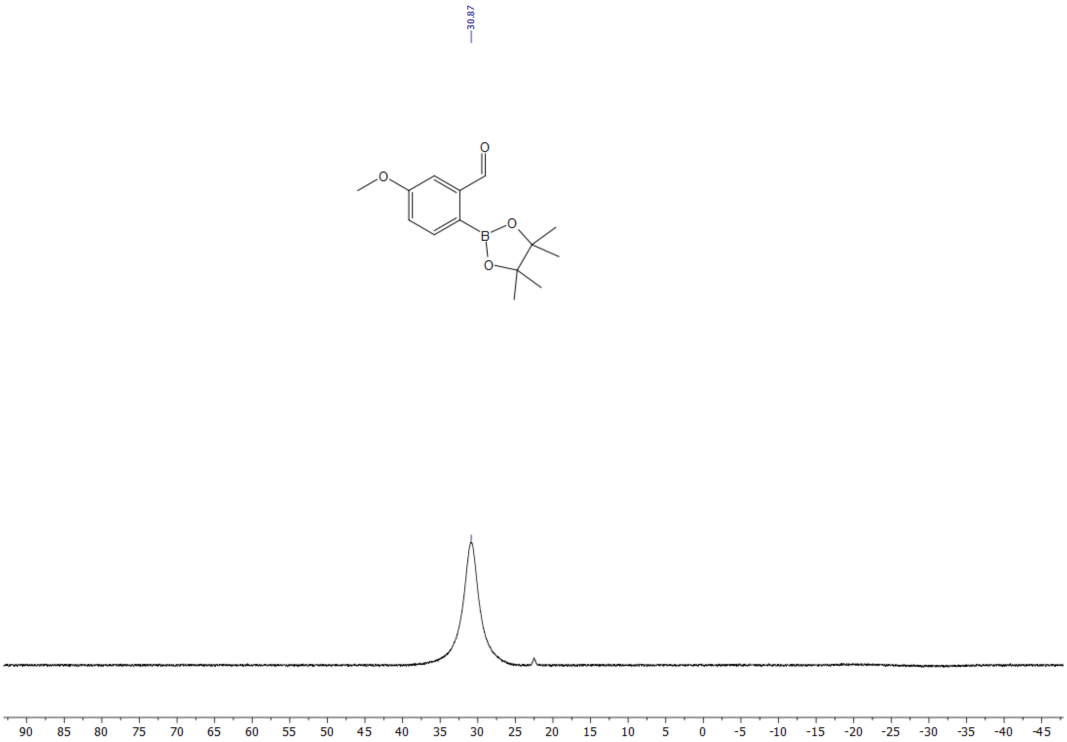

Figure S38: $^{11}B\{^1H\}$ NMR (128 MHz, CD_3OD, 298 K) spectrum of 5-methoxy-2-(4,4,5,5-tetramethyl-1,3,2-dioxaborolan-2-yl)benzaldehyde.

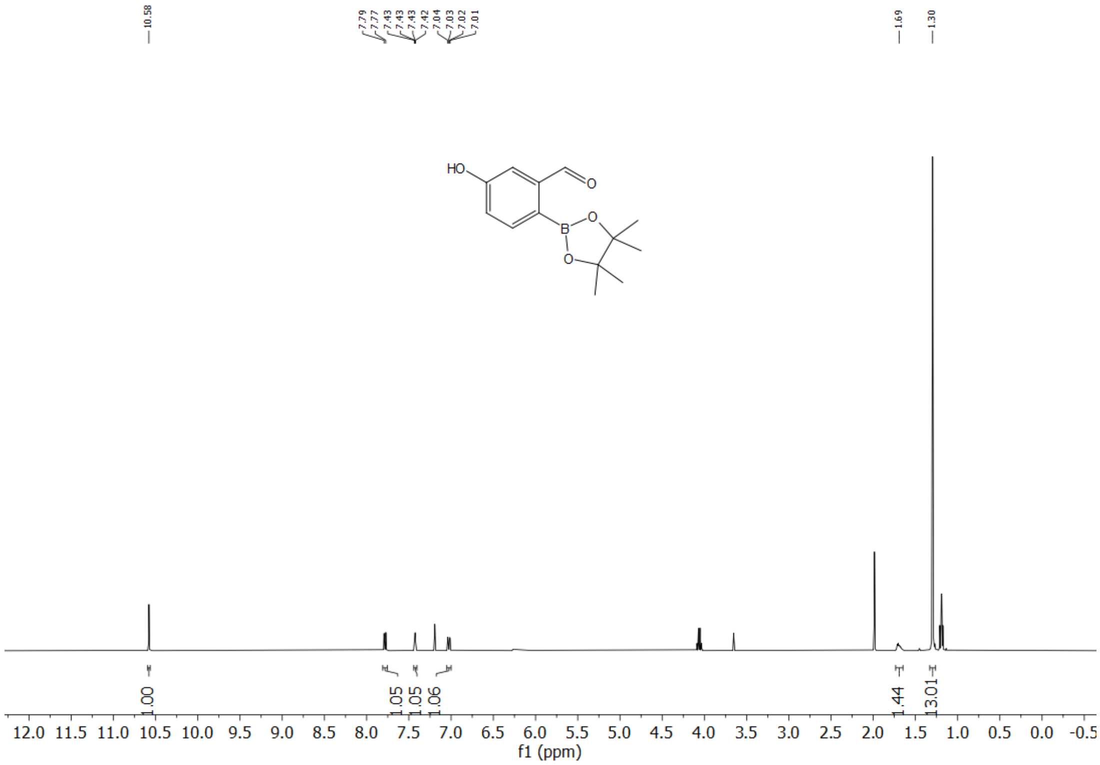

Figure S39: ^{1}H NMR (400 MHz, $CDCl_3$, 298 K) spectrum of 5-hydroxy-2-(4,4,5,5-tetramethyl-1,3,2-dioxaborolan-2-yl)benzaldehyde.

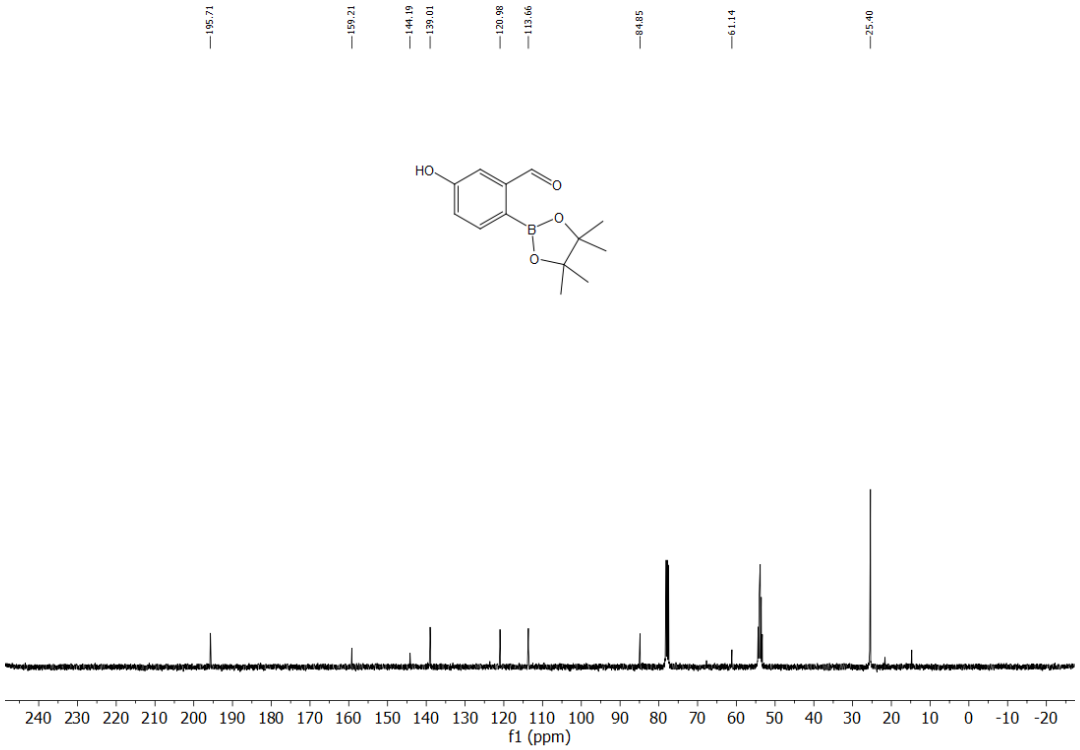

Figure S40: $^{13}C\{^{1}H\}$ NMR (101 MHz, $CDCl_3$, 298 K) spectrum of 5-hydroxy-2-(4,4,5,5-tetramethyl-1,3,2-dioxaborolan-2-yl)benzaldehyde.

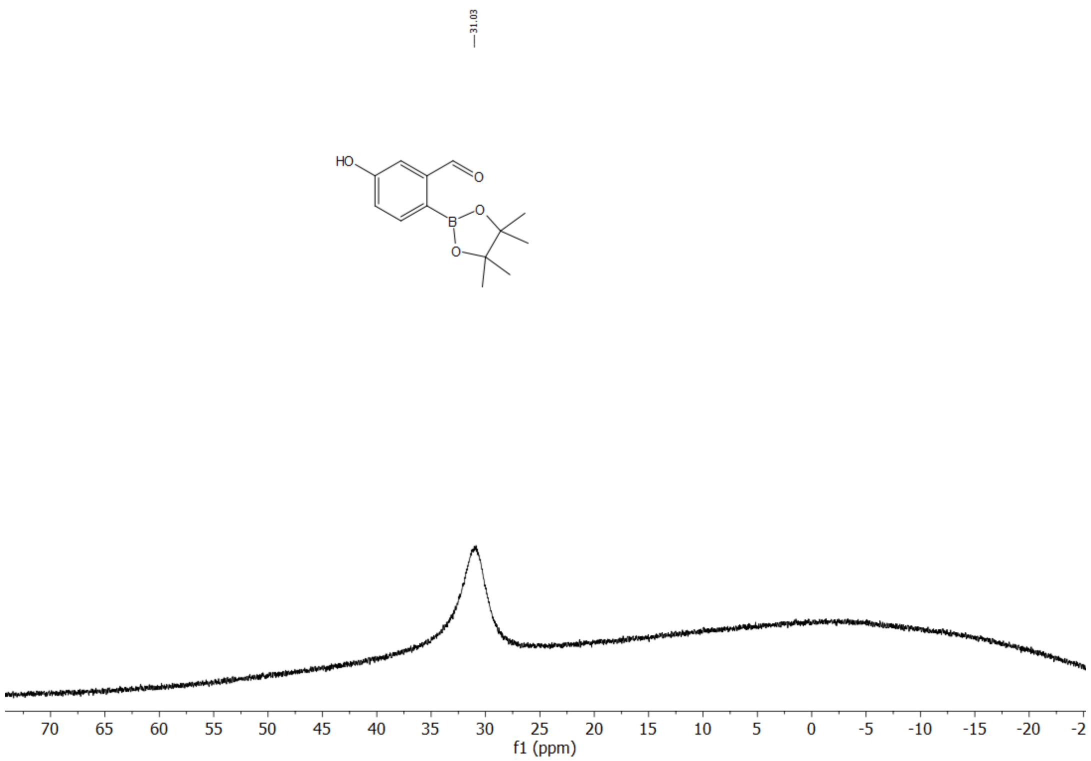

Figure S41: $^{11}B\{^{1}H\}$ NMR (128 MHz, $CDCl_3$, 298 K) spectrum of 5-hydroxy-2-(4,4,5,5-tetramethyl-1,3,2-dioxaborolan-2-yl)benzaldehyde.

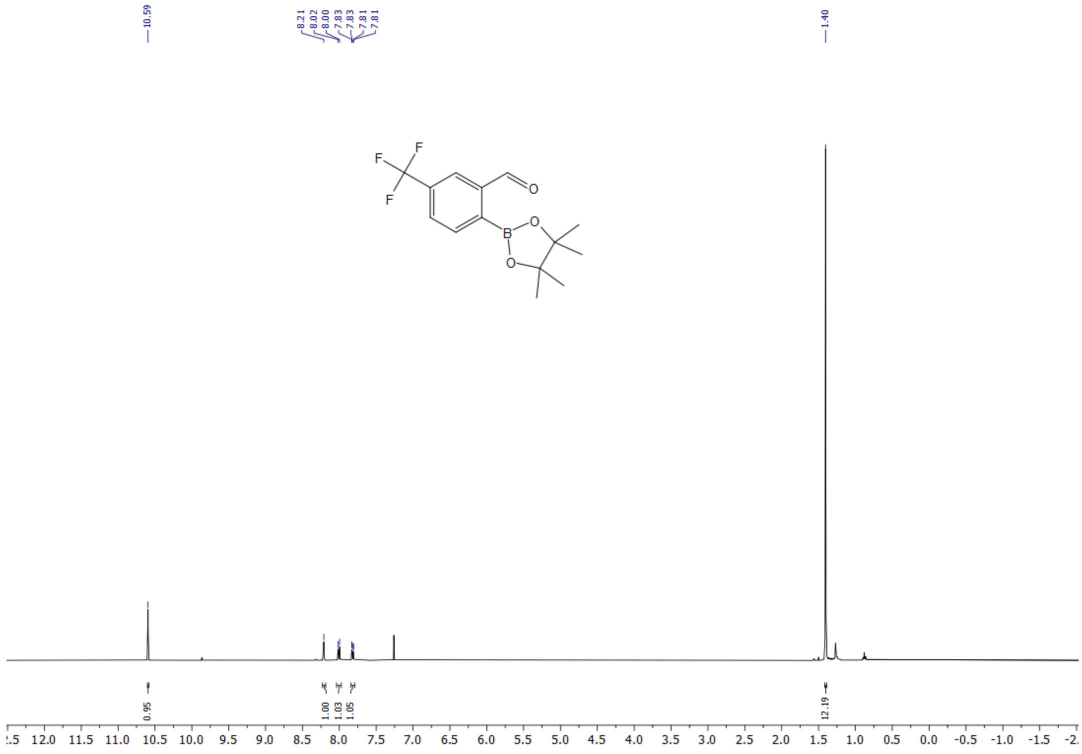

Figure S42: ^{1}H NMR (400 MHz, $CDCl_3$, 298 K) spectrum of 5-(trifluoromethyl)-2-(4,4,5,5-tetramethyl-1,3,2-dioxaborolan-2-yl)benzaldehyde.

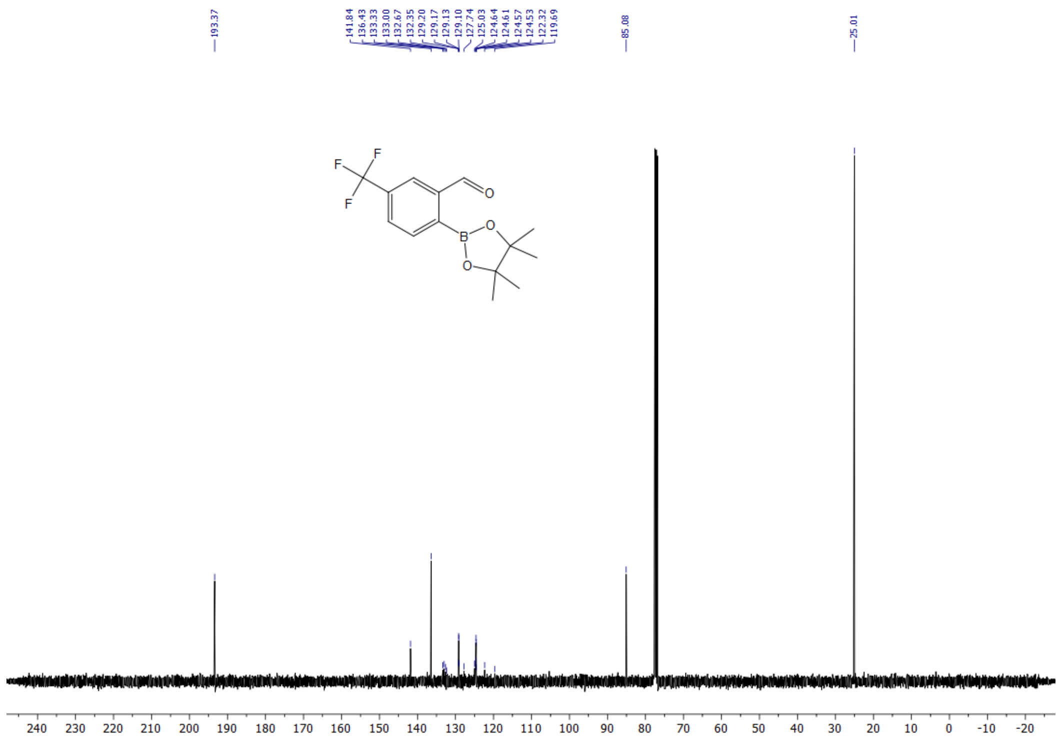

Figure S43: $^{13}C\{^1H\}$ NMR (101 MHz, $CDCl_3$, 298 K) spectrum of 5-(trifluoromethyl)-2-(4,4,5,5-tetramethyl-1,3,2-dioxaborolan-2-yl)benzaldehyde.

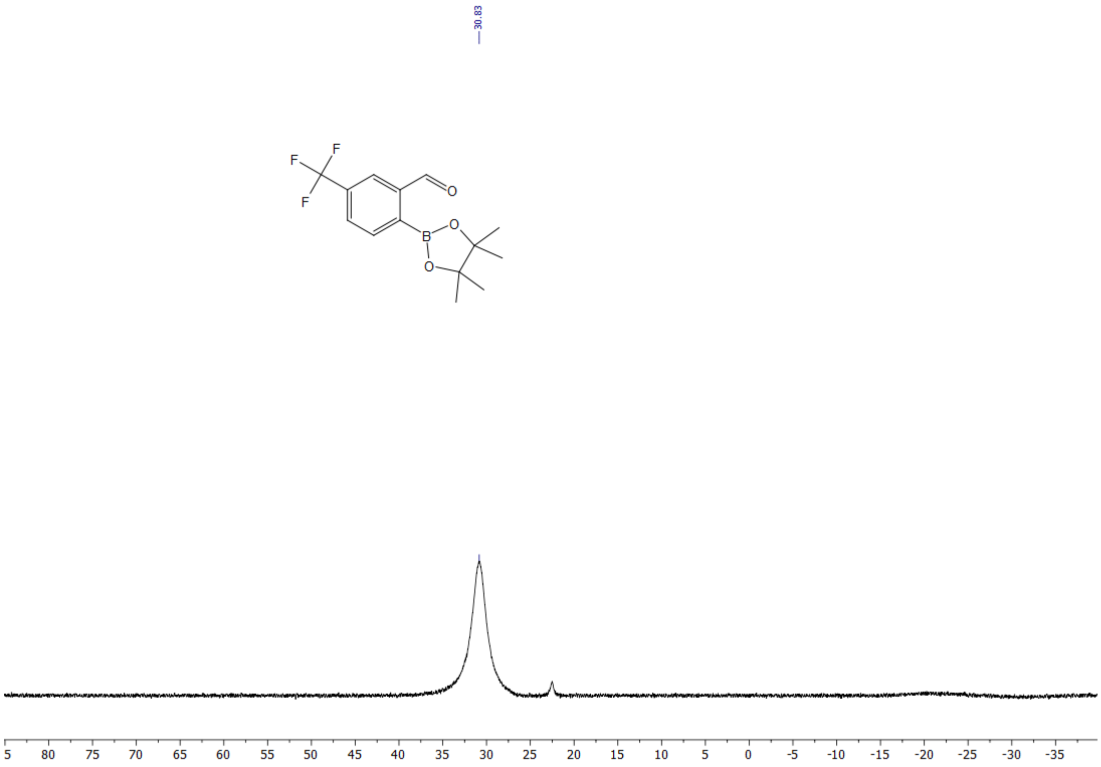

Figure S44: $^{11}B\{^1H\}$ NMR (128 MHz, $CDCl_3$, 298 K) spectrum of 5-(trifluoromethyl)-2-(4,4,5,5-tetramethyl-1,3,2-dioxaborolan-2-yl)benzaldehyde.

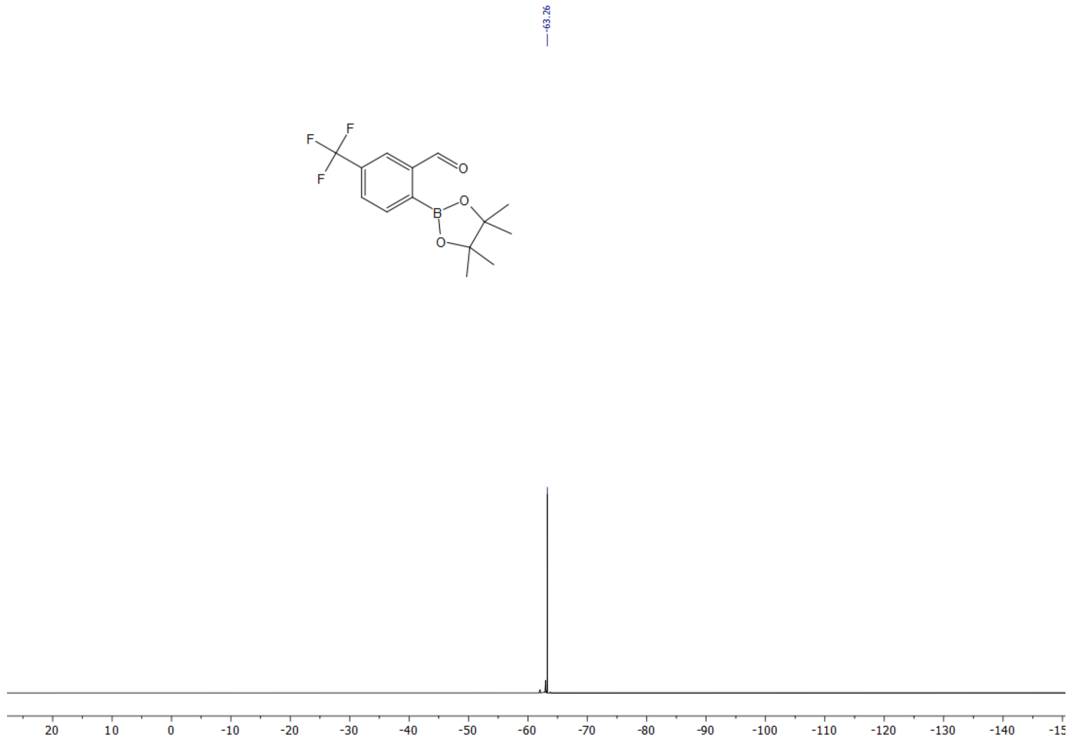

Figure S45: $^{19}F\{^1H\}$ NMR (376 MHz, $CDCl_3$, 298 K) 5-(trifluoromethyl)-2-(4,4,5,5-tetramethyl-1,3,2-dioxaborolan-2-yl)benzaldehyde.

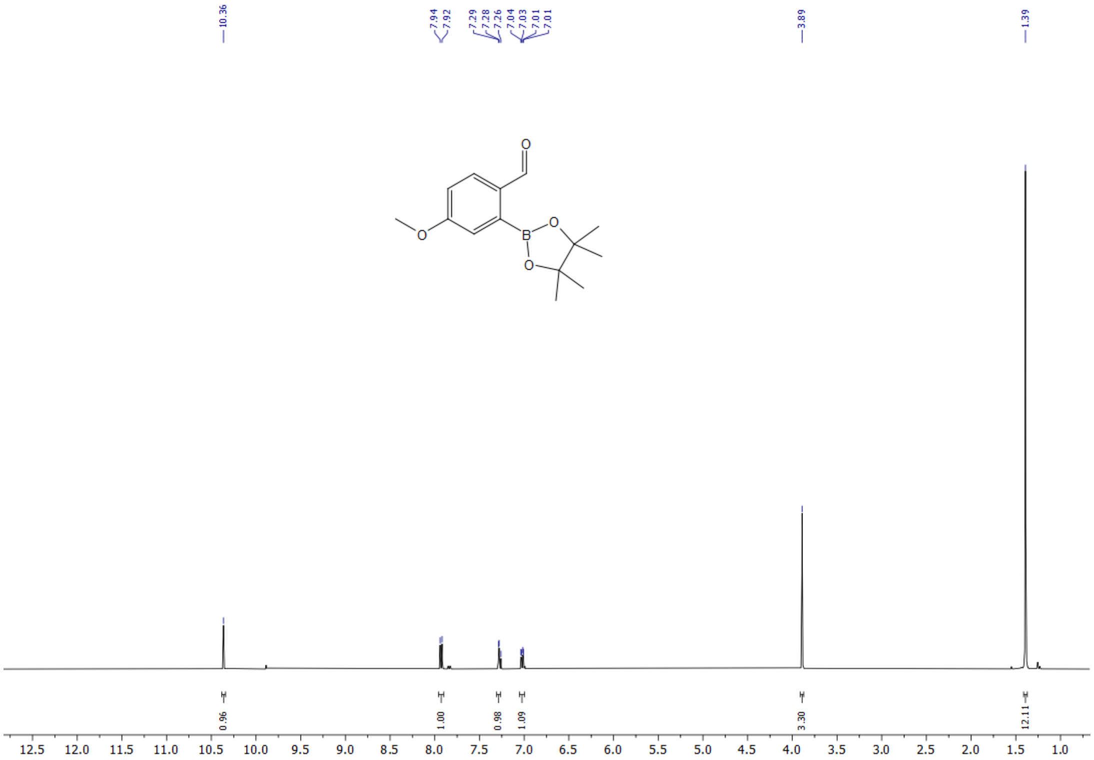

Figure S46: ^1H NMR (400 MHz, $CDCl_3$, 298 K) spectrum of 4-methoxy-2-(4,4,5,5-tetramethyl-1,3,2-dioxaborolan-2-yl)benzaldehyde.

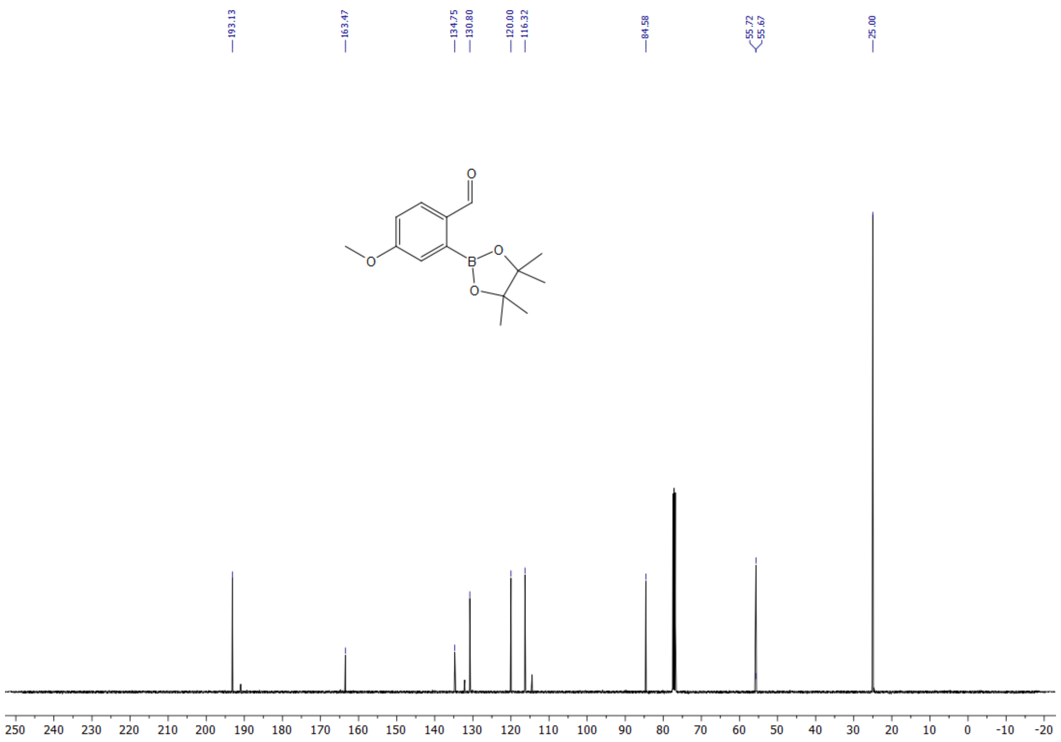

Figure S47: $^{13}C\{^1H\}$ NMR (101 MHz, $CDCl_3$, 298 K) spectrum of 4-methoxy-2-(4,4,5,5-tetramethyl-1,3,2-dioxaborolan-2-yl)benzaldehyde.

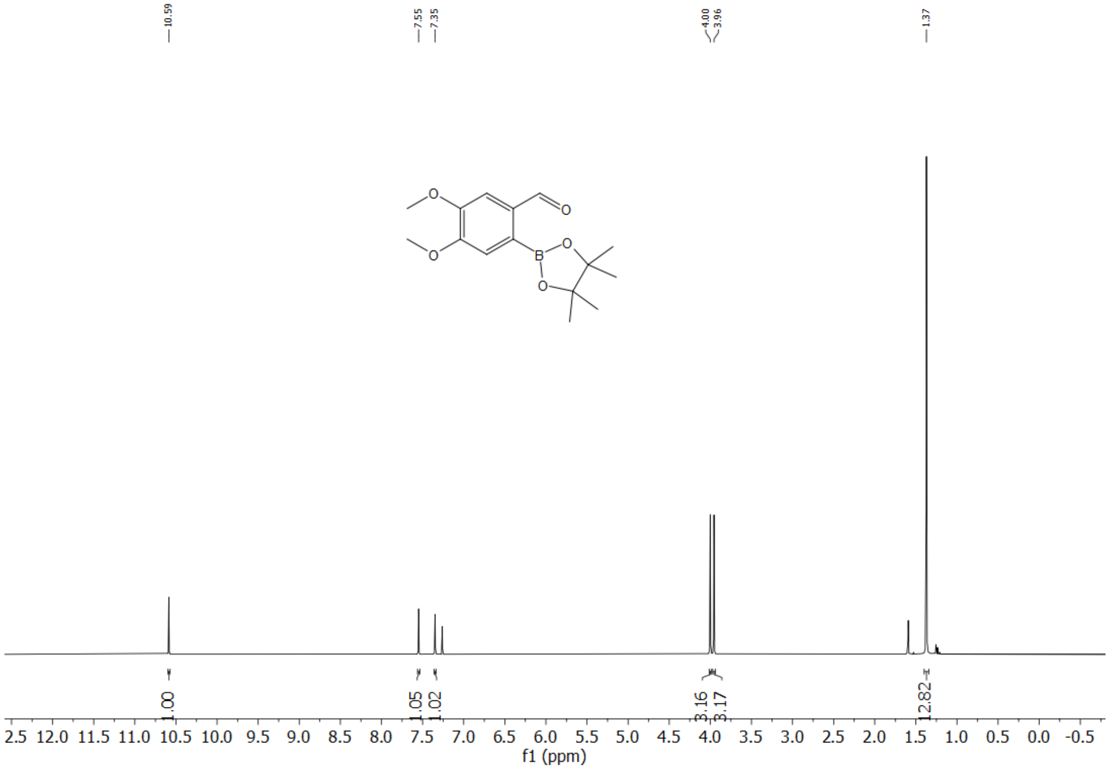

Figure S48: ^1H NMR (400 MHz, $CDCl_3$, 298 K) spectrum of 4,5-dimethoxy-2-(4,4,5,5-tetramethyl-1,3,2-dioxaborolan-2-yl)benzaldehyde.

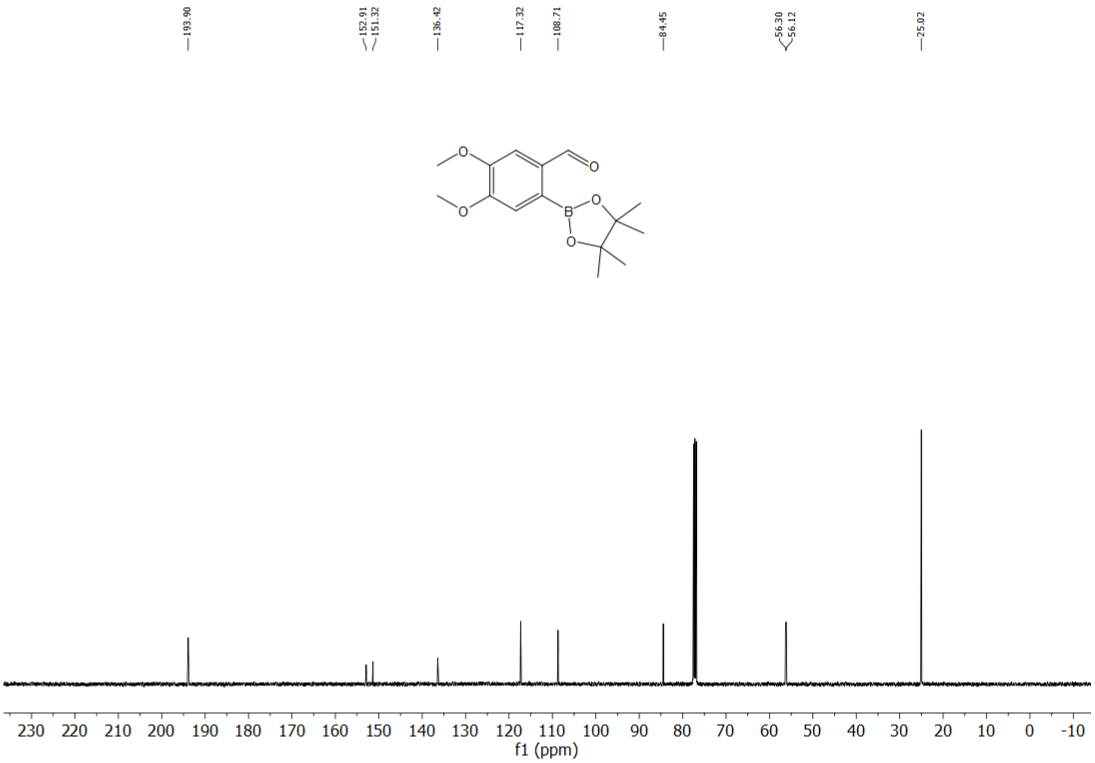

Figure S49: $^{13}C\{^{1}H\}$ NMR (101 MHz, $CDCl_3$, 298 K) spectrum of 4,5-dimethoxy-2-(4,4,5,5-tetramethyl-1,3,2-dioxaborolan-2-yl)benzaldehyde.

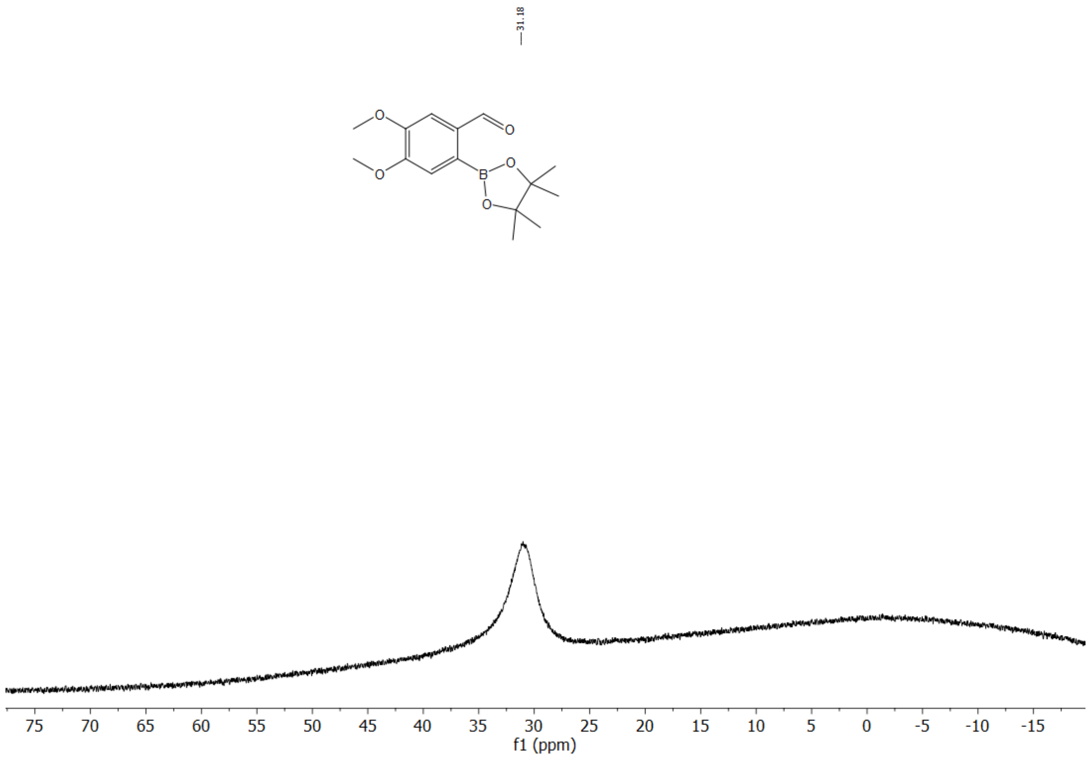

Figure S50: $^{11}B\{^{1}H\}$ NMR (128 MHz, $CDCl_3$, 298 K) spectrum of 4,5-dimethoxy-2-(4,4,5,5-tetramethyl-1,3,2-dioxaborolan-2-yl)benzaldehyde.

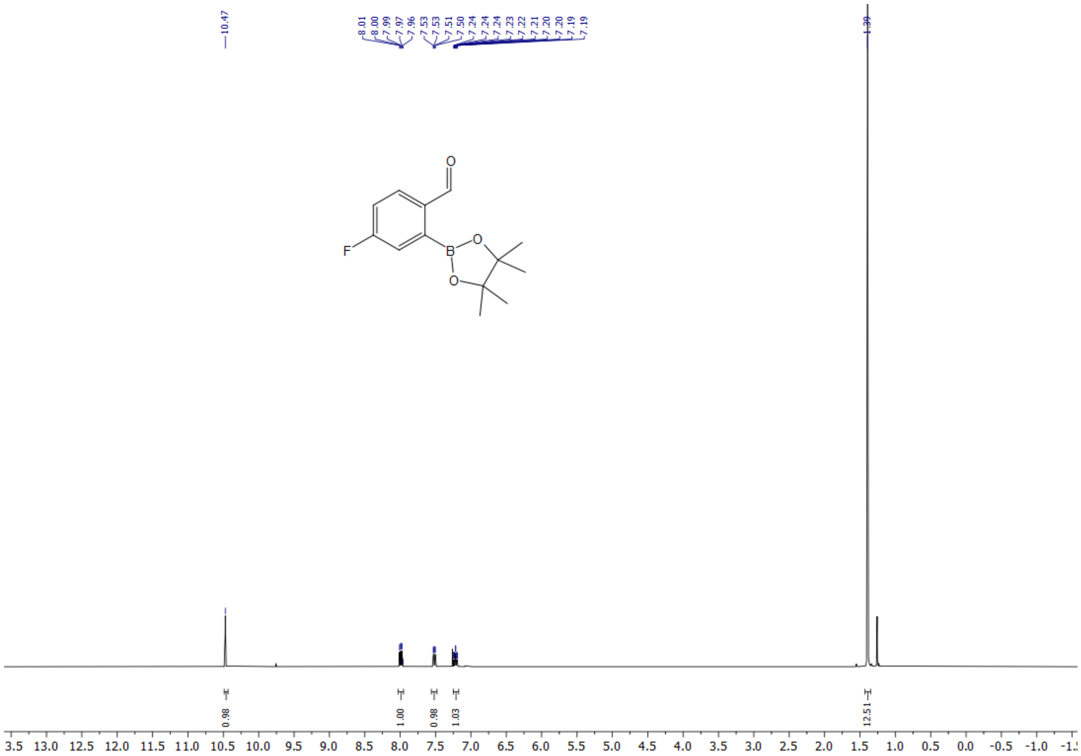

Figure S51: ^{1}H NMR (400 MHz, $CDCl_3$, 298 K) spectrum of 4-fluoro-2-(4,4,5,5-tetramethyl-1,3,2-dioxaborolan-2-yl)benzaldehyde.

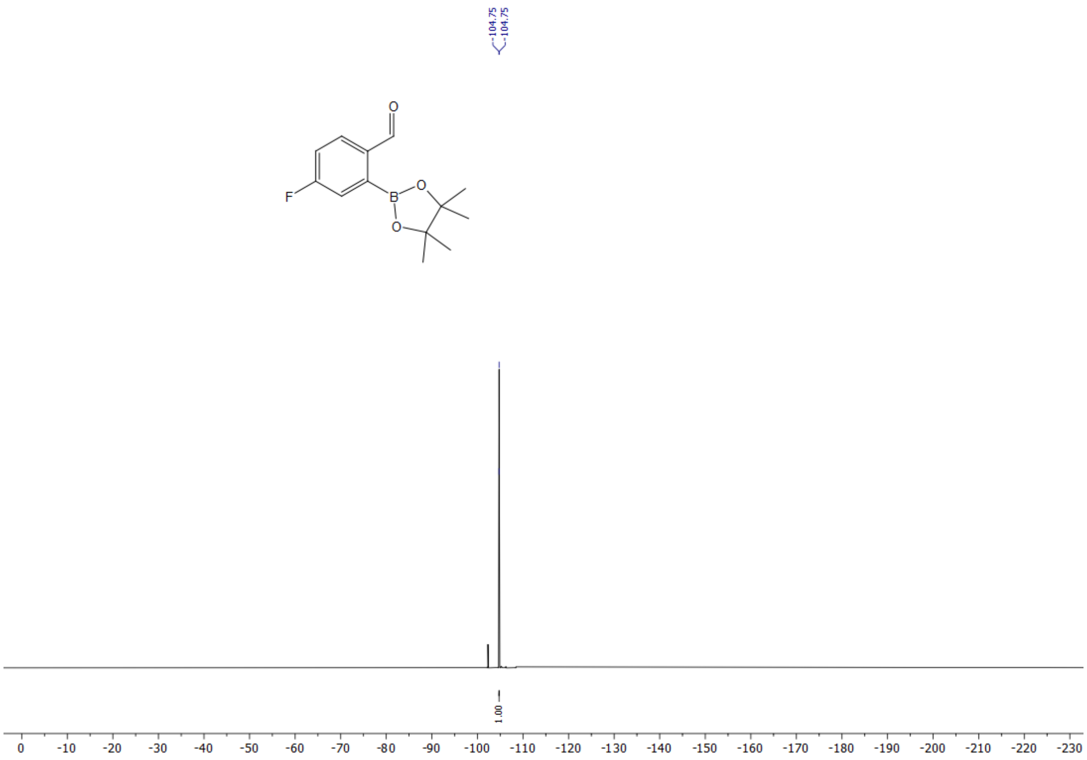

Figure S52: ^{19}F{^{1}H}-NMR (376 MHz, $CDCl_3$, 298 K) spectrum of 4-fluoro-2-(4,4,5,5-tetramethyl-1,3,2-dioxaborolan-2-yl)benzaldehyde.

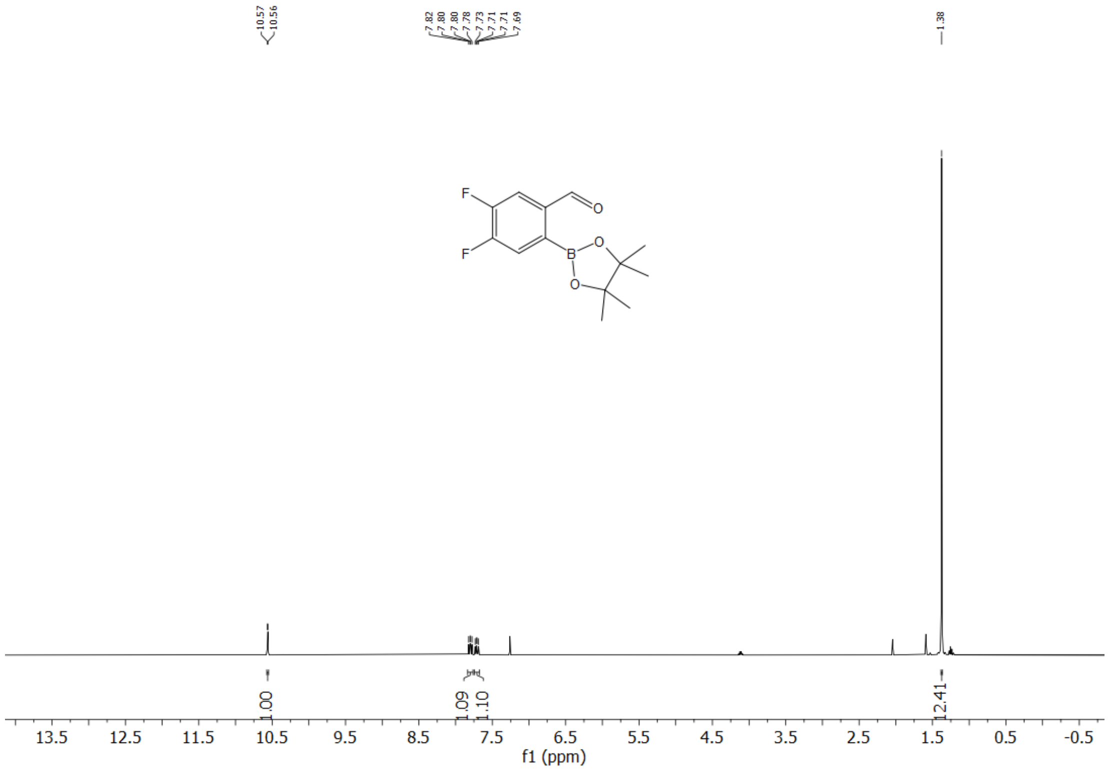

Figure S53: ^{1}H NMR (400 MHz, $CDCl_3$, 298 K) spectrum of 4,5-difluoro-2-(4,4,5,5-tetramethyl-1,3,2-dioxaborolan-2-yl)benzaldehyde.

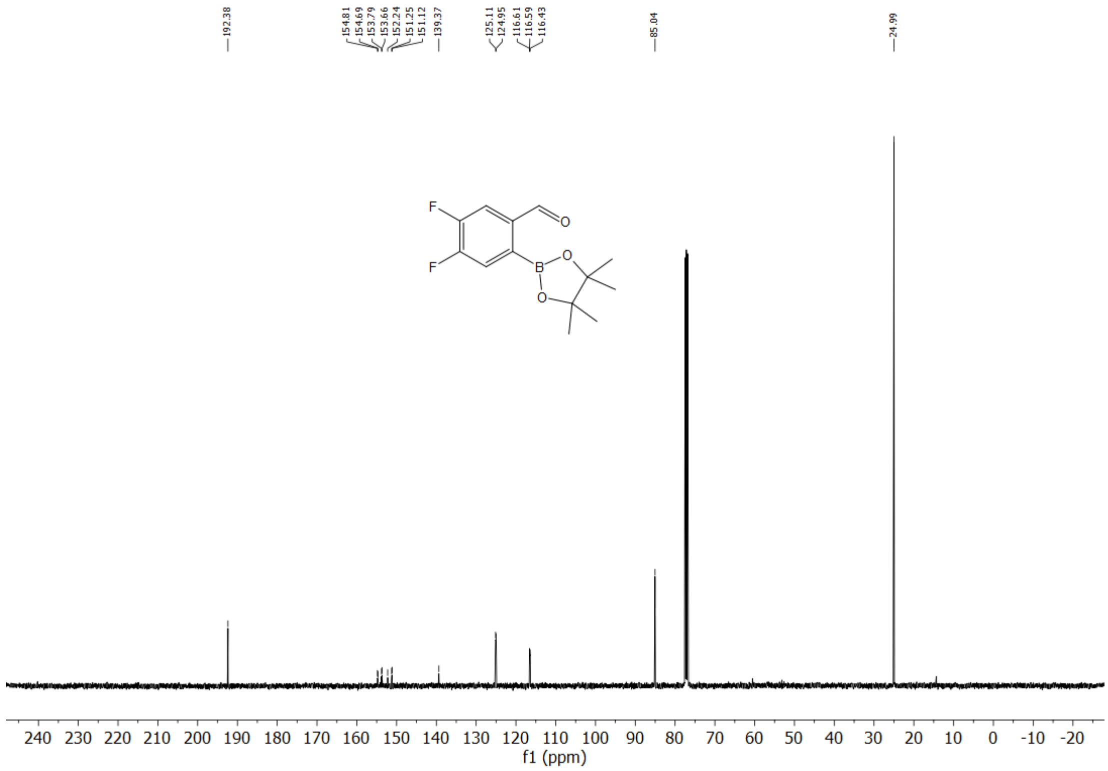

Figure S54: $^{13}C\{^{1}H\}$ NMR (101 MHz, $CDCl_3$, 298 K) spectrum of 4,5-difluoro-2-(4,4,5,5-tetramethyl-1,3,2-dioxaborolan-2-yl)benzaldehyde.

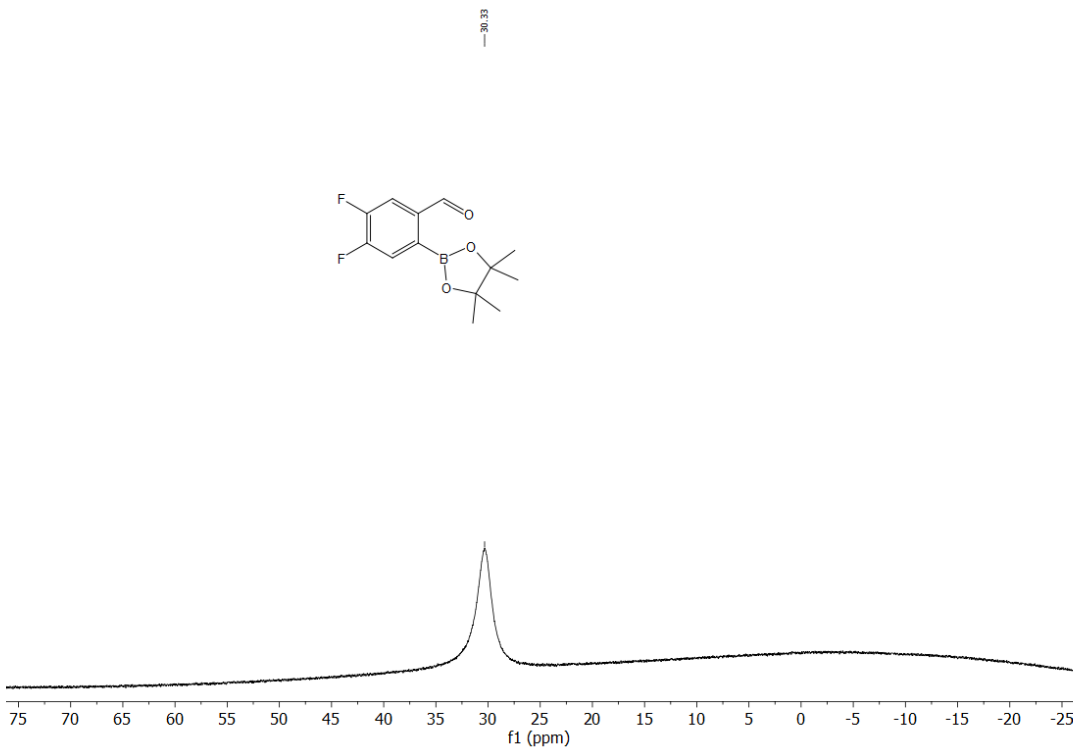

Figure S55: $^{11}B\{^{1}H\}$ NMR (128 MHz, $CDCl_3$, 298 K) spectrum of 4,5-difluoro-2-(4,4,5,5-tetramethyl-1,3,2-dioxaborolan-2-yl)benzaldehyde.

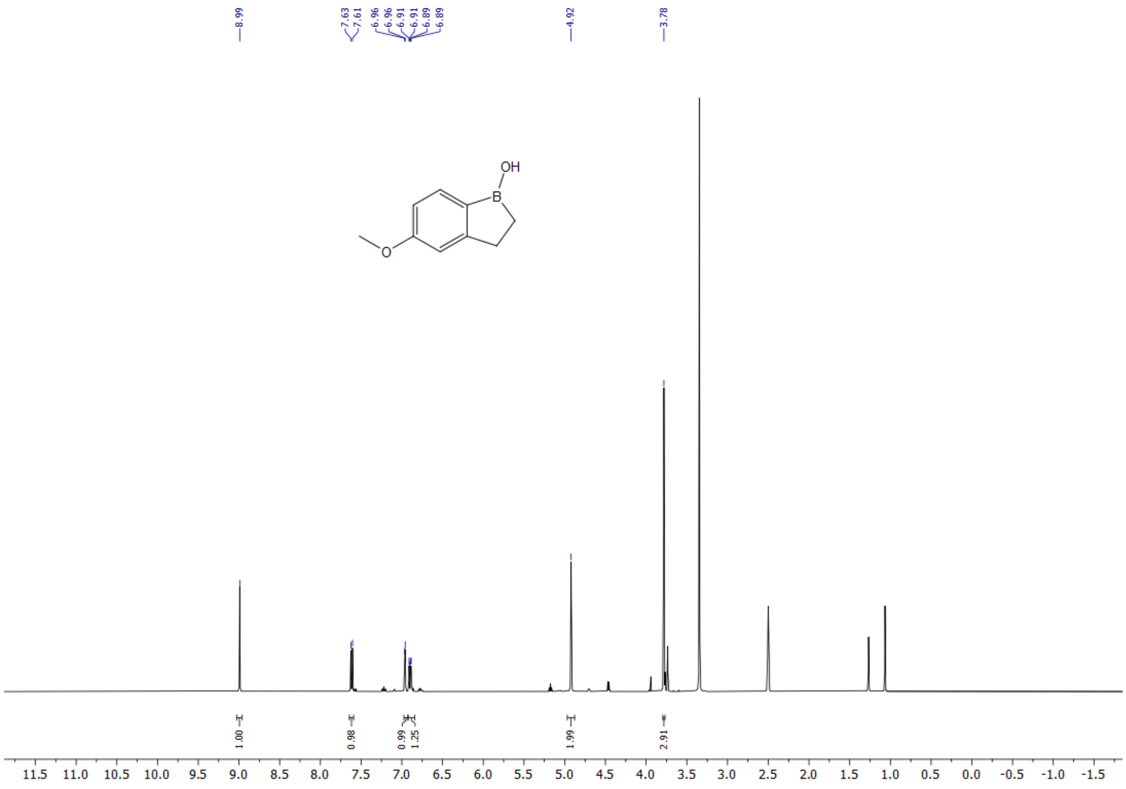

Figure S56: ^{1}H NMR (400 MHz, $CDCl_3$, 298 K) spectrum of 5-methoxybenzo[*c*][1,2]oxaborol-1(3*H*)-ol (5b).

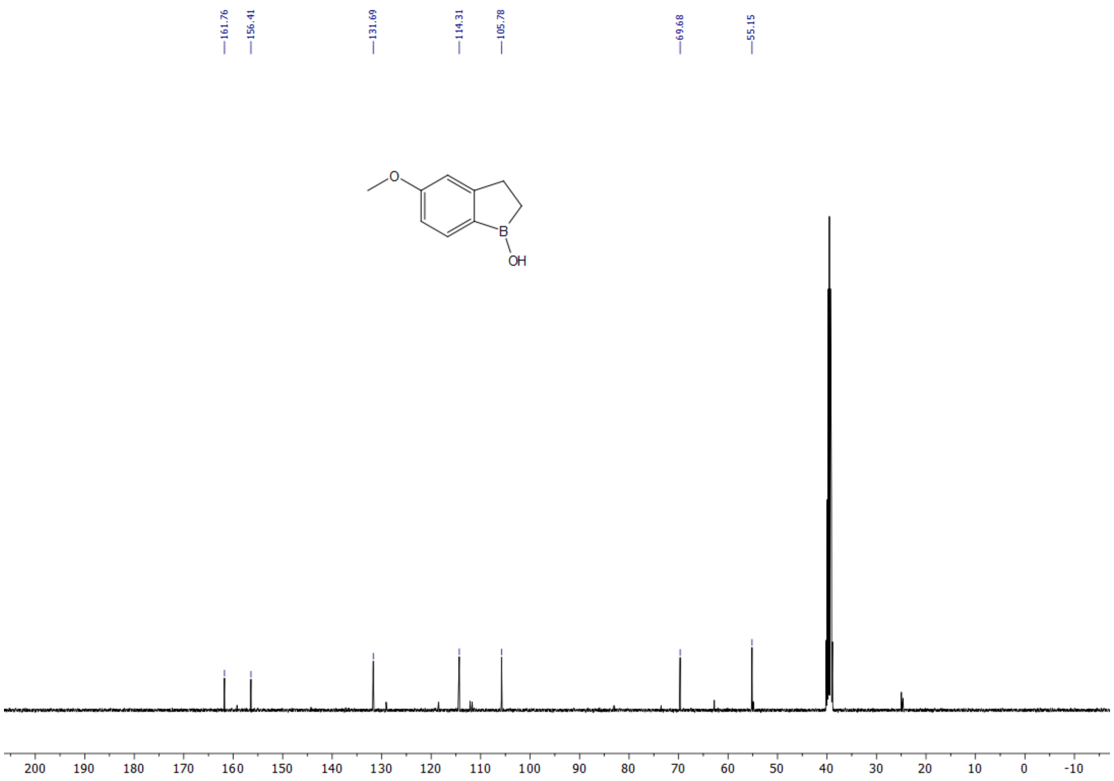

Figure S57: $^{13}C\{^{1}H\}$ **NMR (101 MHz,** $CDCl_3$**, 298 K) spectrum of 5-methoxybenzo[*c*][1,2]oxaborol-1(3*H*)-ol (5b).**

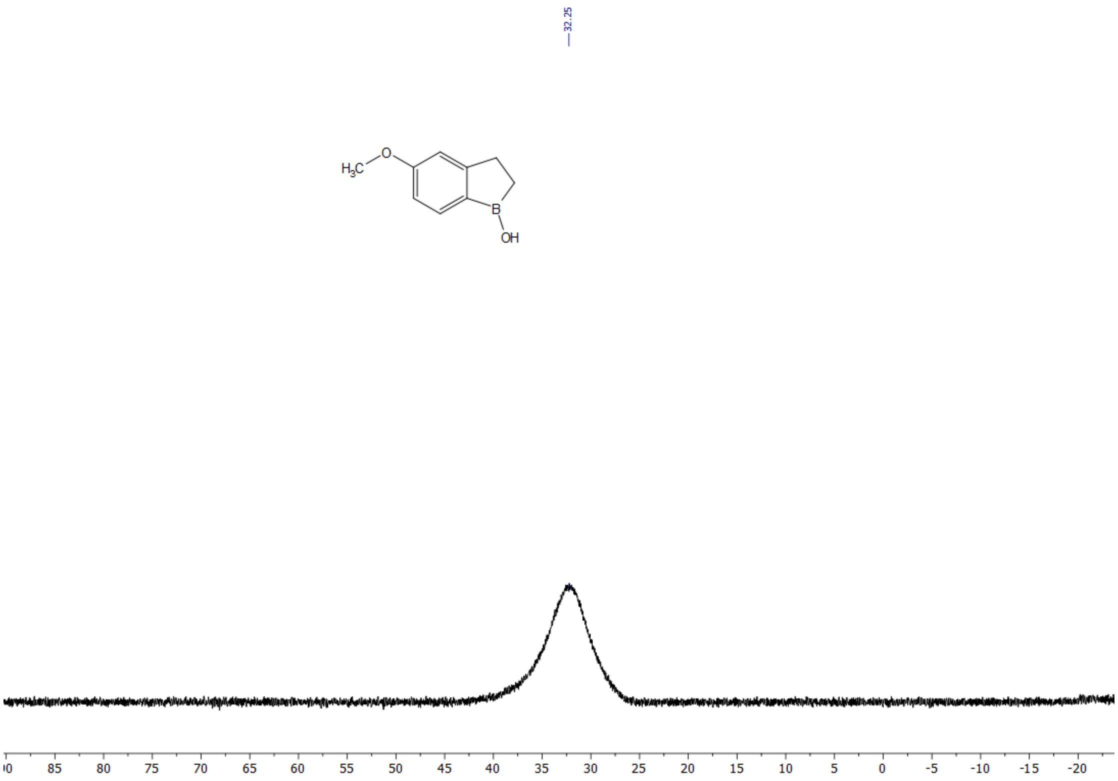

Figure S58: $^{11}B\{^{1}H\}$ **NMR (128 MHz,** $CDCl_3$**, 298 K) spectrum of 5-methoxybenzo[*c*][1,2]oxaborol-1(3*H*)-ol (5b).**

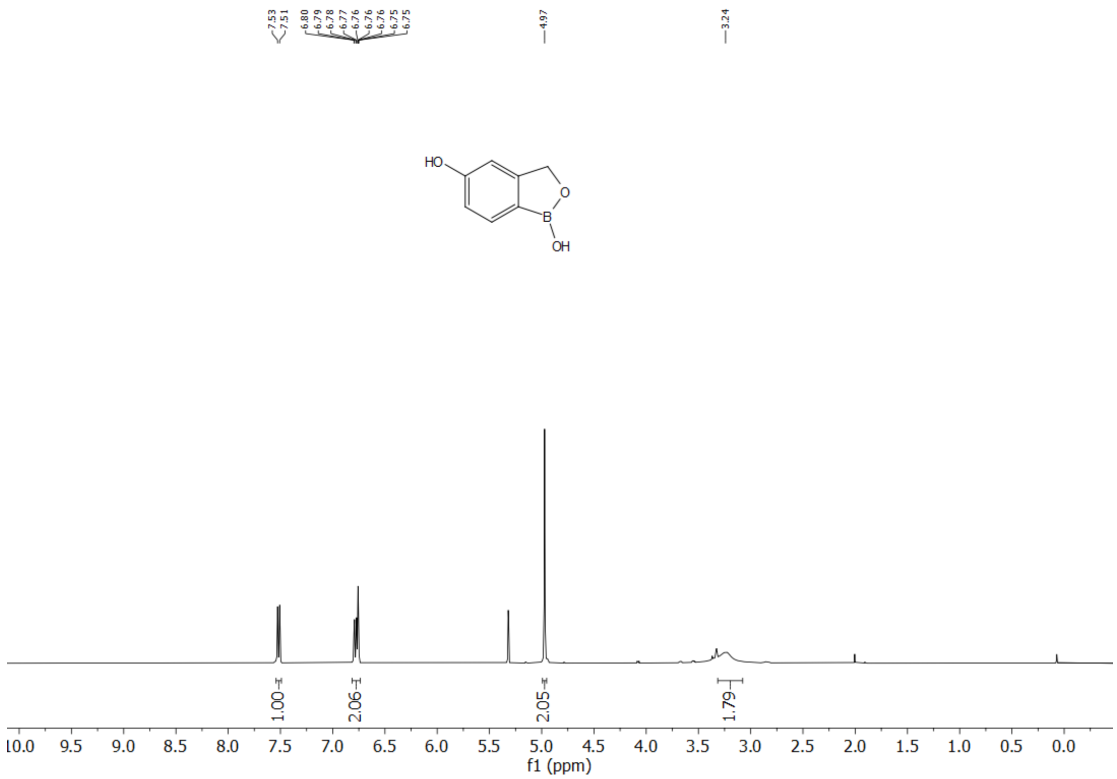

Figure S59: ^{1}H NMR (400 MHz, CD_2Cl_2, 298 K) spectrum of Benzo[*c*][1,2]oxaborole-1,5(3*H*)-diol (5c)

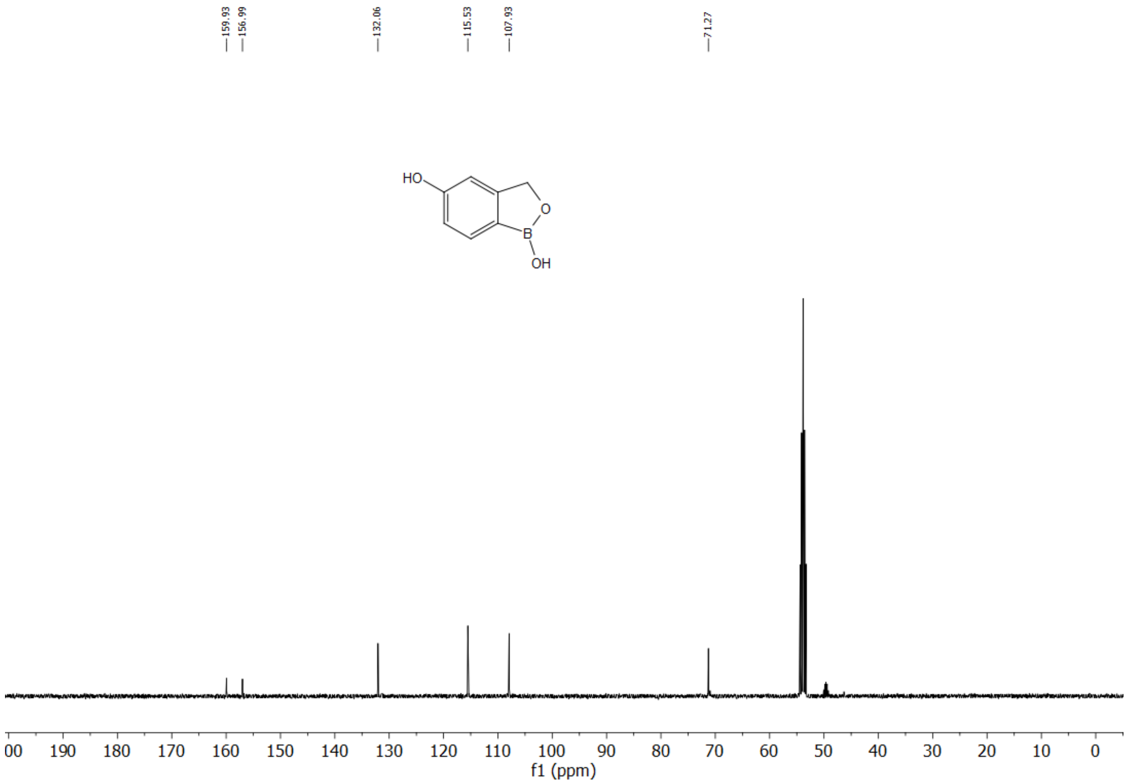

Figure S60: $^{13}C\{^{1}H\}$ NMR (101 MHz, CD_2Cl_2, 298 K) spectrum of Benzo[*c*][1,2]oxaborole-1,5(3*H*)-diol (5c).

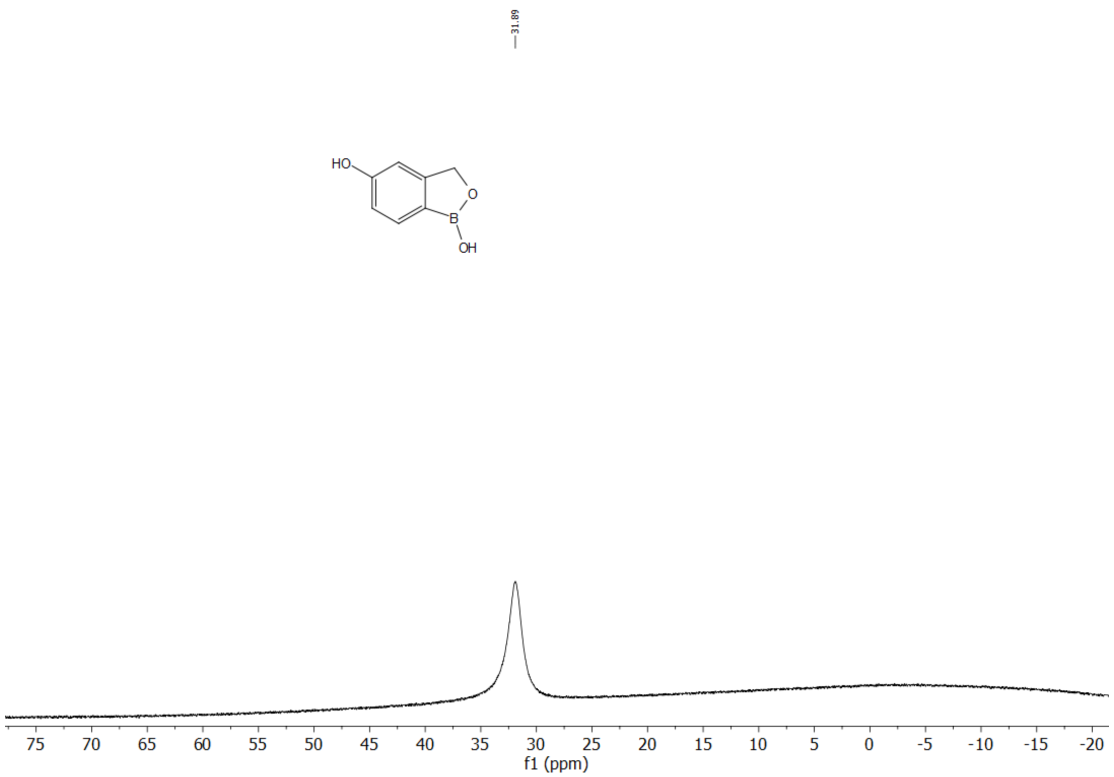

Figure S61: $^{11}B\{^{1}H\}$ NMR (128 MHz, CD_2Cl_2, 298 K) spectrum of Benzo[*c*][1,2]oxaborole-1,5(3*H*)-diol (5c).

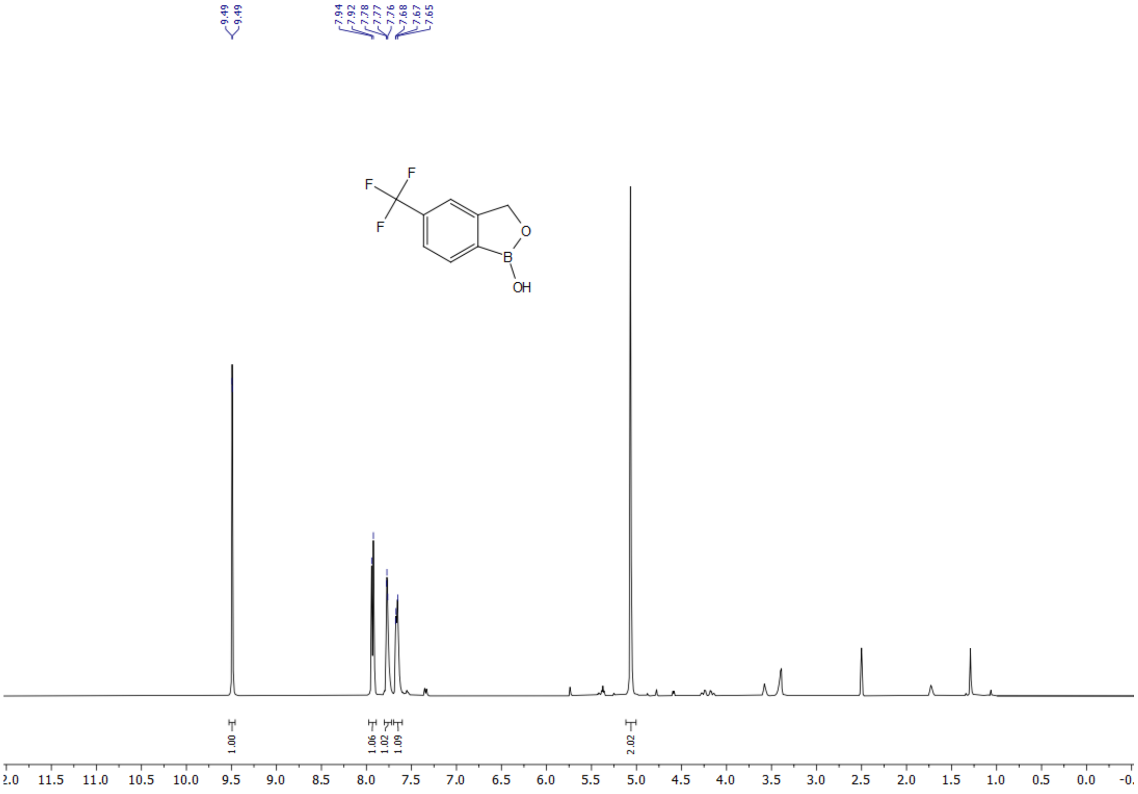

Figure S62: ^{1}H NMR (400 MHz, DMSO-d6, 298 K) spectrum of 5-(trifluoromethyl)benzo[*c*][1,2]oxaborol-1(3*H*)-ol (5d).

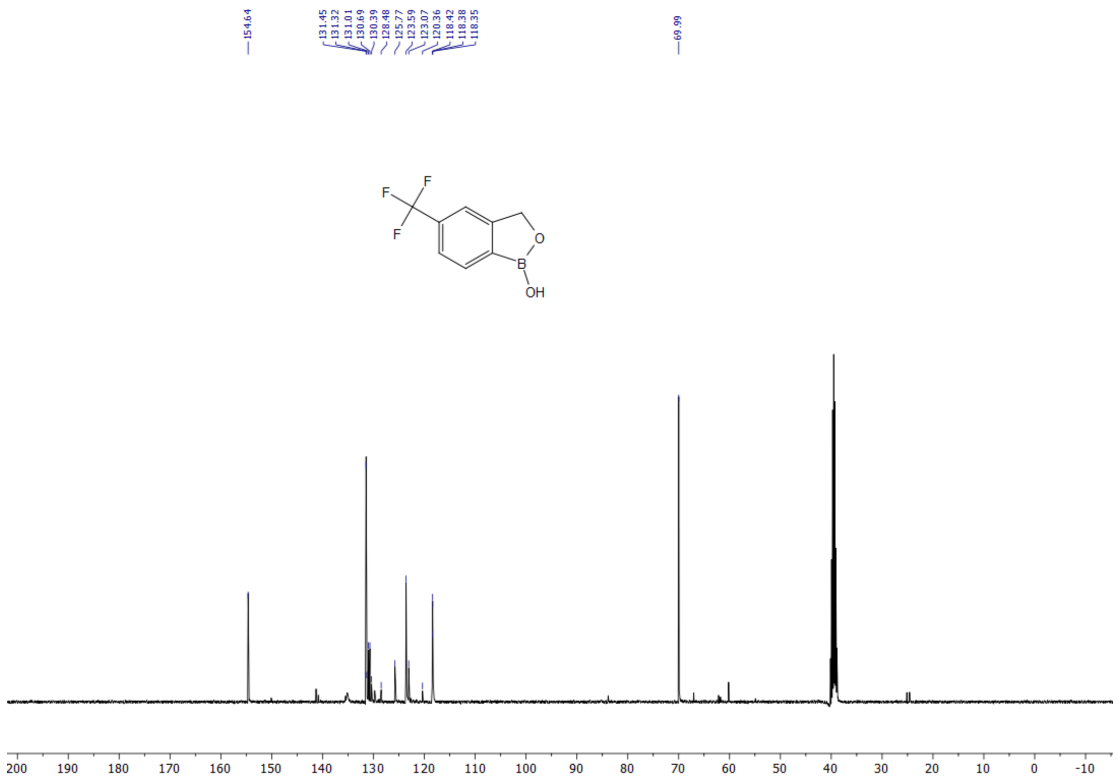

Figure S63: $^{13}C\{^1H\}$ NMR (101 MHz, DMSO-d6, 298 K) spectrum of 5-(trifluoromethyl)benzo[*c*][1,2]oxaborol-1(3*H*)-ol (5d).

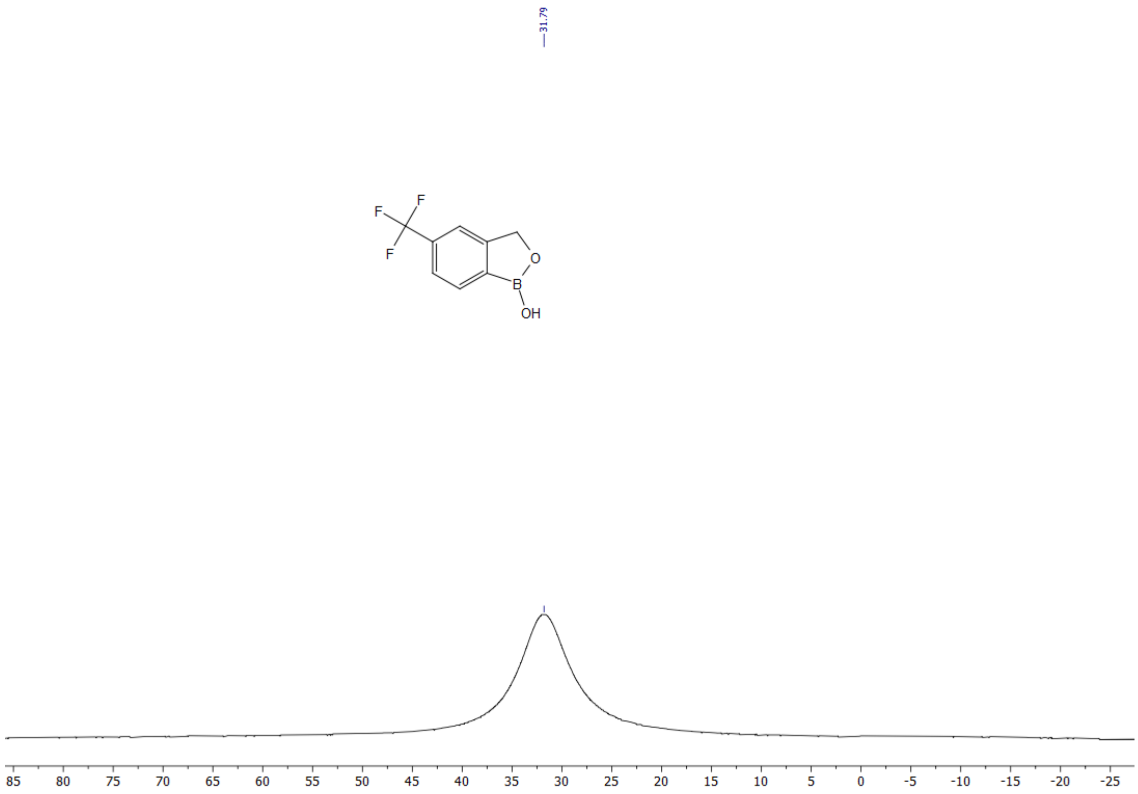

Figure S64: $^{11}B\{^1H\}$ NMR (128 MHz, DMSO-d6, 298 K) spectrum of 5-(trifluoromethyl)benzo[*c*][1,2]oxaborol-1(3*H*)-ol (5d).

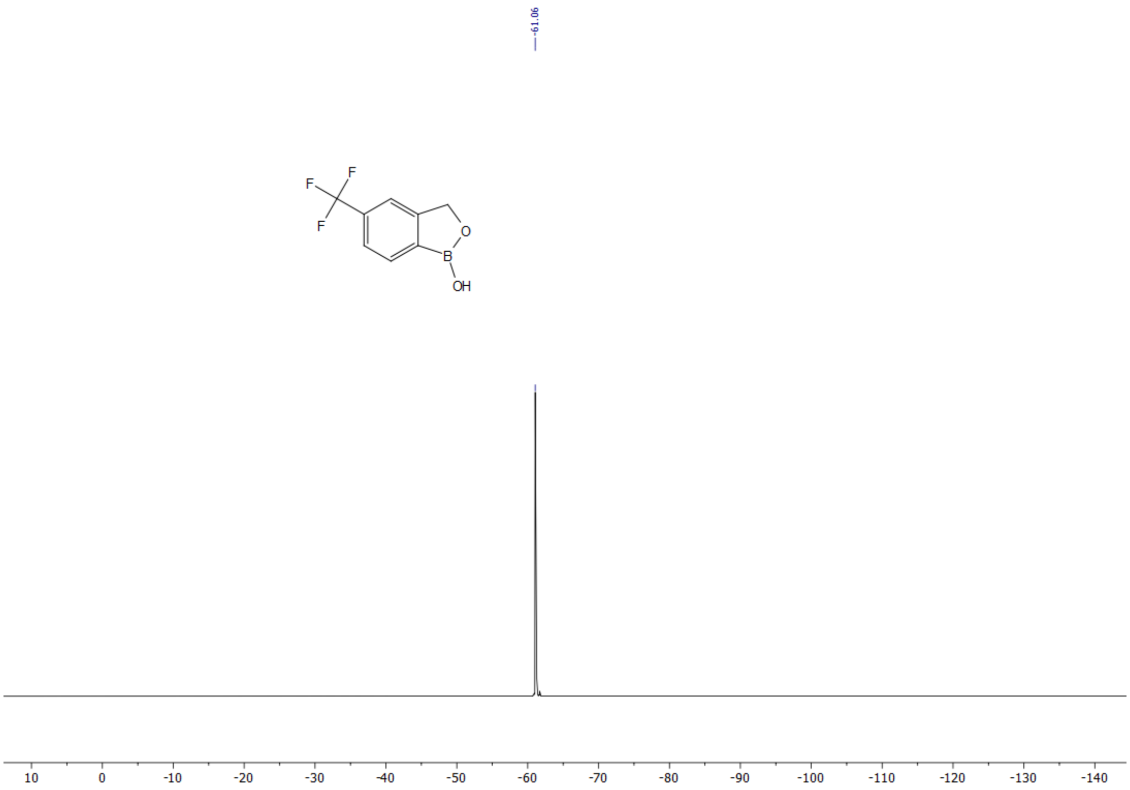

Figure S65: $^{19}F\{^1H\}$-NMR (376 MHz, DMSO-d6, 298 K) spectrum of 5-(trifluoromethyl)benzo[*c*][1,2]oxaborol-1(3*H*)-ol (5d).

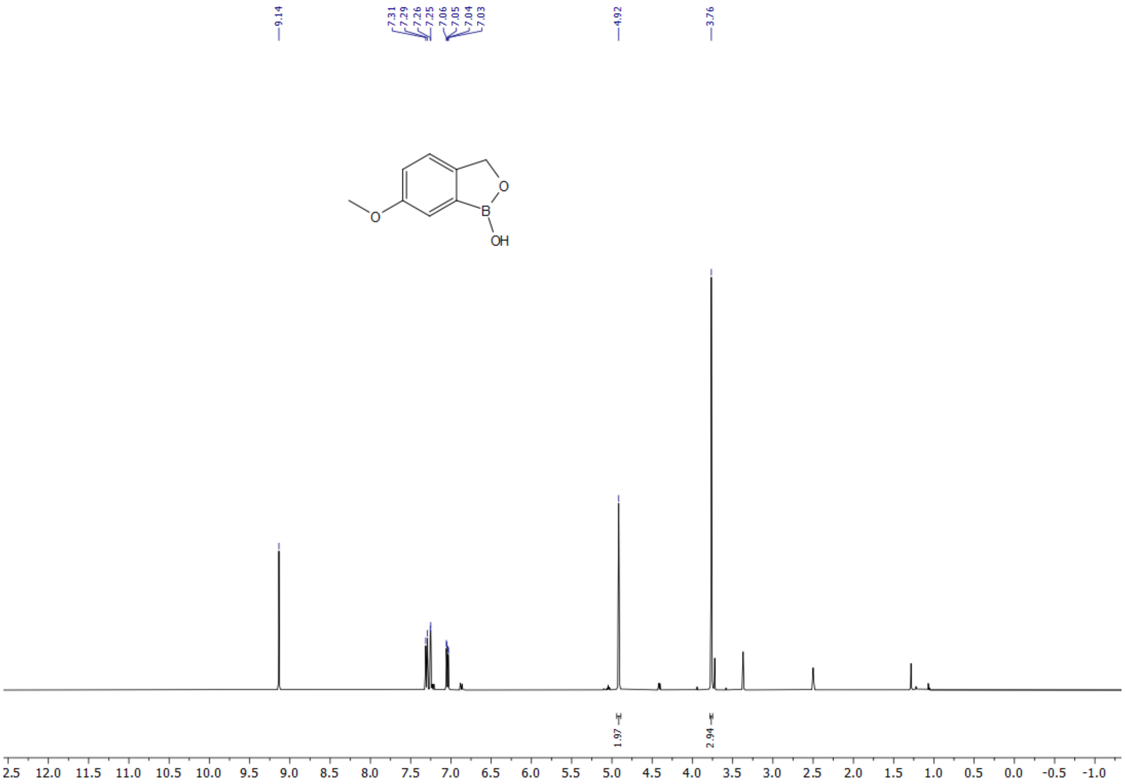

Figure S66: ^1H NMR (400 MHz, $CDCl_3$, 298 K) spectrum of 6-methoxybenzo[*c*][1,2]oxaborol-1(3*H*)-ol (5f).

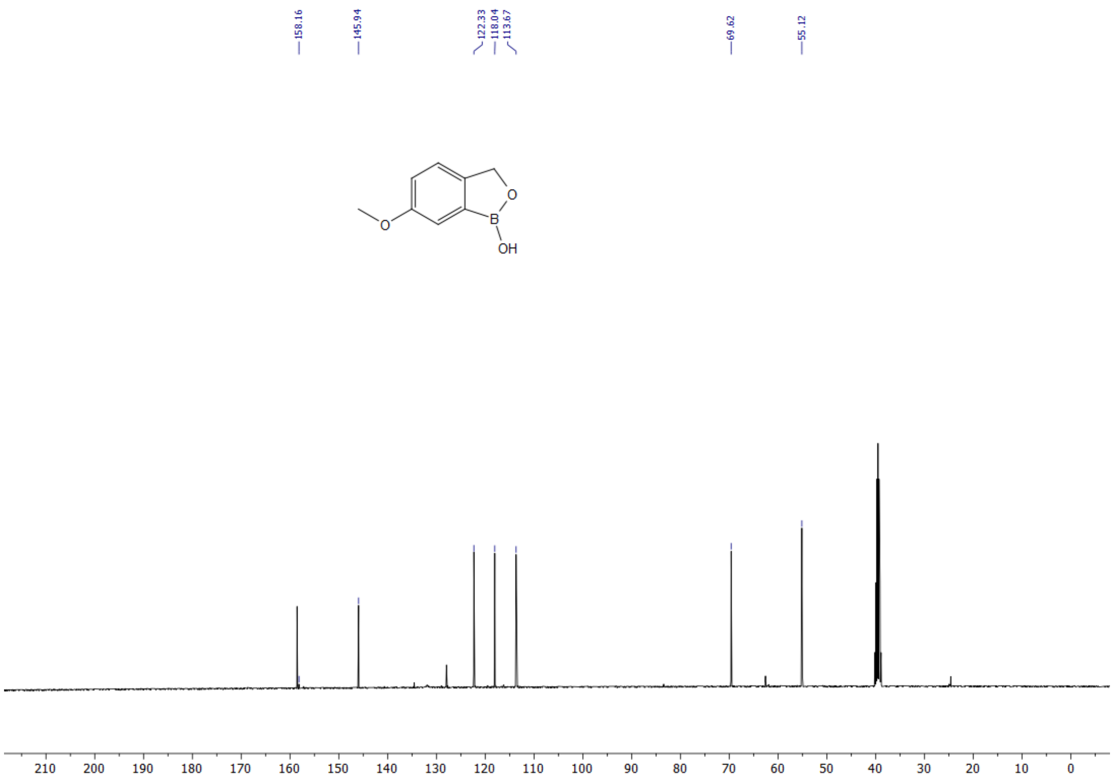

Figure S67: $^{13}C\{^1H\}$ NMR (101 MHz, $CDCl_3$, 298 K) spectrum of 6-methoxybenzo[*c*][1,2]oxaborol-1(3*H*)-ol (5f).

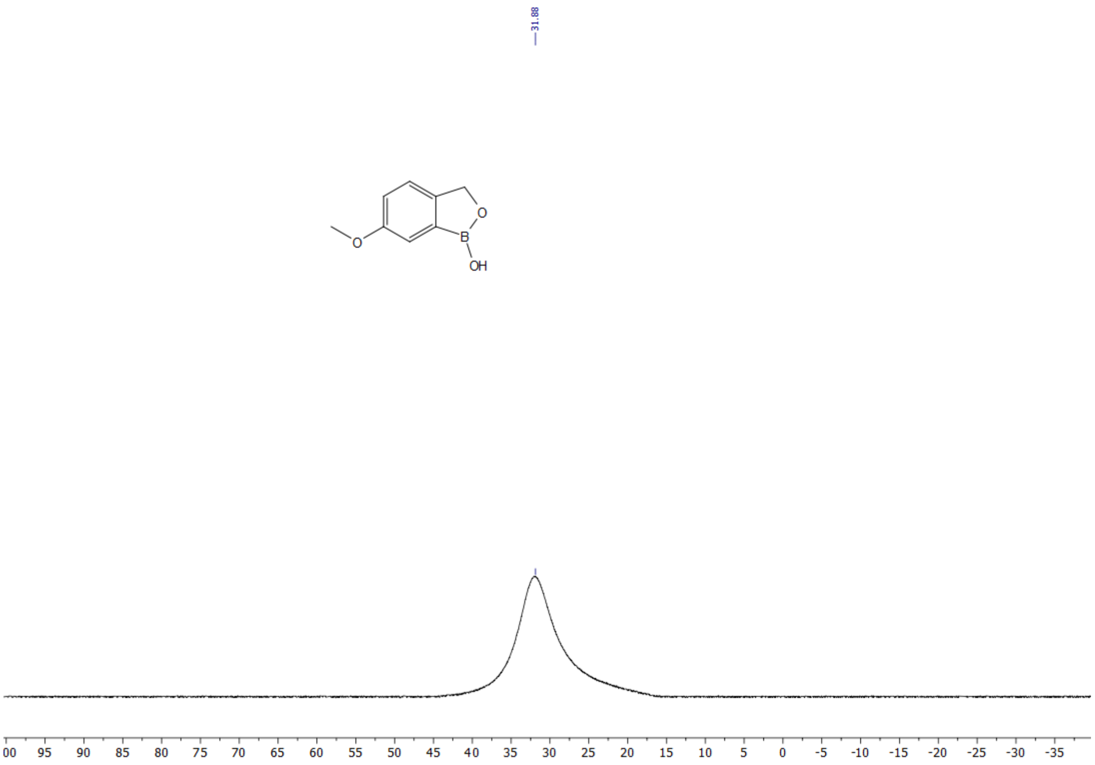

Figure S68: $^{11}B\{^1H\}$ NMR (128 MHz, $CDCl_3$, 298 K) spectrum of 6-methoxybenzo[*c*][1,2]oxaborol-1(3*H*)-ol (5f).

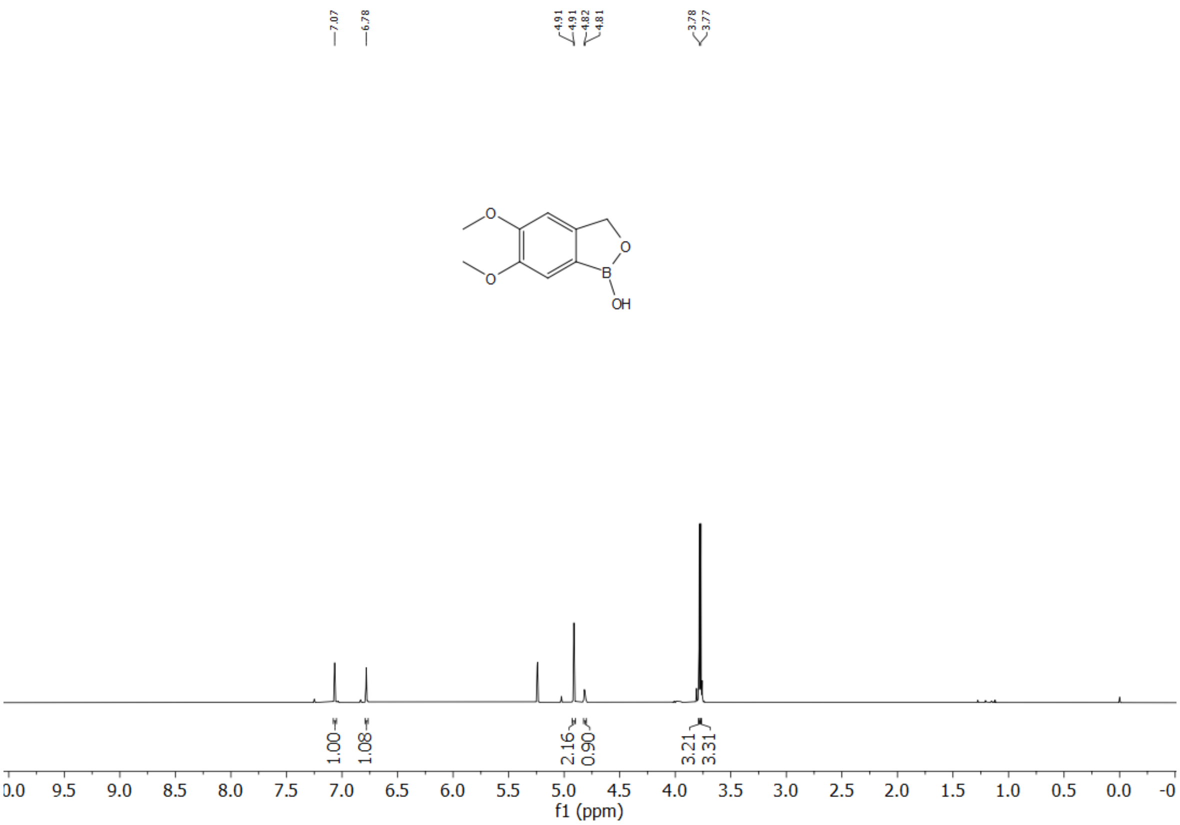

Figure S69: ^{1}H NMR (400 MHz, CD_2Cl_2, 298 K) spectrum of 5,6-dimethoxybenzo[*c*][1,2]oxaborol-1(3*H*)-ol (5j).

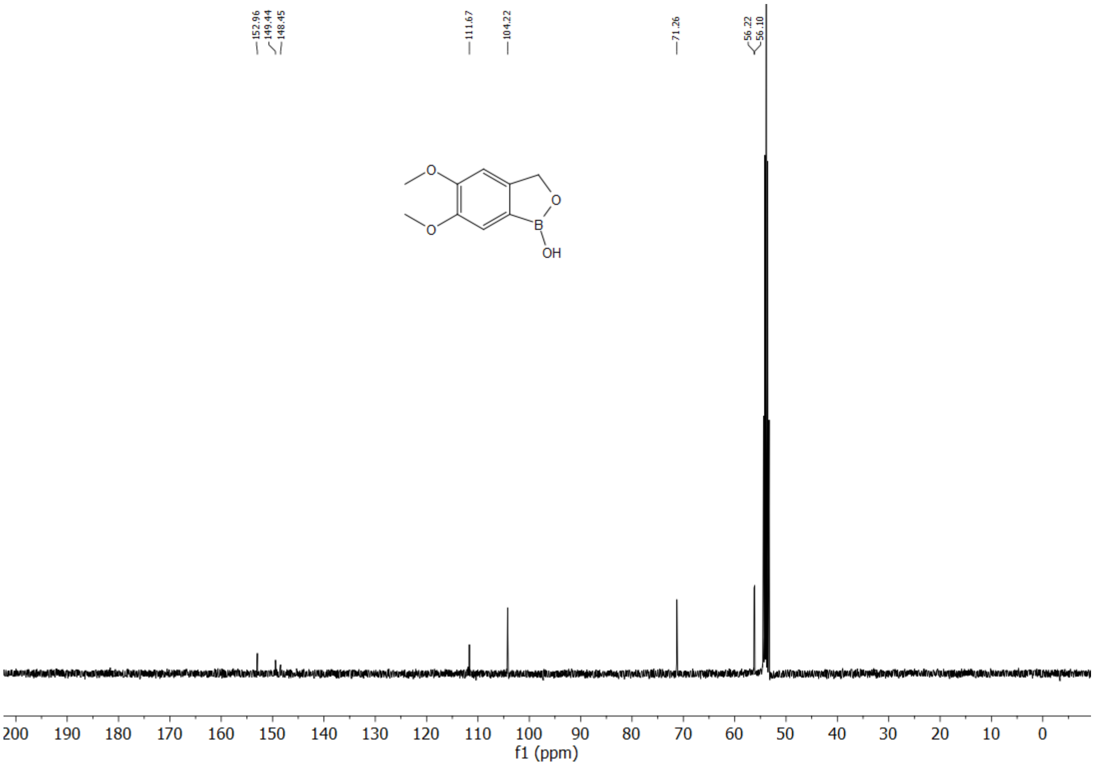

Figure S70: $^{13}C\{^{1}H\}$ NMR (101 MHz, CD_2Cl_2, 298 K) spectrum of 5,6-dimethoxybenzo[*c*][1,2]oxaborol-1(3*H*)-ol (5j).

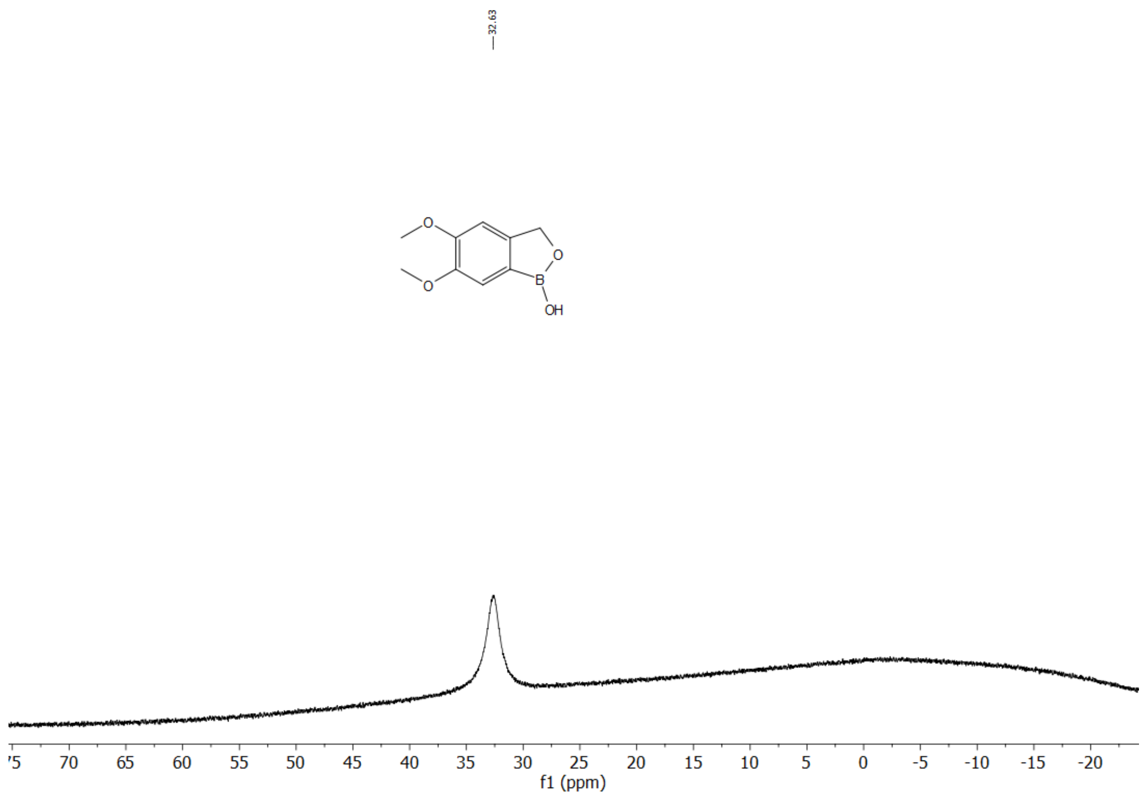

Figure S71: $^{11}B\{^1H\}$ NMR (128 MHz, CD_2Cl_2, 298 K) spectrum of 5,6-dimethoxybenzo[*c*][1,2]oxaborol-1(3*H*)-ol (5j).

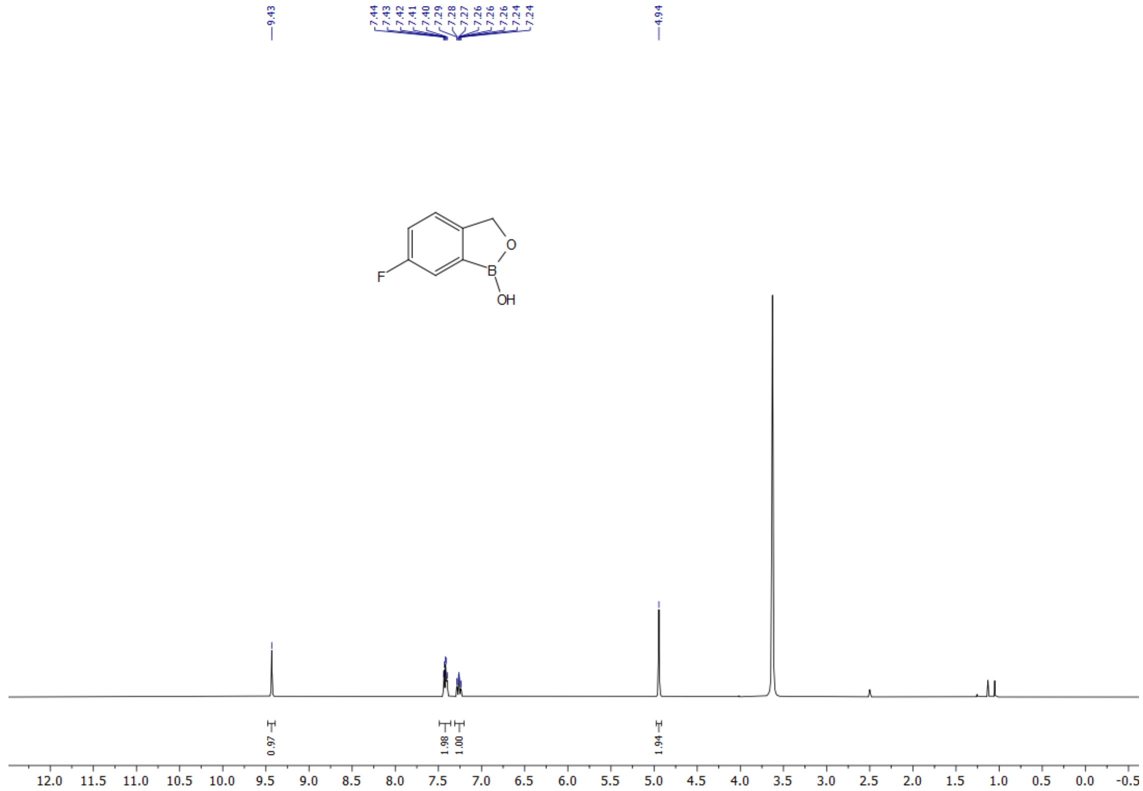

Figure S72: ^1H NMR (400 MHz,DMSO-d6, 298 K) spectrum of 6-fluorobenzo[*c*][1,2]oxaborol-1(3*H*)-ol (5u).

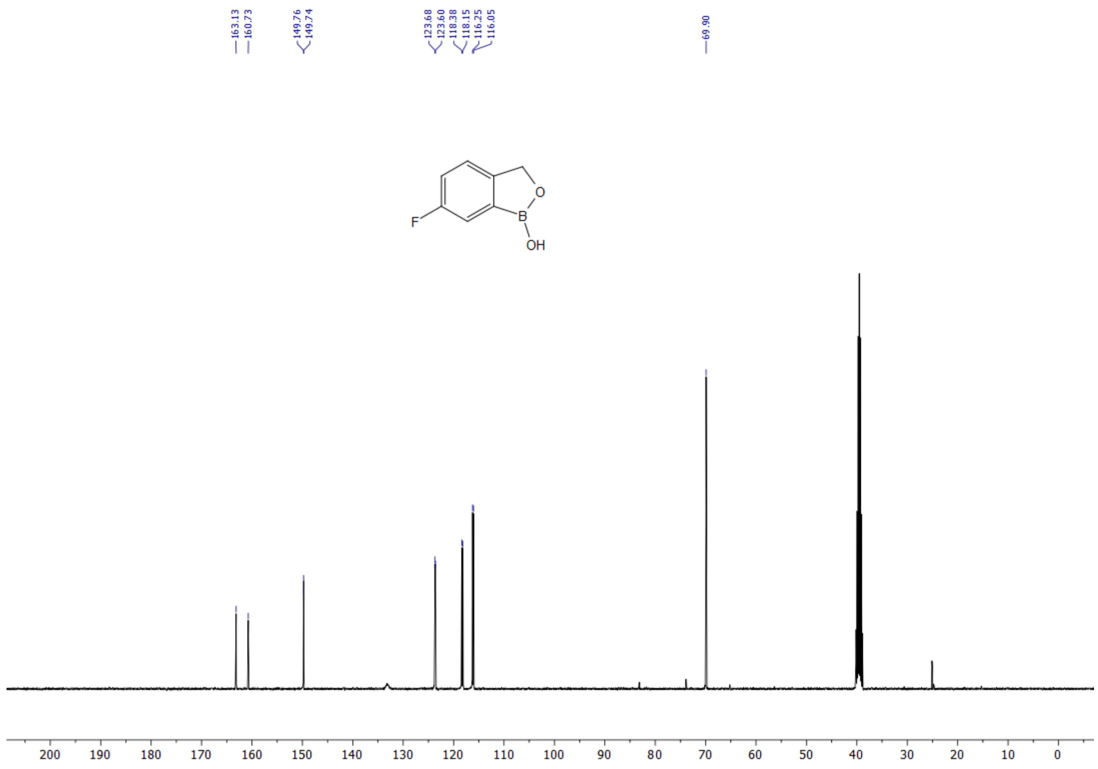

Figure S73: ^{13}C NMR (101 MHz,DMSO-d6, 298 K) spectrum of 6-fluorobenzo[*c*][1,2]oxaborol-1(3*H*)-ol (5u).

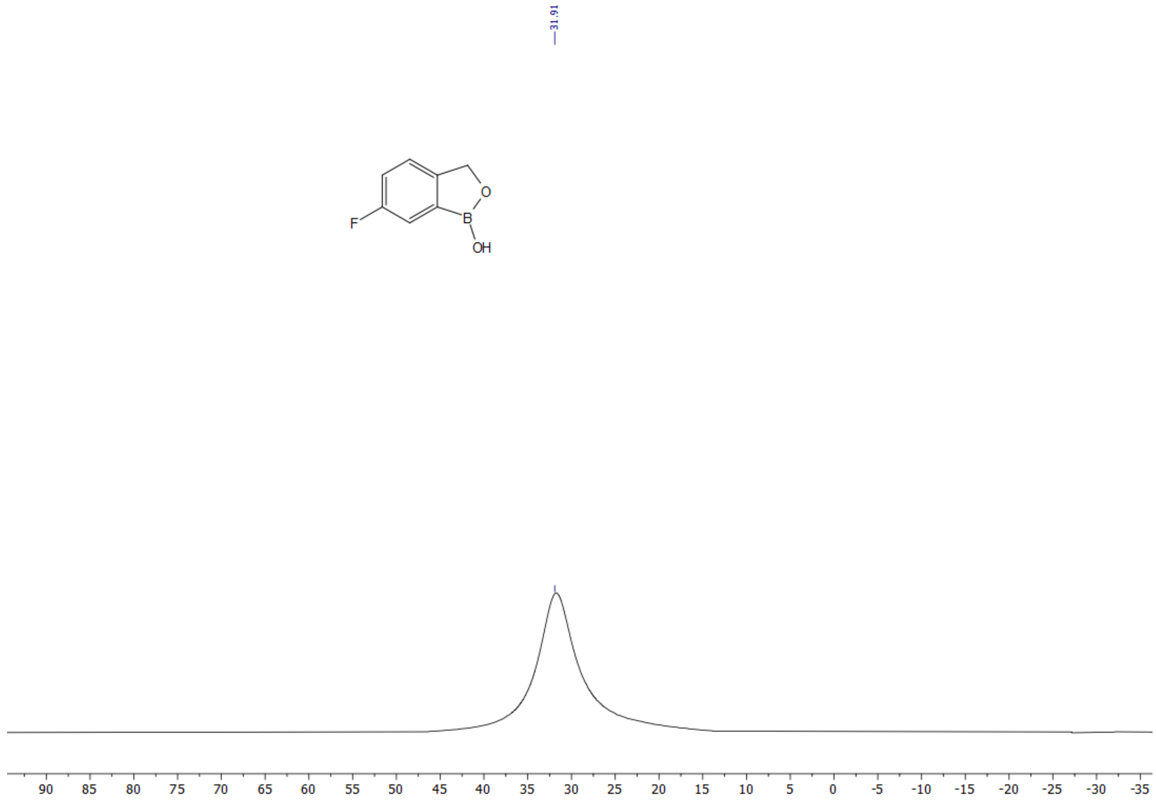

Figure S74: ^{11}B NMR (128 MHz,DMSO-d6, 298 K) spectrum of 6-fluorobenzo[*c*][1,2]oxaborol-1(3*H*)-ol (5u).

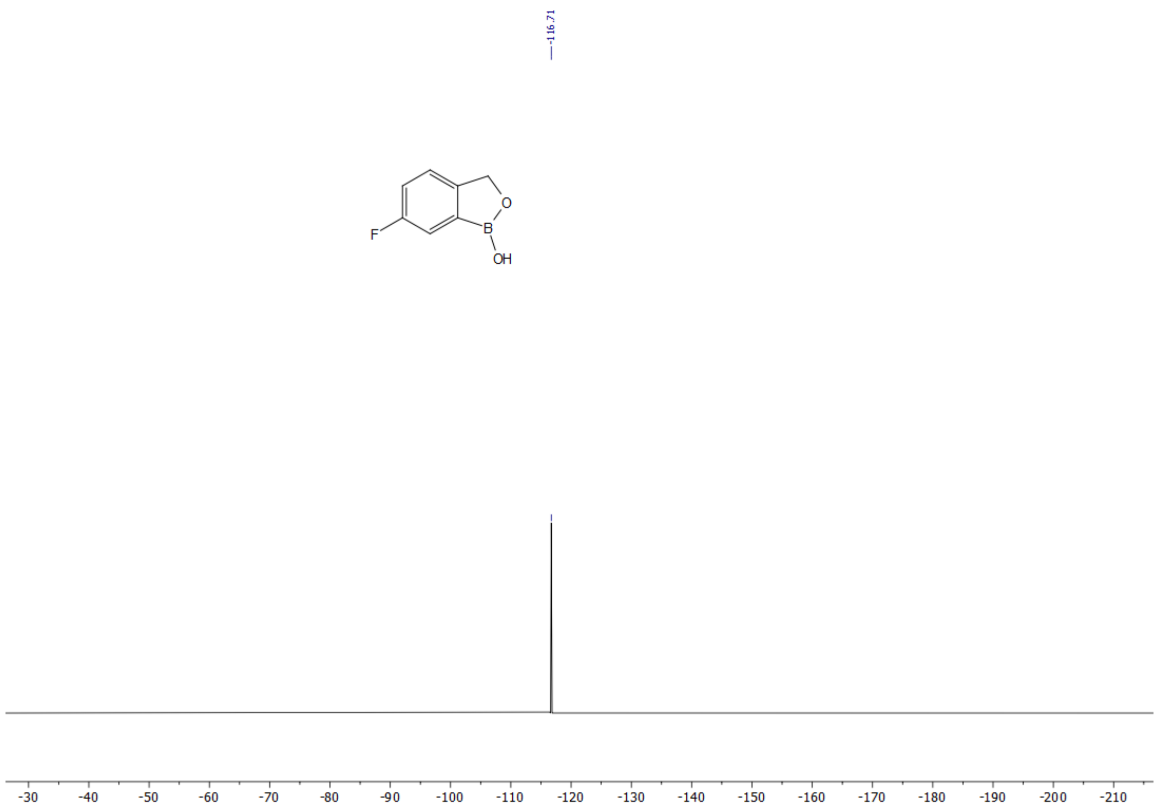

Figure S75: ^{19}F NMR (376 MHz,DMSO-d6, 298 K) spectrum of 6-fluorobenzo[*c*][1,2]oxaborol-1(3*H*)-ol (5u).

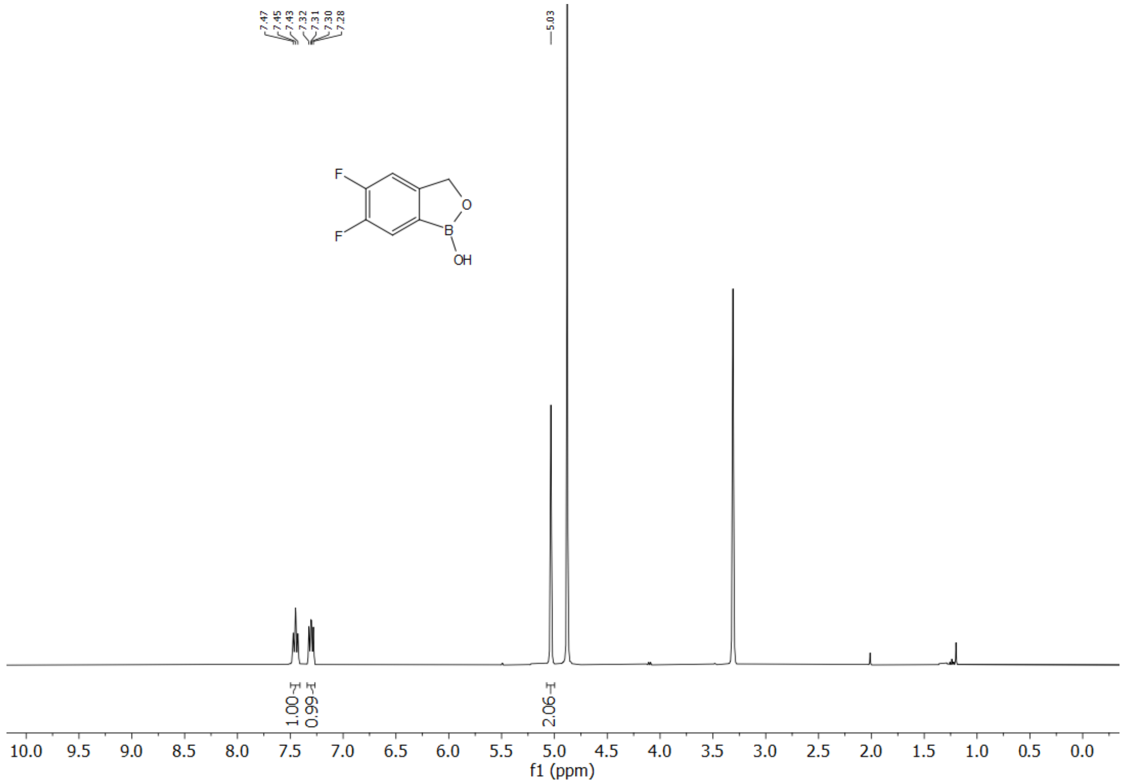

Figure S76: ^{1}H NMR (400 MHz, CD_3OD, 298 K) spectrum of 5,6-difluorobenzo[*c*][1,2]oxaborol-1(3*H*)-ol (5v).

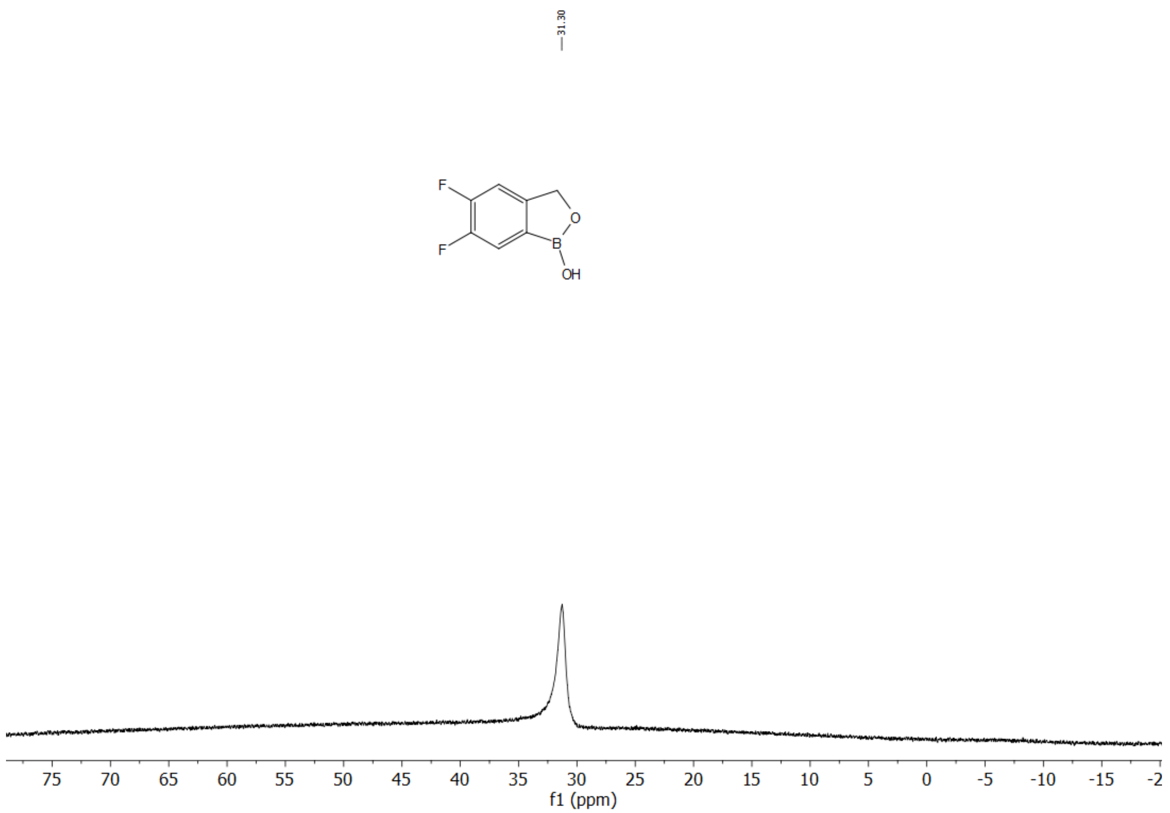

Figure S77: $^{11}B\{^{1}H\}$ NMR (128 MHz, CD_3OD, 298 K) spectrum of 5,6-difluorobenzo[*c*][1,2]oxaborol-1(3*H*)-ol (5v).

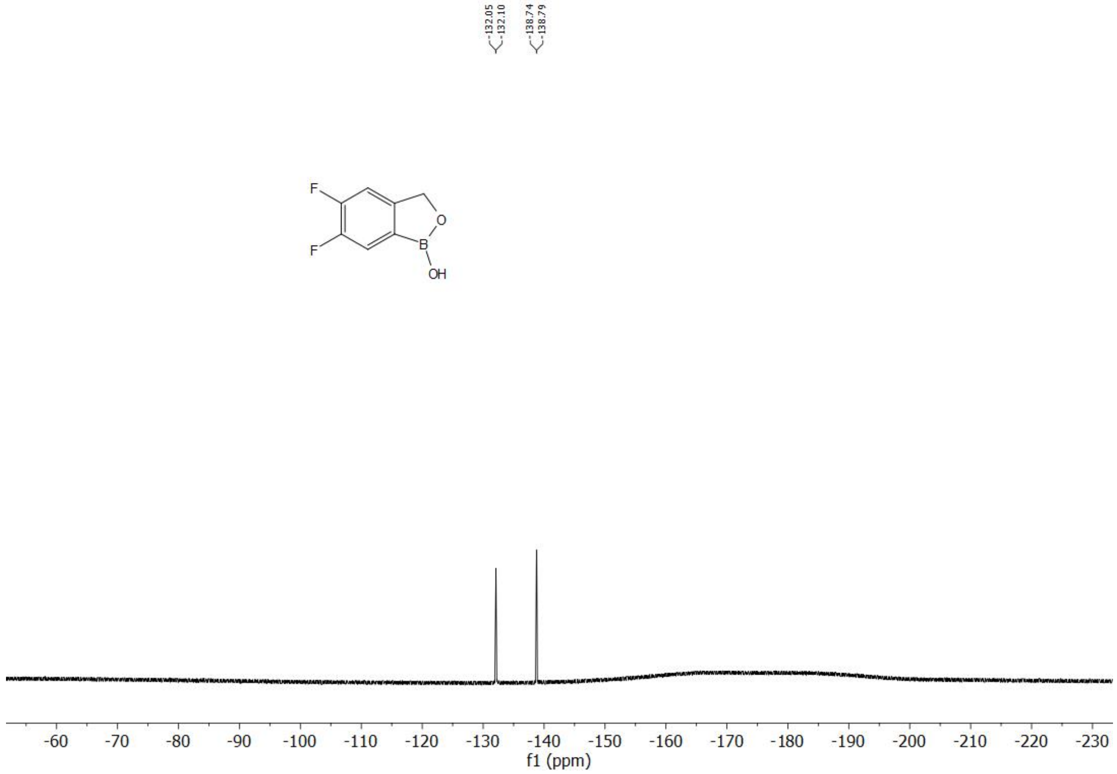

Figure S78: $^{19}F\{^{1}H\}$-NMR (376 MHz, CD_3OD, 298 K) spectrum of 5,6-difluorobenzo[*c*][1,2]oxaborol-1(3*H*)-ol (5v).

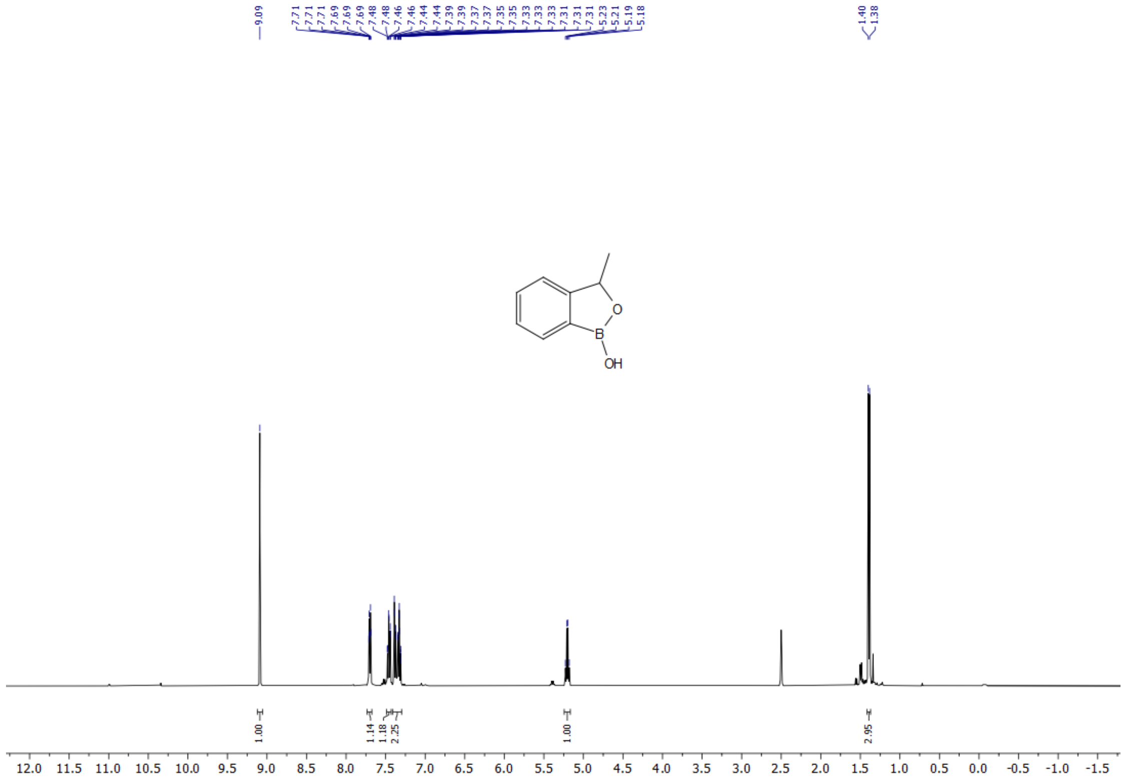

Figure S79: ^{1}H NMR (400 MHz,DMSO-d6, 298 K) spectrum of 3-methylbenzo[*c*][1,2]oxaborol-1(3*H*)-ol (5k).

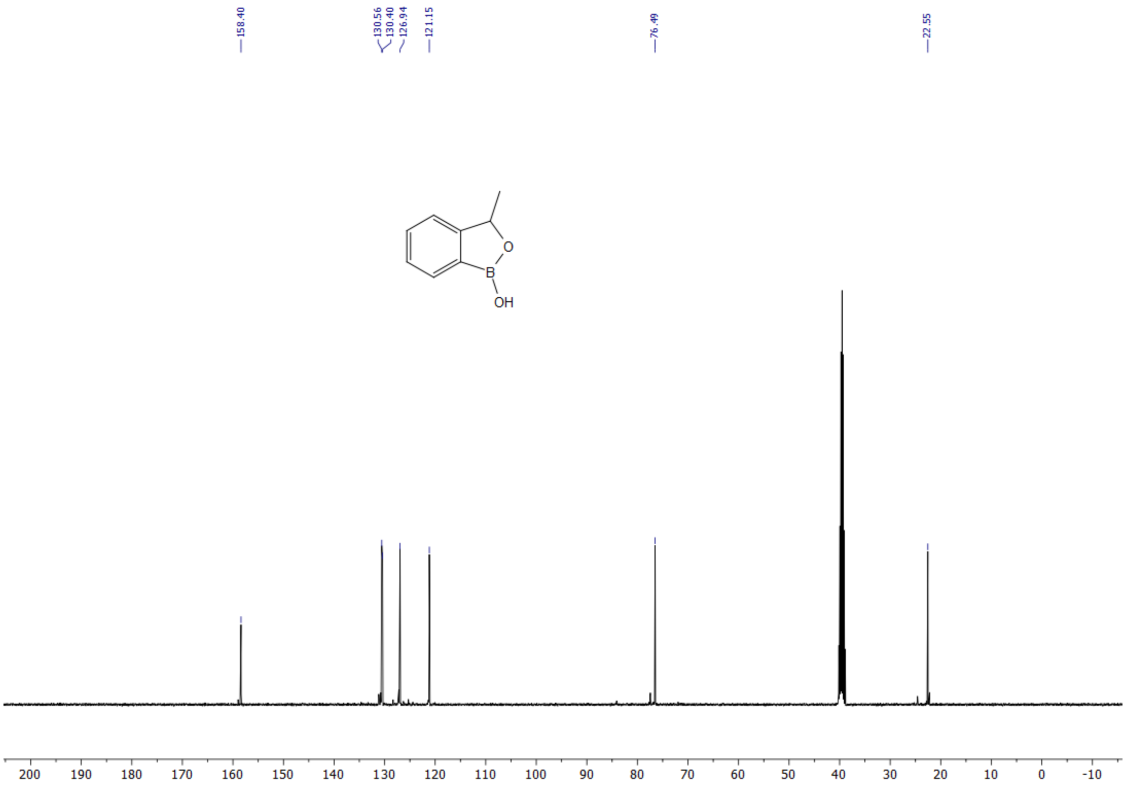

Figure S80: ^{13}C NMR (101 MHz,DMSO-d6, 298 K) spectrum of 3-methylbenzo[*c*][1,2]oxaborol-1(3*H*)-ol (5k).

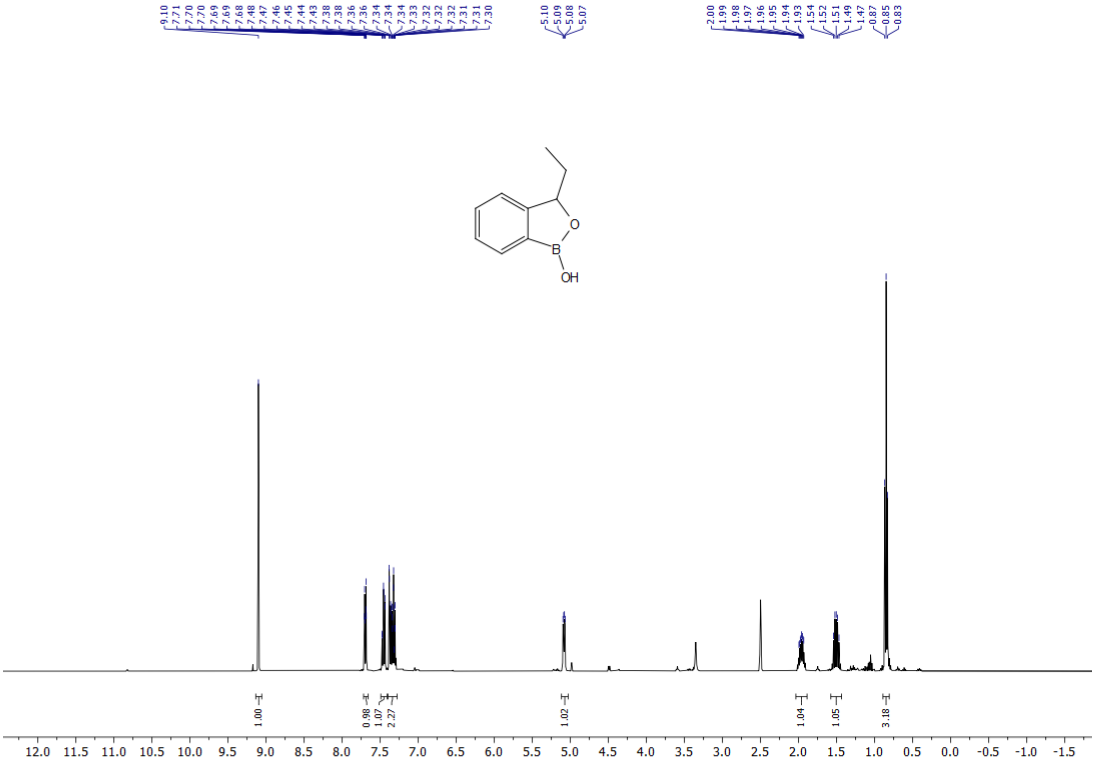

Figure S81: ^{1}H NMR (400 MHz,DMSO-d6, 298 K) spectrum of 3-ethylbenzo[*c*][1,2]oxaborol-1(3*H*)-ol (5l).

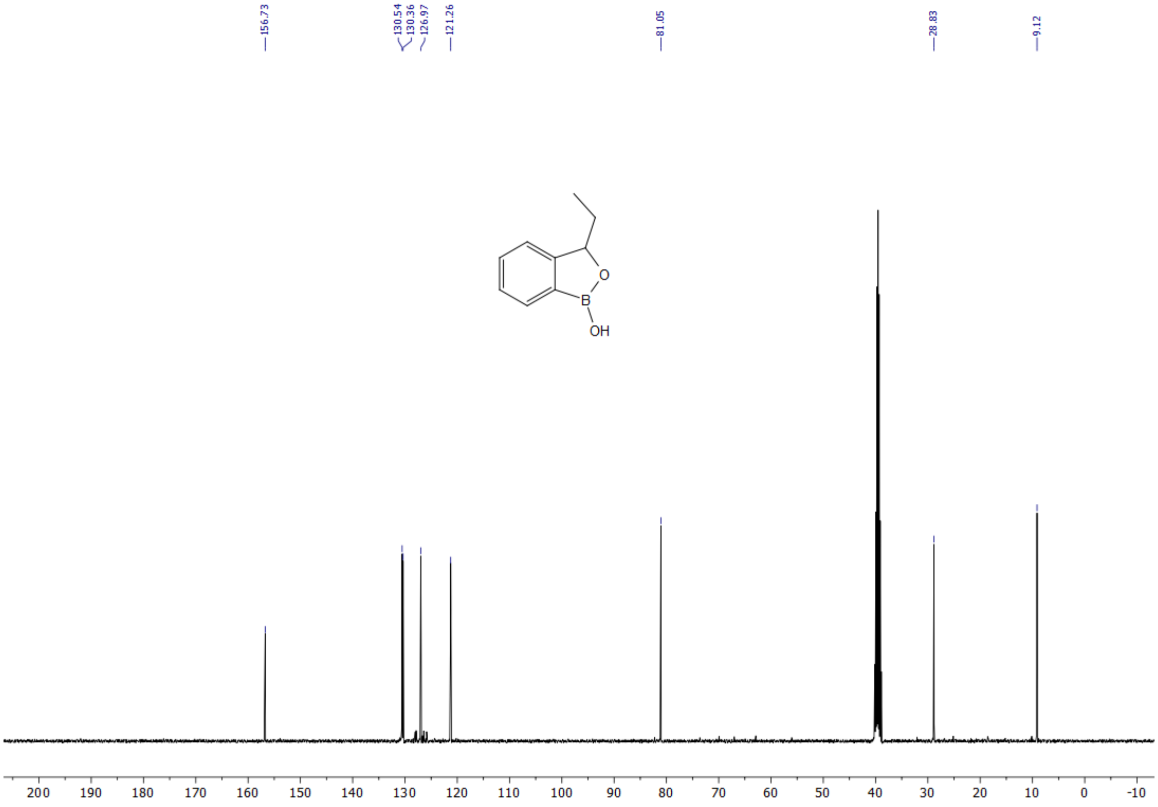

Figure S82: ^{13}C NMR (101 MHz,DMSO-d6, 298 K) spectrum of 3-ethylbenzo[*c*][1,2]oxaborol-1(3*H*)-ol (5l).

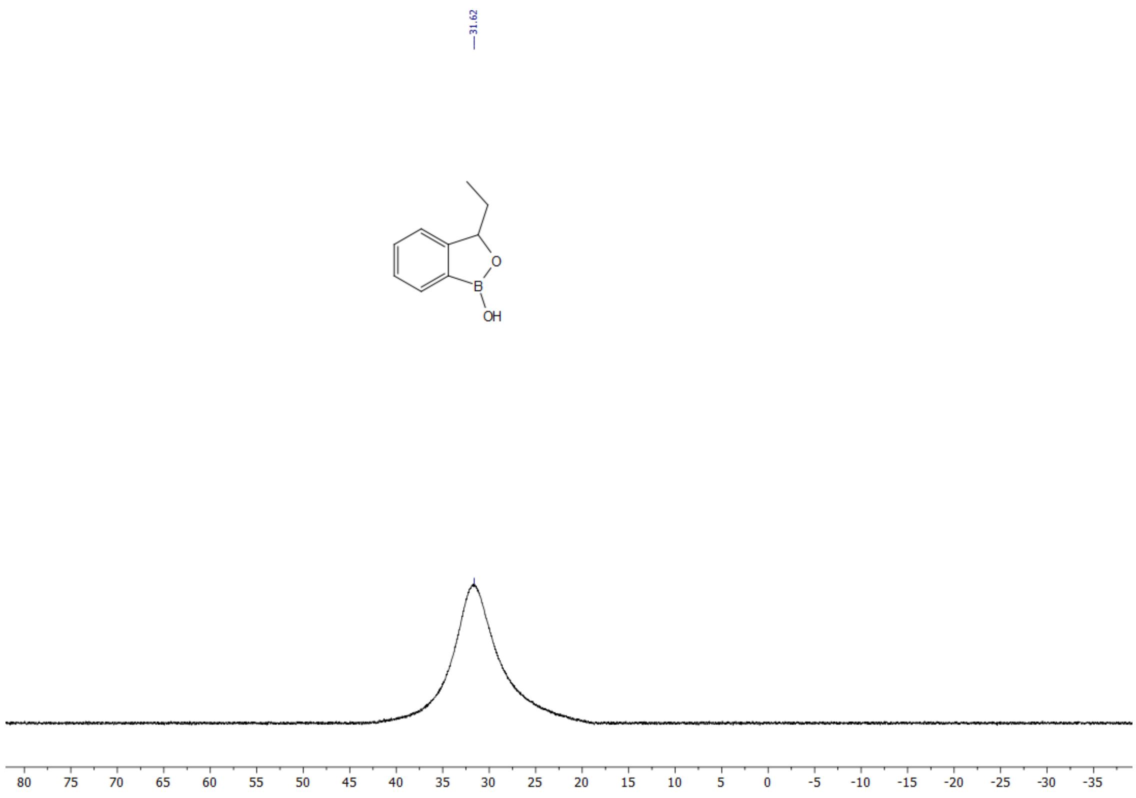

Figure S83: ^{11}B NMR (128 MHz,DMSO-d6, 298 K) spectrum of 3-ethylbenzo[*c*][1,2]oxaborol-1(3*H*)-ol (5l).

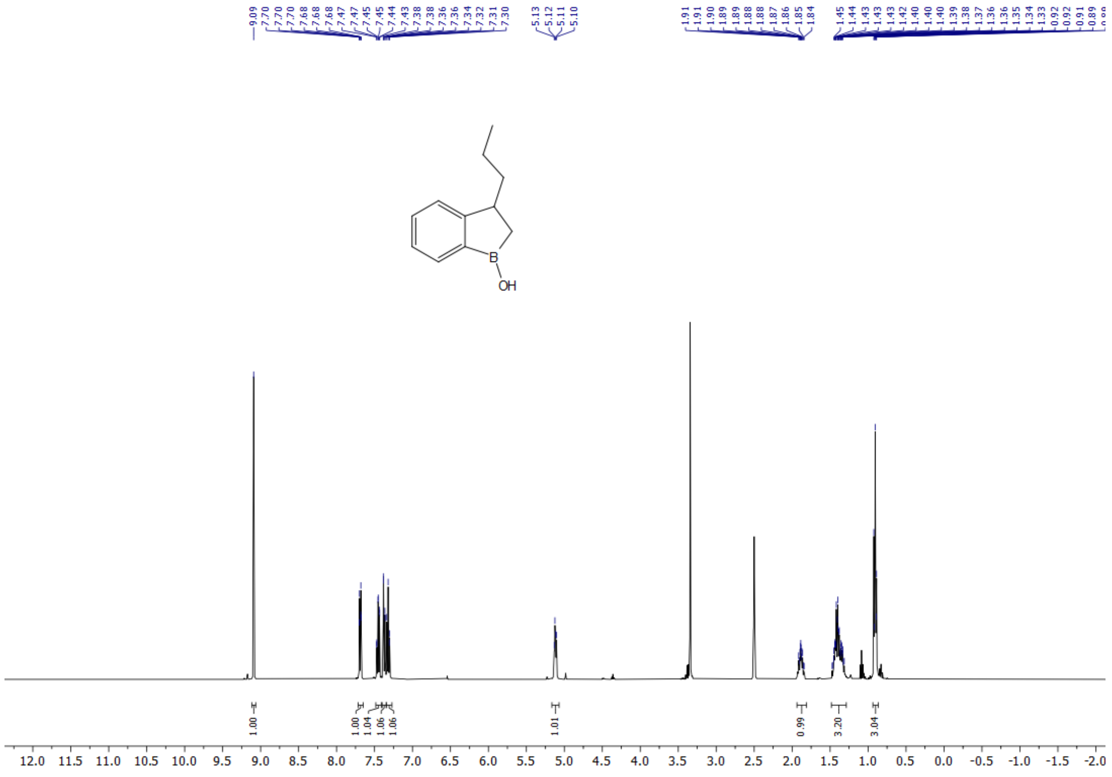

Figure S84: ^{1}H NMR (400 MHz,DMSO-d6, 298 K) spectrum of 3-propylbenzo[*c*][1,2]oxaborol-1(3*H*)-ol (5m).

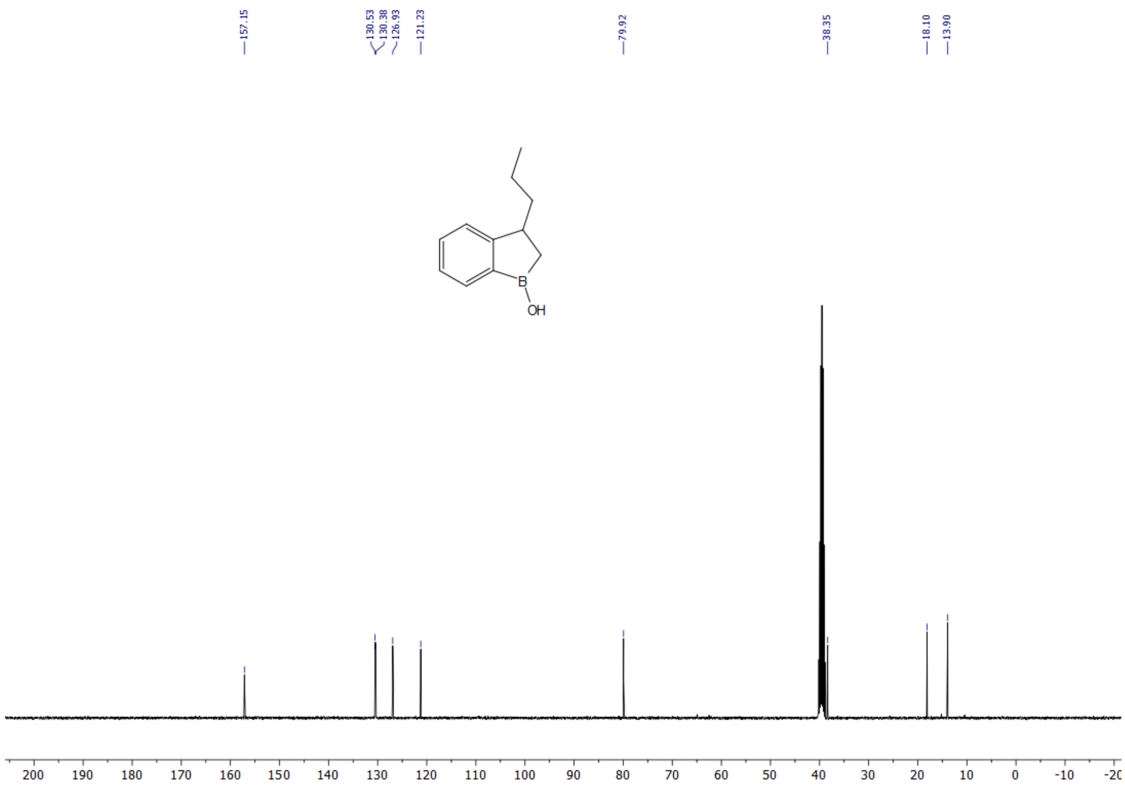

Figure S85: ^{13}C NMR (101 MHz,DMSO-d6, 298 K) spectrum of 3-propylbenzo[*c*][1,2]oxaborol-1(3*H*)-ol (5m).

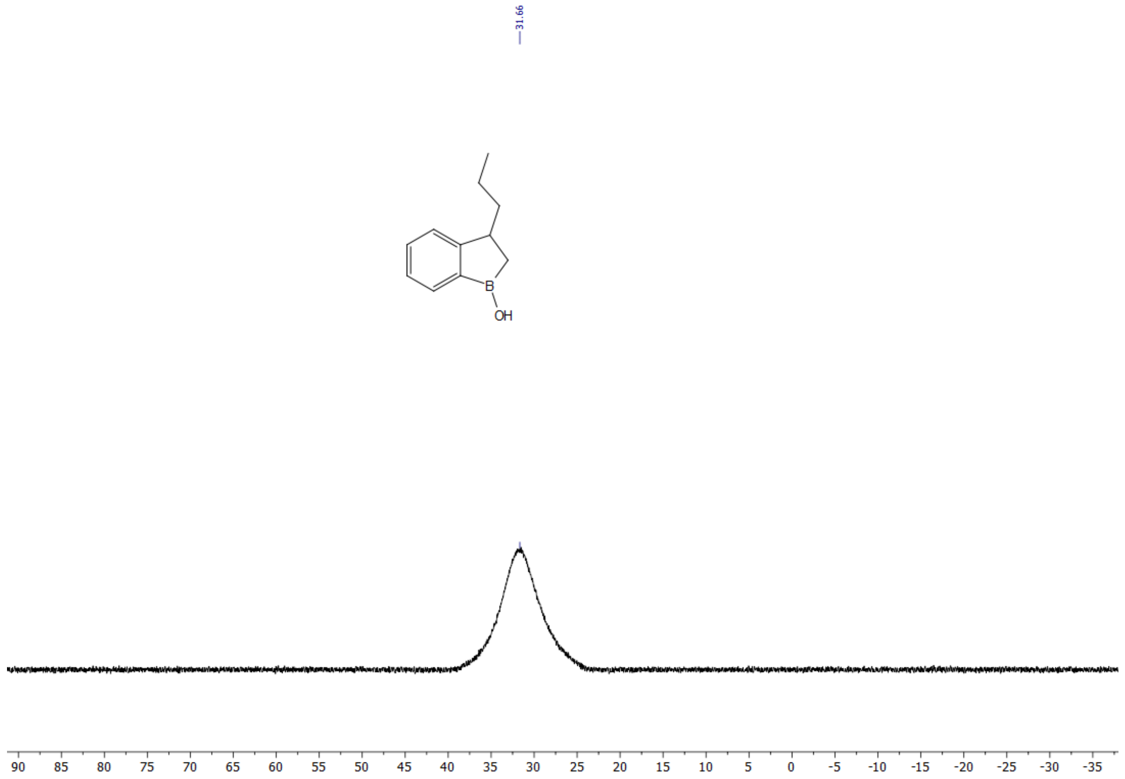

Figure S86: ^{11}B NMR (128 MHz,DMSO-d6, 298 K) spectrum of 3-propylbenzo[*c*][1,2]oxaborol-1(3*H*)-ol (5m).

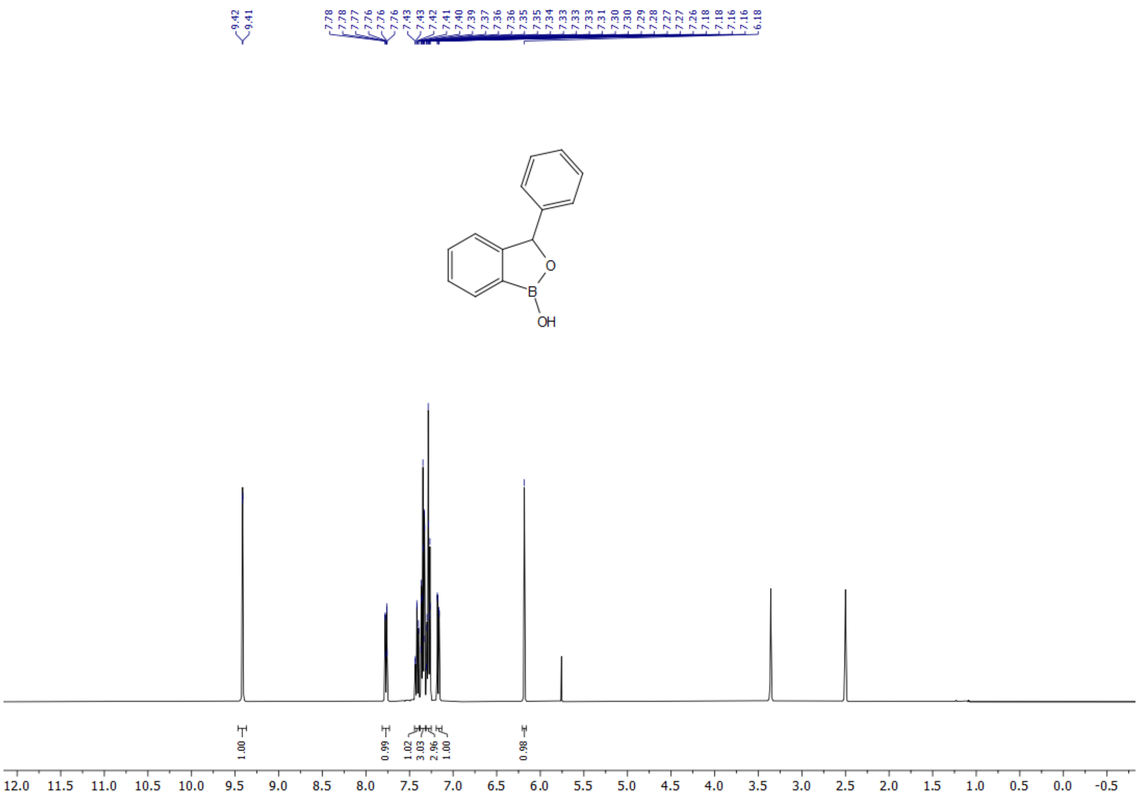

Figure S87: ^{1}H NMR (400 MHz,DMSO-d6, 298 K) spectrum of 3-phenylbenzo[*c*][1,2]oxaborol-1(3*H*)-ol (5n).

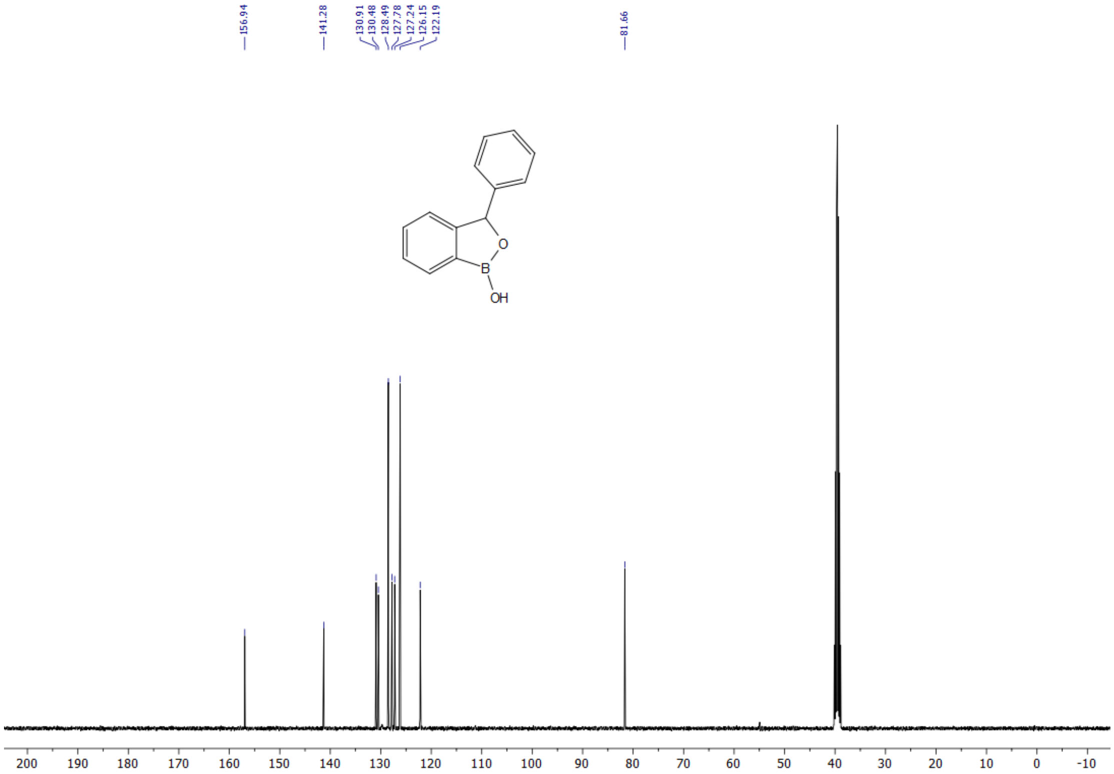

Figure S88: ^{13}C NMR (101 MHz,DMSO-d6, 298 K) spectrum of 3-phenylbenzo[*c*][1,2]oxaborol-1(3*H*)-ol (5n).

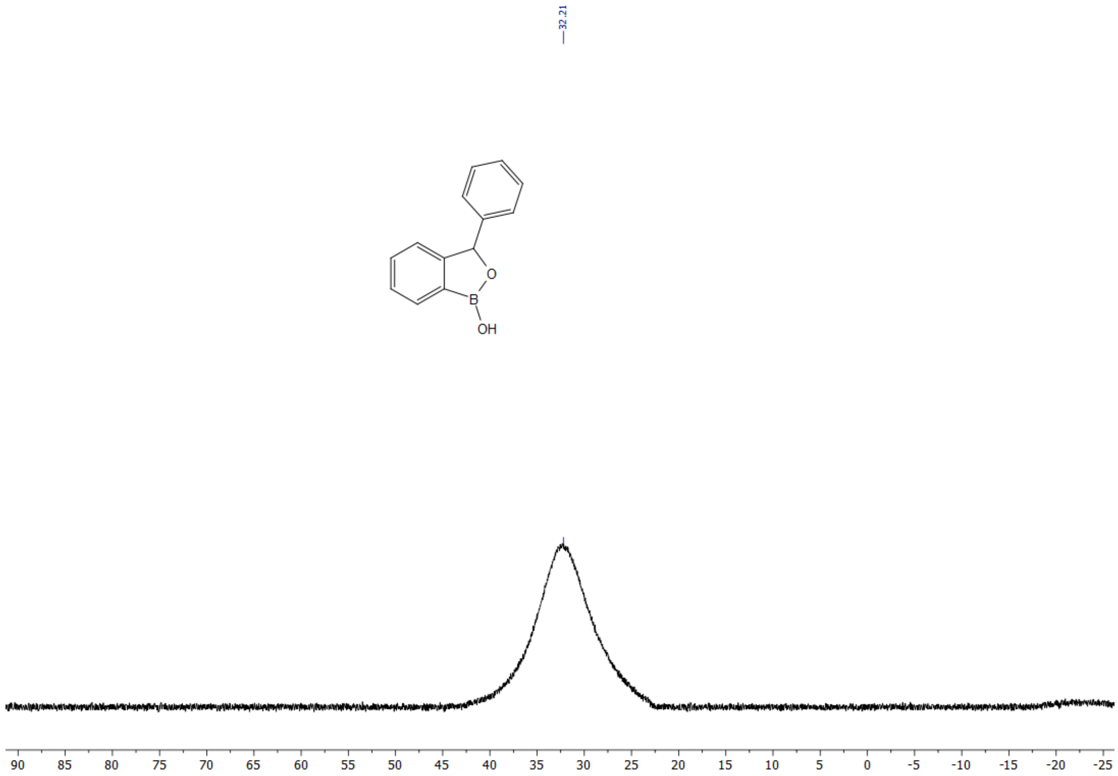

Figure S89: ^{11}B NMR (128 MHz,DMSO-d6, 298 K) spectrum of 3-phenylbenzo[*c*][1,2]oxaborol-1(3*H*)-ol (5n).

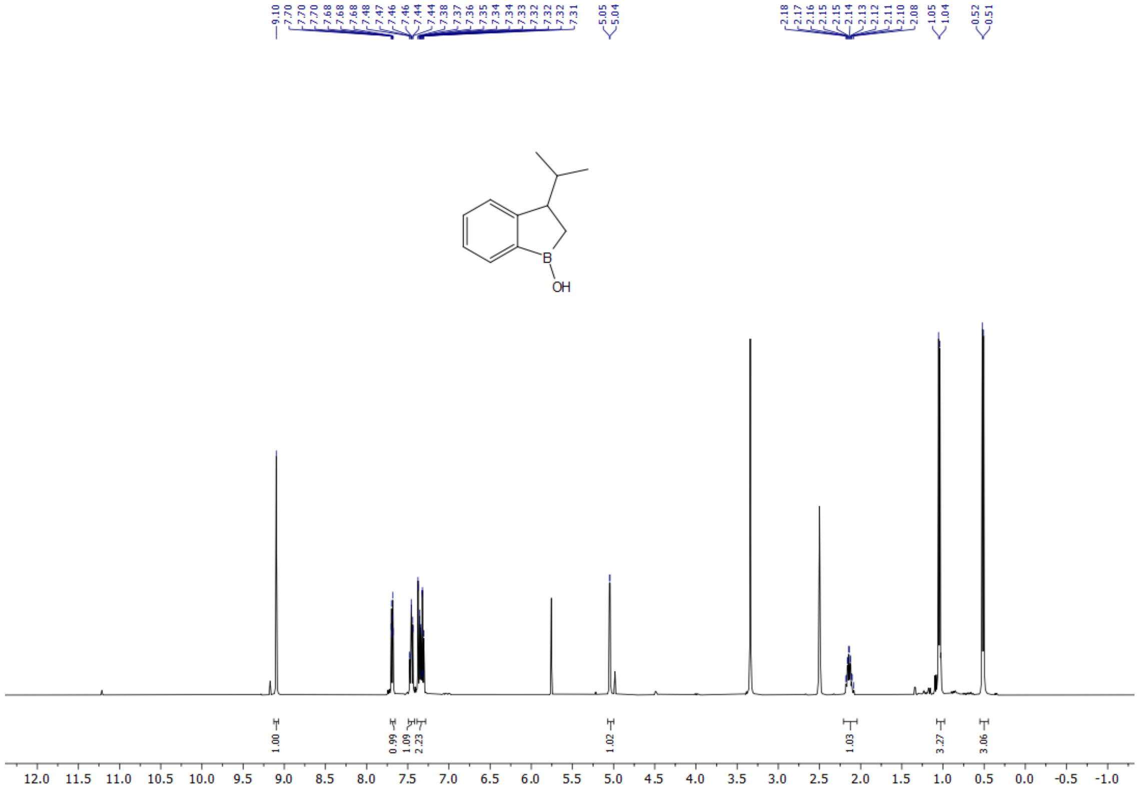

Figure S90: ^{1}H NMR (400 MHz,DMSO-d6, 298 K) spectrum of 3-isopropylbenzo[*c*][1,2]oxaborol-1(3*H*)-ol (5o).

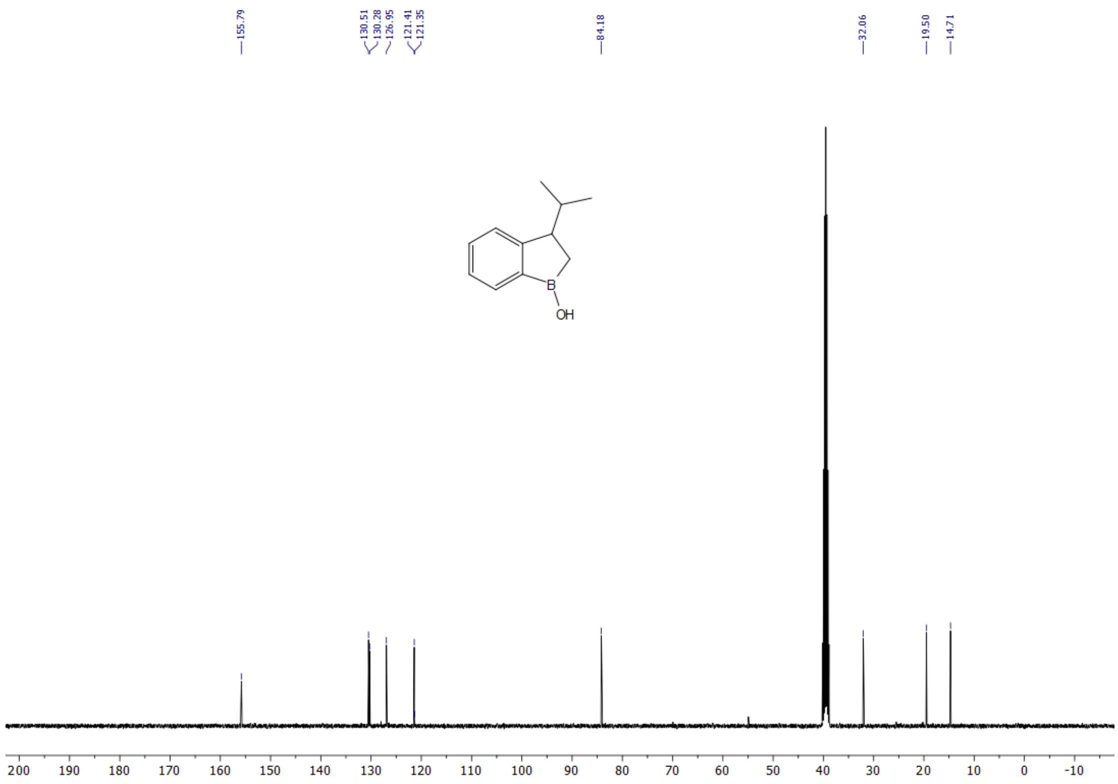

Figure S91: ^{13}C NMR (101 MHz,DMSO-d6, 298 K) spectrum of 3-isopropylbenzo[*c*][1,2]oxaborol-1(3*H*)-ol (5o).

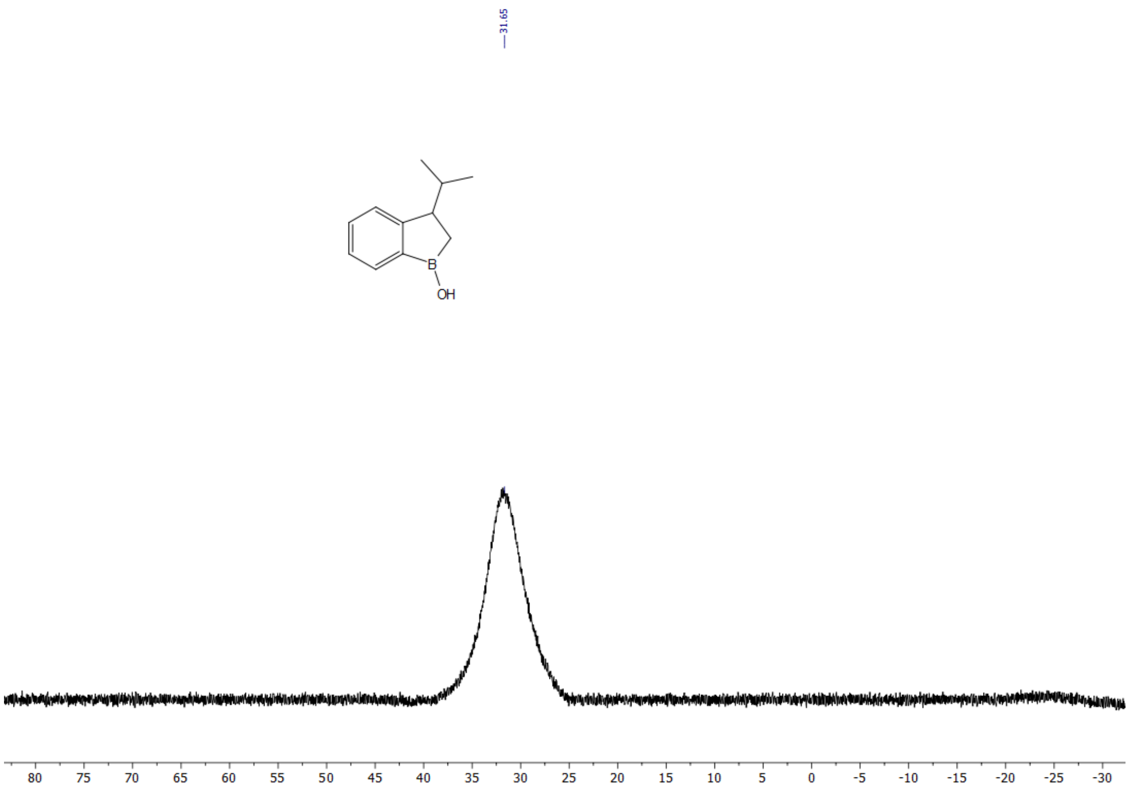

Figure S92: ^{11}B NMR (128 MHz,DMSO-d6, 298 K) spectrum of 3-isopropylbenzo[*c*][1,2]oxaborol-1(3*H*)-ol (5o).

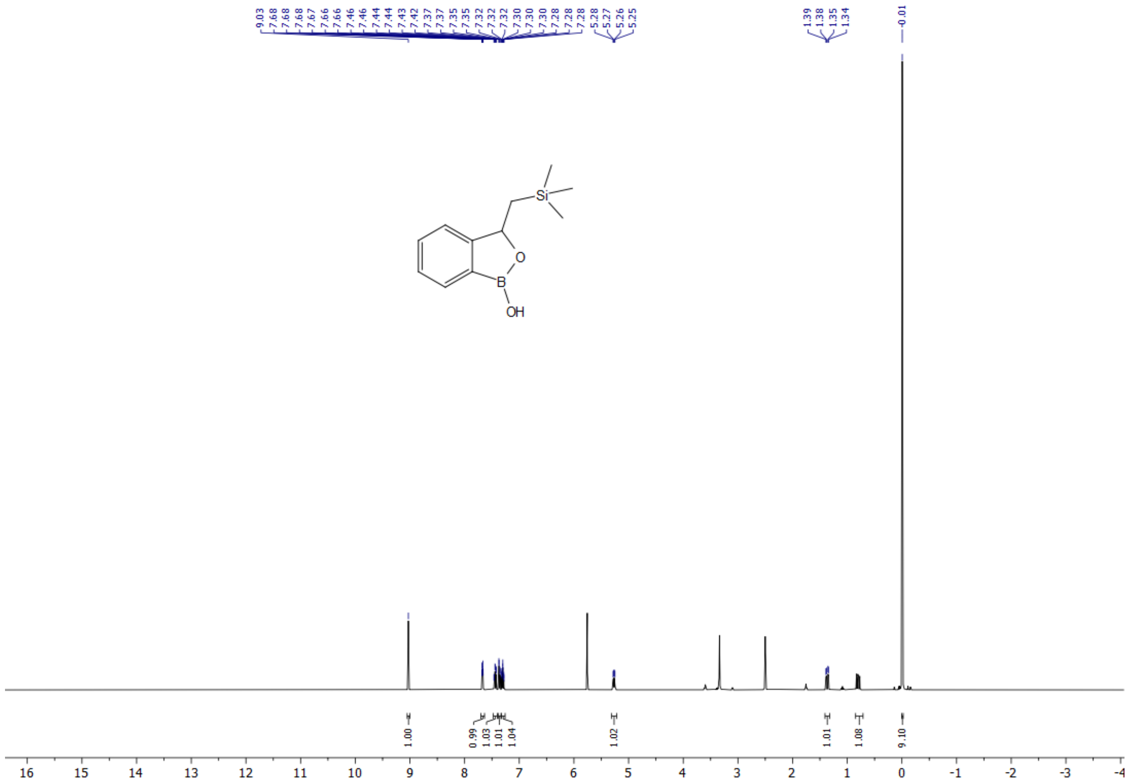

Figure S93: ^{1}H NMR (400 MHz,DMSO-d6, 298 K) spectrum of 3-((trimethylsilyl)methyl)benzo[*c*][1,2]oxaborol-1(3*H*)-ol (5p).

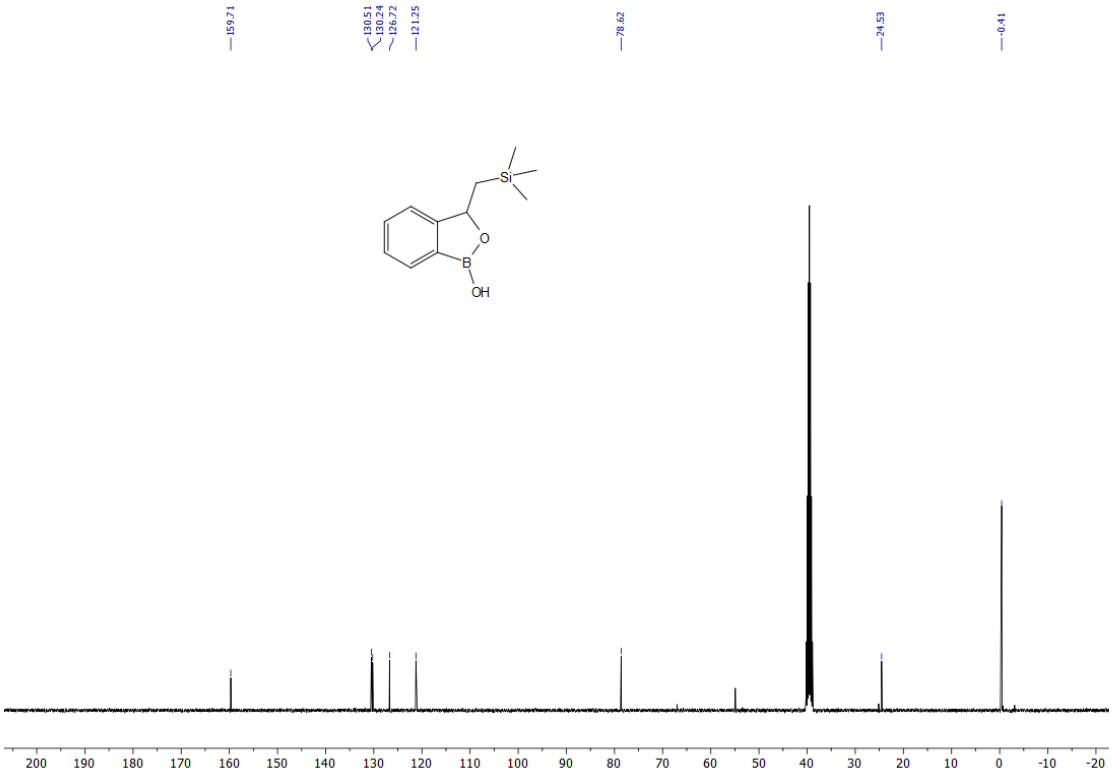

Figure S94: ^{13}C NMR (101 MHz,DMSO-d6, 298 K) spectrum of 3-((trimethylsilyl)methyl)benzo[*c*][1,2]oxaborol-1(3*H*)-ol (5p).

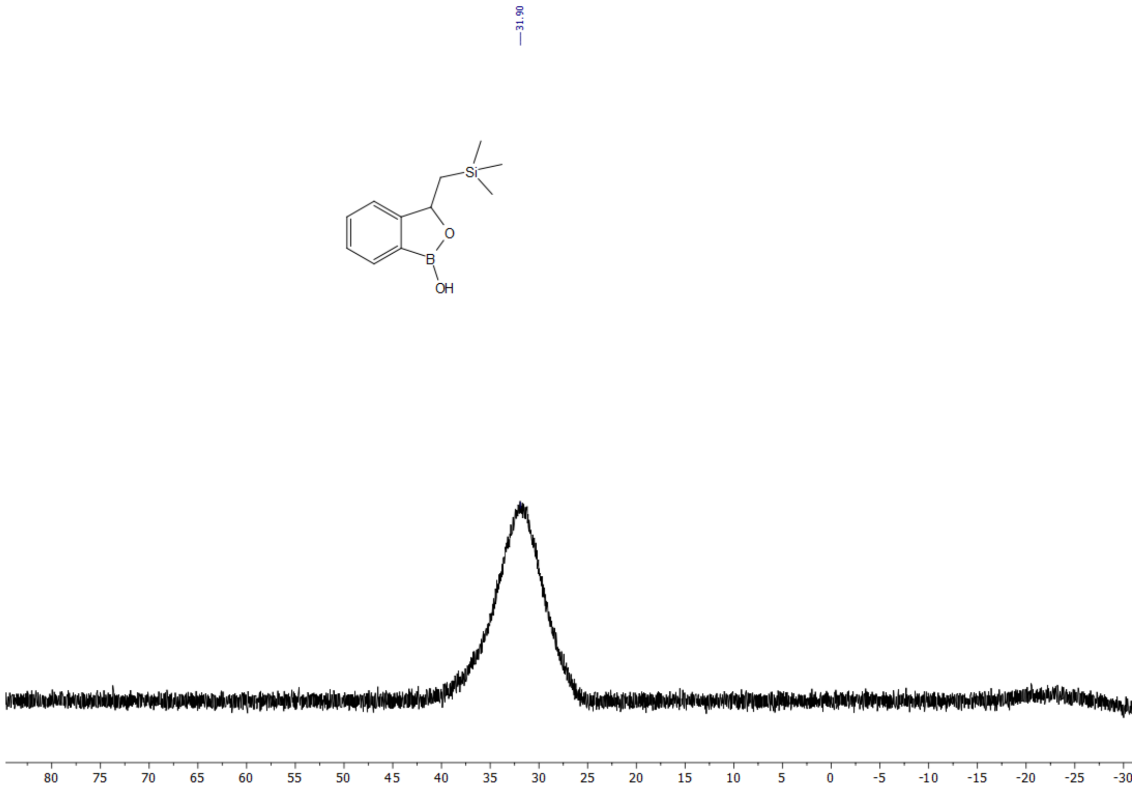

Figure S95: ^{11}B NMR (128 MHz,DMSO-d6, 298 K) spectrum of 3-((trimethylsilyl)methyl)benzo[*c*][1,2]oxaborol-1(3*H*)-ol (5p).

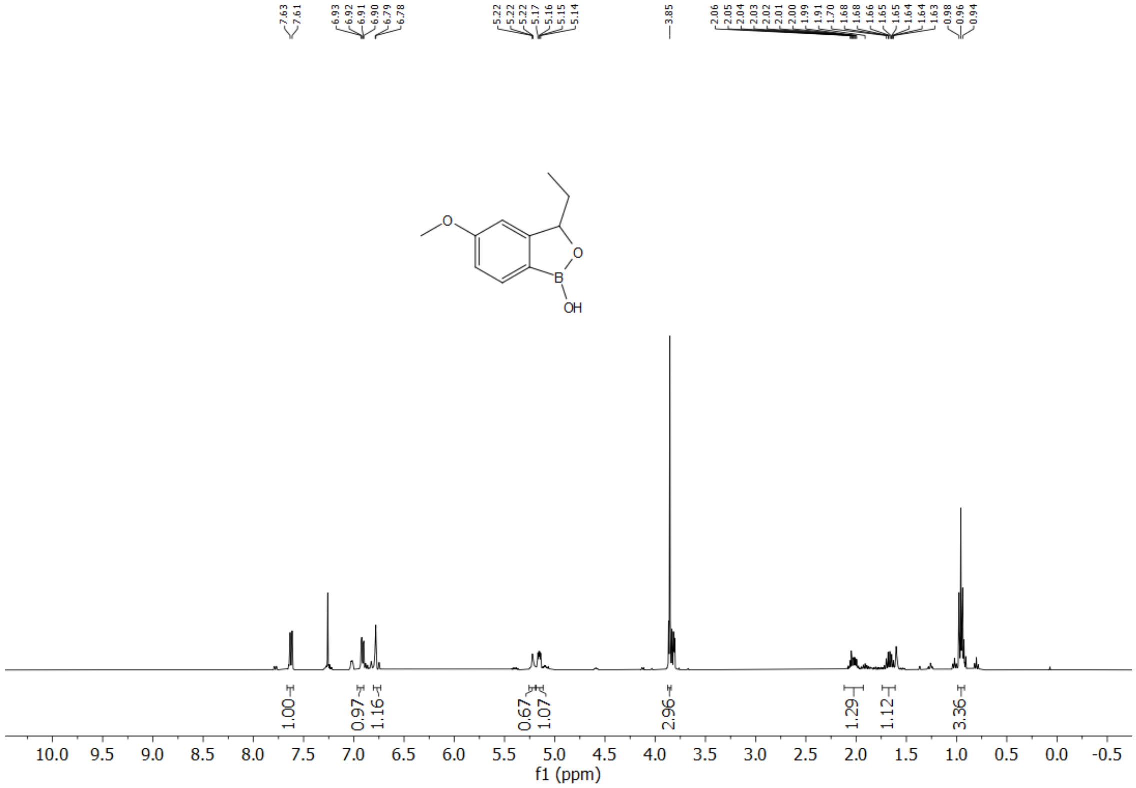

Figure S96: ^{1}H NMR (400 MHz, CDCl$_3$, 298 K) spectrum of 3-ethyl-5-methoxy-1,3-dihydrobenzo[*c*][1,2]oxaborole (5q).

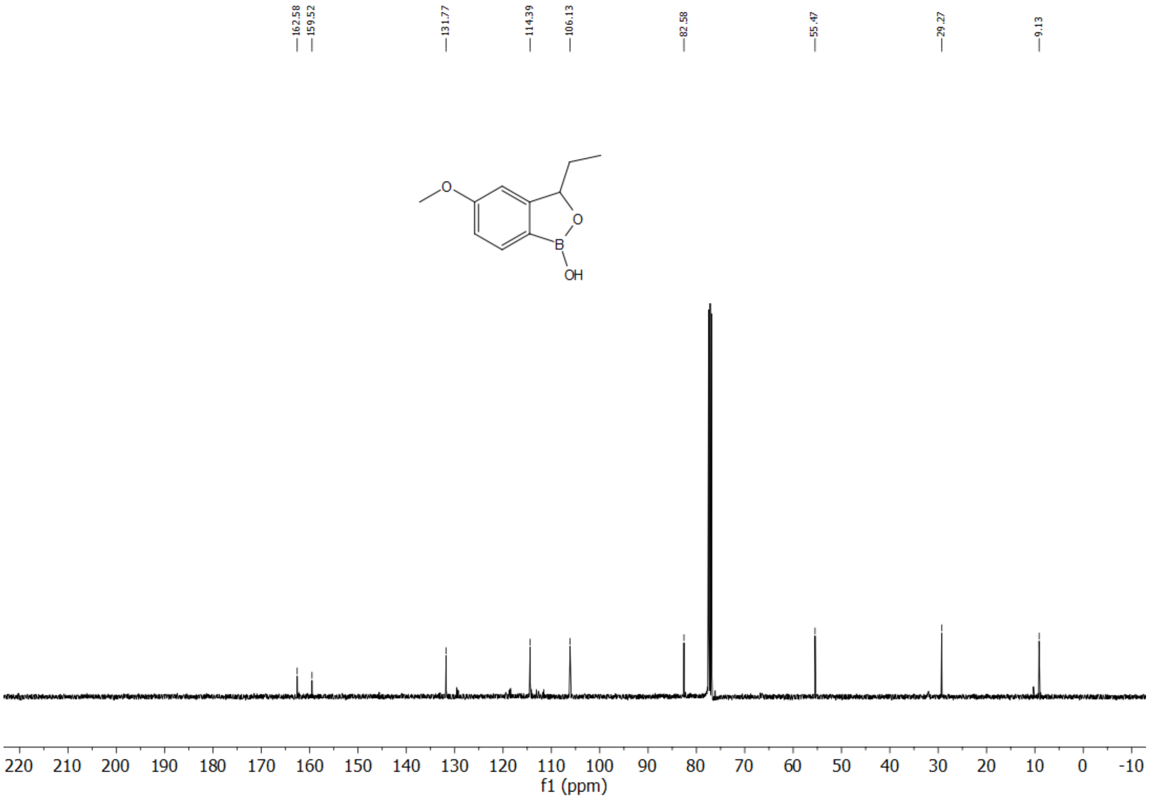

Figure S97: $^{13}C\{^{1}H\}$ NMR (101 MHz, $CDCl_3$, 298 K) spectrum of 3-ethyl-5-methoxy-1,3-dihydrobenzo[*c*][1,2]oxaborole (5q).

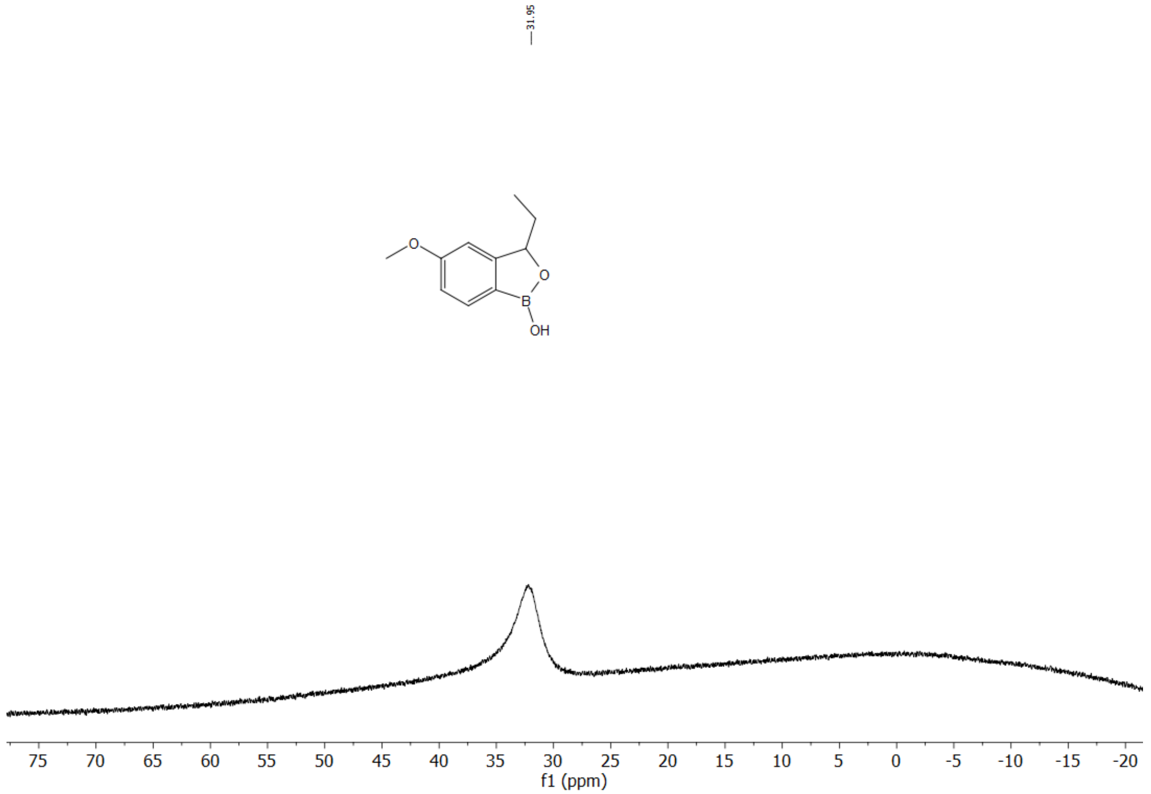

Figure S98: $^{11}B\{^{1}H\}$ NMR (128 MHz, $CDCl_3$, 298 K) spectrum of 3-ethyl-5-methoxy-1,3-dihydrobenzo[*c*][1,2]oxaborole (5q).

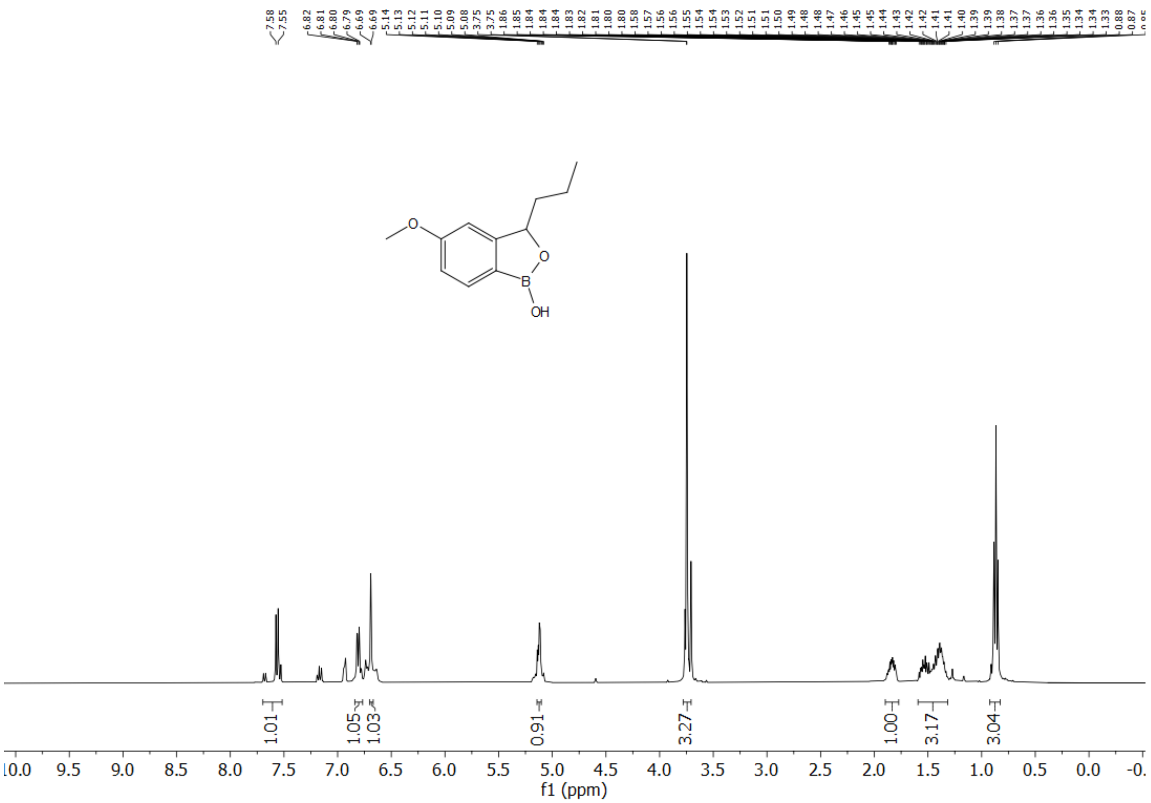

Figure S99: ^{1}H NMR (400 MHz, $CDCl_3$, 298 K) spectrum of 5-methoxy-3-propyl-1,3-dihydrobenzo[*c*][1,2]oxaborole (5r).

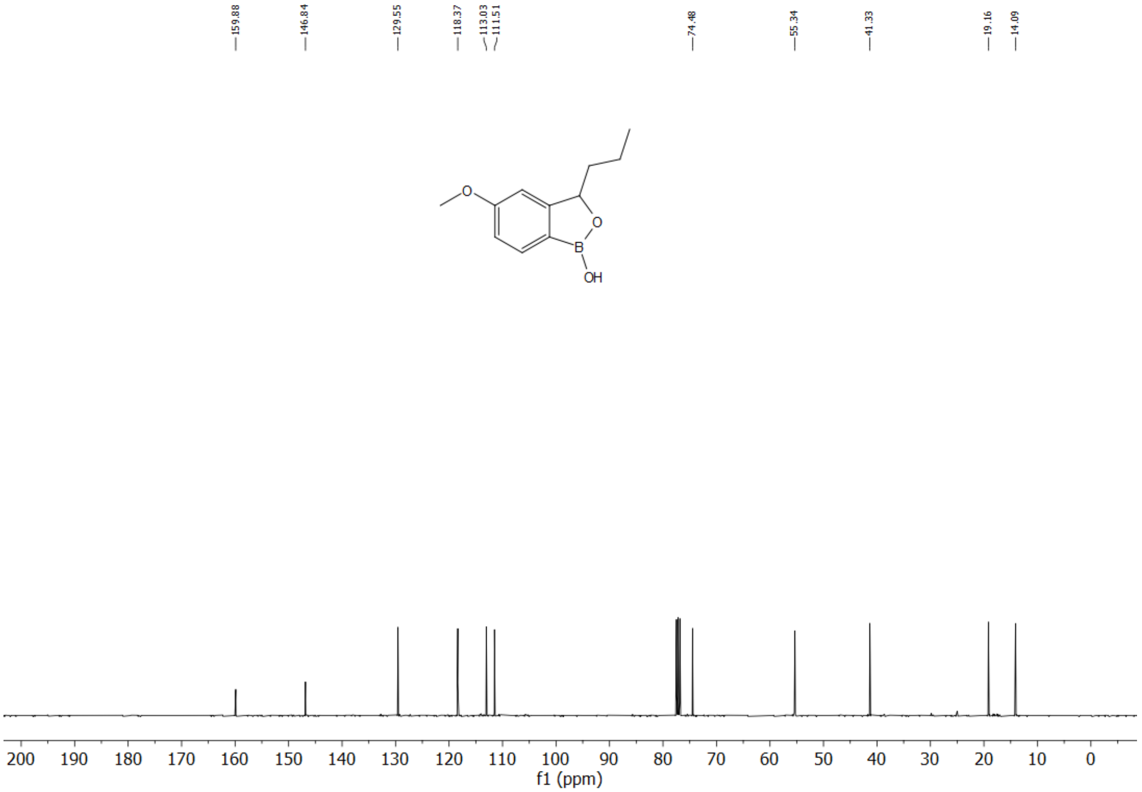

Figure S100: $^{13}C\{^{1}H\}$ NMR (101 MHz, $CDCl_3$, 298 K) spectrum of 5-methoxy-3-propyl-1,3-dihydrobenzo[*c*][1,2]oxaborole (5r).

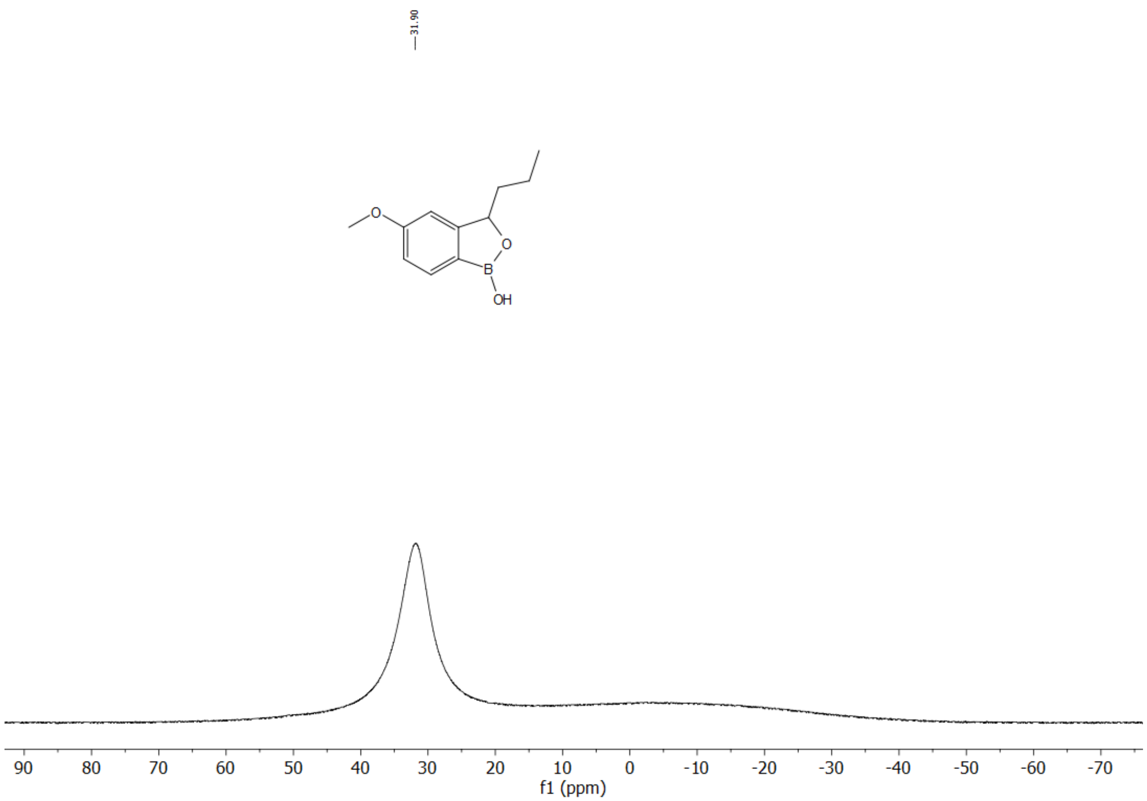

Figure S101: $^{11}B\{^{1}H\}$ NMR (128 MHz, $CDCl_3$, 298 K) spectrum of 5-methoxy-3-propyl-1,3-dihydrobenzo[*c*][1,2]oxaborole (5r).

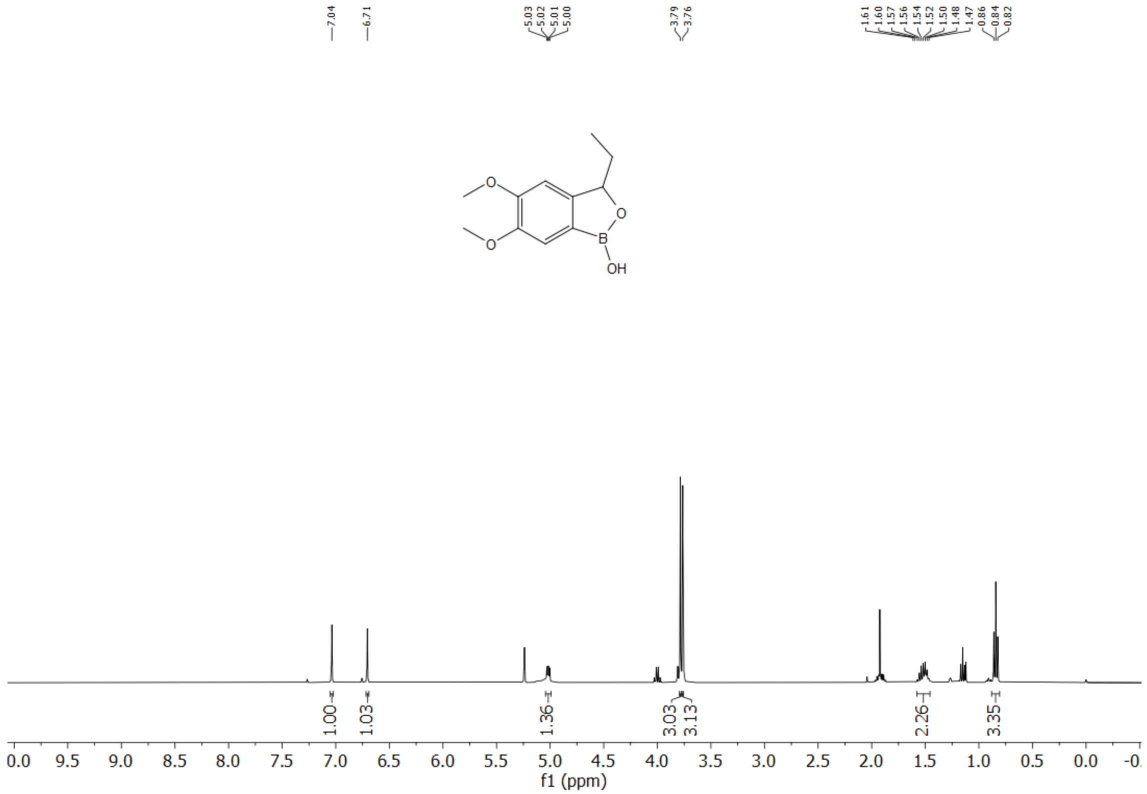

Figure S102: ^{1}H NMR (400 MHz, CD_2Cl_2, 298 K) spectrum of 3-ethyl-5,6-dimethoxybenzo[*c*][1,2]oxaborol-1(3*H*)-ol (5s).

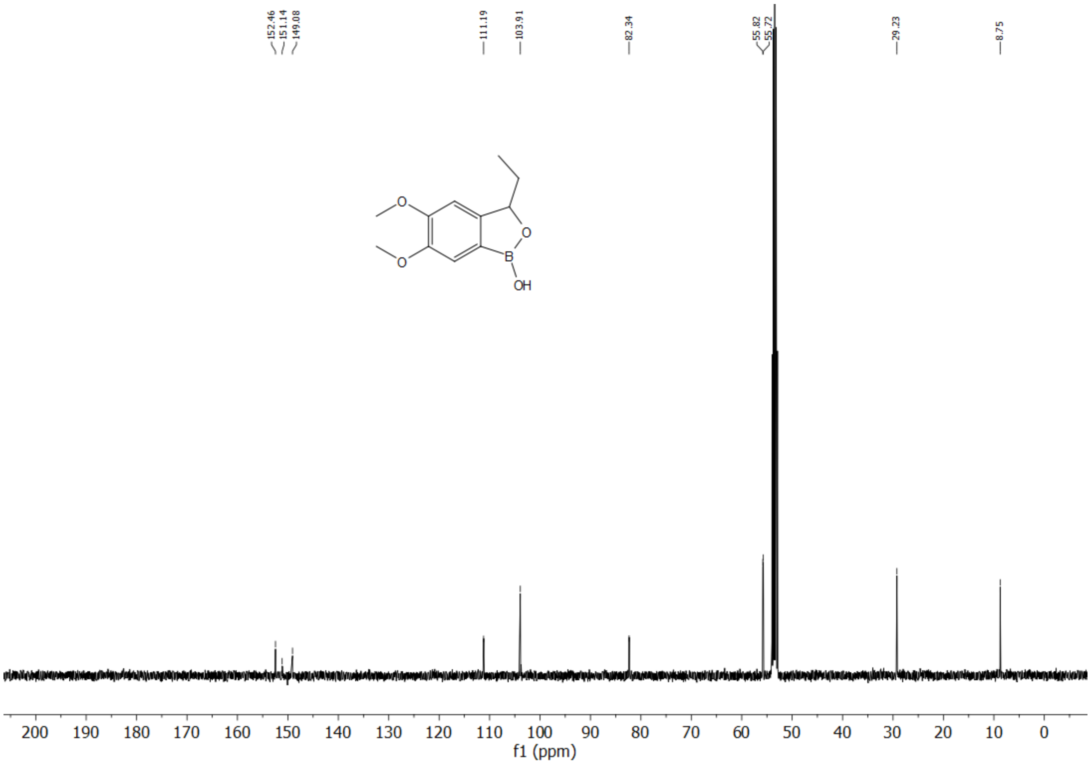

Figure S103: $^{13}C\{^{1}H\}$ NMR (101 MHz, CD_2Cl_2, 298 K) spectrum of 3-ethyl-5,6-dimethoxybenzo[*c*][1,2]oxaborol-1(3*H*)-ol (5s).

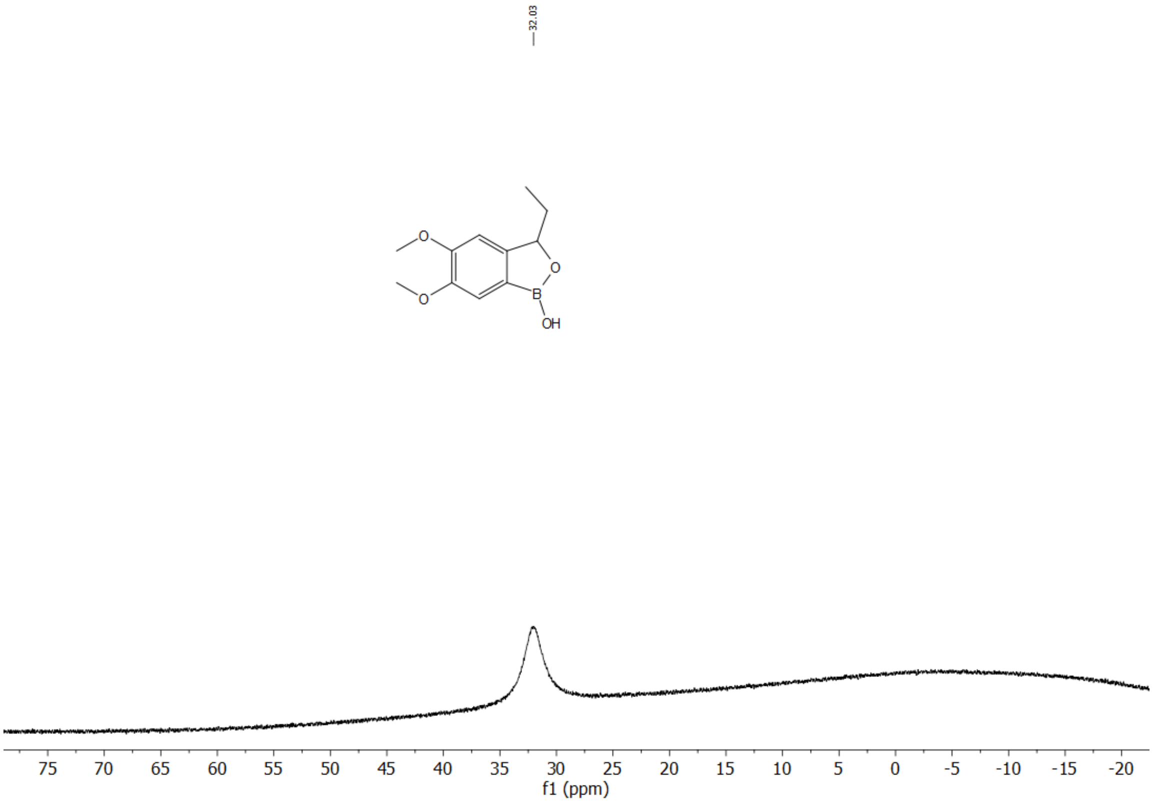

Figure S104: $^{11}B\{^{1}H\}$ NMR (128 MHz, CD_2Cl_2, 298 K) spectrum of 3-ethyl-5,6-dimethoxybenzo[*c*][1,2]oxaborol-1(3*H*)-ol (5s).

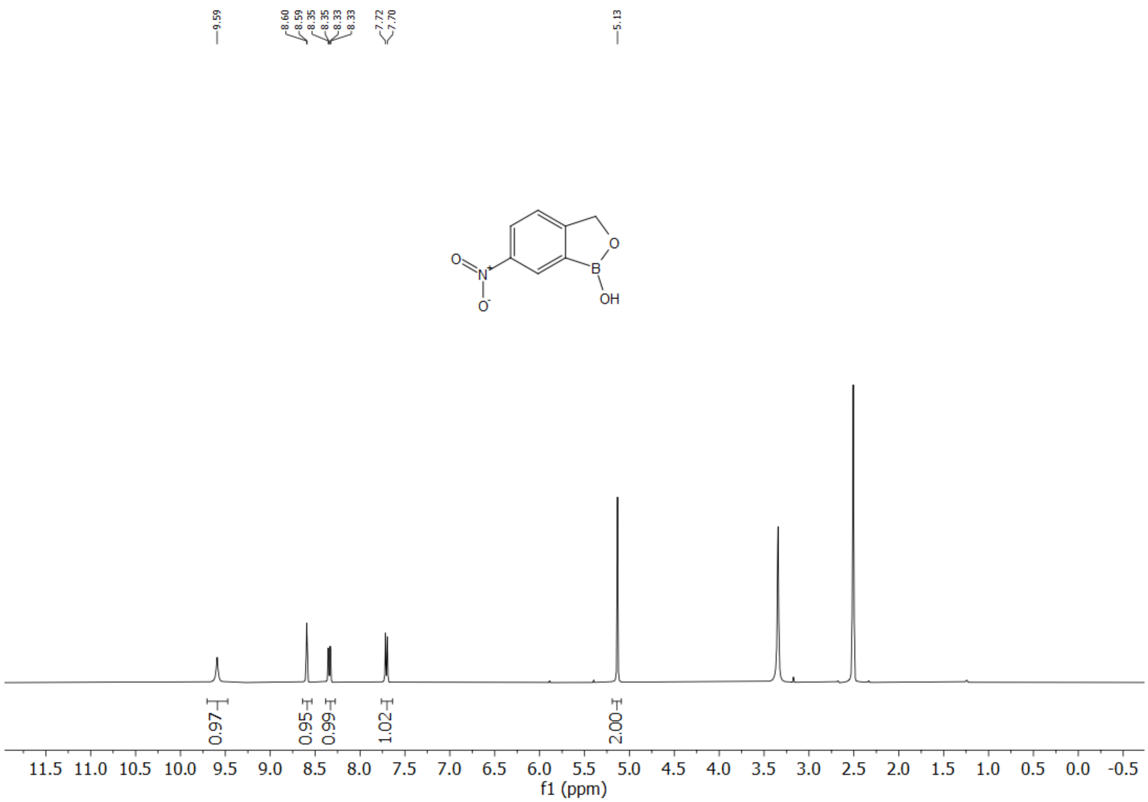

Figure S105: ^{1}H NMR (400 MHz, DMSO-d$_6$, 298 K) spectrum of 6-nitrobenzo[*c*][1,2]oxaborol-1(3*H*)-ol.

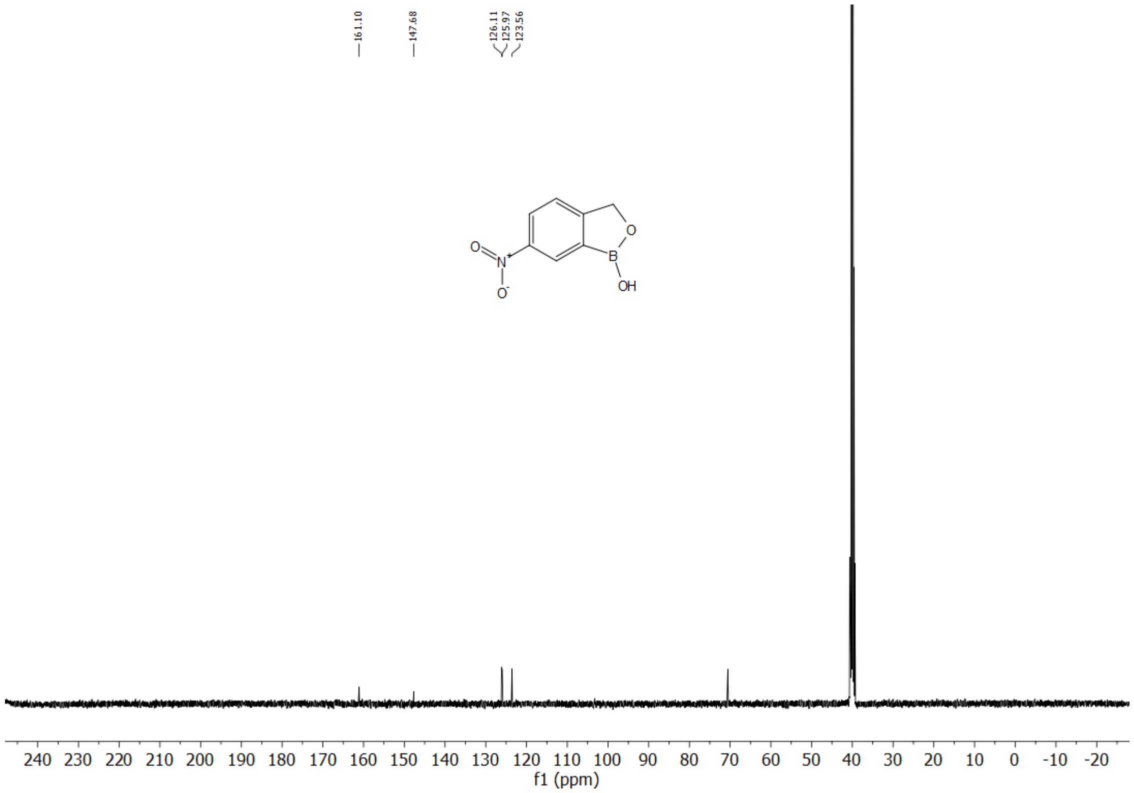

Figure S106: ^{13}C{^{1}H} NMR (101 MHz, DMSO-d6, 298 K) spectrum of 6-nitrobenzo[*c*][1,2]oxaborol-1(3*H*)-ol.

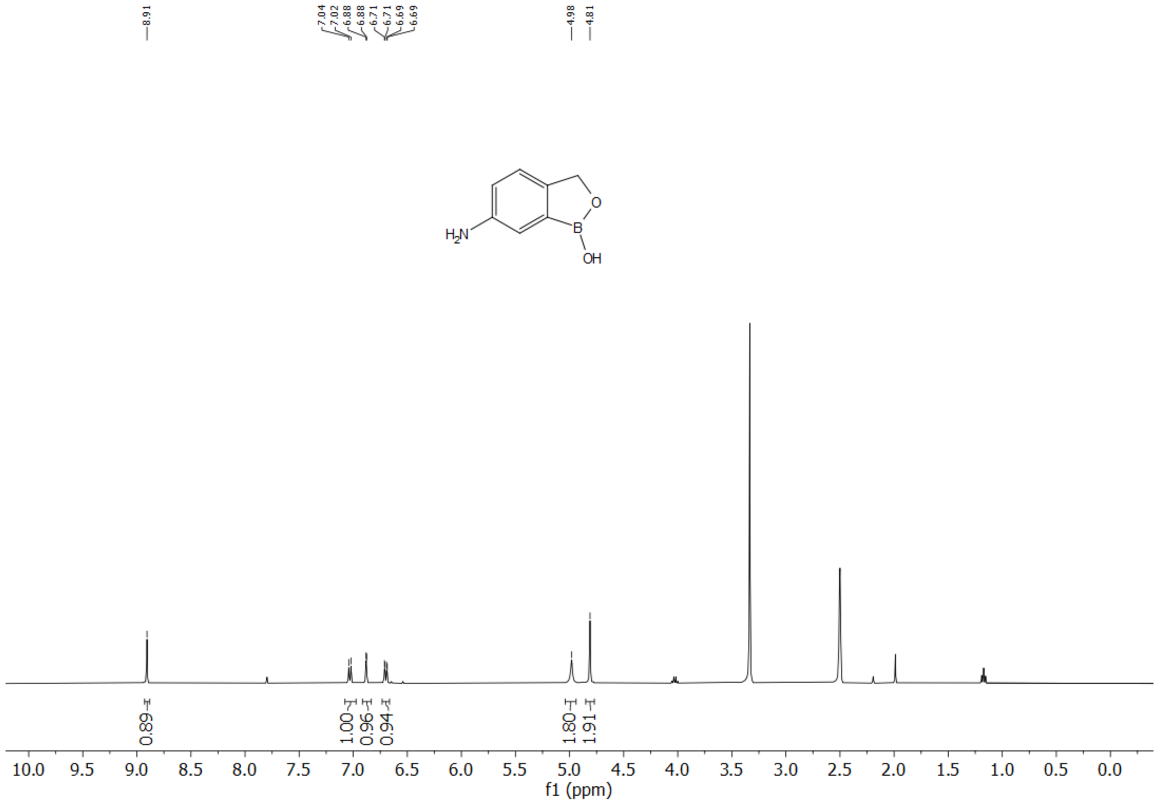

Figure S107: ^{1}H NMR (400 MHz, DMSO-d_6, 298 K) spectrum of 6-aminobenzo[*c*][1,2]oxaborol-1(3*H*)-ol.

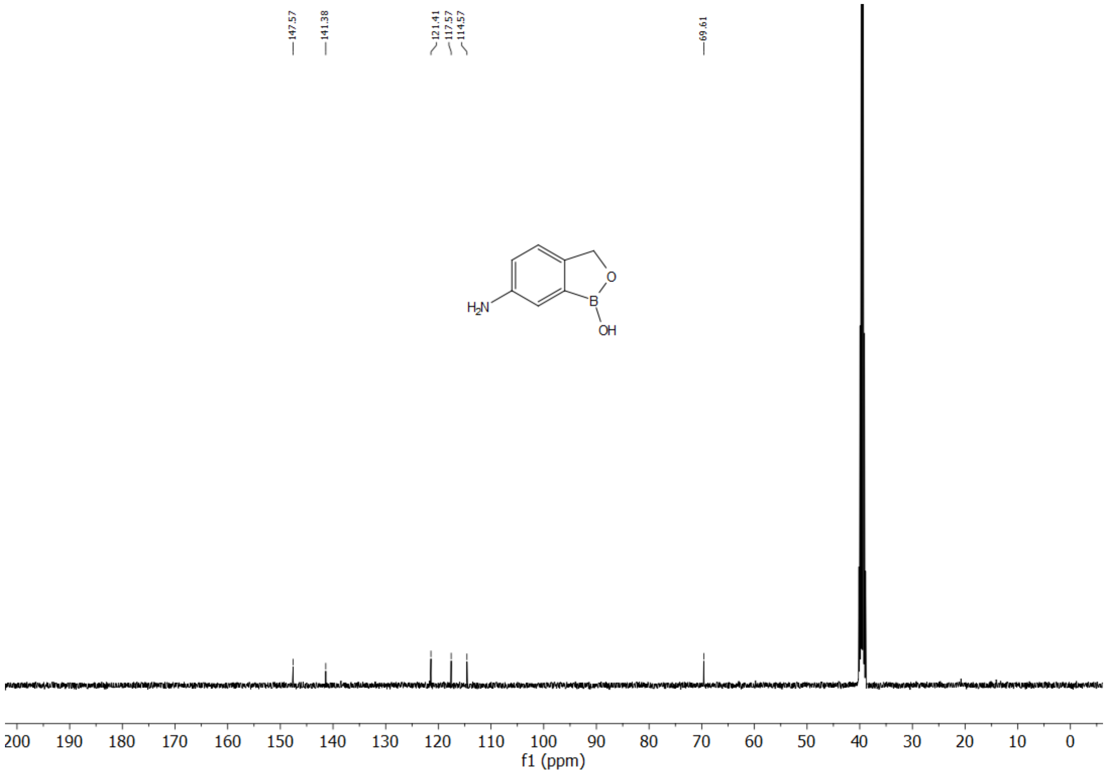

Figure S108: $^{13}C\{^{1}H\}$ NMR (101 MHz, DMSO-d6, 298 K) spectrum of 6-aminobenzo[*c*][1,2]oxaborol-1(3*H*)-ol.

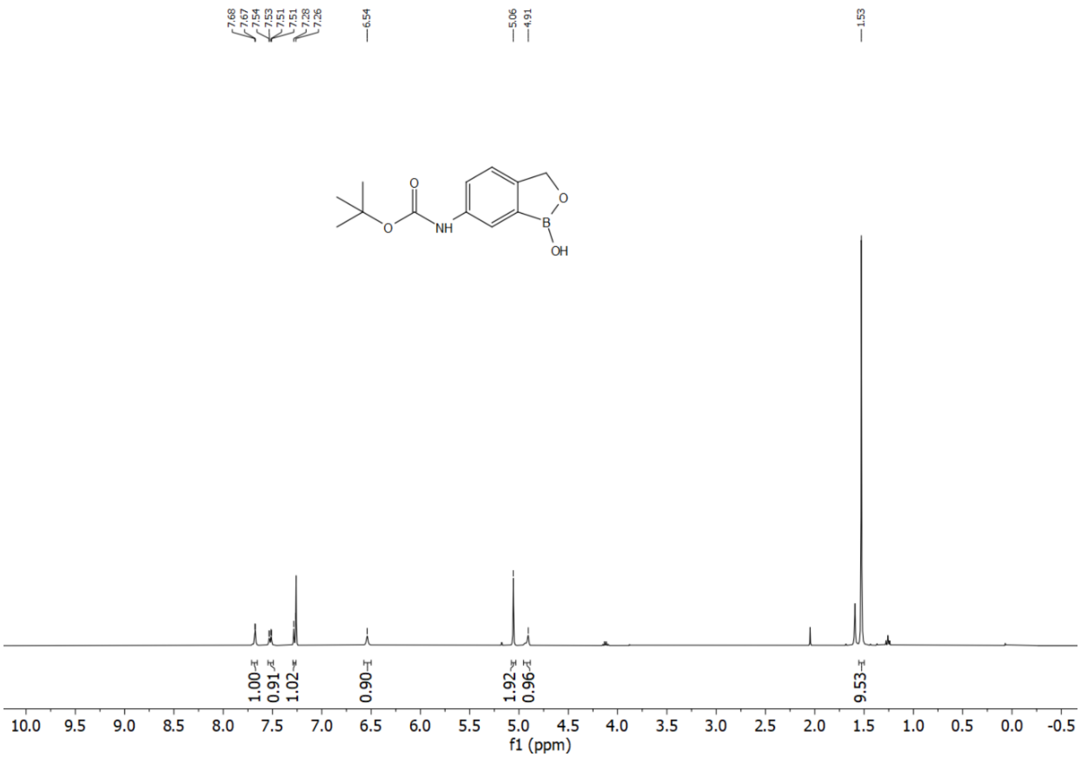

Figure S109: ^{1}H NMR (400 MHz, $CDCl_3$, 298 K) spectrum of *tert*-butyl (1-hydroxy-1,3-dihydrobenzo[*c*][1,2]oxaborol-6-yl)carbamate (5g).

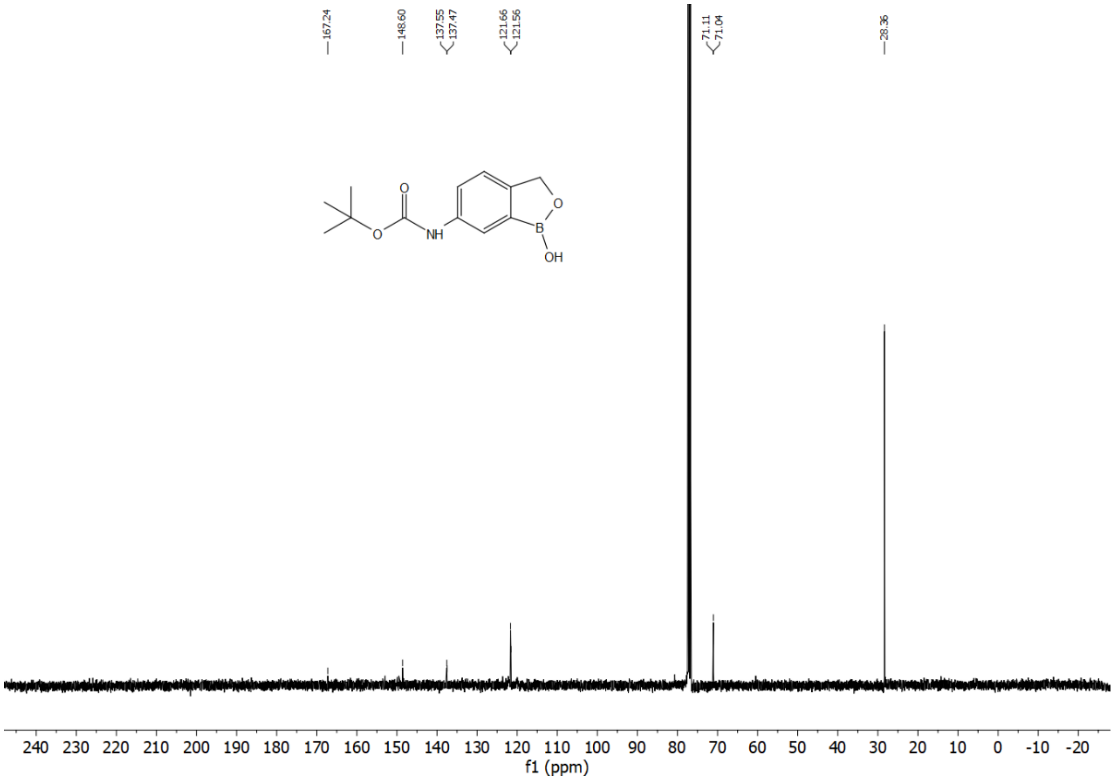

Figure S110: $^{13}C\{^{1}H\}$ NMR (101 MHz, $CDCl_3$, 298 K) spectrum of *tert*-butyl (1-hydroxy-1,3-dihydrobenzo[*c*][1,2]oxaborol-6-yl)carbamate (5g).

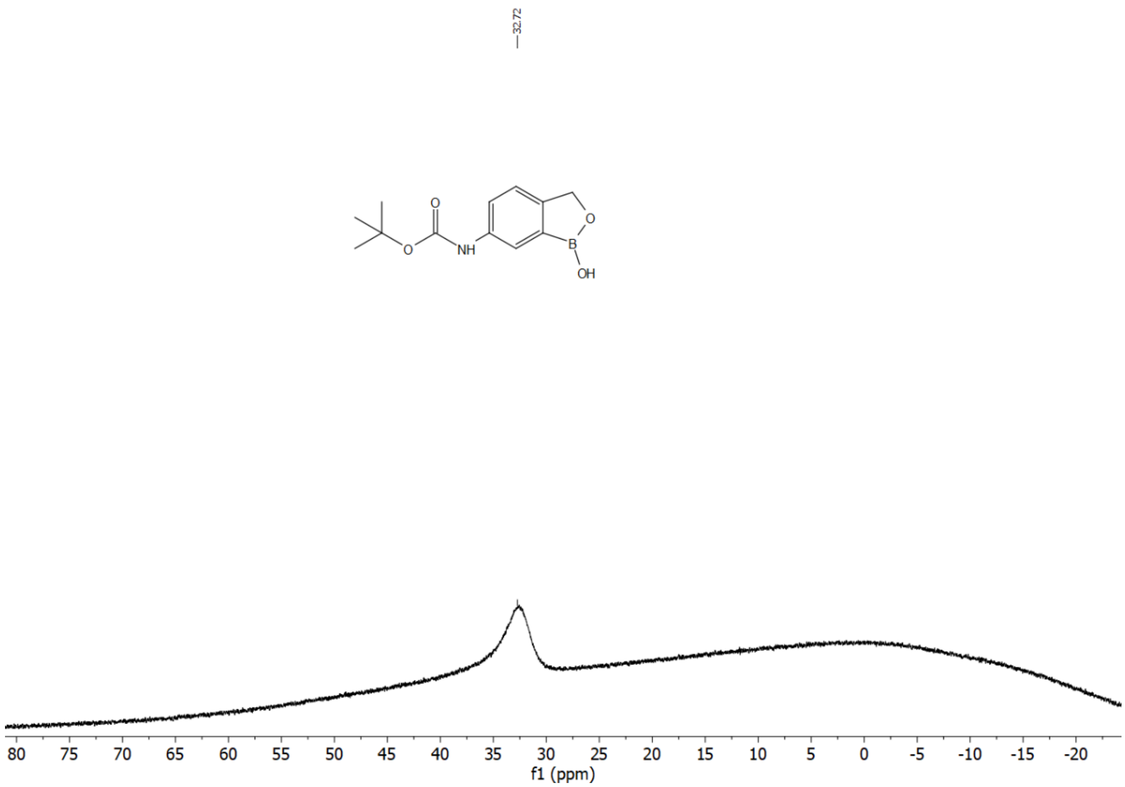

Figure S111: $^{11}B\{^{1}H\}$ NMR (128 MHz, $CDCl_3$, 298 K) spectrum of *tert*-butyl (1-hydroxy-1,3-dihydrobenzo[*c*][1,2]oxaborol-6-yl)carbamate (5g).

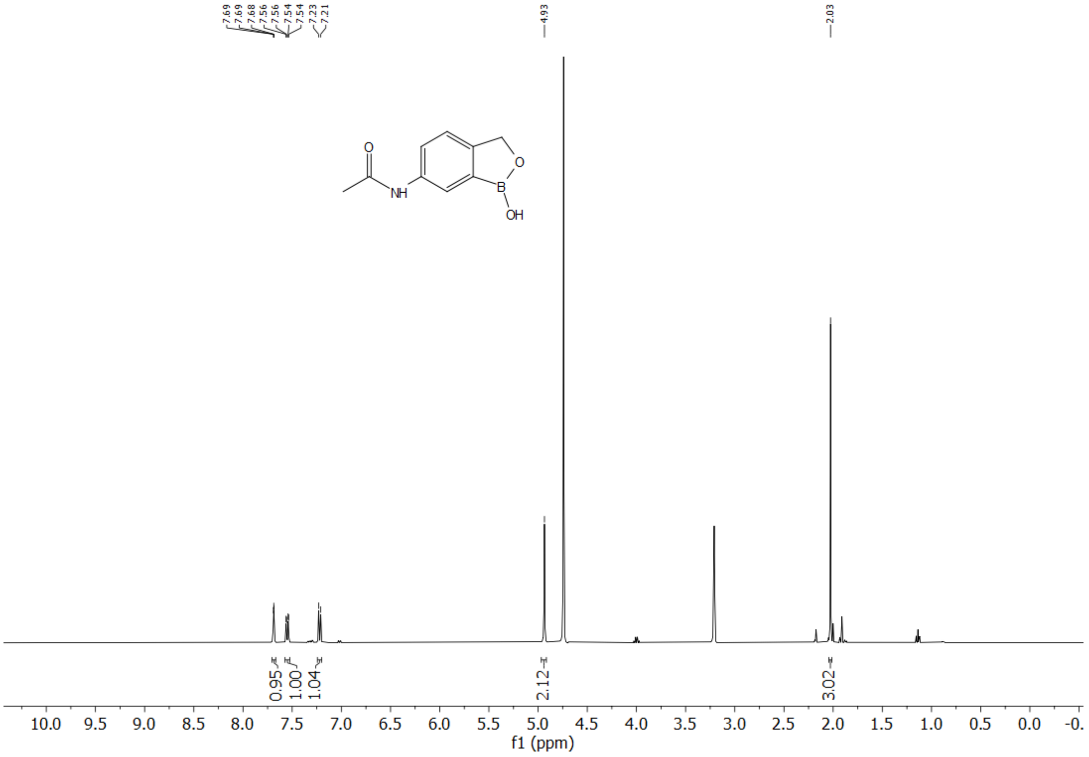

Figure S112: ^{1}H NMR (400 MHz, CD_3OD, 298 K) spectrum of *N*-(1-hydroxy-1,3-dihydrobenzo[*c*][1,2]oxaborol-6-yl)acetamide (5x).

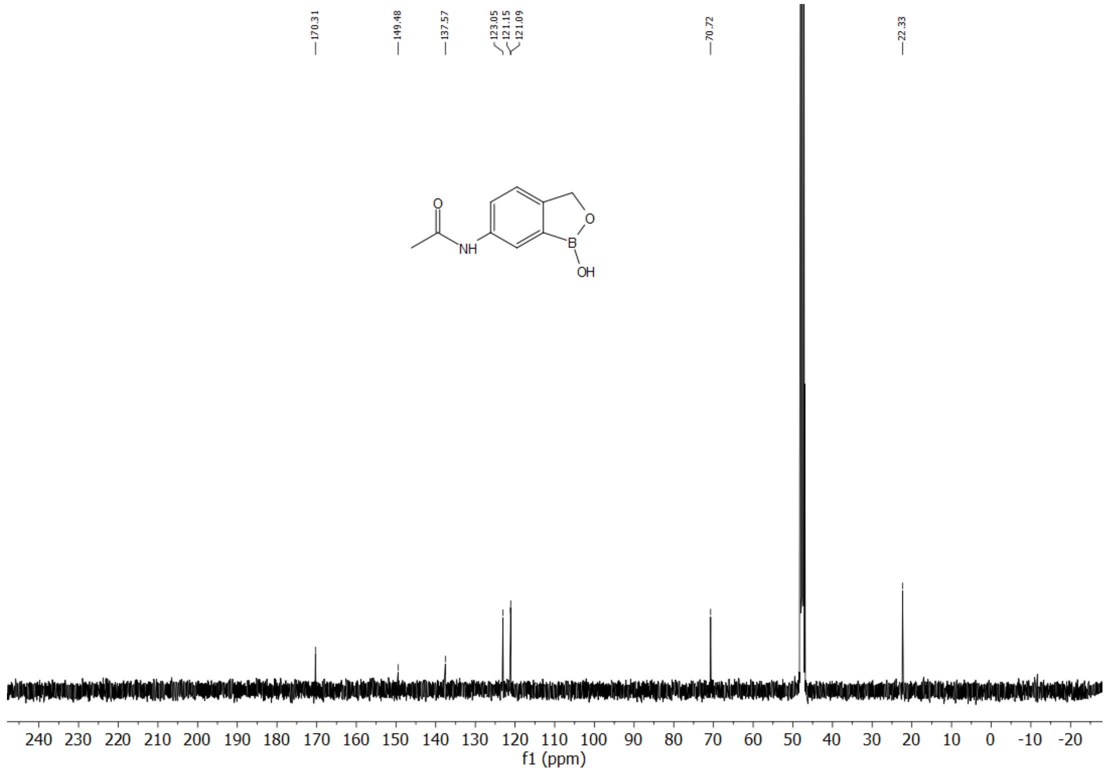

Figure S113: $^{13}C\{^{1}H\}$ NMR (101 MHz, CD_3OD, 298 K) spectrum of *N*-(1-hydroxy-1,3-dihydrobenzo[*c*][1,2]oxaborol-6-yl)acetamide (5x).

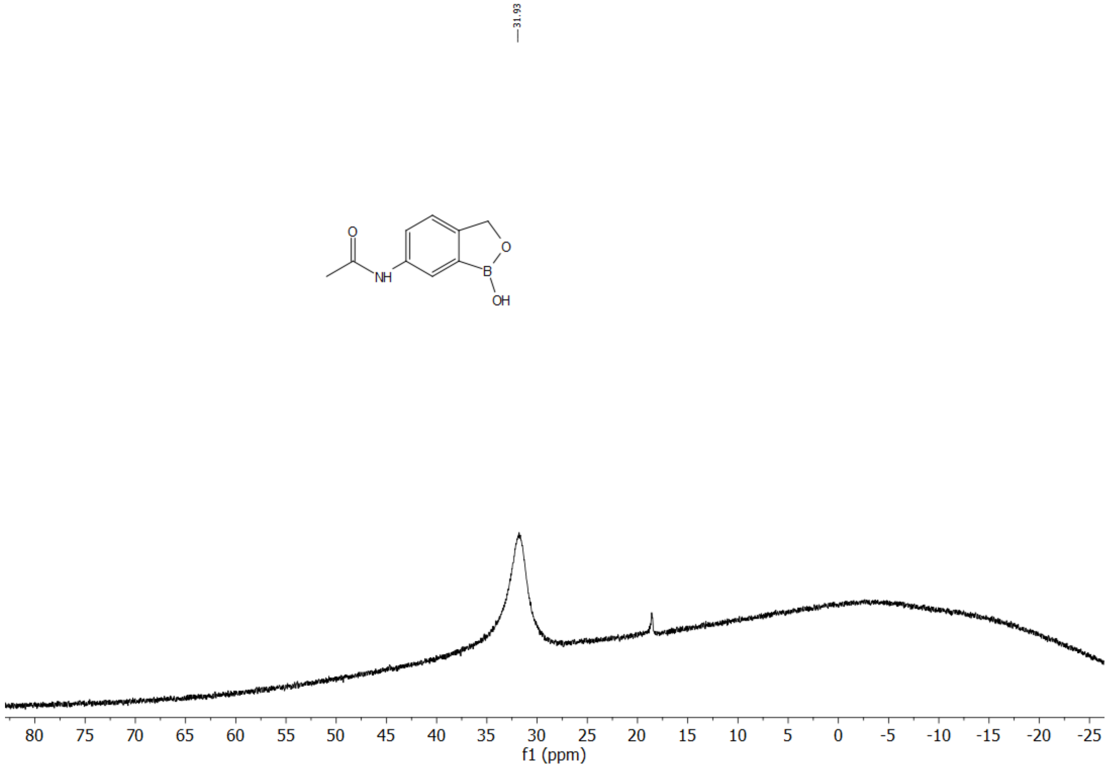

Figure S114: $^{11}B\{^{1}H\}$ NMR (128 MHz, CD_3OD, 298 K) spectrum of *N*-(1-hydroxy-1,3-dihydrobenzo[c][1,2]oxaborol-6-yl)acetamide (5x).

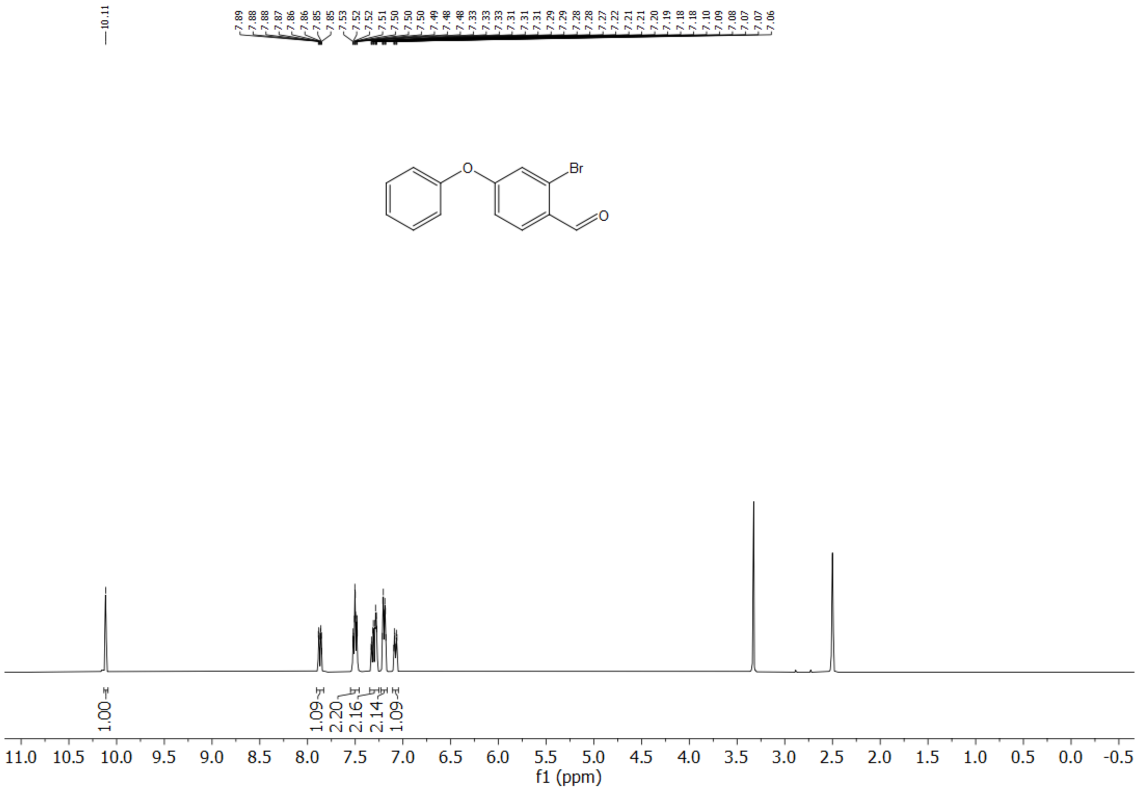

Figure S115: ^{1}H NMR (400 MHz, DMSO-d6, 298 K) spectrum of 2-bromo-4-phenoxybenzaldehyde.

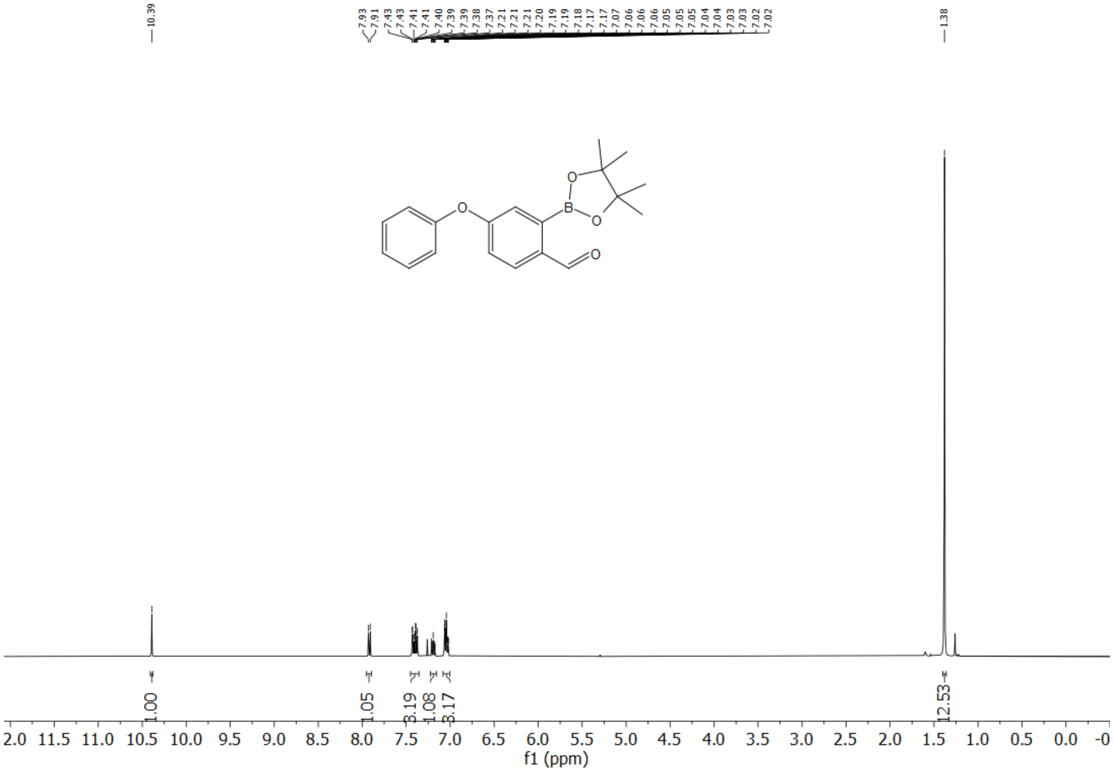

Figure S116: ^{1}H NMR (400 MHz, CDCl$_3$, 298 K) spectrum of 4-phenoxy-2-(4,4,5,5-tetramethyl-1,3,2-dioxaborolan-2-yl)benzaldehyde.

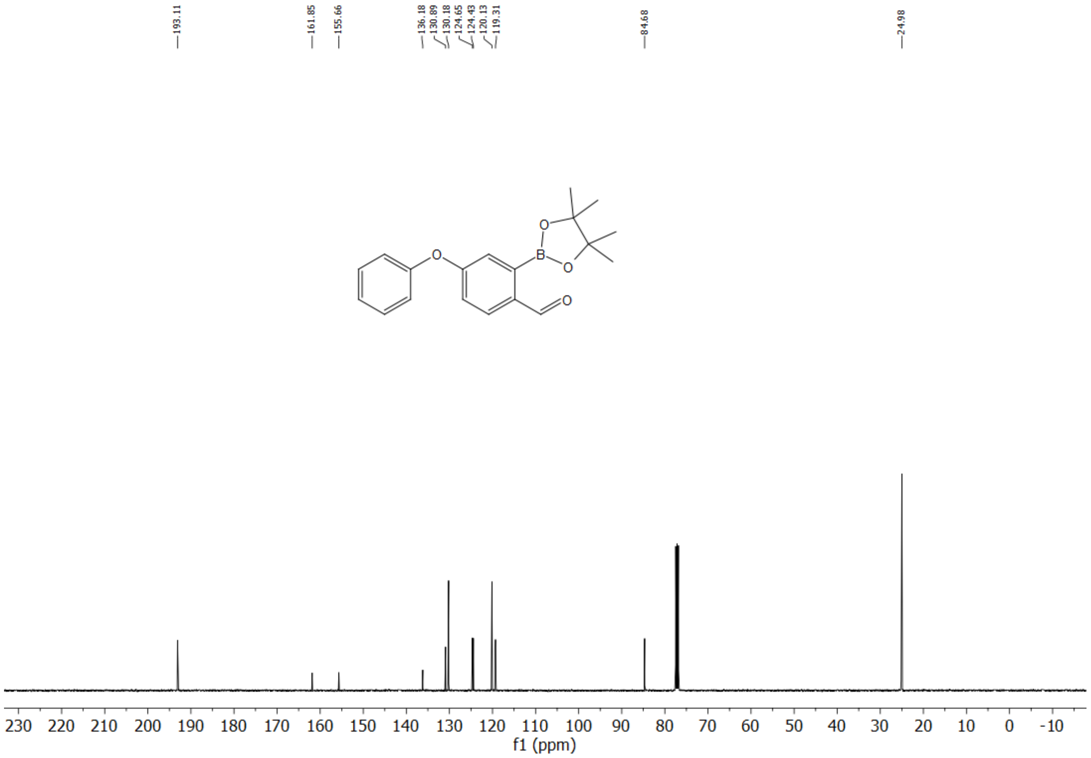

Figure S117: $^{13}C\{^{1}H\}$ NMR (101 MHz, $CDCl_3$, 298 K) spectrum of 4-phenoxy-2-(4,4,5,5-tetramethyl-1,3,2-dioxaborolan-2-yl)benzaldehyde.

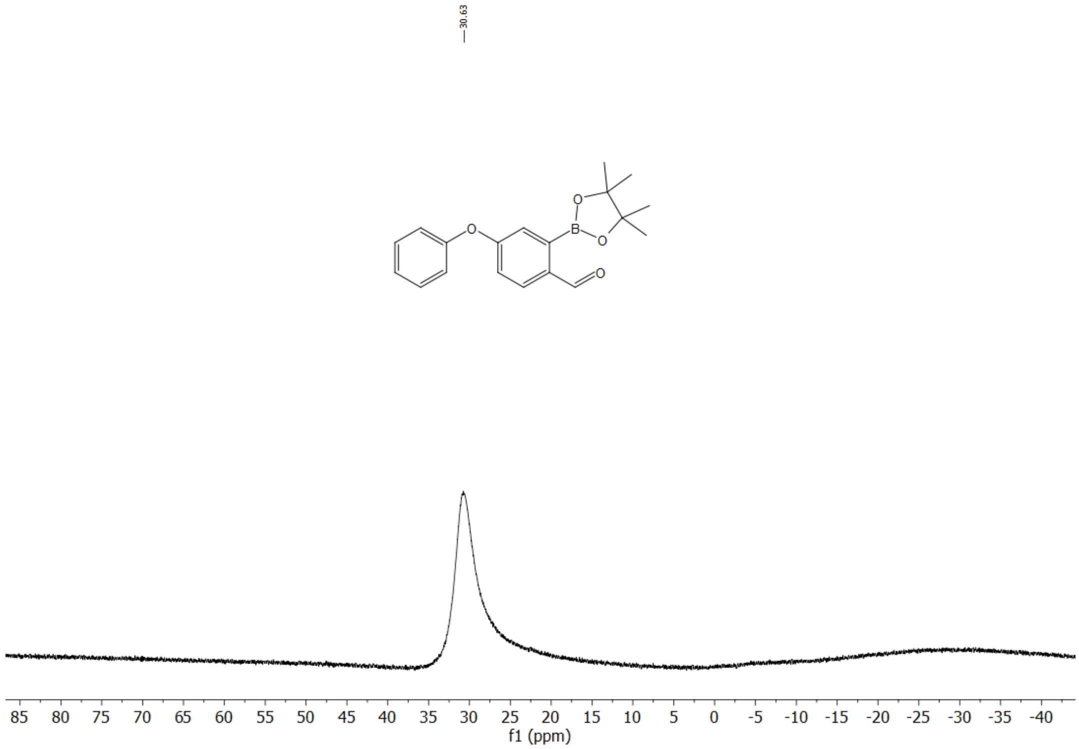

Figure S118: $^{11}B\{^{1}H\}$ NMR (128 MHz, CD_2Cl_2, 298 K) spectrum of of 4-phenoxy-2-(4,4,5,5-tetramethyl-1,3,2-dioxaborolan-2-yl)benzaldehyde.

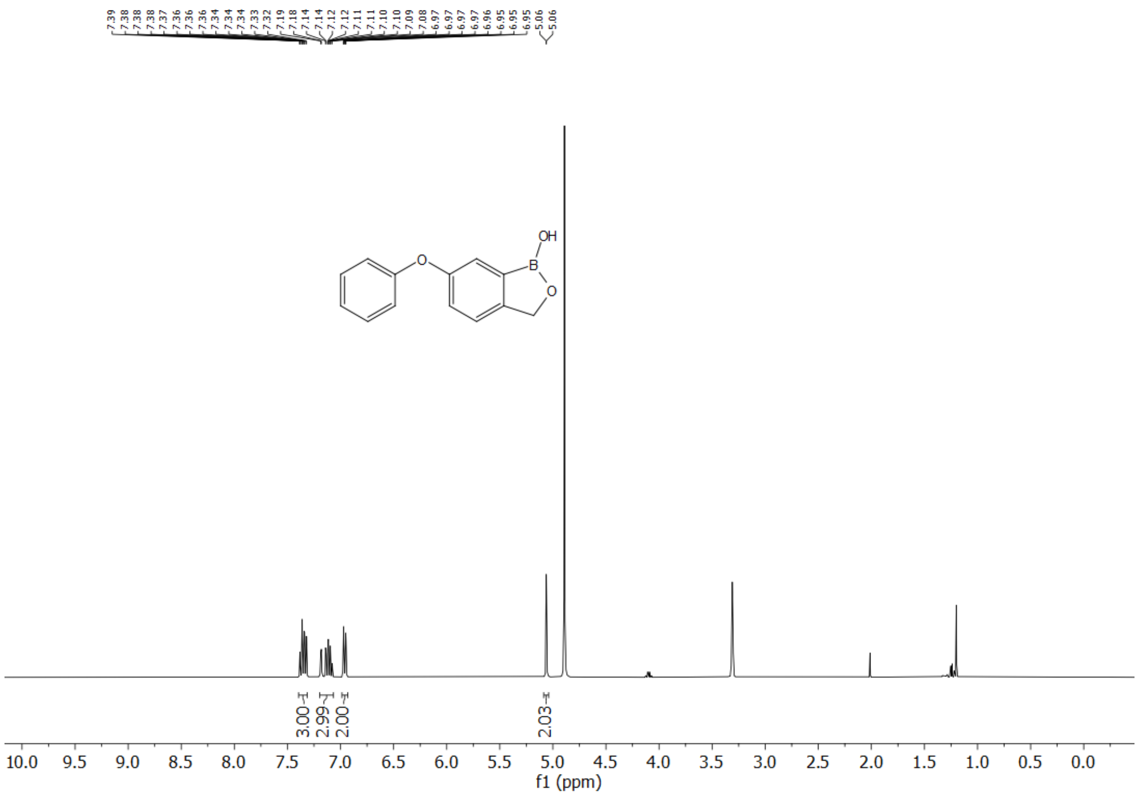

Figure S119: ^{1}H NMR (400 MHz, CD_3OD, 298 K) spectrum of 6-phenoxybenzo[*c*][1,2]oxaborol-1(3*H*)-ol (5y).

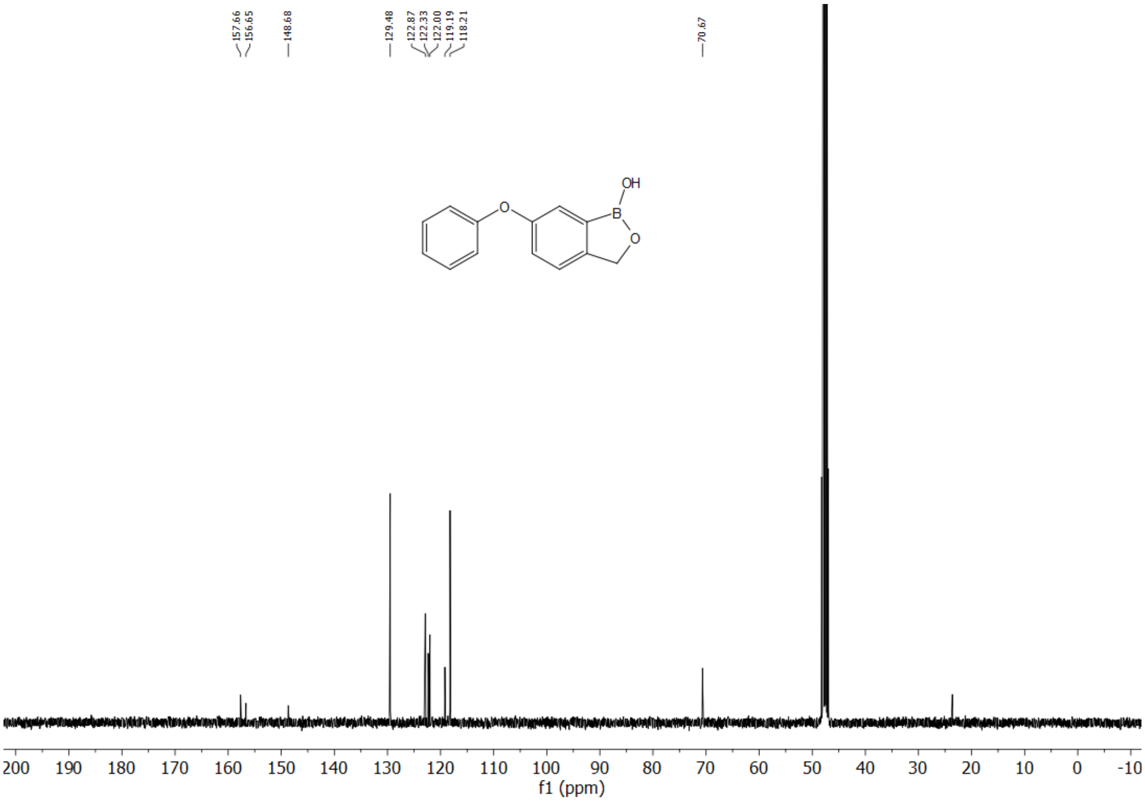

Figure S120: ^{13}C{^{1}H} NMR (101 MHz, CD_3OD, 298 K) spectrum of 6-phenoxybenzo[*c*][1,2]oxaborol-1(3*H*)-ol (5y).

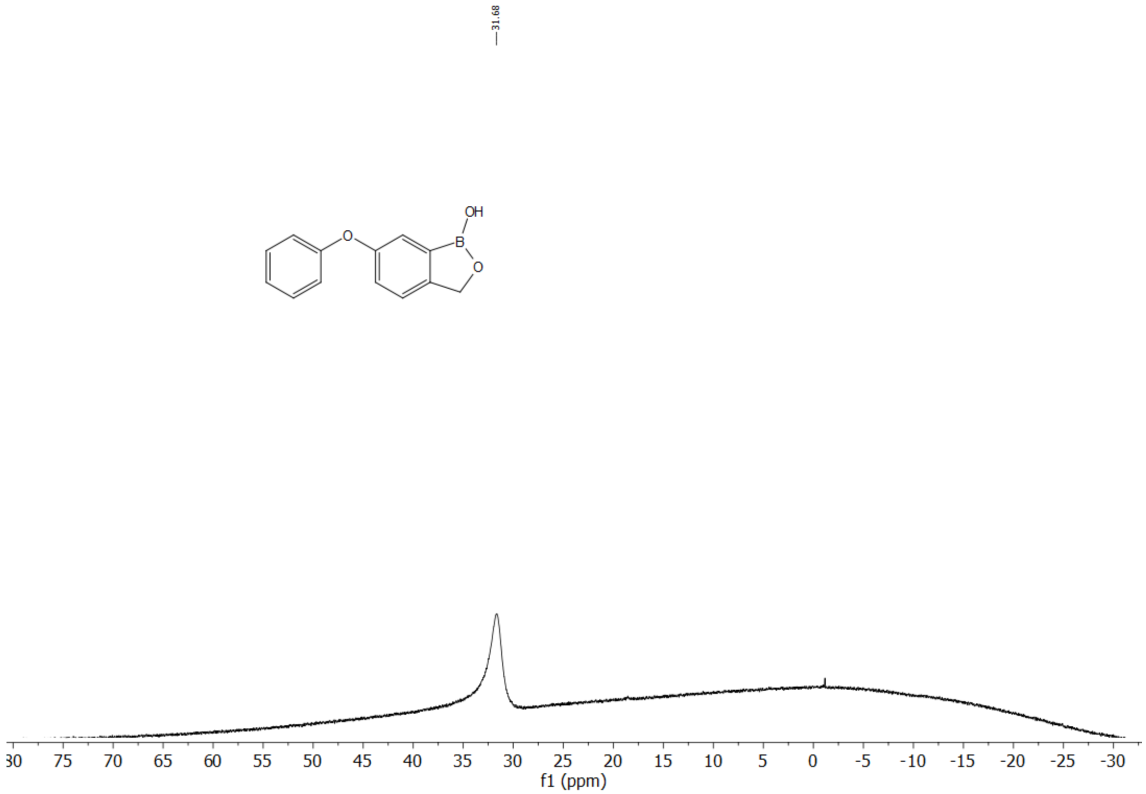

Figure S121: $^{11}B\{^{1}H\}$ NMR (128 MHz, CD_3OD, 298 K) spectrum of 6-phenoxybenzo[*c*][1,2]oxaborol-1(3*H*)-ol (5y).

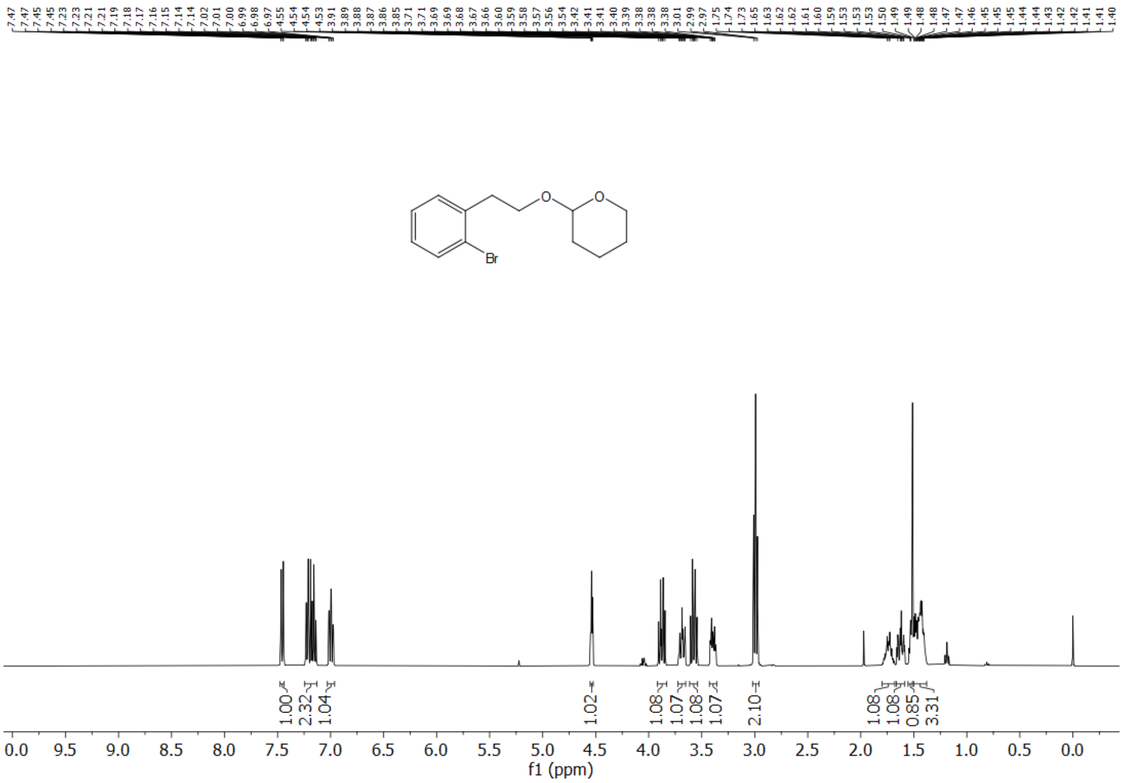

Figure S122: ^{1}H NMR (400 MHz, CD_2Cl_2, 298 K) spectrum of 2-(2-bromophenethoxy)tetrahydro-2*H*-pyran.

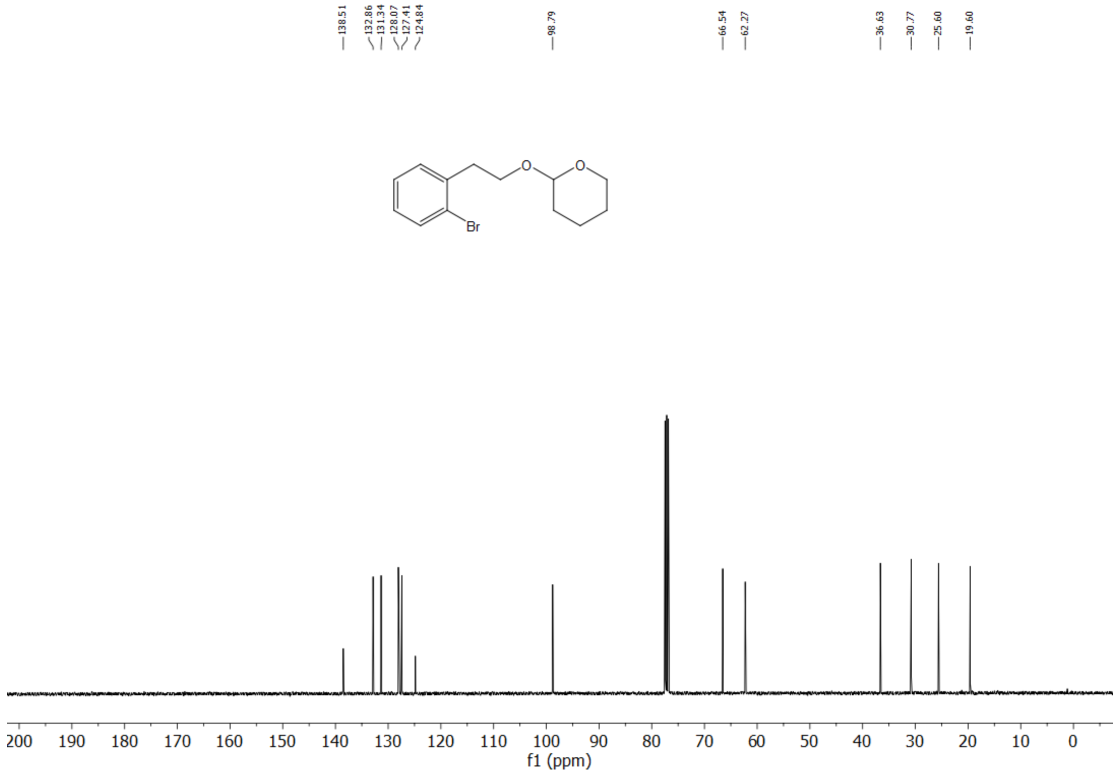

Figure S123: $^{13}C\{^{1}H\}$ NMR (101 MHz, $CDCl_3$, 298 K) spectrum of 2-(2-bromophenethoxy)tetrahydro-2*H*-pyran.

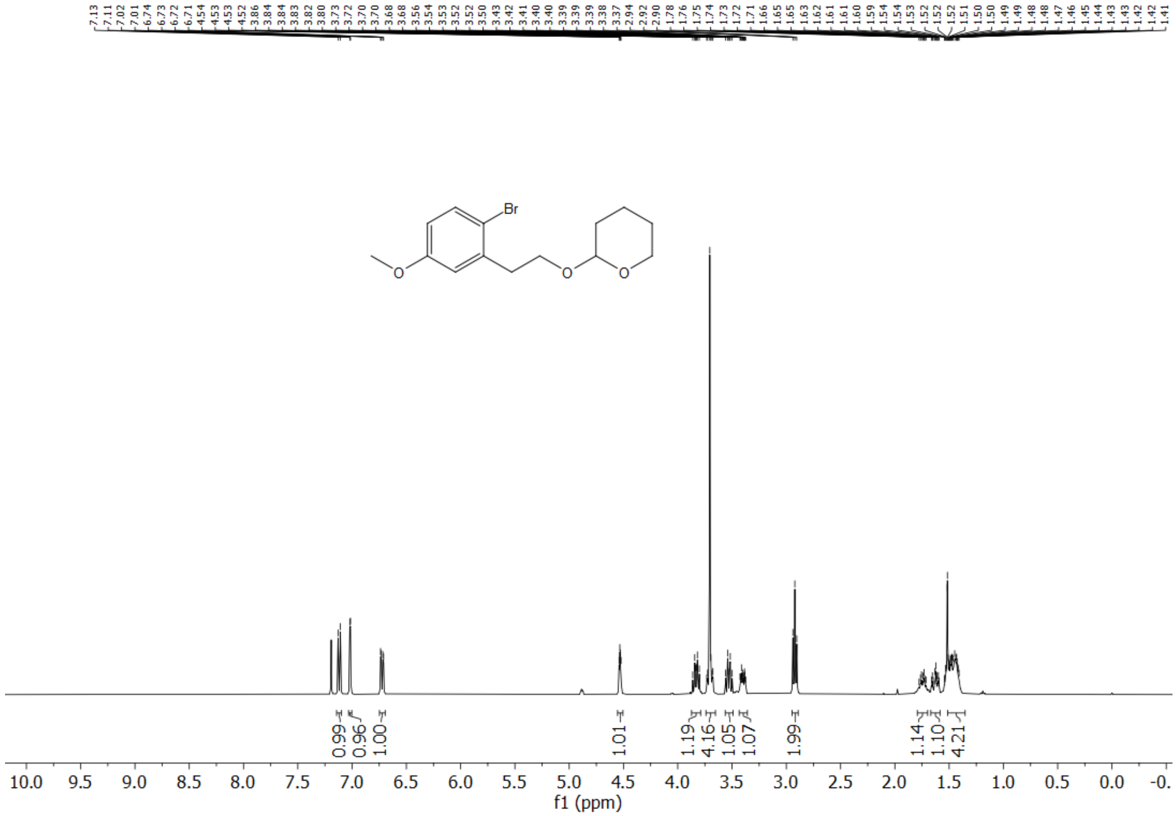

Figure S124: ^{1}H NMR (400 MHz, $CDCl_3$, 298 K) spectrum of 2-(2-bromo-5-methoxyphenethoxy)tetrahydro-2*H*-pyran

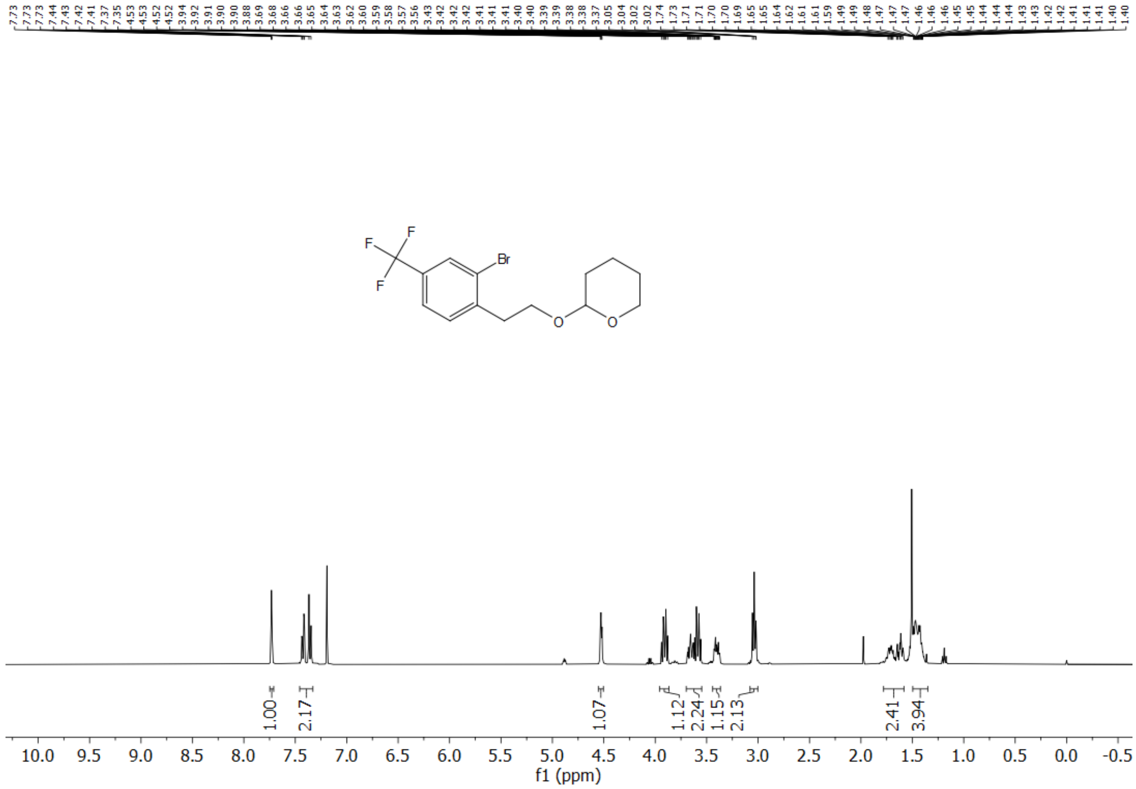

Figure S125: ^{1}H NMR (400 MHz, $CDCl_3$, 298 K) spectrum of 2-(2-bromo-4-(trifluoromethyl)phenethoxy)tetrahydro-2*H*-pyran.

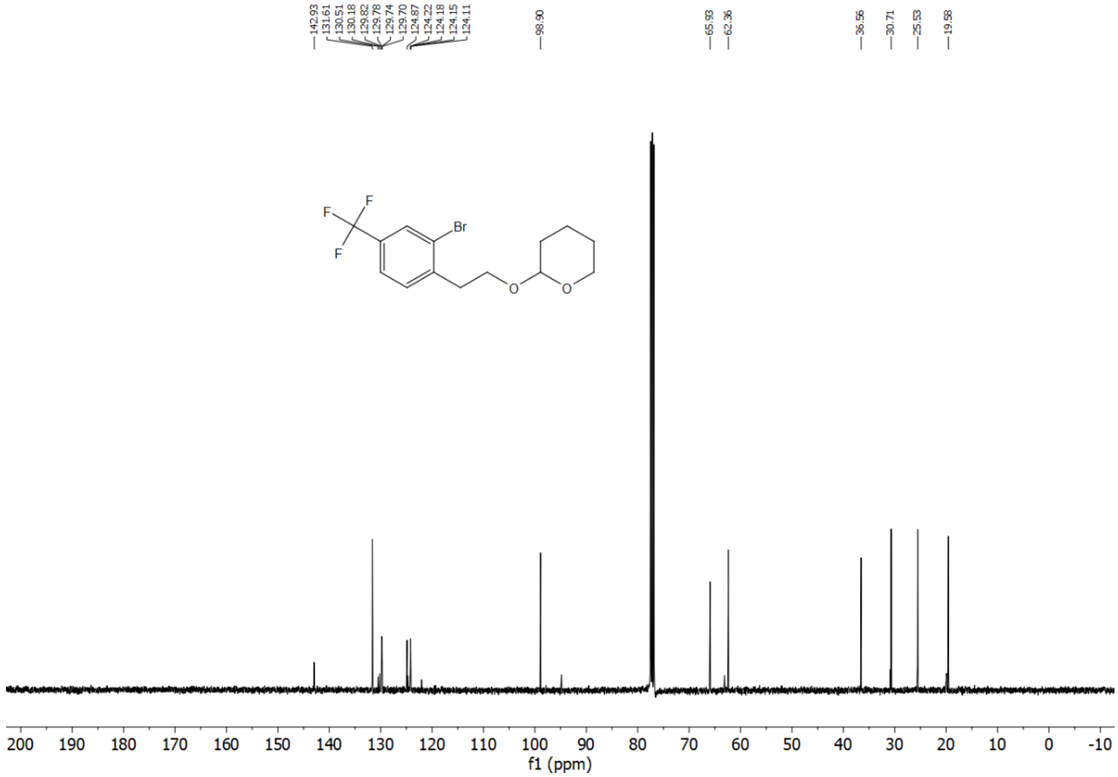

Figure S126: $^{13}C\{^{1}H\}$ NMR (101 MHz, $CDCl_3$, 298 K) spectrum of 2-(2-bromo-4-(trifluoromethyl)phenethoxy)tetrahydro-2*H*-pyran.

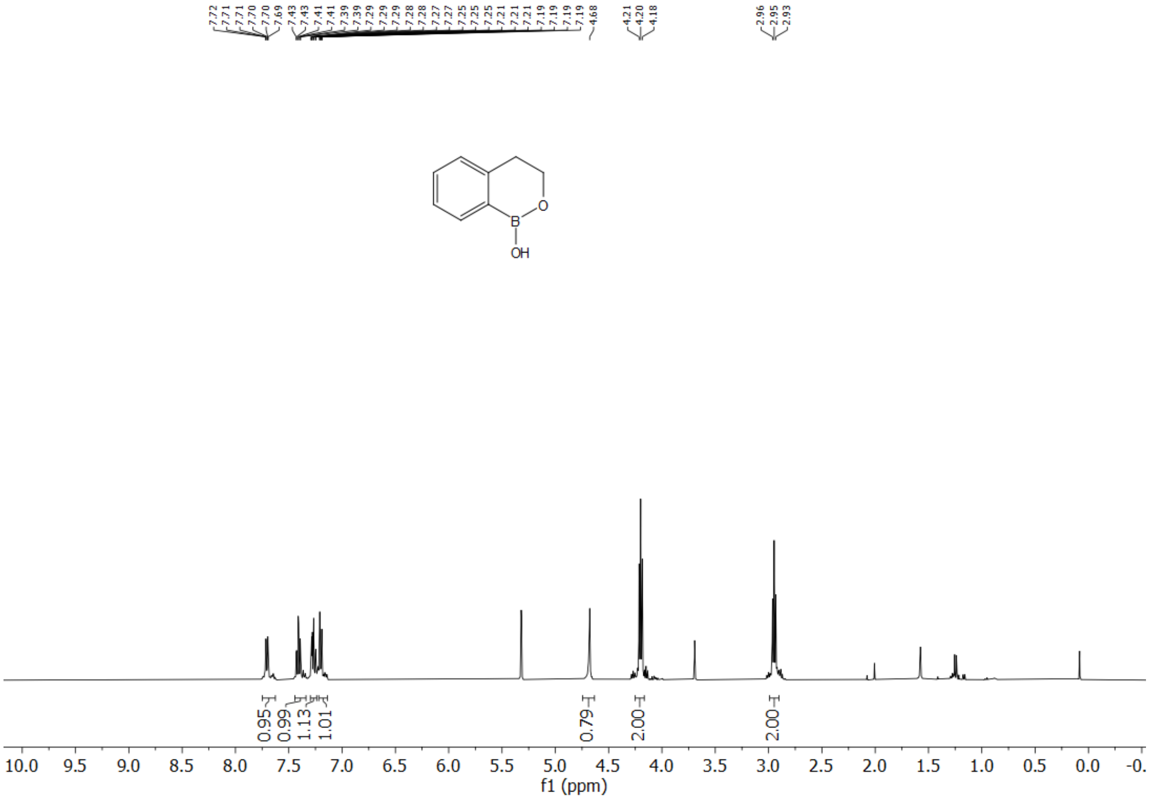

Figure S127: ^{1}H NMR (400 MHz, CD_2Cl_2, 298 K) spectrum of 3,4-dihydro-1*H*-benzo[*c*][1,2]oxaborinin-1-ol (7a).

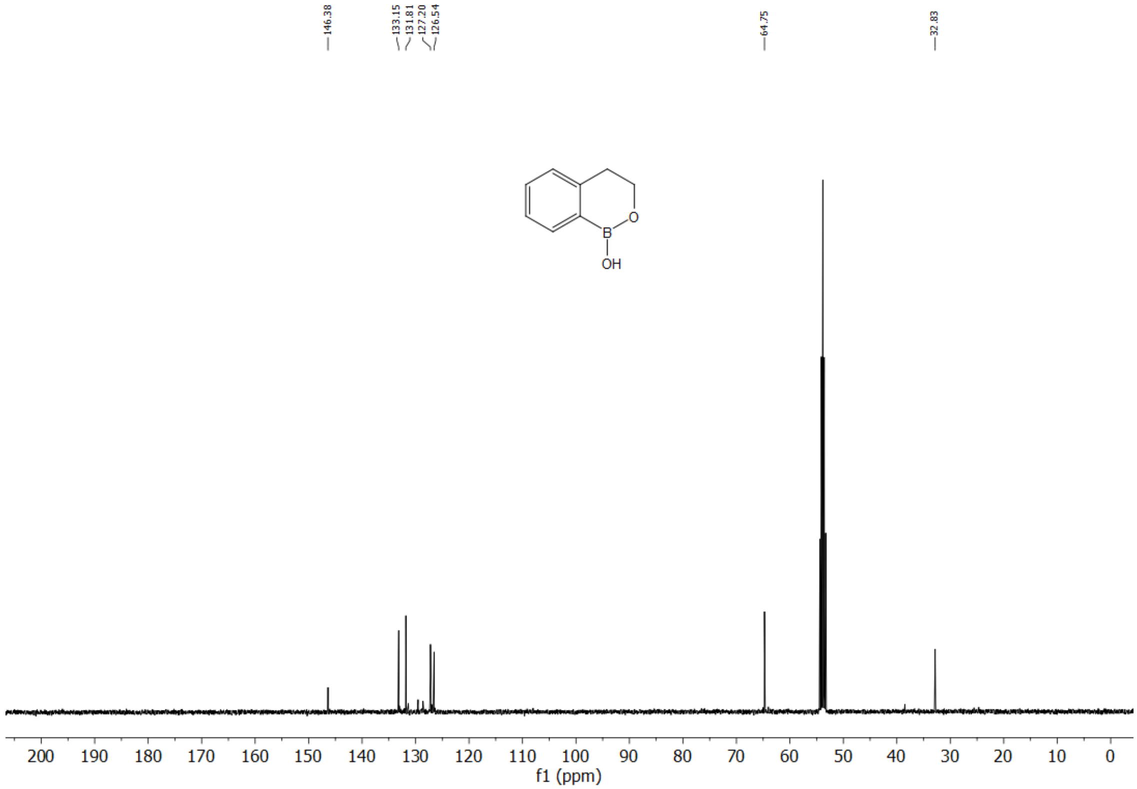

Figure S128: $^{13}C\{^{1}H\}$ NMR (101 MHz, CD_2Cl_2, 298 K) spectrum of 3,4-dihydro-1*H*-benzo[*c*][1,2]oxaborinin-1-ol (7a).

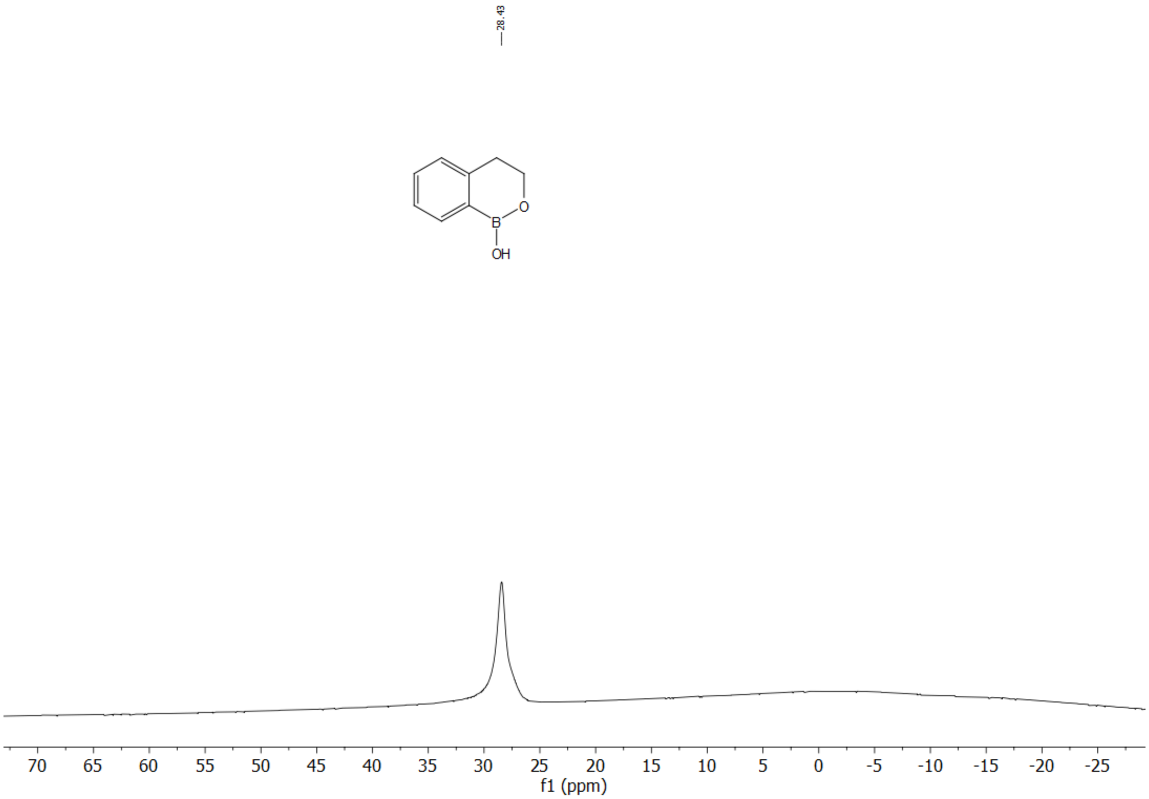

Figure S129: $^{11}B\{^{1}H\}$ NMR (128 MHz, CD_2Cl_2, 298 K) spectrum of 3,4-dihydro-1*H*-benzo[*c*][1,2]oxaborinin-1-ol (7a).

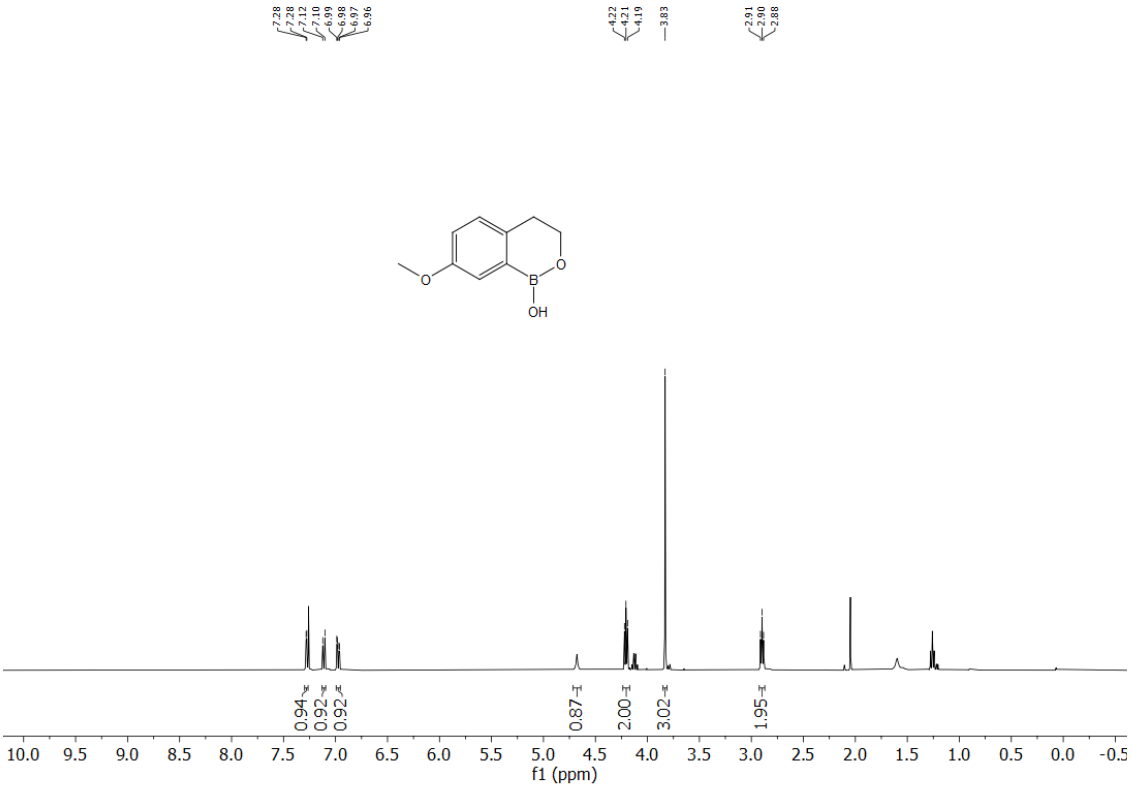

Figure S130: ^{1}H NMR (400 MHz, $CDCl_3$, 298 K) spectrum of 7-methoxy-3,4-dihydro-1*H*-benzo[*c*][1,2]oxaborinin-1-ol (7b).

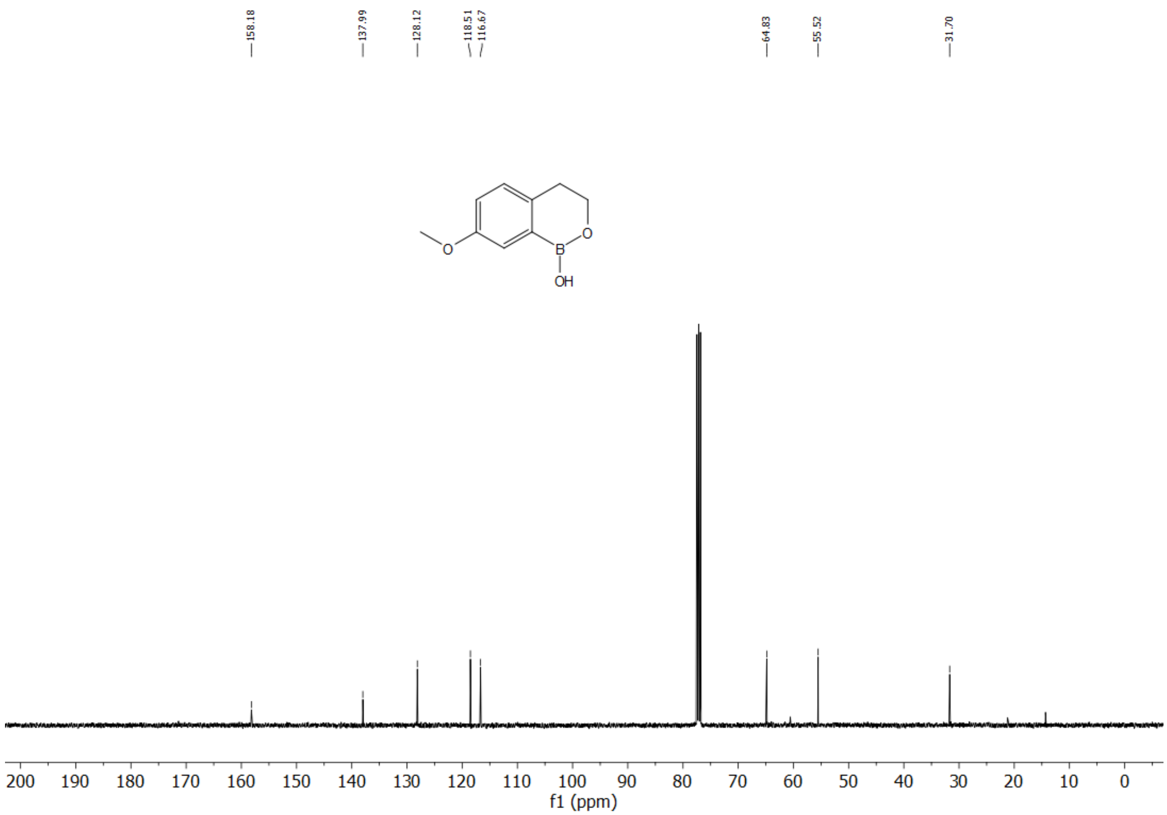

Figure S131: $^{13}C\{^{1}H\}$ NMR (101 MHz, $CDCl_3$, 298 K) spectrum of 7-methoxy-3,4-dihydro-1*H*-benzo[*c*][1,2]oxaborinin-1-ol (7b).

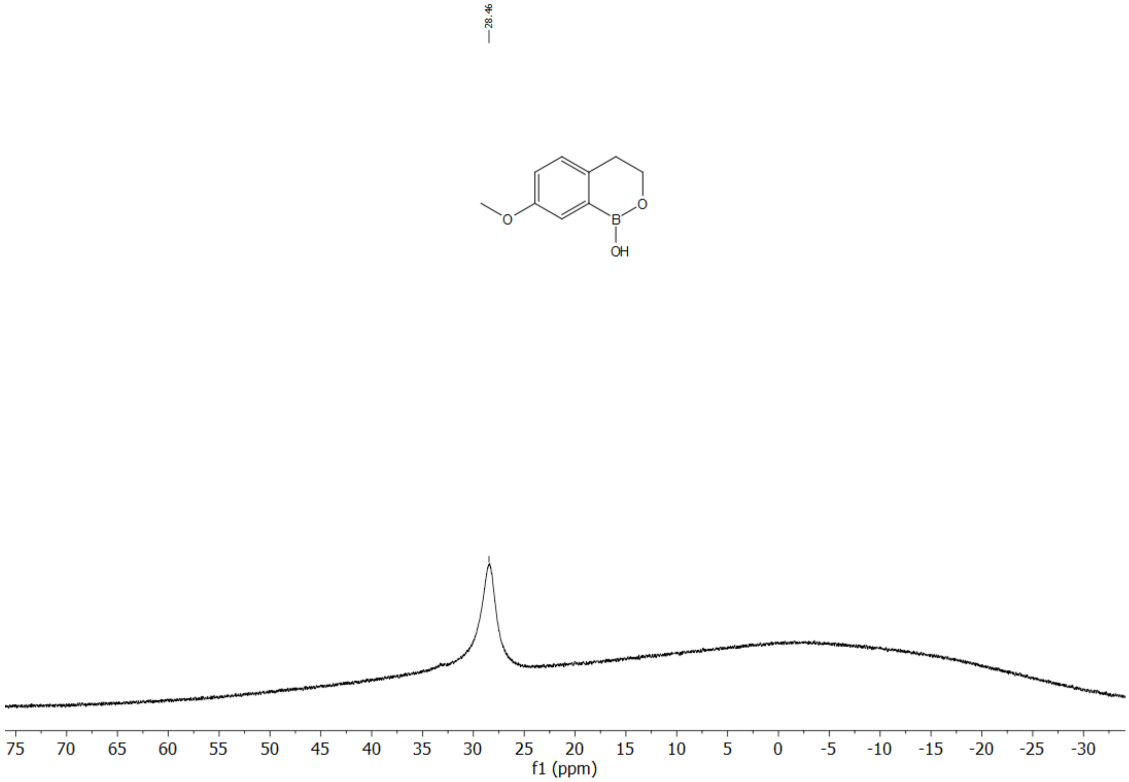

Figure S132: $^{11}B\{^{1}H\}$ NMR (128 MHz, $CDCl_3$, 298 K) spectrum of 7-methoxy-3,4-dihydro-1*H*-benzo[*c*][1,2]oxaborinin-1-ol (7b).

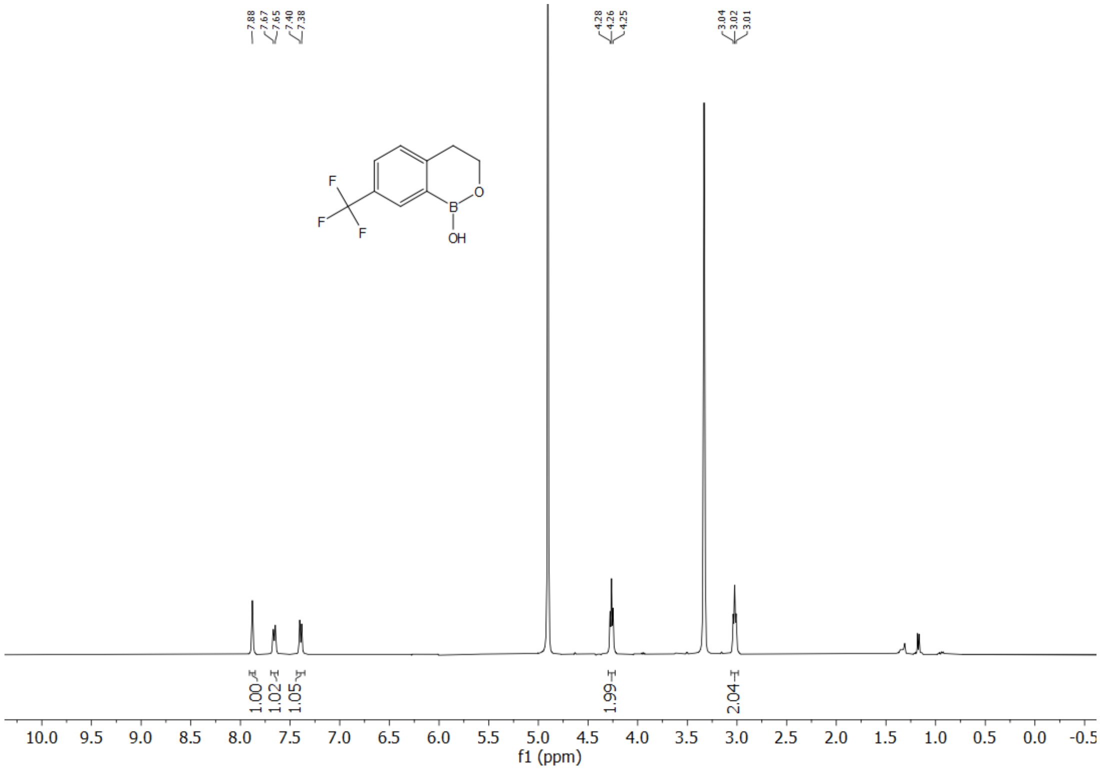

Figure S133: ^{1}H NMR (400 MHz, CD_3OD, 298 K) spectrum of 7-(trifluoromethyl)-3,4-dihydro-1*H*-benzo[*c*][1,2]oxaborinin-1-ol (7c).

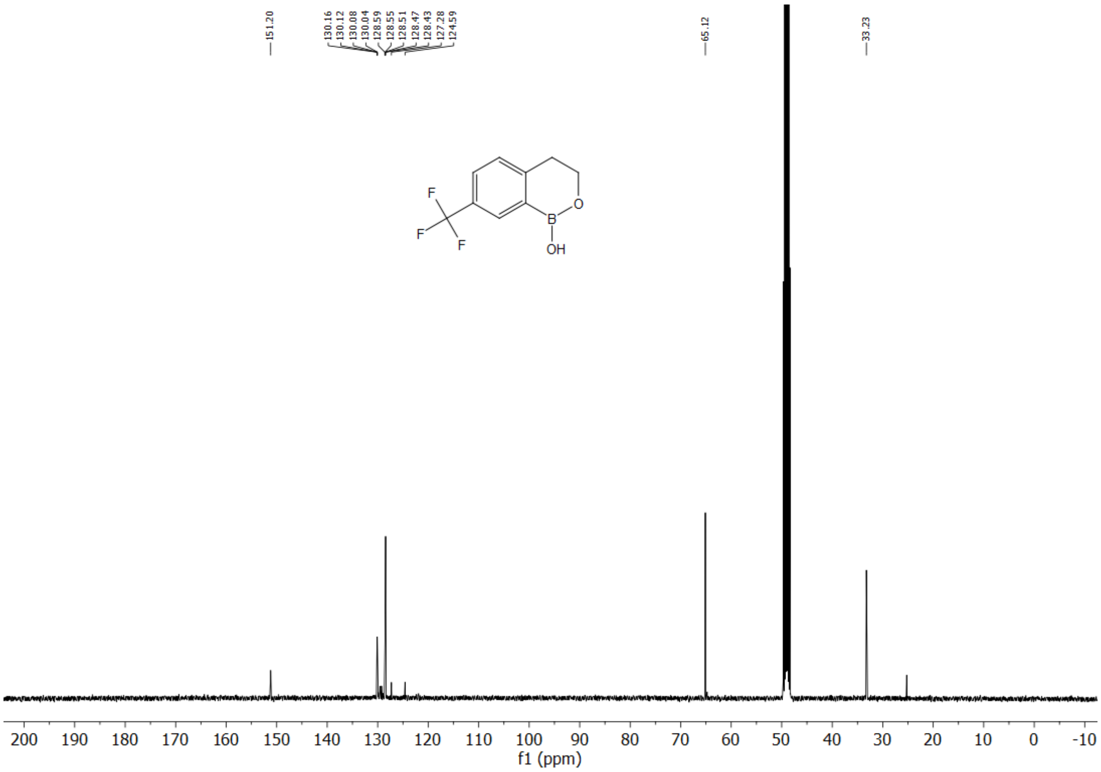

Figure S134: $^{13}C\{^{1}H\}$ NMR (101 MHz, CD_3OD, 298 K) spectrum of 7-(trifluoromethyl)-3,4-dihydro-1*H*-benzo[*c*][1,2]oxaborinin-1-ol (7c).

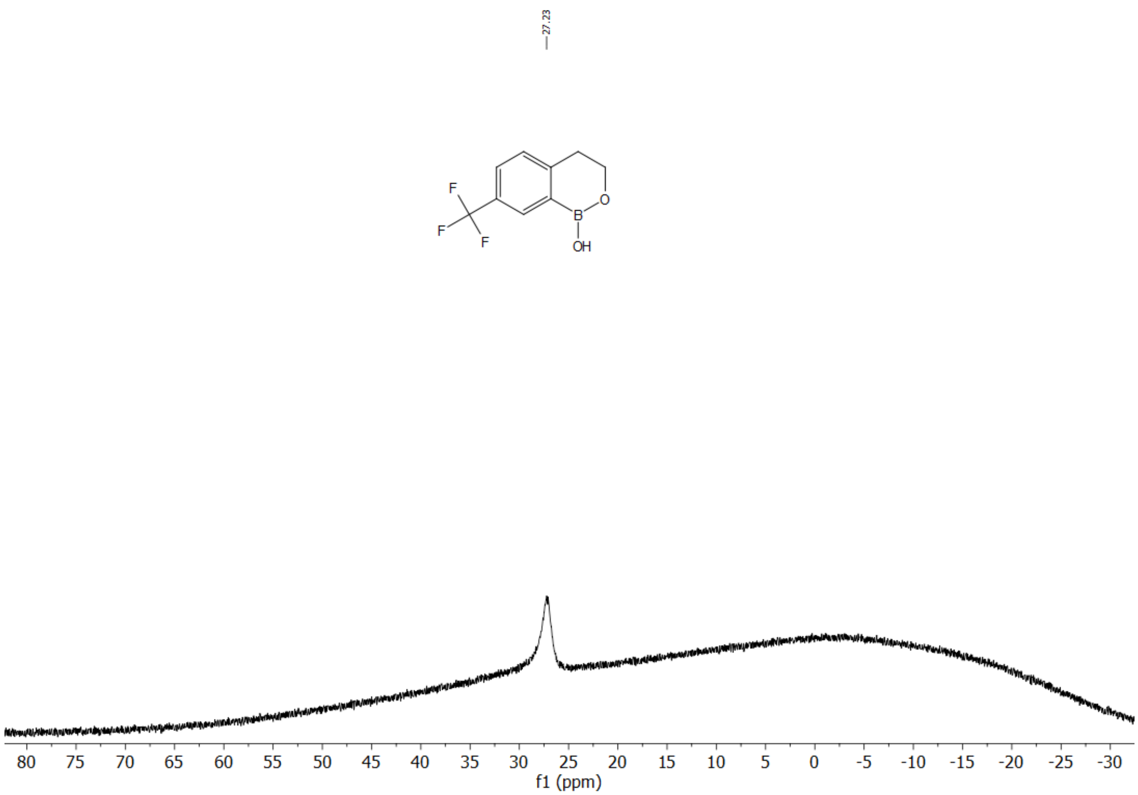

Figure S135: $^{11}B\{^1H\}$ NMR (128 MHz, CD_3OD, 298 K) spectrum of 7-(trifluoromethyl)-3,4-dihydro-1*H*-benzo[*c*][1,2]oxaborinin-1-ol (7c).

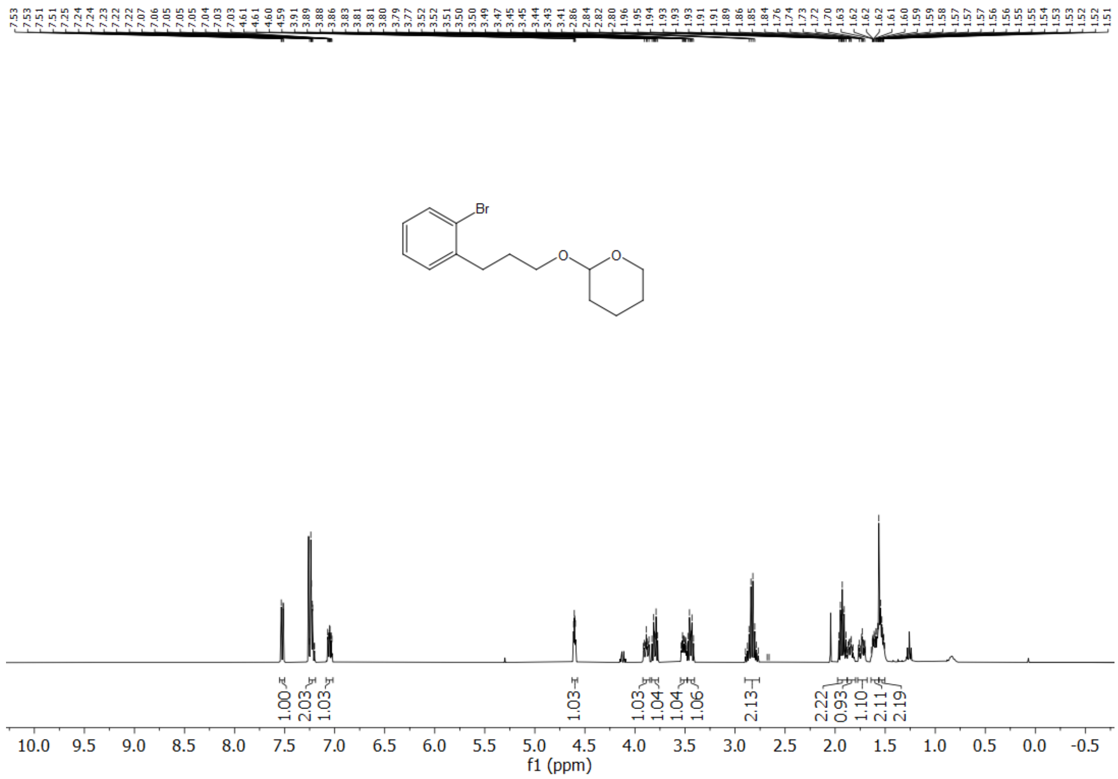

Figure S136: ^1H NMR (400 MHz, $CDCl_3$, 298 K) spectrum of 2-(3-(2-bromophenyl)propoxy)tetrahydro-2*H*-pyran.

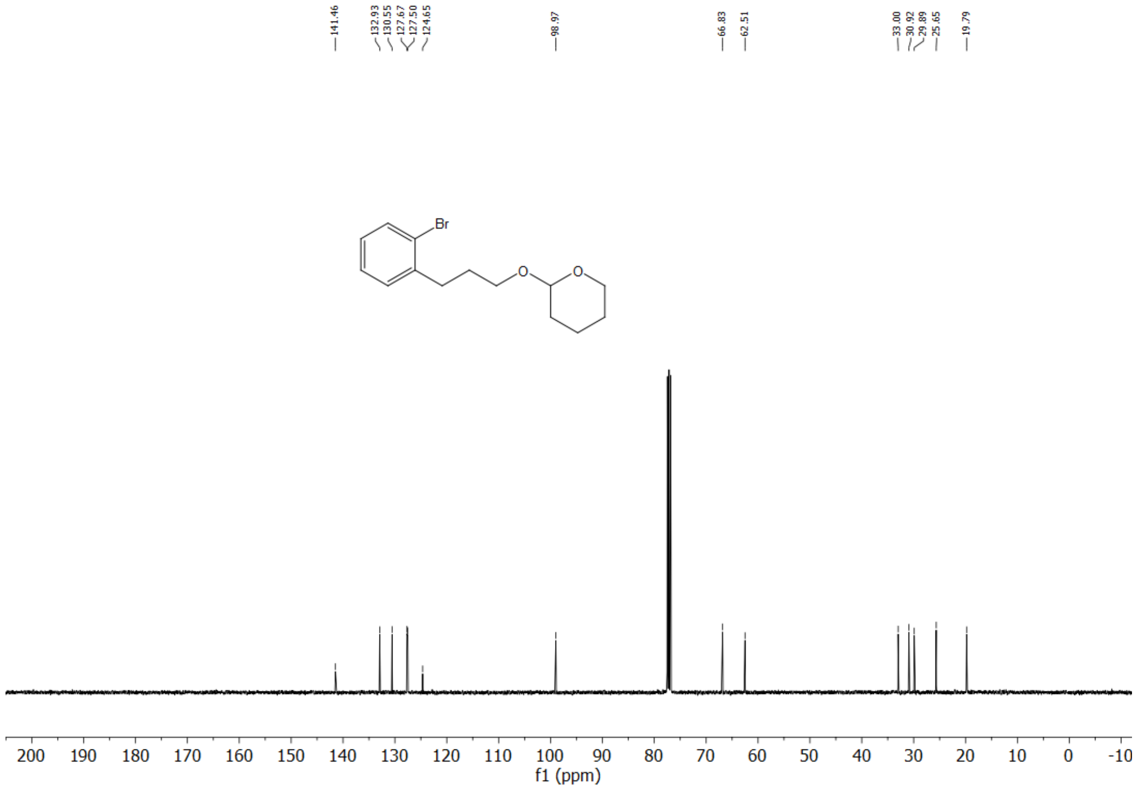

Figure S137: $^{13}C\{^{1}H\}$ NMR (101 MHz, $CDCl_3$, 298 K) spectrum of 2-(3-(2-bromophenyl)propoxy)tetrahydro-2*H*-pyran.

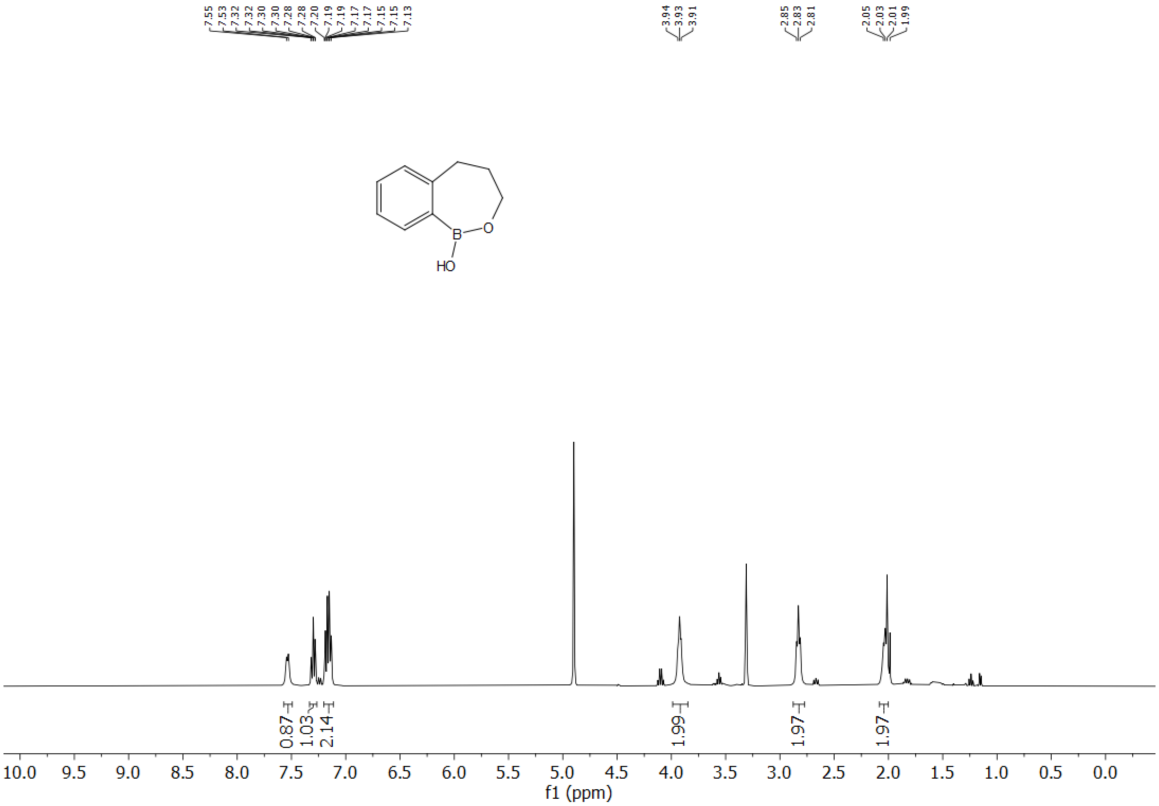

Figure S138: ^{1}H NMR (400 MHz, CD_3OD, 298 K) spectrum of 4,5-dihydrobenzo[*c*][1,2]oxaborepin-1(3*H*)-ol (8d).

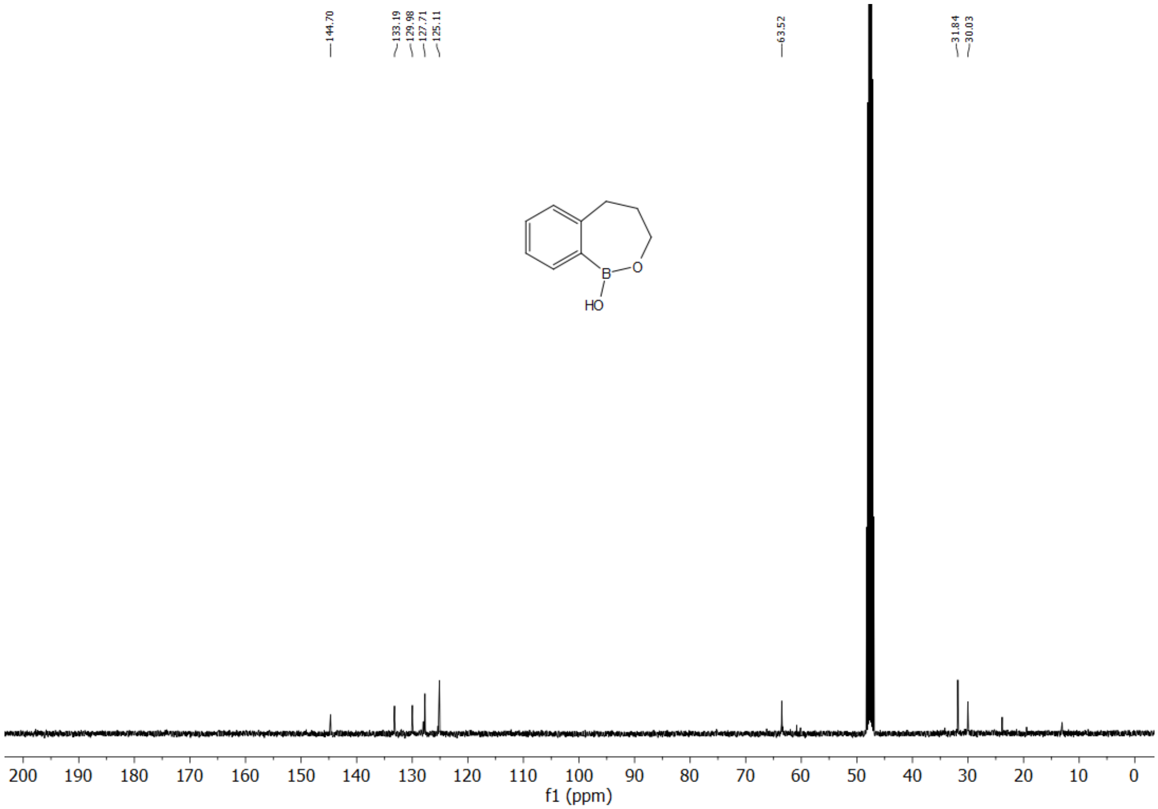

Figure S139: $^{13}C\{^{1}H\}$ NMR (101 MHz, CD_3OD, 298 K) spectrum of 4,5-dihydrobenzo[*c*][1,2]oxaborepin-1(3*H*)-ol (8d).

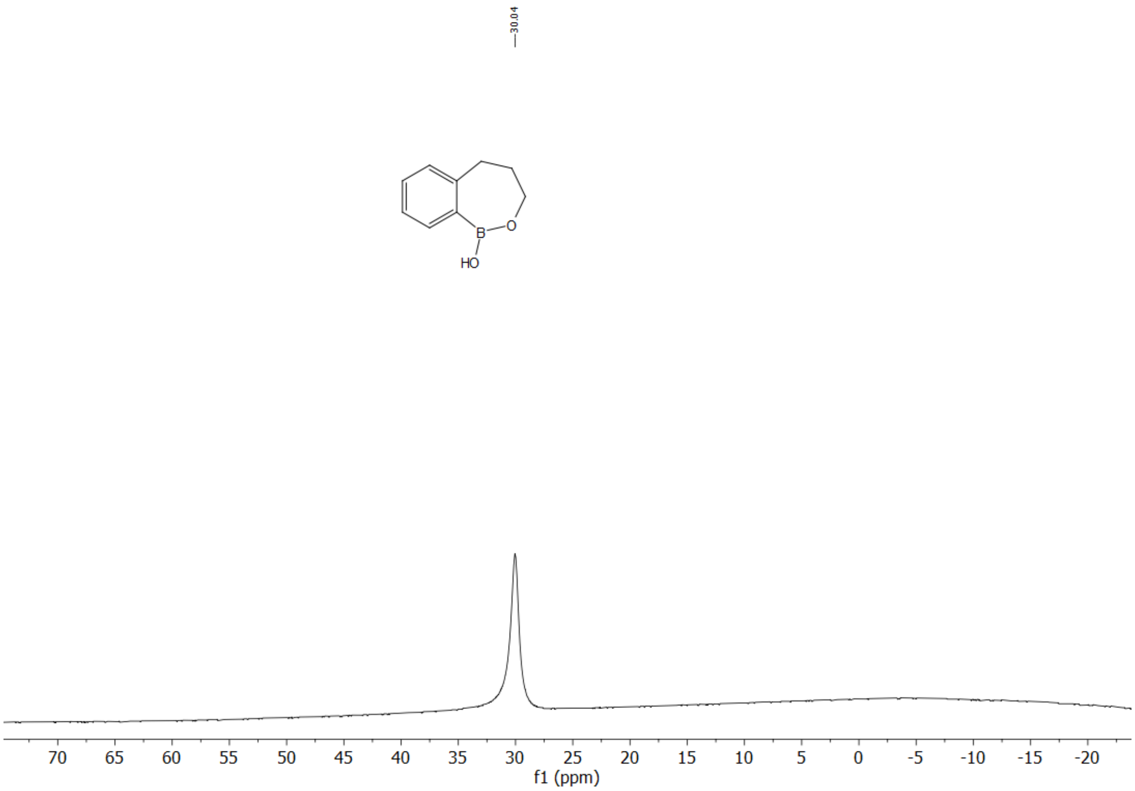

Figure S140: $^{11}B\{^{1}H\}$ NMR (128 MHz, CD_3OD, 298 K) spectrum of 4,5-dihydrobenzo[*c*][1,2]oxaborepin-1(3*H*)-ol (8d).

30.Spectral data of the products

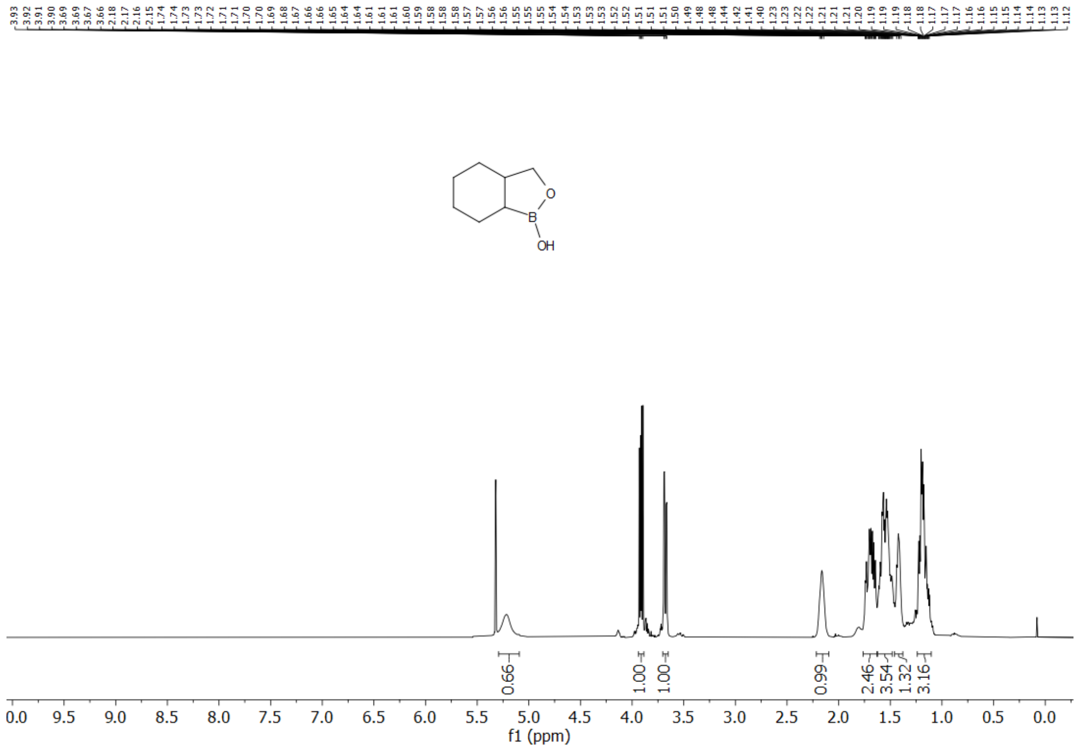

Figure S141: ^{1}H NMR (400 MHz, CD_2Cl_2, 298 K) spectrum of Hexahydrobenzo[*c*][1,2]oxaborol-1(3*H*)-ol (6a).

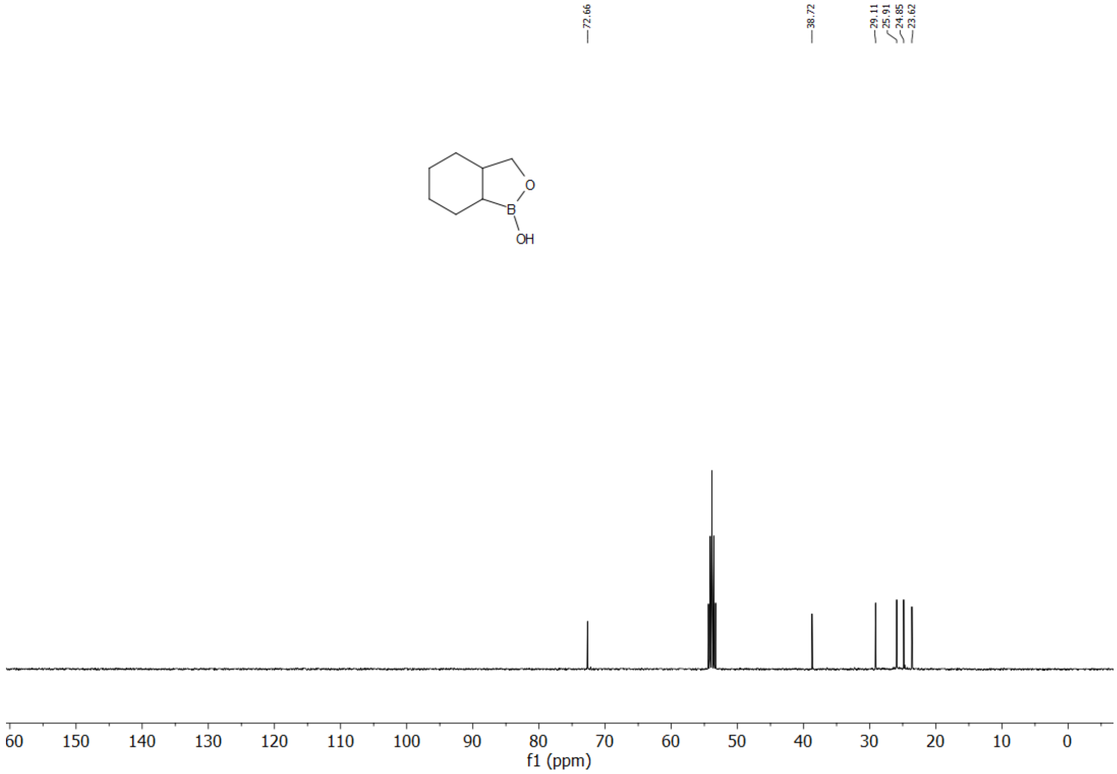

Figure S142: ^{13}C{^{1}H} NMR (101 MHz, CD_2Cl_2, 298 K) spectrum of Hexahydrobenzo[*c*][1,2]oxaborol-1(3*H*)-ol (6a).

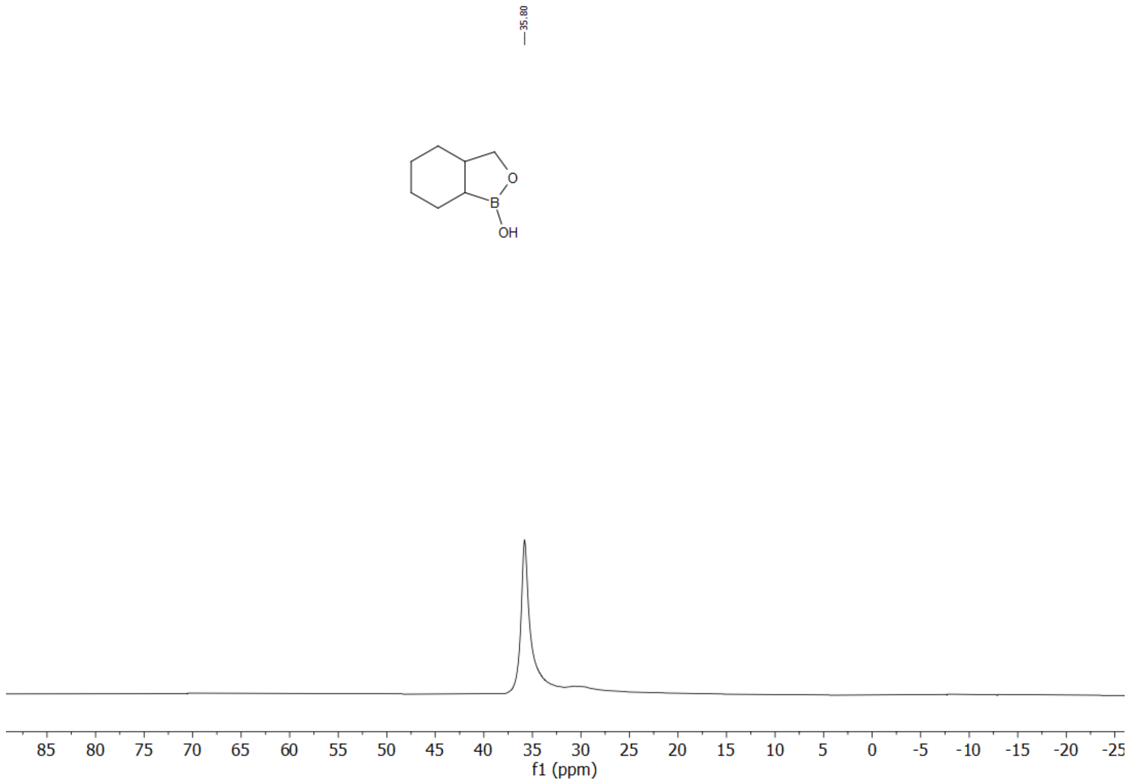

Figure S143: $^{11}B\{^{1}H\}$ NMR (128 MHz, CD_2Cl_2, 298 K) spectrum of Hexahydrobenzo[*c*][1,2]oxaborol-1(3*H*)-ol (6a).

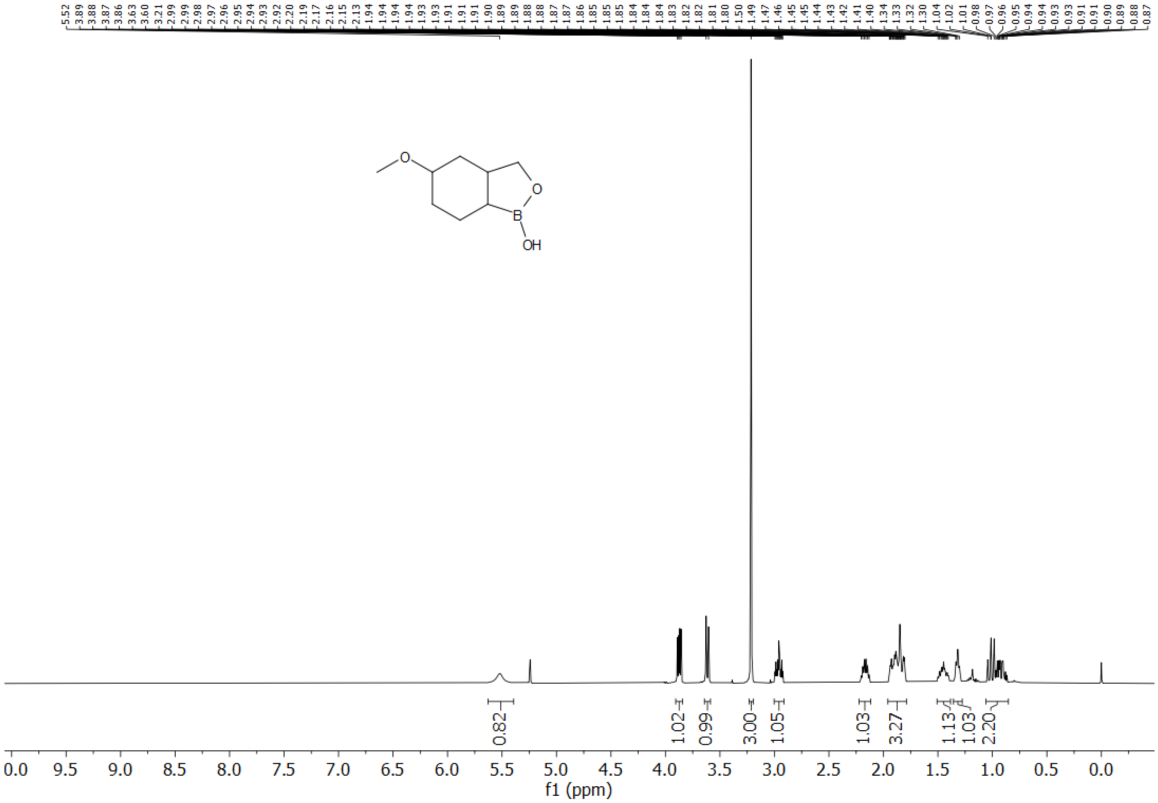

Figure S144: ^{1}H NMR (400 MHz, CD_2Cl_2, 298 K) spectrum of 5-methoxyhexahydrobenzo[*c*][1,2]oxaborol-1(3*H*)-ol (6b).

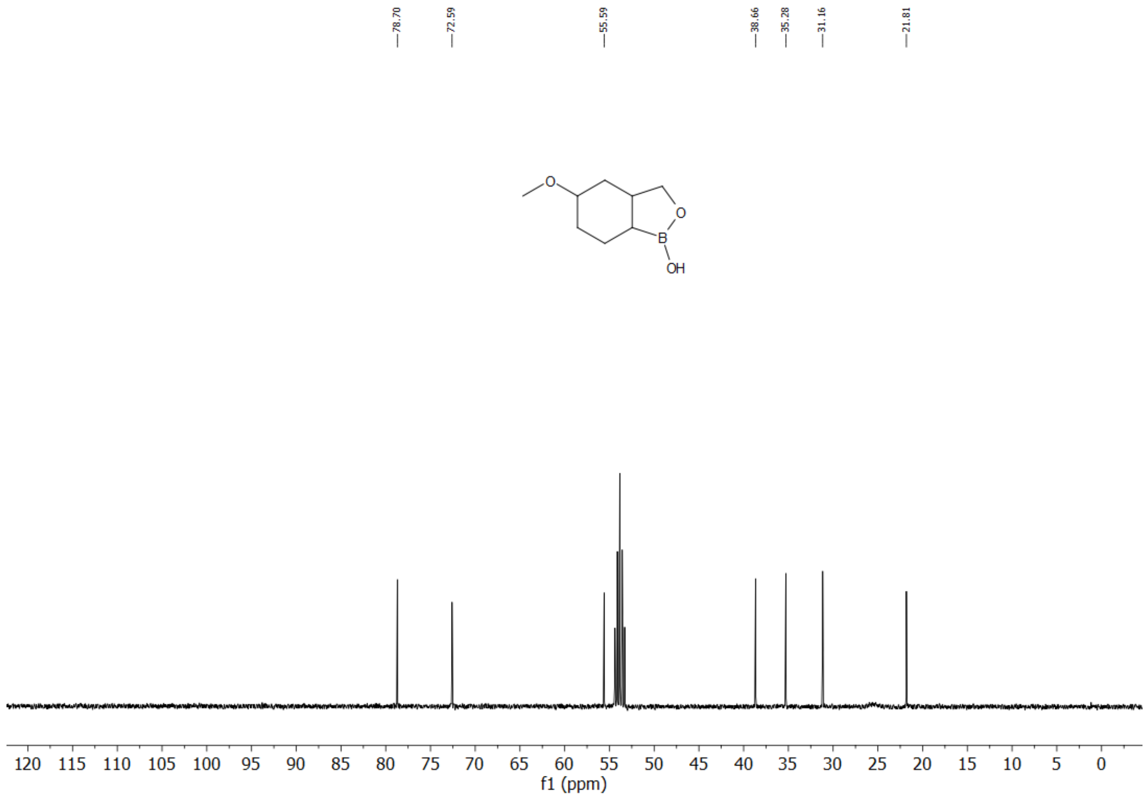

Figure S145: $^{13}C\{^{1}H\}$ NMR (101 MHz, CD_2Cl_2, 298 K) spectrum of 5-methoxyhexahydrobenzo[*c*][1,2]oxaborol-1(3*H*)-ol (6b).

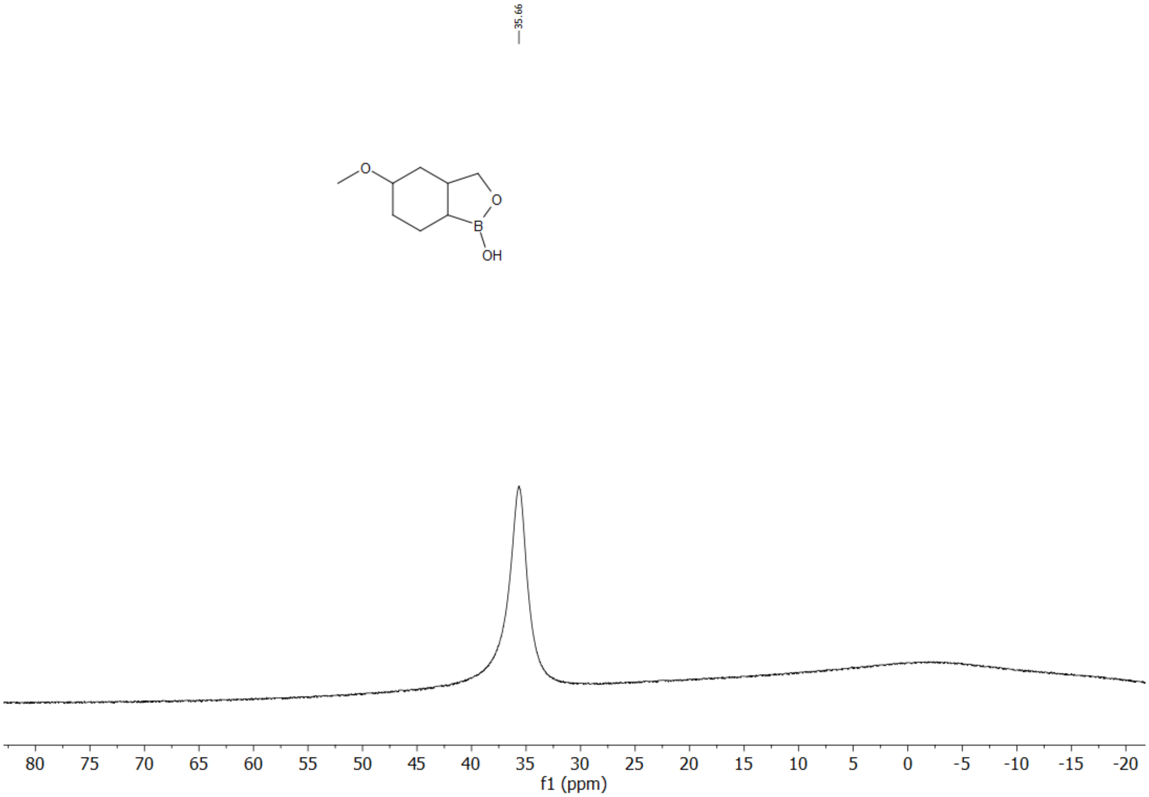

Figure S146: $^{11}B\{^{1}H\}$ NMR (128 MHz, CD_2Cl_2, 298 K) spectrum of 5-methoxyhexahydrobenzo[*c*][1,2]oxaborol-1(3*H*)-ol (6b).

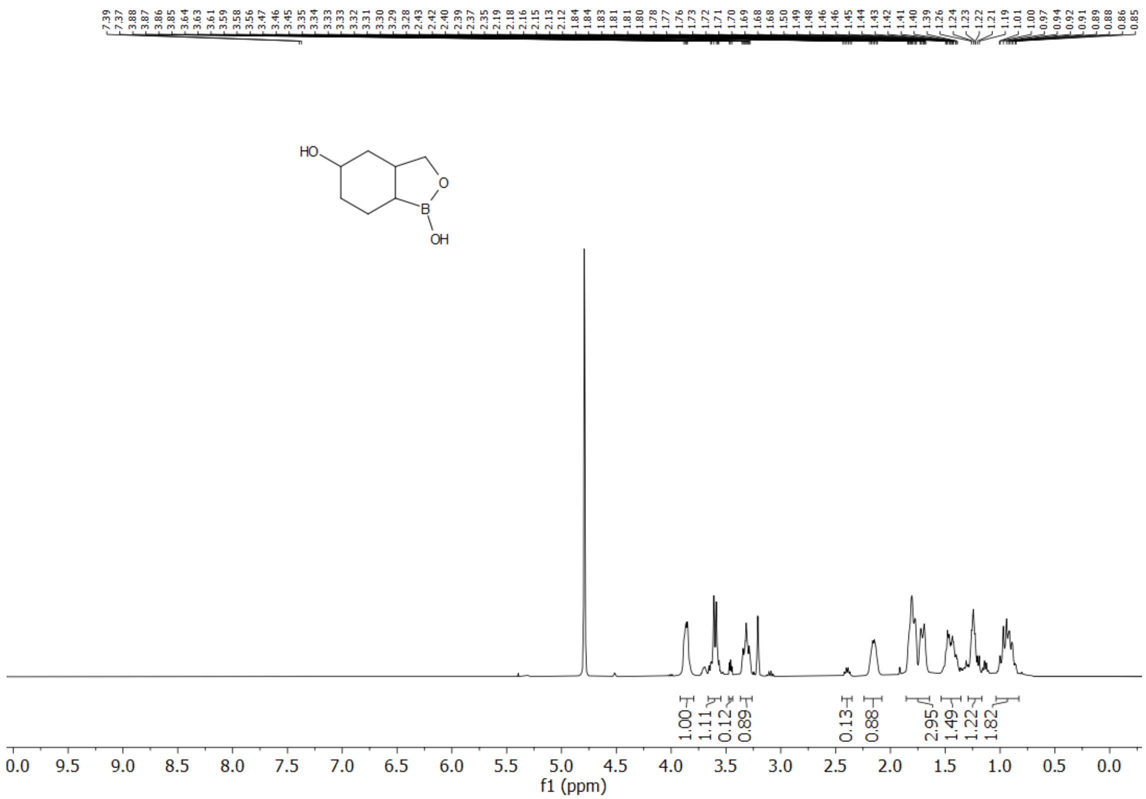

Figure S147: ^{1}H **NMR (400 MHz,** CD_3OD**, 298 K) spectrum of Hexahydrobenzo[*c*][1,2]oxaborole-1,5(3*H*)-diol (6c).**

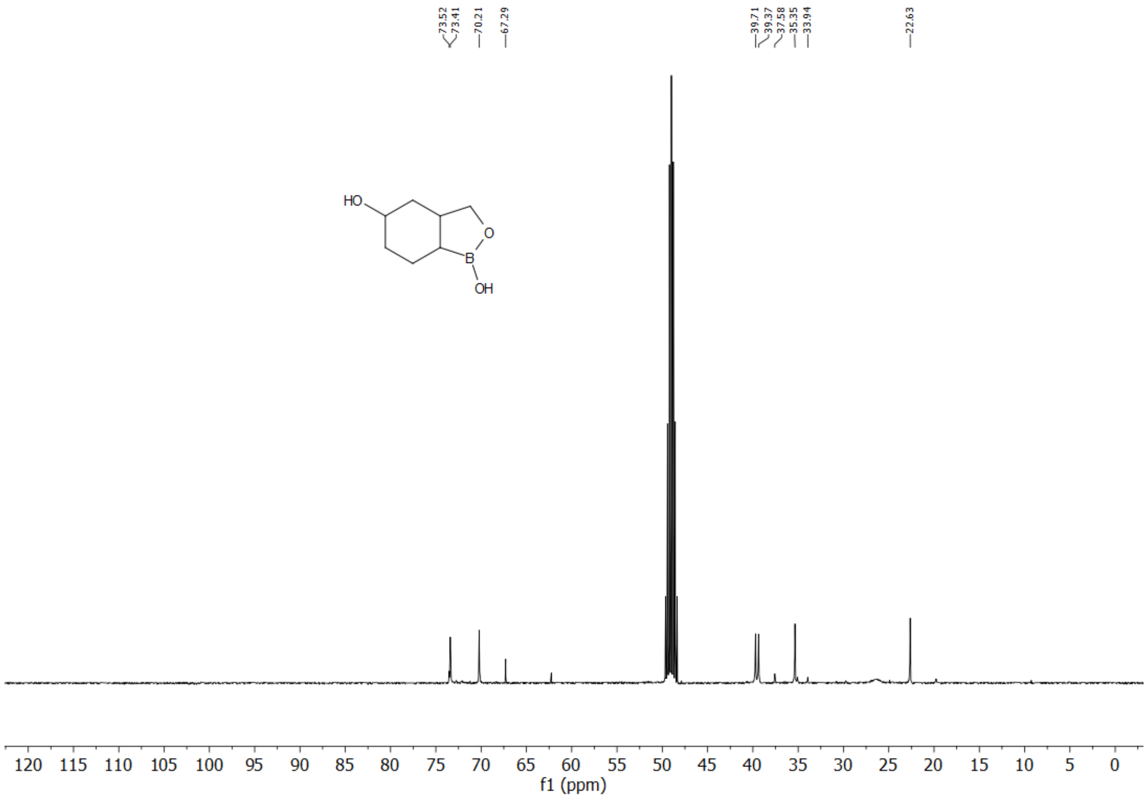

Figure S148: $^{13}C\{^{1}H\}$ **NMR (101 MHz,** CD_3OD**, 298 K) spectrum of Hexahydrobenzo[*c*][1,2]oxaborole-1,5(3*H*)-diol (6c).**

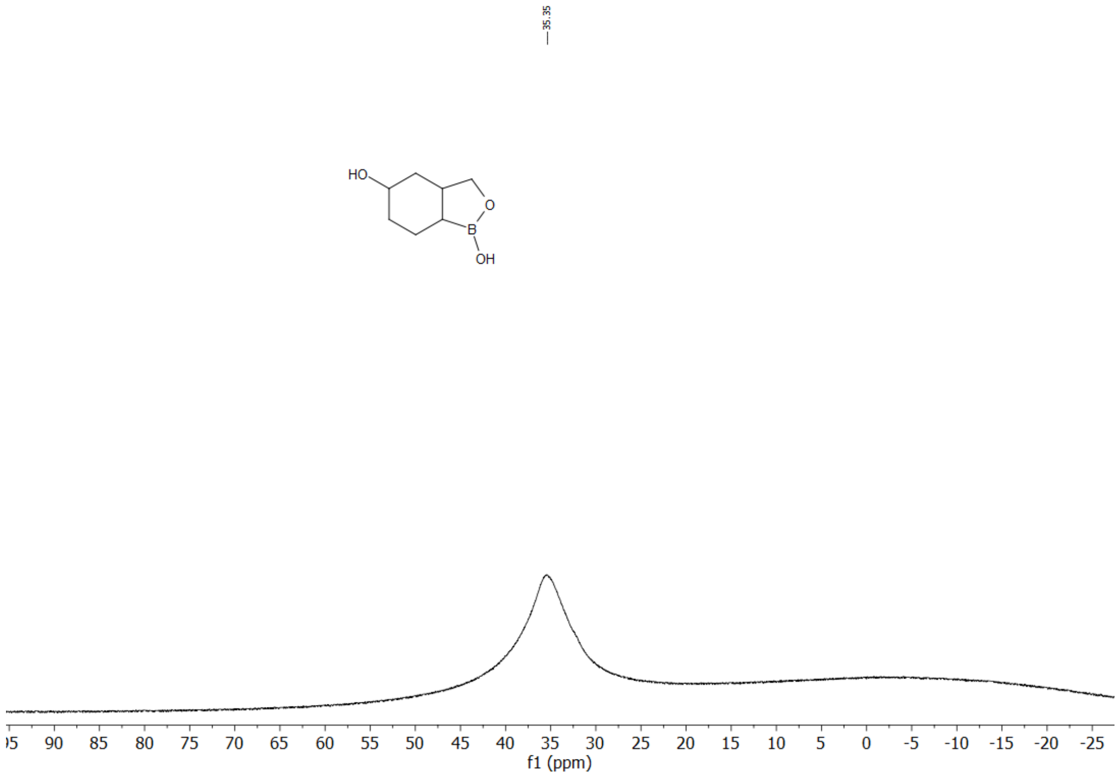

Figure S149: $^{11}B\{^1H\}$ NMR (128 MHz, CD_2Cl_2, 298 K) spectrum of Hexahydrobenzo[*c*][1,2]oxaborole-1,5(3*H*)-diol (6c).

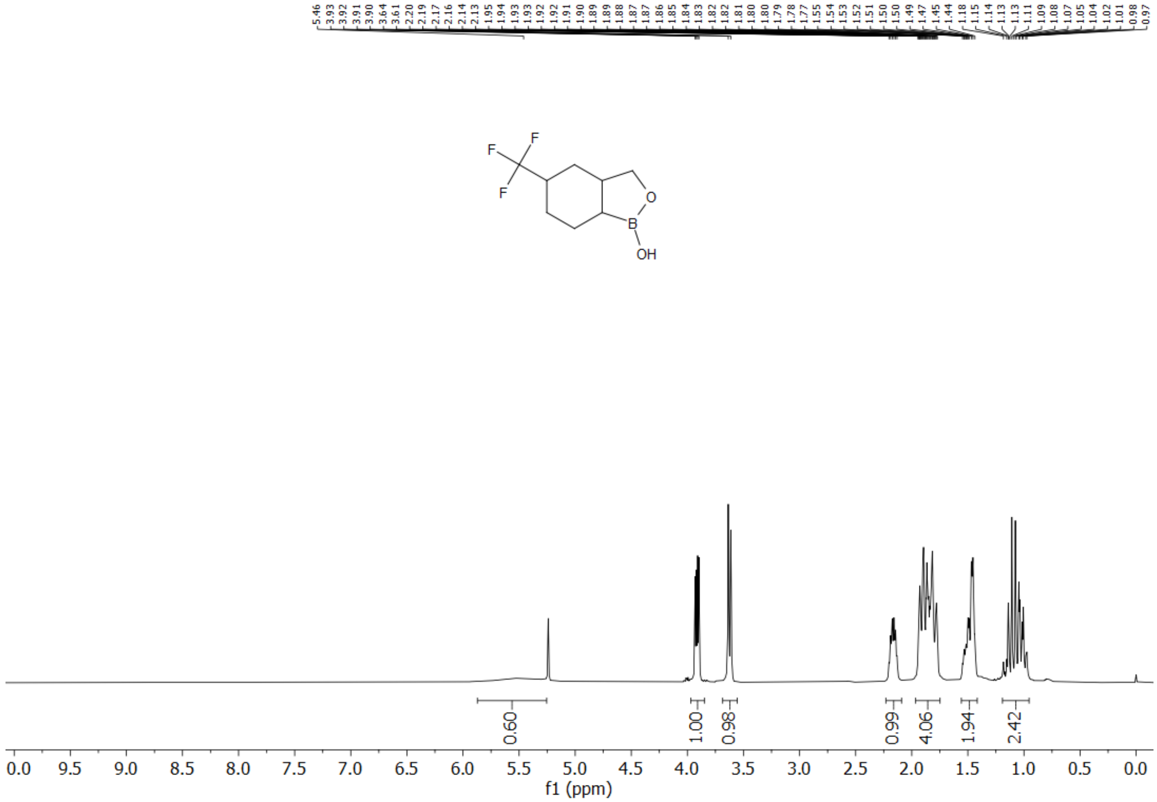

Figure S150: ^1H NMR (400 MHz, CD_2Cl_2, 298 K) spectrum of 5-(trifluoromethyl)hexahydrobenzo[*c*][1,2]oxaborol-1(3*H*)-ol (6d).

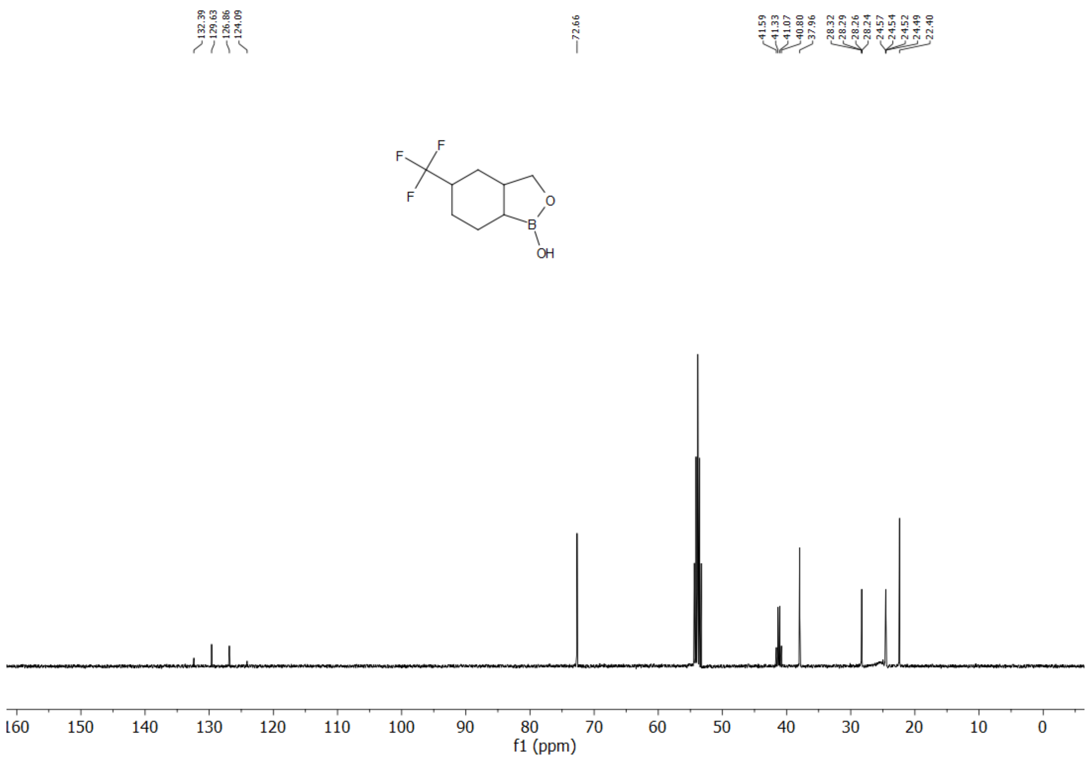

Figure S151: $^{13}C\{^{1}H\}$ NMR (101 MHz, CD_2Cl_2, 298 K) spectrum of 5-(trifluoromethyl)hexahydrobenzo[*c*][1,2]oxaborol-1(3*H*)-ol (6d).

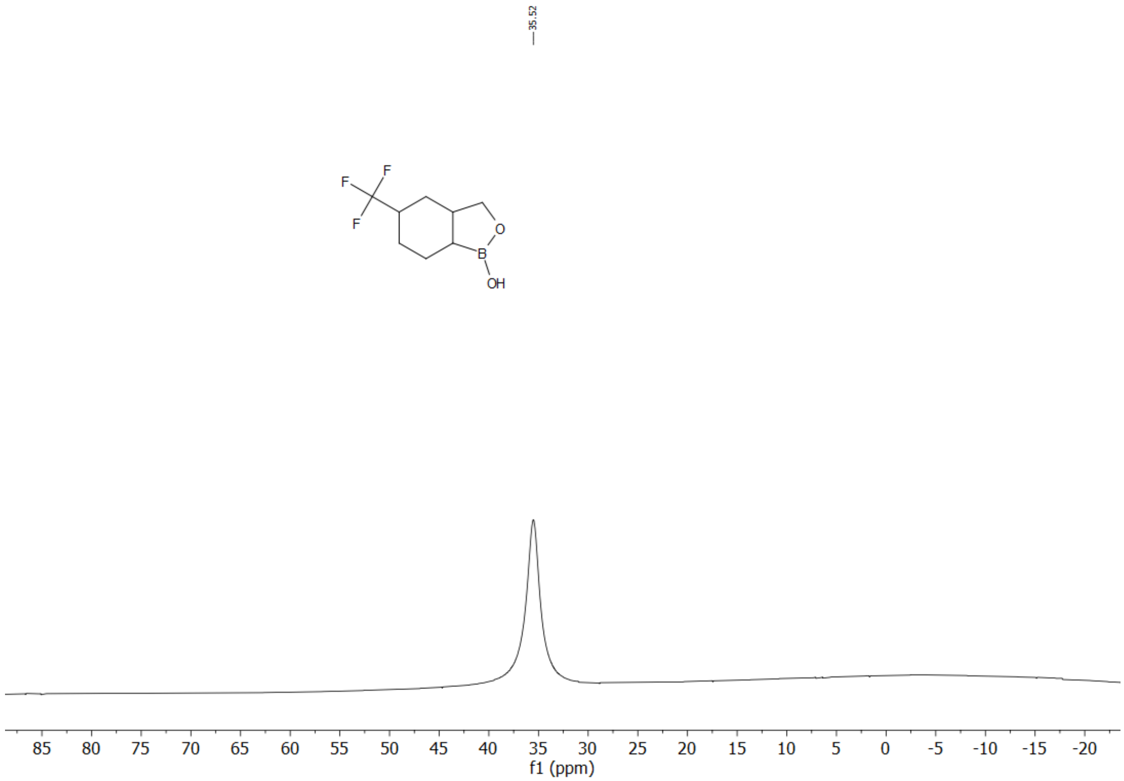

Figure S152: $^{11}B\{^{1}H\}$ NMR (128 MHz, CD_2Cl_2, 298 K) spectrum of 5-(trifluoromethyl)hexahydrobenzo[*c*][1,2]oxaborol-1(3*H*)-ol (6d).

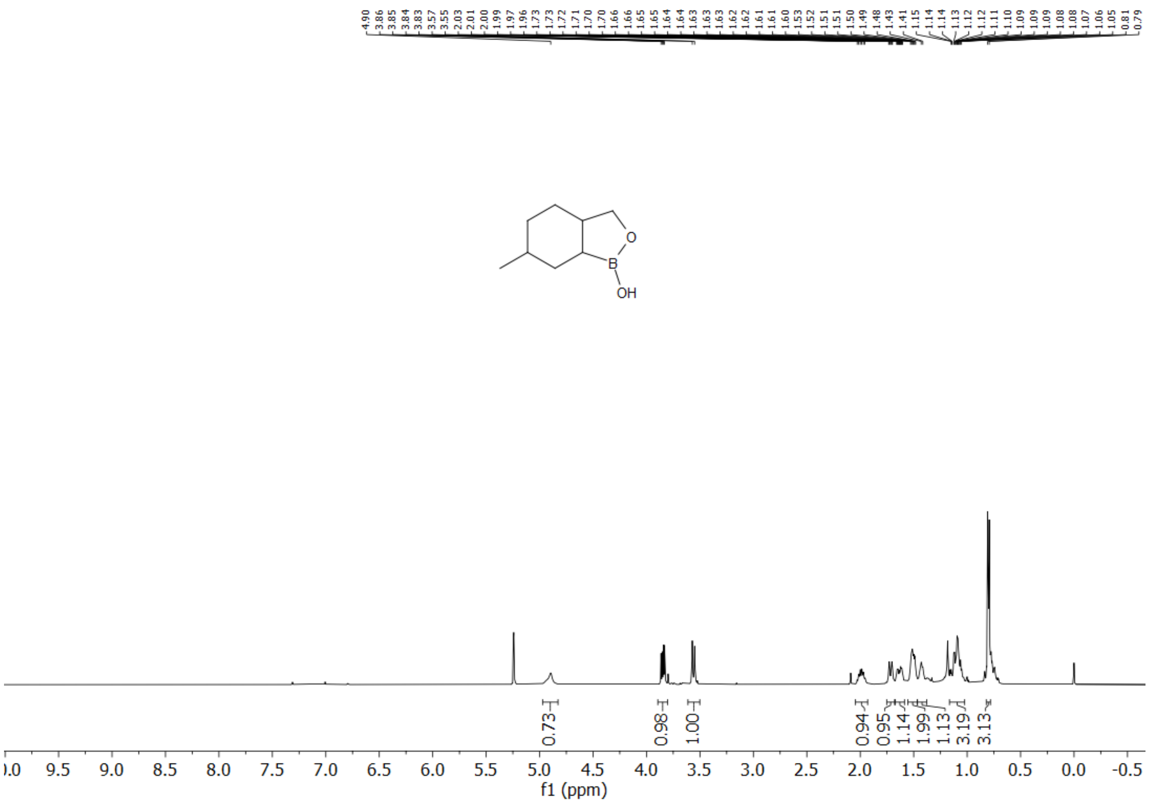

Figure S153: ^{1}H NMR (400 MHz, CD_2Cl_2, 298 K) spectrum of 5-methylhexahydrobenzo[*c*][1,2]oxaborol-1(3*H*)-ol (6e).

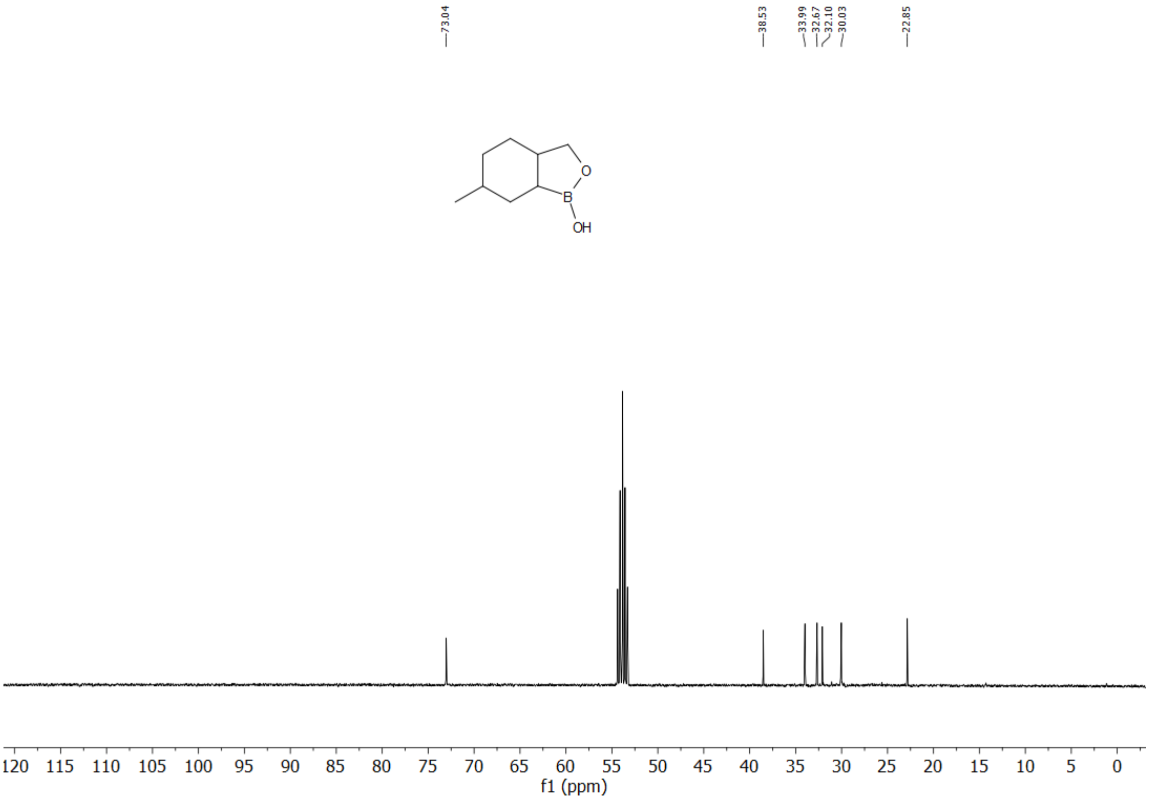

Figure S154: $^{13}C\{^{1}H\}$ NMR (101 MHz, CD_2Cl_2, 298 K) spectrum of 5-methylhexahydrobenzo[*c*][1,2]oxaborol-1(3*H*)-ol (6e).

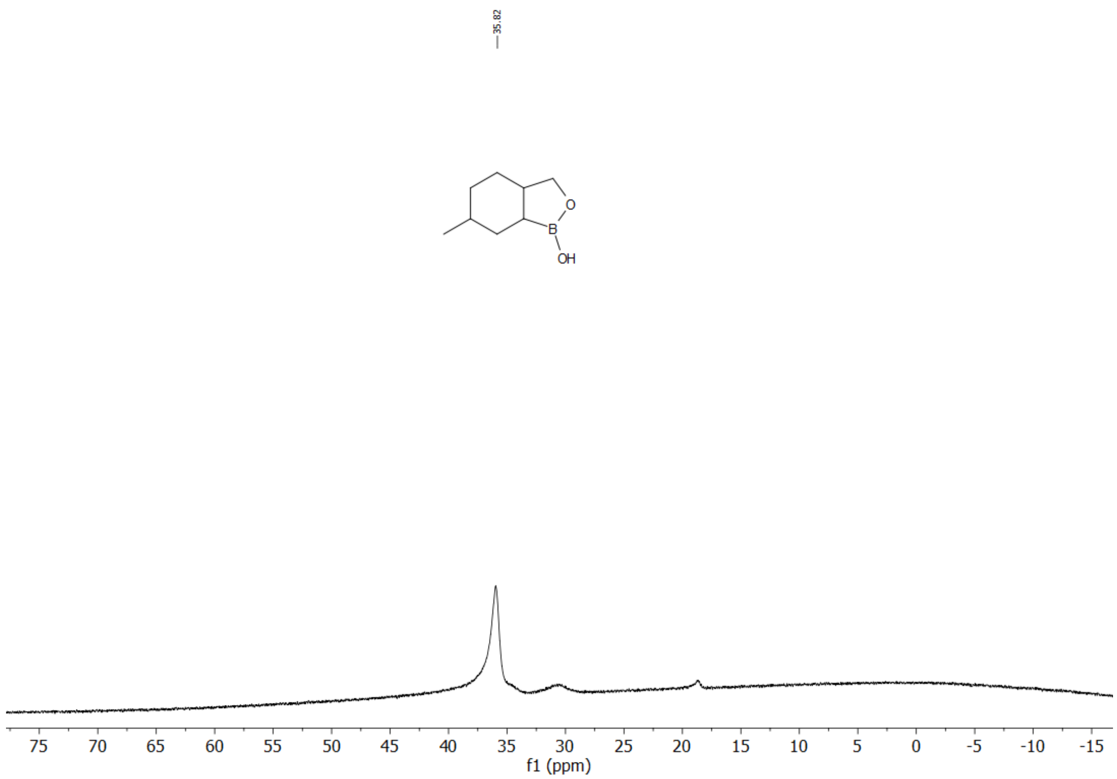

Figure S155: $^{11}B\{^{1}H\}$ NMR (128 MHz, CD_2Cl_2, 298 K) spectrum of 5-methylhexahydrobenzo[*c*][1,2]oxaborol-1(3*H*)-ol (6e).

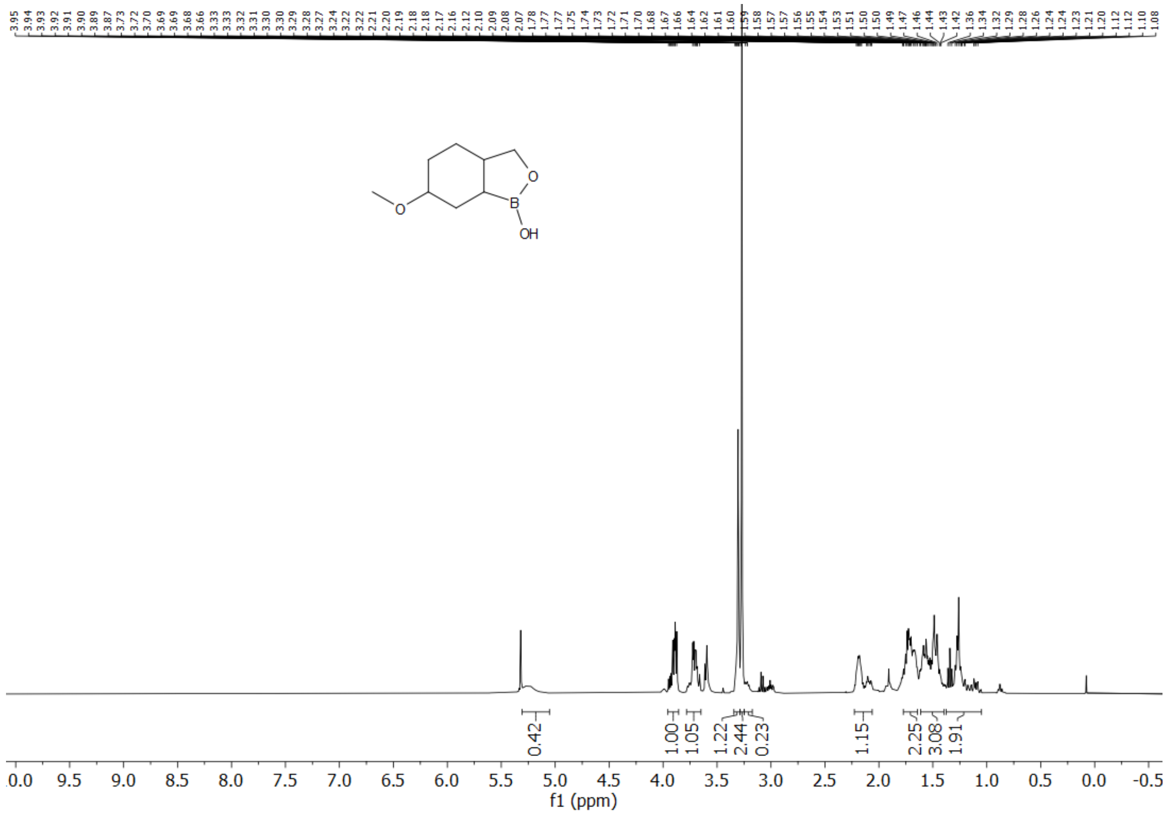

Figure S156: ^{1}H NMR (400 MHz, CD_2Cl_2, 298 K) spectrum of 6-methoxyhexahydrobenzo[*c*][1,2]oxaborol-1(3*H*)-ol (6f).

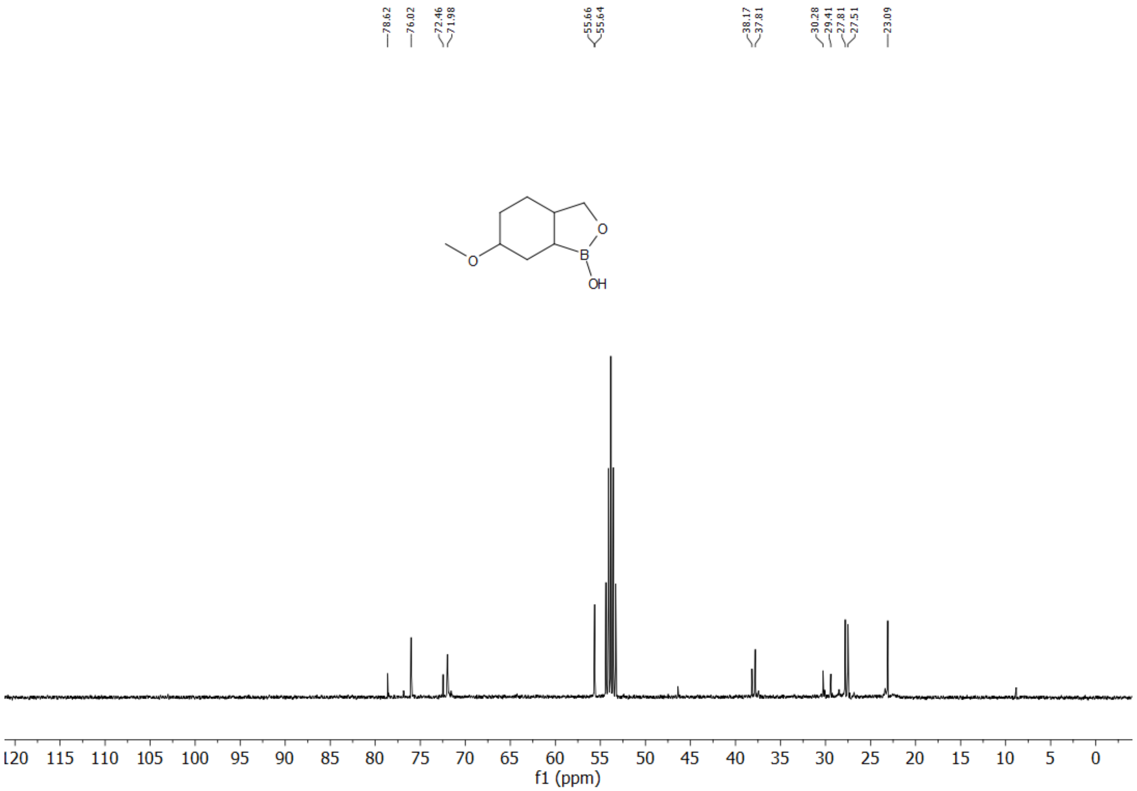

Figure S157: $^{13}C\{^1H\}$ NMR (101 MHz, CD_2Cl_2, 298 K) spectrum of 6-methoxyhexahydrobenzo[*c*][1,2]oxaborol-1(3*H*)-ol (6f).

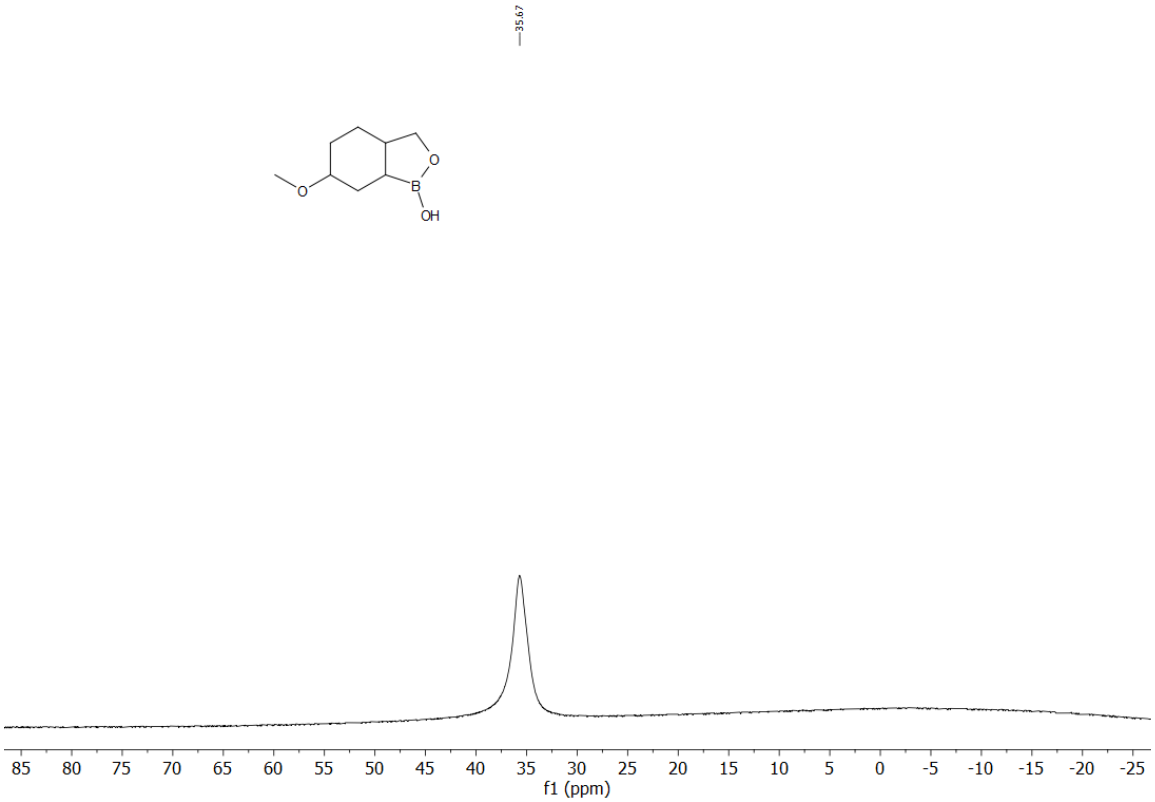

Figure S158: $^{11}B\{^1H\}$ NMR (128 MHz, CD_2Cl_2, 298 K) spectrum of 6-methoxyhexahydrobenzo[*c*][1,2]oxaborol-1(3*H*)-ol (6f).

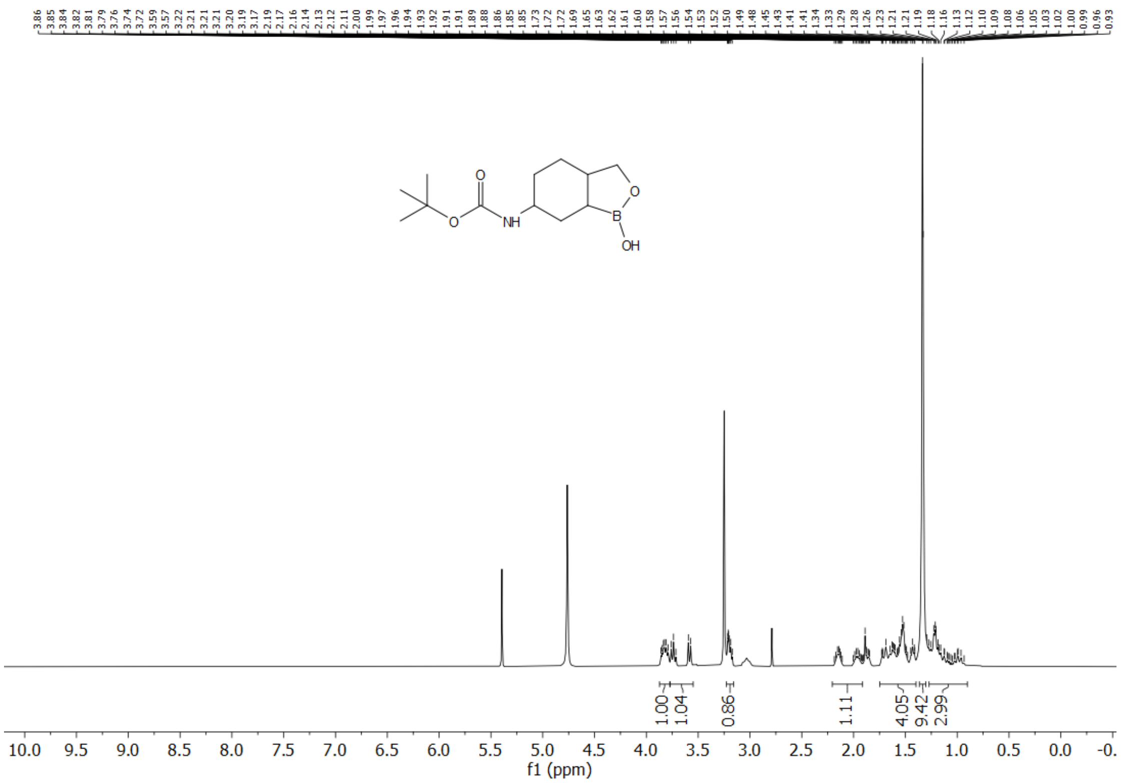

Figure S159: ^{1}H NMR (400 MHz, CD_3OD, 298 K) spectrum of *tert*-butyl (1-hydroxyoctahydrobenzo[*c*][1,2]oxaborol-6-yl)carbamate (6g).

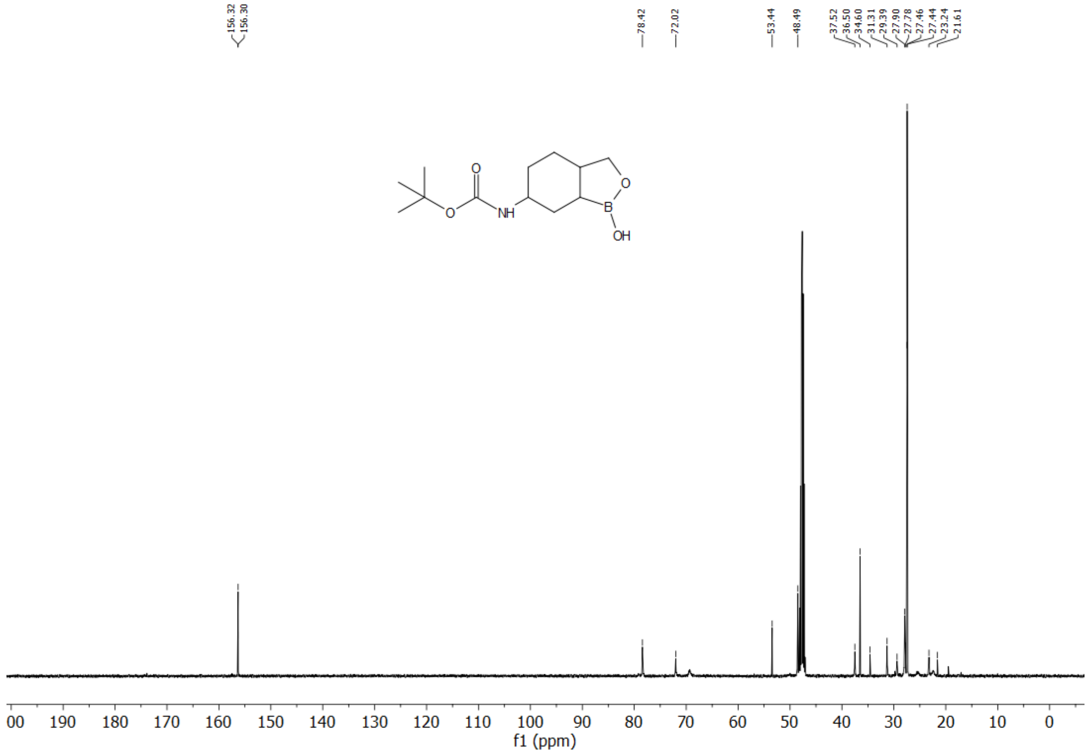

Figure S160: $^{13}C\{^{1}H\}$ NMR (101 MHz, CD_3OD, 298 K) spectrum of *tert*-butyl (1-hydroxyoctahydrobenzo[*c*][1,2]oxaborol-6-yl)carbamate (6g).

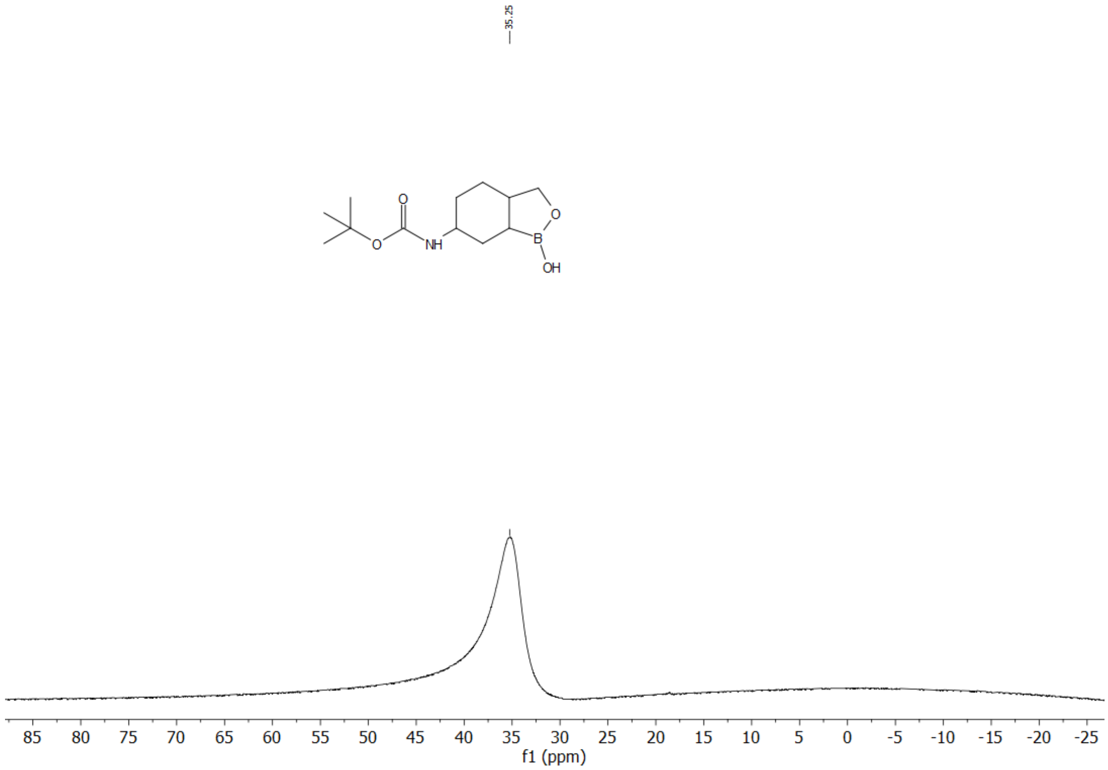

Figure S161: $^{11}B\{^{1}H\}$ NMR (128 MHz, CD_3OD, 298 K) spectrum of *tert*-butyl (1-hydroxyoctahydrobenzo[*c*][1,2]oxaborol-6-yl)carbamate (6g).

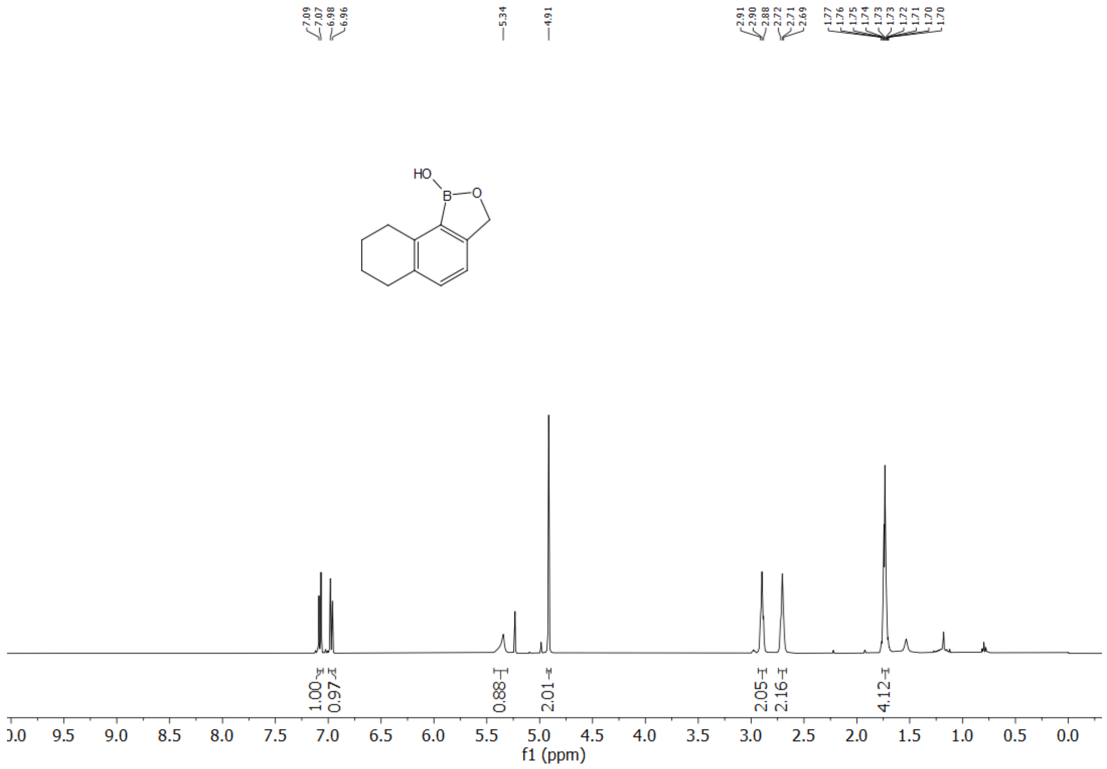

Figure S162: ^{1}H NMR (400 MHz, CD_2Cl_2, 298 K) spectrum of 5a,6,7,8,9,9a-hexahydronaphtho[1,2-*c*][1,2]oxaborol-1(3*H*)-ol (6h).

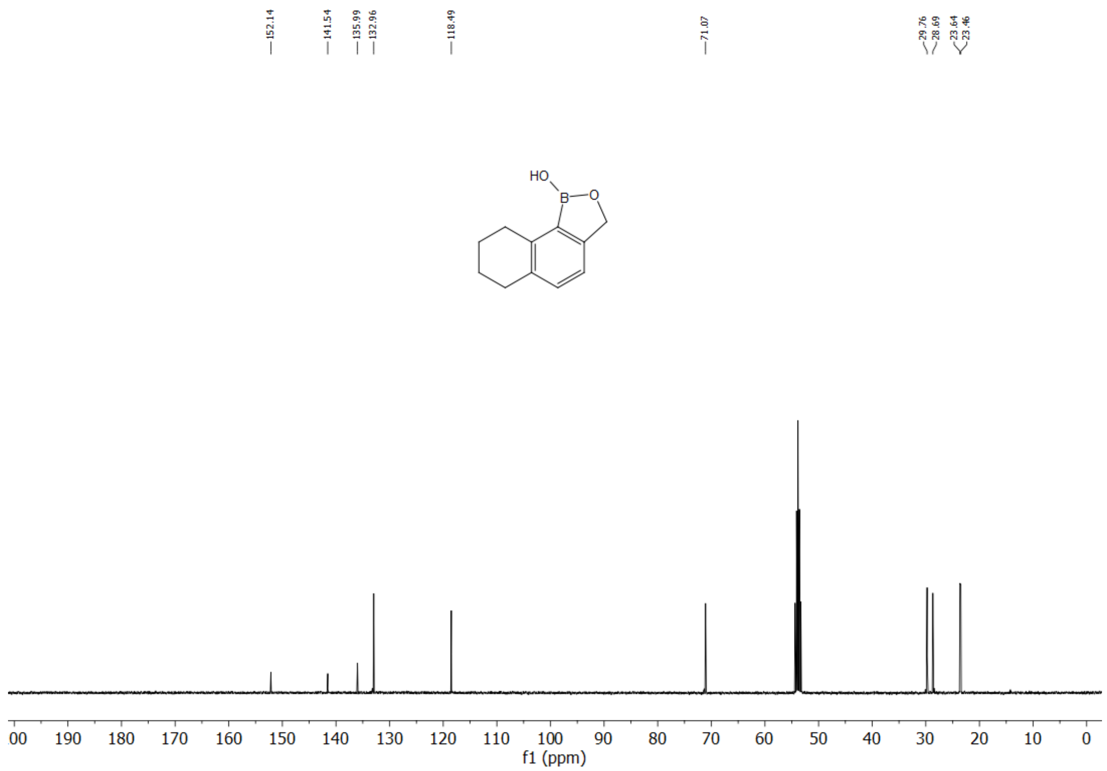

Figure S163: $^{13}C\{^1H\}$ NMR (101 MHz, CD_2Cl_2, 298 K) spectrum of 5a,6,7,8,9,9a-hexahydronaphtho[1,2-*c*][1,2]oxaborol-1(3*H*)-ol (6h).

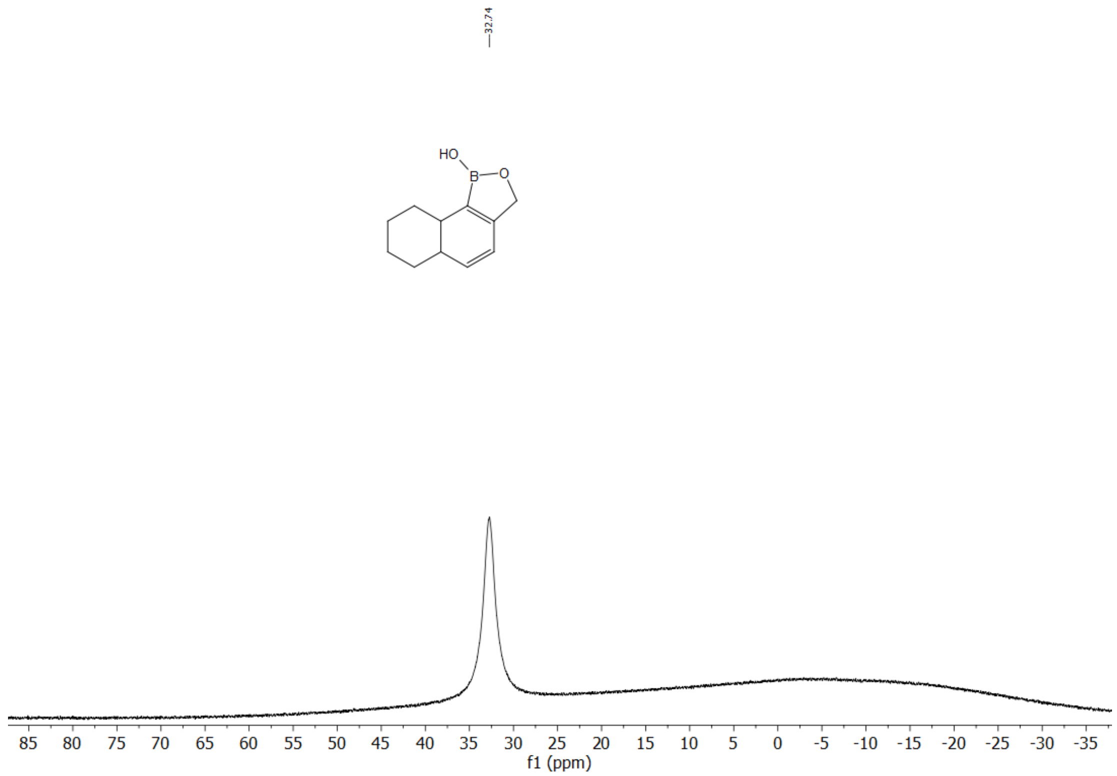

Figure S164: $^{11}B\{^1H\}$ NMR (128 MHz, CD_2Cl_2, 298 K) spectrum of 5a,6,7,8,9,9a-hexahydronaphtho[1,2-*c*][1,2]oxaborol-1(3*H*)-ol (6h).

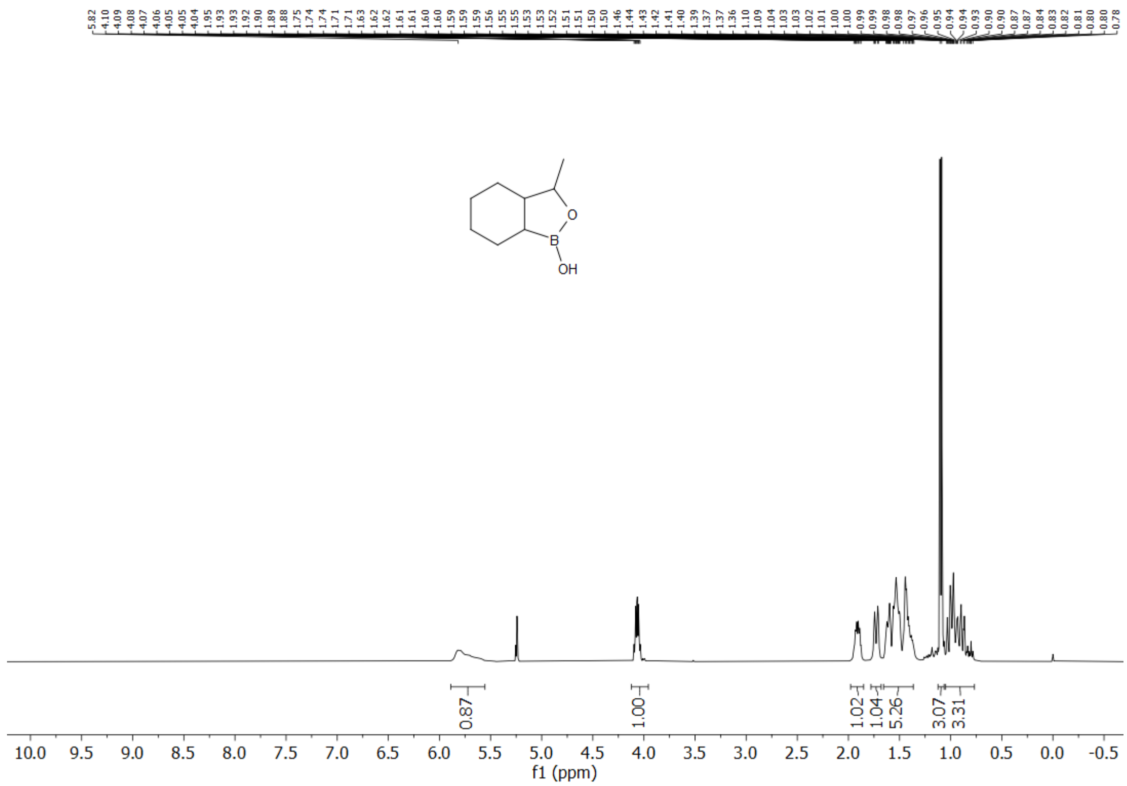

Figure S165: ^{1}H NMR (400 MHz, CD_2Cl_2, 298 K) spectrum of 3-methylhexahydrobenzo[*c*][1,2]oxaborol-1(3*H*)-ol (6k).

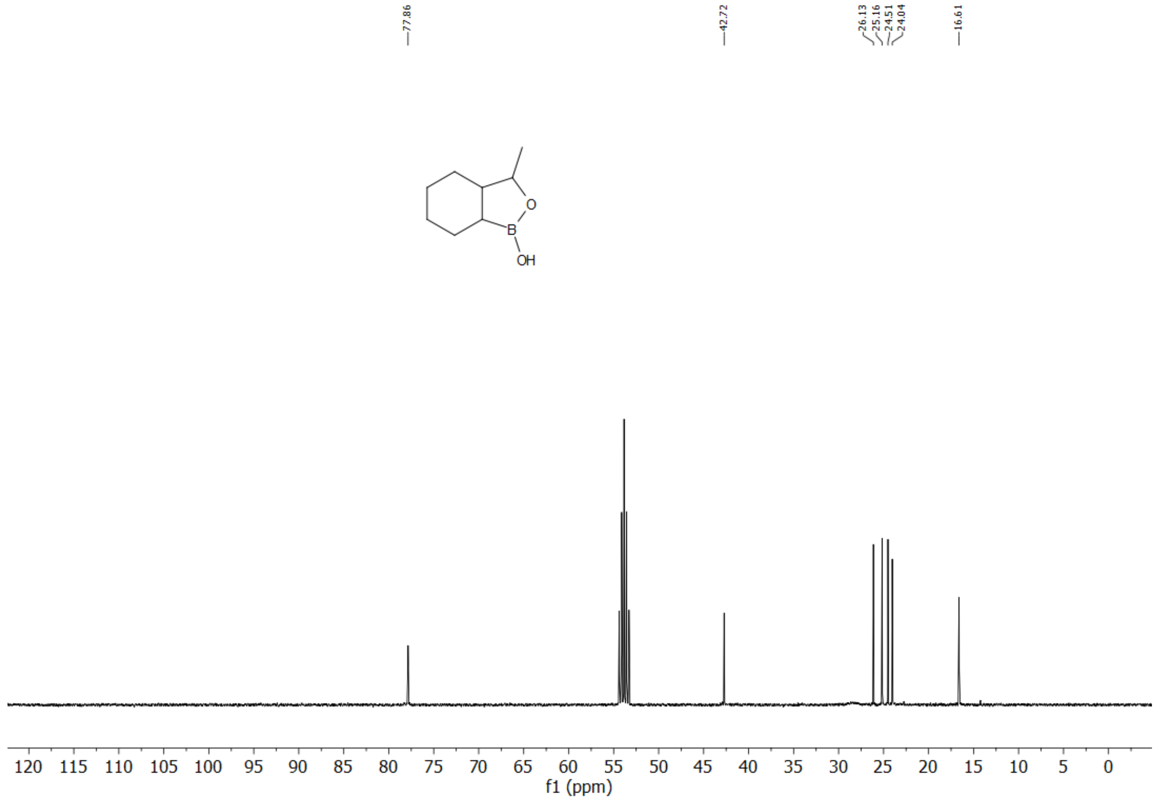

Figure S166: $^{13}C\{^{1}H\}$ NMR (101 MHz, CD_2Cl_2, 298 K) spectrum of 3-methylhexahydrobenzo[*c*][1,2]oxaborol-1(3*H*)-ol (6k).

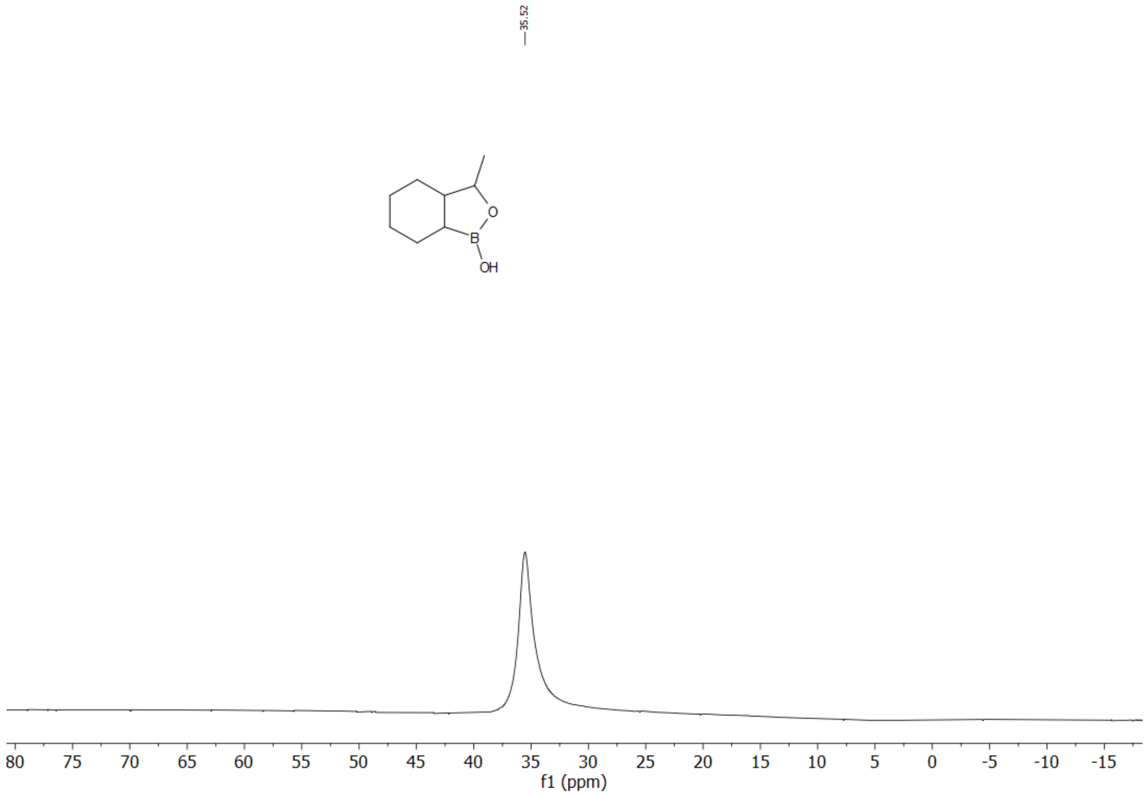

Figure S167: $^{11}B\{^{1}H\}$ NMR (128 MHz, CD_2Cl_2, 298 K) spectrum of 3-methylhexahydrobenzo[*c*][1,2]oxaborol-1(3*H*)-ol (6k).

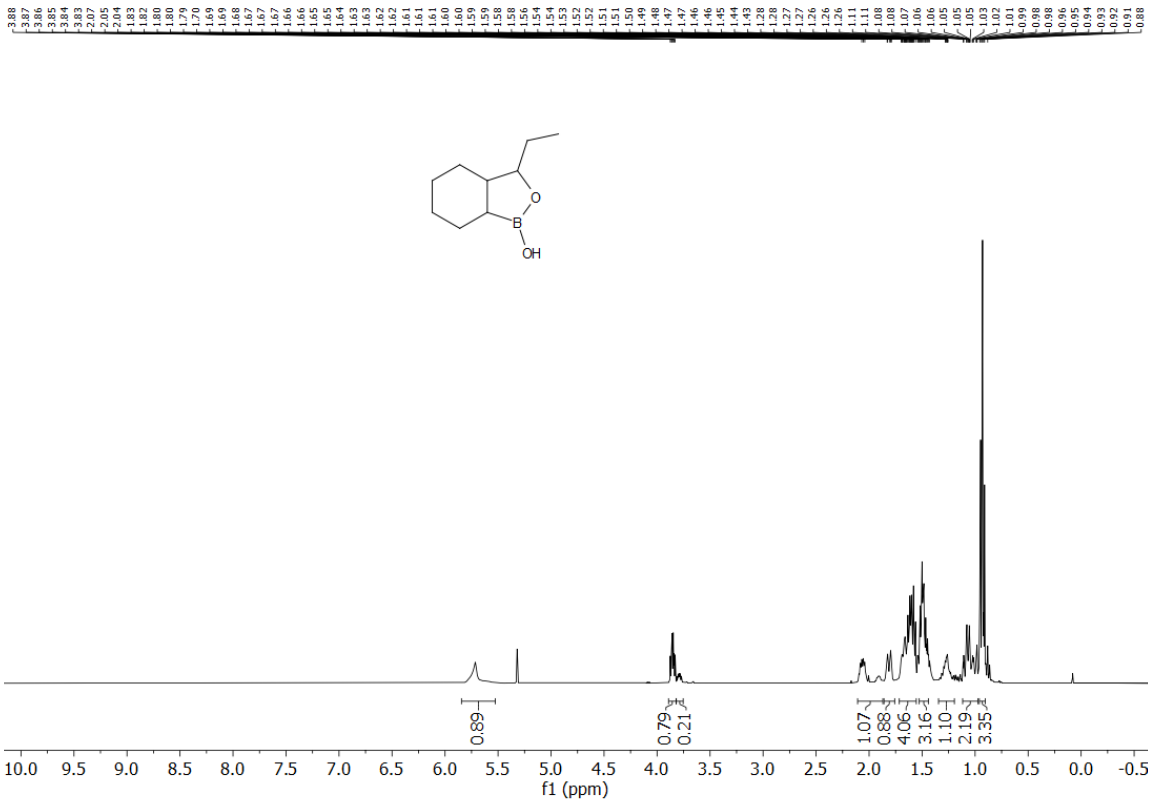

Figure S168: ^{1}H NMR (400 MHz, CD_2Cl_2, 298 K) spectrum of 3-ethylhexahydrobenzo[*c*][1,2]oxaborol-1(3*H*)-ol (6l).

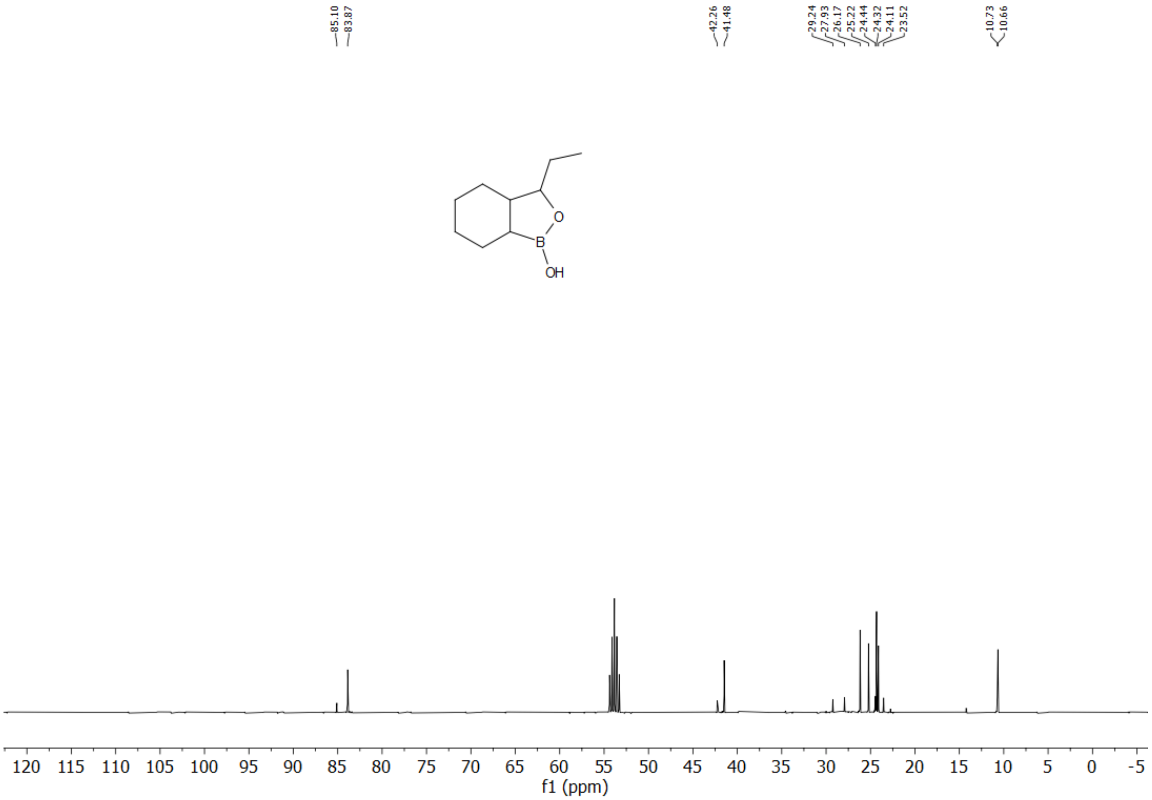

Figure S169: $^{13}C\{^1H\}$ NMR (101 MHz, CD_2Cl_2, 298 K) spectrum of 3-ethylhexahydrobenzo[*c*][1,2]oxaborol-1(3*H*)-ol (6l).

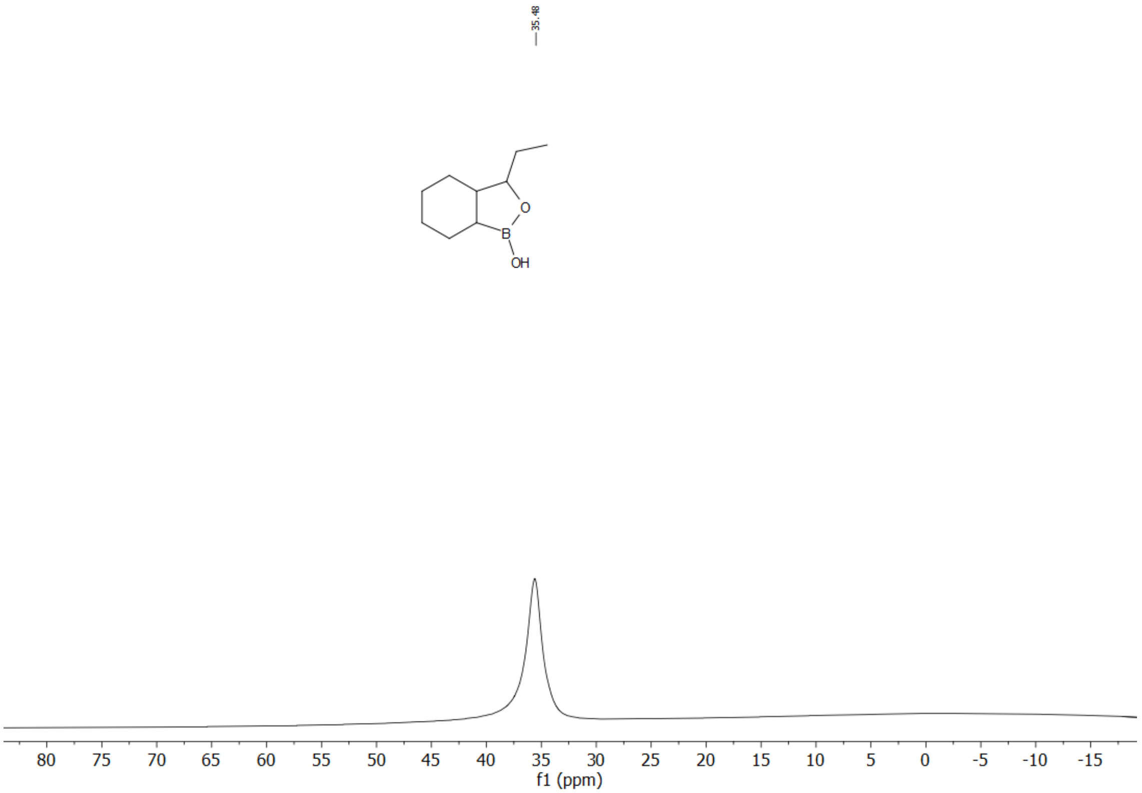

Figure S170: $^{11}B\{^1H\}$ NMR (128 MHz, CD_2Cl_2, 298 K) spectrum of 3-ethylhexahydrobenzo[*c*][1,2]oxaborol-1(3*H*)-ol (6l).

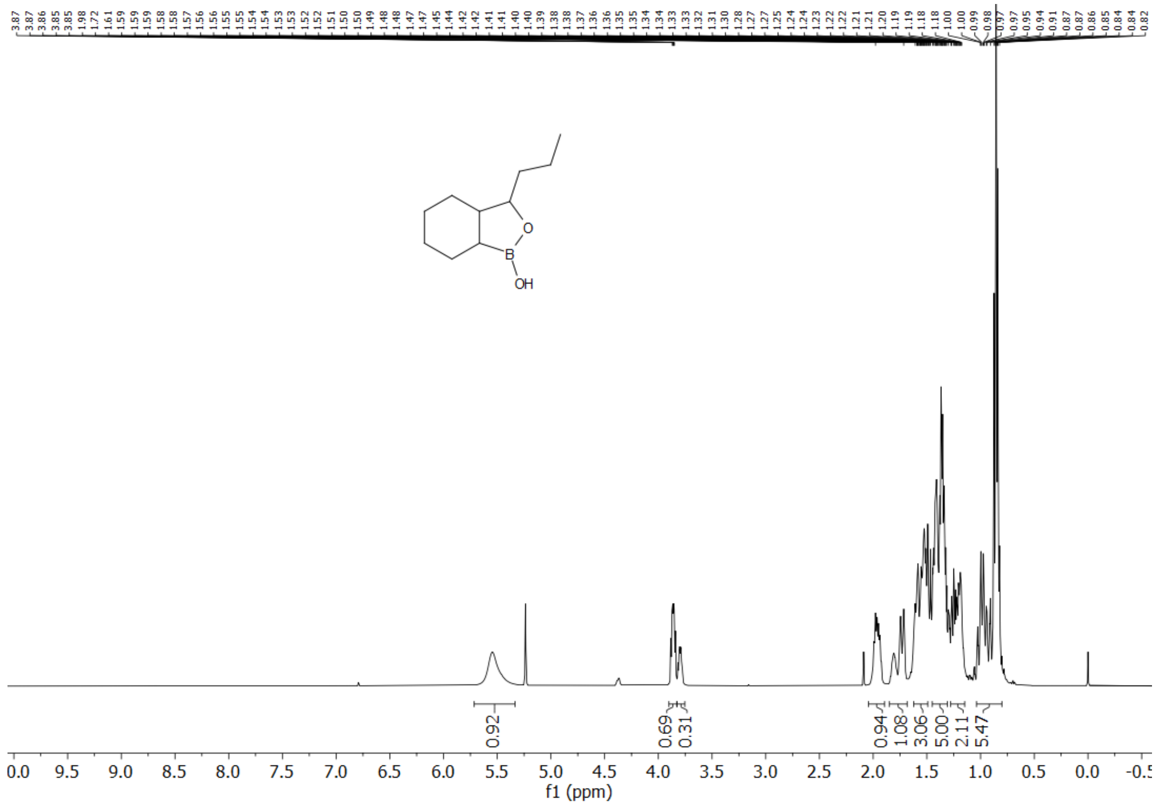

Figure S171: ^{1}H NMR (400 MHz, CD_2Cl_2, 298 K) spectrum of 3-propylhexahydrobenzo[*c*][1,2]oxaborol-1(3*H*)-ol (6m).

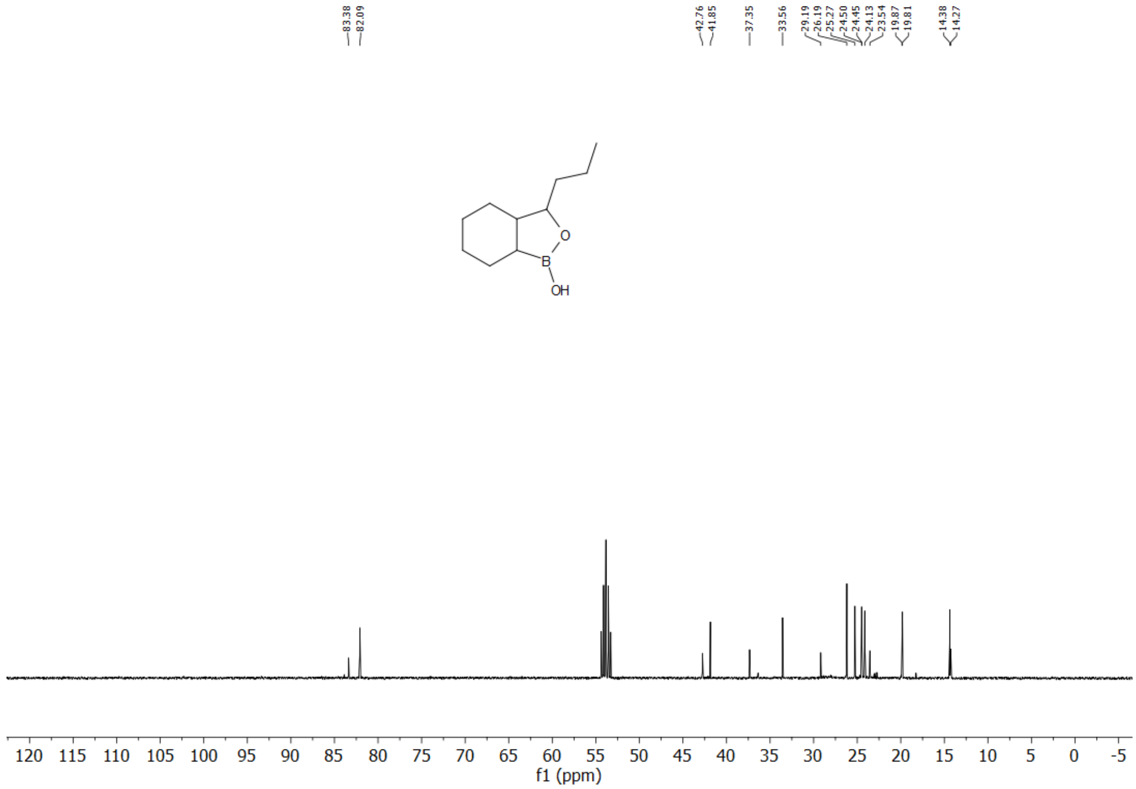

Figure S172: $^{13}C\{^{1}H\}$ NMR (101 MHz, CD_2Cl_2, 298 K) spectrum of 3-propylhexahydrobenzo[*c*][1,2]oxaborol-1(3*H*)-ol (6m).

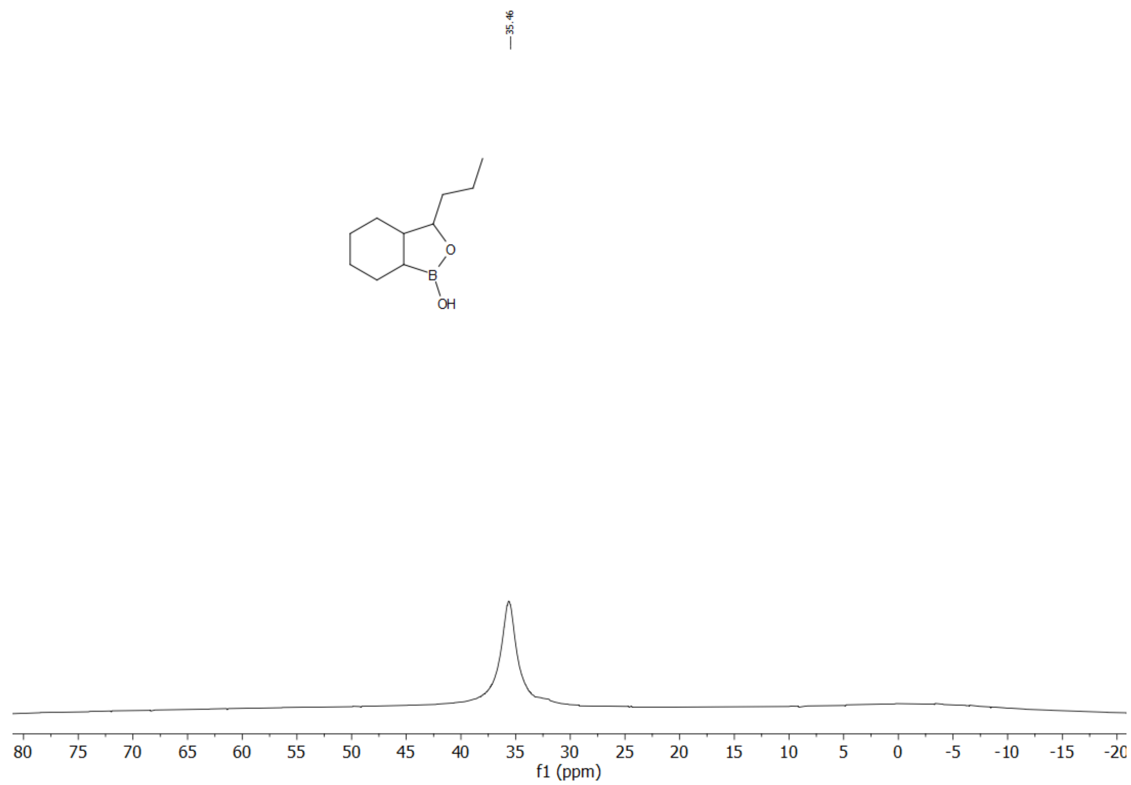

Figure S173: $^{11}B\{^{1}H\}$ NMR (128 MHz, CD_2Cl_2, 298 K) spectrum of 3-propylhexahydrobenzo[*c*][1,2]oxaborol-1(3*H*)-ol (6m).

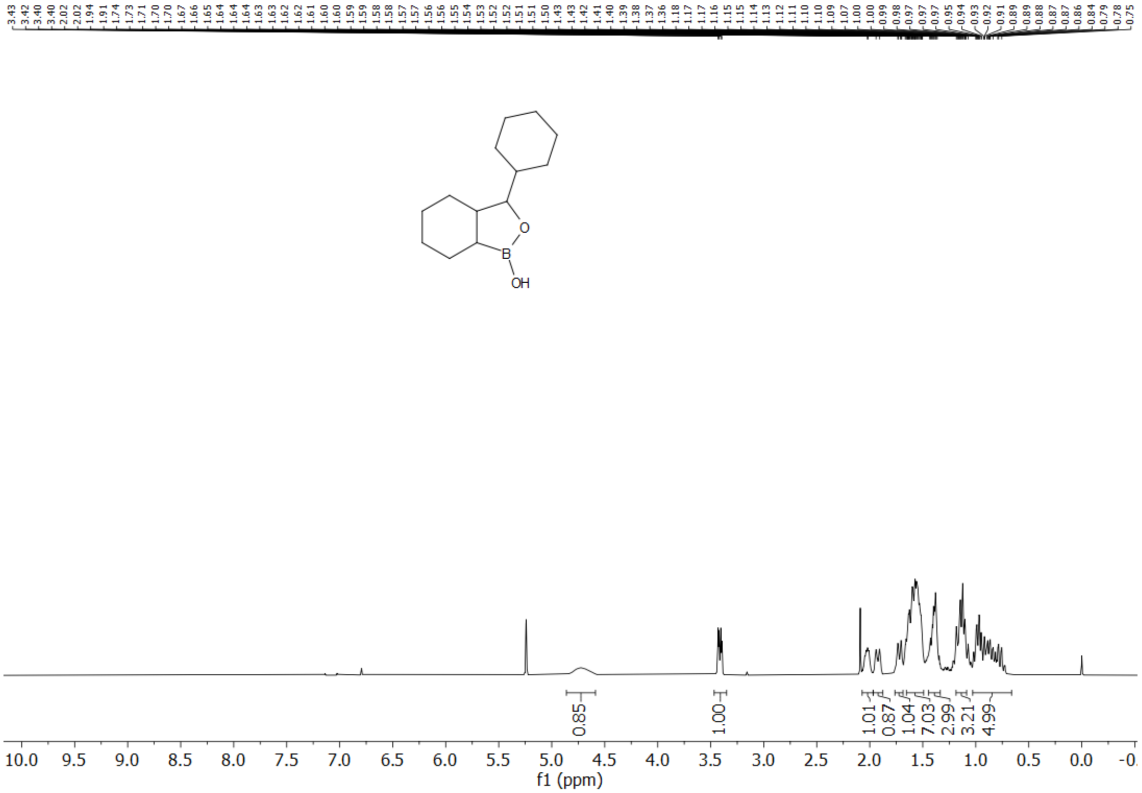

Figure S174: ^{1}H NMR (400 MHz, CD_2Cl_2, 298 K) spectrum of 3-cyclohexylhexahydrobenzo[*c*][1,2]oxaborol-1(3*H*)-ol (6n).

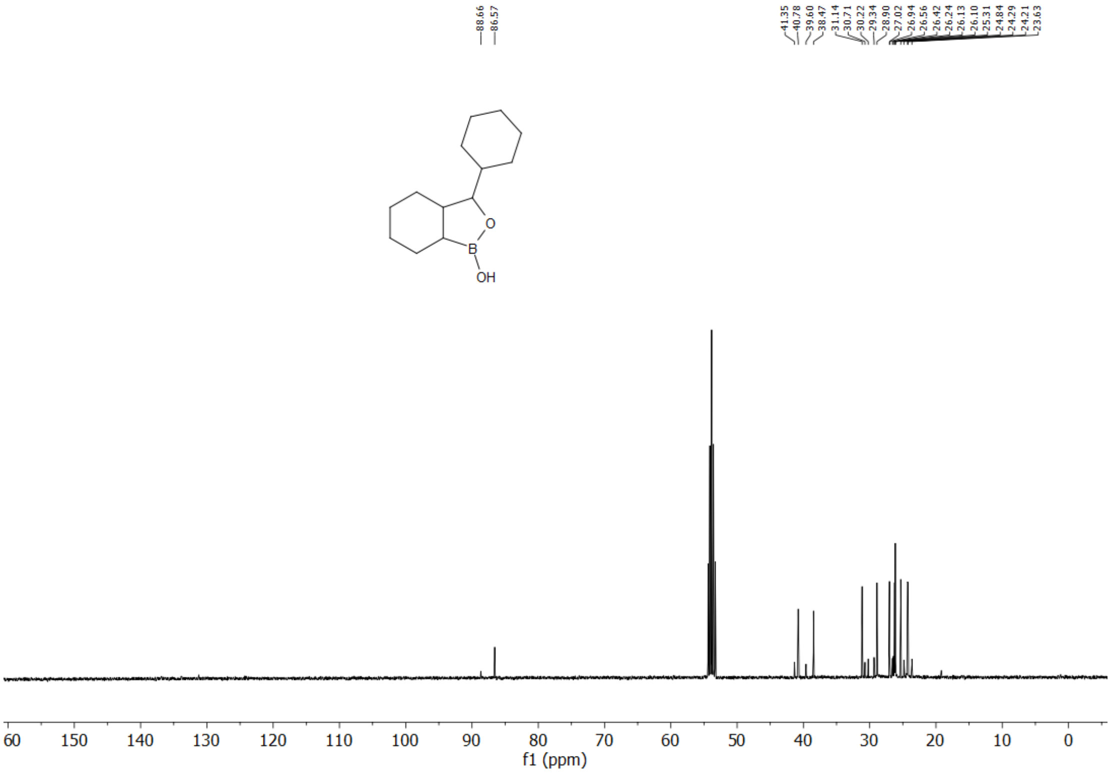

Figure S175: $^{13}C\{^{1}H\}$ NMR (101 MHz, CD_2Cl_2, 298 K) spectrum of 3-cyclohexylhexahydrobenzo[*c*][1,2]oxaborol-1(3*H*)-ol (6n).

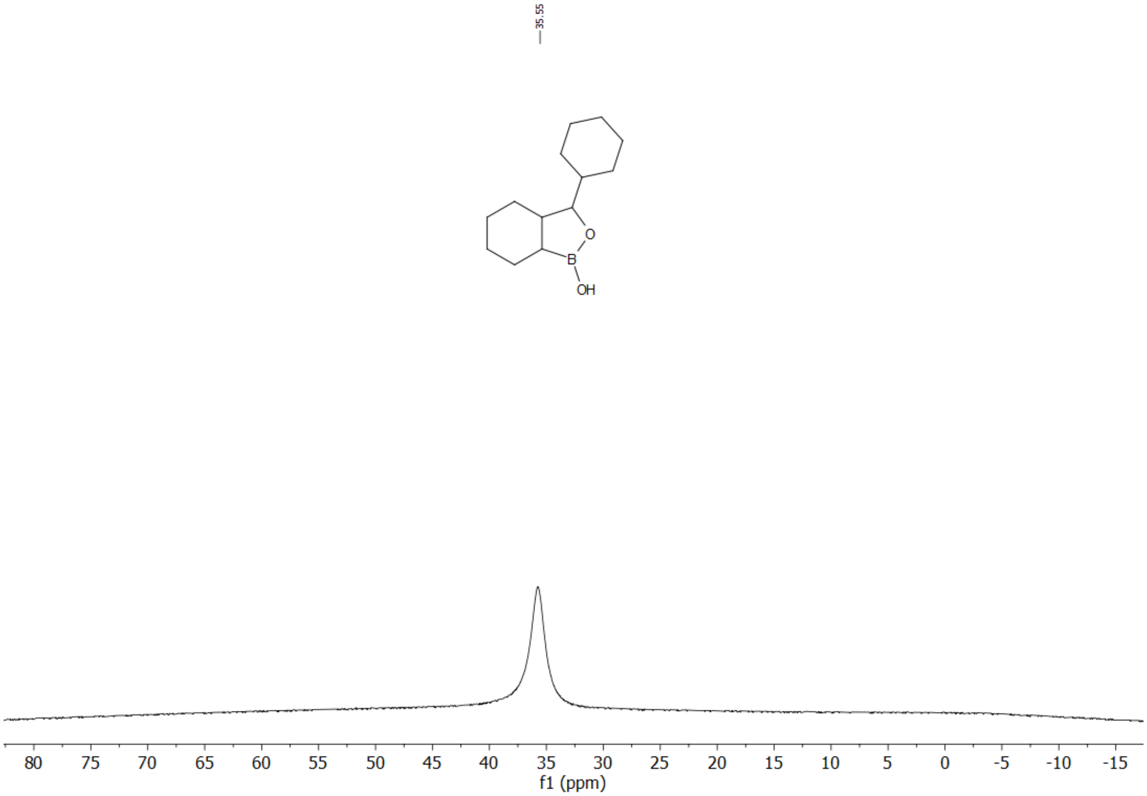

Figure S176: $^{11}B\{^{1}H\}$ NMR (128 MHz, CD_2Cl_2, 298 K) spectrum of 3-cyclohexylhexahydrobenzo[*c*][1,2]oxaborol-1(3*H*)-ol (6n).

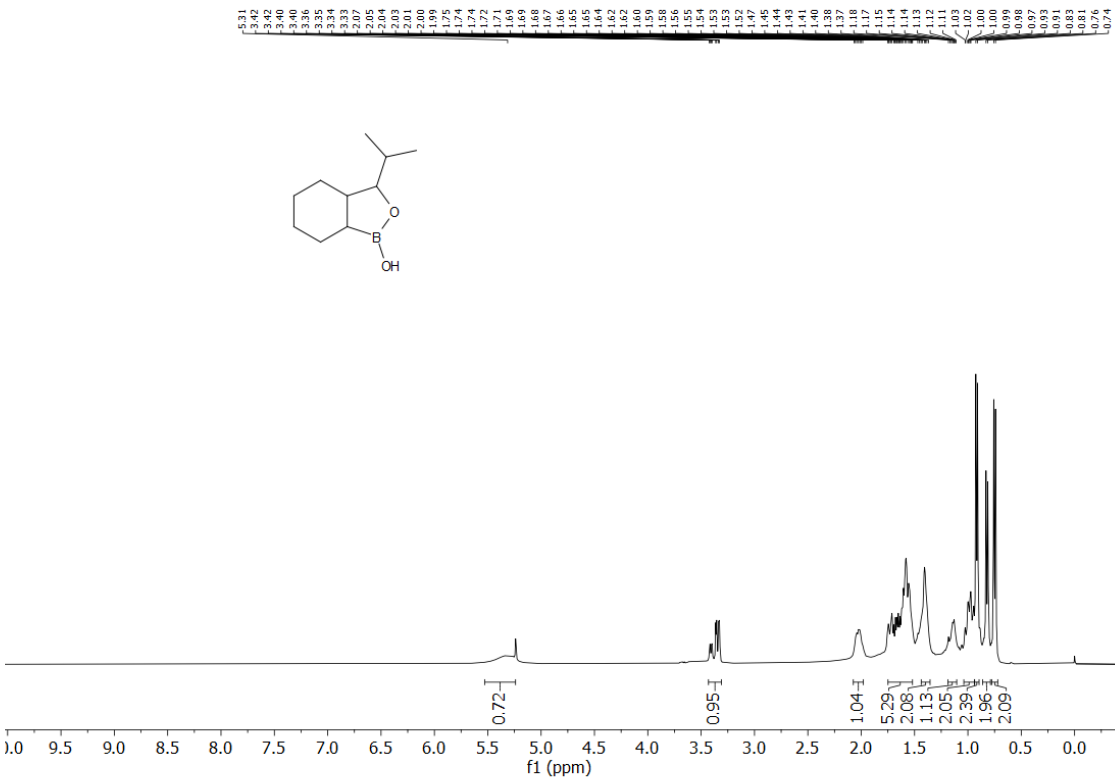

Figure S177: ^{1}H NMR (400 MHz, CD_2Cl_2, 298 K) spectrum of 3-isopropylhexahydrobenzo[*c*][1,2]oxaborol-1(3*H*)-ol (6o).

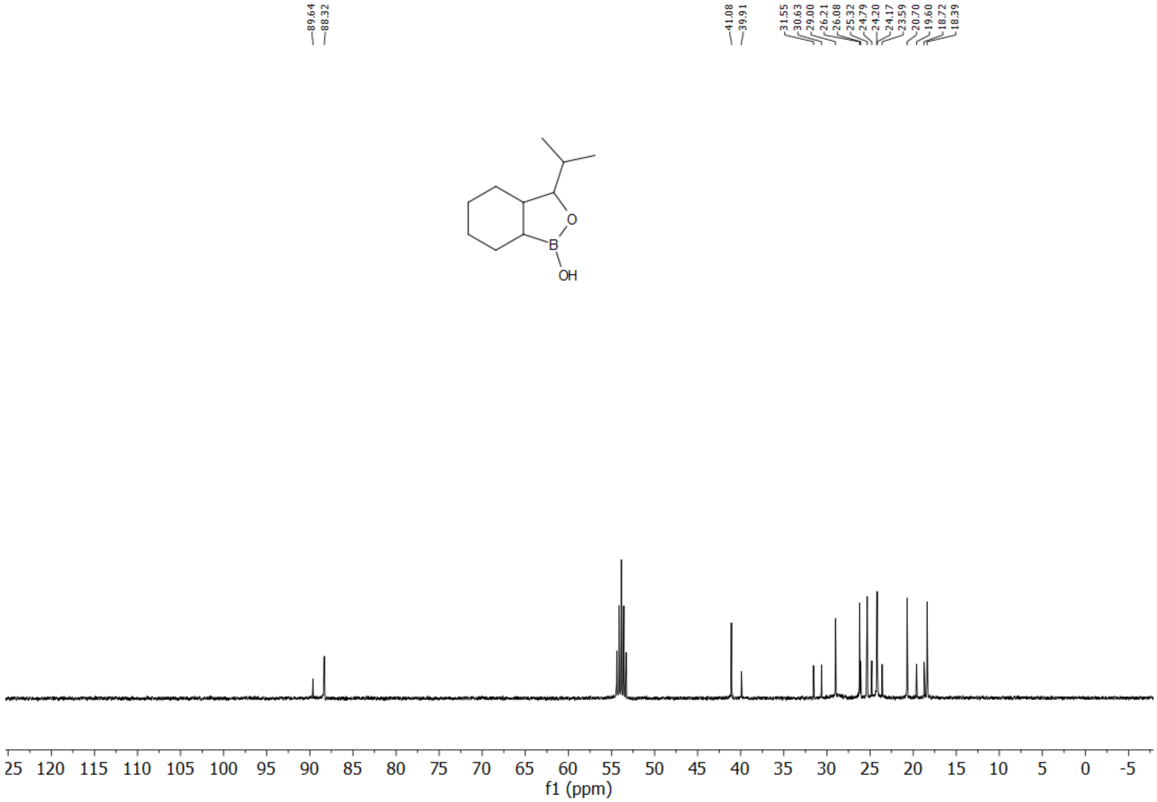

Figure S 178: $^{13}C\{^{1}H\}$ NMR (101 MHz, CD_2Cl_2, 298 K) spectrum of 3-isopropylhexahydrobenzo[*c*][1,2]oxaborol-1(3*H*)-ol (6o).

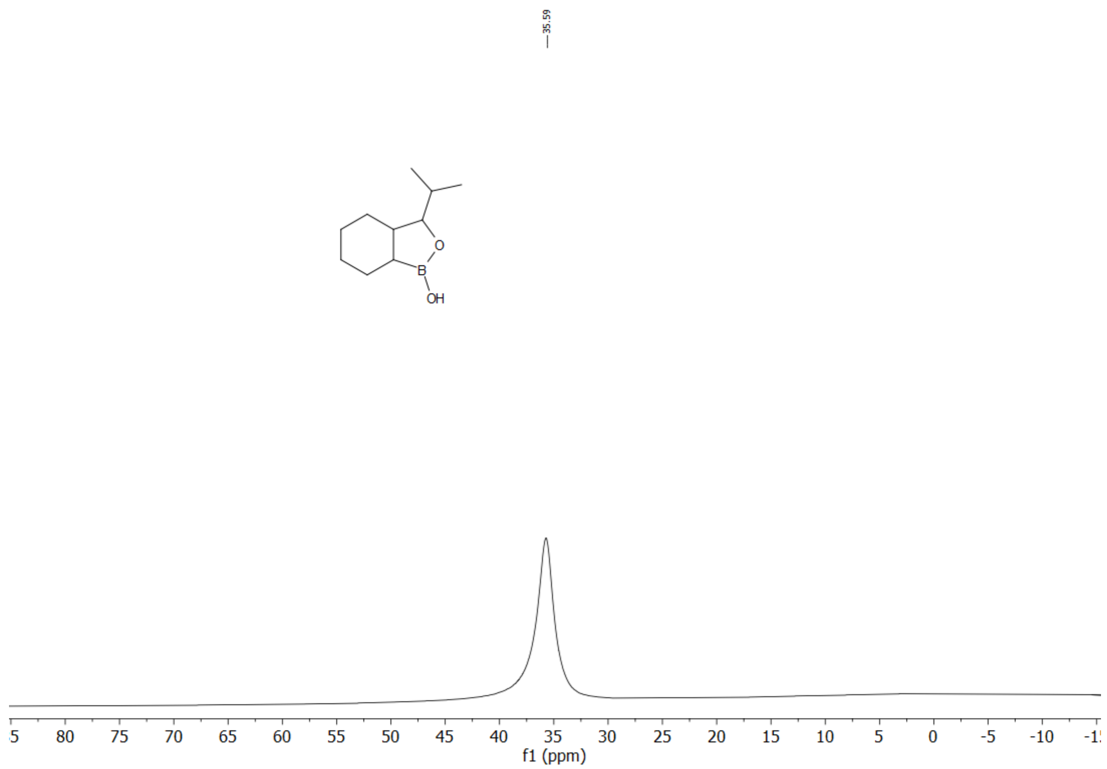

Figure S179: $^{11}B\{^1H\}$ NMR (128 MHz, CD_2Cl_2, 298 K) spectrum of 3-isopropylhexahydrobenzo[*c*][1,2]oxaborol-1(3*H*)-ol (6o).

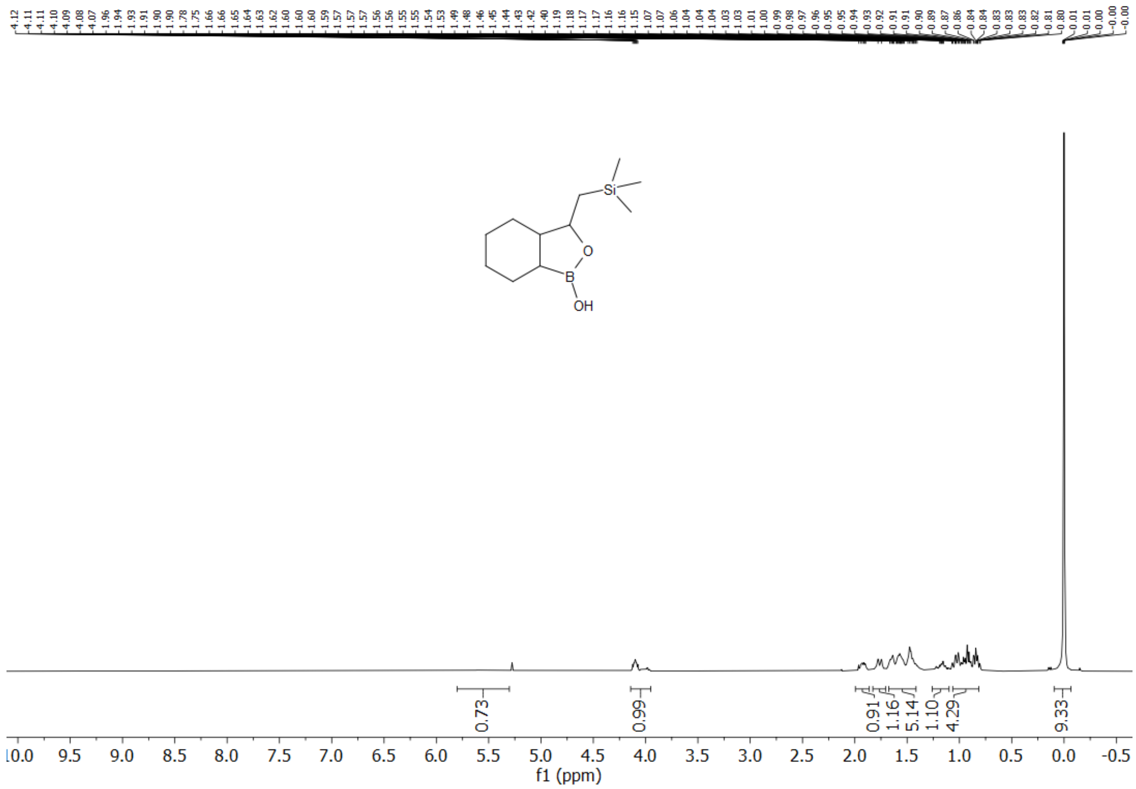

Figure S180: ^1H NMR (400 MHz, CD_2Cl_2, 298 K) spectrum of 3-((trimethylsilyl)methyl)hexahydrobenzo[*c*][1,2]oxaborol-1(3*H*)-ol (6p).

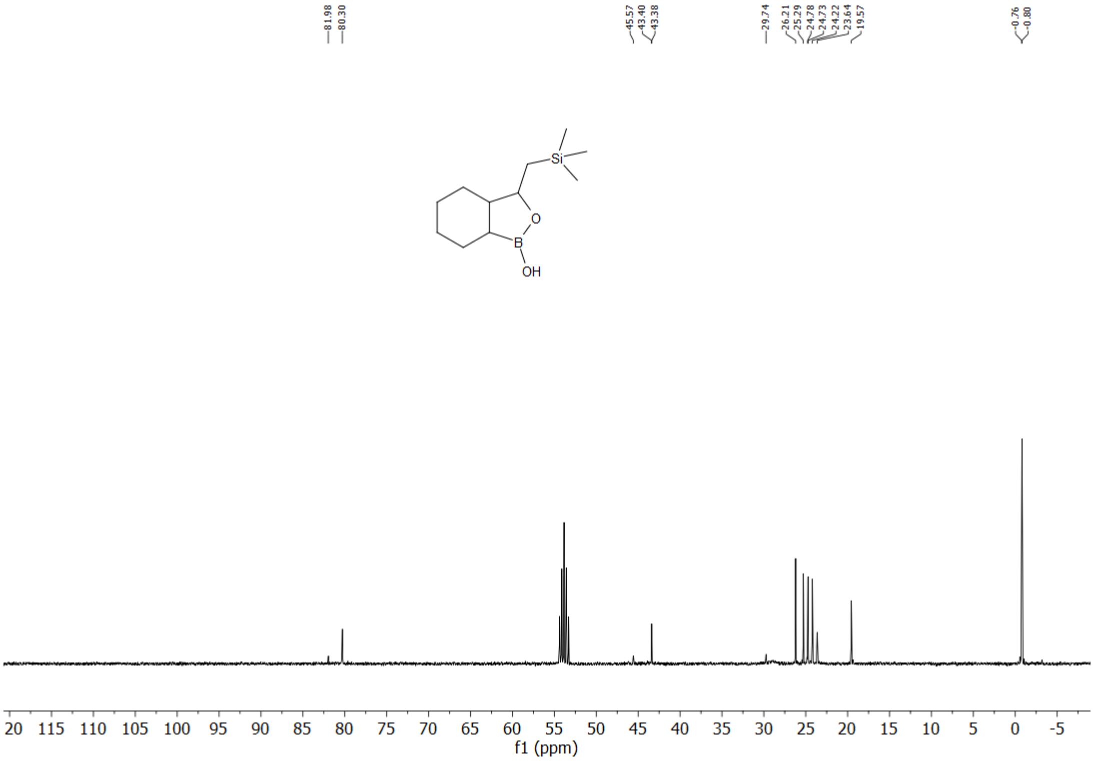

Figure S181: $^{13}C\{^1H\}$ NMR (101 MHz, CD_2Cl_2, 298 K) spectrum of 3-((trimethylsilyl)methyl)hexahydrobenzo[*c*][1,2]oxaborol-1(3*H*)-ol (6p).

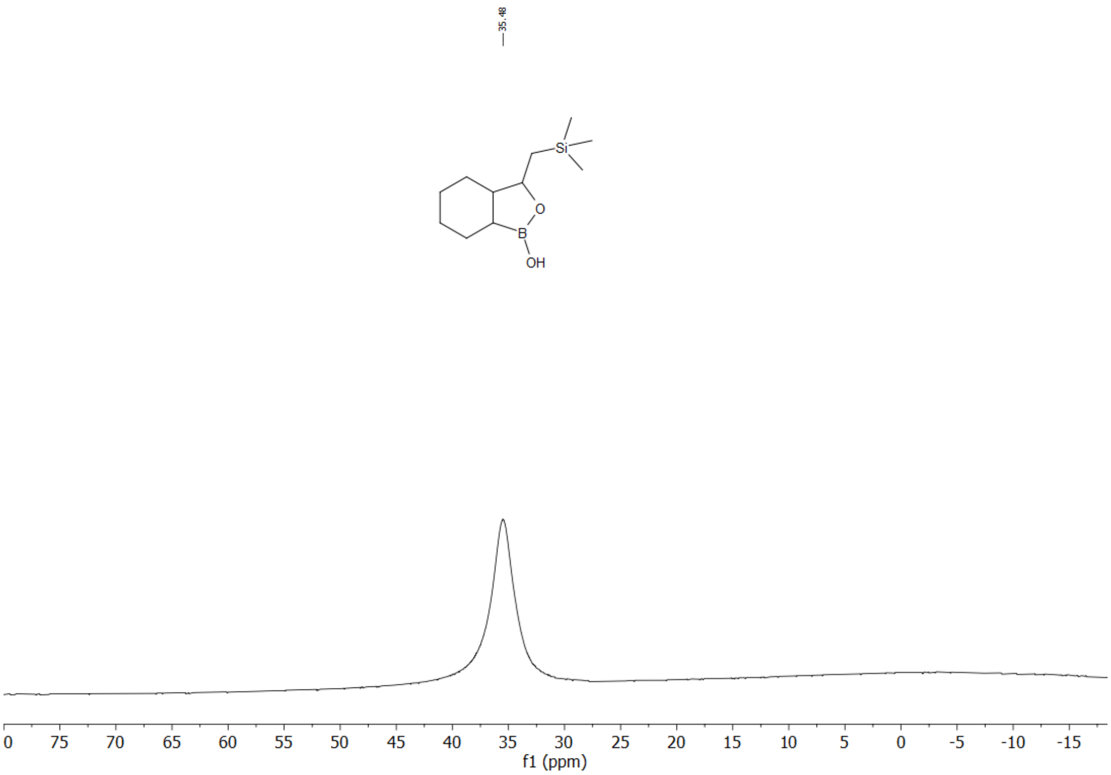

Figure S182: $^{11}B\{^1H\}$ NMR (128 MHz, CD_2Cl_2, 298 K) spectrum of 3-((trimethylsilyl)methyl)hexahydrobenzo[*c*][1,2]oxaborol-1(3*H*)-ol (6p).

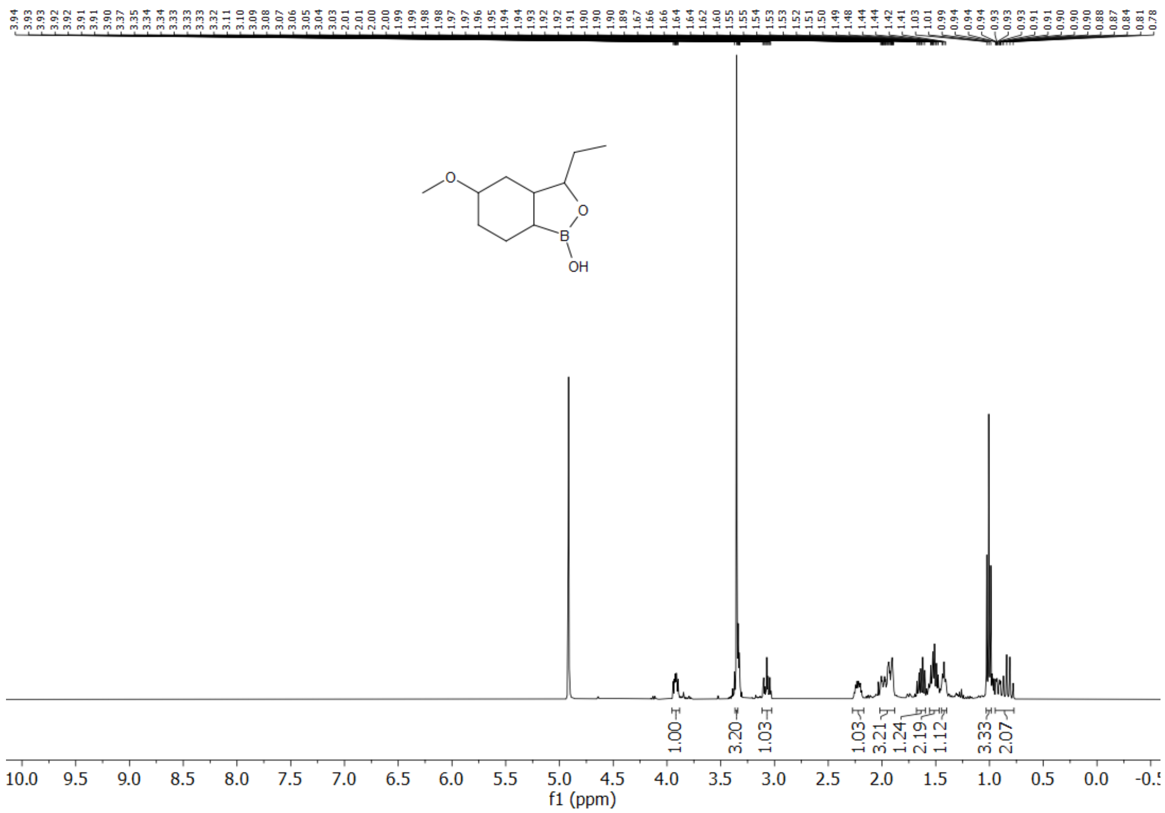

Figure S183: ^{1}H NMR (400 MHz, CD_2Cl_2, 298 K) spectrum of 3-ethyl-5-methoxyhexahydrobenzo[*c*][1,2]oxaborol-1(3*H*)-ol (6q).

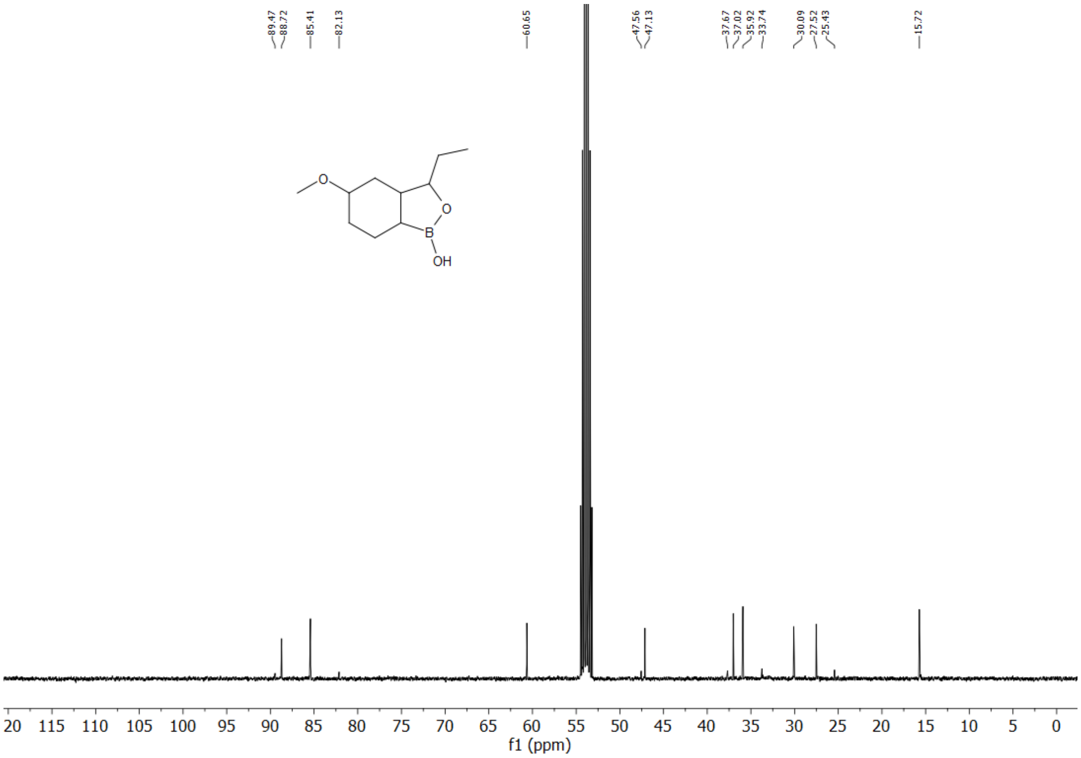

Figure S184: $^{13}C\{^1H\}$ NMR (101 MHz, CD_2Cl_2, 298 K) spectrum of 3-ethyl-5-methoxyhexahydrobenzo[*c*][1,2]oxaborol-1(3*H*)-ol (6q).

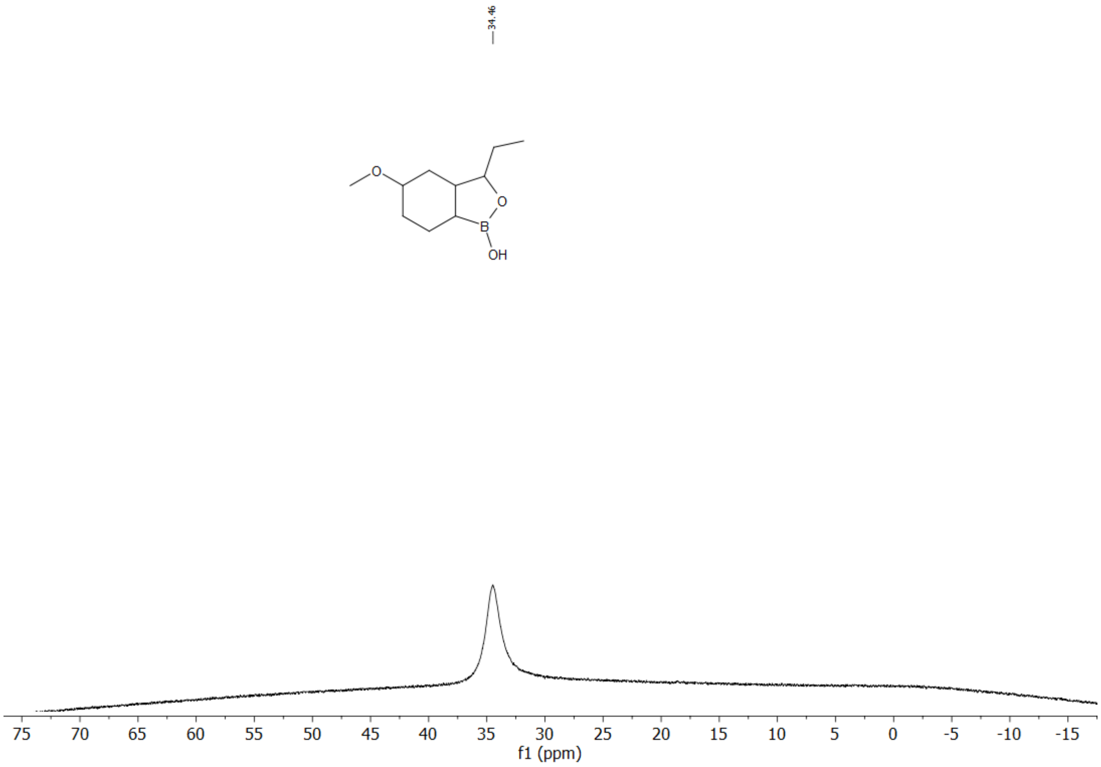

Figure S185: $^{11}B\{^1H\}$ NMR (128 MHz, CD_2Cl_2, 298 K) spectrum of 3-ethyl-5-methoxyhexahydrobenzo[*c*][1,2]oxaborol-1(3*H*)-ol (6q).

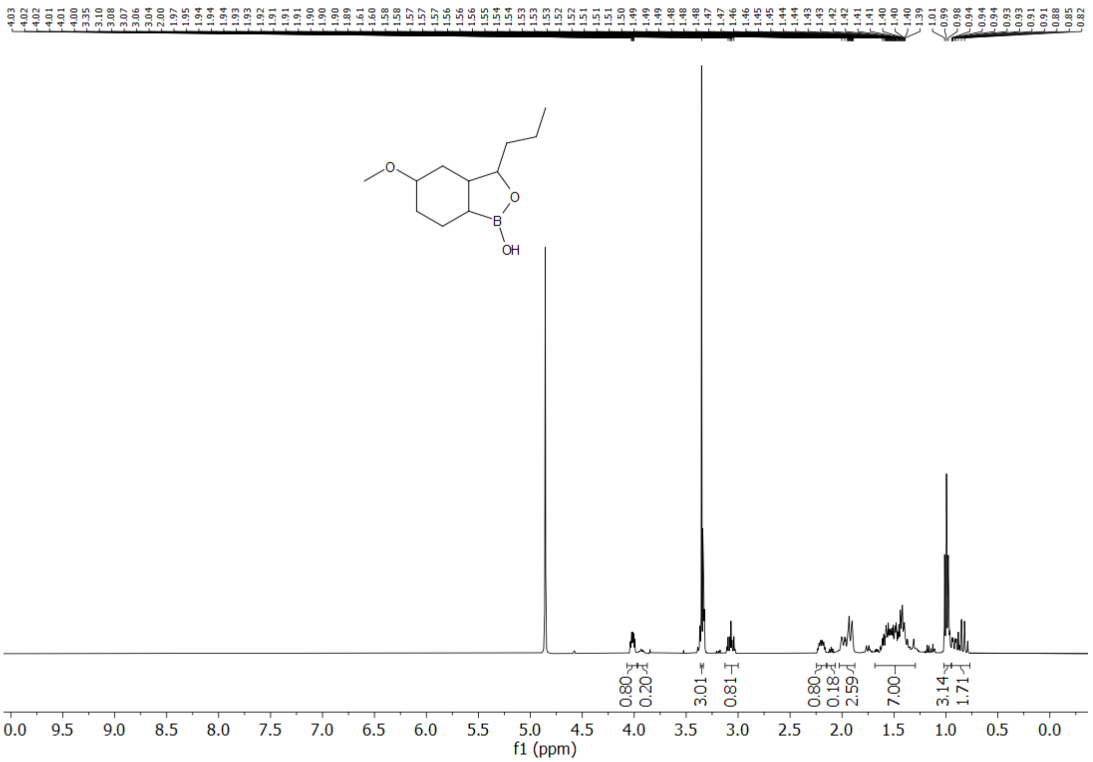

Figure S186: ^1H NMR (400 MHz, CD_3OD, 298 K) spectrum of 5-methoxy-3-propylhexahydrobenzo[*c*][1,2]oxaborol-1(3*H*)-ol (6r).

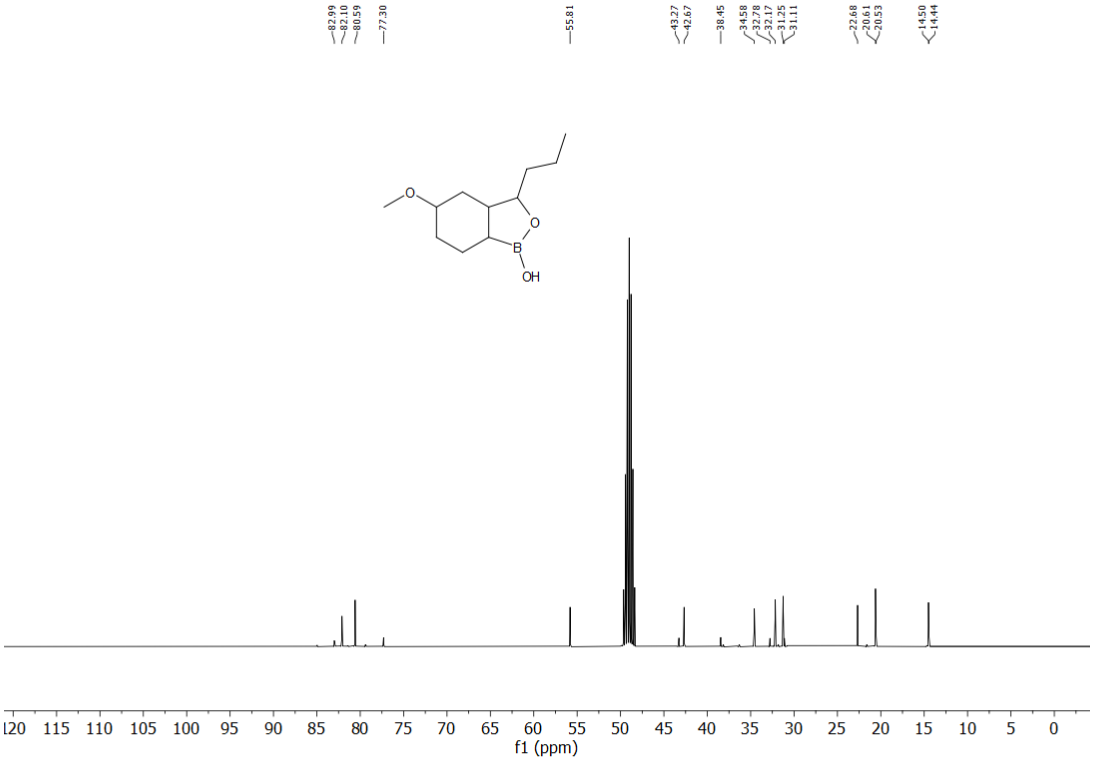

Figure S187: $^{13}C\{^1H\}$ NMR (101 MHz, CD_3OD, 298 K) spectrum of 5-methoxy-3-propylhexahydrobenzo[*c*][1,2]oxaborol-1(3*H*)-ol (6r).

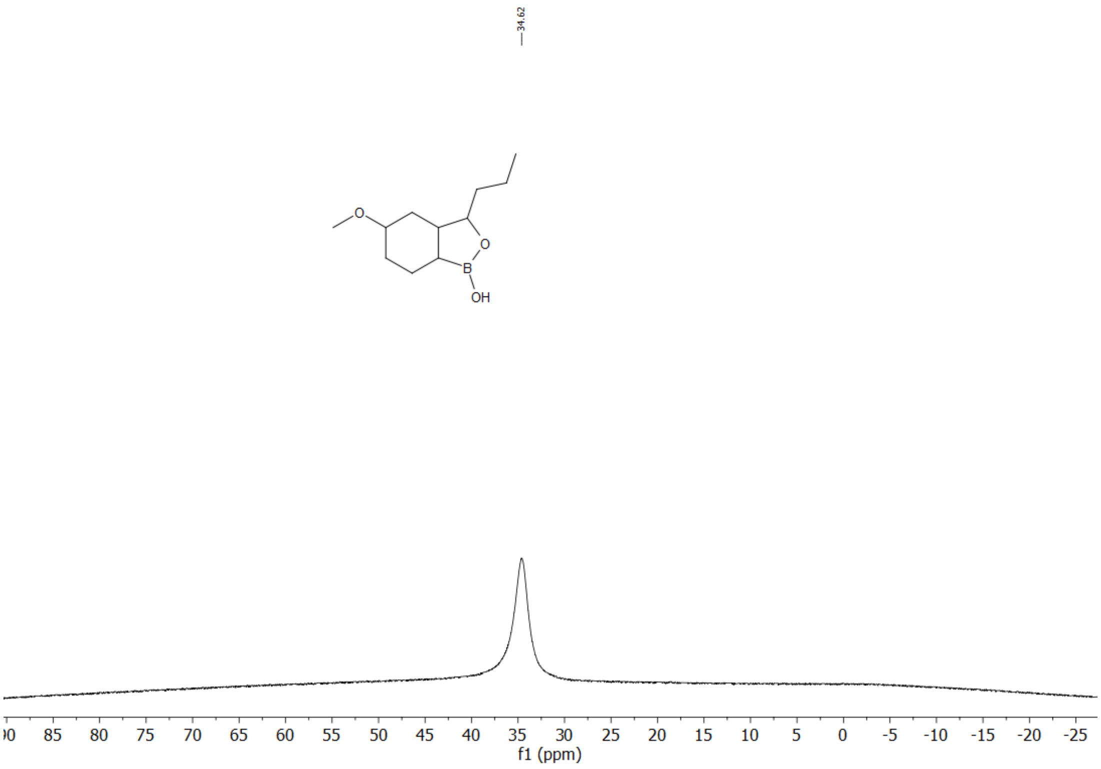

Figure S 188: $^{11}B\{^1H\}$ NMR (128 MHz, CD_3OD, 298 K) spectrum of 5-methoxy-3-propylhexahydrobenzo[*c*][1,2]oxaborol-1(3*H*)-ol (6r).

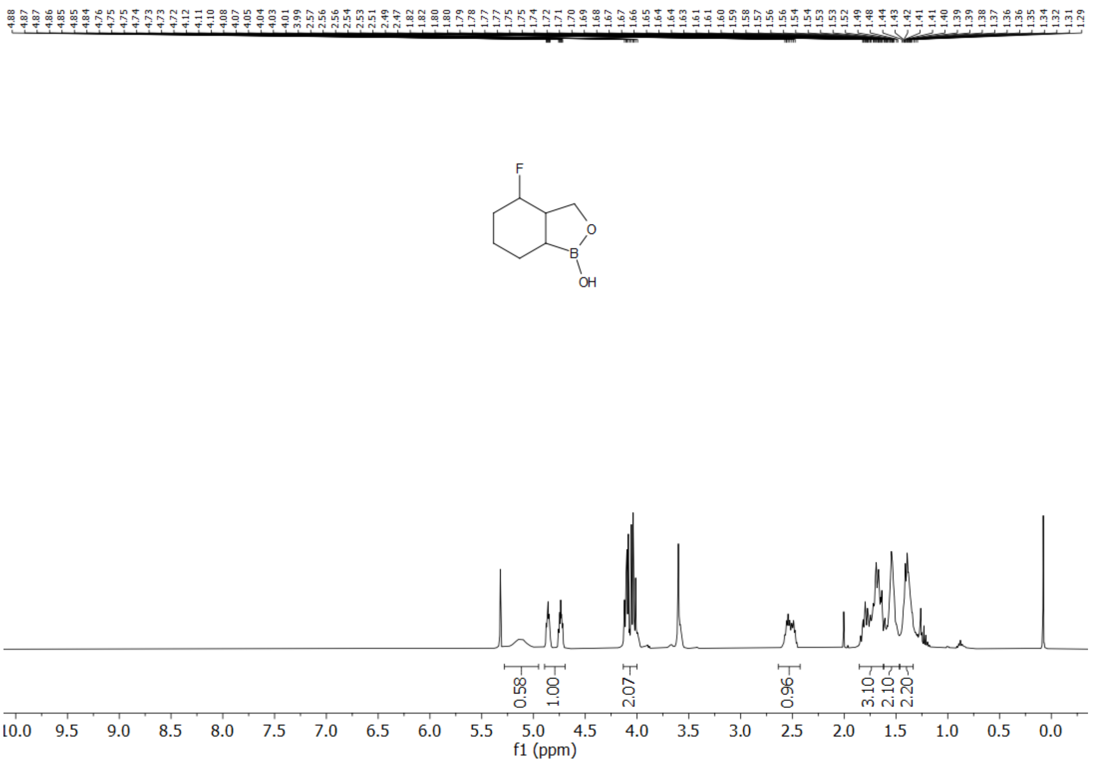

Figure S189: ^{1}H NMR (400 MHz, CD_2Cl_2, 298 K) spectrum of 4-fluorohexahydrobenzo[*c*][1,2]oxaborol-1(3*H*)-ol (6t).

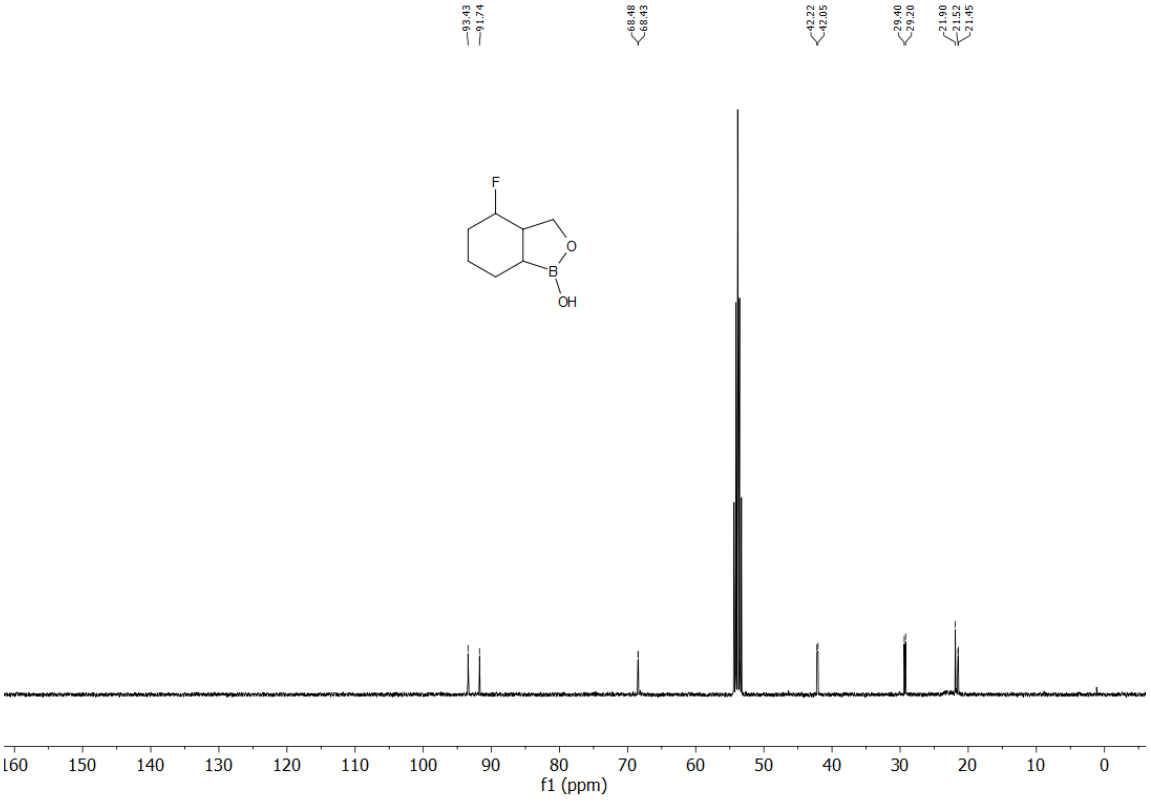

Figure S190: $^{13}C\{^{1}H\}$ NMR (101 MHz, CD_2Cl_2, 298 K) spectrum of 4-fluorohexahydrobenzo[*c*][1,2]oxaborol-1(3*H*)-ol (6t).

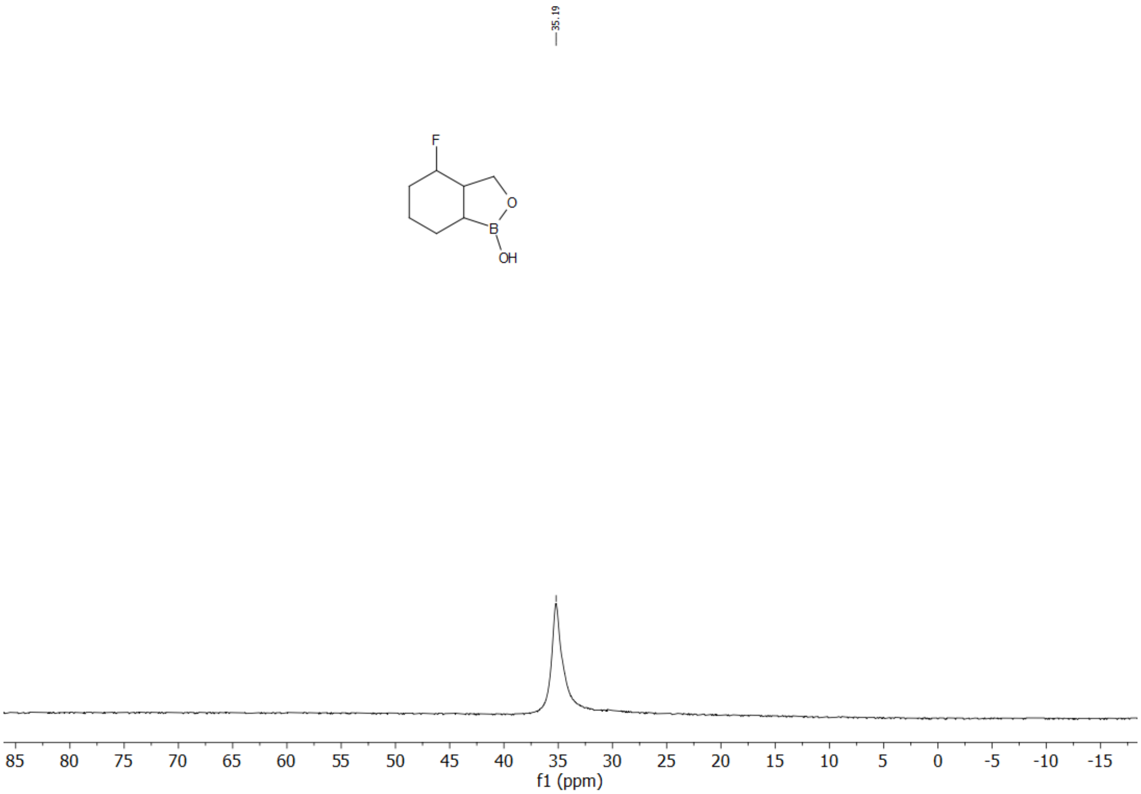

Figure S191: $^{11}B\{^1H\}$ NMR (128 MHz, CD_2Cl_2, 298 K) spectrum of 4-fluorohexahydrobenzo[*c*][1,2]oxaborol-1(3*H*)-ol (6t).

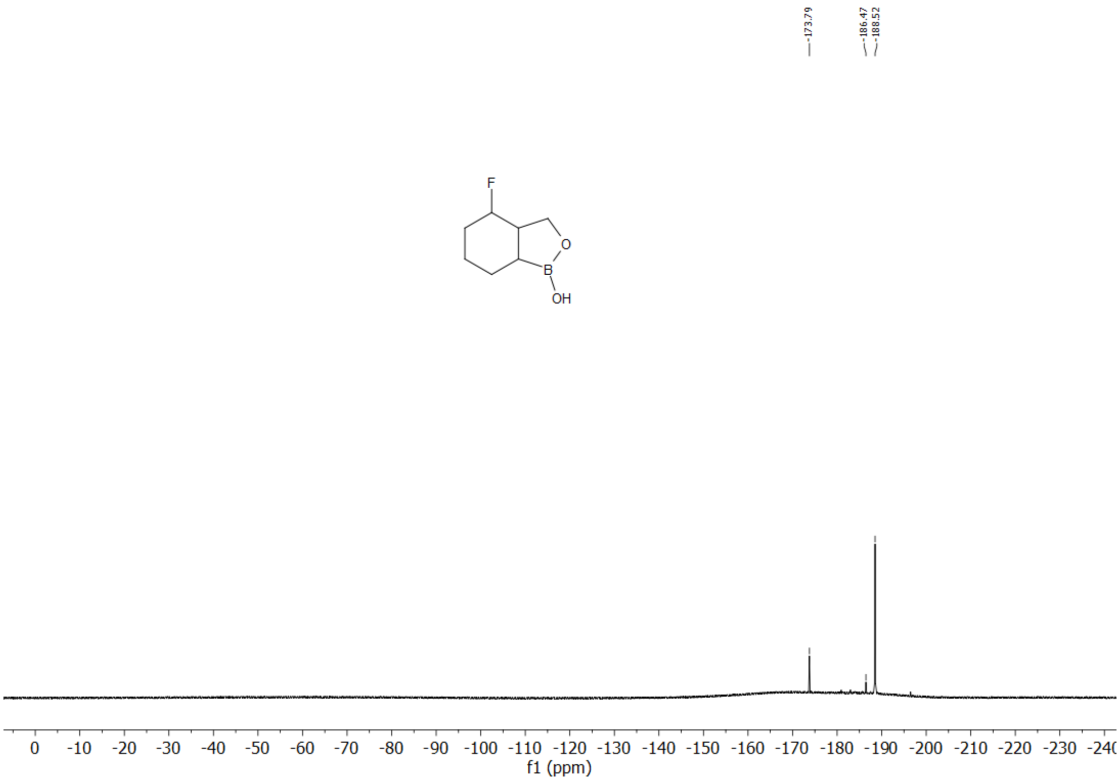

Figure S192: $^{19}F\{^1H\}$ NMR (376 MHz, CD_2Cl_2, 298 K) spectrum of 4-fluorohexahydrobenzo[*c*][1,2]oxaborol-1(3*H*)-ol (6t).

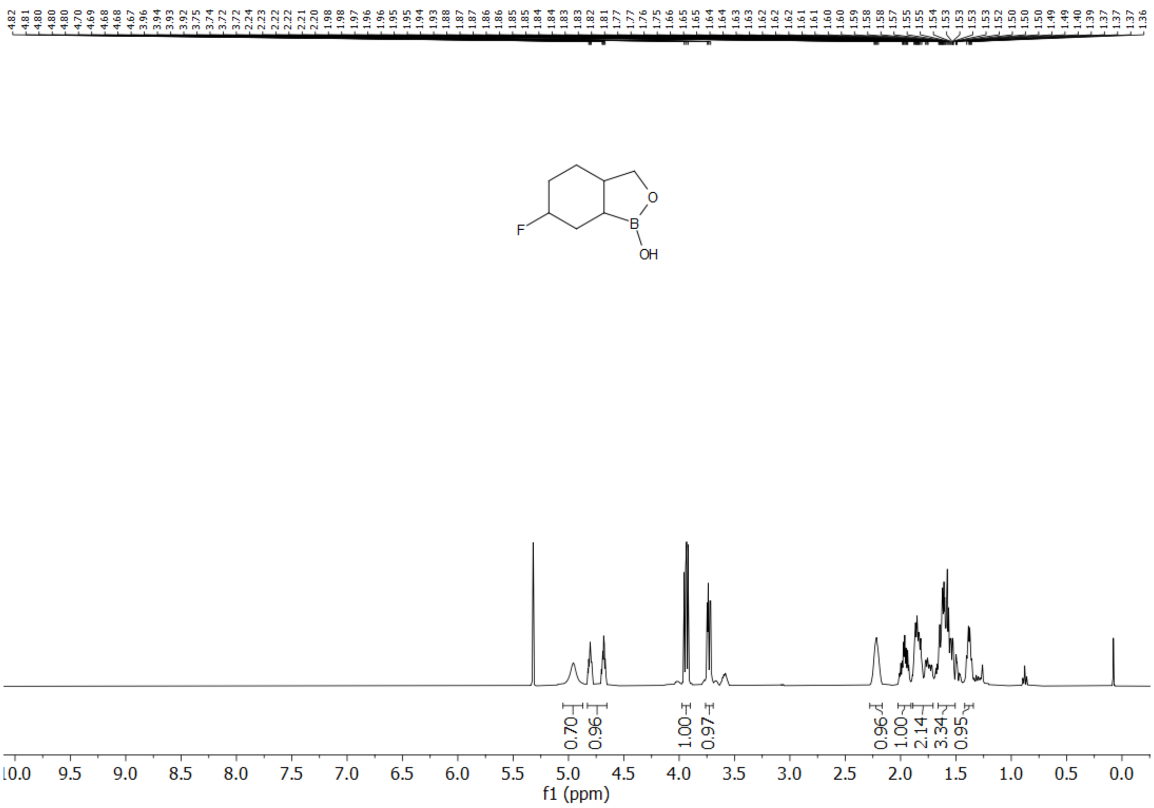

Figure S193: ^{1}H NMR (400 MHz, CD_2Cl_2, 298 K) spectrum of 6-fluorohexahydrobenzo[*c*][1,2]oxaborol-1(3*H*)-ol (6u).

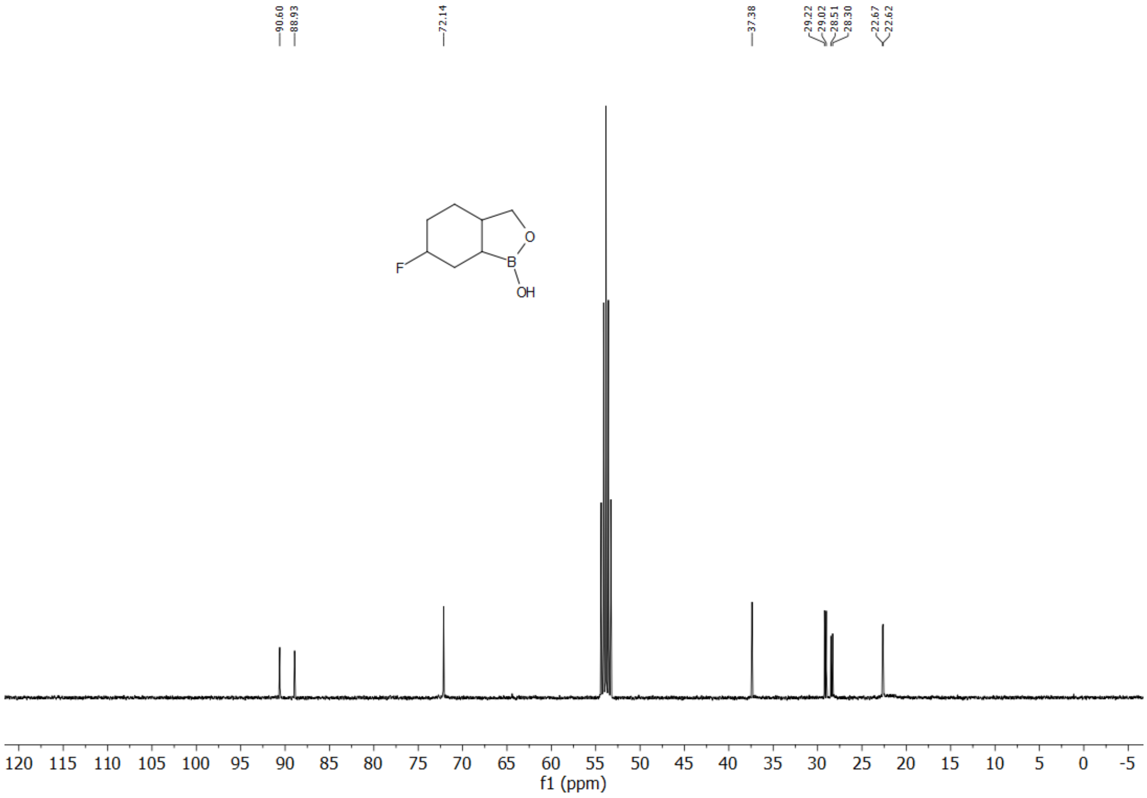

Figure S194: $^{13}C\{^{1}H\}$ NMR (101 MHz, CD_2Cl_2, 298 K) spectrum of 6-fluorohexahydrobenzo[*c*][1,2]oxaborol-1(3*H*)-ol (6u).

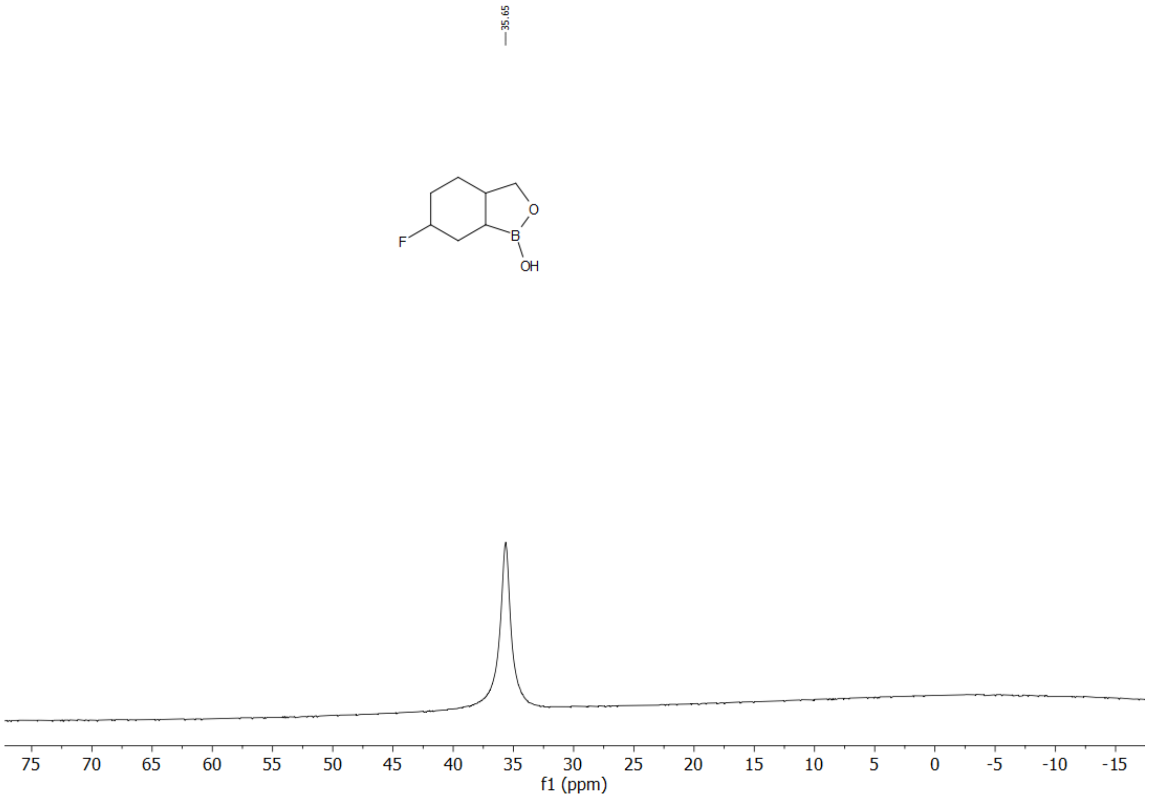

Figure S195: $^{11}B\{^{1}H\}$ NMR (128 MHz, CD_2Cl_2, 298 K) spectrum of 6-fluorohexahydrobenzo[*c*][1,2]oxaborol-1(3*H*)-ol (6u).

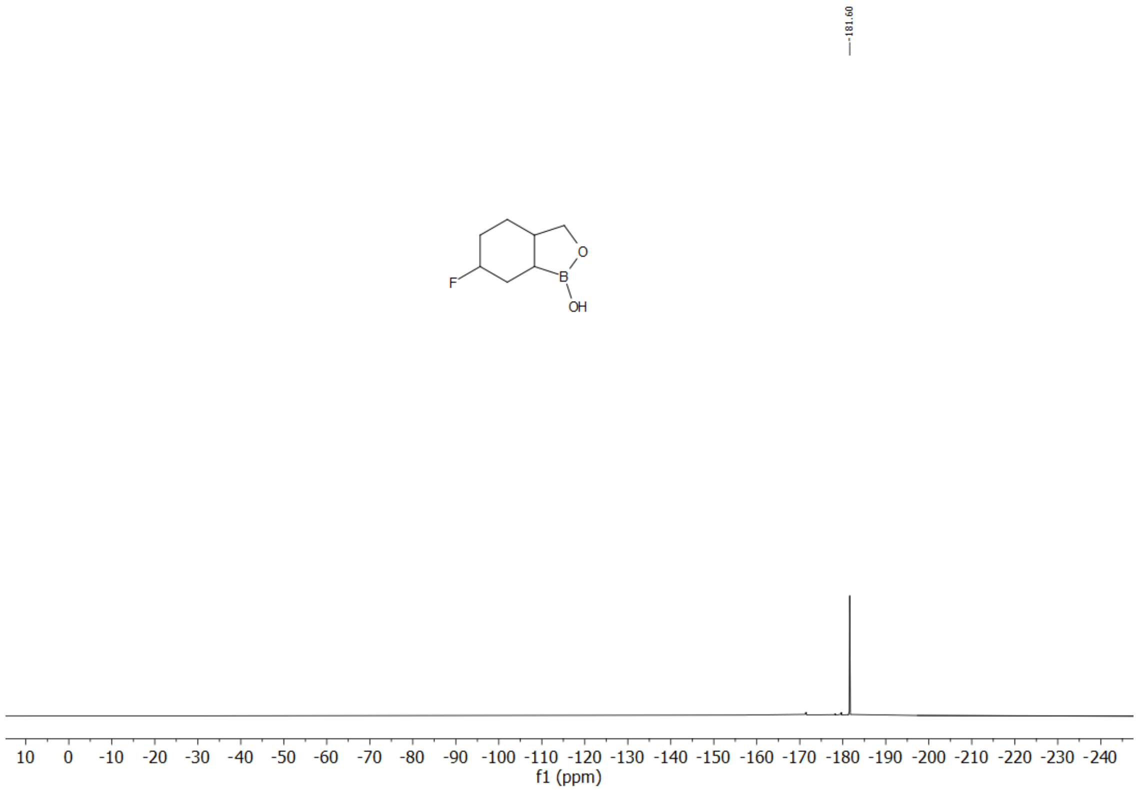

Figure S196: $^{19}F\{^{1}H\}$ NMR (376 MHz, CD_2Cl_2, 298 K) spectrum of 6-fluorohexahydrobenzo[*c*][1,2]oxaborol-1(3*H*)-ol (6u).

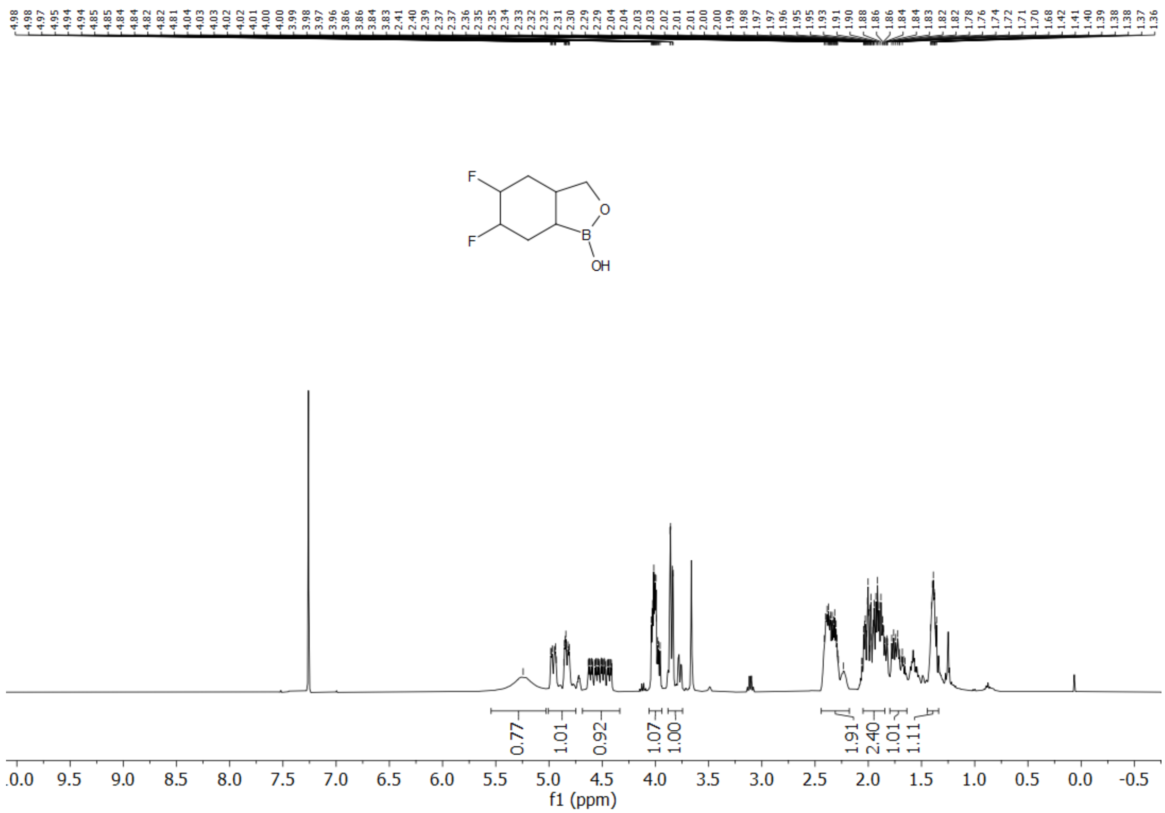

Figure S197: ^{1}H NMR (400 MHz, $CDCl_3$, 298 K) spectrum of 5,6-difluorohexahydrobenzo[*c*][1,2]oxaborol-1(3*H*)-ol (6v).

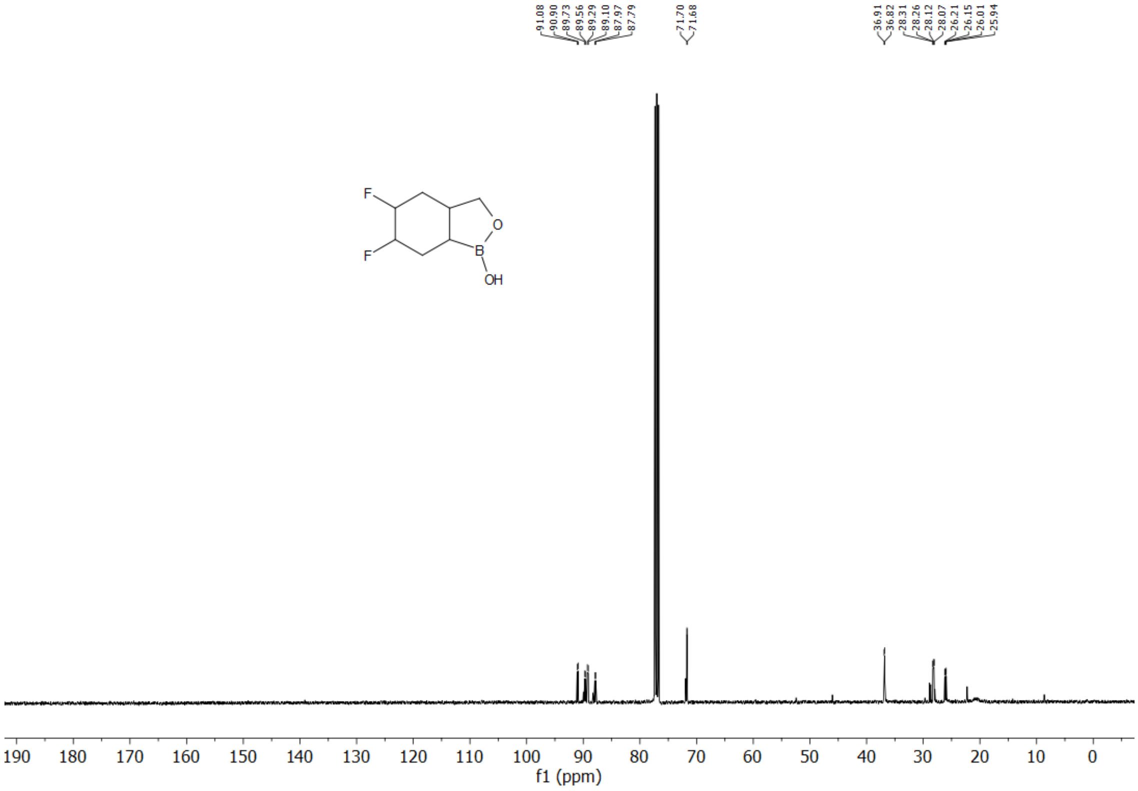

Figure S198: $^{13}C\{^{1}H\}$ NMR (101 MHz, $CDCl_3$, 298 K) spectrum of 5,6-difluorohexahydrobenzo[*c*][1,2]oxaborol-1(3*H*)-ol (6v).

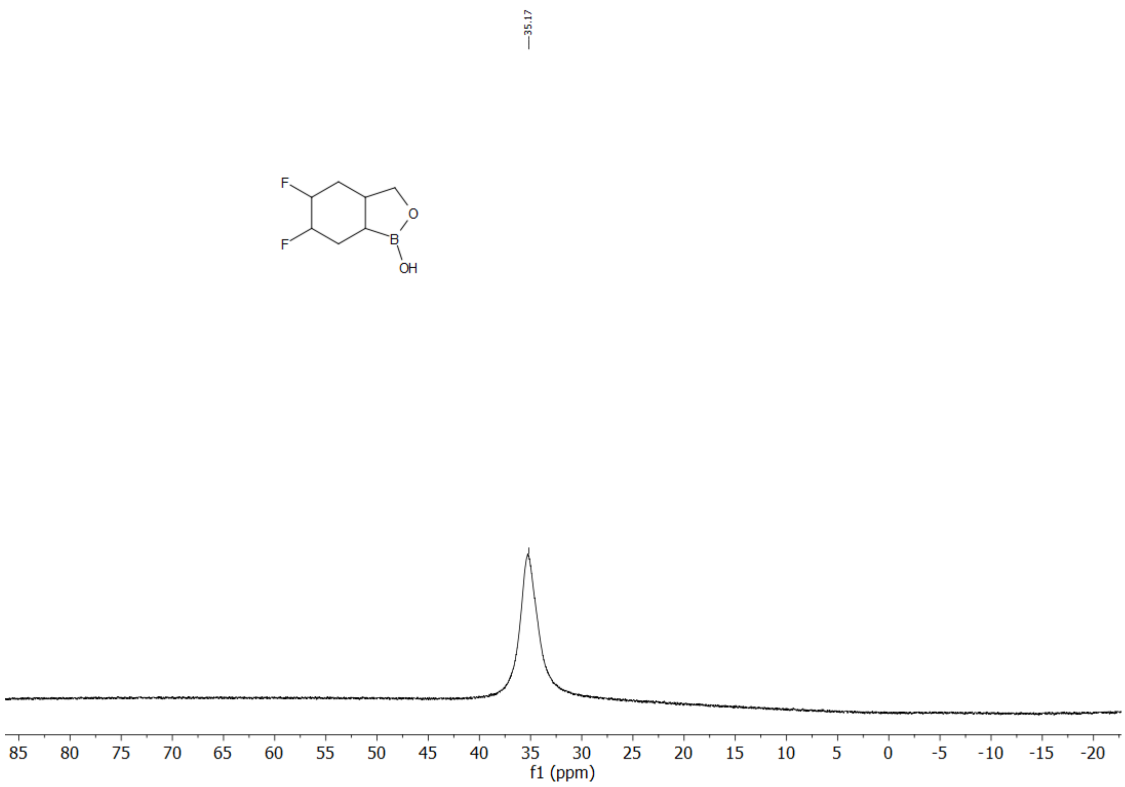

Figure S199: $^{11}B\{^{1}H\}$ NMR (128 MHz, $CDCl_3$, 298 K) spectrum of 5,6-difluorohexahydrobenzo[*c*][1,2]oxaborol-1(3*H*)-ol (6v).

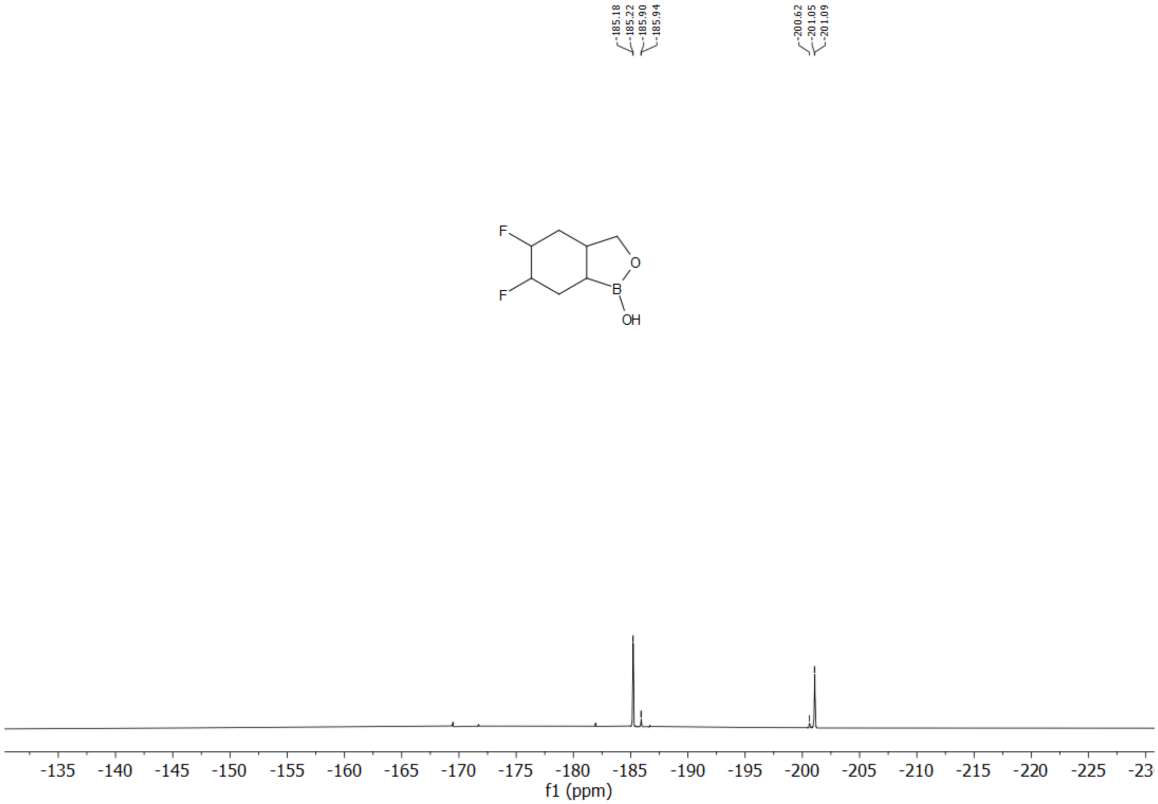

Figure S200: $^{19}F\{^{1}H\}$ NMR (376 MHz, $CDCl_3$, 298 K) spectrum of 5,6-difluorohexahydrobenzo[*c*][1,2]oxaborol-1(3*H*)-ol (6v).

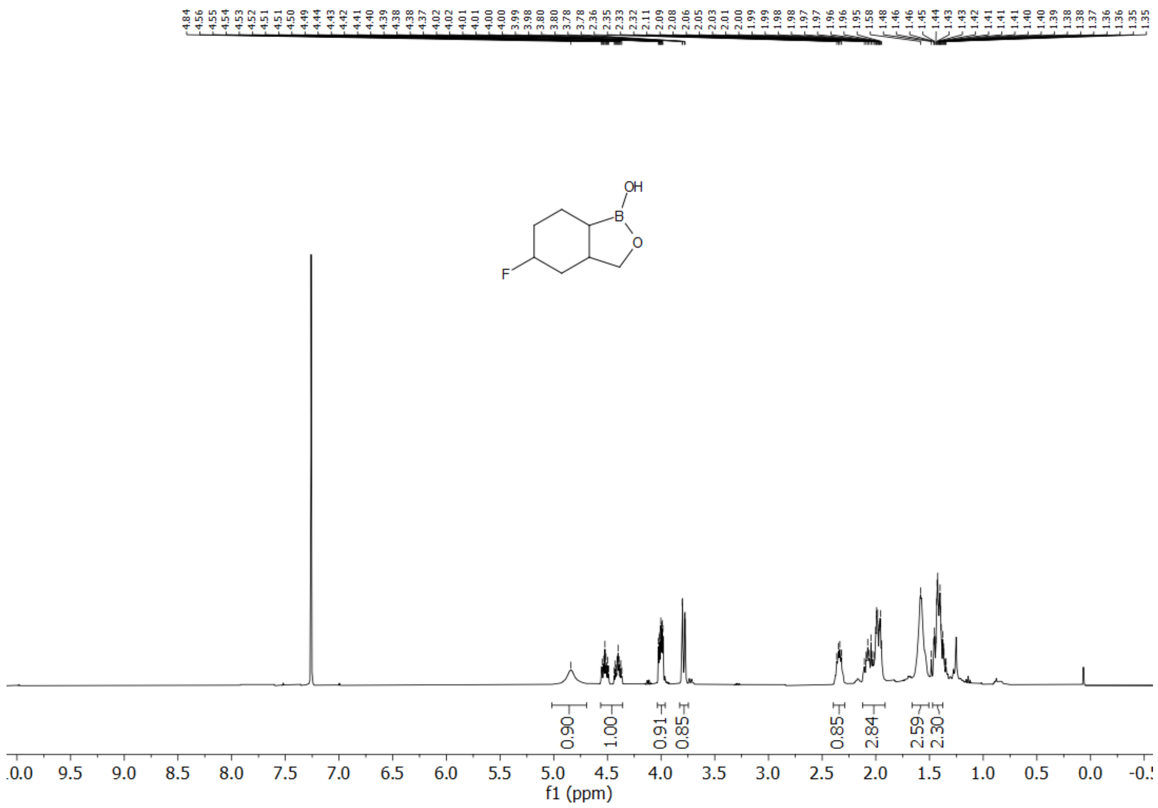

Figure S201: ^{1}H NMR (400 MHz, $CDCl_3$, 298 K) spectrum of 5-fluorohexahydrobenzo[*c*][1,2]oxaborol-1(3*H*)-ol (6w).

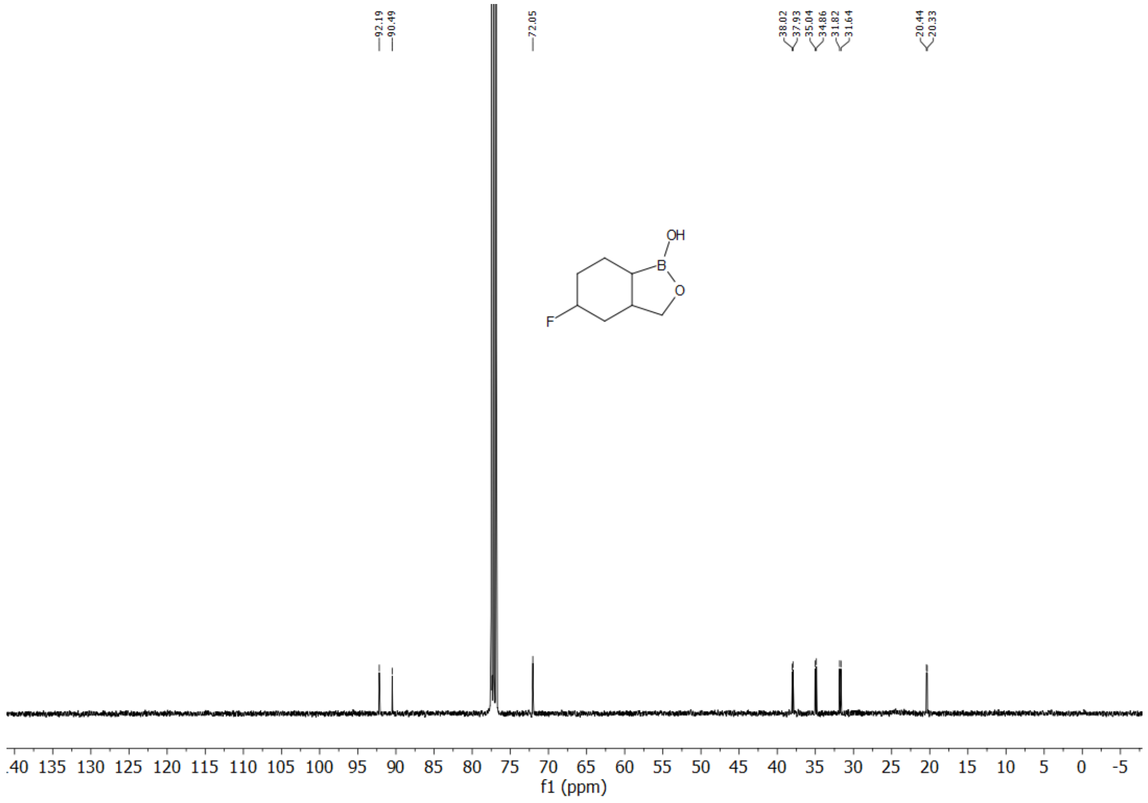

Figure S202: $^{13}C\{^{1}H\}$ NMR (101 MHz, $CDCl_3$, 298 K) spectrum of 5-fluorohexahydrobenzo[*c*][1,2]oxaborol-1(3*H*)-ol (6w).

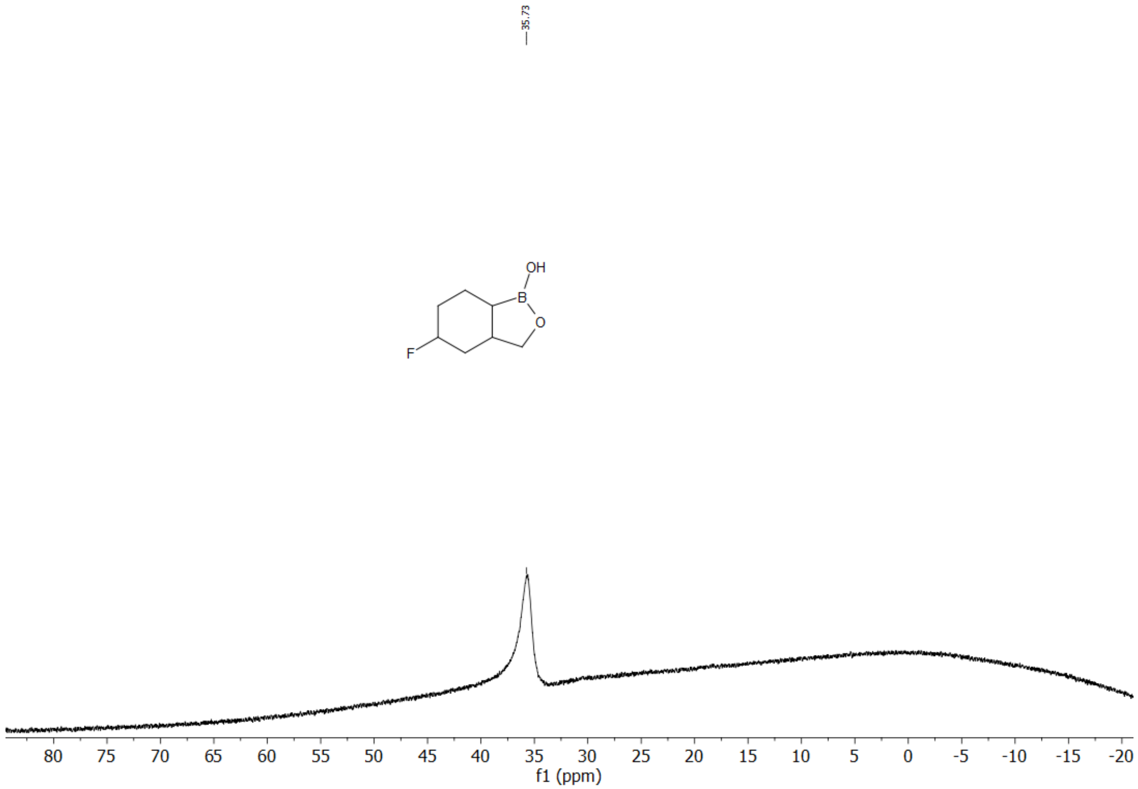

Figure S203: $^{11}B\{^{1}H\}$ NMR (128 MHz, $CDCl_3$, 298 K) spectrum of 5-fluorohexahydrobenzo[*c*][1,2]oxaborol-1(3*H*)-ol (6w).

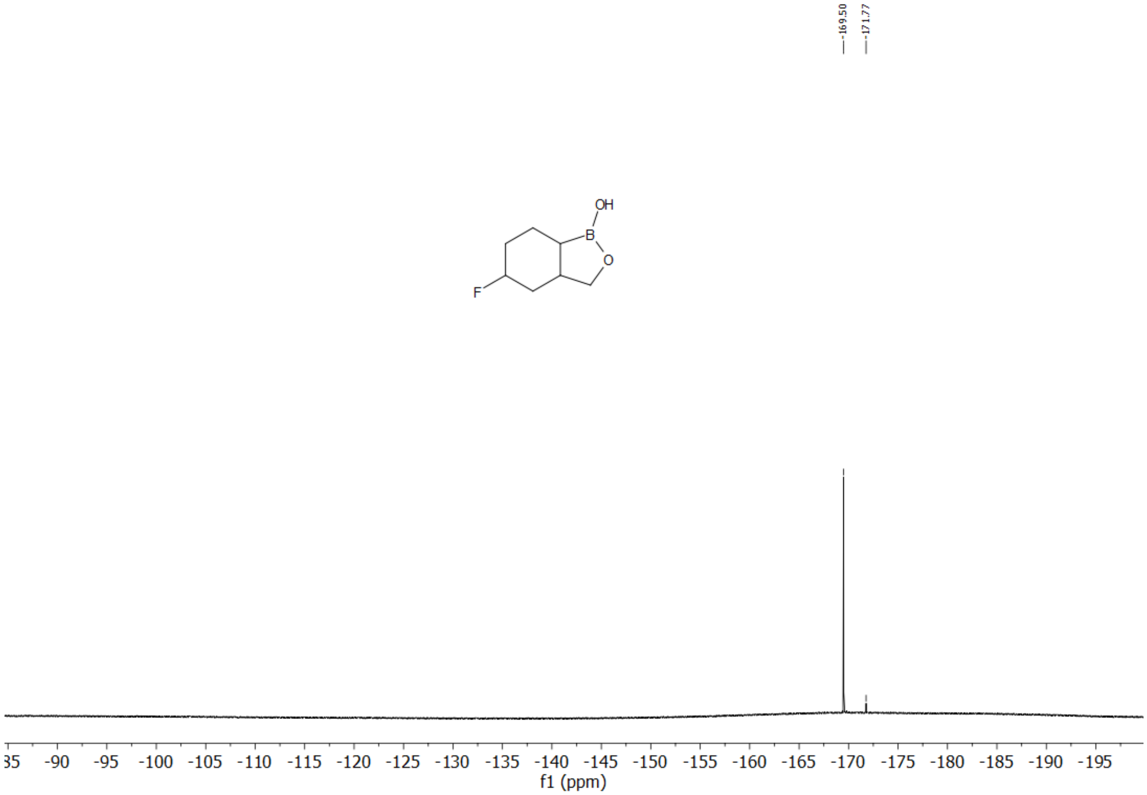

Figure S204: $^{19}F\{^{1}H\}$ NMR (376 MHz, $CDCl_3$, 298 K) spectrum of 5-fluorohexahydrobenzo[*c*][1,2]oxaborol-1(3*H*)-ol (6w).

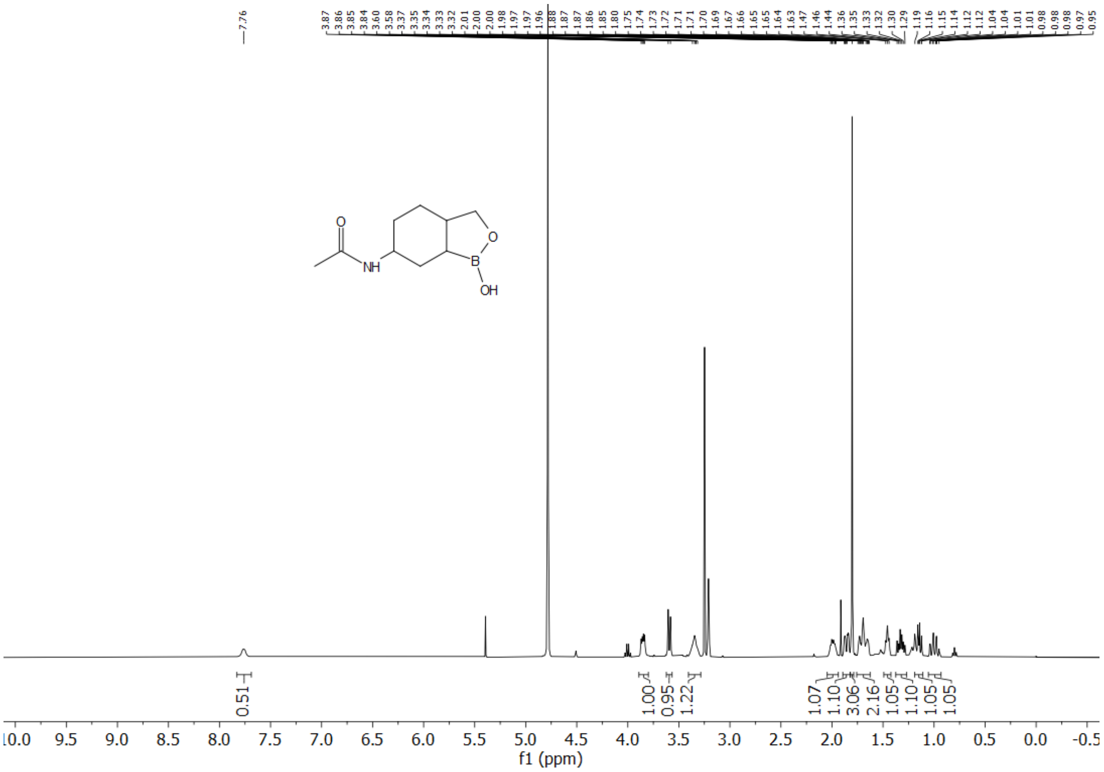

Figure S205: ^{1}H NMR (400 MHz, CD_3OD, 298 K) spectrum of *N*-(1-hydroxyoctahydrobenzo[*c*][1,2]oxaborol-6-yl)acetamide (6x).

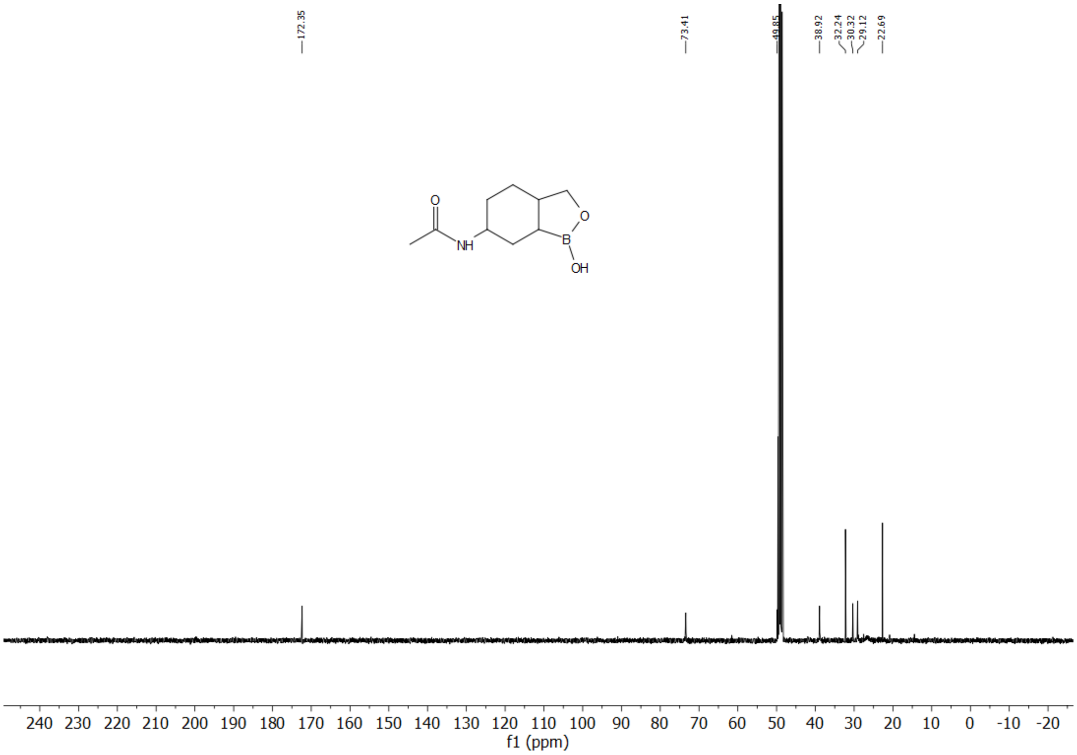

Figure S206: $^{13}C\{^{1}H\}$ NMR (101 MHz, CD_3OD, 298 K) spectrum of *N*-(1-hydroxyoctahydrobenzo[*c*][1,2]oxaborol-6-yl)acetamide (6x).

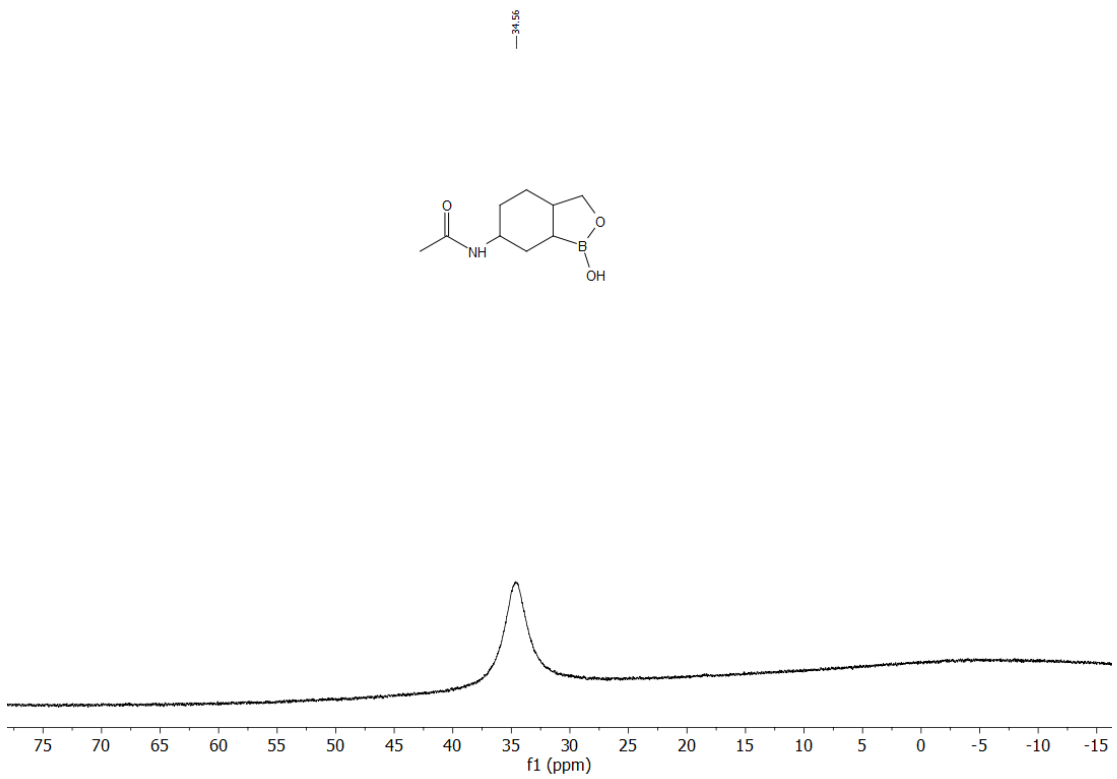

Figure S207: $^{11}B\{^{1}H\}$ NMR (128 MHz, CD_3OD, 298 K) spectrum of *N*-(1-hydroxyoctahydrobenzo[*c*][1,2]oxaborol-6-yl)acetamide (6x).

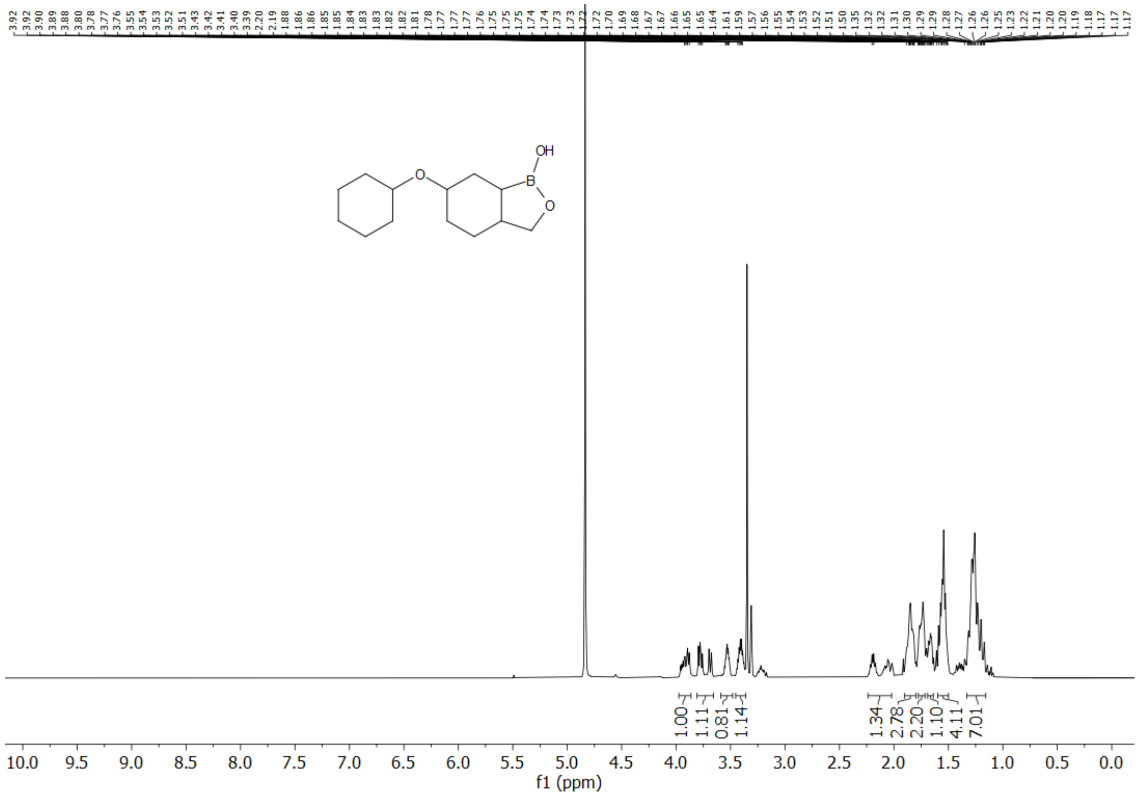

Figure S208: ^{1}H NMR (400 MHz, CD_3OD, 298 K) spectrum of 6-(cyclohexyloxy)hexahydrobenzo[*c*][1,2]oxaborol-1(3*H*)-ol (6y).

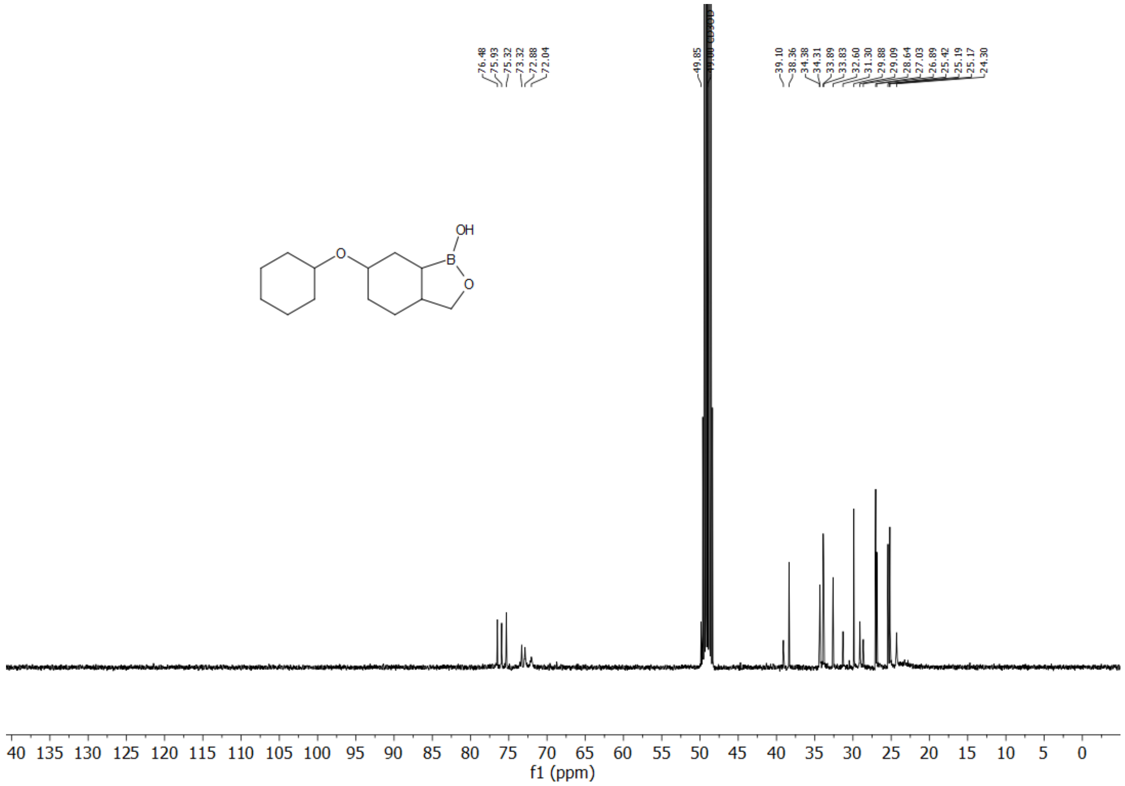

Figure S209: **$^{13}C\{^{1}H\}$ NMR (101 MHz, CD_3OD, 298 K) spectrum of 6-(cyclohexyloxy)hexahydrobenzo[*c*][1,2]oxaborol-1(3*H*)-ol (6y).**

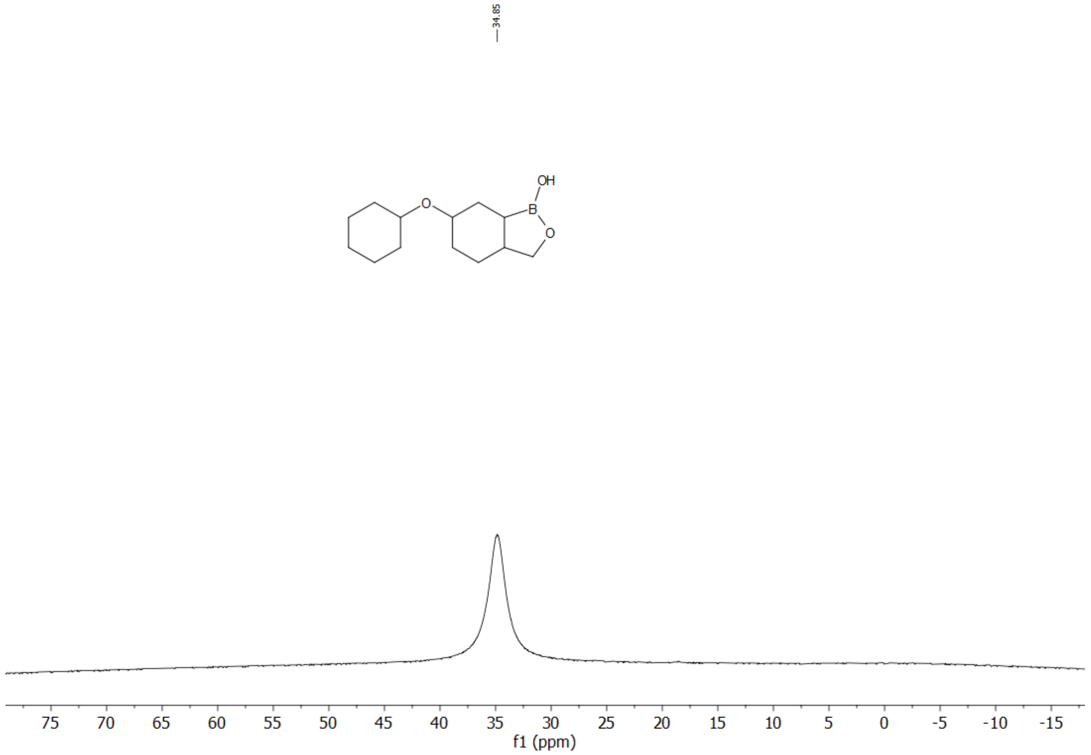

Figure S210: **$^{11}B\{^{1}H\}$ NMR (128 MHz, CD_3OD, 298 K) spectrum of 6-(cyclohexyloxy)hexahydrobenzo[*c*][1,2]oxaborol-1(3*H*)-ol (6y).**

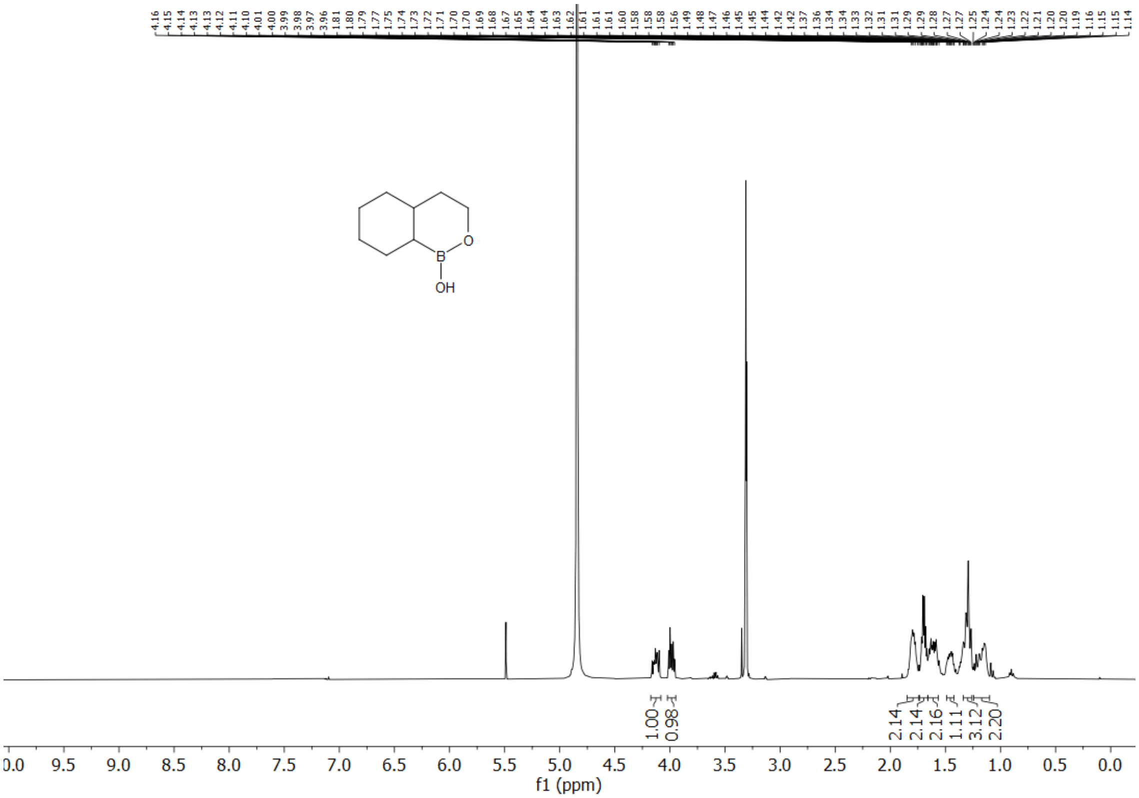

Figure S211: ^{1}H NMR (400 MHz, CD_3OD, 298 K) spectrum of Octahydro-1*H*-benzo[*c*][1,2]oxaborinin-1-ol (8a).

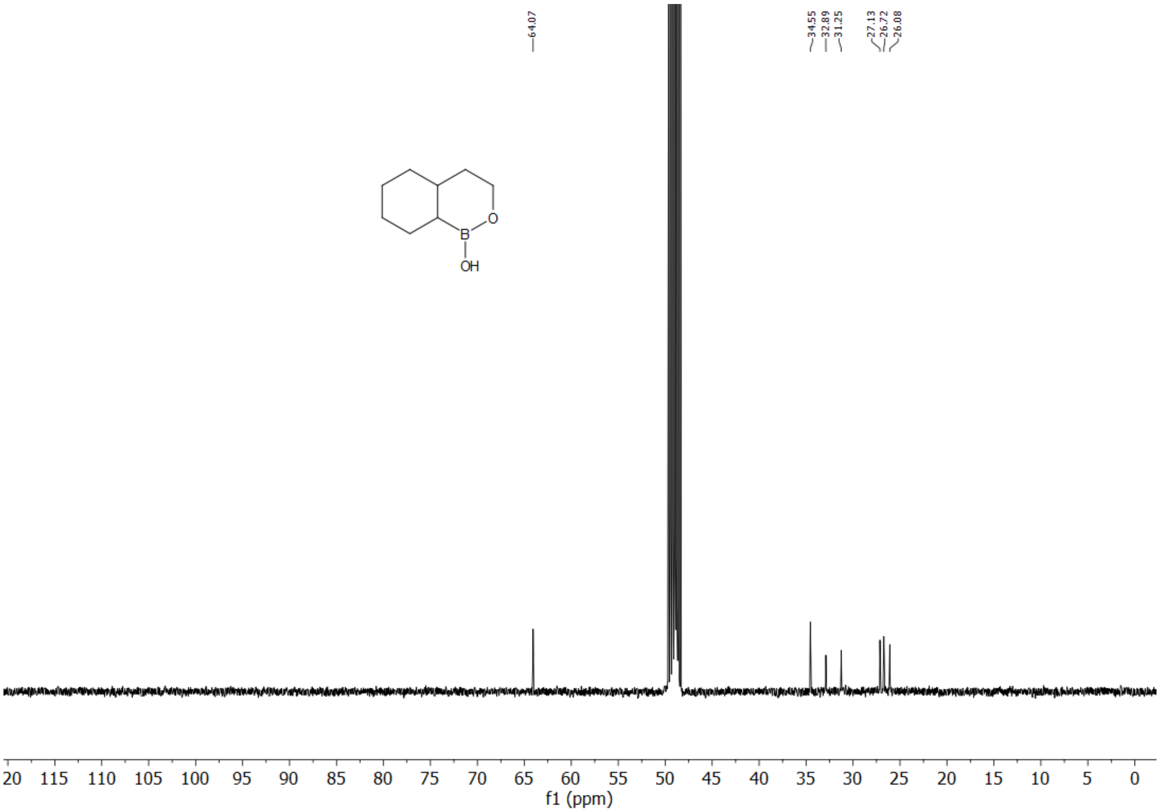

Figure S212: $^{13}C\{^{1}H\}$ NMR (101 MHz, CD_3OD, 298 K) spectrum of Octahydro-1*H*-benzo[*c*][1,2]oxaborinin-1-ol (8a).

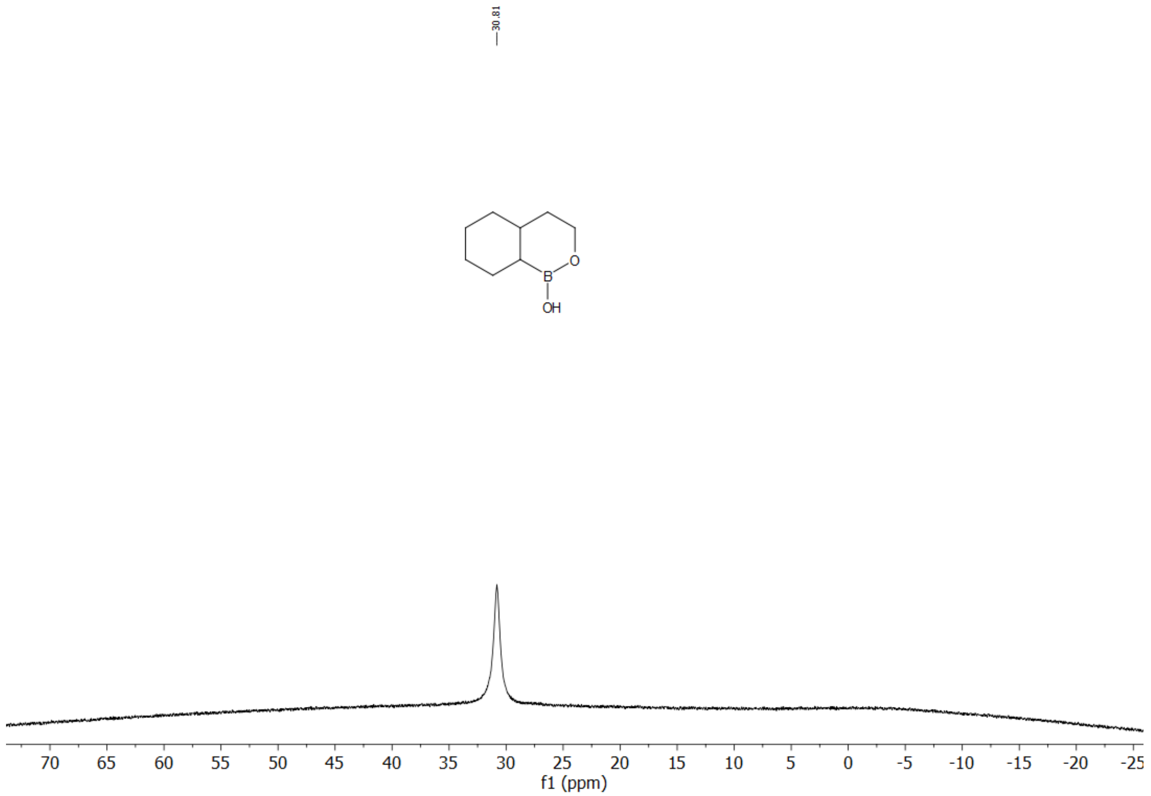

Figure S213: $^{11}B\{^{1}H\}$ NMR (128 MHz, CD_3OD, 298 K) spectrum of Octahydro-1*H*-benzo[*c*][1,2]oxaborinin-1-ol (8a).

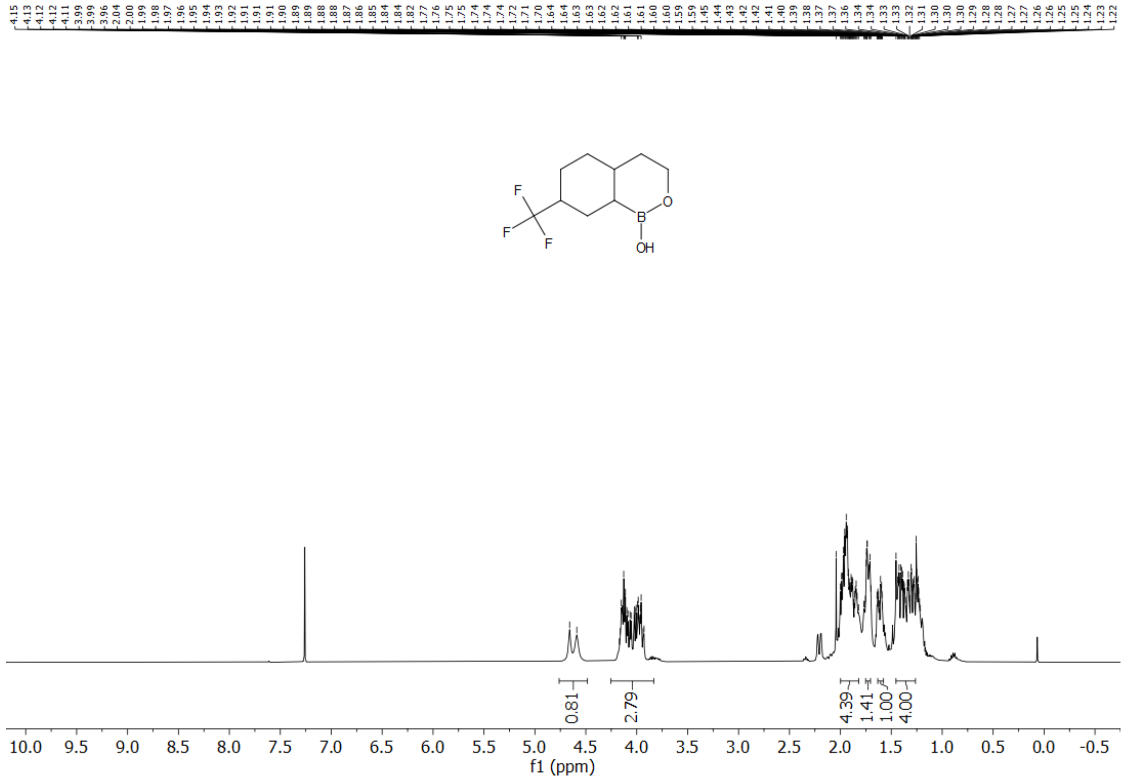

Figure S214: ^{1}H NMR (400 MHz, $CDCl_3$, 298 K) spectrum of 7-(trifluoromethyl)octahydro-1*H*-benzo[*c*][1,2]oxaborinin-1-ol (8b).

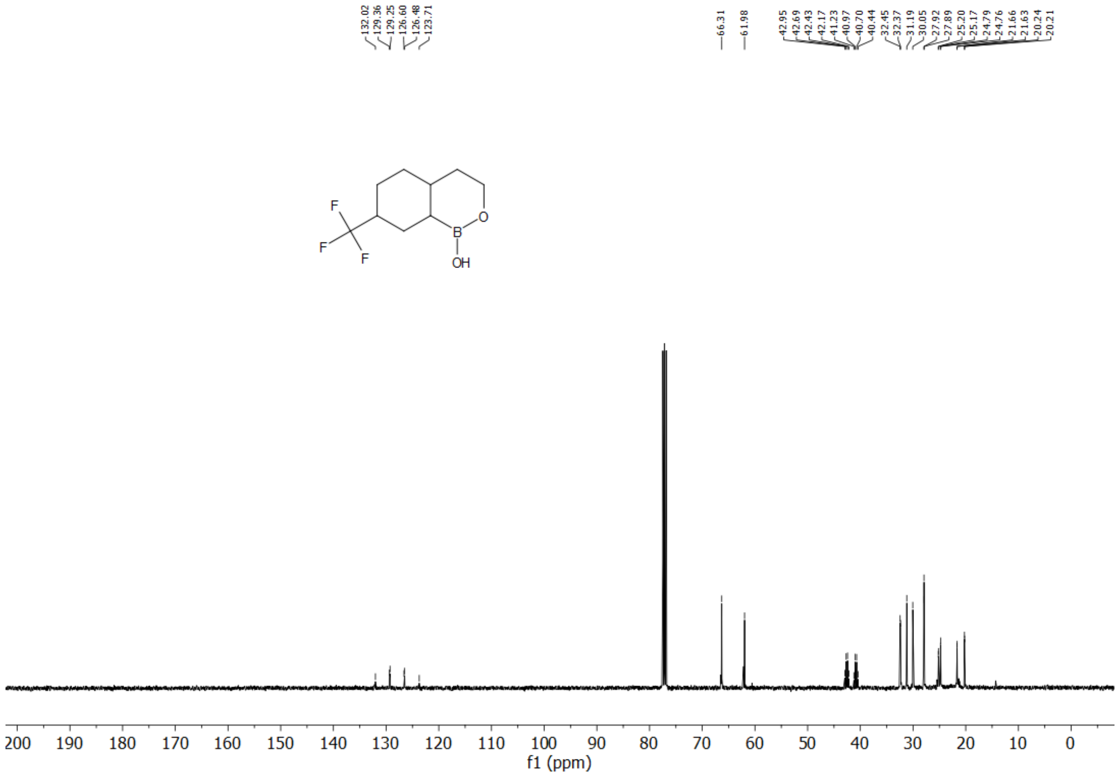

Figure S215: $^{13}C\{^{1}H\}$ NMR (101 MHz, $CDCl_3$, 298 K) spectrum of 7-(trifluoromethyl)octahydro-1*H*-benzo[*c*][1,2]oxaborinin-1-ol (8b).

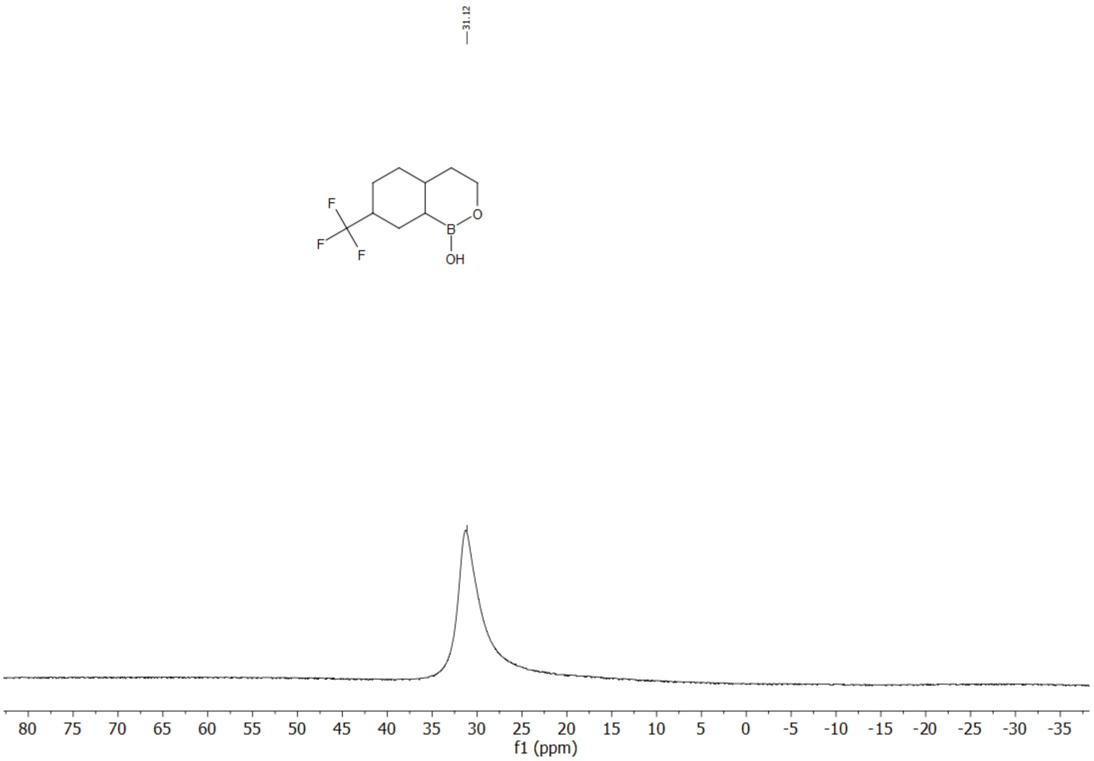

Figure S216: $^{11}B\{^{1}H\}$ NMR (128 MHz, $CDCl_3$, 298 K) spectrum of 7-(trifluoromethyl)octahydro-1*H*-benzo[*c*][1,2]oxaborinin-1-ol (8b).

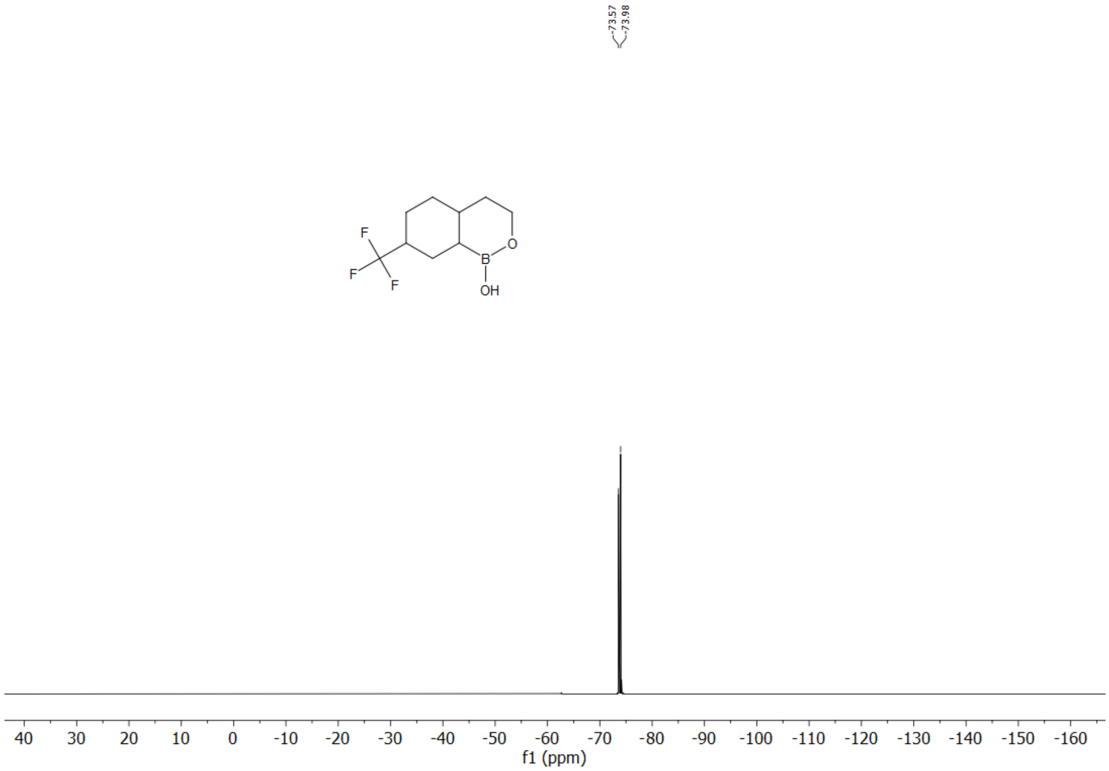

Figure S217: $^{19}F\{^{1}H\}$ NMR (376 MHz, $CDCl_3$, 298 K) spectrum of of 7-(trifluoromethyl)octahydro-1*H*-benzo[*c*][1,2]oxaborinin-1-ol (8b).

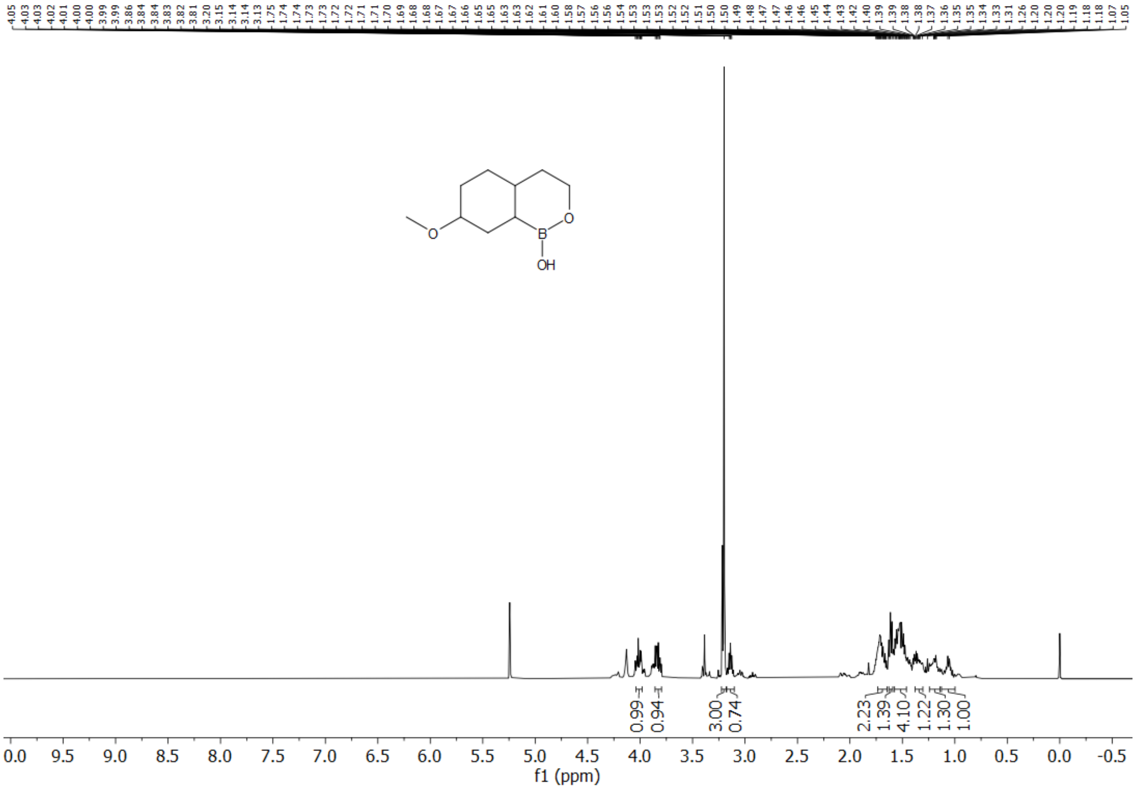

Figure S218: ^{1}H NMR (400 MHz, CD_2Cl_2, 298 K) spectrum of 7-methoxyoctahydro-1*H*-benzo[*c*][1,2]oxaborinin-1-ol (8c).

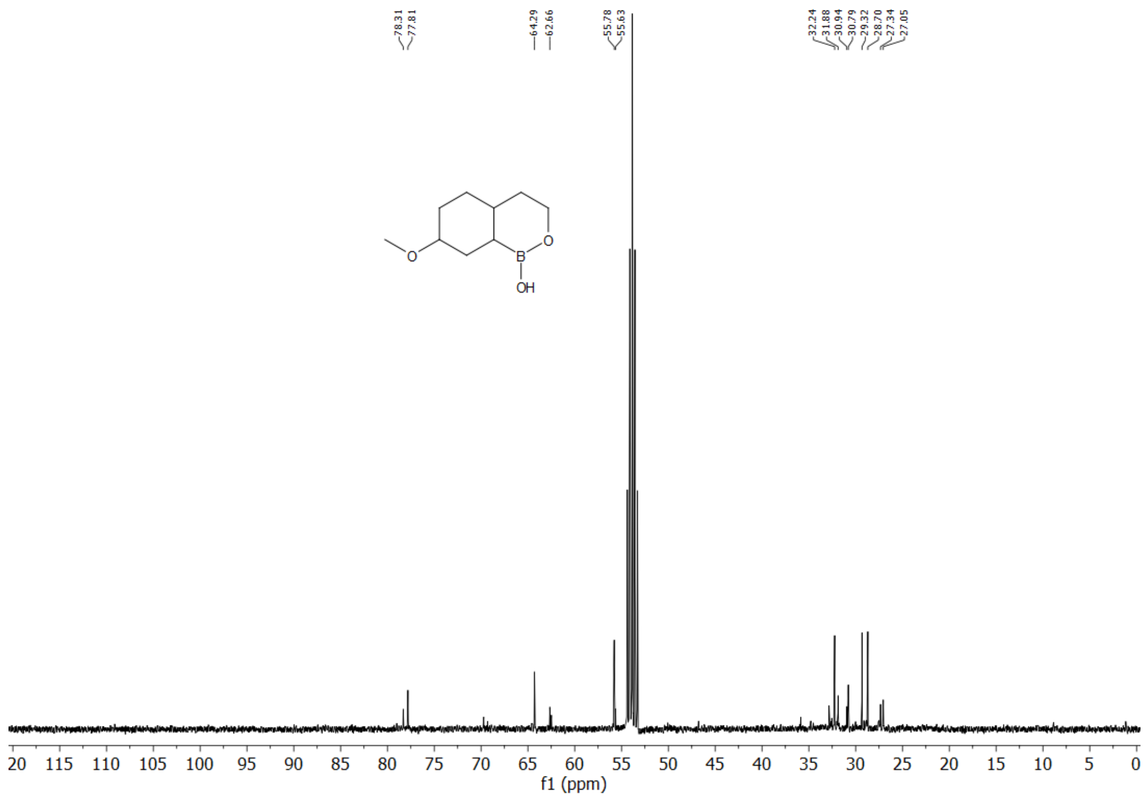

Figure S219: $^{13}C\{^1H\}$ NMR (101 MHz, CD_2Cl_2, 298 K) spectrum of 7-methoxyoctahydro-1*H*-benzo[*c*][1,2]oxaborinin-1-ol (8c).

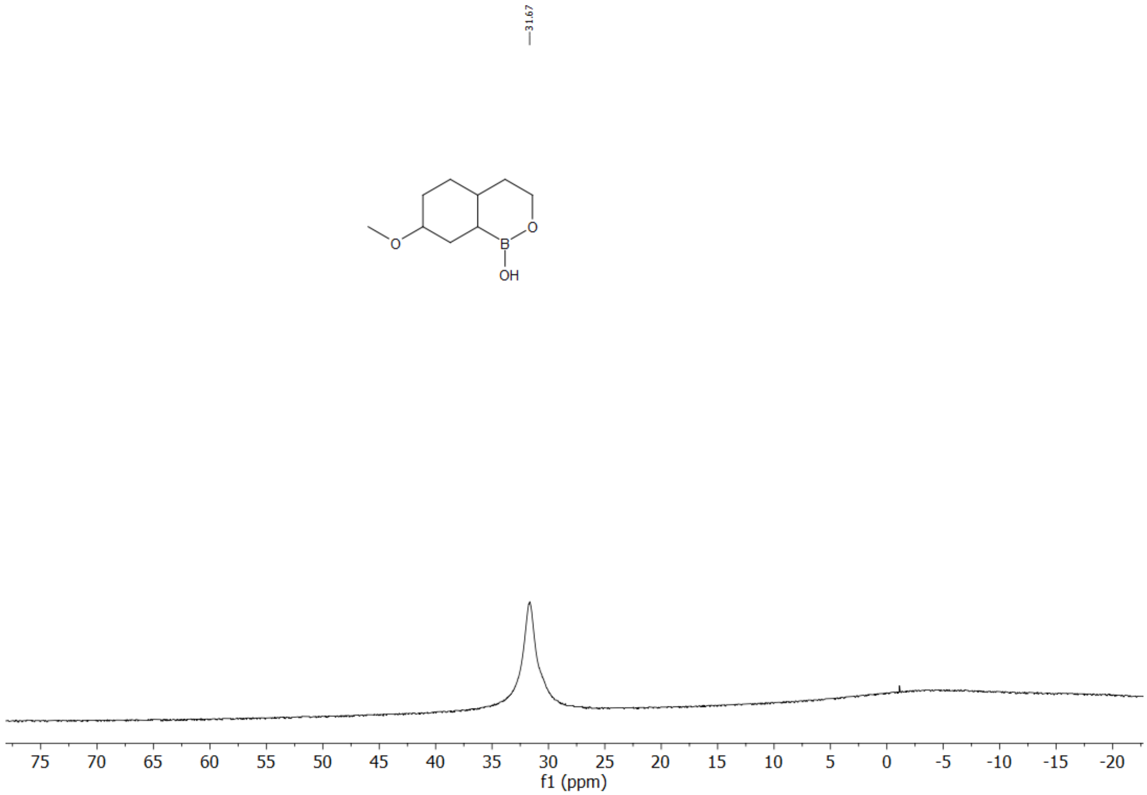

Figure S220: $^{11}B\{^1H\}$ NMR (128 MHz, CD_2Cl_2, 298 K) spectrum of 7-methoxyoctahydro-1*H*-benzo[*c*][1,2]oxaborinin-1-ol (8c).

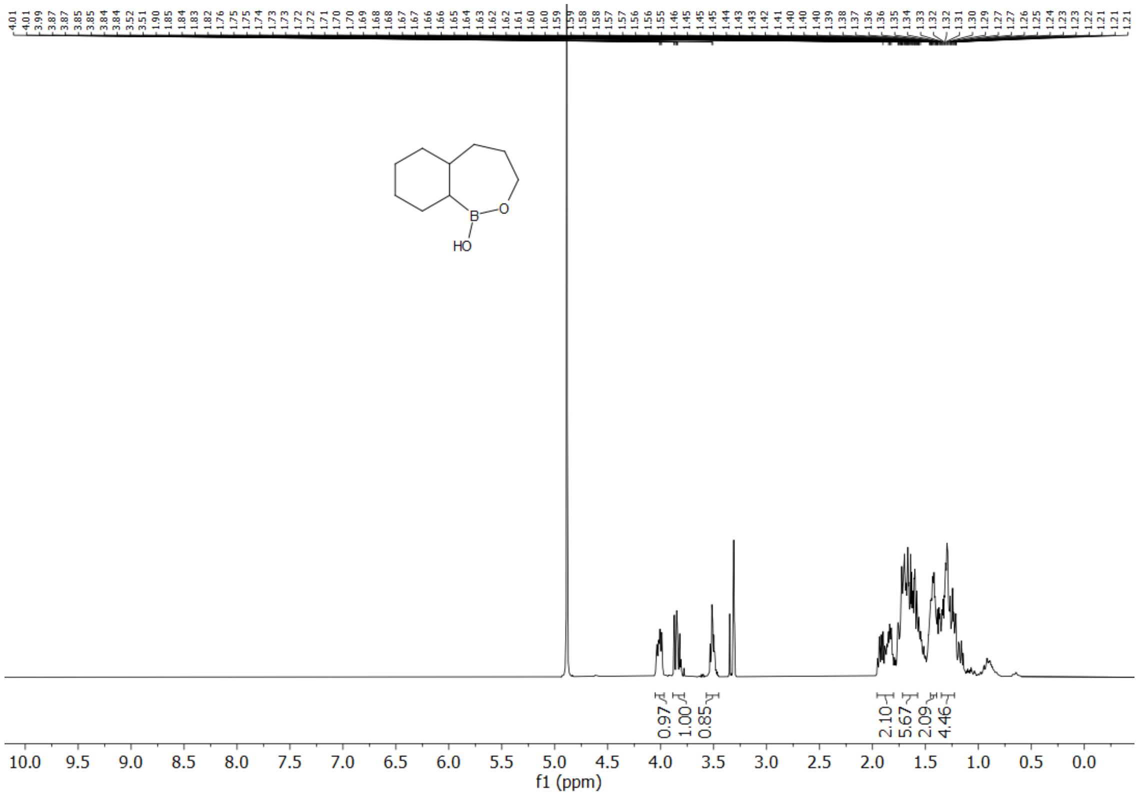

Figure S221: ^{1}H NMR (400 MHz, CD_3OD, 298 K) spectrum of Octahydrobenzo[*c*][1,2]oxaborepin-1(3*H*)-ol (8d).

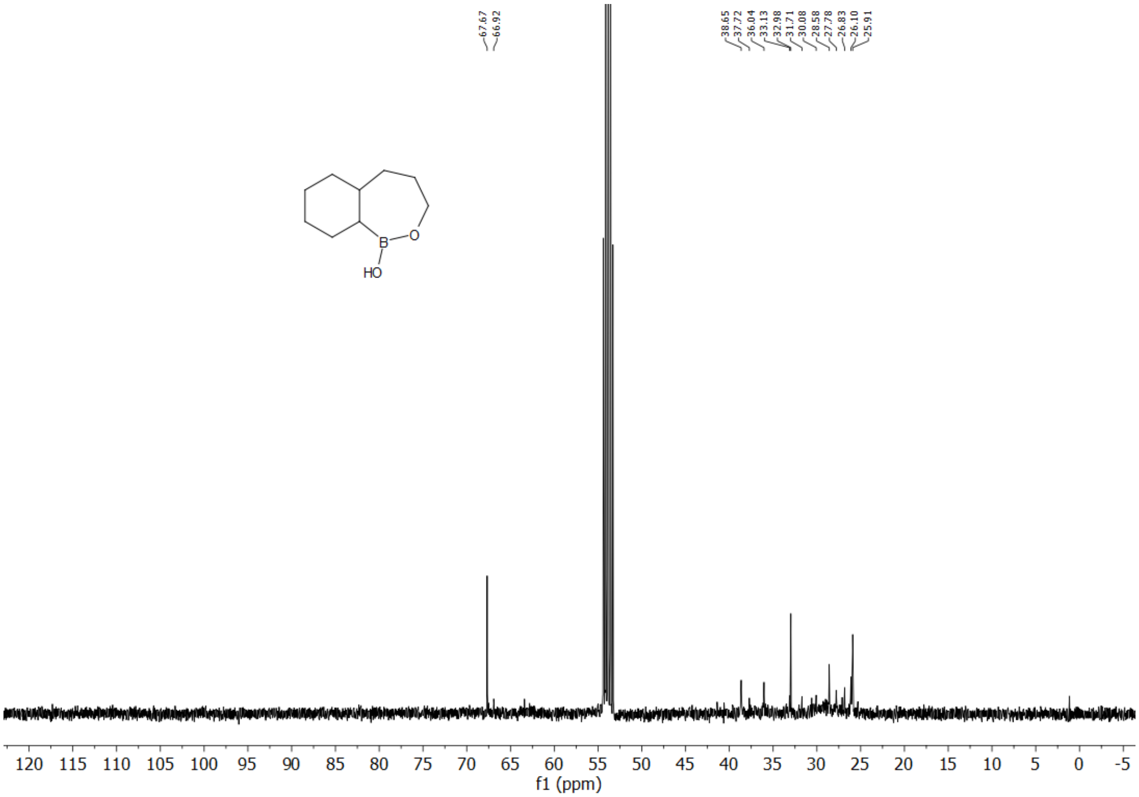

Figure S222: $^{13}C\{^{1}H\}$ NMR (101 MHz, CD_2Cl_2, 298 K) spectrum of Octahydrobenzo[*c*][1,2]oxaborepin-1(3*H*)-ol (8d).

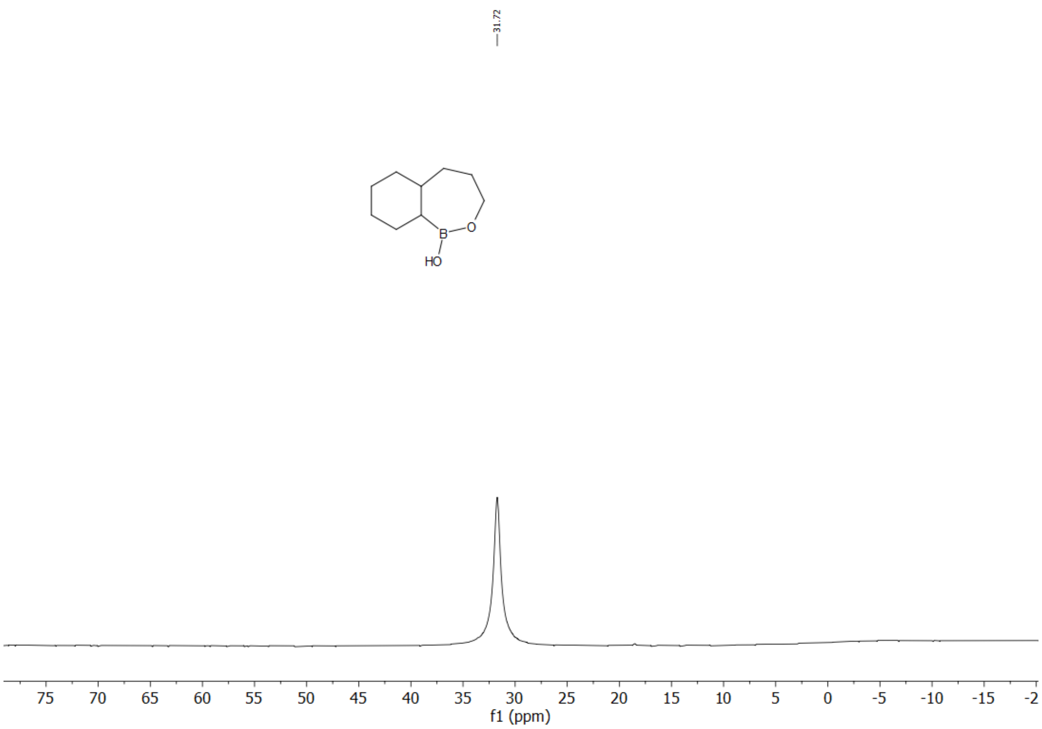

Figure S223: $^{11}B\{^1H\}$ NMR (128 MHz, CD_3OD, 298 K) spectrum of Octahydrobenzo[*c*][1,2]oxaborepin-1(3*H*)-ol (8d).

31.Representative NMR spectra for the determination of diastereomeric ratio

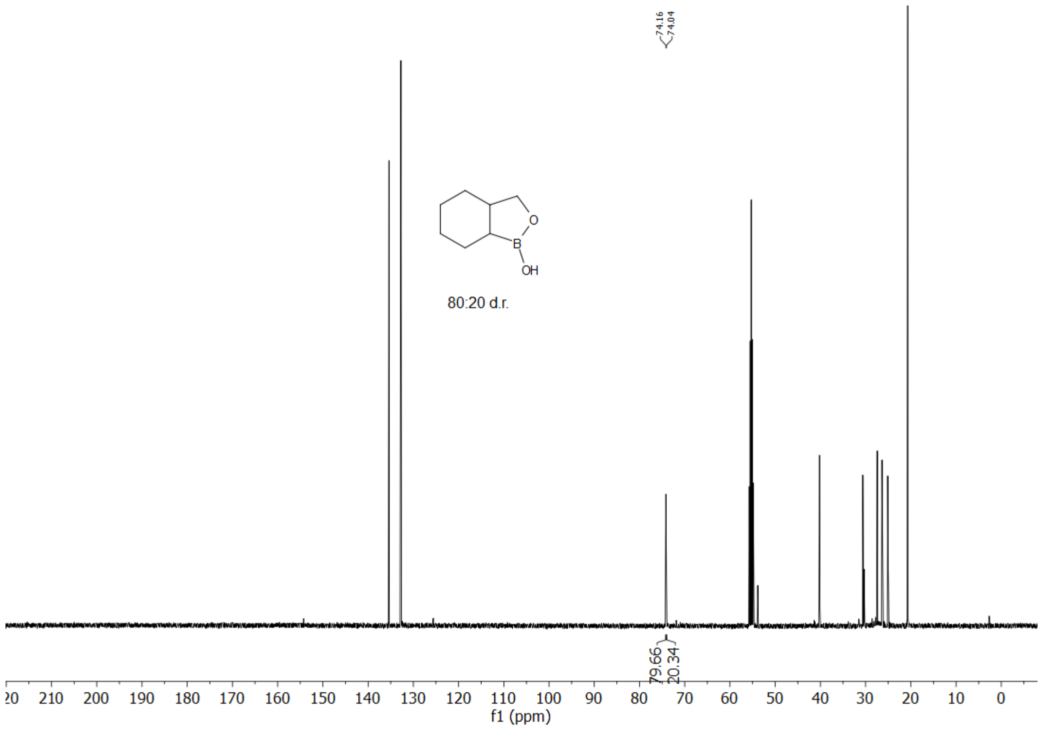

Figure S224: Quantitative $^{13}C\{^1H\}$ NMR (126 MHz, CD_2Cl_2, 298 K) reaction mixture spectrum of Hexahydrobenzo[*c*][1,2]oxaborol-1(3*H*)-ol (6a).

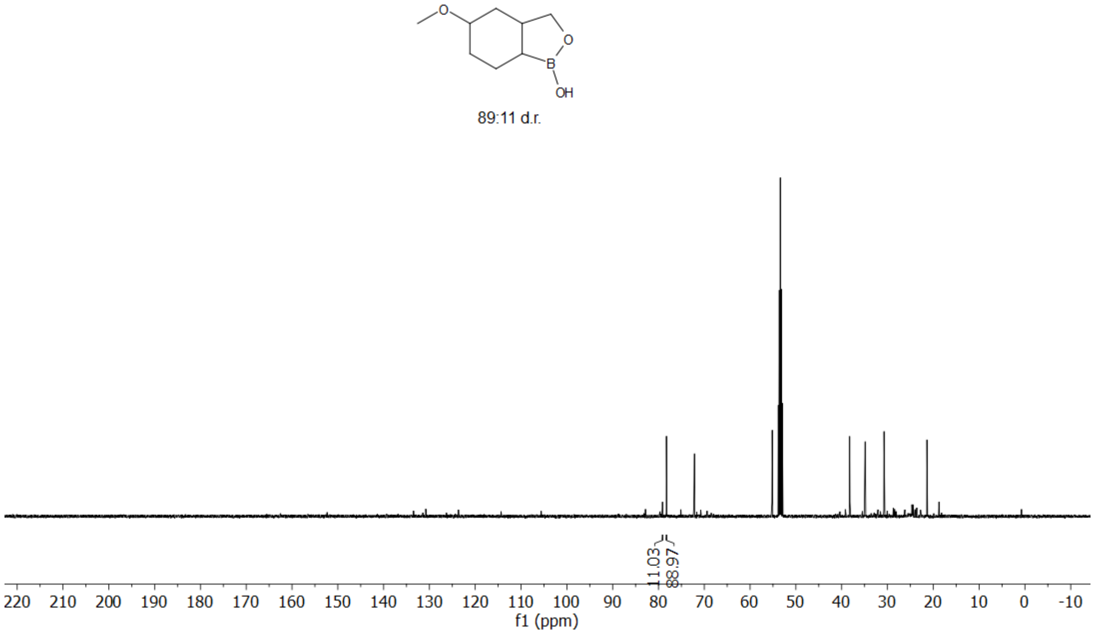

Figure S225: Quantitative $^{13}C\{^1H\}$ NMR (126 MHz, CD_2Cl_2, 298 K) reaction mixture spectrum of 5-methoxyhexahydrobenzo[*c*][1,2]oxaborol-1(3*H*)-ol (6b).

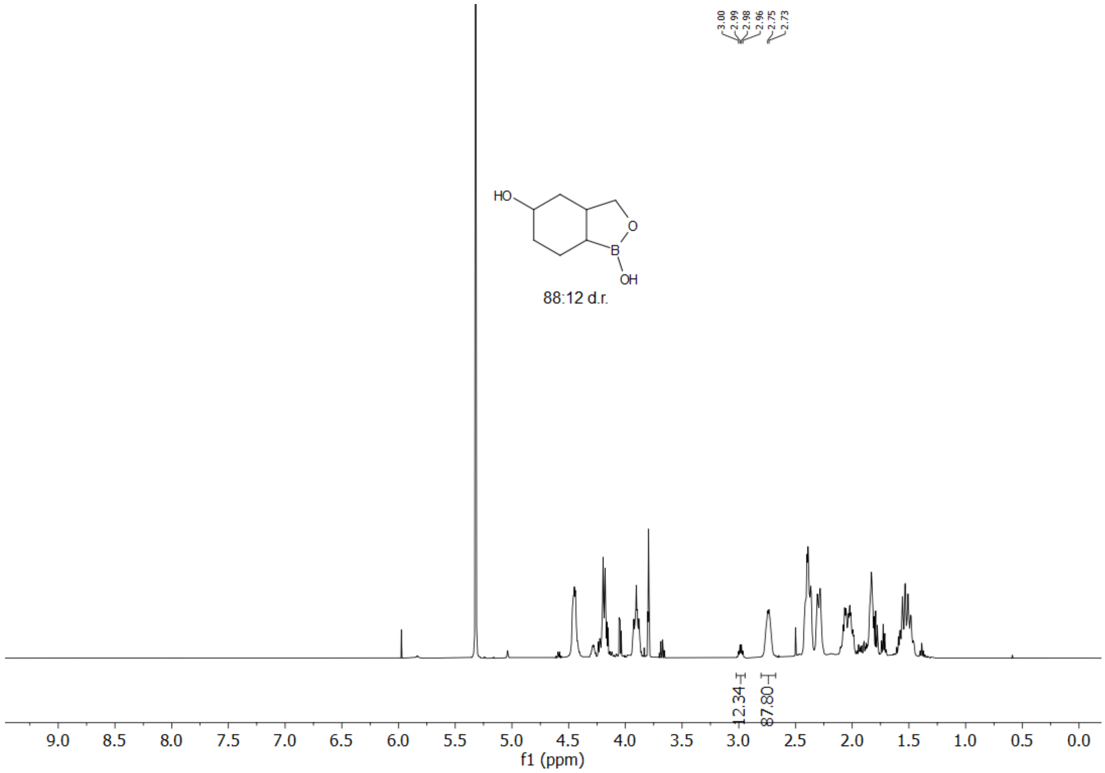

Figure S226: ^1H NMR (400 MHz, CD_3OD, 298 K) reaction mixture spectrum of Hexahydrobenzo[*c*][1,2]oxaborole-1,5(3*H*)-diol (6c).

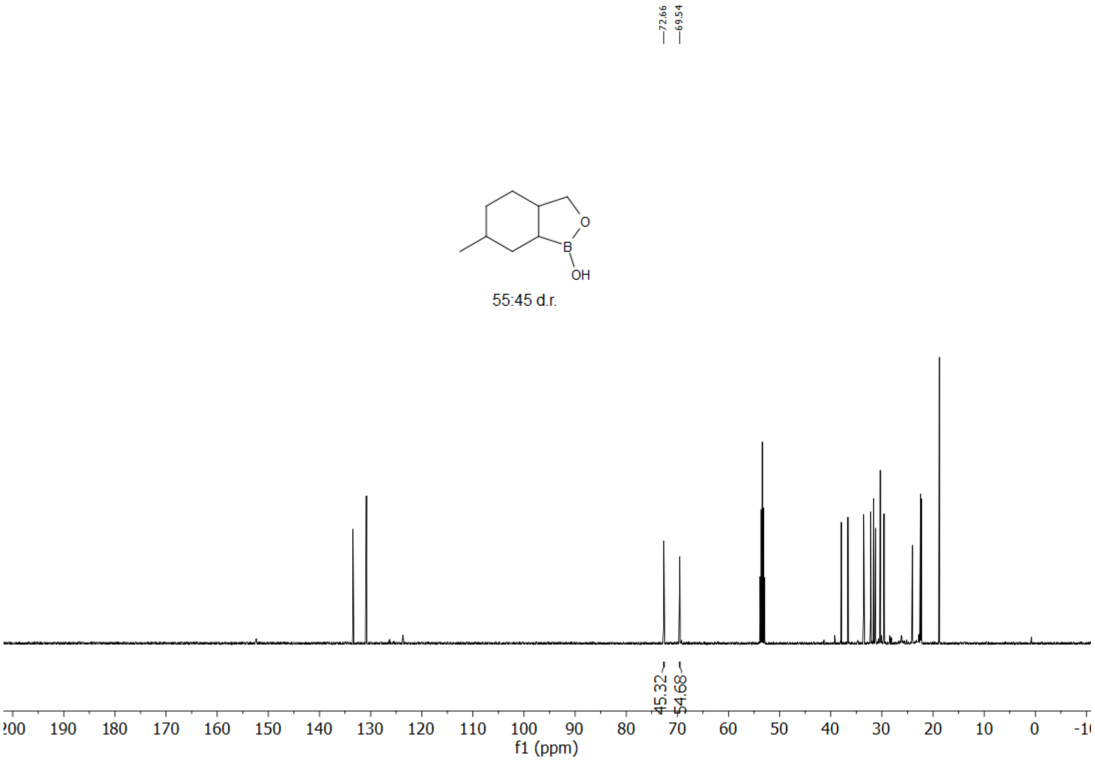

Figure S227: Quantitative $^{13}C\{^1H\}$ NMR (126 MHz, CD_2Cl_2, 298 K) reaction mixture spectrum of 5-methylhexahydrobenzo[*c*][1,2]oxaborol-1(3*H*)-ol (6e).

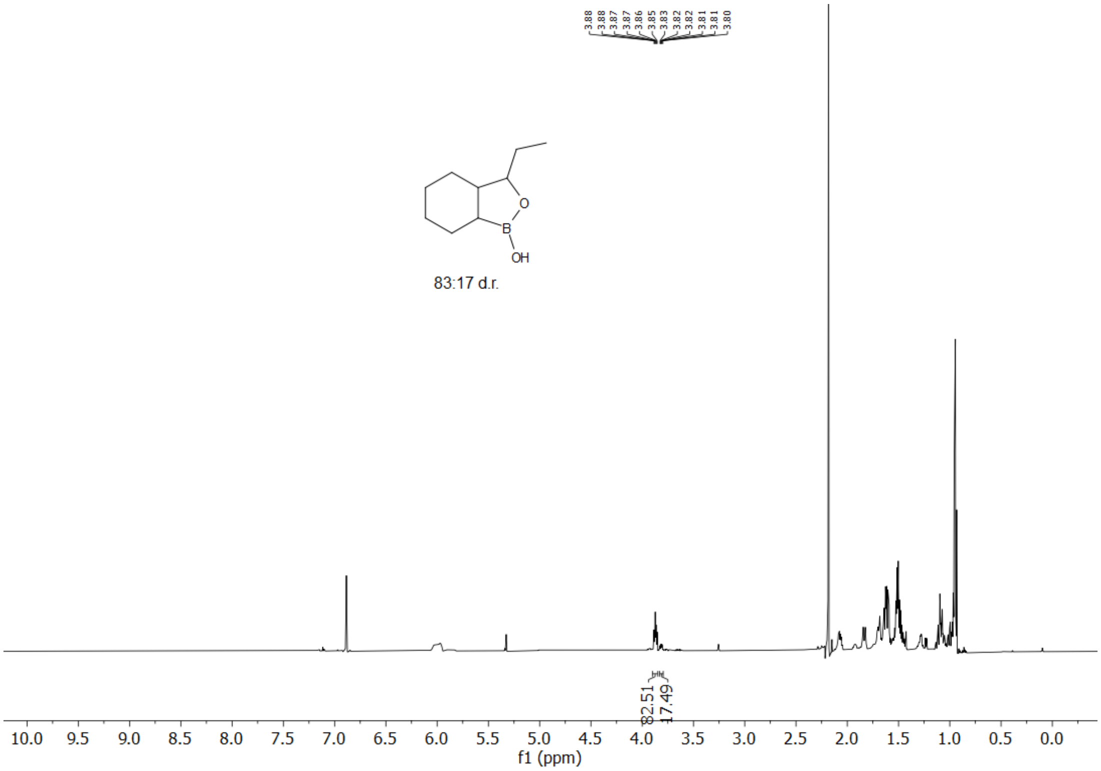

Figure S228: ^1H NMR (400 MHz, CD_2Cl_2, 298 K) reaction mixture spectrum of 3-ethylhexahydrobenzo[*c*][1,2]oxaborol-1(3*H*)-ol (6l).

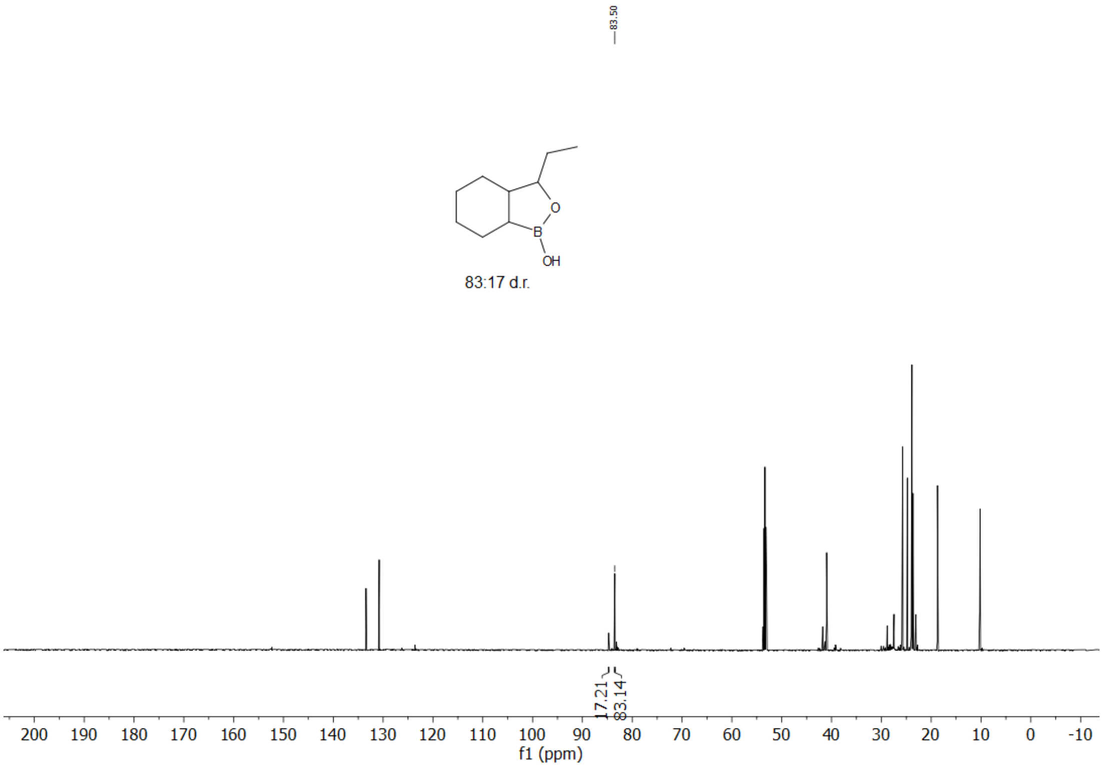

Figure S229: Quantitative $^{13}C\{^{1}H\}$ NMR (126 MHz, CD_2Cl_2, 298 K) reaction mixture spectrum of 3-ethylhexahydrobenzo[*c*][1,2]oxaborol-1(3*H*)-ol (6l).

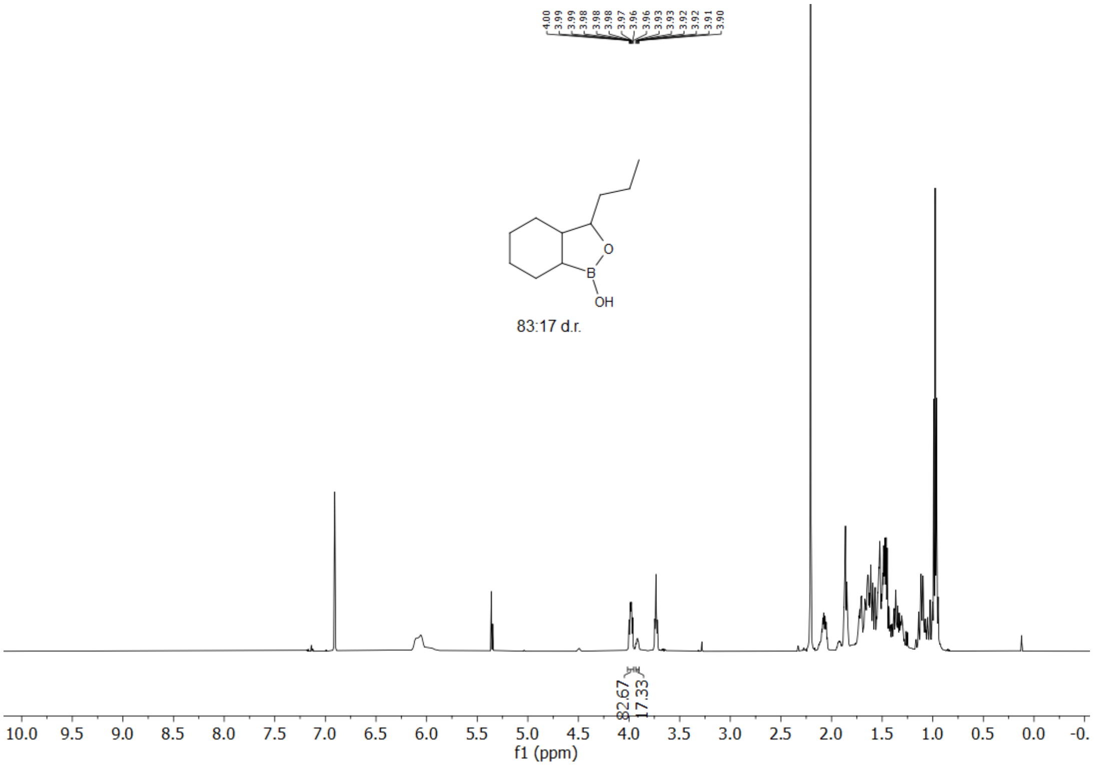

Figure S230: ^{1}H NMR (400 MHz, CD_2Cl_2, 298 K) reaction mixture spectrum of 3-propylhexahydrobenzo[*c*][1,2]oxaborol-1(3*H*)-ol (6m)

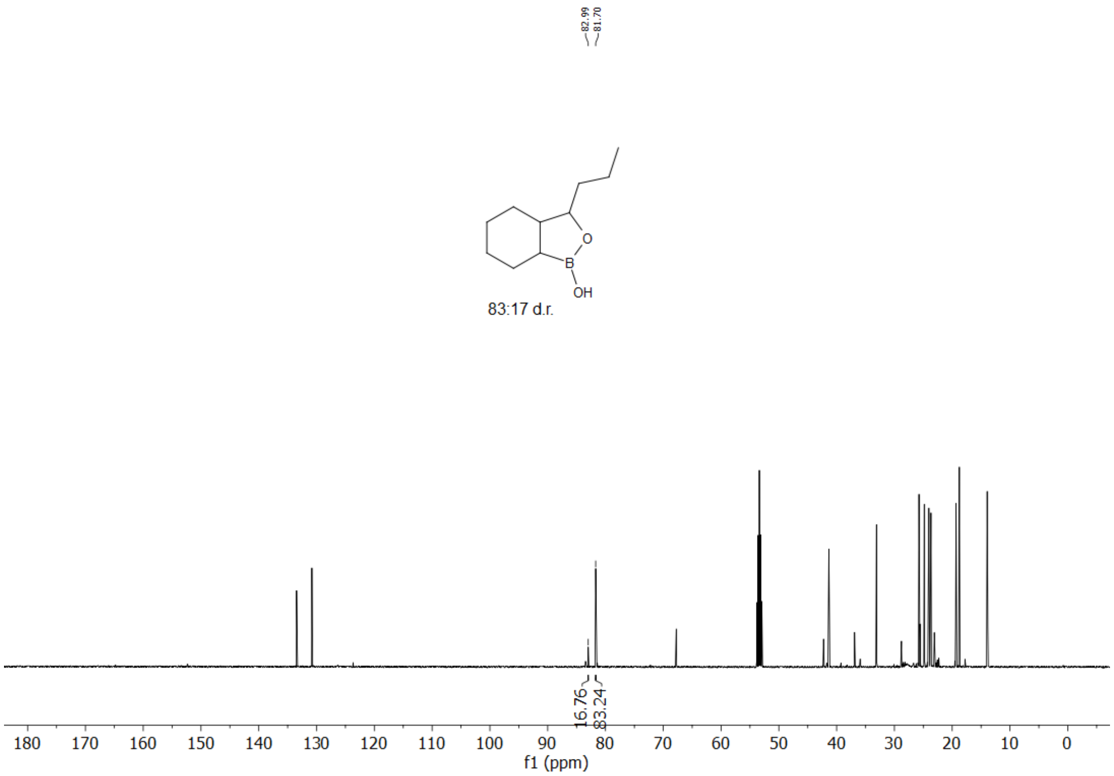

Figure S231: Quantitative $^{13}C\{^{1}H\}$ NMR (126 MHz, CD_2Cl_2, 298 K) reaction mixture spectrum of 3-propylhexahydrobenzo[*c*][1,2]oxaborol-1(3*H*)-ol (6m).

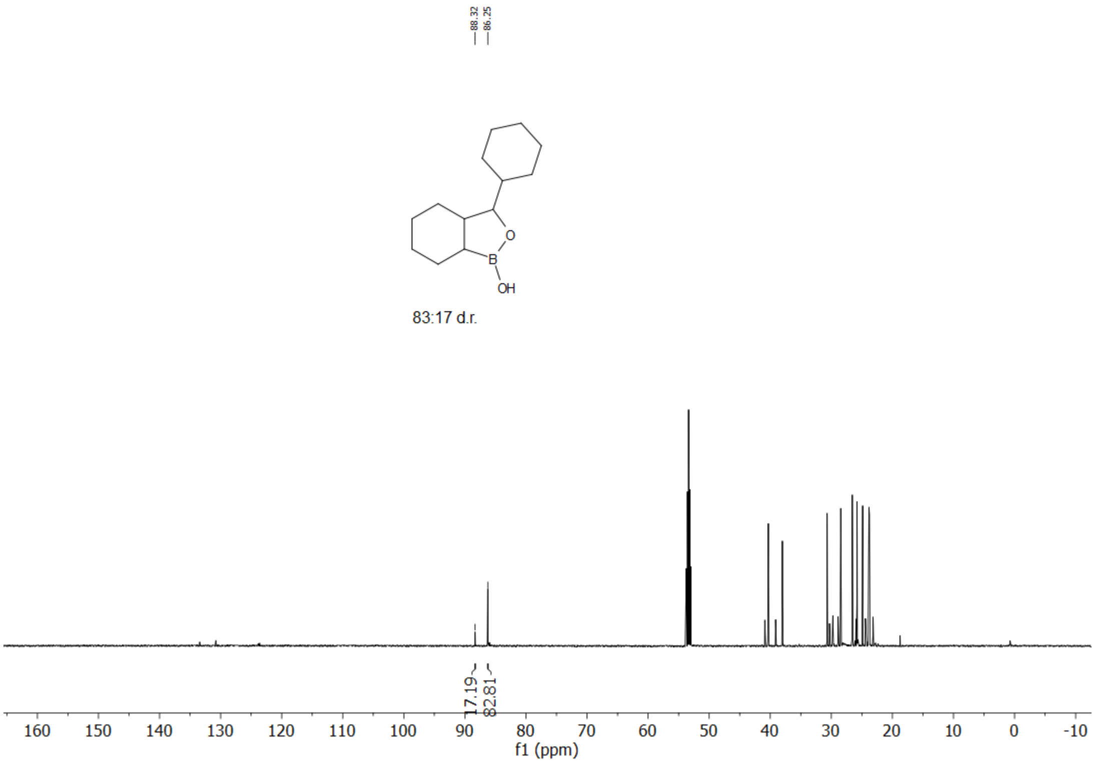

Figure S232: Quantitative $^{13}C\{^{1}H\}$ NMR (126 MHz, CD_2Cl_2, 298 K) reaction mixture spectrum of 3-cyclohexylhexahydrobenzo[*c*][1,2]oxaborol-1(3*H*)-ol (6n).

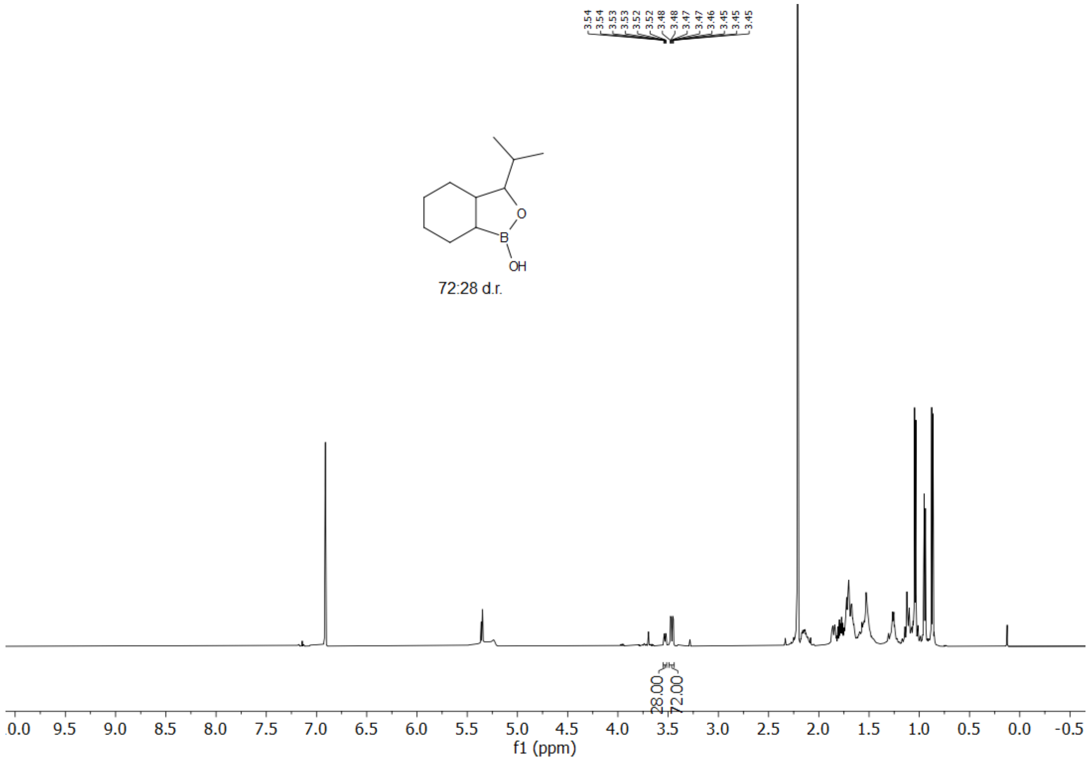

Figure S233: ^{1}H NMR (400 MHz, CD_2Cl_2, 298 K) reaction mixture spectrum of 3-isopropylhexahydrobenzo[*c*][1,2]oxaborol-1(3*H*)-ol (6o).

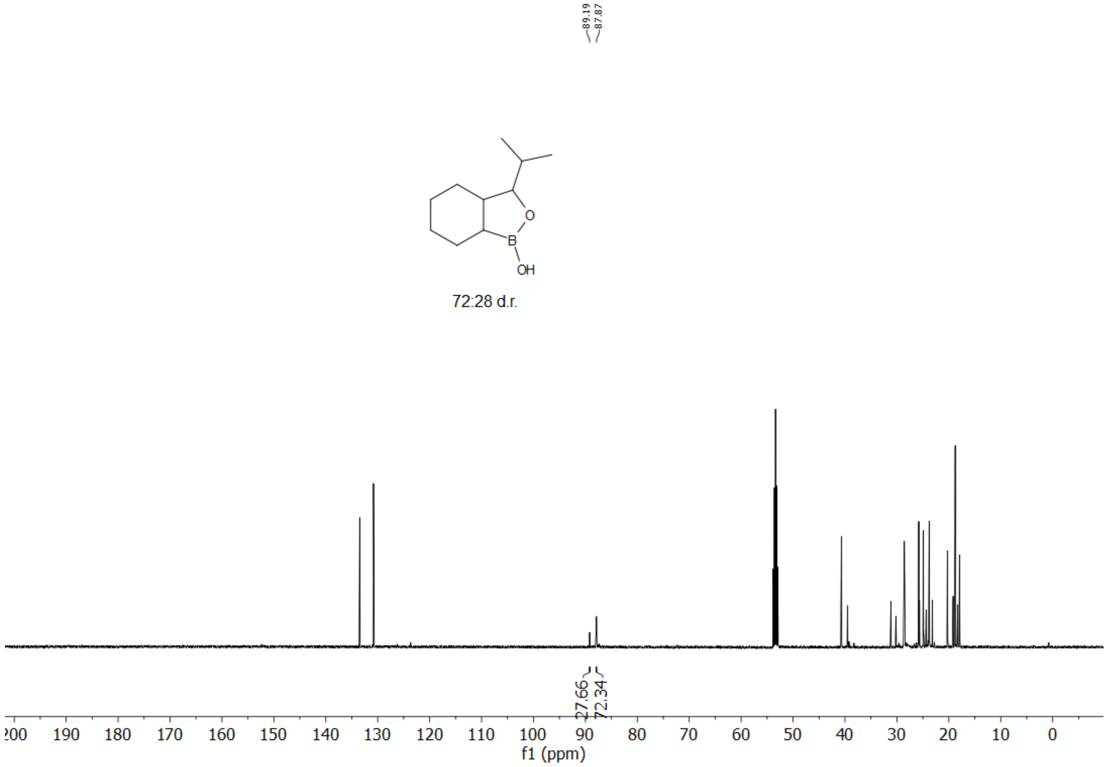

Figure S234: Quantitative $^{13}C\{^{1}H\}$ NMR (126 MHz, CD_2Cl_2, 298 K) reaction mixture spectrum of 3-isopropylhexahydrobenzo[*c*][1,2]oxaborol-1(3*H*)-ol (6o).

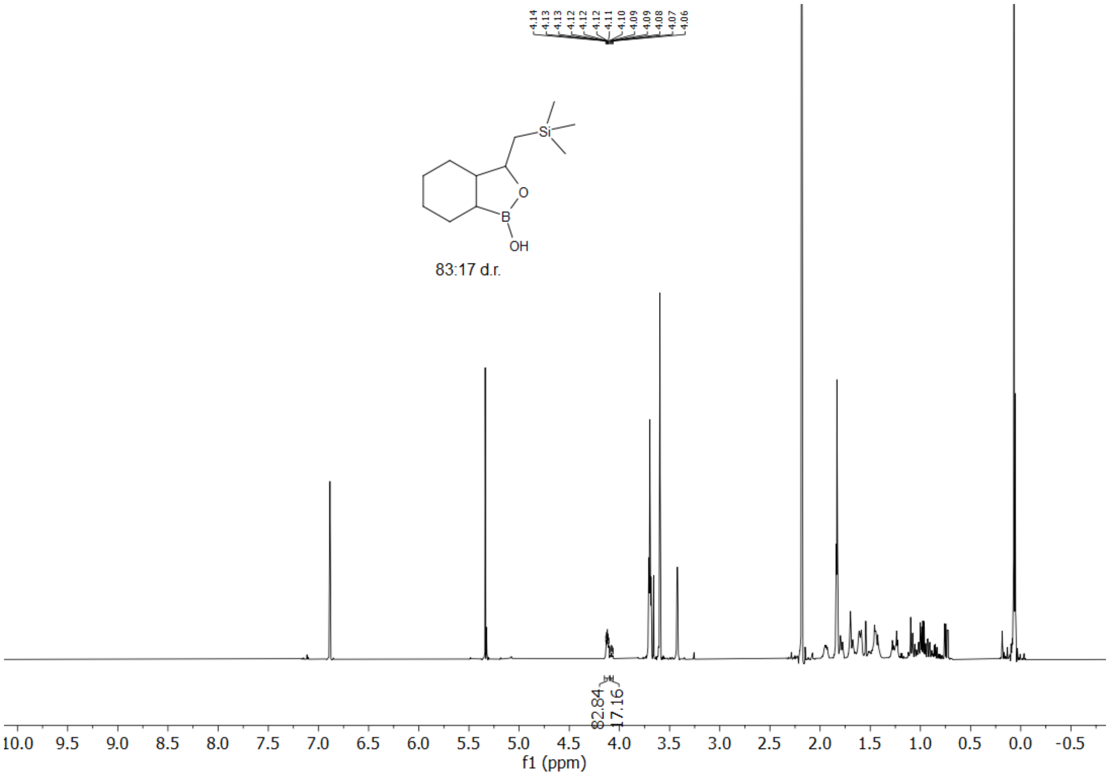

Figure S235: ^{1}H NMR (400 MHz, CD_2Cl_2, 298 K) reaction mixture spectrum of 3-((trimethylsilyl)methyl)hexahydrobenzo[*c*][1,2]oxaborol-1(3*H*)-ol (6p).

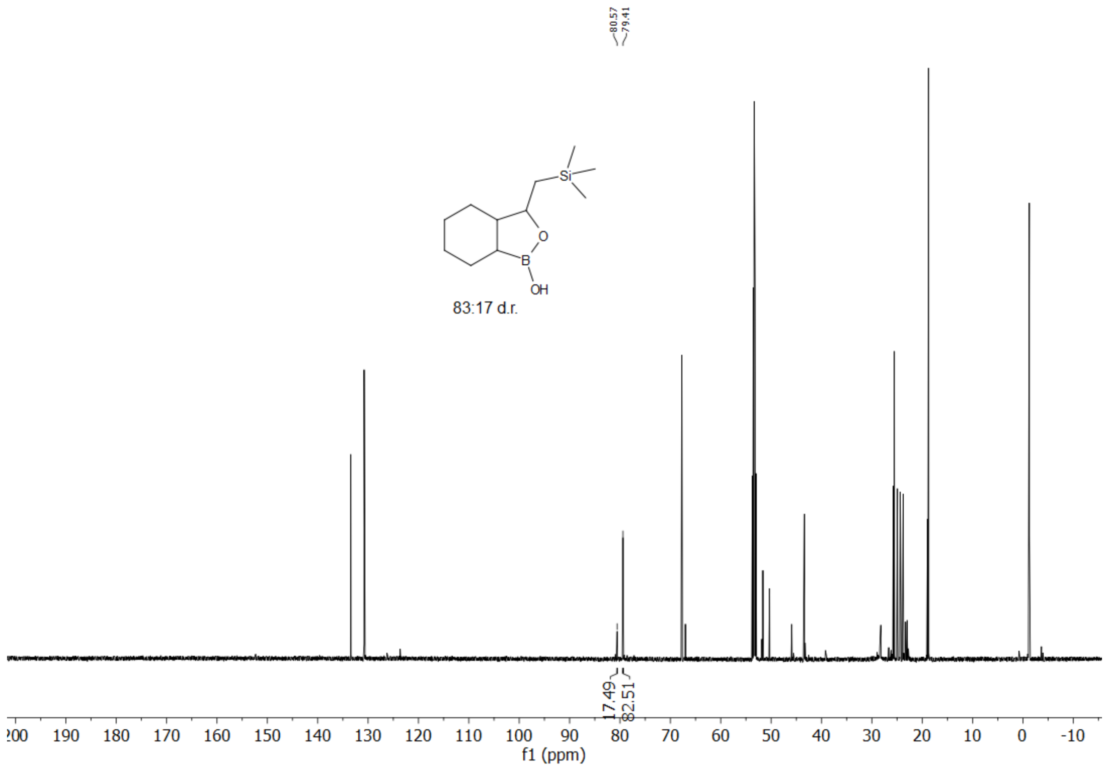

Figure S236: Quantitative $^{13}C\{^1H\}$ NMR (126 MHz, CD_2Cl_2, 298 K) reaction mixture spectrum of 3-((trimethylsilyl)methyl)hexahydrobenzo[*c*][1,2]oxaborol-1(3*H*)-ol (6p).

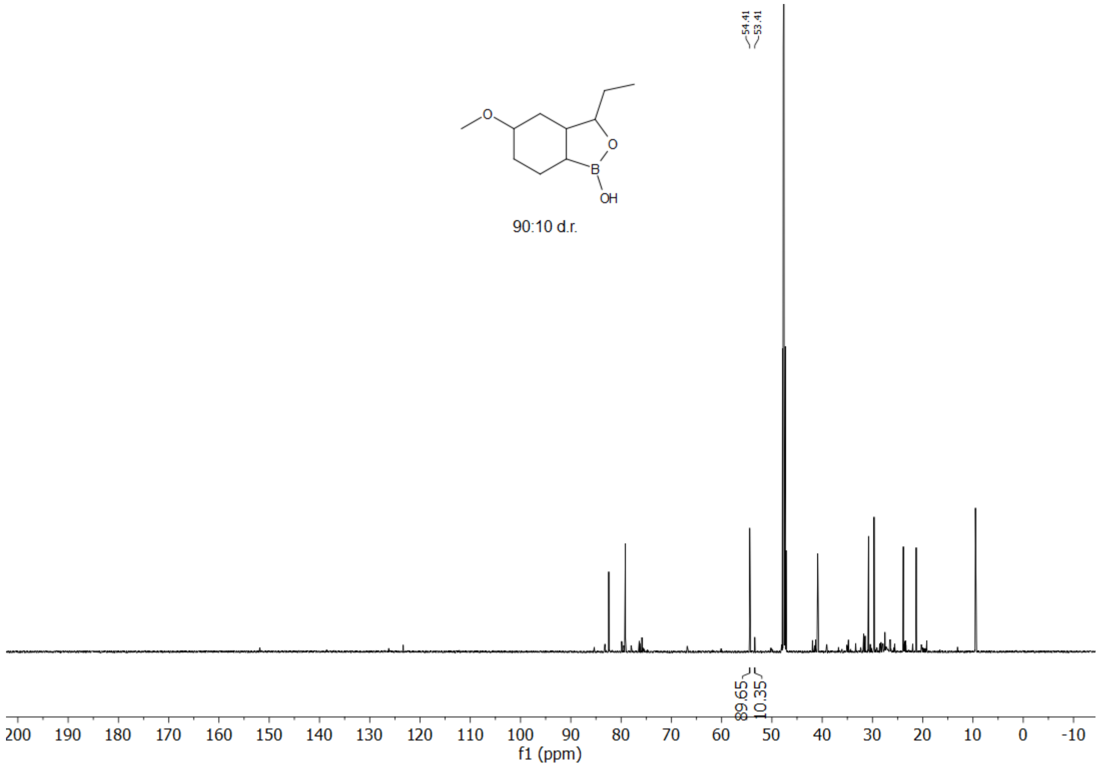

Figure S237: Quantitative $^{13}C\{^{1}H\}$ NMR (126 MHz, CD_2Cl_2, 298 K) reaction mixture spectrum of 3-ethyl-5-methoxyhexahydrobenzo[*c*][1,2]oxaborol-1(3*H*)-ol (6q).

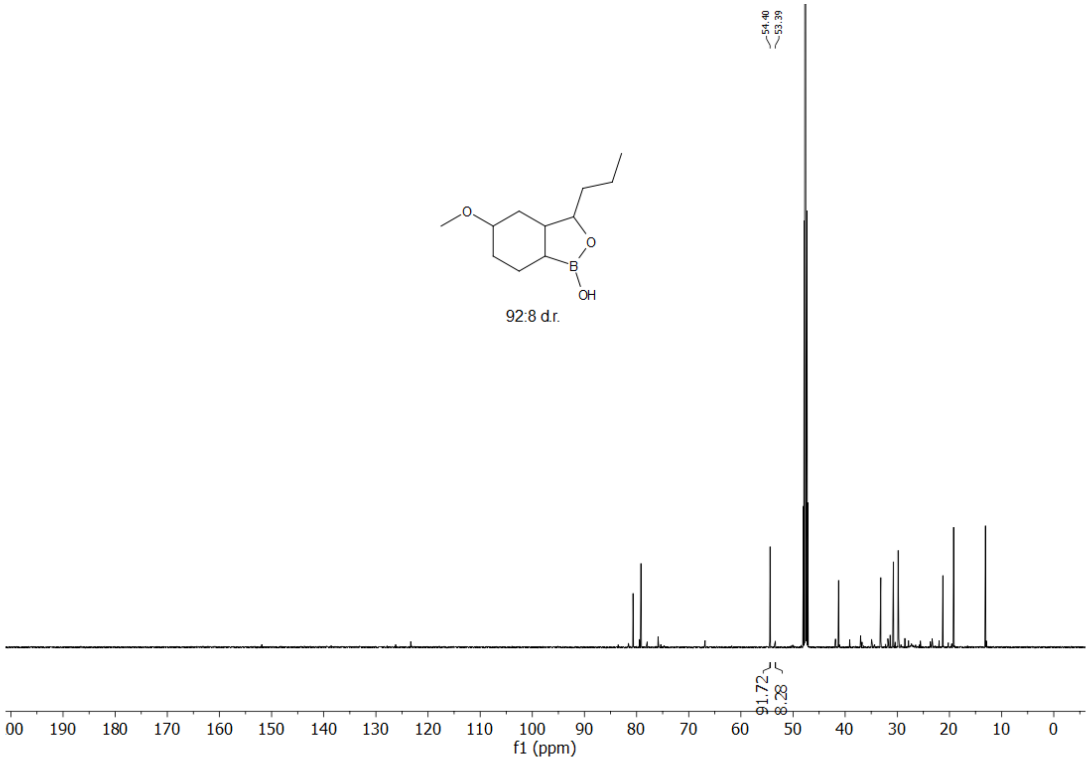

Figure S238: Quantitative $^{13}C\{^{1}H\}$ NMR (126 MHz, CD_2Cl_2, 298 K) reaction mixture spectrum of 5-methoxy-3-propylhexahydrobenzo[*c*][1,2]oxaborol-1(3*H*)-ol (6r).

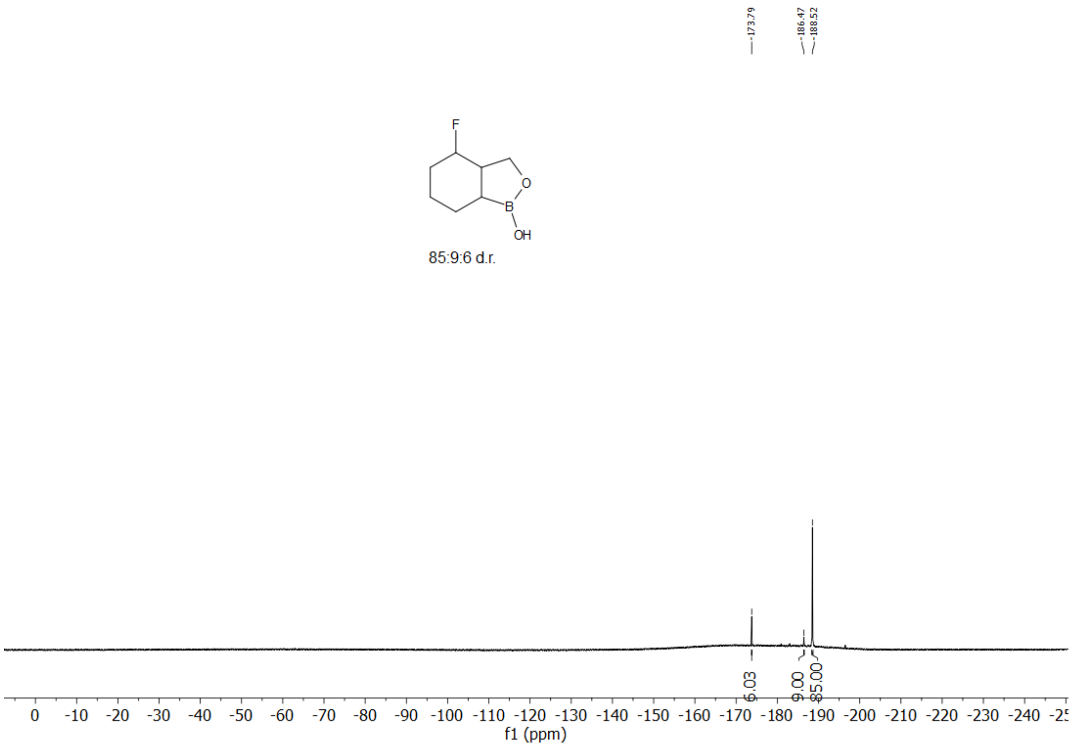

Figure S239: $^{19}F\{^{1}H\}$ NMR (376 MHz, CD_2Cl_2, 298 K) reaction mixture spectrum of 4-fluorohexahydrobenzo[*c*][1,2]oxaborol-1(3*H*)-ol (6t).

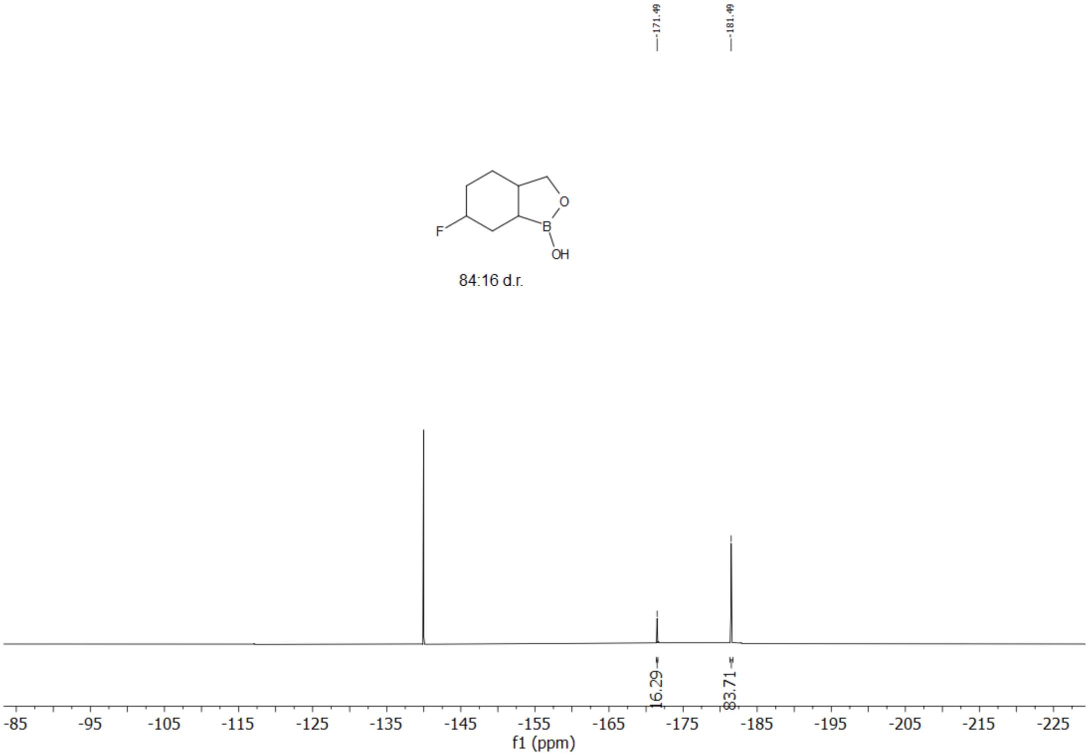

Figure S240: $^{19}F\{^{1}H\}$ NMR (376 MHz, CD_2Cl_2, 298 K) reaction mixture spectrum of 6-fluorohexahydrobenzo[*c*][1,2]oxaborol-1(3*H*)-ol (6u).

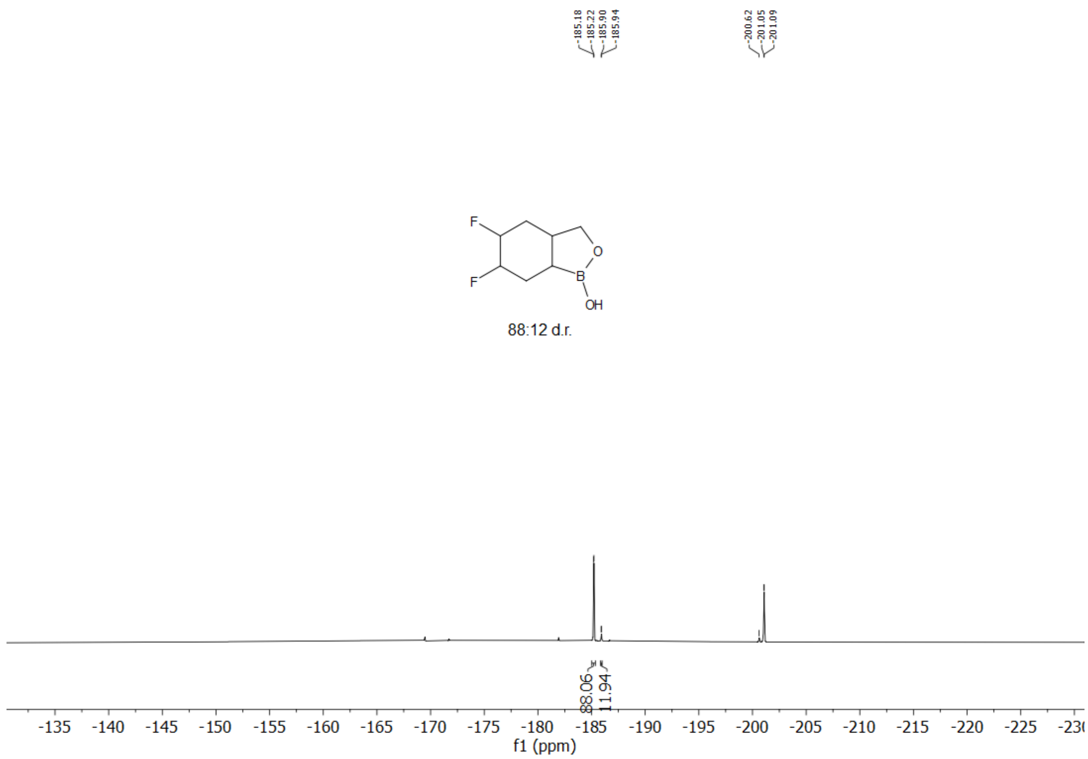

Figure S241: $^{19}F\{^{1}H\}$ NMR (376 MHz, $CDCl_3$, 298 K) reaction mixture spectrum of 5,6-difluorohexahydrobenzo[*c*][1,2]oxaborol-1(3*H*)-ol (6v).

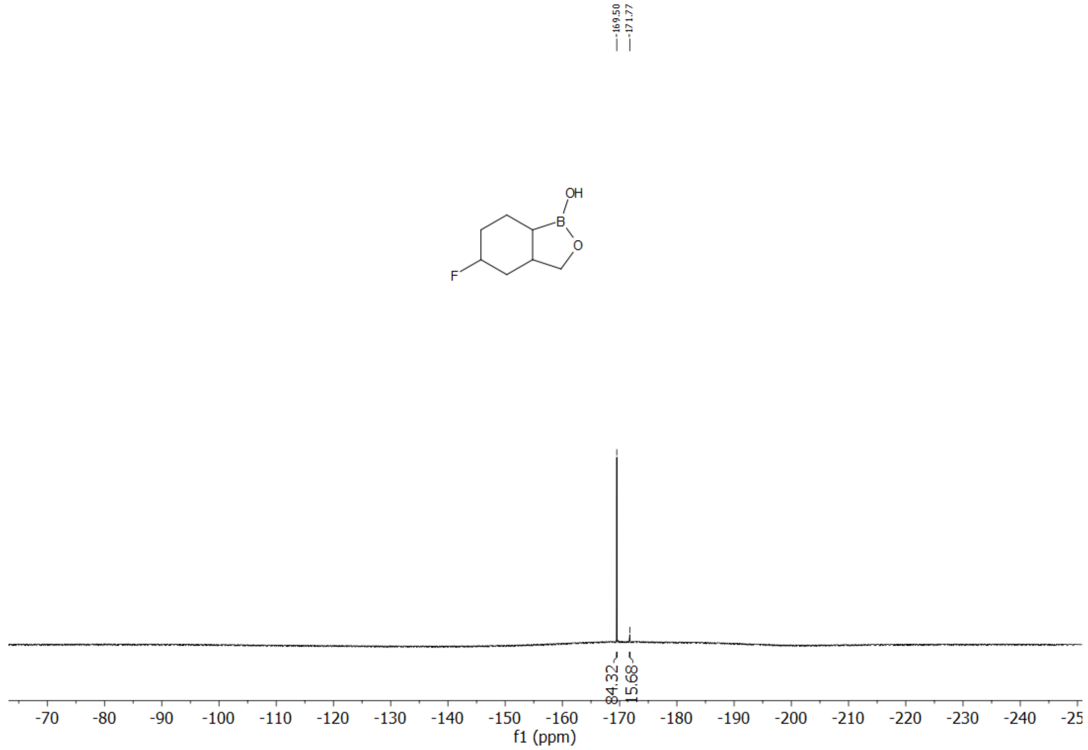

Figure S242: $^{19}F\{^{1}H\}$ NMR (376 MHz, $CDCl_3$, 298 K) reaction mixture spectrum of 5-fluorohexahydrobenzo[*c*][1,2]oxaborol-1(3*H*)-ol (6w)

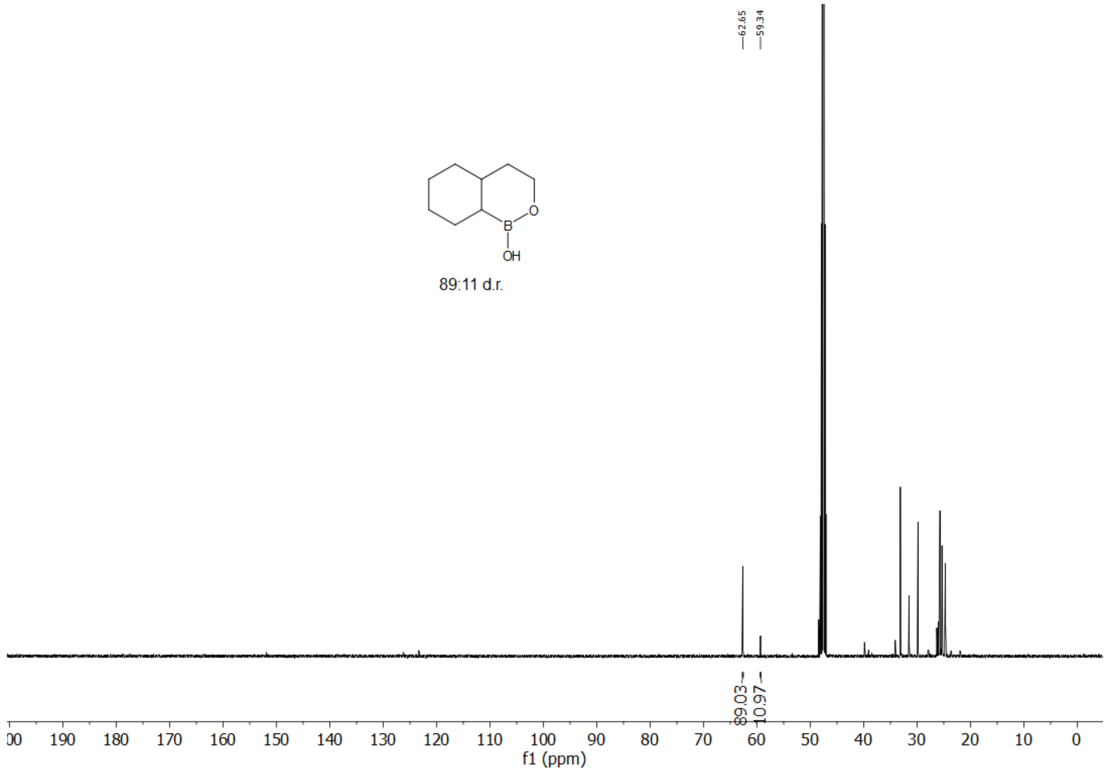

Figure S243: Quantitative $^{13}C\{^1H\}$ NMR (126 MHz, CD_2Cl_2, 298 K) reaction mixture spectrum of Octahydro-1*H*-benzo[*c*][1,2]oxaborinin-1-ol (8a).

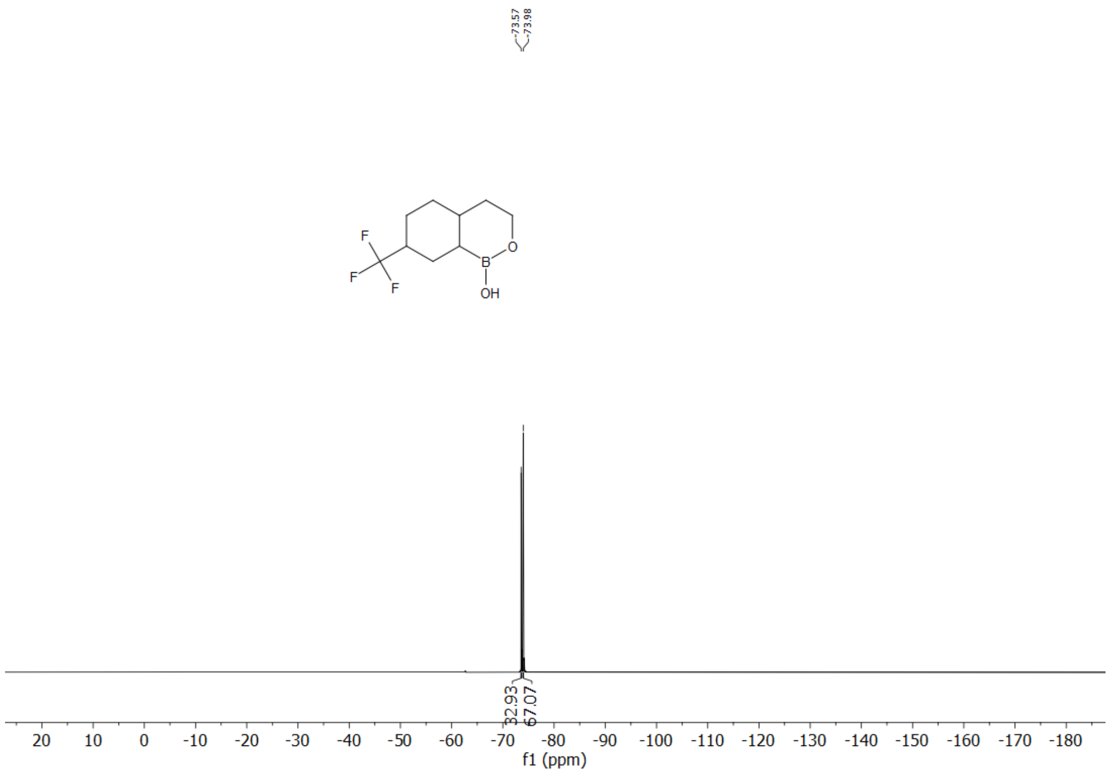

Figure S244: $^{19}F\{^1H\}$ NMR (376 MHz, $CDCl_3$, 298 K) reaction mixture spectrum of 7-(trifluoromethyl)octahydro-1*H*-benzo[*c*][1,2]oxaborinin-1-ol (8c).

32. Representative NMR spectra at different pressures for the hydrogenation of benzoxaborole derivatives to determine the diastereomeric ratio (Table S4-S7)

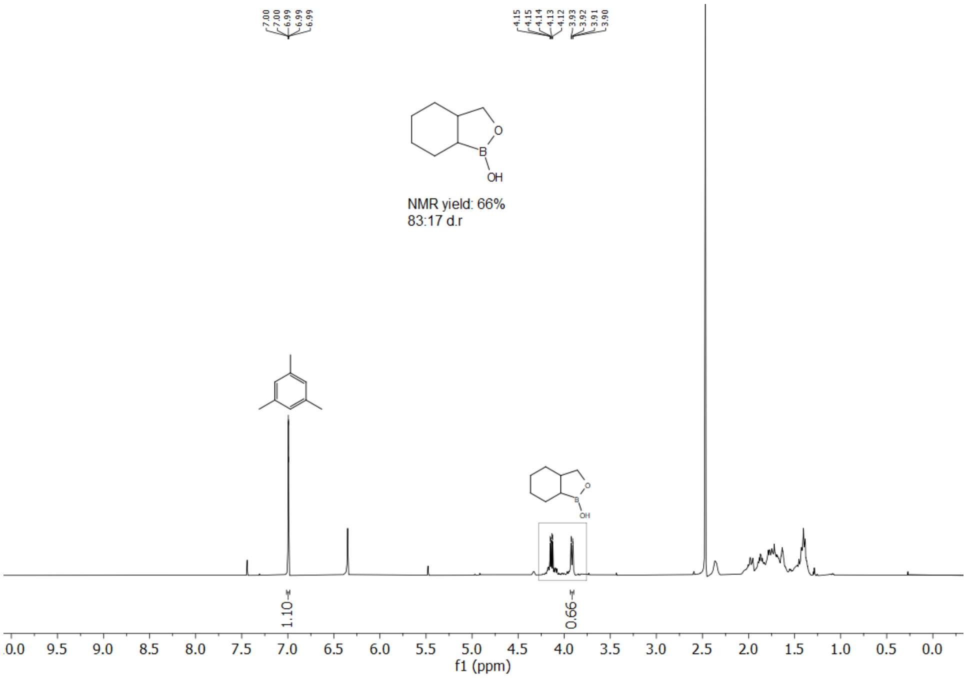

Figure S245: ^1H NMR (400 MHz, $CDCl_3$, 298 K) reaction mixture spectrum of Hexahydrobenzo[*c*][1,2]oxaborol-1(3*H*)-ol (6a) at 60 bar of H_2 pressure.

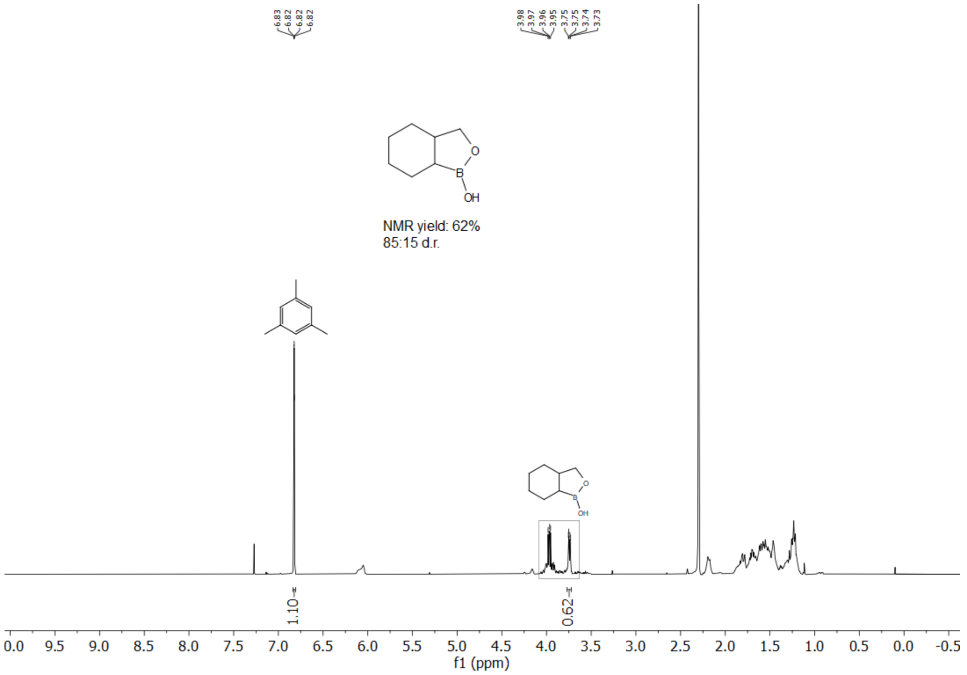

Figure S246: ^1H NMR (400 MHz, $CDCl_3$, 298 K) reaction mixture spectrum of Hexahydrobenzo[*c*][1,2]oxaborol-1(3*H*)-ol (6a) at 80 bar of H_2 pressure.

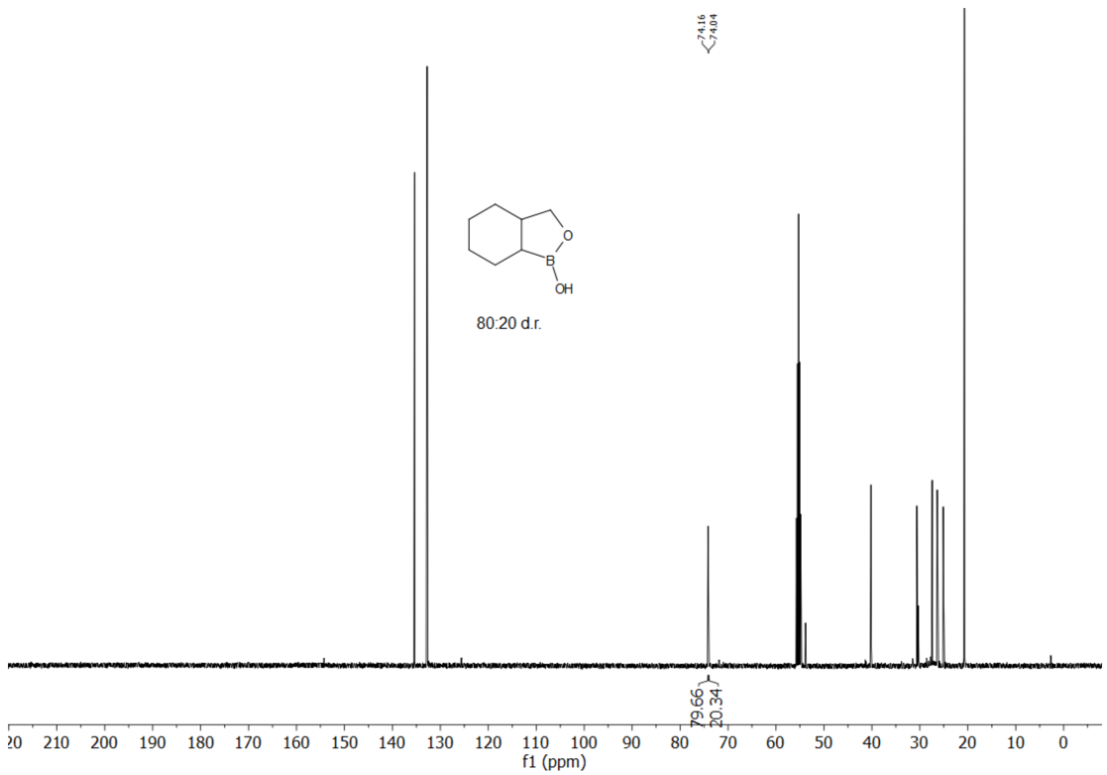

Figure S247: Quantitative $^{13}C\{^{1}H\}$ NMR (126 MHz, CD_2Cl_2, 298 K) reaction mixture spectrum of Hexahydrobenzo[*c*][1,2]oxaborol-1(3*H*)-ol (6a) at 40 bar of H_2 pressure.

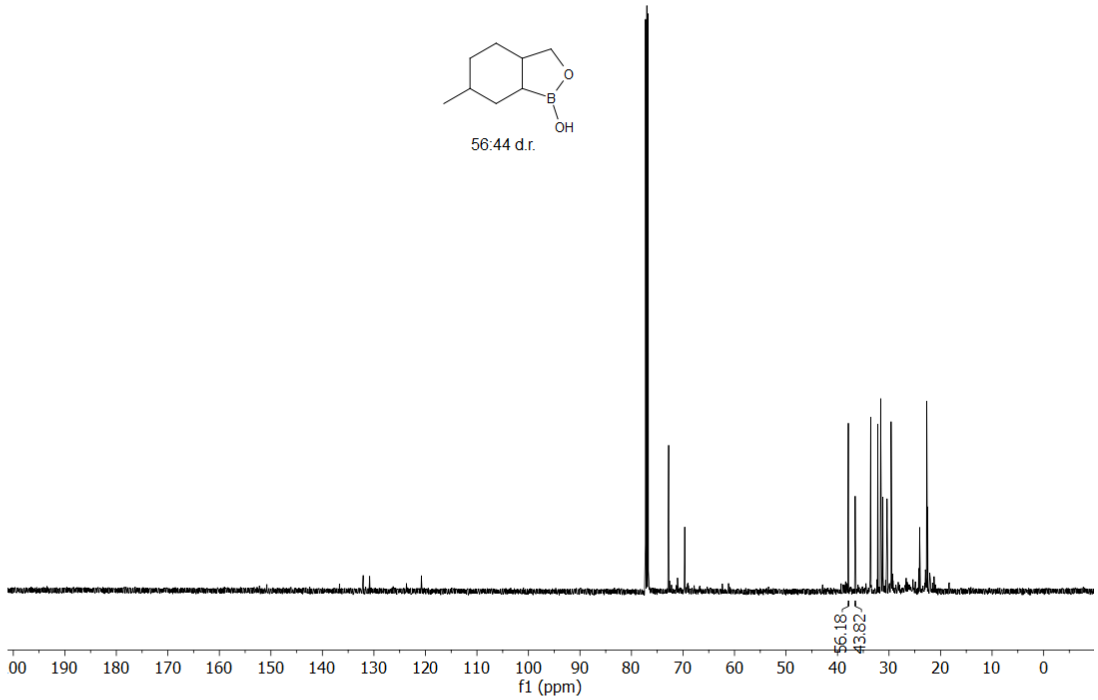

Figure S248: Quantitative $^{13}C\{^{1}H\}$ NMR (126 MHz, $CDCl_3$, 298 K) reaction mixture spectrum of 6-methylhexahydrobenzo[c][1,2]oxaborol-1(3H)-ol (6e) at 80 bar of H_2 pressure.

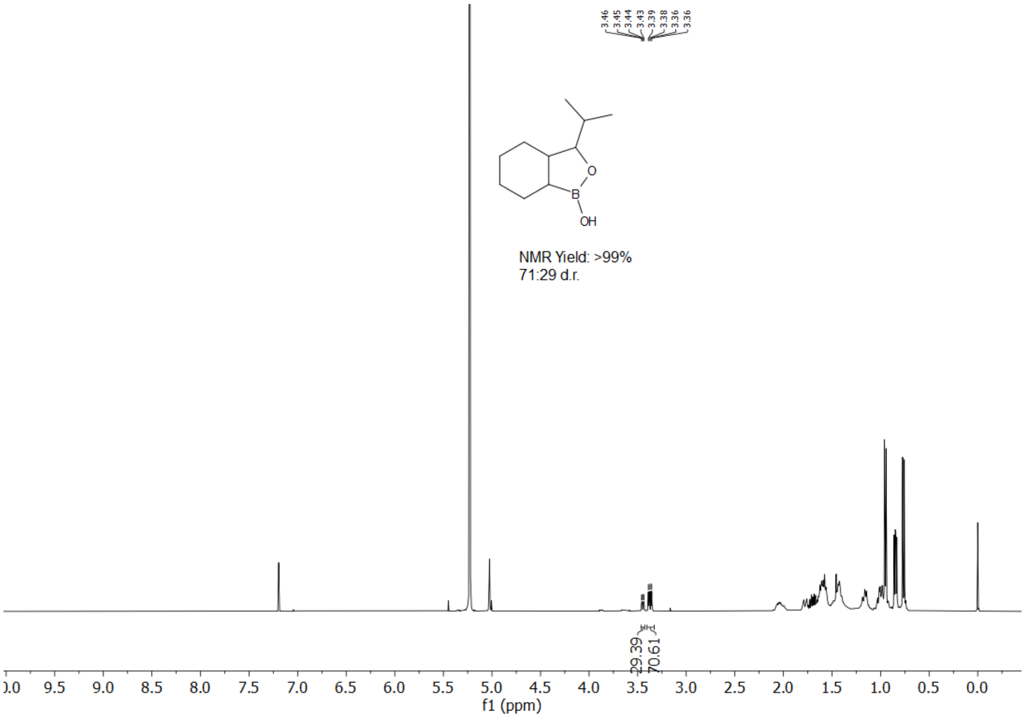

Figure S249: ^{1}H NMR (400 MHz, $CDCl_3$, 298 K) reaction mixture spectrum of 3-isopropylhexahydrobenzo[c][1,2]oxaborol-1(3H)-ol (6o) at 80 bar of H_2 pressure.

33. NMR spectra for the hydrogenation of benzoxaborole derivatives using Rhodium on carbon (Rh/C) as a catalyst

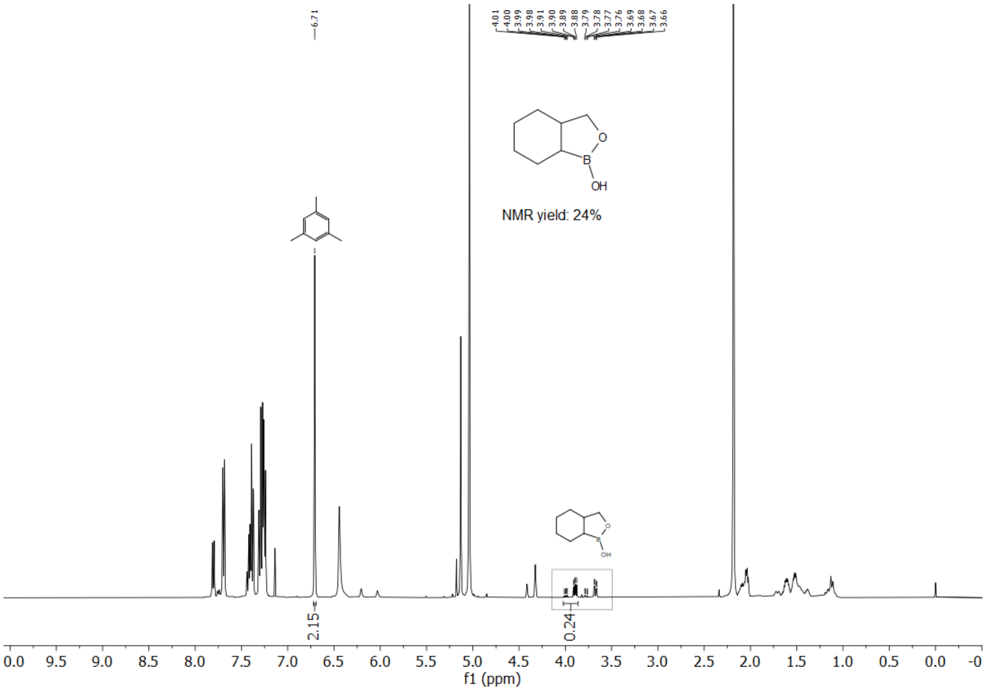

Figure S250: ^{1}H NMR (400 MHz, $CDCl_3$, 298 K) reaction mixture spectrum for the hydrogenation of benzoxaborole (5a) using Rh/C.

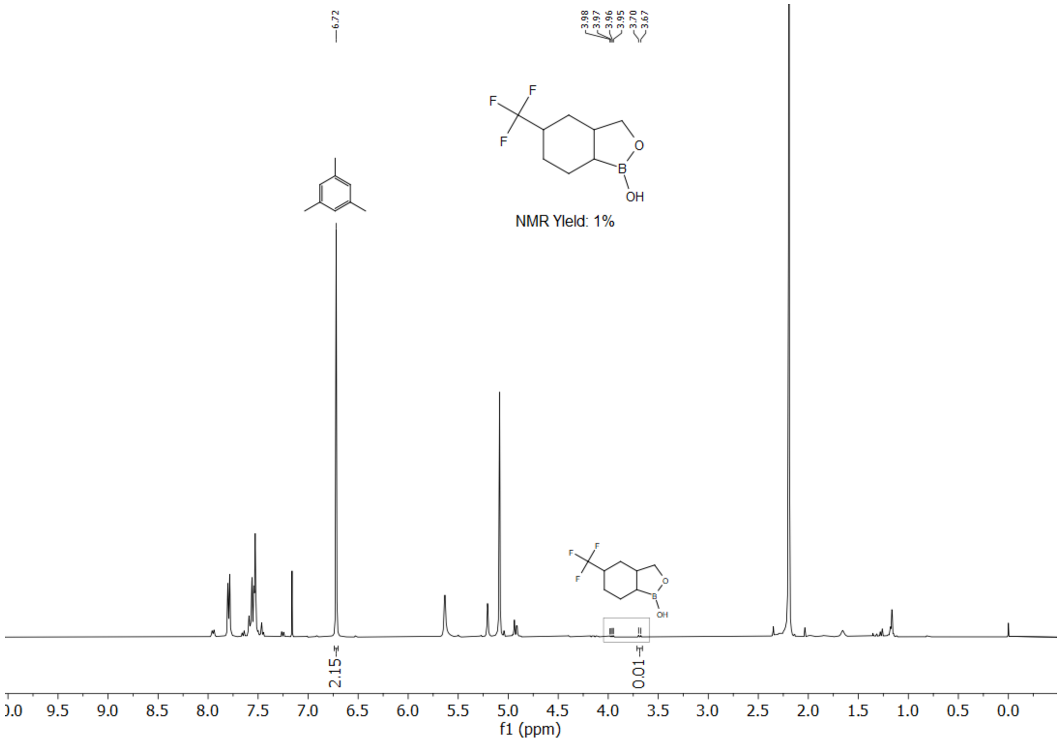

Figure S251: ^{1}H NMR (400 MHz, $CDCl_3$, 298 K) reaction mixture spectrum for the hydrogenation of 5-(trifluoromethyl)benzo[c][1,2]oxaborol-1(3H)-ol (5d) using Rh/C.

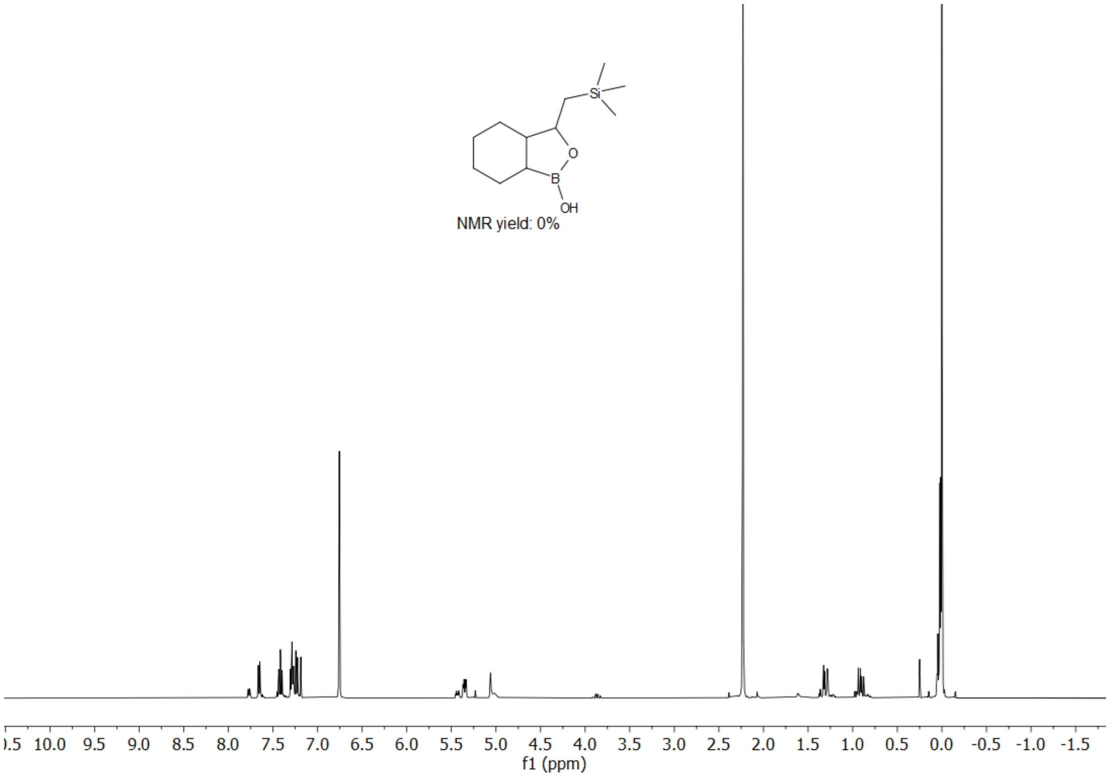

Figure S252: ^{1}H NMR (400 MHz, $CDCl_3$, 298 K) reaction mixture spectrum for the hydrogenation of 3-((trimethylsilyl)methyl)benzo[c][1,2]oxaborol-1(3H)-ol (5p) using Rh/C.

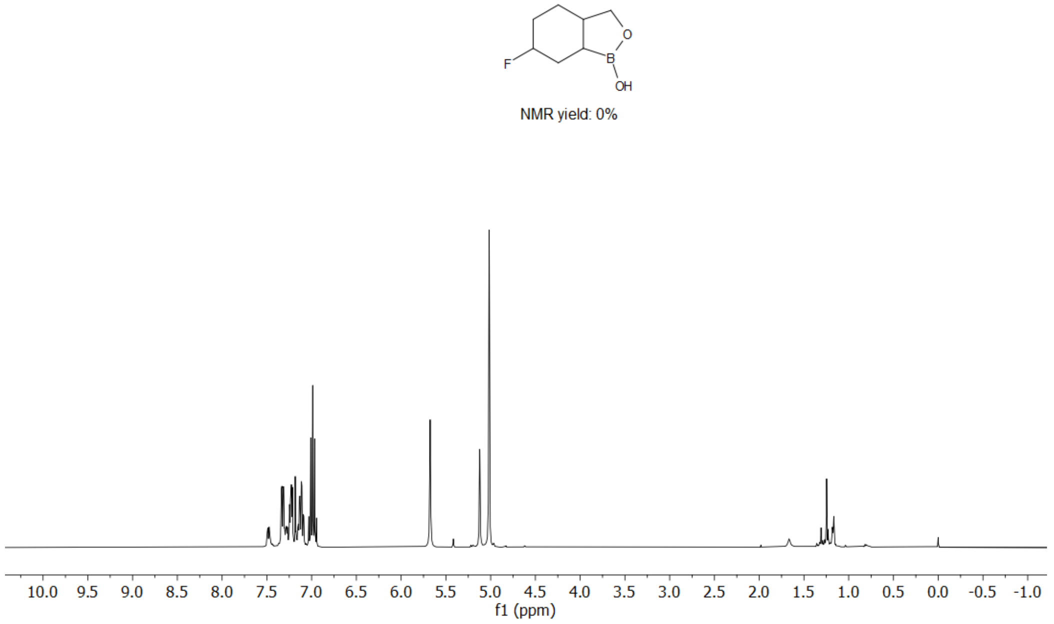

Figure S253: ^{1}H NMR (400 MHz, $CDCl_3$, 298 K) reaction mixture spectrum for the hydrogenation of 6-fluorobenzo[c][1,2]oxaborol-1(3H)-ol (5u) using Rh/C.

34.NMR spectra for the hydrogenation of 5a using filtration test

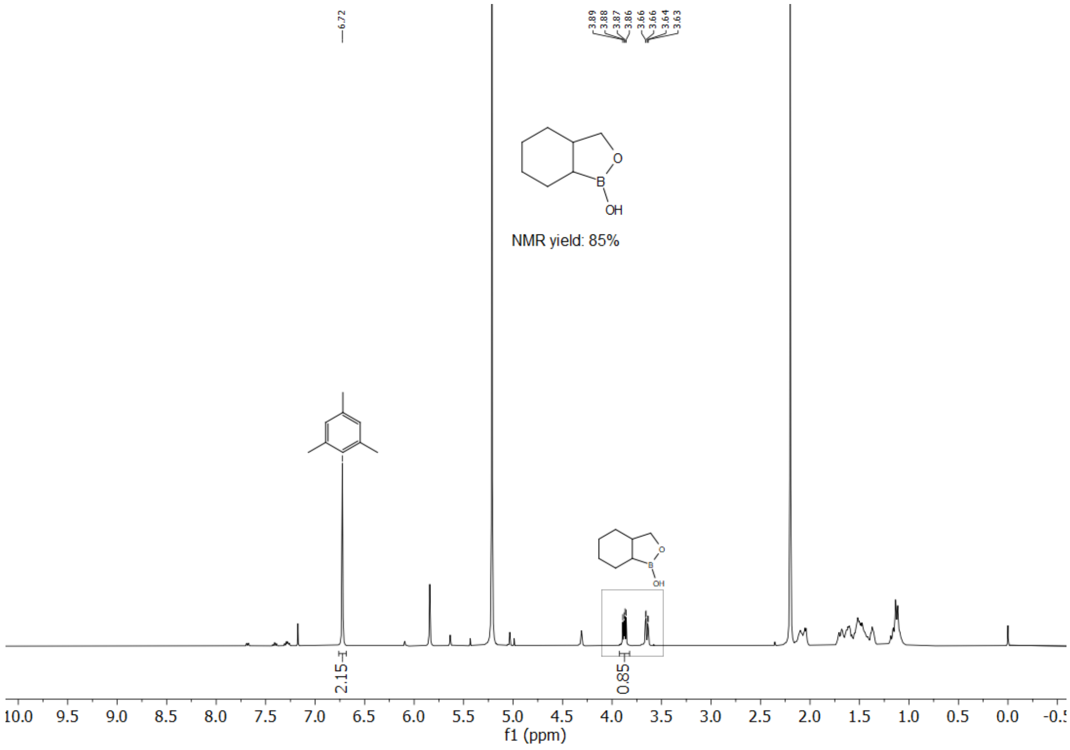

Figure S254: ^{1}H NMR (400 MHz, $CDCl_3$, 298 K) reaction mixture spectrum for the hydrogenation of benzoxaborole (5a) using recovered [Rh] on 4 Å MS catalyst.

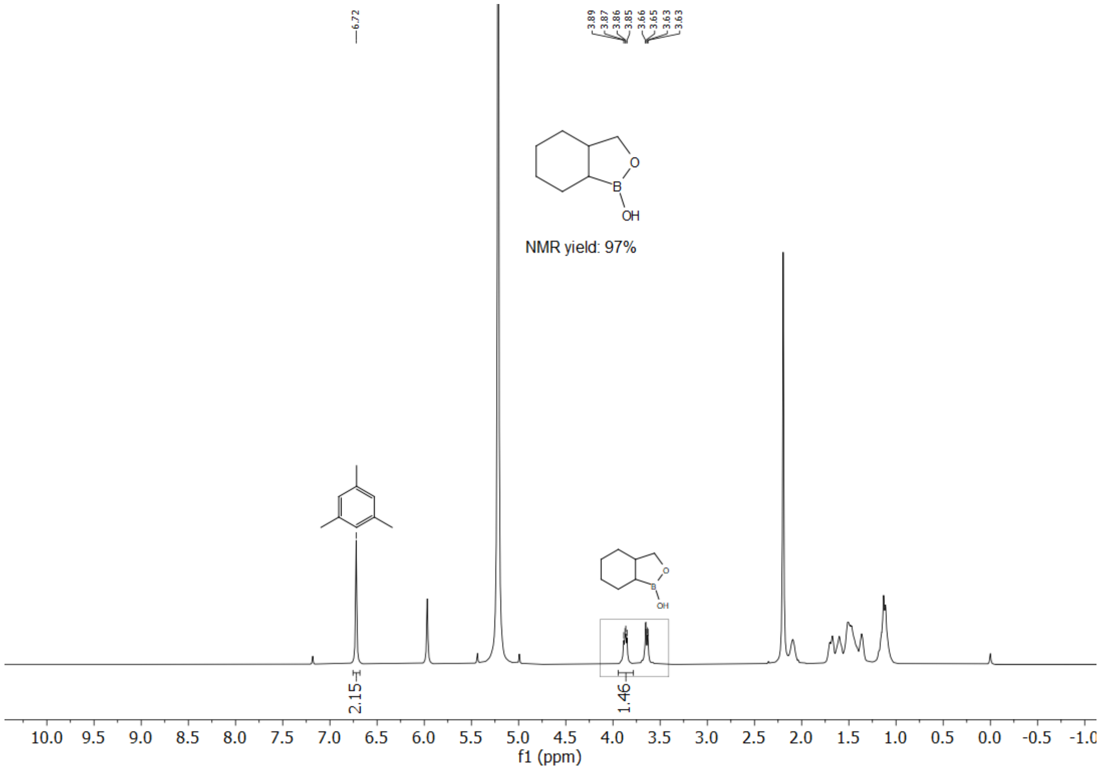

Figure S255: ^{1}H NMR (400 MHz, $CDCl_3$, 298 K) reaction mixture spectrum for the hydrogenation of benzoxaborole (5a) using preformed [Rh] on 4 Å MS catalyst.

References

[1] G. R. Fulmer, A. J. M. Miller, N. H. Sherden, H. E. Gottlieb, A. Nudelman, B. M. Stoltz, J. E. Bercaw, K. I. Goldberg, *Organometallics* **2010**, *29*, 2176-2179.

[2] J. Zhang, M. Zhu, Y. Lin, H. Zhou, *Sci. China Chem.* **2013**, *56*, 1372-1381.

[3] J. F. Cairns, H. R. Snyder, *J. Org. Chem.* **1964**, *29*, 2810-2812.

[4] A. Adamczyk-Woźniak, M. K. Cabaj, P. M. Dominiak, P. Gajowiec, B. Gierczyk, J. Lipok, Ł. Popenda, G. Schroeder, E. Tomecka, P. Urbański, D. Wieczorek, A. Sporzyński, *Bioorg. Chem.* **2015**, *60*, 130-135.

[5] R. Bisht, B. Chattopadhyay, *J. Am. Chem. Soc.* **2016**, *138*, 84-87.

[6] S. Rej, N. Chatani, *J. Am. Chem. Soc.* **2021**, *143*, 2920-2929.

[7] S. J. Baker, Y.-K. Zhang, T. Akama, A. Lau, H. Zhou, V. Hernandez, W. Mao, M. R. K. Alley, V. Sanders, J. J. Plattner, *J. Med. Chem.* **2006**, *49*, 4447-4450.

[8] R. T. Jacobs, C. S. Lunde, Y. R. Freund, V. Hernandez, X. Li, Y. Xia, D. S. Carter, P. W. Berry, J. Halladay, F. Rock, R. Stefanakis, E. Easom, J. J. Plattner, L. Ford, K. L. Johnston, D. A. N. Cook, R. Clare, A. Cassidy, L. Myhill, H. Tyrer, J. Gamble, A. F. Guimaraes, A. Steven, F. Lenz, A. Ehrens, S. J. Frohberger, M. Koschel, A. Hoerauf, M. P. Hübner, C. W. McNamara, M. A. Bakowski, J. D. Turner, M. J. Taylor, S. A. Ward, *J. Med. Chem.* **2019**, *62*, 2521-2540.

[9] X.-P. Huang, J. Karpiak, W. K. Kroeze, H. Zhu, X. Chen, S. S. Moy, K. A. Saddoris, V. D. Nikolova, M. S. Farrell, S. Wang, T. J. Mangano, D. A. Deshpande, A. Jiang, R. B. Penn, J. Jin, B. H. Koller, T. Kenakin, B. K. Shoichet, B. L. Roth, *Nature* **2015**, *527*, 477-483.

[10] M. I. Naumov, S. A. Sutirin, A. S. Shavyrin, O. G. Ganina, I. P. Beletskaya, V. Bourgarel-Rey, S. Combes, J.-P. Finet, A. Y. Fedorov, *J. Org. Chem.* **2007**, *72*, 3293-3301.
[11] J. Zhu, Y. Wei, D. Lin, C. Ou, L. Xie, Y. Zhao, W. Huang, *Org. Biomol. Chem* **2015**, *13*, 11362-11368.
[12] S. H. Kim, C. Liu, Y. Zhou, Y.-K. Zhang, C. McGregor, L. Steere, B. H. Frederick, C. T. Liu, L. Whitesell, L. E. Cowen, *ACS Chemical Biology* **2020**, *15*, 1930-1941.
[13] D. Ding, Y. Zhao, Q. Meng, D. Xie, B. Nare, D. Chen, C. J. Bacchi, N. Yarlett, Y.-K. Zhang, V. Hernandez, Y. Xia, Y. Freund, M. Abdulla, K.-H. Ang, J. Ratnam, J. H. McKerrow, R. T. Jacobs, H. Zhou, J. J. Plattner, *ACS Med. Chem. Lett.* **2010**, *1*, 165-169.
[14] J. W. Tomsho, A. Pal, D. G. Hall, S. J. Benkovic, *ACS Med. Chem. Lett.* **2012**, *3*, 48-52.
[15] K. Kunihiro, L. Dumais, G. Lafitte, E. Varvier, L. Tomas, C. S. Harris, *Adv. Synth. Catal.* **2018**, *360*, 2757-2761.
[16] M. P. Wiesenfeldt, Z. Nairoukh, W. Li, F. Glorius, *Science* **2017**, *357*, 908-912.
[17] S. Kim, F. Loose, M. J. Bezdek, X. Wang, P. J. Chirik, *J. Am. Chem. Soc.* **2019**, *141*, 17900-17908.
[18] a) M. Wollenburg, D. Moock, F. Glorius, *Angew. Chem. Int. Ed.* **2019**, *58*, 6549-6553; b) D. Moock, M. P. Wiesenfeldt, M. Freitag, S. Muratsugu, S. Ikemoto, R. Knitsch, J. Schneidewind, W. Baumann, A. H. Schäfer, A. Timmer, M. Tada, M. R. Hansen, F. Glorius, *ACS Catal.* **2020**, *10*, 6309-6317; c) M. P. Wiesenfeldt, T. Knecht, C. Schlepphorst, F. Glorius, *Angew. Chem. Int. Ed.* **2018**, *57*, 8297-8300.
[19] Bruker AXS (**2019**) *APEX3 Version 2019.1-0, SAINT Version 8.40A and SADABS Bruker AXS area detector scaling and absorption correction Version 2016/2*, Bruker AXS Inc., Madison, Wisconsin, USA.
[20] G. Sheldrick, *Acta Crystallogr. A* **2015**, *71*, 3-8.
[21] G. Sheldrick, *Acta Crystallogr. C Struct. Chem.* **2015**, *71*, 3-8.
[22] Bruker AXS (**1998**) *XP – Interactive molecular graphics, Version 5.1*, Bruker AXS Inc., Madison, Wisconsin, USA.
